# Intron turnover of *slc26a1* and *slc26a2* and convergence of intron insertion sites

**DOI:** 10.1038/s41598-025-15147-w

**Published:** 2025-08-16

**Authors:** Kota Torii, Chihiro Ota, Ayumi Nagashima, Masaki Kajikawa, Akira Kato

**Affiliations:** 1 School of Life Science and Technology, Tokyo Institute of Technology, Yokohama, Japan; 2 School of Life Science and Technology, Institute of Science Tokyo, Yokohama, Japan

**Keywords:** Intron turnover, Intron gain, Ray-finned fish, Convergent evolution, Transposable element, Molecular evolution, Evolutionary biology

## Abstract

Intron gain and loss are rare events in vertebrates; however, comparative genome analysis of elephant sharks, tetrapods, and teleosts revealed a higher level of intron turnover in teleosts. *slc26a1* and *slc26a2* are members of the anion-exchanger gene family. Human, zebrafish, and Japanese pufferfish *slc26a1* consist of two, two, and seven exons, respectively, and *slc26a2*, two, three, and four exons, respectively. To better understand intron turnover in teleosts, we analyzed the exon–intron organization of *slc26a1* and *slc26a2* in 81 vertebrates, including 62 ray-finned fish. In most Eurypterygii, which comprise the majority of the Neoteleostei and include Acanthomorpha, Aulopiformes, and Myctophiformes, *slc26a1* and *slc26a2* have seven and four exons, respectively, whereas those of most other ray-finned fishes consist of two and three exons, respectively, suggesting that intron gain occurred in both *slc26a1* and *slc26a2* of the Eurypterygii ancestor. In addition, notothenioid *slc26a2* has six exons, suggesting that two introns were inserted into the notothenioid ancestor. The two newly acquired introns in the notothenioid consist of transposon-like sequences, suggesting that they were generated via transposon insertion. The positions of some of the newly acquired introns of *slc26a1* and *slc26a2* in Eurypterygii are identical or very close to those of other *slc26* members. These results demonstrate the lineage-specific intron gains of *slc26a1* and *slc26a2* in ray-finned fish and convergence at the insertion sites of some of the newly acquired introns.

## Introduction

Spliceosomal introns, hereafter referred to as introns, are present in eukaryotic nuclear genes and contribute to diverse gene functions. The origin of introns in eukaryotes is ancient, and many introns present in the genomes of extant species originate from relatively old eukaryotic ancestors^1,2^. In early eukaryotes, introns may have been present as selfish elements, but they later gained many functions independently in different eukaryotic lineages, and a wide range of functions are thought to have been inherited by modern species^1^. Intron sequences are transcribed to RNA and are involved in the regulation of splicing, transcription rate, nuclear export, RNA stability, and alternative splicing^1,3–6^, as well as sources of non-coding RNAs such as microRNAs^1,7^. Introns also function as DNA molecules in the nuclear genome. The intron sequences in the genome regulate gene expression via transcriptional regulation as cis-regulatory elements^1,4,6^, control chromatin assembly^4,8^, enhance the efficiency of natural selection^4,9^, and serve as sources of new genes^4,10,11^. In eukaryotes, intron-rich and intron-poor species are interspersed, and there are conserved intron positions between widely diverged species^2,12,13^. These observations suggest that ancestral eukaryotes are intron-rich and that lineage-specific losses affect the exon–intron structure of genes in extant species^2^. Relatively new intron gains and losses are specific to each lineage. Mechanisms of intron loss include reverse transcriptase-mediated intron loss^2,14,15^ and genomic deletion^2^. The mechanisms of intron gain include intron transposition, transposon insertion, tandem genomic duplication, intron gain during double-strand break repair, insertion of a group II intron, intron transfer, and intronization^2,16^.

Several studies comparing eukaryotic genes have shown that specific intron locations (10–40%) are conserved among eukaryotes and that the number and placement of most introns are dynamic during evolution^17^. However, in vertebrates, analyses of the whole-genome sequences of vertebrate species have shown that intron turnover is low^18^. Genomic comparisons between the Japanese pufferfish and spotted green pufferfish indicated very low levels of intron turnover in these lineages^19^. Subsequent analysis using cartilaginous fish as an outgroup confirmed that intron turnover was low when comparing cartilaginous and tetrapod genome sequences, but comparison of cartilaginous and teleost genome sequences revealed high intron turnover in the teleost genome^18,20^. High intron turnover can potentially contribute to the phenotypic diversity of teleosts; however, it remains unclear why teleosts have a high intron turnover rate and whether high intron turnover contributes to phenotypic diversity^18^.

Vertebrate body fluids contain the major inorganic anions such as Cl^−^, HCO_3_^−^, phosphate, and sulphate. Anion homeostasis in body fluids is handled by anion channels and anion transporter families. The solute carrier 26 (Slc26) is one of the anion transporter families and comprises 11 members, Slc26a1-a11, in mammals^21,22^ (Note that, in this article, protein name abbreviations of all species are shown with the first letter capitalized, and gene names of all species are shown as lowercase and italicized).　Recently, we identified a novel member of Slc26, Slc26a12, which is widely present in coelacanths, amphibians, various reptiles, and birds but not in cartilaginous fishes, ray-finned fishes, most turtles, some lineages of birds, and mammals^23^. In species that possess Slc26a12 gene (*slc26a12*), *slc26a12* and *slc26a2* are always tandemly present at the same locus on the same chromosome. Since *slc26a12* is present in coelacanths, tetrapods that lack it may have secondarily lost *slc26a12*. Cartilaginous and ray-finned fishes lack *slc26a12*, and a jawless fish, inshore hagfish, possess a gene similar to *slc26a12*. Therefore, it is also possible that cartilaginous and ray-finned fishes also secondarily lost *slc26a12*, but no clear evidence has been provided^23^. As physiological functions, the Slc26 family are involved in sulfate transport^24,25^, bicarbonate secretion and Cl^−^ absorption by the digestive tract^26–28^, Cl^−^ reabsorption in the kidney^29,30^, Cl^−^ secretion in the stomach^29,30^, oxalate efflux^31^, and auditory organ function^32–34^. Slc26 proteins share 12 transmembrane regions and a sulfate transporter anti-sigma factor antagonist (STAS) domain in the intracellular carboxy-terminal region^21,22^.

Slc26a1, also known as sulfate anion transporter 1 (Sat-1), is an Na^+^-independent sulfate transporter found on the basolateral membrane of intestinal and renal epithelial cells and hepatocytes that transports sulfate between body fluids and the cytoplasm in mammals^35^. Study of a patient presenting with painful perichondritis, hyposulfatemia, and renal sulfate wasting revealed a mutation in the human *slc26a1*^25^. Slc26a2 was first isolated by positional cloning of diastrophic dysplasia and is also called diastrophic dysplasia sulfate transporter (Dtdst)^24^. Various *slc26a2* mutations have been found in chondrodysplasia syndromes, and the analysis of mice expressing *slc26a2* mutants has revealed skeletal abnormalities, decreased chondrocyte proliferative activity, and decreased sulfate absorption into chondrocytes^21,36^. Slc26a1 has also been isolated from rainbow trout, Japanese eel, and elephant sharks^37–39^. It is localized in the basolateral membrane of proximal tubules and exhibits sulfate transport activity when heterogeneously expressed in *Xenopus laevis* oocytes. In the Japanese eel, a euryhaline species, the kidney functions to retain sulfate during freshwater acclimation and to excrete sulfate during seawater acclimation. Slc26a1 is thought to contribute to both freshwater and seawater acclimation by increasing the sulfate permeability of the basolateral membrane of the proximal tubule^39–41^. When *slc26a2* was knocked down in zebrafish, significant defects were observed in otolith patterns, semicircular canal morphology, and lateral neuromast distribution in morphants, indicating that this gene is important for auditory development. Expression of *slc26a2* has also been observed in the proximal tubules of the pronephric duct in zebrafish embryos^42^.

The exon–intron structure of the *slc26* genes is conserved within this subfamily. The protein-coding regions of the mammalian *slc26a1* and *slc26a2* genes are encoded by two exons^24,43–46^, and the tetrapod *slc26a12* genes are encoded by two exons^23^. In contrast, the coding region of the other *slc26* genes consists of approximately 20 exons^47–50^. In our previous study on the Slc26 family in pufferfish and zebrafish, we found diversity in the number of exons in *the slc26a1* and *slc26a2* genes. *slc26a1* has three exons in zebrafish and seven exons in Japanese pufferfish, whereas *slc26a2* has three exons in zebrafish and four exons in Japanese pufferfish. Therefore, *slc26a1* and *slc26a2* are good examples for studying intron turnover in vertebrates. In the present study, we analyzed the exon–intron structure of these genes using the genome databases of 81 vertebrate species, including 62 ray-finned fish. These results suggested that intron insertions occurred in both *slc26a1* and *slc26a2* in the ancestor of Eurypterygii. Further analysis revealed that *slc26a2* in notothenioids has a six-exon structure because of the relatively recent acquisition of two introns and that the newly acquired intron is homologous to transposon-like sequences. These results provide a useful example for understanding high intron turnover in teleosts.

## Methods

### Identification of orthologs for slc26a1, slc26a2, and slc26a12

First, we collected amino acid and cDNA sequences of Slc26a1 and Slc26a2 from humans, western clawed frogs, zebrafish, Japanese pufferfish, three-spined sticklebacks, and Japanese medaka, and Slc26a12 from Western clawed frogs. Using these sequences as queries, BLASTp and tBLASTn analyses were performed against the protein and genome databases in the NCBI (https://blast.ncbi.nlm.nih.gov)^51^ and ENSEMBL (https://www.ensembl.org)^52^ databases of the sequences listed in Table 1 to collect the sequences expected to be *slc26a1*, *slc26a2*, and *slc26a12*. Some of these genes were manually annotated registered them as third-party annotations (TPA) to the DDBJ (BR002474–BR002489 and BR002490–BR002502). The collected amino acid sequences for Slc26a1, Slc26a2, and Slc26a12 were aligned using ClustalW software (https://www.genome.jp/tools-bin/clustalw)^53^ and a phylogenetic tree was constructed to confirm that the nomenclature was correct. Due to weak evidence of a direct orthologous relationship between these genes and related genes in jawless vertebrates, we designated them as Slc26a2-like and Slc26a12-like.

**Table 1:** Accession numbers of *slc26a1*, *slc26a2*, and *slc26a12* in various vertebrate species.

Species	Genome assembly	Reference	*slc26a1*	*slc26a2*	*slc26a12*
Inshore hagfish (*Eptatretus burgeri*)	GCA_900186335.2	^69^	Not present	ENSEBUT00000017110.1	ENSEBUT00000009389.1
Sea lamprey (*Petromyzon marinus*)	GCA_010993605.1	^70^	Not present	XM_032950720.1	Not present
Elephant shark (*Callorhinchus milii*)	GCA_018977255.1	^20^	XM_007907698.2	XM_007912295.2	Not present
Little skate (*Leucoraja erinacea*)	GCA_028641065.1	^71^	XM_055646986.1	XM_055643019.1	Not present
Smaller spotted catshark (*Scyliorhinus canicula*)	GCA_902713615.1	^72^	XM_038792328.1	XM_038795555.1	Not present
Human (*Homo sapiens*)	GCA_000001405.29	^73^	NM_022042.4	NM_000112.4	Not present
Dog (*Canis lupus*)	GCA_014441545.1	^74^	XM_038662138.1	XM_038663660.1	Not present
Nine-banded armadillo (*Dasypus novemcinctus*)	GCA_030445035.2	^75^	XM_058301884.1	XM_071210691.1, BR002500	Not present
African savanna elephant (*Loxodonta africana*)	GCA_000001905.1	^76^	XM_010591446.2	XM_023552693.1	Not present
Gray short-tailed opossum (*Monodelphis domestica*)	GCA_027887165.1	^77^	XM_007496877.3	XM_007473862.3	Not present
Platypus (*Ornithorhynchus anatinus*)	GCA_004115215.4	^78^	XM_007672822.4	XM_007673322.3	Not present
Chicken (*Gallus gallus*)	GCA_016699485.1	^79^	XM_004949339.5	NM_001389738.2	XM_046900518.1
American alligator (*Alligator mississippiensis*)	GCA_030867095.1	^80^	XM_006259257.3	XM_014597637.3	XM_059733419.1
Painted turtle (*Chrysemys picta*)	GCA_000241765.5	^81^	XM_005308972.3	XM_005298017.3	Not present
Green anole (*Anolis carolinensis*)	GCA_000090745.2	^82^	XM_008114587.2	XM_003217337.3	XM_003217349.3
Western clawed frog (*Xenopus tropicalis*)	GCA_000004195.4	^83^	XM_002935465.3	XM_002943149.5	XM_012959807.2
Two-lined caecilian (*Rhinatrema bivittatum*)	GCA_901001135.2	^84^	XM_029602309.1	XM_029583273.1	XM_029583269.1
West african lungfish (*Protopterus annectens*)	GCA_019279795.1	^85^	XM_044061139.1	XM_044069214.1	Not present
Coelacanth (*Latimeria chalumnae*)	GCA_000225785.1	^86^	XM_006011442.1	XM_006003784.2	XM_006003789.1
Reedfish (*Erpetoichthys calabaricus*)	GCA_900747795.4	Vertebrate Genomes Project, Wellcome Sanger Institute	XM_028802590.2	XM_028812907.2	Not present
Gray bichir (*Polypterus senegalus*)	GCA_016835505.1	^87^	XM_039750381.1	XM_039774862.1	Not present
Sterlet (*Acipenser ruthenus*)	GCA_902713425.2	^88^	XM_034015637.3	XM_058997183.1	Not present
Spotted gar (*Lepisosteus oculatus*)	GCA_000242695.1	^89^	XM_006627158.2	XM_006632082.2	Not present
Tarpon (*Megalops atlanticus*)	GCA_019176425.1	^90^	MATL_G00085360	MATL_G00171530	Not present
West African bonefish (*Albula goreensis*)	GCA_022829145.1	^90^	KAI1881981.1	KAI1899047.1	Not present
European eel (*Anguilla anguilla*)	GCA_013347855.1	^90^	XM_035389685.1	XM_035410385.1	Not present
European conger (*Conger conger*)	GCA_029692045.1	^90^	KAJ8260901.1	KAJ8282142.1	Not present
Asian bonytongue (*Scleropages formosus*)	GCA_900964775.1	^91^	XM_029252658.1	XM_018733194.2	Not present
Atlantic herring (*Clupea harengus*)	GCA_900700415.2	^92^	XM_012837634.3	XM_031568942.2	Not present
Milkfish (*Chanos chanos*)	GCA_902362185.1	Vertebrate Genomes Project, Wellcome Sanger Institute	XM_030769916.1	XM_030764670.1	Not present
Fathead minnow (*Pimephales promelas*)	GCA_016745375.1	^93^	XM_039688210.1	XM_039654486.1	Not present
Zebrafish (*Danio rerio*)	GCA_000002035.4	^94^	XM_005161206.4	XM_680022.5	Not present
Mexican tetra (*Astyanax mexicanus*)	GCA_023375975.1	^95^	XM_049466379.1	XM_007246413.4	Not present
Electric eel (*Electrophorus electricus*)	GCA_013358815.1	^96^	XM_027028493.2	XM_027003556.2	Not present
Channel catfish (*Ictalurus punctatus*)	GCA_001660625.3	^97^	XM_017493276.3	XM_017475076.3	Not present
Northern pike (*Esox lucius*)	GCA_011004845.1	^98^	XM_010877286.4	XM_010898954.5	Not present
Rainbow trout (*Oncorhynchus mykiss*)	GCA_013265735.3	^99^	XM_021602834.2	XM_036970229.1	Not present
Greater argentine (*Argentina silus*)	GCA_951799395.1	Tree of Life Programme, Wellcome Sanger Institute	BR002490	BR002495	Not present
European smelt (*Osmerus eperlanus*)	GCA_963692335.1	Darwin Tree of Life Project, Wellcome Sanger Institute	XM_062478671.1	XM_062476172.1	Not present
Ayu (*Plecoglossus altivelis*)	GCA_021733525.1	^100^	BR002474	BR002477	Not present
Large-eye snaggletooth (*Borostomias antarcticus*)	GCA_949987545.1	^101^	BR002475	BR002478	Not present
Peladilla (*Aplochiton taeniatus*)	GCA_017639685.1	Vertebrate Genomes Project, G10K Consortium, Gene Myers Lab	BR002476	BR002479	Not present
Antarctic jonasfish (*Notolepis coatsorum*)	GCA_963971535.1	Darwin Tree of Life Project, Wellcome Sanger Institute	BR002491	BR002496	Not present
Minispotted lanternfish (*Gymnoscopelus microlampas*)	GCA_963454915.1	Tree of Life Programme, Wellcome Sanger Institute	BR002492	BR002497	Not present
Atlantic cod (*Gadus morhua*)	GCA_902167405.1	^102^	XM_030341376.1	XM_030368538.1	Not present
Bigeye Pacific opah (*Lampris megalopsis*)	GCA_022114975.2	^103^	BR002493	BR002498	Not present
Pinecone soldierfish (*Myripristis murdjan*)	GCA_902150065.1	Vertebrate Genomes Project, Wellcome Sanger Institute, Cambridge University team	XM_030065095.1	XM_030062832.1	Not present
Great blue-spotted mudskipper (*Boleophthalmus pectinirostris*)	GCA_026225935.1	^104^	XM_055153669.1	XM_020925467.2	Not present
Yellowfin tuna (*Thunnus albacares*)	GCA_914725855.1	^101^	XM_044334097.1	XM_044363739.1	Not present
Mandarinfish (*Synchiropus splendidus*)	GCA_027744825.1	^105^	XM_053885922.1	XM_053876165.1	Not present
Straightnose pipefish (*Nerophis ophidion*)	GCA_033978795.1	^106^	XM_061876964.1	XM_061891969.1	Not present
Broad-nosed pipefish (*Syngnathus typhle*)	GCA_033458585.1	^106^	XM_061294257.1	XM_061286189.1	Not present
Common seadragon (*Phyllopteryx taeniolatus*)	GCA_024500385.1	^107^	XM_061799645.1	XM_061787208.1	Not present
Big-belly seahorse (*Hippocampus abdominalis*)	GCA_018466805.1	^108^	BR002501	BR002502	Not present
Swamp eel (*Monopterus albus*)	GCA_001952655.1	^109^	XM_020609878.1	XM_020594048.1	Not present
Northern snakehead (*Channa argus*)	GCA_033026475.1	^110^	XM_067484895.1	XM_067519065.1	Not present
Japanese lates (*Lates japonicus*)	GCA_033238685.1	^111^	GLD55153	GLD70538	Not present
Greater amberjack (*Seriola dumerili*)	GCA_002260705.1	^112^	XM_022766577.1	XM_022760335.1	Not present
Turbot (*Scophthalmus maximus*)	GCA_022379125.1	^113^	XM_035607435.2	XM_035649711.2	Not present
Indian glassy fish (*Parambassis ranga*)	GCA_900634625.2	Vertebrate Genomes Project, Wellcome Sanger Institute, Cambridge University team	XM_028417908.1	XM_028416832.1	Not present
Clown anemonefish (*Amphiprion ocellaris*)	GCA_022539595.1	^114^	XM_023262045.3	XM_023287835.3	Not present
Jewelled blenny (*Salarias fasciatus*)	GCA_902148845.1	Vertebrate Genomes Project, Wellcome Sanger Institute, Cambridge University team	XM_030085450.1	XM_030111117.1	Not present
Nile tilapia (*Oreochromis niloticus*)	GCA_001858045.3	^115^	XM_005451405.2	XM_019355496.2	Not present
Japanese medaka hdrr (*Oryzias latipes*)	GCA_002234675.1	^116^	XM_011481933.3	XM_011480560.2	Not present
Bonti rainbowfish (*Telmatherina bonti*)	GCA_933228915.1	Vertebrate Genomes Project, Wellcome Sanger Institute, Cambridge University team	BR002494	BR002499	Not present
Mangrove rivulus (*Kryptolebias marmoratus*)	GCA_001649575.2	^117^	XM_017410759.3	XM_025008958.2	Not present
Platyfish (*Xiphophorus maculatus*)	GCA_002775205.2	^118^	XM_014470576.2	XM_005808628.2	Not present
Humphead wrasse (*Cheilinus undulatus*)	GCA_018320785.1	^119^	XM_041810300.1	XM_041797676.1	Not present
Chinese perch (*Siniperca chuatsi*)	GCA_020085105.1	^120^	XM_044173457.1	XM_044205709.1	Not present
European seabass (*Dicentrarchus labrax*)	GCA_905237075.1	^121^	XM_051412960.1	XM_051419895.1	Not present
Large yellow croaker (*Larimichthys crocea*)	GCA_000972845.2	^122^	XM_019259540.2	XM_019255190.2	Not present
Gilthead seabream (*Sparus aurata*)	GCA_900880675.1	^123^	XM_030436761.1	XM_030396978.1	Not present
Japanese pufferfish (*Takifugu rubripes*)	GCA_901000725.2	^124^	XM_029838056.1	XM_003978227.3	Not present
Three-spined stickleback (*Gasterosteus aculeatus*)	GCA_016920845.1	^125,126^	XM_040197011.1	XM_040175264.1	Not present
Tristan klipfish (*Bovichtus diacanthus*)	GCA_943590825.1	^63^	BR002480	BR002485	Not present
Patagonian blennie (*Eleginops maclovinus*)	GCA_025505465.1	^127^	XM_063888790.1, BR002481	XM_063877144.1, BR002486	Not present
Antarctic spiny plunderfish (*Harpagifer antarcticus*)	GCA_902827135.1	^63^	BR002482	BR002487	Not present
Blackfin icefish (*Chaenocephalus aceratus*)	GCA_023974075.1	^128^	BR002483	BR002488	Not present
Marbled rockcod (*Notothenia rossii*)	GCA_943590865.1	^63^	BR002484	BR002489	Not present
White-fin plunderfish (*Pogonophryne albipinna*)	GCA_028583405.1	^129^	KAJ4926520.1	KAJ4921587.1	Not present
Emerald rockcod (*Trematomus bernacchii*)	GCA_902827165.1	^63^	XM_034123481.1	XM_034135411.1	Not present

The evolutionary history was inferred by the Maximum Likelihood method and Le and Gascuel (LG) model^54^ using IQ-TREE^55^ (https://www.hiv.lanl.gov/content/sequence/IQTREE/iqtree.html). The percentages of trees in which the associated taxa were clustered together were generated from 1000 ultrafast bootstrap approximation^56^. The alignment had 167 sequences with 861 columns, 815 distinct patterns, 695 parsimony-informative, 81 singleton sites, and 85 constant sites. The resulting Newick format tree was visualized using MEGA11^57^.

### Estimation of exon–intron organization of slc26a1, slc26a2, and slc26a12

Using the annotated amino acid sequences of Slc26a1, Slc26a2, and Slc26a12 and their coding regions in the cDNA sequences as queries, we performed tBLASTn and BLASTn analyses against the genome databases of the species listed in Table 1 in NCBI (https://blast.ncbi.nlm.nih.gov)^58^ and ENSEMBL (https://www.ensembl.org) to confirm the exons encoding the proteins and the introns that separate the exons (Supplementary Fig. S1). Introns were predicted according to the gt-ag rule, and the exon–intron organization of each gene was confirmed. For species whose genome analysis results are shown in the NCBI Genome Data Viewer, we referred to the RNA-seq exon coverage (aggregate, filtered) results to visually confirm whether the prediction of the exon–intron region was reasonable^59^ (Supplementary Figs. S2, S3). Some of the annotated amino acid sequences contained amino-terminal extensions that are not well conserved in other species. Exons encoding species-specific amino-terminal sequences were excluded from the analysis in this study, and only exons encoding amino acid sequences that are conserved among species were included in the analysis.

### Comparison of the sites of intron insertion in slc26a1, slc26a2, and slc26a12

The amino acid sequences of Slc26a1 (human, European smelt, peladilla, and Atlantic cod), Slc26a2 (human, zebrafish, European smelt, Atlantic cod, big-belly seahorse, and emerald rockcod), Slc26a12 (chicken), Slc26a2-like (inshore hagfish and sea lamprey), Slc26a12-like (sea lamprey), and Slc26a3 (human) were aligned using ClustalW software. The intron insertion sites of each protein are marked. Introns were classified by checking where they were inserted before the first, second, and third bases of the codon and labeled as 0, 1, and 2, respectively. A schematic diagram was created from the alignment obtained (Supplementary Fig. S4).

### Comparison sequences between introns and putative transposable elements

Using the sequences of the newly acquired introns 3 and 5 in the Notothenia *slc26a2* as queries, BLASTn analysis was performed on the Notothenia genome database in NCBI (https://blast.ncbi.nlm.nih.gov)^58^ to identify and classify sites that are homologous to other loci. Sequences homologous to multiple loci were designated putative transposable elements (NTEs).

### Synteny analyses

Representative *slc26a1*, *slc26a2*, and *slc26a12* shown in Table 1 were selected for synteny analysis, and information on the neighboring genes of each gene was collected using NCBI genome Data viewer (https://www.ncbi.nlm.nih.gov/genome/gdv/)^51^ and ENSEMBL (https://www.ensembl.org)^52^, and the order and orientation of each gene were summarized in a figure.

### Analyses of the ohnologs generated by the teleost-specific whole-genome duplication in ancestral teleosts

All teleosts examined harbored one *slc26a1* and one *slc26a2*. In other words, we could not find any species that harbored both ohnologs derived from teleost-specific whole-genome duplications. To confirm whether teleost *slc26a1* and *slc26a2* obtained in this study were derived from one of the ohnologs generated by the teleost-specific whole-genome duplication or from both ohnologs, we performed a series of analyses. We selected genes that existed in loci close to *slc26a1* and *slc26a2* and that conserved an ohnolog in another locus. The amino acid sequences of these genes were collected, and phylogenetic trees were generated for each gene using ClustalW and IQ-TREE, as described above. The alignment had 11 sequences with 1314 columns, 372 distinct patterns, 182 parsimony-informative, 136 singleton sites, and 996 constant sites. We then assessed whether genes in loci close to *slc26a1* and *slc26a2* in the phylogenetic tree were clustered in one branch or distributed in two branches and determined the ohnologous relationship between extant *slc26a1* and *slc26a2* in teleosts.

## Results

### Exon–intron structures of slc26a1, slc26a2, and slc26a12 in tetrapods, lobe-finned fishes, cartilaginous fishes, and jawless fishes

**Fig. 1:**
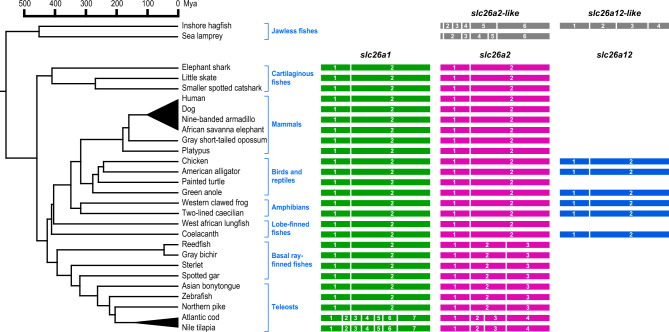
Exon–intron organization of *slc26a1*, *slc26a2*, and *slc26a12* in vertebrates. Results for 28 species are shown. Exons are indicated by filled-in colored boxes and numbers, and introns are indicated by white vertical bars (*right*). Divergence times of species were retrieved from the TimeTree database (http://www.timetree.org/)^62^ and shown on the *left*. The accession number of each sequence is summarized in Table 1.

**Fig. 2:**
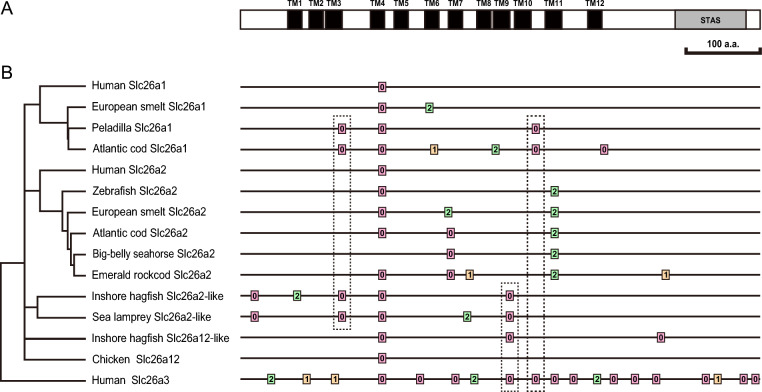
Comparison of intron positions among jawed vertebrate *slc26a1*, *slc26a2*, and *slc26a12*, related genes of lamprey had hagfish, and human *slc26a3*. (**A**) Schematic representation of the domain structure of human Slc26a1 protein. Transmembrane domains and the STAS (Sulfate Transporter and Anti-Sigma factor antagonist) domain are indicated by black and gray boxes, respectively. (**B**) Position of intron insertion sites in comparison with Slc26a1 domain structure shown in (**A**). Horizontal bars indicate polypeptide of each protein. Boxes indicate the site of intron insertion. The numbers indicate the position of intron insertion within each codon.

**Fig. 3:**
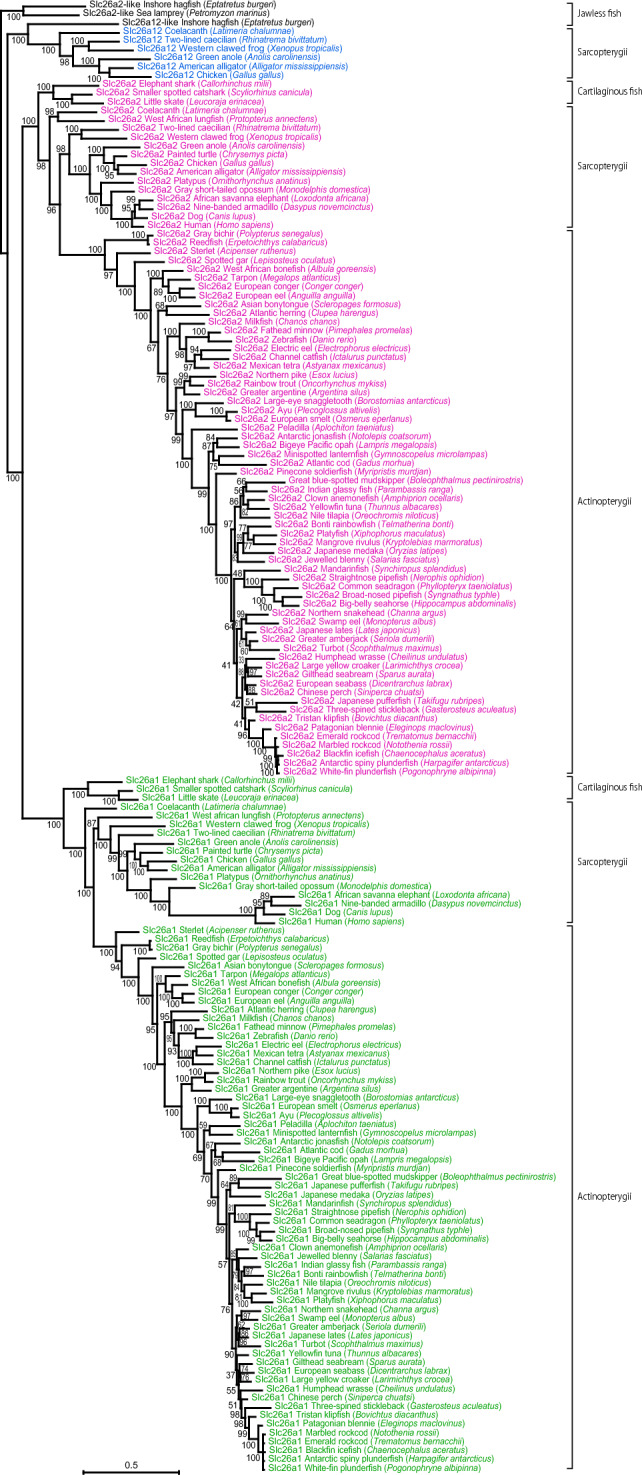
Phylogenetic analysis of Slc26a1, Slc26a2, and Slc26a12 in vertebrates**.** The amino acid sequences of Slc26a1, Slc26a2, and Slc26a12 in jawed vertebrates were aligned with Slc26a2-like and Slc26a12-like in jawless fishes using ClustalW software and a phylogenetic tree was constructed by the maximum-likelihood method using IQ-TREE^55^. Numbers indicate bootstrap values. The accession numbers of the amino-acid sequences used in this study are listed in Table 1.

Before describing the results for ray-finned fish, the exon–intron structures of *slc26a1*, *slc26a2*, and *slc26a12* in tetrapods, lobe-finned fish, cartilaginous fishes, and jawless fishes were analyzed and compared (Figs. 1, 2). The validity of the exon–intron structure prediction was confirmed by comparing the sequence conservation using tBLASTn (Supplementary Fig. S1) and the RNA-seq exon coverage (aggregate, filtered) results shown in the NCBI Genome Data Viewer (Supplementary Figs. S2, S3)^59^. The correct name of each gene was confirmed by molecular phylogenetic analysis (Fig. 3). The species used in this study are listed in Table 1. Among tetrapods, mammals have *slc26a1* and *slc26a2*, whereas birds, reptiles, and amphibians have *slc26a1*, *slc26a2*, and *slc26a12*^23^. In the examined placental mammals, marsupials, monotremes, birds, reptiles, and amphibians, the protein-coding regions of *slc26a1* and *slc26a2* consisted of two exons, and the positions of these introns were conserved (Figs. 1, 2). In amphibians, reptiles, and birds, the protein-coding region of *slc26a12* has two exons, and the positions of the introns were conserved with those of tetrapod *slc26a1* and *slc26a2* (Figs. 1 and 2).

Lobe-finned fish, such as coelacanths, have *slc26a1*, *slc26a2*, and *slc26a12*, all of which are encoded by two protein-coding exons, and the positions of the introns were conserved with those of the tetrapod orthologs (Fig. 1). Cartilaginous fish have both *slc26a1* and *slc26a2*. In the holocephalans, sharks, and rays examined, the protein-coding regions of *slc26a1* and *slc26a2* contained two exons, and the positions of the introns were conserved with those of the orthologs in tetrapods and lobe-finned fish (Fig. 1).

The results for jawless fish differed from those for the vertebrate species described above. Hagfish have *slc26a2-like* and *slc26a12-like* genes, whereas lamprey has *slc26a2-like*^23^. In hagfish and lamprey, the protein-coding region of *slc26a2* consists of six exons (Fig. 1). Four of the five introns in *slc26a2-like* were conserved between hagfish and lamprey, whereas intron 2 of hagfish *slc26a2-like* and intron 4 of lamprey *slc26a2-like* were present at unique positions in each gene (Figs. 1 and 2). In hagfish, *slc26a12-like* is encoded by four exons, and the sites of two of the three introns were conserved with those of *slc26a2-like* in hagfish and lamprey (Figs. 1 and 2). The positions of intron 4 of hagfish *slc26a2-like*, intron 3 of lamprey *slc26a2-like*, and intron 1 of hagfish *slc26a12-like* were conserved with those of intron 1 of *slc26a1*, *slc26a2*, and *slc26a12* in tetrapods, lobe-finned fish, and cartilaginous fish (Fig. 2).

### Exon–intron structures of slc26a1 and slc26a2 in basal ray-finned fishes and teleosts in Eloposteoglossocephala, Ostariophysi, Argentiniformes, Esociformes, and Salmoniformes

Since ray-finned fish have *slc26a1* and *slc26a2* but not *slc26a12,* we analyzed the exon–intron structures of the protein-coding regions of these two genes in ray-finned fish species. Here, the results of basal ray-fined fishes and teleosts other than Eurypterygii, Galaxiiformes, Osmeriformes, and Stomiiformes are described.

In basal ray-finned fishes, such as polypterids, sturgeonids, and gariforms, the protein-coding regions of *slc26a1* and *slc26a2* had two and three exons, respectively (Fig. 1). The positions of intron 1 of *slc26a1* and *slc26a2* in these species were conserved with those of intron 1 of *slc26a1* and *slc26a2* in cartilaginous fish, lobe-finned fish, and tetrapods. Intron 2 of *slc26a2* in basal ray-finned fish was present at a unique position and was conserved between *slc26a2* of basal ray-finned fish.

**Fig. 4:**
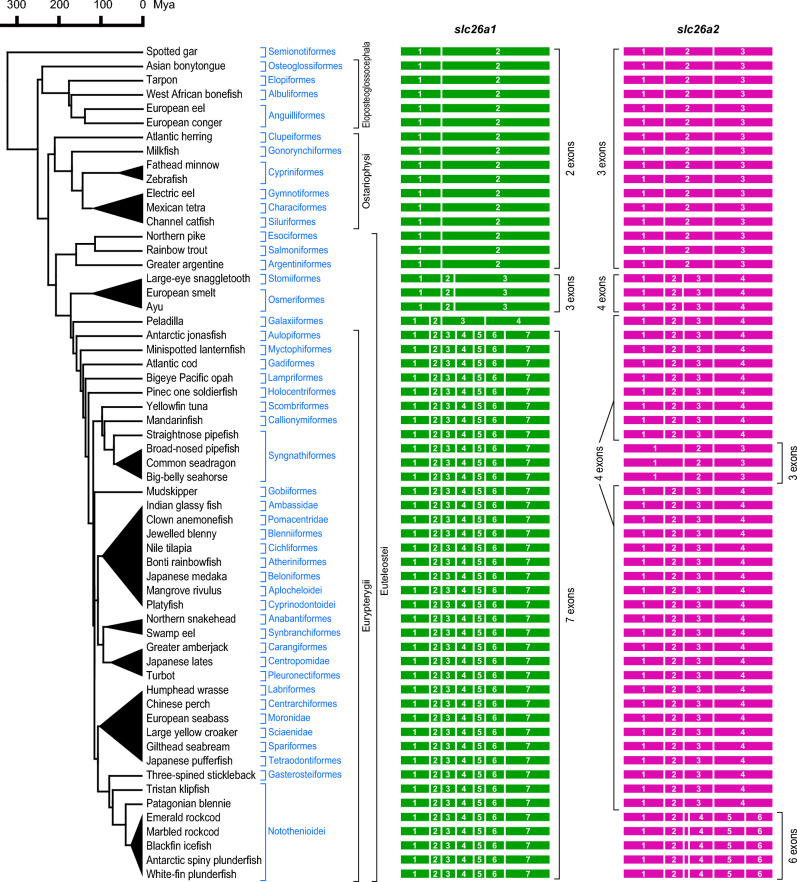
Exon–intron organization of *slc26a1* and *slc26a2* in spotted gar and teleosts. Results from 59 teleost species from 47 orders/suborders/families are presented and compared with those of the spotted gar, a basal ray-finned fish that is not a teleost. Exons are indicated by filled colored boxes and numbered, and introns are indicated by white vertical bars (*right*). Divergence times of species were retrieved from the TimeTree database (http://www.timetree.org/)^62^ and shown on the *left*. The tree topology between Osteoglossiformes (Asian bonytongue), Elopiformes (tarpon), Albuliformes (West African bonefish), and Anguilliformes (European eel and European conger) was drawn based on the recent study by Parey et al.^90^. The tree topology between Galaxiiformes (peladilla) and Eurypterygii was drawn based on the recent study by Lavoué et al.^60^ and Near et al.^61^. The accession number of each sequence is summarized in Table 1.

In 15 teleost species in 13 orders/suborders/families (Eloposteoglossocephala including Osteoglossiformes, Elopiformes, Albuliformes, Anguilliformes; Ostariophysi including Clupeiformes, Gonorynchiformes, Cypriniformes, Gymnotiformes, Characiformes, Siluriformes; and Argentiniformes, Esociformes, and Salmoniformes), *slc26a1* and *slc26a2* had two and three exons, respectively, and their intron positions were conserved with those of the basal ray-finned fish (Fig. 4). These results suggest that the protein-coding regions of *slc26a1* and *slc26a2* in the common ancestral species of ray-finned fish had two and three exons, respectively, and that *slc26a2* acquired intron 2 in the common ancestor ray-finned fish.

### Intron gain of slc26a1 in Eurypterygii and Galaxiiformes

The preliminary analysis showed that the protein-coding region of *slc26a1* had seven exons in species such as cod, medaka, tilapia, stickleback, and pufferfish. Therefore, we performed a detailed analysis of *slc26a1* in 39 species of 30 orders/suborders/families in Eurypterygii (Aulopiformes; Myctophiformes; and Acanthomorpha including Gadiformes, Lampriformes, Holocentriformes, Scombriformes, Callionymiformes, Syngnathiformes, Gobiiformes, Ambassidae, Pomacentridae, Blenniiformes, Cichliformes, Atheriniformes, Beloniformes, Aplocheloidei, Cyprinodontoidei, Anabantiformes, Synbranchiformes, Carangiformes, Centropomidae, Pleuronectiformes, Labriformes, Centrarchiformes, Moronidae, Sciaenidae, Spariformes, Tetraodontiformes, Gasterosteiformes, and Notothenioidei). The protein-coding region of *slc26a1* in these 39 species contains seven exons (Fig. 4). The position of intron 2 of *slc26a1* in Eurypterygii species was conserved with that of intron 1 of *slc26a1* in cartilaginous fish, tetrapods, lobe-finned fish, basal ray-finned fish, and teleost species other than Eurypterygii, as described in the chapter above (Figs. 1, 2, and 4). The positions of introns 1, 3, 4, 5, and 6 of *slc26a1* were unique and conserved among the Eurypterygii (Figs. 2 and 4).

The protein-coding region of *slc26a1* in peladilla (*Aplochiton taeniatus*, Galaxiiformes), which is relatively close to Eurypterygii based on the evolutionary analyses by Lavoué et al.^60^ and Near et al.^61^, consists of four exons (Figs. 2 and 4). The positions of introns 1, 2, and 3 of peladilla *slc26a1* were conserved with those of introns 1, 2, and 5 of Eurypterygii *slc26a1*, respectively (Figs. 2 and 4). These results suggest that intron gain occurred in the common ancestor of Eurypterygii and Galaxiiformes and that subsequent intron turnover, such as intron gain or loss, may have occurred in either or both of the ancestral species of Eurypterygii and Galaxiiformes. The estimated divergence date between the lineages is 139–169 million years ago (Mya)^62^.

### Intron gain of slc26a1 in Osmeriformes and Stomiiformes

The protein-coding regions of *slc26a1* in three species belonging to Stomiiformes and Osmeriformes, which are relatively close to Eurypterygii and peladilla, consist of three exons. The position of intron 1 of *slc26a1* in these species was conserved with that of intron 1 of *slc26a1* in basal ray-finned fishes and teleosts other than Eurypterygii, and intron 2 of *slc26a1* in Eurypterygii. In contrast, intron 2 of these three species was inserted at 23 bp from the position of intron 3 in *slc26a1* of Eurypterygii (Fig. 2).

### Intron gain of slc26a2 in Eurypterygii, Galaxiiformes, Osmeriformes, and Stomiiformes

As the preliminary analysis showed that the protein-coding region of *slc26a2* consists of four exons in species such as cod, medaka, stickleback, and pufferfish, we performed a detailed analysis of *slc26a2* in 39 species of 30 orders/suborders/families in Eurypterygii. In species other than Syngnathiformes and Notothenioidei, the protein-coding regions of *slc26a2* consisted of four exons (Fig. 4). The positions of introns 1 and 3 of *slc26a2* in Eurypterygii, other than Syngnathiformes and Notothenioides, were conserved with those of introns 1 and 2 of *slc26a2* in basal ray-finned fish and most teleosts other than Eurypterygii (Figs. 2 and 4). The protein-coding regions of *slc26a2* of the peladilla (Galaxiiformes), which is relatively close to Eurypterygii based on the evolutionary analyses by Lavoué et al.^60^ and Near et al.^61^, also had a four-exon structure similar to that of Eurypterygii (Fig. 4), suggesting that the intron 2 was acquired in the common ancestor of Eurypterygii and Galaxiiformes.

Three species belonging to Stomiiformes and Osmeriformes also contained four exons in the protein coding region of *slc26a2*. However, the intron 2 of these three species was positioned 7 bp away from intron 2 of *slc26a2* in Eurypterygii (Fig. 2, Supplementary Fig. S4).

In some species belonging to Syngnathiformes and Notothenioidei, the protein-coding regions of *slc26a2* consisted of three and six exons, respectively (Fig. 4). Since Syngnathiformes and Notothenioidei belong to Eurypterygii, it was hypothesized that *slc26a2* in these lineages caused intron loss and gain, respectively, as described below.

### Intron loss of slc26a2 in seahorses and some pipefishes

**Fig. 5:**
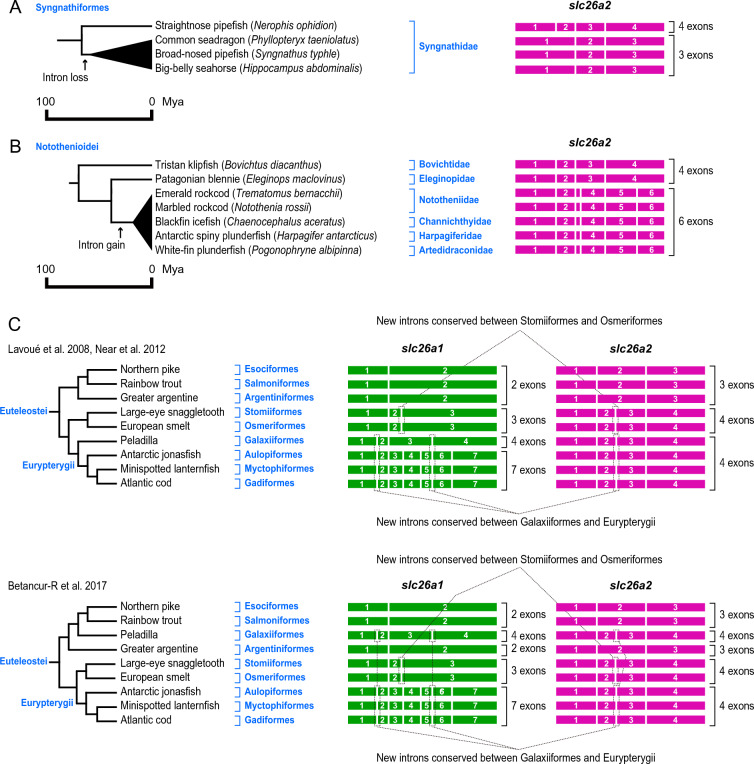
Timing of recent intron turnovers of *slc26a2* in Syngnathiformes and Notothenioidei and two scenarios for the intron turnovers of *slc26a1* and *slc26a2* in Euteleostei. (**A**) Timing of recent intron loss of *slc26a2* in Syngnathiformes. (**B**) Timing of recent intron gain of *slc26a2* in Notothenioidei. In (**A**) and (**B**), arrows indicate timing of recent intron turnovers of *slc26a* in each lineage. Divergence times of species were retrieved from the TimeTree database (http://www.timetree.org/)^62^ and shown on the *left*. (**C**) Two scenarios for the intron turnovers of *slc26a1* and *slc26a2* in Euteleostei. Upper panel indicates a scenario for the intron turnovers based on the evolutionary analyses by Lavoué et al.^60^ and Near et al.^61^, and lower panel indicates that based on the study by Betancur-R et al.^65^.

Analysis of *slc26a2* in the genome database of Syngnathiformes, pipefishes and seahorses, showed that the *slc26a2* of the straightnose pipefish (*Nerophis ophidion*) consisted of four exons, like most other Eurypterygii, but that of three other species, such as the broad-nosed pipefish (*Syngnathus typhle*), common seadragon (*Phyllopteryx taeniolatus*), and big-belly seahorse (*Hippocampus abdominalis*), consisted of three exons (Figs. 4 and 5A). The positions of the two introns of *slc26a2* in these species were conserved with those of introns 2 and 3 of the *slc26a2* in straightnose pipefish and most other Eurypterygii, suggesting that an intron was lost in the common ancestor of broad-nosed pipefish common seadragon, and big-belly seahorse (Fig. 5A).

### Intron gain of slc26a2 in notothenioids

Seven species belonging to the genus Notothenioidei were analyzed. According to Bista et al.^63^, these seven species can be classified into six groups. The phylogenetic relationships of the Notothenioidei species are shown in Fig. 4B. The *slc26a2* of the five notothenioid species (Emerald rockcod *Trematomus bernacchii* and marbled rockcod *Notothenia rossii* in Nototheniidae, blackfin icefish *Chaenocephalus aceratus* in Channichthyidae, Antarctic spiny plunderfish *Harpagifer antarcticus* in Harpagiferidae, and white-fin plunderfish *Pogonophryne albipinna* in Artedidraconidae) had a common six-exon structure, whereas *slc26a2* of the other notothenioid species (Tristan klipfish *Bovichtus diacanthus* in Bovichtidae and Patagonian blennie *Eleginops maclovinus* in Eleginopidae) had a four-exon structure similar to non-notothenioid species in Eurypterygii (Figs. 4 and 5B). The positions of introns 1, 2, and 4 of *slc26a2* in the five notothenioids were conserved with those of introns 1, 2, and 3 of *slc26a2* in the other Eurypterygii, respectively (Fig. 2). This result suggests that introns 3 and 5 of *slc26a2* in the five notothenioids were newly acquired in their common ancestor (Fig. 5B). The estimated divergence date between these five notothenioids and the Patagonian blennie was 17–38 Mya^64^ (Fig. 5B).

### Alternative scenarios for the intron turnovers of slc26a1 and slc26a2 in Euteleostei

Euteleostei is a group of ray-finned fish consisting of Neoteleostei (including Eurypterygii), Esociformes, Salmoniformes, Argentiniformes, Stomiiformes, Osmeriformes, Galaxiiformes, and so on^60,61,65^. There are some hypotheses regarding the evolutionary history of Euteleostei. As aforementioned and shown in Fig. 4, Lavoué et al. ^60^ and Near et al. ^61^ also showed that the above species in Euteleostei consists of three clades: 1) Esociformes, Salmoniformes, and Argentiniformes; 2) Stomiiformes and Osmeriformes; and 3) Galaxiiformes and Neoteleostei (including Eurypterygii). In contrast, Betancur-R et al.^65^ showed that the above species in Euteleostei consist of three clades: 1) Esociformes, Salmoniformes, Argentiniformes, and Galaxiiformes, 2) Stomiiformes and Osmeriformes, and 3) Neoteleostei (including Eurypterygii). The scenarios for the intron turnover of *slc26a1* and *slc26a2* in Euteleostei based on these two hypotheses are summarized in Fig. 5C. *slc26a1* and *slc26a2* have new introns conserved between Galaxiiformes and Eurypterygii. Based on the evolutionary tree by Lavoué et al.^60^ and Near et al.^61^ that places Galaxiiformes close to Neoteleostei, the intron turnover scenario is simple because there is a good agreement between the similarity of the exon–intron structures of *slc26a1* and *slc26a2* and the clade composition of the species. (Fig. 5C, upper panel). However, based on the evolutionary tree of Betancur-R^65^ which places Galaxiiformes close to Esociformes, Salmoniformes and Argentiniformes, the intron turnover scenario is more complex because of the discrepancy between the exon and intron structure of *slc26a1* and *slc26a2* and the clade organization of the species (Fig. 5C, lower panel).

### Origin of newly inserted introns of slc26a2 in notothenioids

Introns 3 and 5 of *slc26a2* in the five notothenioids were assumed to have been acquired relatively recently, as aforementioned and shown in Fig. 5B, indicating that little time has passed since intron insertion, and that they may remain in sequences similar to those at the time of intron insertion. Introns 3 and 5 of *slc26a2* in the five notothenioids showed high sequence similarity, indicating that they were homologous. In contrast, the introns of *slc26a1* and *slc26a2* acquired in the common Eurypterygii ancestor, that is, introns 1, 3, 4, 5, and 6 of *slc26a1* and intron 3 of *slc26a2* in most Eurypterygii species, showed little sequence homology among species, probably because of the accumulation of many neutral substitutions during the approximately 139–169 million years since their divergence after intron insertion. Therefore, the intron 3 and 5 sequences of *slc26a2* in the five notothenioid species were considered useful for analyzing the mechanism of intron gain.

**Fig. 6:**
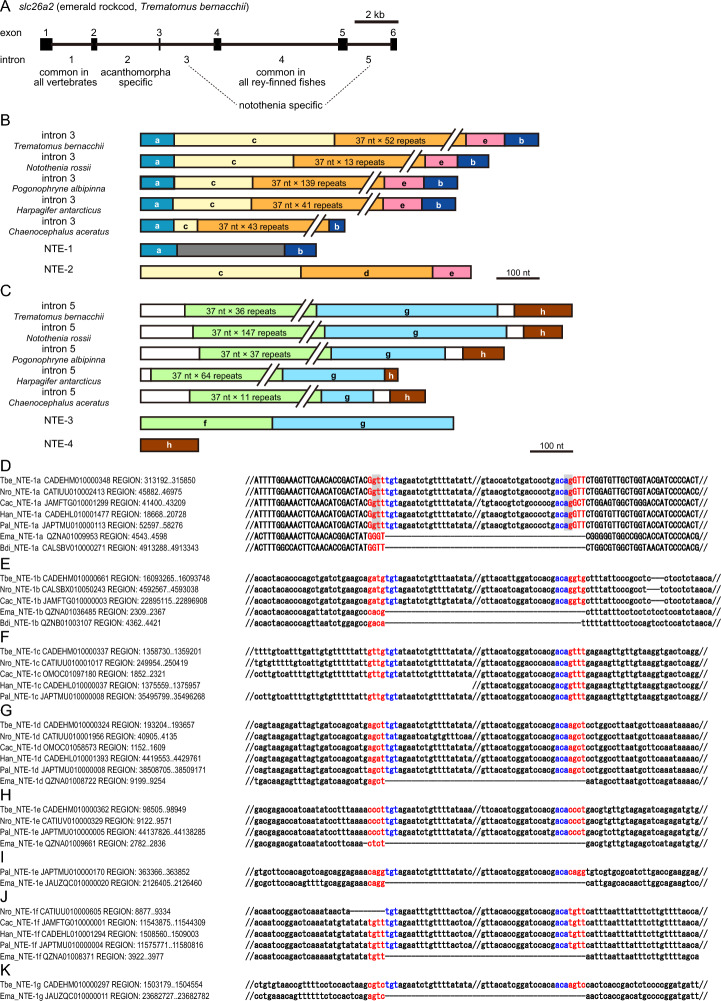
Schematic representation of the primary structure of recently acquired introns in notothenioid *slc26a2* and transposable element-like sequence. (**A**) Length and exon–intron organization of *slc26a2* in a notothenioid emerald rockcod. Exons and introns are indicated by black boxes and horizontal bars, respectively. (**B**) Schematic representation of the sequence of the newly acquired intron 3 in notothenioid. (**C**) Schematic representation of the sequence of the newly acquired intron 5 in notothenioid. NTE, notothenioid putative transposable element. (**D**–**K**) Insertions of notothenioid putative transposable elements NTE-1 s to multiple loci of the notothenioid genomes. Insertion of NTE-1 to *slc26a2* (**D**) and other loci (**E**–**K**) is shown. Accession numbers and the regions of indicated sequences are listed at the beginning of each line. Double slash indicates shortening sequence. Gaps are indicated by dashes in the sequences. Putative direct and inverted repeats are shown in red and blue, respectively. Protein coding and noncoding sequences are indicated by upper- and lower-case letters, respectively. gt-ag of intron 3 in *slc26a2* are shown by gray boxes. *Tbe Trematomus bernacchii*, *Nro Notothenia rossii*, *Cac Chaenocephalus aceratus*, *Han Harpagifer antarcticus*, *Pal Pogonophryne albipinna*, *Ema Eleginops maclovinus*, *Bdi Bovichtus diacanthus*.

A BLAST analysis of the whole genome sequences of Nototenia species using intron sequences as queries revealed that sequences similar to parts of the intron were interspersed throughout the genome, and suggest that these introns consisted of putative transposon sequences (Fig. 6). We tentatively refer to these sequences as notothenioid putative transposable elements (NTEs). The 5′ and 3′ end of the new intron 3 of *slc26a2* in the five notothenioids were highly homologous to NTE-1, and the central region of intron 3 was homologous to NTE-2 (Fig. 6B). NTE-2 contained a 37-nt repeat at the center.

The insertion of NTE-1 s into multiple loci of the notothenioid genome is shown in Fig. 6D–K. NTE-1 s had a putative inverted repeat sequence beginning at tgt and ending at aca, flanked by 4 nt putative direct repeat sequences (Fig. 6D–K). Importantly, gt-ag of intron 3 was present in the putative direct and inverted repeats (Fig. 6D). These results suggest a history of intron 3 generation: insertion of NTE-1 into the exon of *slc26a2* formed a new intron, and subsequent insertion of NTE-2 elongated the new intron. The central region of the new intron 5 of *slc26a2* in the five notothenioids were highly homologous to NTE-3, and the 3′ end was homologous to NTE-4 (Fig. 6C). NTE-3 also contained a 37-nt repeat; however, the sequence was not homologous to that of NTE-2.

### The extant teleost slc26a1 and slc26a2 genes are derived from one of the ohnologs resulting from the teleost-specific whole-genome duplication (TGD)

Teleosts often have ohnologs derived from the TGD; however, all teleost species analyzed had one *slc26a1* and *slc26a2* each. No species with two ohnologs of these genes were identified. This indicated that one of the two ohnologs, once acquired from the ancestral teleost species, was deleted during evolution. Simultaneously, *slc26a1* and *slc26a2* in extant teleosts can be derived from one of the two previously acquired ohnologs.

Therefore, to clarify whether *slc26a1* and *slc26a2* of extant teleosts originated from a single ohnolog or were derived from two ohnologs, we performed synteny analysis and analyzed the composition of ohnologs in neighboring genes (Supplementary Fig. S5). The *tnks1* gene was commonly found near *slc26a1* (Supplementary Fig. S5A), and other ohnologs of *tnks1* were found at other loci in teleosts. Molecular phylogenetic analysis *tnks1* classified them as *tnks1a* or *tnks1b* (Supplementary Fig. S5B). All teleost species examined had *slc26a1* in close proximity to *tnks1a* but not to *tnks1b* (Supplementary Fig. S5). In the phylogenetic tree shown in Fig. 4, no branches indicated the presence of the two *slc26a1* ohnologs. These results indicated that all teleost *slc26a1* examined in this study originated from one of the two ohnologs derived from the TGD. In the case of *slc26a2*, we could not find neighboring genes with conserved ohnologs at other loci. However, the phylogenetic tree of teleost Slc26a2 did not show any branches indicating the presence of two *slc26a2* ohnologs. Therefore, it is highly likely that all teleost *slc26a2* examined in this study originated from one of the two ohnologs derived from the TGD.

## Discussion

In this study, we demonstrated the presence of intron turnover of *slc26a1* and *slc26a2* in ray-finned fish and determined the timing of intron turnover**.** In cartilaginous fish, lobe-finned fish, and tetrapods, *slc26a1* and *slc26a2* share a common two-exon structure (Fig. 1), and some conservation of synteny exist between their loci^23^. These results suggest that *slc26a1* and *slc26a2* are ohnologs of the 2R whole-genome duplication occurring in the ancestral species of jawed vertebrates and that the two-exon structure is the primitive structure of *slc26a1* and *slc26a2* in jawed vertebrates^23^. In addition, these analyses indicated that cartilaginous fishes, lobe-finned fish, and tetrapods have conserved two-exon structures of *slc26a1* and *slc26a2*. In contrast to species in these lineages, in ray-finned fish, exon–intron structures are heterogeneous, and intron turnover has been observed at certain times during their evolution. These results are consistent with previous reports that indicate high intron turnover in ray-finned fishes^18,20^, and provide a good sample for understanding intron turnover in ray-finned fishes.

In ray-finned fish, intron turnover occurred in *slc26a1* and *slc26a2* in the common ancestor of Eurypterygii, increasing by five and one intron(s), respectively (Fig. 4). In addition, further intron turnover was observed in *slc26a2*; an ancestral species of some Syngnathiformes lost one intron, and an ancestral species of Notothenioidei gained two introns (Fig. 5). These observations showed that intron turnover occurred at particular times and did not occur broadly or frequently in a variety of species of ray-finned fish. The reasons for this include the following: acquisition of these introns may have been advantageous for survival, or some bottleneck or founder effect may have influenced the timing of intron turnover. Currently, the benefits arising from the acquisition of these introns are unclear. Intron gain can lead to diversity in alternative splicing. Several reports of alternative splicing exist in other members with approximately 20 exons, such as *slc26a6* and *slc26a7*^66,67^. Future analysis of Eurypterygii species transcripts is expected to reveal whether the intron gain plays a role in generating new splicing isoforms. Synteny analyses suggested that extant *slc26a1* and *slc26a2* are orthologs of one of two ohnologs generated by the teleost-specific whole-genome duplication, and no paralogs for *slc26a1* and *slc26a2* have been found in ray-finned fishes. Therefore, the intron turnover observed in this study occurred in the orthologs of *slc26a1* and *slc26a2* in ray-finned fish.

The intron 3 and 5 of the five Notothenia species were recently acquired from their common ancestor. These introns consisted of putative transposable elements, indicating that they were generated by transposon insertion. Genome analyses of Notothenia species indicated a two-fold change in genome size due to the expansion of the transposable element family^63,68^. Therefore, the intron gain in Notothenia *slc26a2* is likely to be explained as part of a genome-wide change. Transposon insertion is one of the major mechanisms of intron acquisition^2,16^. Expansion of the transposable element family in Notothenia species is expected to be an excellent target for understanding the mechanism of intron acquisition via transposon insertion.

Some of the newly acquired introns in *slc26a1* and *slc26a2* of Eurypterygii were inserted at the same positions as the introns of other *slc26* family members, such as *slc26a3* or jawless fish *slc26a2*. A comparison of the intron insertion sites is presented in Fig. 2. Several new introns were inserted at the same or very close to the introns of the other *slc26* members, such as *slc26a3*, which does not belong to *slc26a1*/*slc26a2*/*slc26a12* subfamily and consists of 19 exons and 18 introns (Fig. 2). All genes had an intron corresponding to intron 1 of *slc26a1* and *slc26a2* in tetrapods and cartilaginous fishes, suggesting that this intron is very old and was present before the separation of jawed and jawless vertebrates. The insertion sites of intron 1 of Eurypterygii (e.g., Atlantic cod) and Galaxiiformes (peladilla) *slc26a1* matched those of introns 3 and 2 of *slc26a1-like* in hagfish and lamprey, respectively. The insertion sites of intron 5 of Eurypterygii *slc26a1* and intron 3 of Galaxiiformes *slc26a1* matched intron 9 of *slc26a3*. The position of intron 2 of basal ray-finned fish *slc26a2* and its corresponding introns, intron 3 of *slc26a2* in Osmeriformes (e.g., European smelt), intron 3 of *slc26a2* in Eurypterygii other than notothenioid, and intron 4 of *slc26a2* in the five notothenioids (e.g., emerald rockcod), matched the site of intron 10 of *slc26a3*. The positions of intron 2 of *slc26a12-like* in hagfish and intron 5 of *slc26a2-like* in hagfish and lamprey matched those of intron 8 in *slc26a3*. Therefore, the *slc26* family genes convergently acquired introns at the same sites. The mechanism and functional significance of convergent intron acquisition in *slc26* genes are novel questions that should be addressed in future research.

## Conclusion

Intron gain and loss are rare events in vertebrates; however, high levels of intron turnover have been observed in teleosts. *slc26a1* and *slc26a2* are members of the anion exchanger gene family, and their protein-coding regions are encoded by two exons in cartilaginous fish and tetrapods. In the present study, a comparative analysis of the genomes of 62 ray-finned fish species showed that new intron insertions and deletions in these genes occurred at different times in certain fish species. These results provide a useful example for understanding the high levels of intron turnover in teleosts.

## Supplementary Information

Supplementary Information.

## Data Availability

The data underlying this article are available in the International Nucleotide Sequence Database Collaboration (INSDC), consisting of DDBJ, EMBL Bank and GenBank, under the accession numbers indicated in Materials and Methods.
