# Supplementary material for: Intron turnover of slc26a1 and slc26a2 and convergence of intron insertion sites
Source: Sci Rep. 2025 Aug 16;15:30007. doi: 10.1038/s41598-025-15147-w (PMC12357937; doi:10.1038/s41598-025-15147-w)
Supplement: Supplementary file 1 — Supplementary Information. [file 41598_2025_15147_MOESM1_ESM.pdf]

Supplementary Information for

Intron turnover of *slc26a1* and *slc26a2* and convergence of intron insertion sites

Kota Torii[1], Chihiro Ota[1,2], Ayumi Nagashima[1,2], Masaki Kajikawa[1,2], Akira Kato[1,2]*

[1]School of Life Science and Technology, Tokyo Institute of Technology, Yokohama, Japan; [2]School of Life Science and Technology, Institute of Science Tokyo, Yokohama, Japan

Correspondence:

Akira Kato (akirkato@life.isct.ac.jp)
School of Life Science and Technology, Institute of Science Tokyo
4259-B22 Nagatsuta-cho, Midori-ku, Yokohama, 226-8501 JAPAN
Office Phone: +81-45-924-5794

This PDF file includes:

Supplementary Figs. S1–S5

A Elephant shark *slc26a1*

```
Query: sulfate anion transporter 1 [Callorhinchus milii] Query ID: XP_007905889.1 Length: 720


>Callorhinchus milii isolate IMCB2004 unplaced genomic scaffold, IMCB_Cmil_1.0 Scaffold1, whole genome shotgun sequence
Sequence ID: NW_024704742.1 Length: 139202109
Range 1: 31661066 to 31662595

Score:1886 bits(3854), Expect:0.0,
Method:,
Identities:510/510(100%), Positives:510/510(100%), Gaps:0/510(0%)

Query  211       VLMGVFNLGIVSKYLSQPLLDGFATGASITILTTQVKYLVGLKIPRSHGPGTVILTWINI  270
                 VLMGVFNLGIVSKYLSQPLLDGFATGASITILTTQVKYLVGLKIPRSHGPGTVILTWINI
Sbjct  31661066  VLMGVFNLGIVSKYLSQPLLDGFATGASITILTTQVKYLVGLKIPRSHGPGTVILTWINI  31661245

Query  271       FKNIHQTNICDLITTAVCLPVLITVKELGDRYKHKIKFPLPTEMVAVIVATIISHYANLN  330
                 FKNIHQTNICDLITTAVCLPVLITVKELGDRYKHKIKFPLPTEMVAVIVATIISHYANLN
Sbjct  31661246  FKNIHQTNICDLITTAVCLPVLITVKELGDRYKHKIKFPLPTEMVAVIVATIISHYANLN  31661425

Query  331       EKYGSSITGEIPTGFLTPQIPNWSLFSRVAIDAVSIAIMSFTFTISICEMFAKKHGYNIK  390
                 EKYGSSITGEIPTGFLTPQIPNWSLFSRVAIDAVSIAIMSFTFTISICEMFAKKHGYNIK
Sbjct  31661426  EKYGSSITGEIPTGFLTPQIPNWSLFSRVAIDAVSIAIMSFTFTISICEMFAKKHGYNIK  31661605

Query  391       ANQEIFALGFCNIVPSFFHSFASSGTLVKTLIKESSGCQTQLSSIITAMIVLLMLLSLAP  450
                 ANQEIFALGFCNIVPSFFHSFASSGTLVKTLIKESSGCQTQLSSIITAMIVLLMLLSLAP
Sbjct  31661606  ANQEIFALGFCNIVPSFFHSFASSGTLVKTLIKESSGCQTQLSSIITAMIVLLMLLSLAP  31661785

Query  451       LLYSLQKSVLACIIIVSLRGAFRKFRDVPKQWQISKIDTLVWGVTMLSVALLTTELGLLI  510
                 LLYSLQKSVLACIIIVSLRGAFRKFRDVPKQWQISKIDTLVWGVTMLSVALLTTELGLLI
Sbjct  31661786  LLYSLQKSVLACIIIVSLRGAFRKFRDVPKQWQISKIDTLVWGVTMLSVALLTTELGLLI  31661965

Query  511       GIVFSLLCIIARTQLPRTPLLGQIQGTSLYKDETEYKNLSFIPRIKIFRFEAPLYYANKD  570
                 GIVFSLLCIIARTQLPRTPLLGQIQGTSLYKDETEYKNLSFIPRIKIFRFEAPLYYANKD
Sbjct  31661966  GIVFSLLCIIARTQLPRTPLLGQIQGTSLYKDETEYKNLSFIPRIKIFRFEAPLYYANKD  31662145

Query  571       FFLNALYKKVDLNPVLVAAKCKKAEKQQNVPKKGRKEKEEFSGNMQENDITNERFVYKSY  630
                 FFLNALYKKVDLNPVLVAAKCKKAEKQQNVPKKGRKEKEEFSGNMQENDITNERFVYKSY
Sbjct  31662146  FFLNALYKKVDLNPVLVAAKCKKAEKQQNVPKKGRKEKEEFSGNMQENDITNERFVYKSY  31662325

Query  631       DFHTIILECSTIQFLDTAGINTMKSVLKDYNEIGIRVLLANCNPSVIDSLSRGEYFGASS  690
                 DFHTIILECSTIQFLDTAGINTMKSVLKDYNEIGIRVLLANCNPSVIDSLSRGEYFGASS
Sbjct  31662326  DFHTIILECSTIQFLDTAGINTMKSVLKDYNEIGIRVLLANCNPSVIDSLSRGEYFGASS  31662505

Query  691       KGIDTLMFYSVHSAVRYAEAAWSVSADSSI  720
                 KGIDTLMFYSVHSAVRYAEAAWSVSADSSI
Sbjct  31662506  KGIDTLMFYSVHSAVRYAEAAWSVSADSSI  31662595



Range 2: 31656844 to 31657476

Score:785 bits(1602), Expect:0.0,
Method:,
Identities:211/211(100%), Positives:211/211(100%), Gaps:0/211(0%)

Query  1         MENQDQLIKMEIHSDSQIYIERTEREKPKVKEIIKKKLKKNCSCSLHKVKNIITGFFPVL  60
                 MENQDQLIKMEIHSDSQIYIERTEREKPKVKEIIKKKLKKNCSCSLHKVKNIITGFFPVL
Sbjct  31656844  MENQDQLIKMEIHSDSQIYIERTEREKPKVKEIIKKKLKKNCSCSLHKVKNIITGFFPVL  31657023

Query  61        QWLPKYNIKKWIVGDAISGLLVAVVLVPQSIAYSLLAGLEPISSLYTSFFANTVYFLMGT  120
                 QWLPKYNIKKWIVGDAISGLLVAVVLVPQSIAYSLLAGLEPISSLYTSFFANTVYFLMGT
Sbjct  31657024  QWLPKYNIKKWIVGDAISGLLVAVVLVPQSIAYSLLAGLEPISSLYTSFFANTVYFLMGT  31657203

Query  121       SRHISVGIFSLMCLMVGQVVDRELQLAGFDVSEDSKKLPRVLASGEKWNASVNSAVRNIT  180
                 SRHISVGIFSLMCLMVGQVVDRELQLAGFDVSEDSKKLPRVLASGEKWNASVNSAVRNIT
Sbjct  31657204  SRHISVGIFSLMCLMVGQVVDRELQLAGFDVSEDSKKLPRVLASGEKWNASVNSAVRNIT  31657383

Query  181       FLGPLNIECDEKCYAASIAVALTFMAGVYQV  211
                 FLGPLNIECDEKCYAASIAVALTFMAGVYQV
Sbjct  31657384  FLGPLNIECDEKCYAASIAVALTFMAGVYQV  31657476
```

B Little skate *slc26a1*

```
Query: sulfate anion transporter 1 [Leucoraja erinacea] Query ID: XP_055502961.1 Length: 720


>Leucoraja erinacea ecotype New England chromosome 1, Leri_hhj_1, whole genome shotgun sequence
Sequence ID: NC_073377.1 Length: 170109691
Range 1: 161478488 to 161480017

Score:1891 bits(3864), Expect:0.0,
Method:,
Identities:509/510(99%), Positives:510/510(100%), Gaps:0/510(0%)

Query  211        YQLLMAIFHLGFVSKYLSEPLLDGFATGASITIVTMQVKYLIGVKLPRSHGPGSVVVTWI  270
                  +QLLMAIFHLGFVSKYLSEPLLDGFATGASITIVTMQVKYLIGVKLPRSHGPGSVVVTWI
```

Sbjct 161478488 FQLLMAIFHLGFVSKYLSEPLLDGFATGASITIVTMQVKYLIGVKLPRSHGPGSVVVTWI 161478667

Query 271   NIFKNIHKTNLCDLITTAICMPLLVVSKELGERYKDKCKFPFPMELTIVVIATIISHFVN 330
                  NIFKNIHKTNLCDLITTAICMPLLVVSKELGERYKDKCKFPFPMELTIVVIATIISHFVN
Sbjct 161478668 NIFKNIHKTNLCDLITTAICMPLLVVSKELGERYKDKCKFPFPMELTIVVIATIISHFVN 161478847

Query 331   LNEIYGSSITGPIPTGFLPPTVPSLTILPRVAIDAITLAIISFAFTISLSELFAKKHGYT 390
                  LNEIYGSSITGPIPTGFLPPTVPSLTILPRVAIDAITLAIISFAFTISLSELFAKKHGYT
Sbjct 161478848 LNEIYGSSITGPIPTGFLPPTVPSLTILPRVAIDAITLAIISFAFTISLSELFAKKHGYT 161479027

Query 391   IQANQETYALGFCNVIQSFFHSYASSATLVKTLVKDSSGCQTQLSSLVSAVLVLMLLLFL 450
                  IQANQETYALGFCNVIQSFFHSYASSATLVKTLVKDSSGCQTQLSSLVSAVLVLMLLLFL
Sbjct 161479028 IQANQETYALGFCNVIQSFFHSYASSATLVKTLVKDSSGCQTQLSSLVSAVLVLMLLLFL 161479207

Query 451   APVFYSLQKCVLASIIIVSLRGALMKFRDLPQLWRMSKIDTMVWWVTMLSVCLISTELGL 510
                  APVFYSLQKCVLASIIIVSLRGALMKFRDLPQLWRMSKIDTMVWWVTMLSVCLISTELGL
Sbjct 161479208 APVFYSLQKCVLASIIIVSLRGALMKFRDLPQLWRMSKIDTMVWWVTMLSVCLISTELGL 161479387

Query 511   FTGVTFSLLCVIVRTQVPRTALLGRKQDSTLYEDQAEYNNLSSIPQIKIFRFEASLYYCN 570
                  FTGVTFSLLCVIVRTQVPRTALLGRKQDSTLYEDQAEYNNLSSIPQIKIFRFEASLYYCN
Sbjct 161479388 FTGVTFSLLCVIVRTQVPRTALLGRKQDSTLYEDQAEYNNLSSIPQIKIFRFEASLYYCN 161479567

Query 571   KDYFMESLMRKVGLNPALEIAKQKKAENKRKGKKKSGETFNGNINQEQPVVIKELIPKIY 630
                  KDYFMESLMRKVGLNPALEIAKQKKAENKRKGKKKSGETFNGNINQEQPVVIKELIPKIY
Sbjct 161479568 KDYFMESLMRKVGLNPALEIAKQKKAENKRKGKKKSGETFNGNINQEQPVVIKELIPKIY 161479747

Query 631   NFHTIILDCSTIQFLDSVGINTMKTVLKDYNDIGITVLLANCNPSVLDSLSRGQYFGENC 690
                  NFHTIILDCSTIQFLDSVGINTMKTVLKDYNDIGITVLLANCNPSVLDSLSRGQYFGENC
Sbjct 161479748 NFHTIILDCSTIQFLDSVGINTMKTVLKDYNDIGITVLLANCNPSVLDSLSRGQYFGENC 161479927

Query 691   KEMDNLLFYSVHAAVQYAEVKWSESGNSSL 720
                  KEMDNLLFYSVHAAVQYAEVKWSESGNSSL
Sbjct 161479928 KEMDNLLFYSVHAAVQYAEVKWSESGNSSL 161480017

Range 2: 161472086 to 161472721

Score:800 bits(1633), Expect:0.0,
Method:,
Identities:212/212(100%), Positives:212/212(100%), Gaps:0/212(0%)

Query 1   MENEEQMTKMELHLESQIRIERTVKEEVDTKKTIEKKLRENCSCSLDKLKNLIIGFFPVL 60
                  MENEEQMTKMELHLESQIRIERTVKEEVDTKKTIEKKLRENCSCSLDKLKNLIIGFFPVL
Sbjct 161472086 MENEEQMTKMELHLESQIRIERTVKEEVDTKKTIEKKLRENCSCSLDKLKNLIIGFFPVL 161472265

Query 61   HWLPRYKFKEWILGDTISGLLVAVVIVPQAIAYALLAGLETSSSLYASFFFSCIIYFLMG 120
                  HWLPRYKFKEWILGDTISGLLVAVVIVPQAIAYALLAGLETSSSLYASFFFSCIIYFLMG
Sbjct 161472266 HWLPRYKFKEWILGDTISGLLVAVVIVPQAIAYALLAGLETSSSLYASFFFSCIIYFLMG 161472445

Query 121   TSRHISVGIFSLICLLVGQVVDRELLLAGYEVEDFTVLPSAMNNANNWTSSNVTTSMIMN 180
                  TSRHISVGIFSLICLLVGQVVDRELLLAGYEVEDFTVLPSAMNNANNWTSSNVTTSMIMN
Sbjct 161472446 TSRHISVGIFSLICLLVGQVVDRELLLAGYEVEDFTVLPSAMNNANNWTSSNVTTSMIMN 161472625

Query 181   ITFPGSLNMECDRKCYAISVSAALTFMCGVYQ 212
                  ITFPGSLNMECDRKCYAISVSAALTFMCGVYQ
Sbjct 161472626 ITFPGSLNMECDRKCYAISVSAALTFMCGVYQ 161472721

C Smaller spotted catshark *slc26a1*

Query: sulfate anion transporter 1 [Scyliorhinus canicula] Query ID: XP_038648256.1 Length: 719

>Scyliorhinus canicula chromosome 3, sScyCan1.1, whole genome shotgun sequence
Sequence ID: NC_052148.1 Length: 277254249
Range 1: 226975002 to 226976528

Score:1886 bits(3853), Expect:0.0,
Method:,
Identities:508/509(99%), Positives:509/509(100%), Gaps:0/509(0%)

Query 211   YQVLMAVFNLGFVSKYLSEPLLDGFATGASITIVTMQVKYLIGIKLPRSHGPGSVVVTWI 270
                  +QVLMAVFNLGFVSKYLSEPLLDGFATGASITIVTMQVKYLIGIKLPRSHGPGSVVVTWI
Sbjct 226976528 FQVLMAVFNLGFVSKYLSEPLLDGFATGASITIVTMQVKYLIGIKLPRSHGPGSVVVTWI 226976349

Query 271   NIFKNIHKTNFCDLITTAICMPLLIVSKELGERYKHKLKFPFPMELTIVVIATIISHFVN 330
                  NIFKNIHKTNFCDLITTAICMPLLIVSKELGERYKHKLKFPFPMELTIVVIATIISHFVN
Sbjct 226976348 NIFKNIHKTNFCDLITTAICMPLLIVSKELGERYKHKLKFPFPMELTIVVIATIISHFVN 226976169

Query 331   LNEIYGSSITGPIPTGFLPPKVPNWHLLPRVAMDAVTLAIISFAFTISLSELFAKKHGYT 390
                  LNEIYGSSITGPIPTGFLPPKVPNWHLLPRVAMDAVTLAIISFAFTISLSELFAKKHGYT
Sbjct 226976168 LNEIYGSSITGPIPTGFLPPKVPNWHLLPRVAMDAVTLAIISFAFTISLSELFAKKHGYT 226975989

Query 391   IQANQETYALGFCNVVQSFFHSYASSATLVKTLVKDSNGCQTQLSSVVSAVLVLMLLLFL 450
                  IQANQETYALGFCNVVQSFFHSYASSATLVKTLVKDSNGCQTQLSSVVSAVLVLMLLLFL
Sbjct 226975988 IQANQETYALGFCNVVQSFFHSYASSATLVKTLVKDSNGCQTQLSSVVSAVLVLMLLLFL 226975809

Query 451   APVFYSLQKCVLASIIIVSLRGALMKFRDLPKQWRMSKIDTLVWWVTMLSVTLISTELGL 510
                  APVFYSLQKCVLASIIIVSLRGALMKFRDLPKQWRMSKIDTLVWWVTMLSVTLISTELGL
Sbjct 226975808 APVFYSLQKCVLASIIIVSLRGALMKFRDLPKQWRMSKIDTLVWWVTMLSVTLISTELGL 226975629

```
Query  511        FTGVTFSLLCVIIRTQVPRTALLGQIQGSNLYEDQAEYNNLTSIPQIKIFRFEAPLYYAN  570
                  FTGVTFSLLCVIIRTQVPRTALLGQIQGSNLYEDQAEYNNLTSIPQIKIFRFEAPLYYAN
Sbjct  226975628  FTGVTFSLLCVIIRTQVPRTALLGQIQGSNLYEDQAEYNNLTSIPQIKIFRFEAPLYYAN  226975449

Query  571        KDFFLSALMRKVDLNPALEVAKQKKEESKQIKKKEEKAFNGNINKEQPVISKKLIPKVYS  630
                  KDFFLSALMRKVDLNPALEVAKQKKEESKQIKKKEEKAFNGNINKEQPVISKKLIPKVYS
Sbjct  226975448  KDFFLSALMRKVDLNPALEVAKQKKEESKQIKKKEEKAFNGNINKEQPVISKKLIPKVYS  226975269

Query  631        FHTIILDCSTVQFLDTVGINTLKTVLKDYNEIGIRVLLANCNPSVIDSLSRGGYFGENCK  690
                  FHTIILDCSTVQFLDTVGINTLKTVLKDYNEIGIRVLLANCNPSVIDSLSRGGYFGENCK
Sbjct  226975268  FHTIILDCSTVQFLDTVGINTLKTVLKDYNEIGIRVLLANCNPSVIDSLSRGGYFGENCK  226975089

Query  691        EMDTLLFYSVHVAVQYAKANWPEPGNSSL  719
                  EMDTLLFYSVHVAVQYAKANWPEPGNSSL
Sbjct  226975088  EMDTLLFYSVHVAVQYAKANWPEPGNSSL  226975002
```

```
Range 2: 226981067 to 226981705

Score:801 bits(1635), Expect:0.0,
Method:,
Identities:213/213(100%), Positives:213/213(100%), Gaps:0/213(0%)

Query  1          MENEEQLSKMELHSESHIQIERTVKEKVAVKKVIERKLQENCSCSLDKFKTLITGFFPVL  60
                  MENEEQLSKMELHSESHIQIERTVKEKVAVKKVIERKLQENCSCSLDKFKTLITGFFPVL
Sbjct  226981705  MENEEQLSKMELHSESHIQIERTVKEKVAVKKVIERKLQENCSCSLDKFKTLITGFFPVL  226981526

Query  61         EWLPKYKLKEWILGDTISGLLVAVVMVPQSIAYALLAGLETSSSLYASFFSCIIYFMMGT  120
                  EWLPKYKLKEWILGDTISGLLVAVVMVPQSIAYALLAGLETSSSLYASFFSCIIYFMMGT
Sbjct  226981525  EWLPKYKLKEWILGDTISGLLVAVVMVPQSIAYALLAGLETSSSLYASFFSCIIYFMMGT  226981346

Query  121        SRHISVGIFSLICLLVGQVVDRELLLAGFDVEEESKVMSTAMGSVDNWNISDVNPTMIMN  180
                  SRHISVGIFSLICLLVGQVVDRELLLAGFDVEEESKVMSTAMGSVDNWNISDVNPTMIMN
Sbjct  226981345  SRHISVGIFSLICLLVGQVVDRELLLAGFDVEEESKVMSTAMGSVDNWNISDVNPTMIMN  226981166

Query  181        FTLPGSQNFECDRKCYAVSVSAALTFMCGIYQV  213
                  FTLPGSQNFECDRKCYAVSVSAALTFMCGIYQV
Sbjct  226981165  FTLPGSQNFECDRKCYAVSVSAALTFMCGIYQV  226981067
```

D Human *slc26a1*

```
Query: sulfate anion transporter 1 isoform a [Homo sapiens] Query ID: NP_071325.2 Length: 701


>Homo sapiens chromosome 4, GRCh38.p14 Primary Assembly
Sequence ID: NC_000004.12 Length: 190214555
Range 1: 988836 to 990365

Score:1832 bits(3743), Expect:0.0,
Method:,
Identities:510/510(100%), Positives:510/510(100%), Gaps:0/510(0%)

Query  192     QVLMGVLRLGFVSAYLSQPLLDGFAMGASVTILTSQLKHLLGVRIPRHQGPGMVVLTWLS  251
               QVLMGVLRLGFVSAYLSQPLLDGFAMGASVTILTSQLKHLLGVRIPRHQGPGMVVLTWLS
Sbjct  990365  QVLMGVLRLGFVSAYLSQPLLDGFAMGASVTILTSQLKHLLGVRIPRHQGPGMVVLTWLS  990186

Query  252     LLRGAGQANVCDVVTSTVCLAVLLAAKELSDRYRHRLRVPLPTELLVIVVATLVSHFGQL  311
               LLRGAGQANVCDVVTSTVCLAVLLAAKELSDRYRHRLRVPLPTELLVIVVATLVSHFGQL
Sbjct  990185  LLRGAGQANVCDVVTSTVCLAVLLAAKELSDRYRHRLRVPLPTELLVIVVATLVSHFGQL  990006

Query  312     HKRFGSSVAGDIPTGFMPPQVPEPRLMQRVALDAVALALVAAAFSISLAEMFARSHGYSV  371
               HKRFGSSVAGDIPTGFMPPQVPEPRLMQRVALDAVALALVAAAFSISLAEMFARSHGYSV
Sbjct  990005  HKRFGSSVAGDIPTGFMPPQVPEPRLMQRVALDAVALALVAAAFSISLAEMFARSHGYSV  989826

Query  372     RANQELLAVGCCNVLPAFLHCFATSAALAKSLVKTATGCRTQLSSVVSATVVLLVLLALA  431
               RANQELLAVGCCNVLPAFLHCFATSAALAKSLVKTATGCRTQLSSVVSATVVLLVLLALA
Sbjct  989825  RANQELLAVGCCNVLPAFLHCFATSAALAKSLVKTATGCRTQLSSVVSATVVLLVLLALA  989646

Query  432     PLFHDLQRSVLACVIVVSLRGALRKVWDLPRLWRMSPADALVWAGTAATCMLVSTEAGLL  491
               PLFHDLQRSVLACVIVVSLRGALRKVWDLPRLWRMSPADALVWAGTAATCMLVSTEAGLL
Sbjct  989645  PLFHDLQRSVLACVIVVSLRGALRKVWDLPRLWRMSPADALVWAGTAATCMLVSTEAGLL  989466

Query  492     AGVILSLLSLAGRTQRPRTALLARIGDTAFYEDATEFEGLVPEPGVRVFRFGGPLYYANK  551
               AGVILSLLSLAGRTQRPRTALLARIGDTAFYEDATEFEGLVPEPGVRVFRFGGPLYYANK
Sbjct  989465  AGVILSLLSLAGRTQRPRTALLARIGDTAFYEDATEFEGLVPEPGVRVFRFGGPLYYANK  989286

Query  552     DFFLQSLYSLTGLDAGCMAARRKEGGSETGVGEGGPAQGEDLGPVSTRAALVPAAAGFHT  611
               DFFLQSLYSLTGLDAGCMAARRKEGGSETGVGEGGPAQGEDLGPVSTRAALVPAAAGFHT
Sbjct  989285  DFFLQSLYSLTGLDAGCMAARRKEGGSETGVGEGGPAQGEDLGPVSTRAALVPAAAGFHT  989106

Query  612     VVIDCAPLLFLDAAGVSTLQDLRRDYGALGISLLLACCSPPVRDILSRGGFLGEGPGDTA  671
               VVIDCAPLLFLDAAGVSTLQDLRRDYGALGISLLLACCSPPVRDILSRGGFLGEGPGDTA
Sbjct  989105  VVIDCAPLLFLDAAGVSTLQDLRRDYGALGISLLLACCSPPVRDILSRGGFLGEGPGDTA  988926

Query  672     EEEQLFLSVHDAVQTARARHRELEATDAHL  701
               EEEQLFLSVHDAVQTARARHRELEATDAHL
Sbjct  988925  EEEQLFLSVHDAVQTARARHRELEATDAHL  988836
```

Range 2: 991125 to 991703

Score:718 bits(1465), Expect:0.0,
Method:,
Identities:193/193(100%), Positives:193/193(100%), Gaps:0/193(0%)

```
Query  1       MDESPEPLQQGRGPVPVRRQRPAPRGLREMLKARLWCSCSCSVLCVRALVQDLLPATRWL  60
               MDESPEPLQQGRGPVPVRRQRPAPRGLREMLKARLWCSCSCSVLCVRALVQDLLPATRWL
Sbjct  991703  MDESPEPLQQGRGPVPVRRQRPAPRGLREMLKARLWCSCSCSVLCVRALVQDLLPATRWL  991524

Query  61      RQYRPREYLAGDVMSGLVIGIILVPQAIAYSLLAGLQPIYSLYTSFFANLIYFLMGTSRH  120
               RQYRPREYLAGDVMSGLVIGIILVPQAIAYSLLAGLQPIYSLYTSFFANLIYFLMGTSRH
Sbjct  991523  RQYRPREYLAGDVMSGLVIGIILVPQAIAYSLLAGLQPIYSLYTSFFANLIYFLMGTSRH  991344

Query  121     VSVGIFSLLCLMVGQVVDRELQLAGFDPSQDGLQPGANSSTLNGSAAMLDCGRDCYAIRV  180
               VSVGIFSLLCLMVGQVVDRELQLAGFDPSQDGLQPGANSSTLNGSAAMLDCGRDCYAIRV
Sbjct  991343  VSVGIFSLLCLMVGQVVDRELQLAGFDPSQDGLQPGANSSTLNGSAAMLDCGRDCYAIRV  991164

Query  181     ATALTLMTGLYQV  193
               ATALTLMTGLYQV
Sbjct  991163  ATALTLMTGLYQV  991125
```

E Dog *slc26a1*

Query: sulfate anion transporter 1 [Canis lupus familiaris] Query ID: XP_038518066.1 Length: 706

>Canis lupus familiaris isolate SID07034 breed Labrador retriever chromosome 3, ROS_Cfam_1.0, whole genome shotgun sequence
Sequence ID: NC_051807.1 Length: 92870237
Range 1: 92529654 to 92531183

Score:1826 bits(3730), Expect:0.0,
Method:,
Identities:510/510(100%), Positives:510/510(100%), Gaps:0/510(0%)

```
Query  197       QVLMGILRLGFVSAYLSQPLLDGFAMGASVTILTSQLRHLLGVQIPRHQGLGMVVSTWLS  256
                 QVLMGILRLGFVSAYLSQPLLDGFAMGASVTILTSQLRHLLGVQIPRHQGLGMVVSTWLS
Sbjct  92529654  QVLMGILRLGFVSAYLSQPLLDGFAMGASVTILTSQLRHLLGVQIPRHQGLGMVVSTWLS  92529833

Query  257       LLRSIGQANLCDVLTSATCLVVLLAAKELADRCRHRLKVPLPTELLVIVTATLVSHYGQF  316
                 LLRSIGQANLCDVLTSATCLVVLLAAKELADRCRHRLKVPLPTELLVIVTATLVSHYGQF
Sbjct  92529834  LLRSIGQANLCDVLTSATCLVVLLAAKELADRCRHRLKVPLPTELLVIVTATLVSHYGQF  92530013

Query  317       HERFGSSVAGDIPTGFVAPRVPDPGLMWRVVLDAVPLALVASAFSISLAEMFARSHGYSV  376
                 HERFGSSVAGDIPTGFVAPRVPDPGLMWRVVLDAVPLALVASAFSISLAEMFARSHGYSV
Sbjct  92530014  HERFGSSVAGDIPTGFVAPRVPDPGLMWRVVLDAVPLALVASAFSISLAEMFARSHGYSV  92530193

Query  377       RANQELLAVGCCNVLPAFFHCYVTSAALSKTLVKTATGCRTQLSSVVSAAVVLLVLLALA  436
                 RANQELLAVGCCNVLPAFFHCYVTSAALSKTLVKTATGCRTQLSSVVSAAVVLLVLLALA
Sbjct  92530194  RANQELLAVGCCNVLPAFFHCYVTSAALSKTLVKTATGCRTQLSSVVSAAVVLLVLLALA  92530373

Query  437       PLFRDLQRCVLACVIVVSLRGALRKVRDVPQLWRLSPADALVWVATAATCVLLSVEAGLL  496
                 PLFRDLQRCVLACVIVVSLRGALRKVRDVPQLWRLSPADALVWVATAATCVLLSVEAGLL
Sbjct  92530374  PLFRDLQRCVLACVIVVSLRGALRKVRDVPQLWRLSPADALVWVATAATCVLLSVEAGLL  92530553

Query  497       AGLVLSLLSLAGRTQRPRAVLLAQIGDTGFYEDAAEFEGLVPEPGVRVFRFAGPLYYANK  556
                 AGLVLSLLSLAGRTQRPRAVLLAQIGDTGFYEDAAEFEGLVPEPGVRVFRFAGPLYYANK
Sbjct  92530554  AGLVLSLLSLAGRTQRPRAVLLAQIGDTGFYEDAAEFEGLVPEPGVRVFRFAGPLYYANK  92530733

Query  557       DFFLRSLYGLTGLDAGQAAARRKERGPGAGAGEGDAVGGVDLGPAGSSAALMPTEGGFHA  616
                 DFFLRSLYGLTGLDAGQAAARRKERGPGAGAGEGDAVGGVDLGPAGSSAALMPTEGGFHA
Sbjct  92530734  DFFLRSLYGLTGLDAGQAAARRKERGPGAGAGEGDAVGGVDLGPAGSSAALMPTEGGFHA  92530913

Query  617       VVIDCAPLLFLDAAGVATLRDLRRDYGALDITLLLACCSPLVRNTLRRGGFLGDDPGDAA  676
                 VVIDCAPLLFLDAAGVATLRDLRRDYGALDITLLLACCSPLVRNTLRRGGFLGDDPGDAA
Sbjct  92530914  VVIDCAPLLFLDAAGVATLRDLRRDYGALDITLLLACCSPLVRNTLRRGGFLGDDPGDAA  92531093

Query  677       EEAQLFHSVHGAVQVARARRREAAATDSTL  706
                 EEAQLFHSVHGAVQVARARRREAAATDSTL
Sbjct  92531094  EEAQLFHSVHGAVQVARARRREAAATDSTL  92531183
```

Range 2: 92528503 to 92529096

Score:725 bits(1479), Expect:0.0,
Method:,
Identities:198/198(100%), Positives:198/198(100%), Gaps:0/198(0%)

```
Query  1         MEVSPECPRRGGGPVLVRRRSPVPLGLRETLKARLWRSCTCSTRGAWAWVQDLLPATRWL  60
                 MEVSPECPRRGGGPVLVRRRSPVPLGLRETLKARLWRSCTCSTRGAWAWVQDLLPATRWL
Sbjct  92528503  MEVSPECPRRGGGPVLVRRRSPVPLGLRETLKARLWRSCTCSTRGAWAWVQDLLPATRWL  92528682

Query  61        RQYRPREALAGDVMSGLVIGIILVPQAIAYSLLAGLQPIYSLYTSFFANLIYFVMGTSRH  120
                 RQYRPREALAGDVMSGLVIGIILVPQAIAYSLLAGLQPIYSLYTSFFANLIYFVMGTSRH
Sbjct  92528683  RQYRPREALAGDVMSGLVIGIILVPQAIAYSLLAGLQPIYSLYTSFFANLIYFVMGTSRH  92528862
```

```
Query  121       VSVGIFSLLCLMVGQVVDRELLLAGFGPAQDGPGPAANGSAANASASAAGLGPPDCGQDC  180
                 VSVGIFSLLCLMVGQVVDRELLLAGFGPAQDGPGPAANGSAANASASAAGLGPPDCGQDC
Sbjct  92528863  VSVGIFSLLCLMVGQVVDRELLLAGFGPAQDGPGPAANGSAANASASAAGLGPPDCGQDC  92529042

Query  181       YAIRVATALTLVAGIYQV  198
                 YAIRVATALTLVAGIYQV
Sbjct  92529043  YAIRVATALTLVAGIYQV  92529096
```

F Nine-banded armadillo *slc26a1*

Query: sulfate anion transporter 1 [Dasypus novemcinctus] ID: XP_058157867.1(amino acid) Length: 748

Q >Dasypus novemcinctus isolate mDasNov1 chromosome 1, whole genome shotgun sequence
Sequence ID: JAUJUB010000001.1 Length: 210449487
Range 1: 209608125 to 209609654

Score:833 bits(2151), Expect:0.0,
Method:Compositional matrix adjust.,
Identities:506/510(99%), Positives:506/510(99%), Gaps:0/510(0%)

```
Query  239        LMAGIYQVLMGVLRLGFVSAYLSQPLLDGFAAGASLTILTSQLRHLLGVRVPRHRGPGMV  298
                  L A   QVLMGVLRLGFVSAYLSQPLLDGFAAGASLTILTSQLRHLLGVRVPRHRGPGMV
Sbjct  209608125  LTARCPQVLMGVLRLGFVSAYLSQPLLDGFAAGASLTILTSQLRHLLGVRVPRHRGPGMV  209608304

Query  299        VSTWLSLLRSAGQANLCDVLTSAVCLVVLLATKELSERCRHRLRVPLPTELAVIVVATLV  358
                  VSTWLSLLRSAGQANLCDVLTSAVCLVVLLATKELSERCRHRLRVPLPTELAVIVVATLV
Sbjct  209608305  VSTWLSLLRSAGQANLCDVLTSAVCLVVLLATKELSERCRHRLRVPLPTELAVIVVATLV  209608484

Query  359        SHFGRLHERFGSSVAGDIPTGFLAPRAPDPVLMQRvaldavalalvGSTFSVSLAEMFAR  418
                  SHFGRLHERFGSSVAGDIPTGFLAPRAPDPVLMQRVALDAVALALVGSTFSVSLAEMFAR
Sbjct  209608485  SHFGRLHERFGSSVAGDIPTGFLAPRAPDPVLMQRVALDAVALALVGSTFSVSLAEMFAR  209608664

Query  419        SHGYSVRANQELLAVGCCNVVPAFFHCFATSAALAKSLVKTATGCHTQlssvvsaavvll  478
                  SHGYSVRANQELLAVGCCNVVPAFFHCFATSAALAKSLVKTATGCHTQLSSVVSAAVVLL
Sbjct  209608665  SHGYSVRANQELLAVGCCNVVPAFFHCFATSAALAKSLVKTATGCHTQLSSVVSAAVVLL  209608844

Query  479        vllvlaPLFRDLQRCVLACVIVVSLRGALRKVVDVPRLWRLSPADGLVWVATAATCVLLS  538
                  VLLVLAPLFRDLQRCVLACVIVVSLRGALRKVVDVPRLWRLSPADGLVWVATAATCVLLS
Sbjct  209608845  VLLVLAPLFRDLQRCVLACVIVVSLRGALRKVVDVPRLWRLSPADGLVWVATAATCVLLS  209609024

Query  539        TEagllagvllslltlagRTQRPHAALLARVGDSACFEDAAEFEGLVPEPGVHVFRFGGP  598
                  TEAGLLAGVLLSLLTLAGRTQRPHAALLARVGDSACFEDAAEFEGLVPEPGVHVFRFGGP
Sbjct  209609025  TEAGLLAGVLLSLLTLAGRTQRPHAALLARVGDSACFEDAAEFEGLVPEPGVHVFRFGGP  209609204

Query  599        LHYANKDFFLRALYSLTGLDAGHVaaggrepgaraaaSQGPRDPTSTVAALLPGGTGFHA  658
                  LHYANKDFFLRALYSLTGLDAGHVAAGGREPGARAAASQGPRDPTSTVAALLPGGTGFHA
Sbjct  209609205  LHYANKDFFLRALYSLTGLDAGHVAAGGREPGARAAASQGPRDPTSTVAALLPGGTGFHA  209609384

Query  659        VVVDCAPLLFLDAAGLATLRDLRRDYEalgvalllaccgPSVRDMLRRGGFLGEDLGDAA  718
                  VVVDCAPLLFLDAAGLATLRDLRRDYEALGVALLLACCGPSVRDMLRRGGFLGEDLGDAA
Sbjct  209609385  VVVDCAPLLFLDAAGLATLRDLRRDYEALGVALLLACCGPSVRDMLRRGGFLGEDLGDAA  209609564

Query  719        EEGQLFHSVHSAVLAAQARHRALVVADATL  748
                  EEGQLFHSVHSAVLAAQARHRALVVADATL
Sbjct  209609565  EEGQLFHSVHSAVLAAQARHRALVVADATL  209609654
```

Range 2: 209607034 to 209607654

Score:333 bits(854), Expect:2e-95,
Method:Compositional matrix adjust.,
Identities:206/207(99%), Positives:206/207(99%), Gaps:0/207(0%)

```
Query  40         LPGRPEAPGMDAPSELVLQDVGPalvrrralaarGLHEELKGRLRRGCECSWARAWALLQ  99
                  L GRPEAPGMDAPSELVLQDVGPALVRRRALAARGLHEELKGRLRRGCECSWARAWALLQ
Sbjct  209607034  LAGRPEAPGMDAPSELVLQDVGPALVRRRALAARGLHEELKGRLRRGCECSWARAWALLQ  209607213

Query  100        ALVPATHWLPRYRPREALAGDALSGLVIGILLVPQAIAYSLLAGLQPVYSLYTSFFANLI  159
                  ALVPATHWLPRYRPREALAGDALSGLVIGILLVPQAIAYSLLAGLQPVYSLYTSFFANLI
Sbjct  209607214  ALVPATHWLPRYRPREALAGDALSGLVIGILLVPQAIAYSLLAGLQPVYSLYTSFFANLI  209607393

Query  160        YALLGTSRHVSVGIFSLLCLMVGQVVDRELQLAGFDPAQDGPGPaahggarnasaaglal  219
                  YALLGTSRHVSVGIFSLLCLMVGQVVDRELQLAGFDPAQDGPGPAAHGGARNASAAGLAL
Sbjct  209607394  YALLGTSRHVSVGIFSLLCLMVGQVVDRELQLAGFDPAQDGPGPAAHGGARNASAAGLAL  209607573

Query  220        glqgcgRDCYAIRVATALTLMAGIYQV  246
                  GLQGCGRDCYAIRVATALTLMAGIYQV
Sbjct  209607574  GLQGCGRDCYAIRVATALTLMAGIYQV  209607654
```

G African savanna elephant *slc26a1*

Query: sulfate anion transporter 1 [Loxodonta africana] Query ID: XP_010589748.1 Length: 703

>Loxodonta africana isolate ISIS603380 unplaced genomic scaffold, Loxafr3.0 scaffold_18, whole genome shotgun sequence
Sequence ID: NW_003573438.1 Length: 50675436
Range 1: 4824266 to 4825786

Score:1820 bits(3719), Expect:0.0,
Method:,
Identities:507/507(100%), Positives:507/507(100%), Gaps:0/507(0%)

```
Query  197      QVLMGVLRLGFMSTYLSPPLLDGFAMGASVTILTSQLKHLLGVRVPRHQGPGMVLSTWLS  256
                QVLMGVLRLGFMSTYLSPPLLDGFAMGASVTILTSQLKHLLGVRVPRHQGPGMVLSTWLS
Sbjct  4824266  QVLMGVLRLGFMSTYLSPPLLDGFAMGASVTILTSQLKHLLGVRVPRHQGPGMVLSTWLS  4824445

Query  257      LLRSASQANVCDVVTSAVCLAVLLAAKELSDRYRHHLKVPLPMELVVIVLATLASHFGQF  316
                LLRSASQANVCDVVTSAVCLAVLLAAKELSDRYRHHLKVPLPMELVVIVLATLASHFGQF
Sbjct  4824446  LLRSASQANVCDVVTSAVCLAVLLAAKELSDRYRHHLKVPLPMELVVIVLATLASHFGQF  4824625

Query  317      HERFGSSVAGHIPTGFLAPQVPDPGLMWRVALDAVPLALVGSAFSISLAEMFARNHGYSV  376
                HERFGSSVAGHIPTGFLAPQVPDPGLMWRVALDAVPLALVGSAFSISLAEMFARNHGYSV
Sbjct  4824626  HERFGSSVAGHIPTGFLAPQVPDPGLMWRVALDAVPLALVGSAFSISLAEMFARNHGYSV  4824805

Query  377      RANQELLAVGFCNVVPAFFHCFATSAALAKSLVKTATGCRTQLSSVVSAAVVLLVLLALA  436
                RANQELLAVGFCNVVPAFFHCFATSAALAKSLVKTATGCRTQLSSVVSAAVVLLVLLALA
Sbjct  4824806  RANQELLAVGFCNVVPAFFHCFATSAALAKSLVKTATGCRTQLSSVVSAAVVLLVLLALA  4824985

Query  437      PLFQDLQRCVLACVIVVSLRGALRKVGDLPQLWRLSPADALVWVATAAPCVLVSIEAGLL  496
                PLFQDLQRCVLACVIVVSLRGALRKVGDLPQLWRLSPADALVWVATAAPCVLVSIEAGLL
Sbjct  4824986  PLFQDLQRCVLACVIVVSLRGALRKVGDLPQLWRLSPADALVWVATAAPCVLVSIEAGLL  4825165

Query  497      VGVLLSLLSLVGRTQRPHATLLARISGSAFYEDTMEFEGLLPEPGVQIFRFAGPLYYANK  556
                VGVLLSLLSLVGRTQRPHATLLARISGSAFYEDTMEFEGLLPEPGVQIFRFAGPLYYANK
Sbjct  4825166  VGVLLSLLSLVGRTQRPHATLLARISGSAFYEDTMEFEGLLPEPGVQIFRFAGPLYYANK  4825345

Query  557      DFFLRSLYSLTGLDAGRTAARRRGLDAGAHKGNSSGKDLGPAGTTSVLVSSAASFHTVVI  616
                DFFLRSLYSLTGLDAGRTAARRRGLDAGAHKGNSSGKDLGPAGTTSVLVSSAASFHTVVI
Sbjct  4825346  DFFLRSLYSLTGLDAGRTAARRRGLDAGAHKGNSSGKDLGPAGTTSVLVSSAASFHTVVI  4825525

Query  617      DCAPLLFLDVAGLAALHDLRRNYEALGIVLLLACCSPSVTDMLRRGGFLGEDEGDAAEEE  676
                DCAPLLFLDVAGLAALHDLRRNYEALGIVLLLACCSPSVTDMLRRGGFLGEDEGDAAEEE
Sbjct  4825526  DCAPLLFLDVAGLAALHDLRRNYEALGIVLLLACCSPSVTDMLRRGGFLGEDEGDAAEEE  4825705

Query  677      QLFHSVHSAVQAARARHGELAATDSTL  703
                QLFHSVHSAVQAARARHGELAATDSTL
Sbjct  4825706  QLFHSVHSAVQAARARHGELAATDSTL  4825786
```

Range 2: 4822619 to 4823212

Score:727 bits(1483), Expect:0.0,
Method:,
Identities:198/198(100%), Positives:198/198(100%), Gaps:0/198(0%)

```
Query  1        MEASPECTQQDGGLGLVRRRPPAPQSLRETLKGRLQQSCTCRGRKAWALVQDLVPATHWL  60
                MEASPECTQQDGGLGLVRRRPPAPQSLRETLKGRLQQSCTCRGRKAWALVQDLVPATHWL
Sbjct  4822619  MEASPECTQQDGGLGLVRRRPPAPQSLRETLKGRLQQSCTCRGRKAWALVQDLVPATHWL  4822798

Query  61       PRYRLQEDLAGDVMSGLVIGIILVPQAIAYSLLAGLQPLYSLYTSFFANLIYFLLGTSRH  120
                PRYRLQEDLAGDVMSGLVIGIILVPQAIAYSLLAGLQPLYSLYTSFFANLIYFLLGTSRH
Sbjct  4822799  PRYRLQEDLAGDVMSGLVIGIILVPQAIAYSLLAGLQPLYSLYTSFFANLIYFLLGTSRH  4822978

Query  121      VSVGIFSLLCLMVGQVVDRELQLAGLDPTQDGLWPGANNSTFNASATGLALGPQDCGRDC  180
                VSVGIFSLLCLMVGQVVDRELQLAGLDPTQDGLWPGANNSTFNASATGLALGPQDCGRDC
Sbjct  4822979  VSVGIFSLLCLMVGQVVDRELQLAGLDPTQDGLWPGANNSTFNASATGLALGPQDCGRDC  4823158

Query  181      YAIRVATALTLVAGIYQV  198
                YAIRVATALTLVAGIYQV
Sbjct  4823159  YAIRVATALTLVAGIYQV  4823212
```

H Gray short-tailed opossum *slc26a1*

Query: sulfate anion transporter 1 isoform X1 [Monodelphis domestica] Query ID: XP_007496939.2 Length: 710

>Monodelphis domestica isolate mMonDom1 chromosome 6, mMonDom1.pri, whole genome shotgun sequence
Sequence ID: NC_077232.1 Length: 311077132
Range 1: 75303866 to 75305386

Score:1864 bits(3809), Expect:0.0,
Method:,
Identities:507/507(100%), Positives:507/507(100%), Gaps:0/507(0%)

```
Query  204       VLMGLFQLGFVSTYLSQPLLDGFATGASLTILTSQVKYLFGIKIPRHQGYGMFLATWVNM  263
                 VLMGLFQLGFVSTYLSQPLLDGFATGASLTILTSQVKYLFGIKIPRHQGYGMFLATWVNM
Sbjct  75305386  VLMGLFQLGFVSTYLSQPLLDGFATGASLTILTSQVKYLFGIKIPRHQGYGMFLATWVNM  75305207

Query  264       IRNIGQANVCDVITSAICLTVLVSAKELADRYKKRLKIPLPTELLIIIAATLVSHFGNLH  323
                 IRNIGQANVCDVITSAICLTVLVSAKELADRYKKRLKIPLPTELLIIIAATLVSHFGNLH
Sbjct  75305206  IRNIGQANVCDVITSAICLTVLVSAKELADRYKKRLKIPLPTELLIIIAATLVSHFGNLH  75305027
```

```
Query  324       QRYGSSISGDIPTGFIPPKAPDFGLMHRVALDAVPIAIIGFAFTISLSEMFAKNYGYTIR  383
                 QRYGSSISGDIPTGFIPPKAPDFGLMHRVALDAVPIAIIGFAFTISLSEMFAKNYGYTIR
Sbjct  75305026  QRYGSSISGDIPTGFIPPKAPDFGLMHRVALDAVPIAIIGFAFTISLSEMFAKNYGYTIR  75304847

Query  384       ANQEMFAVGFCNIIPSFFHSFTTSAALAKSLVKTSTGCHTQVSSVVSAAVVLLVLLVLAP  443
                 ANQEMFAVGFCNIIPSFFHSFTTSAALAKSLVKTSTGCHTQVSSVVSAAVVLLVLLVLAP
Sbjct  75304846  ANQEMFAVGFCNIIPSFFHSFTTSAALAKSLVKTSTGCHTQVSSVVSAAVVLLVLLVLAP  75304667

Query  444       LFYSLQKSVLACIIIVSLKGALWKFRDVPKQYRMNRTDALVWCVTMASSALISTEIGLLV  503
                 LFYSLQKSVLACIIIVSLKGALWKFRDVPKQYRMNRTDALVWCVTMASSALISTEIGLLV
Sbjct  75304666  LFYSLQKSVLACIIIVSLKGALWKFRDVPKQYRMNRTDALVWCVTMASSALISTEIGLLV  75304487

Query  504       GVLFSMLCVVGRTQHPRVALLGQVEDTVFYEDSRRYENLLPVPKIKIFRFEAPLYYANKD  563
                 GVLFSMLCVVGRTQHPRVALLGQVEDTVFYEDSRRYENLLPVPKIKIFRFEAPLYYANKD
Sbjct  75304486  GVLFSMLCVVGRTQHPRVALLGQVEDTVFYEDSRRYENLLPVPKIKIFRFEAPLYYANKD  75304307

Query  564       FFLKSLYKMADLDPALEAAKRKRREKEEPALEGRLVEKGAVGELGQVDTTCHLVPRQSEF  623
                 FFLKSLYKMADLDPALEAAKRKRREKEEPALEGRLVEKGAVGELGQVDTTCHLVPRQSEF
Sbjct  75304306  FFLKSLYKMADLDPALEAAKRKRREKEEPALEGRLVEKGAVGELGQVDTTCHLVPRQSEF  75304127

Query  624       HTIIIDCSSVLFLDTAGVSTLKNVRKDYRVVNISVLLAGCNPSVMDSLKRGGYFDNTEAG  683
                 HTIIIDCSSVLFLDTAGVSTLKNVRKDYRVVNISVLLAGCNPSVMDSLKRGGYFDNTEAG
Sbjct  75304126  HTIIIDCSSVLFLDTAGVSTLKNVRKDYRVVNISVLLAGCNPSVMDSLKRGGYFDNTEAG  75303947

Query  684       VKESQFYSVHGAVQFVRAREQVMDSTL  710
                 VKESQFYSVHGAVQFVRAREQVMDSTL
Sbjct  75303946  VKESQFYSVHGAVQFVRAREQVMDSTL  75303866
```

Range 2: 75309814 to 75310425

Score:747 bits(1524), Expect:0.0,
Method:,
Identities:204/204(100%), Positives:204/204(100%), Gaps:0/204(0%)

```
Query  1         MMDGKAELERVDLGLQAPILIQRQAPSQKSLLETAKAKLKKNCSLSPATLKDALLGFFPV  60
                 MMDGKAELERVDLGLQAPILIQRQAPSQKSLLETAKAKLKKNCSLSPATLKDALLGFFPV
Sbjct  75310425  MMDGKAELERVDLGLQAPILIQRQAPSQKSLLETAKAKLKKNCSLSPATLKDALLGFFPV  75310246

Query  61        IGWLPKYRFRDYIVGDIMSGLVIGVILVPQAIAYSLLAGLKPIYSLYTSFFANIIYFLMG  120
                 IGWLPKYRFRDYIVGDIMSGLVIGVILVPQAIAYSLLAGLKPIYSLYTSFFANIIYFLMG
Sbjct  75310245  IGWLPKYRFRDYIVGDIMSGLVIGVILVPQAIAYSLLAGLKPIYSLYTSFFANIIYFLMG  75310066

Query  121       TSRHVSVGIFSLLCLMVGQVVDRELQLAGFDLNEDISLGSRNSSNETFLASDLPLEGIGP  180
                 TSRHVSVGIFSLLCLMVGQVVDRELQLAGFDLNEDISLGSRNSSNETFLASDLPLEGIGP
Sbjct  75310065  TSRHVSVGIFSLLCLMVGQVVDRELQLAGFDLNEDISLGSRNSSNETFLASDLPLEGIGP  75309886

Query  181       ECGKECYAISIATALTFLAGVYQV  204
                 ECGKECYAISIATALTFLAGVYQV
Sbjct  75309885  ECGKECYAISIATALTFLAGVYQV  75309814
```

Platypus *slc26a1*

Query: sulfate anion transporter 1 [Ornithorhynchus anatinus] Query ID: XP_007671012.2 Length: 707

>Ornithorhynchus anatinus isolate Pmale09 chromosome X3, mOrnAna1.pri.v4, whole genome shotgun sequence
Sequence ID: NC_041751.1 Length: 33863336
Range 1: 31027173 to 31028693

Score:1871 bits(3823), Expect:0.0,
Method:,
Identities:507/507(100%), Positives:507/507(100%), Gaps:0/507(0%)

```
Query  201       VLMGLFHLGFISMYLSEPLLDGFATGASLTILTSQVKYLFGIRIPRHHGYGMFLVTWINL  260
                 VLMGLFHLGFISMYLSEPLLDGFATGASLTILTSQVKYLFGIRIPRHHGYGMFLVTWINL
Sbjct  31028693  VLMGLFHLGFISMYLSEPLLDGFATGASLTILTSQVKYLFGIRIPRHHGYGMFLVTWINL  31028514

Query  261       FQNISQANVCDVVTSAICLVVLVAAKELTDRYKQQLKIPLPIELIVIIVATLISHYGNLN  320
                 FQNISQANVCDVVTSAICLVVLVAAKELTDRYKQQLKIPLPIELIVIIVATLISHYGNLN
Sbjct  31028513  FQNISQANVCDVVTSAICLVVLVAAKELTDRYKQQLKIPLPIELIVIIVATLISHYGNLN  31028334

Query  321       ERYASSISGEIPTGFIAPKAPDFQLMRRVALDAVPLAVIGFAFTVSLSEMFAKKFAYTIK  380
                 ERYASSISGEIPTGFIAPKAPDFQLMRRVALDAVPLAVIGFAFTVSLSEMFAKKFAYTIK
Sbjct  31028333  ERYASSISGEIPTGFIAPKAPDFQLMRRVALDAVPLAVIGFAFTVSLSEMFAKKFAYTIK  31028154

Query  381       ANQEMFAIGFCNIIPSFFHSIITSAALAKSLVKTSTGCHTQVSSVVSAVVVLLVLLFFAP  440
                 ANQEMFAIGFCNIIPSFFHSIITSAALAKSLVKTSTGCHTQVSSVVSAVVVLLVLLFFAP
Sbjct  31028153  ANQEMFAIGFCNIIPSFFHSIITSAALAKSLVKTSTGCHTQVSSVVSAVVVLLVLLFFAP  31027974

Query  441       LFYSLQKCVLACIIIVSLRGALRKFKEVAKQYRLSKIDTLVWCVTMASCALISTEIGLLV  500
                 LFYSLQKCVLACIIIVSLRGALRKFKEVAKQYRLSKIDTLVWCVTMASCALISTEIGLLV
Sbjct  31027973  LFYSLQKCVLACIIIVSLRGALRKFKEVAKQYRLSKIDTLVWCVTMASCALISTEIGLLV  31027794

Query  501       GAVFSIMCIVSRTQRPHTALLGQIENTVFYEDDREYENLLPVLKIKVFRFEAPLYYANKD  560
                 GAVFSIMCIVSRTQRPHTALLGQIENTVFYEDDREYENLLPVLKIKVFRFEAPLYYANKD
Sbjct  31027793  GAVFSIMCIVSRTQRPHTALLGQIENTVFYEDDREYENLLPVLKIKVFRFEAPLYYANKD  31027614
```

```
Query  561       FFVRSLYRMTGLDPVLETAKRKKGAKGKRIHPEKGNEEEASWKGLSQIDTTSSLVPNQVD  620
                 FFVRSLYRMTGLDPVLETAKRKKGAKGKRIHPEKGNEEEASWKGLSQIDTTSSLVPNQVD
Sbjct  31027613  FFVRSLYRMTGLDPVLETAKRKKGAKGKRIHPEKGNEEEASWKGLSQIDTTSSLVPNQVD  31027434

Query  621       FQTIIIDCSSVLFLDTAGINTFKEIAKDYKEVNIAVLLACCNPSVIDSLKRGGYFGNAEI  680
                 FQTIIIDCSSVLFLDTAGINTFKEIAKDYKEVNIAVLLACCNPSVIDSLKRGGYFGNAEI
Sbjct  31027433  FQTIIIDCSSVLFLDTAGINTFKEIAKDYKEVNIAVLLACCNPSVIDSLKRGGYFGNAEI  31027254

Query  681       TKELVFYSVHSAIQFVREREGMADSTV  707
                 TKELVFYSVHSAIQFVREREGMADSTV
Sbjct  31027253  TKELVFYSVHSAIQFVREREGMADSTV  31027173
```

Range 2: 31030606 to 31031208

Score:752 bits(1535), Expect:0.0,
Method:,
Identities:201/201(100%), Positives:201/201(100%), Gaps:0/201(0%)

```
Query  1         MEMQSESMAVENHLQFPVLVQRSPPGEGSTRERIKAKLRKNFVCTTGKLKAMILDFFPVV  60
                 MEMQSESMAVENHLQFPVLVQRSPPGEGSTRERIKAKLRKNFVCTTGKLKAMILDFFPVV
Sbjct  31031208  MEMQSESMAVENHLQFPVLVQRSPPGEGSTRERIKAKLRKNFVCTTGKLKAMILDFFPVV  31031029

Query  61        GWLPHYRFKEYIWGDIMSGLIIGIILVPQAIAYSLLAGLKPIYCLYTSFFANIIYFTMGT  120
                 GWLPHYRFKEYIWGDIMSGLIIGIILVPQAIAYSLLAGLKPIYCLYTSFFANIIYFTMGT
Sbjct  31031028  GWLPHYRFKEYIWGDIMSGLIIGIILVPQAIAYSLLAGLKPIYCLYTSFFANIIYFTMGT  31030849

Query  121       SRHVSVGIFSLLSLMVGQVVDRELQLAGFDLNDDMPAGNASGMNLTTVPLTLGGMGVECG  180
                 SRHVSVGIFSLLSLMVGQVVDRELQLAGFDLNDDMPAGNASGMNLTTVPLTLGGMGVECG
Sbjct  31030848  SRHVSVGIFSLLSLMVGQVVDRELQLAGFDLNDDMPAGNASGMNLTTVPLTLGGMGVECG  31030669

Query  181       KECYAISIATALTFLAGAYQV  201
                 KECYAISIATALTFLAGAYQV
Sbjct  31030668  KECYAISIATALTFLAGAYQV  31030606
```

J Chicken *slc26a1*

Query: sulfate anion transporter 1 isoform X3 [Gallus gallus] Query ID: XP_004949396.2 Length: 712

>Gallus gallus isolate bGalGal1 chromosome Z, bGalGal1.mat.broiler.GRCg7b, whole genome shotgun sequence
Sequence ID: NC_052572.1 Length: 86044486
Range 1: 54257085 to 54258605

Score:1870 bits(3820), Expect:0.0,
Method:,
Identities:507/507(100%), Positives:507/507(100%), Gaps:0/507(0%)

```
Query  206       QVLMGVFRLGFVSMYLSESVLDGFATGASLTILTAQVKYLIGIKIPRSQGHGMLVITWIN  265
                 QVLMGVFRLGFVSMYLSESVLDGFATGASLTILTAQVKYLIGIKIPRSQGHGMLVITWIN
Sbjct  54257085  QVLMGVFRLGFVSMYLSESVLDGFATGASLTILTAQVKYLIGIKIPRSQGHGMLVITWIN  54257264

Query  266       IFRNISQANICDIITSSICIVVLVTAKELGDRYKHKLKFPLPTELVVIVVATLVSHYGNL  325
                 IFRNISQANICDIITSSICIVVLVTAKELGDRYKHKLKFPLPTELVVIVVATLVSHYGNL
Sbjct  54257265  IFRNISQANICDIITSSICIVVLVTAKELGDRYKHKLKFPLPTELVVIVVATLVSHYGNL  54257444

Query  326       NEVYSSSVSGAIPTGFIAPKVPRFDLMIRVAIDALPLAVVSFVFTVSLSEMCAKKYAYTI  385
                 NEVYSSSVSGAIPTGFIAPKVPRFDLMIRVAIDALPLAVVSFVFTVSLSEMCAKKYAYTI
Sbjct  54257445  NEVYSSSVSGAIPTGFIAPKVPRFDLMIRVAIDALPLAVVSFVFTVSLSEMCAKKYAYTI  54257624

Query  386       RANQEMFAVGFCNIIPSFFHSFATSAALAKTLVKTSTGCQTQVSGVISAMVVLLVLLFLA  445
                 RANQEMFAVGFCNIIPSFFHSFATSAALAKTLVKTSTGCQTQVSGVISAMVVLLVLLFLA
Sbjct  54257625  RANQEMFAVGFCNIIPSFFHSFATSAALAKTLVKTSTGCQTQVSGVISAMVVLLVLLFLA  54257804

Query  446       PLFYSLQKCVLACIIIVSLRGALRKFRDVPARYHVNKVDTVVWVVTMFASALISTEIGLL  505
                 PLFYSLQKCVLACIIIVSLRGALRKFRDVPARYHVNKVDTVVWVVTMFASALISTEIGLL
Sbjct  54257805  PLFYSLQKCVLACIIIVSLRGALRKFRDVPARYHVNKVDTVVWVVTMFASALISTEIGLL  54257984

Query  506       VGIVFSMLCIIVRTQQPRTALLGQIPDTNFYEDDLEYENLSSVPKVKIFRFEAPLYYANR  565
                 VGIVFSMLCIIVRTQQPRTALLGQIPDTNFYEDDLEYENLSSVPKVKIFRFEAPLYYANR
Sbjct  54257985  VGIVFSMLCIIVRTQQPRTALLGQIPDTNFYEDDLEYENLSSVPKVKIFRFEAPLYYANR  54258164

Query  566       NYFLKSLYRLTDLDPNLEAARRKKYEKKERQQLKEGNQPTVNGLGSRDTTLQLVPKQIDF  625
                 NYFLKSLYRLTDLDPNLEAARRKKYEKKERQQLKEGNQPTVNGLGSRDTTLQLVPKQIDF
Sbjct  54258165  NYFLKSLYRLTDLDPNLEAARRKKYEKKERQQLKEGNQPTVNGLGSRDTTLQLVPKQIDF  54258344

Query  626       QALIVDCSSIPFLDTTGVNTLKEILKDYKELNISVLLACCNPSVIDSLKRGGYFGKDFGS  685
                 QALIVDCSSIPFLDTTGVNTLKEILKDYKELNISVLLACCNPSVIDSLKRGGYFGKDFGS
Sbjct  54258345  QALIVDCSSIPFLDTTGVNTLKEILKDYKELNISVLLACCNPSVIDSLKRGGYFGKDFGS  54258524

Query  686       MQEMLFYSIHNAVRFAKDQKLTADSSV  712
                 MQEMLFYSIHNAVRFAKDQKLTADSSV
Sbjct  54258525  MQEMLFYSIHNAVRFAKDQKLTADSSV  54258605
```

Range 2: 54255127 to 54255747

```
Score:774 bits(1579), Expect:0.0,
Method:,
Identities:207/207(100%), Positives:207/207(100%), Gaps:0/207(0%)

Query  1         MERPLEATKMENSTSCFFMERKAHVKARRKEIVLAKLRKSFSCTPRKLKNFVMDFLPVLR  60
                 MERPLEATKMENSTSCFFMERKAHVKARRKEIVLAKLRKSFSCTPRKLKNFVMDFLPVLR
Sbjct  54255127  MERPLEATKMENSTSCFFMERKAHVKARRKEIVLAKLRKSFSCTPRKLKNFVMDFLPVLR  54255306

Query  61        WLPKYQCKEYIWGDVMSGLVIGIILVPQAIAYSLLAGLKPIYSLYTSFFANIIYFLMGTS  120
                 WLPKYQCKEYIWGDVMSGLVIGIILVPQAIAYSLLAGLKPIYSLYTSFFANIIYFLMGTS
Sbjct  54255307  WLPKYQCKEYIWGDVMSGLVIGIILVPQAIAYSLLAGLKPIYSLYTSFFANIIYFLMGTS  54255486

Query  121       RHVSVGIFSLISLMVGQVVDRELLLAGFDLNDDVTPALGPGSLQTDSQFNTTVFNLTTEG  180
                 RHVSVGIFSLISLMVGQVVDRELLLAGFDLNDDVTPALGPGSLQTDSQFNTTVFNLTTEG
Sbjct  54255487  RHVSVGIFSLISLMVGQVVDRELLLAGFDLNDDVTPALGPGSLQTDSQFNTTVFNLTTEG  54255666

Query  181       MNAECGKECYAIGIATALTFVAGVYQV  207
                 MNAECGKECYAIGIATALTFVAGVYQV
Sbjct  54255667  MNAECGKECYAIGIATALTFVAGVYQV  54255747
```

K American alligator *slc26a1*

```
Query: sulfate anion transporter 1 [Alligator mississippiensis] Query ID: XP_006259319.2 Length: 723


>Alligator mississippiensis isolate rAllMis1 chromosome 3, rAllMis1, whole genome shotgun sequence
Sequence ID: NC_081826.1 Length: 303743212
Range 1: 153379097 to 153380626

Score:1880 bits(3841), Expect:0.0,
Method:,
Identities:509/510(99%), Positives:510/510(100%), Gaps:0/510(0%)

Query  214        YQILMGIFRLGFVSVYLSESMLDGFATGASLTILTAQVKYLIGIKIPRSQGLGMLVMTWF  273
                  +QILMGIFRLGFVSVYLSESMLDGFATGASLTILTAQVKYLIGIKIPRSQGLGMLVMTWF
Sbjct  153380626  FQILMGIFRLGFVSVYLSESMLDGFATGASLTILTAQVKYLIGIKIPRSQGLGMLVMTWF  153380447

Query  274        NIFQNIAQANLCDVITSTICIVVLVAAKEVGDRYKHKLKFPLPTELVIIVVATLVSHYGK  333
                  NIFQNIAQANLCDVITSTICIVVLVAAKEVGDRYKHKLKFPLPTELVIIVVATLVSHYGK
Sbjct  153380446  NIFQNIAQANLCDVITSTICIVVLVAAKEVGDRYKHKLKFPLPTELVIIVVATLVSHYGK  153380267

Query  334        LNEVYGSSVSGAIPTGFIPPQVPSFNLMFRVAVDAVPLAIVSFAFTVSLSEMFAKKYAYT  393
                  LNEVYGSSVSGAIPTGFIPPQVPSFNLMFRVAVDAVPLAIVSFAFTVSLSEMFAKKYAYT
Sbjct  153380266  LNEVYGSSVSGAIPTGFIPPQVPSFNLMFRVAVDAVPLAIVSFAFTVSLSEMFAKKYAYT  153380087

Query  394        IRANQEMFAIAFCNIIPSFFHSFATSAALAKTLVKTSTGCETQVSSVVSAMVVLLVLLFL  453
                  IRANQEMFAIAFCNIIPSFFHSFATSAALAKTLVKTSTGCETQVSSVVSAMVVLLVLLFL
Sbjct  153380086  IRANQEMFAIAFCNIIPSFFHSFATSAALAKTLVKTSTGCETQVSSVVSAMVVLLVLLFL  153379907

Query  454        APLFYSLQKCVLACIIIVSLRGALRKFKDVPQRYYLNKVDTVVWCVTMLSSALISTEVGL  513
                  APLFYSLQKCVLACIIIVSLRGALRKFKDVPQRYYLNKVDTVVWCVTMLSSALISTEVGL
Sbjct  153379906  APLFYSLQKCVLACIIIVSLRGALRKFKDVPQRYYLNKVDTVVWCVTMLSSALISTEVGL  153379727

Query  514        LVGVAFSMLSIIGRTQRPPTALLSQIPSTVFYENDQEYENLSPVPKVKIFRFEAPLYYAN  573
                  LVGVAFSMLSIIGRTQRPPTALLSQIPSTVFYENDQEYENLSPVPKVKIFRFEAPLYYAN
Sbjct  153379726  LVGVAFSMLSIIGRTQRPPTALLSQIPSTVFYENDQEYENLSPVPKVKIFRFEAPLYYAN  153379547

Query  574        KDFFVKSLYRMTGLDPTLEVARRKKQDKREKEHLKKENQATNVNGFSQADTTLHLVPKQL  633
                  KDFFVKSLYRMTGLDPTLEVARRKKQDKREKEHLKKENQATNVNGFSQADTTLHLVPKQL
Sbjct  153379546  KDFFVKSLYRMTGLDPTLEVARRKKQDKREKEHLKKENQATNVNGFSQADTTLHLVPKQL  153379367

Query  634        DFQTIIIDCSSFSFLDIPGVNTLKEILKDYGALNITILLACCTPSVIDSLKRGGYFEKDD  693
                  DFQTIIIDCSSFSFLDIPGVNTLKEILKDYGALNITILLACCTPSVIDSLKRGGYFEKDD
Sbjct  153379366  DFQTIIIDCSSFSFLDIPGVNTLKEILKDYGALNITILLACCTPSVIDSLKRGGYFEKDD  153379187

Query  694        RNMHEMLFYSVHSAVQFARDRKSLADNSTV  723
                  RNMHEMLFYSVHSAVQFARDRKSLADNSTV
Sbjct  153379186  RNMHEMLFYSVHSAVQFARDRKSLADNSTV  153379097



Range 2: 153383488 to 153384135

Score:806 bits(1646), Expect:0.0,
Method:,
Identities:215/216(99%), Positives:216/216(100%), Gaps:0/216(0%)

Query  1          MHEVNAQKMERQIEAIKIDDSSASAFQMERKVHMKINRKEIIQAKLRKSCSCTTQRLKNT  60
                  MHEVNAQKMERQIEAIKIDDSSASAFQMERKVHMKINRKEIIQAKLRKSCSCTTQRLKNT
Sbjct  153384135  MHEVNAQKMERQIEAIKIDDSSASAFQMERKVHMKINRKEIIQAKLRKSCSCTTQRLKNT  153383956

Query  61         VLDFFPVLQWLPKYSYKEYIWGDIMSGLVIGIILVPQAIAYSLLAGLKPIYSLYTSFFAN  120
                  VLDFFPVLQWLPKYSYKEYIWGDIMSGLVIGIILVPQAIAYSLLAGLKPIYSLYTSFFAN
Sbjct  153383955  VLDFFPVLQWLPKYSYKEYIWGDIMSGLVIGIILVPQAIAYSLLAGLKPIYSLYTSFFAN  153383776

Query  121        IIYFLMGTSRHVSVGIFSLISLMVGQVVDRELLLAGFDLNDDVPPAFSDSSPWKRNDSNI  180
                  IIYFLMGTSRHVSVGIFSLISLMVGQVVDRELLLAGFDLNDDVPPAFSDSSPWKRNDSNI
Sbjct  153383775  IIYFLMGTSRHVSVGIFSLISLMVGQVVDRELLLAGFDLNDDVPPAFSDSSPWKRNDSNI  153383596

Query  181        TAFNLTFAGLNAECGKECYAIGIATALTFLAGVYQI  216
```

```
                TAFNLTFAGLNAECGKECYAIGIATALTFLAGVYQ+
Sbjct  153383595  TAFNLTFAGLNAECGKECYAIGIATALTFLAGVYQV  153383488
```

L Painted turtle *slc26a1*

Query: sulfate anion transporter 1 [Chrysemys picta bellii] Query ID: XP_005309029.1 Length: 714

>Chrysemys picta bellii isolate RCT428 unplaced genomic scaffold, Chrysemys_picta_BioNano-3.0.4 Scaffold272, whole genome shotgun sequence
Sequence ID: NW_024885921.1 Length: 559669
Range 1: 390435 to 391967

Score:1883 bits(3848), Expect:0.0,
Method:,
Identities:510/511(99%), Positives:511/511(100%), Gaps:0/511(0%)

```
Query  204     FYQVLMGVFRLGFVSMYLSESVLDGFATGASLTILTAQVKYLIGIKIPRSQGHGILLTTW  263
               F+QVLMGVFRLGFVSMYLSESVLDGFATGASLTILTAQVKYLIGIKIPRSQGHGILLTTW
Sbjct  390435  FFQVLMGVFRLGFVSMYLSESVLDGFATGASLTILTAQVKYLIGIKIPRSQGHGILLTTW  390614

Query  264     INIFRNISQANQCDVITSGVCIAVLVAAKELGDRYKQKLKIPLPTELVVIVVATLVSHYG  323
               INIFRNISQANQCDVITSGVCIAVLVAAKELGDRYKQKLKIPLPTELVVIVVATLVSHYG
Sbjct  390615  INIFRNISQANQCDVITSGVCIAVLVAAKELGDRYKQKLKIPLPTELVVIVVATLVSHYG  390794

Query  324     KLNEVYASSVSGAIPTGFIPPQLPNFNMMHRVALDAVPLAIVGFAFTISLSEMFAKKYAY  383
               KLNEVYASSVSGAIPTGFIPPQLPNFNMMHRVALDAVPLAIVGFAFTISLSEMFAKKYAY
Sbjct  390795  KLNEVYASSVSGAIPTGFIPPQLPNFNMMHRVALDAVPLAIVGFAFTISLSEMFAKKYAY  390974

Query  384     TVRANQEMFAIGFCNIIPSFFHCFATSAALAKTLVKSSTGCQTQVSSVISAVVVLLVLLF  443
               TVRANQEMFAIGFCNIIPSFFHCFATSAALAKTLVKSSTGCQTQVSSVISAVVVLLVLLF
Sbjct  390975  TVRANQEMFAIGFCNIIPSFFHCFATSAALAKTLVKSSTGCQTQVSSVISAVVVLLVLLF  391154

Query  444     LAPLFYSLQKCVLACIIIVSLRGALRKFRDVPQRYHVNKVDTLVWCVTAFSSALISTEMG  503
               LAPLFYSLQKCVLACIIIVSLRGALRKFRDVPQRYHVNKVDTLVWCVTAFSSALISTEMG
Sbjct  391155  LAPLFYSLQKCVLACIIIVSLRGALRKFRDVPQRYHVNKVDTLVWCVTAFSSALISTEMG  391334

Query  504     LLIGVLFSMLCIVVRTQRPRTALLGQIQNTAFYEDDWEYENLCPVPNAKIFRFEAPLYYA  563
               LLIGVLFSMLCIVVRTQRPRTALLGQIQNTAFYEDDWEYENLCPVPNAKIFRFEAPLYYA
Sbjct  391335  LLIGVLFSMLCIVVRTQRPRTALLGQIQNTAFYEDDWEYENLCPVPNAKIFRFEAPLYYA  391514

Query  564     NKDFFLKSLYRMTGLDPTLEAAKRKKNEKKQKEHLKEGNRGTIVKGSDQADTALRLVPKQ  623
               NKDFFLKSLYRMTGLDPTLEAAKRKKNEKKQKEHLKEGNRGTIVKGSDQADTALRLVPKQ
Sbjct  391515  NKDFFLKSLYRMTGLDPTLEAAKRKKNEKKQKEHLKEGNRGTIVKGSDQADTALRLVPKQ  391694

Query  624     VDFQTIIIDCSSVTFLDTAGIITLKEIRKDYNELKITVLLACCNPSVIDSLKRGDFFGKD  683
               VDFQTIIIDCSSVTFLDTAGIITLKEIRKDYNELKITVLLACCNPSVIDSLKRGDFFGKD
Sbjct  391695  VDFQTIIIDCSSVTFLDTAGIITLKEIRKDYNELKITVLLACCNPSVIDSLKRGDFFGKD  391874

Query  684     CKKMHELLFYSIHGAVQFARDRKPLADGSAV  714
               CKKMHELLFYSIHGAVQFARDRKPLADGSAV
Sbjct  391875  CKKMHELLFYSIHGAVQFARDRKPLADGSAV  391967
```

Range 2: 387702 to 388322

Score:777 bits(1585), Expect:0.0,
Method:,
Identities:207/207(100%), Positives:207/207(100%), Gaps:0/207(0%)

```
Query  1       MESQTETIKMDIGPSPSFPMERKVHIQISGRQIIQTKLRKSCSCTTKRLKNVVMDFFPVL  60
               MESQTETIKMDIGPSPSFPMERKVHIQISGRQIIQTKLRKSCSCTTKRLKNVVMDFFPVL
Sbjct  387702  MESQTETIKMDIGPSPSFPMERKVHIQISGRQIIQTKLRKSCSCTTKRLKNVVMDFFPVL  387881

Query  61      RWLPKYHCREHIWGDVMSGVVIGIILVPQAIAYSLLAGLKPIYSLYTSFFANIIYFLMGT  120
               RWLPKYHCREHIWGDVMSGVVIGIILVPQAIAYSLLAGLKPIYSLYTSFFANIIYFLMGT
Sbjct  387882  RWLPKYHCREHIWGDVMSGVVIGIILVPQAIAYSLLAGLKPIYSLYTSFFANIIYFLMGT  388061

Query  121     SRHVSVGIFSLLSLMVGQVVDRELLLAGFDLNDDDDAISNNYEWNGNNSNVTAFNLTLGG  180
               SRHVSVGIFSLLSLMVGQVVDRELLLAGFDLNDDDDAISNNYEWNGNNSNVTAFNLTLGG
Sbjct  388062  SRHVSVGIFSLLSLMVGQVVDRELLLAGFDLNDDDDAISNNYEWNGNNSNVTAFNLTLGG  388241

Query  181     LSAECGKDCYAIGVATALTFLAGFYQV  207
               LSAECGKDCYAIGVATALTFLAGFYQV
Sbjct  388242  LSAECGKDCYAIGVATALTFLAGFYQV  388322
```

M Green anole *slc26a1*

Query: PREDICTED: sulfate anion transporter 1 [Anolis carolinensis] Query ID: XP_008112794.1 Length: 702

>Anolis carolinensis unplaced genomic scaffold, AnoCar2.0 chrUn0009, whole genome shotgun sequence
Sequence ID: NW_003338748.1 Length: 4541181

Range 1: 949784 to 951289

Score:1846 bits(3771), Expect:0.0,
Method:,
Identities:501/502(99%), Positives:502/502(100%), Gaps:0/502(0%)

```
Query  201     YQVLMGIFHLGFVSMYLSEPVLDGFATGASVTILTAQVKYLVGIKIPRAQGYGVLITTWV  260
               +QVLMGIFHLGFVSMYLSEPVLDGFATGASVTILTAQVKYLVGIKIPRAQGYGVLITTWV
Sbjct  949784  FQVLMGIFHLGFVSMYLSEPVLDGFATGASVTILTAQVKYLVGIKIPRAQGYGVLITTWV  949963

Query  261     NIFSNISQANVCDVITSTICITVLVTAKELGDRYKDRLKVPLPTELIVIVMATLVSHYGQ  320
               NIFSNISQANVCDVITSTICITVLVTAKELGDRYKDRLKVPLPTELIVIVMATLVSHYGQ
Sbjct  949964  NIFSNISQANVCDVITSTICITVLVTAKELGDRYKDRLKVPLPTELIVIVMATLVSHYGQ  950143

Query  321     LKEMYGSSVSGEIPTGFIPPQVPSLTLMQRVAVDALPLAIVGFAFTISLSEMFAKKYAYT  380
               LKEMYGSSVSGEIPTGFIPPQVPSLTLMQRVAVDALPLAIVGFAFTISLSEMFAKKYAYT
Sbjct  950144  LKEMYGSSVSGEIPTGFIPPQVPSLTLMQRVAVDALPLAIVGFAFTISLSEMFAKKYAYT  950323

Query  381     VKANQEMFAIGFCNIIPAFFHCFATSAALAKSLVKASTGCQTQVSSLVSAVVVLLVLLFF  440
               VKANQEMFAIGFCNIIPAFFHCFATSAALAKSLVKASTGCQTQVSSLVSAVVVLLVLLFF
Sbjct  950324  VKANQEMFAIGFCNIIPAFFHCFATSAALAKSLVKASTGCQTQVSSLVSAVVVLLVLLFF  950503

Query  441     APLFFSLQKCVLACIIIVSLRGALRKFKDLPQRYRLDRVDALVWCVTMLSSALISTEMGL  500
               APLFFSLQKCVLACIIIVSLRGALRKFKDLPQRYRLDRVDALVWCVTMLSSALISTEMGL
Sbjct  950504  APLFFSLQKCVLACIIIVSLRGALRKFKDLPQRYRLDRVDALVWCVTMLSSALISTEMGL  950683

Query  501     LVGVVFSILCIIGRTQRPHAALLGQIENTVFYEDEEEYTNLLPVPRVKIFRFEAPLYYAN  560
               LVGVVFSILCIIGRTQRPHAALLGQIENTVFYEDEEEYTNLLPVPRVKIFRFEAPLYYAN
Sbjct  950684  LVGVVFSILCIIGRTQRPHAALLGQIENTVFYEDEEEYTNLLPVPRVKIFRFEAPLYYAN  950863

Query  561     KDFFLKCLHNKTGFDPTIEIARRKKAKKKGQPNSHKGLEHGDTGLSLVTKPRDIQAIIID  620
               KDFFLKCLHNKTGFDPTIEIARRKKAKKKGQPNSHKGLEHGDTGLSLVTKPRDIQAIIID
Sbjct  950864  KDFFLKCLHNKTGFDPTIEIARRKKAKKKGQPNSHKGLEHGDTGLSLVTKPRDIQAIIID  951043

Query  621     CSSIPFLDTAGVSALKETFKDHQEWKVTVLLACCSPCVIASLERGGYSASANKNMHELVF  680
               CSSIPFLDTAGVSALKETFKDHQEWKVTVLLACCSPCVIASLERGGYSASANKNMHELVF
Sbjct  951044  CSSIPFLDTAGVSALKETFKDHQEWKVTVLLACCSPCVIASLERGGYSASANKNMHELVF  951223

Query  681     HNIHSAVQFVKERKTTADDSVV  702
               HNIHSAVQFVKERKTTADDSVV
Sbjct  951224  HNIHSAVQFVKERKTTADDSVV  951289
```

Range 2: 947094 to 947705

Score:764 bits(1560), Expect:0.0,
Method:,
Identities:203/204(99%), Positives:204/204(100%), Gaps:0/204(0%)

```
Query  1       MEKEMETNKLENGSHLQFFMEKKTPPKISKTAVIKSQLRKNCSCSRERMKKTVMDFFPVL  60
               MEKEMETNKLENGSHLQFFMEKKTPPKISKTAVIKSQLRKNCSCSRERMKKTVMDFFPVL
Sbjct  947094  MEKEMETNKLENGSHLQFFMEKKTPPKISKTAVIKSQLRKNCSCSRERMKKTVMDFFPVL  947273

Query  61      QWLPKYKCKEYIWGDIMSGLVIGIILVPQAIAYSLLAGLKPIYSLYTSFFANIIYFLMGT  120
               QWLPKYKCKEYIWGDIMSGLVIGIILVPQAIAYSLLAGLKPIYSLYTSFFANIIYFLMGT
Sbjct  947274  QWLPKYKCKEYIWGDIMSGLVIGIILVPQAIAYSLLAGLKPIYSLYTSFFANIIYFLMGT  947453

Query  121     SRHVSVGIFSLLSLMVGQVVDRELLLAGFDLNDDPHEVTGGRNPLNVTVHNLTLGTMSME  180
               SRHVSVGIFSLLSLMVGQVVDRELLLAGFDLNDDPHEVTGGRNPLNVTVHNLTLGTMSME
Sbjct  947454  SRHVSVGIFSLLSLMVGQVVDRELLLAGFDLNDDPHEVTGGRNPLNVTVHNLTLGTMSME  947633

Query  181     CGKECYAIGVATALTFLAGVYQVL  204
               CGKECYAIGVATALTFLAGVYQV+
Sbjct  947634  CGKECYAIGVATALTFLAGVYQVM  947705
```

N Western clawed frog *slc26a1*

Query: sulfate anion transporter 1 [Xenopus tropicalis] Query ID: XP_002935511.2 Length: 724

>Xenopus tropicalis strain Nigerian chromosome 1, UCB_Xtro_10.0, whole genome shotgun sequence
Sequence ID: NC_030677.2 Length: 217471166
Range 1: 84156498 to 84158036

Score:1899 bits(3881), Expect:0.0,
Method:,
Identities:513/513(100%), Positives:513/513(100%), Gaps:0/513(0%)

```
Query  212       QVLMGIFRLGFLSMYLSEPMLDGFATGASLTILTAQVKYLLGLKIPRSPGIGMLVTTWYN  271
                 QVLMGIFRLGFLSMYLSEPMLDGFATGASLTILTAQVKYLLGLKIPRSPGIGMLVTTWYN
Sbjct  84158036  QVLMGIFRLGFLSMYLSEPMLDGFATGASLTILTAQVKYLLGLKIPRSPGIGMLVTTWYN  84157857

Query  272       IFKNIHHANYCDIVTSAICIAVLVAAKEIGDRYKEKIKIPLPTELVVIVVATLVSHYCNL  331
                 IFKNIHHANYCDIVTSAICIAVLVAAKEIGDRYKEKIKIPLPTELVVIVVATLVSHYCNL
Sbjct  84157856  IFKNIHHANYCDIVTSAICIAVLVAAKEIGDRYKEKIKIPLPTELVVIVVATLVSHYCNL  84157677

Query  332       KEIYGSSVSGVIPTGFIPPQVPDFSLFSKIAVDAIPLAVISFAFTISLSEMFAKKYAYTV  391
                 KEIYGSSVSGVIPTGFIPPQVPDFSLFSKIAVDAIPLAVISFAFTISLSEMFAKKYAYTV
Sbjct  84157676  KEIYGSSVSGVIPTGFIPPQVPDFSLFSKIAVDAIPLAVISFAFTISLSEMFAKKYAYTV  84157497
```

```
Query  392       EANQEMFAIGFCNIIPSFFHCFATSAALAKTLVKTSTGCMTQVSSVISAIVVLLVLLFFA  451
                 EANQEMFAIGFCNIIPSFFHCFATSAALAKTLVKTSTGCMTQVSSVISAIVVLLVLLFFA
Sbjct  84157496  EANQEMFAIGFCNIIPSFFHCFATSAALAKTLVKTSTGCMTQVSSVISAIVVLLVLLFFA  84157317

Query  452       PLFYSLQKCVLACIIIVSLRGALRKFKDLPALWRLNKVDAVVWCITLAAAALVSTEVGLM  511
                 PLFYSLQKCVLACIIIVSLRGALRKFKDLPALWRLNKVDAVVWCITLAAAALVSTEVGLM
Sbjct  84157316  PLFYSLQKCVLACIIIVSLRGALRKFKDLPALWRLNKVDAVVWCITLAAAALVSTEVGLM  84157137

Query  512       VGVIFSMLCLILRSQLPYTTMLNQIQDTVFYEDCQKYDNLLPIPTVKIFRFDSPLHYANK  571
                 VGVIFSMLCLILRSQLPYTTMLNQIQDTVFYEDCQKYDNLLPIPTVKIFRFDSPLHYANK
Sbjct  84157136  VGVIFSMLCLILRSQLPYTTMLNQIQDTVFYEDCQKYDNLLPIPTVKIFRFDSPLHYANK  84156957

Query  572       GYFLKSLYKMAKMDPGLVNAQRKKMEKKAKKQGRRKQVDGKQVDSANNIADGETQIELVE  631
                 GYFLKSLYKMAKMDPGLVNAQRKKMEKKAKKQGRRKQVDGKQVDSANNIADGETQIELVE
Sbjct  84156956  GYFLKSLYKMAKMDPGLVNAQRKKMEKKAKKQGRRKQVDGKQVDSANNIADGETQIELVE  84156777

Query  632       KRNDLQTIILDCSCIAFLDITGINVLKGLLKDYKEVQVSVLLACCSTSVIDSLIRGGYFG  691
                 KRNDLQTIILDCSCIAFLDITGINVLKGLLKDYKEVQVSVLLACCSTSVIDSLIRGGYFG
Sbjct  84156776  KRNDLQTIILDCSCIAFLDITGINVLKGLLKDYKEVQVSVLLACCSTSVIDSLIRGGYFG  84156597

Query  692       KENSDIHKLLFYTVHDAVQFARAQSISFTDSTV  724
                 KENSDIHKLLFYTVHDAVQFARAQSISFTDSTV
Sbjct  84156596  KENSDIHKLLFYTVHDAVQFARAQSISFTDSTV  84156498
```

Range 2: 84159938 to 84160576

Score:801 bits(1635), Expect:0.0,
Method:,
Identities:213/213(100%), Positives:213/213(100%), Gaps:0/213(0%)

```
Query  1         MKDSVNMKYESEQSKMEEYSQHAHIHLERKVTNRVSLCKTVKAKVKKQCTCNSKQIKNTF  60
                 MKDSVNMKYESEQSKMEEYSQHAHIHLERKVTNRVSLCKTVKAKVKKQCTCNSKQIKNTF
Sbjct  84160576  MKDSVNMKYESEQSKMEEYSQHAHIHLERKVTNRVSLCKTVKAKVKKQCTCNSKQIKNTF  84160397

Query  61        IGFFPVLRWLPKYDFKENTWGDVMSGLIIGIILVPQAIAYSLLAGLKPIYSLYTSFFANI  120
                 IGFFPVLRWLPKYDFKENTWGDVMSGLIIGIILVPQAIAYSLLAGLKPIYSLYTSFFANI
Sbjct  84160396  IGFFPVLRWLPKYDFKENTWGDVMSGLIIGIILVPQAIAYSLLAGLKPIYSLYTSFFANI  84160217

Query  121       IYFLMGTSRHVSVGIFSLISLMVGQVVDREVQLAGFDLDDDAVPLINNFNMSDMNITRAI  180
                 IYFLMGTSRHVSVGIFSLISLMVGQVVDREVQLAGFDLDDDAVPLINNFNMSDMNITRAI
Sbjct  84160216  IYFLMGTSRHVSVGIFSLISLMVGQVVDREVQLAGFDLDDDAVPLINNFNMSDMNITRAI  84160037

Query  181       NISLGLVDIECGKECYAISIAAILTFTAGVYQV  213
                 NISLGLVDIECGKECYAISIAAILTFTAGVYQV
Sbjct  84160036  NISLGLVDIECGKECYAISIAAILTFTAGVYQV  84159938
```

O Two-lined caecilian *slc26a1*

Query: sulfate anion transporter 1 [Rhinatrema bivittatum] Query ID: XP_029458169.1 Length: 721

>Rhinatrema bivittatum chromosome 1, aRhiBiv1.1, whole genome shotgun sequence
Sequence ID: NC_042615.1 Length: 839681426
Range 1: 410992190 to 410993716

Score:1892 bits(3866), Expect:0.0,
Method:,
Identities:509/509(100%), Positives:509/509(100%), Gaps:0/509(0%)

```
Query  213        VLMGVFHLGFLSMYLSEPMLDGFATGASLMILTAQVKYLLGIKIPRSQGYGMIISTWINI  272
                  VLMGVFHLGFLSMYLSEPMLDGFATGASLMILTAQVKYLLGIKIPRSQGYGMIISTWINI
Sbjct  410992190  VLMGVFHLGFLSMYLSEPMLDGFATGASLMILTAQVKYLLGIKIPRSQGYGMIISTWINI  410992369

Query  273        FKNIYATNFCDVLTSAICIAVLVTAKELGDRYKQKLKVPLPTELIVIVVATLISHFGNLN  332
                  FKNIYATNFCDVLTSAICIAVLVTAKELGDRYKQKLKVPLPTELIVIVVATLISHFGNLN
Sbjct  410992370  FKNIYATNFCDVLTSAICIAVLVTAKELGDRYKQKLKVPLPTELIVIVVATLISHFGNLN  410992549

Query  333        HVYGTSVSGSIPTGFIPPKMPDIWVMPRVAGDAVPLAVVSFAFTISLSEMFAKKYEYTVK  392
                  HVYGTSVSGSIPTGFIPPKMPDIWVMPRVAGDAVPLAVVSFAFTISLSEMFAKKYEYTVK
Sbjct  410992550  HVYGTSVSGSIPTGFIPPKMPDIWVMPRVAGDAVPLAVVSFAFTISLSEMFAKKYEYTVK  410992729

Query  393        ANQEMFAIGFCNIIPSFFHSFASSAALAKTLVKASTGCKTQVSSVVSAVVVLLVLLLFAP  452
                  ANQEMFAIGFCNIIPSFFHSFASSAALAKTLVKASTGCKTQVSSVVSAVVVLLVLLLFAP
Sbjct  410992730  ANQEMFAIGFCNIIPSFFHSFASSAALAKTLVKASTGCKTQVSSVVSAVVVLLVLLLFAP  410992909

Query  453        LFYSLQKCVLACIIIVSLREALWKFKDLPTRWHLSKVDCLVWCVTVLSSAFISTEMGLLV  512
                  LFYSLQKCVLACIIIVSLREALWKFKDLPTRWHLSKVDCLVWCVTVLSSAFISTEMGLLV
Sbjct  410992910  LFYSLQKCVLACIIIVSLREALWKFKDLPTRWHLSKVDCLVWCVTVLSSAFISTEMGLLV  410993089

Query  513        GVIFSMMCIIARLQVPHTALLSQIQNTVFYEDDGKYENLCPVPQVKIFRFEAPIYYANKG  572
                  GVIFSMMCIIARLQVPHTALLSQIQNTVFYEDDGKYENLCPVPQVKIFRFEAPIYYANKG
Sbjct  410993090  GVIFSMMCIIARLQVPHTALLSQIQNTVFYEDDGKYENLCPVPQVKIFRFEAPIYYANKG  410993269

Query  573        FFLQSLYKMTGLDPALEIIRRKKNEVKEKTFMKKGNKEIAAVKGNNKTDTNVNLVPAQLD  632
                  FFLQSLYKMTGLDPALEIIRRKKNEVKEKTFMKKGNKEIAAVKGNNKTDTNVNLVPAQLD
Sbjct  410993270  FFLQSLYKMTGLDPALEIIRRKKNEVKEKTFMKKGNKEIAAVKGNNKTDTNVNLVPAQLD  410993449
```

```
Query  633        FHTIILDCSSIPFLDTTGVNTLKGILKGYKEVDISIILSCCNPSVIDSLERSGYFGKDNK  692
                  FHTIILDCSSIPFLDTTGVNTLKGILKGYKEVDISIILSCCNPSVIDSLERSGYFGKDNK
Sbjct  410993450  FHTIILDCSSIPFLDTTGVNTLKGILKGYKEVDISIILSCCNPSVIDSLERSGYFGKDNK  410993629

Query  693        DIHELHFYSVHSAVHFARERKSIVADTTI  721
                  DIHELHFYSVHSAVHFARERKSIVADTTI
Sbjct  410993630  DIHELHFYSVHSAVHFARERKSIVADTTI  410993716
```

Range 2: 410987552 to 410988190

Score:813 bits(1659), Expect:0.0,
Method:,
Identities:213/213(100%), Positives:213/213(100%), Gaps:0/213(0%)

```
Query  1          MNANDMEREIEAIQLDKHPSCQIVMERKACINTNLQDTVKIKLKNNCFCTTKRLKNMFID  60
                  MNANDMEREIEAIQLDKHPSCQIVMERKACINTNLQDTVKIKLKNNCFCTTKRLKNMFID
Sbjct  410987552  MNANDMEREIEAIQLDKHPSCQIVMERKACINTNLQDTVKIKLKNNCFCTTKRLKNMFID  410987731

Query  61         FFPIIRWLPKYNCKEYIWGDVMSGLIIGIILIPQAIAYSLLAGLKPIYSLYTSFFANLIY  120
                  FFPIIRWLPKYNCKEYIWGDVMSGLIIGIILIPQAIAYSLLAGLKPIYSLYTSFFANLIY
Sbjct  410987732  FFPIIRWLPKYNCKEYIWGDVMSGLIIGIILIPQAIAYSLLAGLKPIYSLYTSFFANLIY  410987911

Query  121        FLLGTSRHISVGIFSLLSLMVGQVVDRELQLAGFDMNDDTQQTISNTSKWITDESNFTTF  180
                  FLLGTSRHISVGIFSLLSLMVGQVVDRELQLAGFDMNDDTQQTISNTSKWITDESNFTTF
Sbjct  410987912  FLLGTSRHISVGIFSLLSLMVGQVVDRELQLAGFDMNDDTQQTISNTSKWITDESNFTTF  410988091

Query  181        NISTDLMIMECGKECYAISVATALTFLAGVYQV  213
                  NISTDLMIMECGKECYAISVATALTFLAGVYQV
Sbjct  410988092  NISTDLMIMECGKECYAISVATALTFLAGVYQV  410988190
```

P West african lungfish *slc26a1*

Query: sulfate anion transporter 1-like [Protopterus annectens] Query ID: XP_043917074.1 Length: 749

>Protopterus annectens isolate FY-2018-M chromosome 2.part0, PAN1.0, whole genome shotgun sequence
Sequence ID: NC_056728.1 Length: 2000000000
Range 1: 876111868 to 876113415

Score:1914 bits(3910), Expect:0.0,
Method:,
Identities:516/516(100%), Positives:516/516(100%), Gaps:0/516(0%)

```
Query  234        QVLMAVFHLGFVSVYLAEPMLDGFATGASLTILTVQVKYLLGIKLPRSQGFGTLVITWIN  293
                  QVLMAVFHLGFVSVYLAEPMLDGFATGASLTILTVQVKYLLGIKLPRSQGFGTLVITWIN
Sbjct  876111868  QVLMAVFHLGFVSVYLAEPMLDGFATGASLTILTVQVKYLLGIKLPRSQGFGTLVITWIN  876112047

Query  294        IFKNIHNANVCDVITSAICIAVLVGAKELGDRYKQKLKIPLPTELVVVVVATLVSHYANF  353
                  IFKNIHNANVCDVITSAICIAVLVGAKELGDRYKQKLKIPLPTELVVVVVATLVSHYANF
Sbjct  876112048  IFKNIHNANVCDVITSAICIAVLVGAKELGDRYKQKLKIPLPTELVVVVVATLVSHYANF  876112227

Query  354        NEVYGSSVAGEIPTGFIPPKVPNFAMMYRVALDAVPLAIIGFAFTVSLSEMFAKKHGYTV  413
                  NEVYGSSVAGEIPTGFIPPKVPNFAMMYRVALDAVPLAIIGFAFTVSLSEMFAKKHGYTV
Sbjct  876112228  NEVYGSSVAGEIPTGFIPPKVPNFAMMYRVALDAVPLAIIGFAFTVSLSEMFAKKHGYTV  876112407

Query  414        RANQEMFAVGFCNIIPAFFHSFATSAALAKTLVKDSTGCQTQVSGVISAVVVLLVLLFFA  473
                  RANQEMFAVGFCNIIPAFFHSFATSAALAKTLVKDSTGCQTQVSGVISAVVVLLVLLFFA
Sbjct  876112408  RANQEMFAVGFCNIIPAFFHSFATSAALAKTLVKDSTGCQTQVSGVISAVVVLLVLLFFA  876112587

Query  474        PLFYSLQKCILACIIIVSLRGALRKFHELPQRWYVNKTDALIWIVTMLSSALISTEMGLL  533
                  PLFYSLQKCILACIIIVSLRGALRKFHELPQRWYVNKTDALIWIVTMLSSALISTEMGLL
Sbjct  876112588  PLFYSLQKCILACIIIVSLRGALRKFHELPQRWYVNKTDALIWIVTMLSSALISTEMGLL  876112767

Query  534        VGVVFSVICVIGRTQHPHIAVLGQIPNHTFYEDVSEYENLCLMQDIKIFRLQAPLYYANK  593
                  VGVVFSVICVIGRTQHPHIAVLGQIPNHTFYEDVSEYENLCLMQDIKIFRLQAPLYYANK
Sbjct  876112768  VGVVFSVICVIGRTQHPHIAVLGQIPNHTFYEDVSEYENLCLMQDIKIFRLQAPLYYANK  876112947

Query  594        DFFQKFIYKMSGVNPILEITKKQKAEREFKKQAKLEHRVKAVCNNAQTVCSNERNETVMH  653
                  DFFQKFIYKMSGVNPILEITKKQKAEREFKKQAKLEHRVKAVCNNAQTVCSNERNETVMH
Sbjct  876112948  DFFQKFIYKMSGVNPILEITKKQKAEREFKKQAKLEHRVKAVCNNAQTVCSNERNETVMH  876113127

Query  654        LVPNHWGFHTLIFDCSSIPFVDTAGISVLKGILRDYKEIGISVLLAGCSPSVIDSLQRAG  713
                  LVPNHWGFHTLIFDCSSIPFVDTAGISVLKGILRDYKEIGISVLLAGCSPSVIDSLQRAG
Sbjct  876113128  LVPNHWGFHTLIFDCSSIPFVDTAGISVLKGILRDYKEIGISVLLAGCSPSVIDSLQRAG  876113307

Query  714        YFGKNDENRNTSLFCTVHSAVLYAEDSQSREADSQV  749
                  YFGKNDENRNTSLFCTVHSAVLYAEDSQSREADSQV
Sbjct  876113308  YFGKNDENRNTSLFCTVHSAVLYAEDSQSREADSQV  876113415
```

Range 2: 875980653 to 875981357

Score:888 bits(1812), Expect:0.0,
Method:,
Identities:235/235(100%), Positives:235/235(100%), Gaps:0/235(0%)

```
Query  1          MKNKMNACVRHCMDLDYFCFYSILGLQKSLQLLPQKEGNNRLQMEKNLSLQIHLEQKAHV  60
                  MKNKMNACVRHCMDLDYFCFYSILGLQKSLQLLPQKEGNNRLQMEKNLSLQIHLEQKAHV
Sbjct  875980653  MKNKMNACVRHCMDLDYFCFYSILGLQKSLQLLPQKEGNNRLQMEKNLSLQIHLEQKAHV  875980832

Query  61         KKDAKTVLKDKIKRRCSCSKGRLKKQFVDFLPVLQWLPKYRFKEYIWGDVMSGLIIGIIL  120
                  KKDAKTVLKDKIKRRCSCSKGRLKKQFVDFLPVLQWLPKYRFKEYIWGDVMSGLIIGIIL
Sbjct  875980833  KKDAKTVLKDKIKRRCSCSKGRLKKQFVDFLPVLQWLPKYRFKEYIWGDVMSGLIIGIIL  875981012

Query  121        VPQAIAYSLLAGLKPIYSLYTSFFANIIYFFMGTSRHVSVGIFSLMSLLIGQVVDREVQL  180
                  VPQAIAYSLLAGLKPIYSLYTSFFANIIYFFMGTSRHVSVGIFSLMSLLIGQVVDREVQL
Sbjct  875981013  VPQAIAYSLLAGLKPIYSLYTSFFANIIYFFMGTSRHVSVGIFSLMSLLIGQVVDREVQL  875981192

Query  181        AGFDQSEYFNKQNVTDISPANFNITEHSMDQEYEKEHFAVGVAAAVTCLAGVYQV  235
                  AGFDQSEYFNKQNVTDISPANFNITEHSMDQEYEKEHFAVGVAAAVTCLAGVYQV
Sbjct  875981193  AGFDQSEYFNKQNVTDISPANFNITEHSMDQEYEKEHFAVGVAAAVTCLAGVYQV  875981357
```

Q Coelacanth *slc26a1*

Query: PREDICTED: sulfate transporter-like [Latimeria chalumnae] Query ID: XP_006011504.1 Length: 698

>Latimeria chalumnae isolate SAIAB 97564 unplaced genomic scaffold, LatCha1 scaffold02739, whole genome shotgun sequence
Sequence ID: NW_005821749.1 Length: 267182
Range 1: 72019 to 73548

Score:1880 bits(3841), Expect:0.0,
Method:,
Identities:510/510(100%), Positives:510/510(100%), Gaps:0/510(0%)

```
Query  189    QVLMGVFRLGFVSVYLSEPVLDGFATGASITILTVQVKFLLGLKIPRTQGFGTAVITWIN  248
              QVLMGVFRLGFVSVYLSEPVLDGFATGASITILTVQVKFLLGLKIPRTQGFGTAVITWIN
Sbjct  72019  QVLMGVFRLGFVSVYLSEPVLDGFATGASITILTVQVKFLLGLKIPRTQGFGTAVITWIN  72198

Query  249    IFRNIHKTNFCDVITSAICIAVLVGAKELTERYKHKIRFPLPTELVVVLVGTLVSHFANL  308
              IFRNIHKTNFCDVITSAICIAVLVGAKELTERYKHKIRFPLPTELVVVLVGTLVSHFANL
Sbjct  72199  IFRNIHKTNFCDVITSAICIAVLVGAKELTERYKHKIRFPLPTELVVVLVGTLVSHFANL  72378

Query  309    NELYGSSISGTIPTGFIAPRVPSFGLMPRVAIDAVPLAIISFAFTVSLSEMFAKKHGYSV  368
              NELYGSSISGTIPTGFIAPRVPSFGLMPRVAIDAVPLAIISFAFTVSLSEMFAKKHGYSV
Sbjct  72379  NELYGSSISGTIPTGFIAPRVPSFGLMPRVAIDAVPLAIISFAFTVSLSEMFAKKHGYSV  72558

Query  369    QANQEMFAIGFCNIIPSFFHSFATSAALAKSLVKESTGCQTQVSGLASAAVVLLVLLFFA  428
              QANQEMFAIGFCNIIPSFFHSFATSAALAKSLVKESTGCQTQVSGLASAAVVLLVLLFFA
Sbjct  72559  QANQEMFAIGFCNIIPSFFHSFATSAALAKSLVKESTGCQTQVSGLASAAVVLLVLLFFA  72738

Query  429    PFFYSLQKCVLACIIIVSLRGALRKFQDVPKRWQVNKVDTLIWSITMLSSAMISTEMGLL  488
              PFFYSLQKCVLACIIIVSLRGALRKFQDVPKRWQVNKVDTLIWSITMLSSAMISTEMGLL
Sbjct  72739  PFFYSLQKCVLACIIIVSLRGALRKFQDVPKRWQVNKVDTLIWSITMLSSAMISTEMGLL  72918

Query  489    VGVVFSIMCIIARTQLPHMAILGQIQNSVFYEDTKEYENLSPIPGVKIIRFEAPLYYANK  548
              VGVVFSIMCIIARTQLPHMAILGQIQNSVFYEDTKEYENLSPIPGVKIIRFEAPLYYANK
Sbjct  72919  VGVVFSIMCIIARTQLPHMAILGQIQNSVFYEDTKEYENLSPIPGVKIIRFEAPLYYANK  73098

Query  549    DFFLKSLYKITGVDPAWEAAKRKKAENSKRVPTKNEKKENEVSNGSMQGDTTLQLVPKQF  608
              DFFLKSLYKITGVDPAWEAAKRKKAENSKRVPTKNEKKENEVSNGSMQGDTTLQLVPKQF
Sbjct  73099  DFFLKSLYKITGVDPAWEAAKRKKAENSKRVPTKNEKKENEVSNGSMQGDTTLQLVPKQF  73278

Query  609    DFHTIILDCSSIQFLDSVGINTLKGIAKDYKEVGIRIILCCCNPSVIDSLERGNYFGTHC  668
              DFHTIILDCSSIQFLDSVGINTLKGIAKDYKEVGIRIILCCCNPSVIDSLERGNYFGTHC
Sbjct  73279  DFHTIILDCSSIQFLDSVGINTLKGIAKDYKEVGIRIILCCCNPSVIDSLERGNYFGTHC  73458

Query  669    KDINTLLFYTVHNAVEFAASSLPATADSSV  698
              KDINTLLFYTVHNAVEFAASSLPATADSSV
Sbjct  73459  KDINTLLFYTVHNAVEFAASSLPATADSSV  73548
```

Range 2: 66441 to 67010

Score:715 bits(1459), Expect:0.0,
Method:,
Identities:190/190(100%), Positives:190/190(100%), Gaps:0/190(0%)

```
Query  1      MERKICKGKDIKDIIRNKLKKSCSFSTNRMKYFFIDTLPVIKWLPKYNFKEWIWGDNMSG  60
              MERKICKGKDIKDIIRNKLKKSCSFSTNRMKYFFIDTLPVIKWLPKYNFKEWIWGDNMSG
Sbjct  66441  MERKICKGKDIKDIIRNKLKKSCSFSTNRMKYFFIDTLPVIKWLPKYNFKEWIWGDNMSG  66620

Query  61     LIIGIILVPQSIAYSLLAGLQPIYSLYTSLFANIIYFLMGTSKHVSVGIFSLLCLMVGQV  120
              LIIGIILVPQSIAYSLLAGLQPIYSLYTSLFANIIYFLMGTSKHVSVGIFSLLCLMVGQV
Sbjct  66621  LIIGIILVPQSIAYSLLAGLQPIYSLYTSLFANIIYFLMGTSKHVSVGIFSLLCLMVGQV  66800

Query  121    VDREMQLAGFDLSEDSQKGTQGTTDNWNASDANRSAFFVSLGPVHAECGKECYAISIATA  180
              VDREMQLAGFDLSEDSQKGTQGTTDNWNASDANRSAFFVSLGPVHAECGKECYAISIATA
Sbjct  66801  VDREMQLAGFDLSEDSQKGTQGTTDNWNASDANRSAFFVSLGPVHAECGKECYAISIATA  66980

Query  181    LTFVTGVYQV  190
              LTFVTGVYQV
Sbjct  66981  LTFVTGVYQV  67010
```

R Reedfish *slc26a1*

Query: sulfate anion transporter 1 [Erpetoichthys calabaricus] Query ID: XP_028658423.2 Length: 707

```
>Erpetoichthys calabaricus chromosome 5, fErpCal1.3, whole genome shotgun sequence
Sequence ID: NC_041398.2 Length: 252032905
Range 1: 223990395 to 223991915

Score:1865 bits(3811), Expect:0.0,
Method:,
Identities:507/507(100%), Positives:507/507(100%), Gaps:0/507(0%)

Query  201        VLMAVLRLGFVSIYLSSPMLDGFATGASLTILTVQAKYLLGVKIPRHQGFGNFVITWINI  260
                  VLMAVLRLGFVSIYLSSPMLDGFATGASLTILTVQAKYLLGVKIPRHQGFGNFVITWINI
Sbjct  223990395  VLMAVLRLGFVSIYLSSPMLDGFATGASLTILTVQAKYLLGVKIPRHQGFGNFVITWINI  223990574

Query  261        FKNIHKTNFCDLITSALCISVLVAAKELGDRYKHKLKIPLPTELFVVVTATLVSHFADLN  320
                  FKNIHKTNFCDLITSALCISVLVAAKELGDRYKHKLKIPLPTELFVVVTATLVSHFADLN
Sbjct  223990575  FKNIHKTNFCDLITSALCISVLVAAKELGDRYKHKLKIPLPTELFVVVTATLVSHFADLN  223990754

Query  321        GVYGSSISGAIPTGFIMPQVPSLNLMSRVALDAIPLAVISFAFTVSLSEMFAKKHGYTVR  380
                  GVYGSSISGAIPTGFIMPQVPSLNLMSRVALDAIPLAVISFAFTVSLSEMFAKKHGYTVR
Sbjct  223990755  GVYGSSISGAIPTGFIMPQVPSLNLMSRVALDAIPLAVISFAFTVSLSEMFAKKHGYTVR  223990934

Query  381        PNQEMLAIGFCNIIPSFFHCFTTSAALAKTMVKDSTGCQTQVSSVISALVVLLVLLFFAP  440
                  PNQEMLAIGFCNIIPSFFHCFTTSAALAKTMVKDSTGCQTQVSSVISALVVLLVLLFFAP
Sbjct  223990935  PNQEMLAIGFCNIIPSFFHCFTTSAALAKTMVKDSTGCQTQVSSVISALVVLLVLLFFAP  223991114

Query  441        LFYSLQKCVLACIIIVSLRGALRKFRDVPKQWRISKVDTLVWIVSMLSAALISTEMGLLV  500
                  LFYSLQKCVLACIIIVSLRGALRKFRDVPKQWRISKVDTLVWIVSMLSAALISTEMGLLV
Sbjct  223991115  LFYSLQKCVLACIIIVSLRGALRKFRDVPKQWRISKVDTLVWIVSMLSAALISTEMGLLV  223991294

Query  501        GVVFSMFCIVARTQLPHVAILGQIQGSVFYEDAGEYENLTAVPKVKIFRFQAPLYYANKD  560
                  GVVFSMFCIVARTQLPHVAILGQIQGSVFYEDAGEYENLTAVPKVKIFRFQAPLYYANKD
Sbjct  223991295  GVVFSMFCIVARTQLPHVAILGQIQGSVFYEDAGEYENLTAVPKVKIFRFQAPLYYANKD  223991474

Query  561        FFLRSLYKATRLDPLLEIAKRKKAEKKAKQLAAKRAKENVQDNGNCQVNQILVPKDHEFH  620
                  FFLRSLYKATRLDPLLEIAKRKKAEKKAKQLAAKRAKENVQDNGNCQVNQILVPKDHEFH
Sbjct  223991475  FFLRSLYKATRLDPLLEIAKRKKAEKKAKQLAAKRAKENVQDNGNCQVNQILVPKDHEFH  223991654

Query  621        TIVLDCSAIQFLDTSGISTLKGVRNDYKEVGISVLLASCNPCVLDTLKRGSYFGPNEKDI  680
                  TIVLDCSAIQFLDTSGISTLKGVRNDYKEVGISVLLASCNPCVLDTLKRGSYFGPNEKDI
Sbjct  223991655  TIVLDCSAIQFLDTSGISTLKGVRNDYKEVGISVLLASCNPCVLDTLKRGSYFGPNEKDI  223991834

Query  681        STLMFYTIHSAVQFAGRREIAAFDSEV  707
                  STLMFYTIHSAVQFAGRREIAAFDSEV
Sbjct  223991835  STLMFYTIHSAVQFAGRREIAAFDSEV  223991915



Range 2: 223986714 to 223987316

Score:748 bits(1526), Expect:0.0,
Method:,
Identities:201/201(100%), Positives:201/201(100%), Gaps:0/201(0%)

Query  1          MEDAKLDLYFQLNRKEPEQKNIKRLIKSKIQKSCSFSFKKVQPALTGFFPVVKWLPKYRF  60
                  MEDAKLDLYFQLNRKEPEQKNIKRLIKSKIQKSCSFSFKKVQPALTGFFPVVKWLPKYRF
Sbjct  223986714  MEDAKLDLYFQLNRKEPEQKNIKRLIKSKIQKSCSFSFKKVQPALTGFFPVVKWLPKYRF  223986893

Query  61         KEYVWGDVMSGLIIGIILVPQAIAYCLLAGLQPIYGLYTSFFSNIIYFLMGTSRHISVGI  120
                  KEYVWGDVMSGLIIGIILVPQAIAYCLLAGLQPIYGLYTSFFSNIIYFLMGTSRHISVGI
Sbjct  223986894  KEYVWGDVMSGLIIGIILVPQAIAYCLLAGLQPIYGLYTSFFSNIIYFLMGTSRHISVGI  223987073

Query  121        FSLLSLMVGQVVDREVQLAGFDLSEDSRLEGISPEDLFNSSKTNVSSVNLTLGVFNMECG  180
                  FSLLSLMVGQVVDREVQLAGFDLSEDSRLEGISPEDLFNSSKTNVSSVNLTLGVFNMECG
Sbjct  223987074  FSLLSLMVGQVVDREVQLAGFDLSEDSRLEGISPEDLFNSSKTNVSSVNLTLGVFNMECG  223987253

Query  181        KECYAIGVATALTFLAGVYQV  201
                  KECYAIGVATALTFLAGVYQV
Sbjct  223987254  KECYAIGVATALTFLAGVYQV  223987316
```

S Gray bichir *slc26a1*

Query: sulfate anion transporter 1 [Polypterus senegalus] Query ID: XP_039606315.1 Length: 707

```
>Polypterus senegalus isolate Bchr_013 chromosome 4, ASM1683550v1, whole genome shotgun sequence
Sequence ID: NC_053157.1 Length: 254417793
Range 1: 28433580 to 28435100

Score:1866 bits(3813), Expect:0.0,
Method:,
Identities:507/507(100%), Positives:507/507(100%), Gaps:0/507(0%)
```

```
Query  201       VLMAVLRLGFVSIYLSSPMLDGFATGASLTILTVQAKYLLGVKIPRHQGFGNFVITWINI  260
                 VLMAVLRLGFVSIYLSSPMLDGFATGASLTILTVQAKYLLGVKIPRHQGFGNFVITWINI
Sbjct  28435100  VLMAVLRLGFVSIYLSSPMLDGFATGASLTILTVQAKYLLGVKIPRHQGFGNFVITWINI  28434921

Query  261       LKNIHKTNFCDLITSALCISVLVAAKELGDRYKHKLKIPLPTELFVVVTATLVSHFADLN  320
                 LKNIHKTNFCDLITSALCISVLVAAKELGDRYKHKLKIPLPTELFVVVTATLVSHFADLN
Sbjct  28434920  LKNIHKTNFCDLITSALCISVLVAAKELGDRYKHKLKIPLPTELFVVVTATLVSHFADLN  28434741

Query  321       GVYGSSISGAIPTGFIMPQVPSLNLMSRVALDAIPLAVISFAFTVSLSEMFAKKHGYTVR  380
                 GVYGSSISGAIPTGFIMPQVPSLNLMSRVALDAIPLAVISFAFTVSLSEMFAKKHGYTVR
Sbjct  28434740  GVYGSSISGAIPTGFIMPQVPSLNLMSRVALDAIPLAVISFAFTVSLSEMFAKKHGYTVR  28434561

Query  381       PNQEMLAIGFCNIIPSFFHCFTTSAALAKTMVKDSTGCQTQVSSVISALVVLLVLLFFAP  440
                 PNQEMLAIGFCNIIPSFFHCFTTSAALAKTMVKDSTGCQTQVSSVISALVVLLVLLFFAP
Sbjct  28434560  PNQEMLAIGFCNIIPSFFHCFTTSAALAKTMVKDSTGCQTQVSSVISALVVLLVLLFFAP  28434381

Query  441       LFYSLQKCVLACIIIVSLRGALRKFRDVPKQWRISKVDTLIWIVSMLSAALISTEMGLLV  500
                 LFYSLQKCVLACIIIVSLRGALRKFRDVPKQWRISKVDTLIWIVSMLSAALISTEMGLLV
Sbjct  28434380  LFYSLQKCVLACIIIVSLRGALRKFRDVPKQWRISKVDTLIWIVSMLSAALISTEMGLLV  28434201

Query  501       GVVFSMFCIVARTQLPHVAILGQIQGSVFYEDAGEYENLTAVPKVKIFRFQAPLYYANKD  560
                 GVVFSMFCIVARTQLPHVAILGQIQGSVFYEDAGEYENLTAVPKVKIFRFQAPLYYANKD
Sbjct  28434200  GVVFSMFCIVARTQLPHVAILGQIQGSVFYEDAGEYENLTAVPKVKIFRFQAPLYYANKD  28434021

Query  561       FFLRSLYKATRLDPLLEIAKRNKAEKKAKQLAAKRAKENVQDNGNCQVNQILVPKNHEFH  620
                 FFLRSLYKATRLDPLLEIAKRNKAEKKAKQLAAKRAKENVQDNGNCQVNQILVPKNHEFH
Sbjct  28434020  FFLRSLYKATRLDPLLEIAKRNKAEKKAKQLAAKRAKENVQDNGNCQVNQILVPKNHEFH  28433841

Query  621       TIVLDCSAIQFLDTSGISTLKGVRNDYKEVGISFLLASCNPCVLDTLKRASYFGPNEKDI  680
                 TIVLDCSAIQFLDTSGISTLKGVRNDYKEVGISFLLASCNPCVLDTLKRASYFGPNEKDI
Sbjct  28433840  TIVLDCSAIQFLDTSGISTLKGVRNDYKEVGISFLLASCNPCVLDTLKRASYFGPNEKDI  28433661

Query  681       STLMFYTIHSAVQFAGRREIAAFDSEV  707
                 STLMFYTIHSAVQFAGRREIAAFDSEV
Sbjct  28433660  STLMFYTIHSAVQFAGRREIAAFDSEV  28433580
```

Range 2: 28438843 to 28439445

Score:751 bits(1533), Expect:0.0,
Method:,
Identities:201/201(100%), Positives:201/201(100%), Gaps:0/201(0%)

```
Query  1         MEEAKLDLYFQLHRKEPEQKNIKRLIKSKIQKSCSFSFKKVQSALTGFFPVVKWLPKYRF  60
                 MEEAKLDLYFQLHRKEPEQKNIKRLIKSKIQKSCSFSFKKVQSALTGFFPVVKWLPKYRF
Sbjct  28439445  MEEAKLDLYFQLHRKEPEQKNIKRLIKSKIQKSCSFSFKKVQSALTGFFPVVKWLPKYRF  28439266

Query  61        KEYVWGDVMSGLIIGIILVPQAIAYCLLAGLQPIYGLYTSFFSNIIYFLMGTSRHISVGI  120
                 KEYVWGDVMSGLIIGIILVPQAIAYCLLAGLQPIYGLYTSFFSNIIYFLMGTSRHISVGI
Sbjct  28439265  KEYVWGDVMSGLIIGIILVPQAIAYCLLAGLQPIYGLYTSFFSNIIYFLMGTSRHISVGI  28439086

Query  121       FSLLSLMVGQVVDREVQLAGFDLSEDNRWEGISPEDLFNSSKTNVSSVNLTLGVFNMECG  180
                 FSLLSLMVGQVVDREVQLAGFDLSEDNRWEGISPEDLFNSSKTNVSSVNLTLGVFNMECG
Sbjct  28439085  FSLLSLMVGQVVDREVQLAGFDLSEDNRWEGISPEDLFNSSKTNVSSVNLTLGVFNMECG  28438906

Query  181       KECYAIGVATALTFLAGVYQV  201
                 KECYAIGVATALTFLAGVYQV
Sbjct  28438905  KECYAIGVATALTFLAGVYQV  28438843
```

T Sterlet *slc26a1*

Query: sulfate anion transporter 1-like [Acipenser ruthenus] Query ID: XP_033871528.1 Length: 709

>Acipenser ruthenus chromosome 2, fAciRut3.2 maternal haplotype, whole genome shotgun sequence
Sequence ID: NC_081190.1 Length: 117442607
Range 1: 35179051 to 35180571

Score:1849 bits(3778), Expect:0.0,
Method:,
Identities:507/507(100%), Positives:507/507(100%), Gaps:0/507(0%)

```
Query  203       VLMAVFQLGFVSVYLSGPMLDGFATGASLTILTVQVKYLLGIKIPRTQGFGTLIITWINI  262
                 VLMAVFQLGFVSVYLSGPMLDGFATGASLTILTVQVKYLLGIKIPRTQGFGTLIITWINI
Sbjct  35180571  VLMAVFQLGFVSVYLSGPMLDGFATGASLTILTVQVKYLLGIKIPRTQGFGTLIITWINI  35180392

Query  263       FKNIQNTNSCDVITSAICIAVLVAAKELGDRYKDRLKIPLPTELVVVVTATLVSHFADLN  322
                 FKNIQNTNSCDVITSAICIAVLVAAKELGDRYKDRLKIPLPTELVVVVTATLVSHFADLN
Sbjct  35180391  FKNIQNTNSCDVITSAICIAVLVAAKELGDRYKDRLKIPLPTELVVVVTATLVSHFADLN  35180212

Query  323       GVYGSSISGAIPTGFIQPQVPSLGMMPRVALDAIPLAVISFAFTVSLAEMFAKKHGYTVR  382
                 GVYGSSISGAIPTGFIQPQVPSLGMMPRVALDAIPLAVISFAFTVSLAEMFAKKHGYTVR
Sbjct  35180211  GVYGSSISGAIPTGFIQPQVPSLGMMPRVALDAIPLAVISFAFTVSLAEMFAKKHGYTVR  35180032

Query  383       PNQEMLAIGFCNIIPSFFHCFTTSAALAKTLVKDSTGCHTQVSSLISAFVVLLVLLFFAP  442
                 PNQEMLAIGFCNIIPSFFHCFTTSAALAKTLVKDSTGCHTQVSSLISAFVVLLVLLFFAP
Sbjct  35180031  PNQEMLAIGFCNIIPSFFHCFTTSAALAKTLVKDSTGCHTQVSSLISAFVVLLVLLFFAP  35179852
```

```
Query  443       LFHSLQKCVLACIIIVSLRGALRKFRSVPGQWQINKVDTLIWIVTMLSSALISTEMGLLV  502
                 LFHSLQKCVLACIIIVSLRGALRKFRSVPGQWQINKVDTLIWIVTMLSSALISTEMGLLV
Sbjct  35179851  LFHSLQKCVLACIIIVSLRGALRKFRSVPGQWQINKVDTLIWIVTMLSSALISTEMGLLV  35179672

Query  503       GVVFSILCVVARTQLPHVAVLGQIQDSVFYEDSKEYKNLSTVPKVKIFRFQAPLYYANKD  562
                 GVVFSILCVVARTQLPHVAVLGQIQDSVFYEDSKEYKNLSTVPKVKIFRFQAPLYYANKD
Sbjct  35179671  GVVFSILCVVARTQLPHVAVLGQIQDSVFYEDSKEYKNLSTVPKVKIFRFQAPLYYANKD  35179492

Query  563       FFLRSLYKTSGLDPVLEEAKRKKAEKKAKAKAAKQAKENGQEKSNGDTPLILTPAGLDFH  622
                 FFLRSLYKTSGLDPVLEEAKRKKAEKKAKAKAAKQAKENGQEKSNGDTPLILTPAGLDFH
Sbjct  35179491  FFLRSLYKTSGLDPVLEEAKRKKAEKKAKAKAAKQAKENGQEKSNGDTPLILTPAGLDFH  35179312

Query  623       TIILDCSSIQFLDTAGLVTLKGILKDYKEIGISILLACCNTSVIDSLKQGSYFGTYEKDI  682
                 TIILDCSSIQFLDTAGLVTLKGILKDYKEIGISILLACCNTSVIDSLKQGSYFGTYEKDI
Sbjct  35179311  TIILDCSSIQFLDTAGLVTLKGILKDYKEIGISILLACCNTSVIDSLKQGSYFGTYEKDI  35179132

Query  683       STLLFYTVHSAVQFAVSREVAIGDSVV  709
                 STLLFYTVHSAVQFAVSREVAIGDSVV
Sbjct  35179131  STLLFYTVHSAVQFAVSREVAIGDSVV  35179051
```

Range 2: 35181178 to 35181786

Score:761 bits(1554), Expect:0.0,
Method:,
Identities:203/203(100%), Positives:203/203(100%), Gaps:0/203(0%)

```
Query  1         MEGSTLENISYLLERRARKQWNPKEVLKGKLSKSCSCSVQKLKNSFIGFFPVVRWLPKYK  60
                 MEGSTLENISYLLERRARKQWNPKEVLKGKLSKSCSCSVQKLKNSFIGFFPVVRWLPKYK
Sbjct  35181786  MEGSTLENISYLLERRARKQWNPKEVLKGKLSKSCSCSVQKLKNSFIGFFPVVRWLPKYK  35181607

Query  61        LKEHVWGDIMSGLIVGIILVPQAIAYCLLAGLQPINGLYTSFFANIIYFFMGTSRHVSVG  120
                 LKEHVWGDIMSGLIVGIILVPQAIAYCLLAGLQPINGLYTSFFANIIYFFMGTSRHVSVG
Sbjct  35181606  LKEHVWGDIMSGLIVGIILVPQAIAYCLLAGLQPINGLYTSFFANIIYFFMGTSRHVSVG  35181427

Query  121       IFSLMSLMVGQVVDREVQLAGFDMSDDSQVVAMGPEDLWYGSNTANITKLNSTLGAFNME  180
                 IFSLMSLMVGQVVDREVQLAGFDMSDDSQVVAMGPEDLWYGSNTANITKLNSTLGAFNME
Sbjct  35181426  IFSLMSLMVGQVVDREVQLAGFDMSDDSQVVAMGPEDLWYGSNTANITKLNSTLGAFNME  35181247

Query  181       CGKDCYAMSVAAALTFIAGVYQV  203
                 CGKDCYAMSVAAALTFIAGVYQV
Sbjct  35181246  CGKDCYAMSVAAALTFIAGVYQV  35181178
```

U Spotted gar *slc26a1*

Query: PREDICTED: sulfate anion transporter 1-like [Lepisosteus oculatus] Query ID: XP_006627221.1 Length: 708

>Lepisosteus oculatus isolate Spotted Gar 1 linkage group LG2 genomic scaffold, LepOcu1, whole genome shotgun sequence
Sequence ID: NW_006269884.1 Length: 6854591
Range 1: 6777027 to 6778547

Score:1866 bits(3813), Expect:0.0,
Method:,
Identities:507/507(100%), Positives:507/507(100%), Gaps:0/507(0%)

```
Query  202      VLMAVFQLGFVSVYLSAPMLDGFATGASLTILTVQAKYLLGLKIPRHQGFGTVIVTWINI  261
                VLMAVFQLGFVSVYLSAPMLDGFATGASLTILTVQAKYLLGLKIPRHQGFGTVIVTWINI
Sbjct  6778547  VLMAVFQLGFVSVYLSAPMLDGFATGASLTILTVQAKYLLGLKIPRHQGFGTVIVTWINI  6778368

Query  262      FKNIHKTNFCDMITSAICIAVLVGAKELGERYKDRLKIPLPTELVVVTVATLVSHFTDLN  321
                FKNIHKTNFCDMITSAICIAVLVGAKELGERYKDRLKIPLPTELVVVTVATLVSHFTDLN
Sbjct  6778367  FKNIHKTNFCDMITSAICIAVLVGAKELGERYKDRLKIPLPTELVVVTVATLVSHFTDLN  6778188

Query  322      GLYGSSISGAIPTGFIPPKVPSFELMPRVAYDAIPLAVISFAFTVSLSEMFAKKNGYTVR  381
                GLYGSSISGAIPTGFIPPKVPSFELMPRVAYDAIPLAVISFAFTVSLSEMFAKKNGYTVR
Sbjct  6778187  GLYGSSISGAIPTGFIPPKVPSFELMPRVAYDAIPLAVISFAFTVSLSEMFAKKNGYTVR  6778008

Query  382      PNQEMMAIGFCNIIPSFFHCFTTSAALAKTMVKDSTGCQTQVSSIISALVVLLILLVFAP  441
                PNQEMMAIGFCNIIPSFFHCFTTSAALAKTMVKDSTGCQTQVSSIISALVVLLILLVFAP
Sbjct  6778007  PNQEMMAIGFCNIIPSFFHCFTTSAALAKTMVKDSTGCQTQVSSIISALVVLLILLVFAP  6777828

Query  442      LFYSLQKCVLACIIIVSLRGALRKFRDVPKQWHLNKVDAIVWSITMLSSALISVEMGLLV  501
                LFYSLQKCVLACIIIVSLRGALRKFRDVPKQWHLNKVDAIVWSITMLSSALISVEMGLLV
Sbjct  6777827  LFYSLQKCVLACIIIVSLRGALRKFRDVPKQWHLNKVDAIVWSITMLSSALISVEMGLLV  6777648

Query  502      GVIFSMLCILVRTQVPQVAILSQIQDTVFYEDSGEYDNLLVVPKVKIFRFQAPLYYANKD  561
                GVIFSMLCILVRTQVPQVAILSQIQDTVFYEDSGEYDNLLVVPKVKIFRFQAPLYYANKD
Sbjct  6777647  GVIFSMLCILVRTQVPQVAILSQIQDTVFYEDSGEYDNLLVVPKVKIFRFQAPLYYANKD  6777468

Query  562      FFLKSLYKAVGLEPFLEVTKRRKAEKKAKELANKQVTVNCLEKNNGDVTTSLVSQELDFH  621
                FFLKSLYKAVGLEPFLEVTKRRKAEKKAKELANKQVTVNCLEKNNGDVTTSLVSQELDFH
Sbjct  6777467  FFLKSLYKAVGLEPFLEVTKRRKAEKKAKELANKQVTVNCLEKNNGDVTTSLVSQELDFH  6777288

Query  622      TIILDCCSIPFLDSTGIVTFKGVLKEYKEVGVSIILACCNPSVIDSLKRGGFFGTDDKDI  681
                TIILDCCSIPFLDSTGIVTFKGVLKEYKEVGVSIILACCNPSVIDSLKRGGFFGTDDKDI
Sbjct  6777287  TIILDCCSIPFLDSTGIVTFKGVLKEYKEVGVSIILACCNPSVIDSLKRGGFFGTDDKDI  6777108

Query  682      SNLSFYSVHSAVLFANSREPAVSQSVV  708
```

```
                SNLSFYSVHSAVLFANSREPAVSQSVV
Sbjct  6777107  SNLSFYSVHSAVLFANSREPAVSQSVV  6777027
```

Range 2: 6778764 to 6779369

Score:751 bits(1532), Expect:0.0,
Method:,
Identities:202/202(100%), Positives:202/202(100%), Gaps:0/202(0%)

```
Query  1        MEEEGSENVCHLERRTRRRRDLKAVLRKKLSSSFSCSAKKLKSNVTGFFPVVQWLPKYKF  60
                MEEEGSENVCHLERRTRRRRDLKAVLRKKLSSSFSCSAKKLKSNVTGFFPVVQWLPKYKF
Sbjct  6779369  MEEEGSENVCHLERRTRRRRDLKAVLRKKLSSSFSCSAKKLKSNVTGFFPVVQWLPKYKF  6779190

Query  61       KEYVWGDVMSGLIVGIILVPQAIAYCLLAGLQPIYGLYTSFFANIIYFFMGTSRHVSVGI  120
                KEYVWGDVMSGLIVGIILVPQAIAYCLLAGLQPIYGLYTSFFANIIYFFMGTSRHVSVGI
Sbjct  6779189  KEYVWGDVMSGLIVGIILVPQAIAYCLLAGLQPIYGLYTSFFANIIYFFMGTSRHVSVGI  6779010

Query  121      FSLMSLMVGQVVDREMYLAGFDLSEDNKADTLNSGDVWNSSGTSNVSAINLTLGDLSMEC  180
                FSLMSLMVGQVVDREMYLAGFDLSEDNKADTLNSGDVWNSSGTSNVSAINLTLGDLSMEC
Sbjct  6779009  FSLMSLMVGQVVDREMYLAGFDLSEDNKADTLNSGDVWNSSGTSNVSAINLTLGDLSMEC  6778830

Query  181      GKECYAISIAAALTFLAGVYQV  202
                GKECYAISIAAALTFLAGVYQV
Sbjct  6778829  GKECYAISIAAALTFLAGVYQV  6778764
```

V Asian bonytongue *slc26a1*

Query: sulfate anion transporter 1-like [Scleropages formosus] Query ID: XP_029108491.1 Length: 707

>Scleropages formosus chromosome 6, fSclFor1.1, whole genome shotgun sequence
Sequence ID: NC_041811.1 Length: 38292110
Range 1: 29396211 to 29397731

Score:1870 bits(3821), Expect:0.0,
Method:,
Identities:507/507(100%), Positives:507/507(100%), Gaps:0/507(0%)

```
Query  201       QVLMALLRLGFVSVYLSAPMLDGFATGASVTILTVQAKYLLGLKIPRHQGCGTVIVTWIN  260
                 QVLMALLRLGFVSVYLSAPMLDGFATGASVTILTVQAKYLLGLKIPRHQGCGTVIVTWIN
Sbjct  29397731  QVLMALLRLGFVSVYLSAPMLDGFATGASVTILTVQAKYLLGLKIPRHQGCGTVIVTWIN  29397552

Query  261       IFKAIHKTNFCDMITSTICIAVLVSGKELQDRFKDRLKIPLPTELVVVAVATLVSHFADL  320
                 IFKAIHKTNFCDMITSTICIAVLVSGKELQDRFKDRLKIPLPTELVVVAVATLVSHFADL
Sbjct  29397551  IFKAIHKTNFCDMITSTICIAVLVSGKELQDRFKDRLKIPLPTELVVVAVATLVSHFADL  29397372

Query  321       NGQYSSSISGAIPTGFIPPKVPSVELMPRVALDAIPLAVISFAFTVSLSEMFAKKNGYTV  380
                 NGQYSSSISGAIPTGFIPPKVPSVELMPRVALDAIPLAVISFAFTVSLSEMFAKKNGYTV
Sbjct  29397371  NGQYSSSISGAIPTGFIPPKVPSVELMPRVALDAIPLAVISFAFTVSLSEMFAKKNGYTV  29397192

Query  381       RPNQEMMAIGFCNIIPSFFHCFTTSAALAKTMVKDSTGCQTQVSSVVSAFVVLLILLVFA  440
                 RPNQEMMAIGFCNIIPSFFHCFTTSAALAKTMVKDSTGCQTQVSSVVSAFVVLLILLVFA
Sbjct  29397191  RPNQEMMAIGFCNIIPSFFHCFTTSAALAKTMVKDSTGCQTQVSSVVSAFVVLLILLVFA  29397012

Query  441       PFFYALQKCVLACIIIVSLRGALRKFCDVPRQWRINKVDCIVWMVTMSASALLSVELGLL  500
                 PFFYALQKCVLACIIIVSLRGALRKFCDVPRQWRINKVDCIVWMVTMSASALLSVELGLL
Sbjct  29397011  PFFYALQKCVLACIIIVSLRGALRKFCDVPRQWRINKVDCIVWMVTMSASALLSVELGLL  29396832

Query  501       VGVVFSMLCVVFQTQKPKVALMGQIQGSNHYEDIEEYNDLLTVPKVRIVRFQAPLYYANK  560
                 VGVVFSMLCVVFQTQKPKVALMGQIQGSNHYEDIEEYNDLLTVPKVRIVRFQAPLYYANK
Sbjct  29396831  VGVVFSMLCVVFQTQKPKVALMGQIQGSNHYEDIEEYNDLLTVPKVRIVRFQAPLYYANK  29396652

Query  561       DYFLTSLYKAVDLEPVIEISKMKKAEKKAKMLAAKKTGETSPERTNGDVTAGLVSRELDF  620
                 DYFLTSLYKAVDLEPVIEISKMKKAEKKAKMLAAKKTGETSPERTNGDVTAGLVSRELDF
Sbjct  29396651  DYFLTSLYKAVDLEPVIEISKMKKAEKKAKMLAAKKTGETSPERTNGDVTAGLVSRELDF  29396472

Query  621       HTLILDCSSMPFIDTTGMVTLKGLVKDYGEVGVRVVLACCQPSVIDALKRGSFFGVADKD  680
                 HTLILDCSSMPFIDTTGMVTLKGLVKDYGEVGVRVVLACCQPSVIDALKRGSFFGVADKD
Sbjct  29396471  HTLILDCSSMPFIDTTGMVTLKGLVKDYGEVGVRVVLACCQPSVIDALKRGSFFGVADKD  29396292

Query  681       IHTLFFHTVHSAVLFAQDKAGDRDSEV  707
                 IHTLFFHTVHSAVLFAQDKAGDRDSEV
Sbjct  29396291  IHTLFFHTVHSAVLFAQDKAGDRDSEV  29396211
```

Range 2: 29397843 to 29398448

Score:757 bits(1546), Expect:0.0,
Method:,
Identities:202/202(100%), Positives:202/202(100%), Gaps:0/202(0%)

```
Query  1         MADHKWAEVGHLQRRVRGSPDMRQAIRTRMKKSVSCSVARLKEALTGFFPVLLWLPKYKL  60
                 MADHKWAEVGHLQRRVRGSPDMRQAIRTRMKKSVSCSVARLKEALTGFFPVLLWLPKYKL
Sbjct  29398448  MADHKWAEVGHLQRRVRGSPDMRQAIRTRMKKSVSCSVARLKEALTGFFPVLLWLPKYKL  29398269

Query  61        KEYVWGDVMSGLIVGIILVPQAIAYCLLAGLEPIYGLYTSFYANIIYFLMGTSKHVSVGI  120
```

```
                 KEYVWGDVMSGLIVGIILVPQAIAYCLLAGLEPIYGLYTSFYANIIYFLMGTSKHVSVGI
Sbjct  29398268  KEYVWGDVMSGLIVGIILVPQAIAYCLLAGLEPIYGLYTSFYANIIYFLMGTSKHVSVGI  29398089

Query  121       FSLMSLMVGQVVDREVFLAGFDMGDDNKLDPAALGDEWNETSTPILSTINMTLGVYELEC  180
                 FSLMSLMVGQVVDREVFLAGFDMGDDNKLDPAALGDEWNETSTPILSTINMTLGVYELEC
Sbjct  29398088  FSLMSLMVGQVVDREVFLAGFDMGDDNKLDPAALGDEWNETSTPILSTINMTLGVYELEC  29397909

Query  181       GKECYAISVAAALTFLAGVYQV  202
                 GKECYAISVAAALTFLAGVYQV
Sbjct  29397908  GKECYAISVAAALTFLAGVYQV  29397843
```

W Zebrafish *slc26a1*

Query: sulfate anion transporter 1 isoform X1 [Danio rerio] Query ID: XP_005161263.1 Length: 711

>Danio rerio strain Tuebingen chromosome 21, GRCz11 Primary Assembly
Sequence ID: NC_007132.7 Length: 45934066
Range 1: 20325099 to 20326616

Score:1873 bits(3827), Expect:0.0,
Method:,
Identities:506/506(100%), Positives:506/506(100%), Gaps:0/506(0%)

```
Query  206       QVLMAFLRLGFVSVYLSSPMLDGFATGASCTILSVQAKYLLGLKIPRHQGYGTVVVTWIN  265
                 QVLMAFLRLGFVSVYLSSPMLDGFATGASCTILSVQAKYLLGLKIPRHQGYGTVVVTWIN
Sbjct  20326616  QVLMAFLRLGFVSVYLSSPMLDGFATGASCTILSVQAKYLLGLKIPRHQGYGTVVVTWIN  20326437

Query  266       IFKNIHKTNFCDLITSAICIIVLLAGKEIQDRYKKILKIPLPTELVVVAVATIVSHFADL  325
                 IFKNIHKTNFCDLITSAICIIVLLAGKEIQDRYKKILKIPLPTELVVVAVATIVSHFADL
Sbjct  20326436  IFKNIHKTNFCDLITSAICIIVLLAGKEIQDRYKKILKIPLPTELVVVAVATIVSHFADL  20326257

Query  326       NGQFSSSISGAIPTGFIPPKMPSIELMPRIAWDAIPLAVISFAFTVSLSEMFAKKHGYTV  385
                 NGQFSSSISGAIPTGFIPPKMPSIELMPRIAWDAIPLAVISFAFTVSLSEMFAKKHGYTV
Sbjct  20326256  NGQFSSSISGAIPTGFIPPKMPSIELMPRIAWDAIPLAVISFAFTVSLSEMFAKKHGYTV  20326077

Query  386       RPNQEMIAIGFCNIIPSFFHSFTTSAALAKTMVKDSAGCQTQVSSIVSALVVLLVLLFFA  445
                 RPNQEMIAIGFCNIIPSFFHSFTTSAALAKTMVKDSAGCQTQVSSIVSALVVLLVLLFFA
Sbjct  20326076  RPNQEMIAIGFCNIIPSFFHSFTTSAALAKTMVKDSAGCQTQVSSIVSALVVLLVLLFFA  20325897

Query  446       PFFYALQKCVLACIIIVSLRGALRKFRDVPKQWRQSKIEAIVWLVTMSSTALISVELGLV  505
                 PFFYALQKCVLACIIIVSLRGALRKFRDVPKQWRQSKIEAIVWLVTMSSTALISVELGLV
Sbjct  20325896  PFFYALQKCVLACIIIVSLRGALRKFRDVPKQWRQSKIEAIVWLVTMSSTALISVELGLV  20325717

Query  506       IGVIFSMICVVVQTQNPKVSLLGQIEQTNDYEDMEEYDNLSPVPNVKIFRFQAPLFYANK  565
                 IGVIFSMICVVVQTQNPKVSLLGQIEQTNDYEDMEEYDNLSPVPNVKIFRFQAPLFYANK
Sbjct  20325716  IGVIFSMICVVVQTQNPKVSLLGQIEQTNDYEDMEEYDNLSPVPNVKIFRFQAPLFYANK  20325537

Query  566       DFFLKSLYKATKLEPFLELTRRRKLEKKARAKGQKMTDGVDQTNGDVGICLISKDVGFHT  625
                 DFFLKSLYKATKLEPFLELTRRRKLEKKARAKGQKMTDGVDQTNGDVGICLISKDVGFHT
Sbjct  20325536  DFFLKSLYKATKLEPFLELTRRRKLEKKARAKGQKMTDGVDQTNGDVGICLISKDVGFHT  20325357

Query  626       IILDCSCISMIDTTAVSTFKTVSKDYKEVGVNVILACCNTTVIDSLRKGSFFGADDIDMD  685
                 IILDCSCISMIDTTAVSTFKTVSKDYKEVGVNVILACCNTTVIDSLRKGSFFGADDIDMD
Sbjct  20325356  IILDCSCISMIDTTAVSTFKTVSKDYKEVGVNVILACCNTTVIDSLRKGSFFGADDIDMD  20325177

Query  686       RLLFHTIHSAVCFANSTTQPVNDSSV  711
                 RLLFHTIHSAVCFANSTTQPVNDSSV
Sbjct  20325176  RLLFHTIHSAVCFANSTTQPVNDSSV  20325099
```

Range 2: 20327738 to 20328358

Score:772 bits(1576), Expect:0.0,
Method:,
Identities:207/207(100%), Positives:207/207(100%), Gaps:0/207(0%)

```
Query  1         MGASSLEAMEEDLNCVAVGLLKRRVRQKRGVREIIKTKIQRNLSCTGPKIKSTLTGFFPV  60
                 MGASSLEAMEEDLNCVAVGLLKRRVRQKRGVREIIKTKIQRNLSCTGPKIKSTLTGFFPV
Sbjct  20328358  MGASSLEAMEEDLNCVAVGLLKRRVRQKRGVREIIKTKIQRNLSCTGPKIKSTLTGFFPV  20328179

Query  61        VKWLPKYKVKEYIWGDVMSGLIIGIILIPQAIAYCLLAGLEPIYGLYTSFFSNIIYFFMG  120
                 VKWLPKYKVKEYIWGDVMSGLIIGIILIPQAIAYCLLAGLEPIYGLYTSFFSNIIYFFMG
Sbjct  20328178  VKWLPKYKVKEYIWGDVMSGLIIGIILIPQAIAYCLLAGLEPIYGLYTSFFSNIIYFFMG  20327999

Query  121       TSRHVSVGIFSLMSLMVGQVVDREVFLAGFDLNEDSTKNAFGFNDTGETNITAVNLKIMS  180
                 TSRHVSVGIFSLMSLMVGQVVDREVFLAGFDLNEDSTKNAFGFNDTGETNITAVNLKIMS
Sbjct  20327998  TSRHVSVGIFSLMSLMVGQVVDREVFLAGFDLNEDSTKNAFGFNDTGETNITAVNLKIMS  20327819

Query  181       LNMECGKECYAISIATALTFLAGVYQV  207
                 LNMECGKECYAISIATALTFLAGVYQV
Sbjct  20327818  LNMECGKECYAISIATALTFLAGVYQV  20327738
```

X Northern pike *slc26a1*

Query: sulfate anion transporter 1 [Esox lucius] Query ID: XP_010875588.2 Length: 702

>Esox lucius isolate fEsoLuc1 chromosome 14, fEsoLuc1.pri, whole genome shotgun sequence
Sequence ID: NC_047582.1 Length: 37473588
Range 1: 10416122 to 10417648

Score:1786 bits(3650), Expect:0.0,
Method:,
Identities:508/509(99%), Positives:509/509(100%), Gaps:0/509(0%)

```
Query  194       YQVLMAVFRLGFISVYLSAPMLDGFATGASFTILTVQAKYLLGLKIPRHQGYGTVVVTWI  253
                 +QVLMAVFRLGFISVYLSAPMLDGFATGASFTILTVQAKYLLGLKIPRHQGYGTVVVTWI
Sbjct  10417648  FQVLMAVFRLGFISVYLSAPMLDGFATGASFTILTVQAKYLLGLKIPRHQGYGTVVVTWI  10417469

Query  254       SIFSNIQNTNYCDLITSAICIFTLVVGKEFQERYKDRLKIPLPTELLVVAVATLASHFGD  313
                 SIFSNIQNTNYCDLITSAICIFTLVVGKEFQERYKDRLKIPLPTELLVVAVATLASHFGD
Sbjct  10417468  SIFSNIQNTNYCDLITSAICIFTLVVGKEFQERYKDRLKIPLPTELLVVAVATLASHFGD  10417289

Query  314       FHGQYDSSVSGAIPTGFIPPKVPSFGLMPRVAFDAIPLSVISFAFTVSLSEMFAKKNGYT  373
                 FHGQYDSSVSGAIPTGFIPPKVPSFGLMPRVAFDAIPLSVISFAFTVSLSEMFAKKNGYT
Sbjct  10417288  FHGQYDSSVSGAIPTGFIPPKVPSFGLMPRVAFDAIPLSVISFAFTVSLSEMFAKKNGYT  10417109

Query  374       VRPNQEMIAIGFCNIIPSFFHCFTTSAALAKTMVKDSTGCQTQVSSIVSAfvvllvllff  433
                 VRPNQEMIAIGFCNIIPSFFHCFTTSAALAKTMVKDSTGCQTQVSSIVSAFVVLLVLLFF
Sbjct  10417108  VRPNQEMIAIGFCNIIPSFFHCFTTSAALAKTMVKDSTGCQTQVSSIVSAFVVLLVLLFF  10416929

Query  434       APYFYSLQKCVLACIIIVSLRGALRKFRDVPSKWQVSKMDAIVWMVTMSASALISVEMGL  493
                 APYFYSLQKCVLACIIIVSLRGALRKFRDVPSKWQVSKMDAIVWMVTMSASALISVEMGL
Sbjct  10416928  APYFYSLQKCVLACIIIVSLRGALRKFRDVPSKWQVSKMDAIVWMVTMSASALISVEMGL  10416749

Query  494       VVGVVFSMLCVIIQTQKPKVSLLGQIHDTAHYEDMEEYENLMSLPKVKIFRFQAPLYYAN  553
                 VVGVVFSMLCVIIQTQKPKVSLLGQIHDTAHYEDMEEYENLMSLPKVKIFRFQAPLYYAN
Sbjct  10416748  VVGVVFSMLCVIIQTQKPKVSLLGQIHDTAHYEDMEEYENLMSLPKVKIFRFQAPLYYAN  10416569

Query  554       KDFFLKSLYKAVGVEPFQEMTRRRkaekkanklaakeaGRSDKTNGEVKVGLGSRELDFH  613
                 KDFFLKSLYKAVGVEPFQEMTRRRKAEKKANKLAAKEAGRSDKTNGEVKVGLGSRELDFH
Sbjct  10416568  KDFFLKSLYKAVGVEPFQEMTRRRKAEKKANKLAAKEAGRSDKTNGEVKVGLGSRELDFH  10416389

Query  614       TIILDCSAMPFIDSTGMQTFKGIIKDYKEVGVKVLLASCNTTVVDSLRQGSFFGKADKDM  673
                 TIILDCSAMPFIDSTGMQTFKGIIKDYKEVGVKVLLASCNTTVVDSLRQGSFFGKADKDM
Sbjct  10416388  TIILDCSAMPFIDSTGMQTFKGIIKDYKEVGVKVLLASCNTTVVDSLRQGSFFGKADKDM  10416209

Query  674       ESLSFYTVHAAVQFANDRAKSTSIGESMM  702
                 ESLSFYTVHAAVQFANDRAKSTSIGESMM
Sbjct  10416208  ESLSFYTVHAAVQFANDRAKSTSIGESMM  10416122
```

Range 2: 10417823 to 10418410

Score:731 bits(1491), Expect:0.0,
Method:,
Identities:196/196(100%), Positives:196/196(100%), Gaps:0/196(0%)

```
Query  1         MADATVVNVPHLERRVRQRKDTMTVLKTKFSRSLSCSVPRVKNTLTGFFPVVLWLPKYKL  60
                 MADATVVNVPHLERRVRQRKDTMTVLKTKFSRSLSCSVPRVKNTLTGFFPVVLWLPKYKL
Sbjct  10418410  MADATVVNVPHLERRVRQRKDTMTVLKTKFSRSLSCSVPRVKNTLTGFFPVVLWLPKYKL  10418231

Query  61        NEYIWGDLMSGLIIGIILVPQAIAYCLLAGVDPLYGLYTSFFANIIYFFMGTSRHVSVGI  120
                 NEYIWGDLMSGLIIGIILVPQAIAYCLLAGVDPLYGLYTSFFANIIYFFMGTSRHVSVGI
Sbjct  10418230  NEYIWGDLMSGLIIGIILVPQAIAYCLLAGVDPLYGLYTSFFANIIYFFMGTSRHVSVGI  10418051

Query  121       FSLMSLMVGQVVDREVYLAGFDLDDTKAATAEKILNMTGHPAVNLTTGAFDIQCGKECYA  180
                 FSLMSLMVGQVVDREVYLAGFDLDDTKAATAEKILNMTGHPAVNLTTGAFDIQCGKECYA
Sbjct  10418050  FSLMSLMVGQVVDREVYLAGFDLDDTKAATAEKILNMTGHPAVNLTTGAFDIQCGKECYA  10417871

Query  181       ISIAAALTFLAGIYQV  196
                 ISIAAALTFLAGIYQV
Sbjct  10417870  ISIAAALTFLAGIYQV  10417823
```

Y Atlantic cod *slc26a1*

Query: sulfate anion transporter 1-like [Gadus morhua] Query ID: XP_030197236.1 Length: 708

>Gadus morhua chromosome 19, gadMor3.0, whole genome shotgun sequence
Sequence ID: NC_044066.1 Length: 22015597
Range 1: 1719476 to 1720060

Score:707 bits(1443), Expect:0.0,
Method:,
Identities:195/195(100%), Positives:195/195(100%), Gaps:0/195(0%)

```
Query  514      VSLLGRSEDSELYEDLDEYENLAAPAGVRVFRFQAPLYYANKDSFLRTLYETVGVDPFLE  573
                VSLLGRSEDSELYEDLDEYENLAAPAGVRVFRFQAPLYYANKDSFLRTLYETVGVDPFLE
```

```
Sbjct  1720060  VSLLGRSEDSELYEDLDEYENLAAPAGVRVFRFQAPLYYANKDSFLRTLYETVGVDPFLE  1719881

Query  574      MTSRRKRDKKATKRLAAKQAGEDSENRKNGKLCVGLVRRELEFHTIVLDCSAIPFIDSAG  633
                MTSRRKRDKKATKRLAAKQAGEDSENRKNGKLCVGLVRRELEFHTIVLDCSAIPFIDSAG
Sbjct  1719880  MTSRRKRDKKATKRLAAKQAGEDSENRKNGKLCVGLVRRELEFHTIVLDCSAIPFIDSAG  1719701

Query  634      MGAFAGLVKDYGELGVAVLLAGCNTPVIDALRRGAFFGEKDRDMSLRLFYTVHAAVLSAR  693
                MGAFAGLVKDYGELGVAVLLAGCNTPVIDALRRGAFFGEKDRDMSLRLFYTVHAAVLSAR
Sbjct  1719700  MGAFAGLVKDYGELGVAVLLAGCNTPVIDALRRGAFFGEKDRDMSLRLFYTVHAAVLSAR  1719521

Query  694      SRAAAEGHTAGDSQV  708
                SRAAAEGHTAGDSQV
Sbjct  1719520  SRAAAEGHTAGDSQV  1719476
```

Range 2: 1728886 to 1729290

Score:506 bits(1031), Expect:1e-146,
Method:,
Identities:134/135(99%), Positives:135/135(100%), Gaps:0/135(0%)

```
Query  1        MEPQDLPLTDPPQLLERRVRKAQPPLSALRSRVKEEVACSMPRARSTLLGFFPVVSWLPK  60
                MEPQDLPLTDPPQLLERRVRKAQPPLSALRSRVKEEVACSMPRARSTLLGFFPVVSWLPK
Sbjct  1729290  MEPQDLPLTDPPQLLERRVRKAQPPLSALRSRVKEEVACSMPRARSTLLGFFPVVSWLPK  1729111

Query  61       YKLQEYVWGDLMSGVIVGIILVPQAIAYCLLAGVQPIYGLYTSFFANIIYFFMGTSRHVS  120
                YKLQEYVWGDLMSGVIVGIILVPQAIAYCLLAGVQPIYGLYTSFFANIIYFFMGTSRHVS
Sbjct  1729110  YKLQEYVWGDLMSGVIVGIILVPQAIAYCLLAGVQPIYGLYTSFFANIIYFFMGTSRHVS  1728931

Query  121      VGIFSLMSLMVGQVV  135
                VGIFSLMSLMVGQV+
Sbjct  1728930  VGIFSLMSLMVGQVI  1728886
```

Range 3: 1720491 to 1720790

Score:363 bits(739), Expect:2e-103,
Method:,
Identities:99/100(99%), Positives:99/100(99%), Gaps:0/100(0%)

```
Query  417      QVSSLISALVVLLVLLFFAPYFYSLQKCVLACIIIVSLRGALRKFRDVPAKWRVSRADAV  476
                QVSSLISALVVLLVLLFFAPYFYSLQKCVLACIIIVSLRGALRKFRDVPAKWRVSRADAV
Sbjct  1720790  QVSSLISALVVLLVLLFFAPYFYSLQKCVLACIIIVSLRGALRKFRDVPAKWRVSRADAV  1720611

Query  477      VWLVAMAATVLISVEIGLVVGVVFSMVCVIFVTQNPKVSL  516
                VWLVAMAATVLISVEIGLVVGVVFSMVCVIFVTQNPKV L
Sbjct  1720610  VWLVAMAATVLISVEIGLVVGVVFSMVCVIFVTQNPKVGL  1720491
```

Range 4: 1724040 to 1724288

Score:307 bits(626), Expect:7e-87,
Method:,
Identities:83/83(100%), Positives:83/83(100%), Gaps:0/83(0%)

```
Query  196      QVLMAVFRLGFVSVYLSAPMLDGFATGASLTILTVQAKYLLGLKIPRHQGYGTVVVTWIN  255
                QVLMAVFRLGFVSVYLSAPMLDGFATGASLTILTVQAKYLLGLKIPRHQGYGTVVVTWIN
Sbjct  1724288  QVLMAVFRLGFVSVYLSAPMLDGFATGASLTILTVQAKYLLGLKIPRHQGYGTVVVTWIN  1724109

Query  256      IFANIQTNTLCDLVTSAICIFVL  278
                IFANIQTNTLCDLVTSAICIFVL
Sbjct  1724108  IFANIQTNTLCDLVTSAICIFVL  1724040
```

Range 5: 1723493 to 1723726

Score:282 bits(573), Expect:5e-79,
Method:,
Identities:78/78(100%), Positives:78/78(100%), Gaps:0/78(0%)

```
Query  278      LVAGKEIQDRYKSRLKIPLPTELVVVAGATLASHFGNLNALYGSSVSGHIPTGFIPPTVP  337
                LVAGKEIQDRYKSRLKIPLPTELVVVAGATLASHFGNLNALYGSSVSGHIPTGFIPPTVP
Sbjct  1723726  LVAGKEIQDRYKSRLKIPLPTELVVVAGATLASHFGNLNALYGSSVSGHIPTGFIPPTVP  1723547

Query  338      ALDLMHRVALDAVPMAVI  355
                ALDLMHRVALDAVPMAVI
Sbjct  1723546  ALDLMHRVALDAVPMAVI  1723493
```

Range 6: 1723043 to 1723231

Score:237 bits(482), Expect:1e-65,
Method:,
Identities:63/63(100%), Positives:63/63(100%), Gaps:0/63(0%)

```
Query  356      SFAFTVSLSEMFAKKNGYTVRPNQEMLAIGCCNIIPSFFHCFTTSAALAKTMVKDSTGCQ  415
                SFAFTVSLSEMFAKKNGYTVRPNQEMLAIGCCNIIPSFFHCFTTSAALAKTMVKDSTGCQ
Sbjct  1723231  SFAFTVSLSEMFAKKNGYTVRPNQEMLAIGCCNIIPSFFHCFTTSAALAKTMVKDSTGCQ  1723052
```

```
Query  416      TQV  418
                TQV
Sbjct  1723051  TQV  1723043
```

Range 7: 1728338 to 1728535

Score:233 bits(474), Expect:2e-64,
Method:,
Identities:65/66(98%), Positives:66/66(100%), Gaps:0/66(0%)

```
Query  133      QVVDREVFQAGFDLSDTDPGSFNGTGVASNLSAATVEVMGVVYSKECYAIAIAAALTLLA  192
                QVVDREVFQAGFDLSDTDPGSFNGTGVASNLSAATVEVMGVVYSKECYAIAIAAALTLLA
Sbjct  1728535  QVVDREVFQAGFDLSDTDPGSFNGTGVASNLSAATVEVMGVVYSKECYAIAIAAALTLLA  1728356

Query  193      GIYQVL  198
                GIYQV+
Sbjct  1728355  GIYQVM  1728338
```

Z Nile tilapia *slc26a1*

Query: sulfate anion transporter 1 isoform X3 [Oreochromis niloticus] Query ID: XP_005451462.1 Length: 715

>Oreochromis niloticus isolate F11D_XX linkage group LG7, O_niloticus_UMD_NMBU, whole genome shotgun sequence
Sequence ID: NC_031972.2 Length: 64772279
Range 1: 26635421 to 26635999

Score:700 bits(1429), Expect:0.0,
Method:,
Identities:193/193(100%), Positives:193/193(100%), Gaps:0/193(0%)

```
Query  523       VSLLGRVDDTDFYEDLEEYKNLVPPSRVQIFRFQAPLYYANKESFLKSLYKAVGVEPFLE  582
                 VSLLGRVDDTDFYEDLEEYKNLVPPSRVQIFRFQAPLYYANKESFLKSLYKAVGVEPFLE
Sbjct  26635421  VSLLGRVDDTDFYEDLEEYKNLVPPSRVQIFRFQAPLYYANKESFLKSLYKAVGVEPFLE  26635600

Query  583       LTKRKKAEKKAKDASVKQAKANGEKNNGDVVVRLVQRELEFHTIVLDCSAIPFIDSAGLA  642
                 LTKRKKAEKKAKDASVKQAKANGEKNNGDVVVRLVQRELEFHTIVLDCSAIPFIDSAGLA
Sbjct  26635601  LTKRKKAEKKAKDASVKQAKANGEKNNGDVVVRLVQRELEFHTIVLDCSAIPFIDSAGLA  26635780

Query  643       AFLELVKEYKGIGVSVLLACCNTSLIDTLQKGKFFGKNDEDMGSLLFHTVHAAVVHANRA  702
                 AFLELVKEYKGIGVSVLLACCNTSLIDTLQKGKFFGKNDEDMGSLLFHTVHAAVVHANRA
Sbjct  26635781  AFLELVKEYKGIGVSVLLACCNTSLIDTLQKGKFFGKNDEDMGSLLFHTVHAAVVHANRA  26635960

Query  703       AAATDSRSDDSEV  715
                 AAATDSRSDDSEV
Sbjct  26635961  AAATDSRSDDSEV  26635999
```

Range 2: 26631000 to 26631416

Score:517 bits(1055), Expect:0.0,
Method:,
Identities:137/139(99%), Positives:139/139(100%), Gaps:0/139(0%)

```
Query  1         MEEAANTTETPPAVPVLLERQVRQRKPAVSVLKSKLKQAVTCSVPRIRSTLSGFFPVMRW  60
                 MEEAANTTETPPAVPVLLERQVRQRKPAVSVLKSKLKQAVTCSVPRIRSTLSGFFPVMRW
Sbjct  26631000  MEEAANTTETPPAVPVLLERQVRQRKPAVSVLKSKLKQAVTCSVPRIRSTLSGFFPVMRW  26631179

Query  61        LPKYNIKEYVWGDVMSGMIVGIILVPQAIAYCLLAGVEPIYGLYTSFYANIIYFLMGTSR  120
                 LPKYNIKEYVWGDVMSGMIVGIILVPQAIAYCLLAGVEPIYGLYTSFYANIIYFLMGTSR
Sbjct  26631180  LPKYNIKEYVWGDVMSGMIVGIILVPQAIAYCLLAGVEPIYGLYTSFYANIIYFLMGTSR  26631359

Query  121       HVSVGIFSLMSLMIGQVVD  139
                 HVSVGIFSLMSLMIGQV++
Sbjct  26631360  HVSVGIFSLMSLMIGQVIN  26631416
```

Range 3: 26635031 to 26635327

Score:365 bits(743), Expect:0.0,
Method:,
Identities:99/99(100%), Positives:99/99(100%), Gaps:0/99(0%)

```
Query  426       QVSSLISALVVLLVLLFFAPFFQALQKCVLACIIIVSLRGALRKFRDVPAKWRASRNDAI  485
                 QVSSLISALVVLLVLLFFAPFFQALQKCVLACIIIVSLRGALRKFRDVPAKWRASRNDAI
Sbjct  26635031  QVSSLISALVVLLVLLFFAPFFQALQKCVLACIIIVSLRGALRKFRDVPAKWRASRNDAI  26635210

Query  486       VWLVAMSATALISVELGLLVGIIFSMICFIFRTQNPKVS  524
                 VWLVAMSATALISVELGLLVGIIFSMICFIFRTQNPKVS
Sbjct  26635211  VWLVAMSATALISVELGLLVGIIFSMICFIFRTQNPKVS  26635327
```

```
Range 4: 26631492 to 26631704

Score:262 bits(532), Expect:0.0,
Method:,
Identities:71/71(100%), Positives:71/71(100%), Gaps:0/71(0%)

Query  136       QVVDREVFLAGFDLNEDSAVSNIDVLNDTLGINLTVTKVHTVELMGMQCGKECYAISIAV  195
                 QVVDREVFLAGFDLNEDSAVSNIDVLNDTLGINLTVTKVHTVELMGMQCGKECYAISIAV
Sbjct  26631492  QVVDREVFLAGFDLNEDSAVSNIDVLNDTLGINLTVTKVHTVELMGMQCGKECYAISIAV  26631671

Query  196       AVTFLAGVYQV  206
                 AVTFLAGVYQV
Sbjct  26631672  AVTFLAGVYQV  26631704



Range 5: 26633453 to 26633701

Score:307 bits(626), Expect:6e-165,
Method:,
Identities:83/83(100%), Positives:83/83(100%), Gaps:0/83(0%)

Query  205       QVLMAVFRLGFVSVYLSAPMLDGFATGASFTILTVQAKYLLGLKIPRHQGYGTVVVTWVN  264
                 QVLMAVFRLGFVSVYLSAPMLDGFATGASFTILTVQAKYLLGLKIPRHQGYGTVVVTWVN
Sbjct  26633453  QVLMAVFRLGFVSVYLSAPMLDGFATGASFTILTVQAKYLLGLKIPRHQGYGTVVVTWVN  26633632

Query  265       IFSNIHKTNLCDLITSAICISIL  287
                 IFSNIHKTNLCDLITSAICISIL
Sbjct  26633633  IFSNIHKTNLCDLITSAICISIL  26633701



Range 6: 26633789 to 26634031

Score:282 bits(573), Expect:6e-165,
Method:,
Identities:79/81(98%), Positives:79/81(97%), Gaps:0/81(0%)

Query  284       ISILVAAKEIQERYKDRLKMPLPTELVVVAGATLASHFGELNSRYGSSVSGHIPTGFMPP  343
                 I I VAAKEIQERYKDRLKMPLPTELVVVAGATLASHFGELNSRYGSSVSGHIPTGFMPP
Sbjct  26633789  IFISVAAKEIQERYKDRLKMPLPTELVVVAGATLASHFGELNSRYGSSVSGHIPTGFMPP  26633968

Query  344       QLPGFSLMSRVVLDAIPLAVI  364
                 QLPGFSLMSRVVLDAIPLAVI
Sbjct  26633969  QLPGFSLMSRVVLDAIPLAVI  26634031



Range 7: 26634370 to 26634558

Score:235 bits(477), Expect:3e-65,
Method:,
Identities:63/63(100%), Positives:63/63(100%), Gaps:0/63(0%)

Query  365       SFAFTVSLSEMFAKKHGYTVRPNQEMLAIGFCNIIPSFFHSFTTSAALAKTMVKDSTGCK  424
                 SFAFTVSLSEMFAKKHGYTVRPNQEMLAIGFCNIIPSFFHSFTTSAALAKTMVKDSTGCK
Sbjct  26634370  SFAFTVSLSEMFAKKHGYTVRPNQEMLAIGFCNIIPSFFHSFTTSAALAKTMVKDSTGCK  26634549

Query  425       TQV  427
                 TQV
Sbjct  26634550  TQV  26634558
```

AA Tarpon *slc26a1*

```
Query: UNNAMED PROTEIN PRODUCT ID: lcl|Query_164879(amino acid) Length: 674

>Megalops atlanticus isolate YG-15Mar2019-1 chromosome 6, whole genome shotgun sequence
Sequence ID: JAFDVH010000006.1 Length: 42596872
Range 1: 13983351 to 13984865

Score:853 bits(2205), Expect:0.0,
Method:Compositional matrix adjust.,
Identities:473/505(94%), Positives:473/505(93%), Gaps:32/505(6%)

Query  202       VLMAVLQLGFVSVYLSAPMLDGFATGASLTILTVQAKYLLGLKIPRHQ------------  249
                 VLMAVLQLGFVSVYLSAPMLDGFATGASLTILTVQAKYLLGLKIPRHQ
Sbjct  13983351  VLMAVLQLGFVSVYLSAPMLDGFATGASLTILTVQAKYLLGLKIPRHQGYGTVVVTWVNI  13983530

Query  250       --------------------VLVAGKELQDRYKNRLKIPLPTELvvvaaaTLVSHFADLH  289
                                     VLVAGKELQDRYKNRLKIPLPTELVVVAAATLVSHFADLH
Sbjct  13983531  FKNIHKTNFCDMITSALCISVLVAGKELQDRYKNRLKIPLPTELVVVAAATLVSHFADLH  13983710

Query  290       GRYSSSISGAIPTGFIPPKVPSFDLMTKVAFDAIPLAVISFAFTVSLSEMFAKKHGYTVR  349
                 GRYSSSISGAIPTGFIPPKVPSFDLMTKVAFDAIPLAVISFAFTVSLSEMFAKKHGYTVR
Sbjct  13983711  GRYSSSISGAIPTGFIPPKVPSFDLMTKVAFDAIPLAVISFAFTVSLSEMFAKKHGYTVR  13983890

Query  350       ANQEMIAIGFCNIIPSFFHCFTTSAALAKTMVKDSTGCQTQvsslvsafvvllvllvfaP  409
                 ANQEMIAIGFCNIIPSFFHCFTTSAALAKTMVKDSTGCQTQVSSLVSAFVVLLVLLVFAP
Sbjct  13983891  ANQEMIAIGFCNIIPSFFHCFTTSAALAKTMVKDSTGCQTQVSSLVSAFVVLLVLLVFAP  13984070
```

```
Query  410       FFYSLQKCVLACIIIVSLRGALRKFRDVPRQWRINKVDAIVWMVTMSASALVSVEIGLLI  469
                 FFYSLQKCVLACIIIVSLRGALRKFRDVPRQWRINKVDAIVWMVTMSASALVSVEIGLLI
Sbjct  13984071  FFYSLQKCVLACIIIVSLRGALRKFRDVPRQWRINKVDAIVWMVTMSASALVSVEIGLLI  13984250

Query  470       GVVFSMLCVVARTQNPKVALLGQIQHTSHYEDIREYDNLLAVPKVKIFRFQAPLYYANKD  529
                 GVVFSMLCVVARTQNPKVALLGQIQHTSHYEDIREYDNLLAVPKVKIFRFQAPLYYANKD
Sbjct  13984251  GVVFSMLCVVARTQNPKVALLGQIQHTSHYEDIREYDNLLAVPKVKIFRFQAPLYYANKD  13984430

Query  530       NFLKSLYKAVGLEPFIEVTkrkkaekkakelaakqakGSDSTNGDANLGLVPRDLDFHTI  589
                 NFLKSLYKAVGLEPFIEVTKRKKAEKKAKELAAKQAKGSDSTNGDANLGLVPRDLDFHTI
Sbjct  13984431  NFLKSLYKAVGLEPFIEVTKRKKAEKKAKELAAKQAKGSDSTNGDANLGLVPRDLDFHTI  13984610

Query  590       ILDCSAISFIDTTGMLTFKMVIKDYKDVGVRVLLACCNPPVIDALKRGSVFGPDDKEMES  649
                 ILDCSAISFIDTTGMLTFKMVIKDYKDVGVRVLLACCNPPVIDALKRGSVFGPDDKEMES
Sbjct  13984611  ILDCSAISFIDTTGMLTFKMVIKDYKDVGVRVLLACCNPPVIDALKRGSVFGPDDKEMES  13984790

Query  650       MSFHSVHGATLFAQDSAATVGDLAV  674
                 MSFHSVHGATLFAQDSAATVGDLAV
Sbjct  13984791  MSFHSVHGATLFAQDSAATVGDLAV  13984865
```

Range 2: 13981457 to 13982065

Score:402 bits(1032), Expect:6e-121,
Method:Compositional matrix adjust.,
Identities:203/203(100%), Positives:203/203(100%), Gaps:0/203(0%)

```
Query  1         MEGCKLTETCHLErrvrrrrEAKEIILTKLSHSFSCSVTKVKSTLTDFFPVVRWLPKYKL  60
                 MEGCKLTETCHLERRVRRRREAKEIILTKLSHSFSCSVTKVKSTLTDFFPVVRWLPKYKL
Sbjct  13981457  MEGCKLTETCHLERRVRRRREAKEIILTKLSHSFSCSVTKVKSTLTDFFPVVRWLPKYKL  13981636

Query  61        KEYVWGDVMSGLIVGIILVPQAIAYCLLAGLEPIYGLYTSFFANIIYFFMGTSKHVSVGI  120
                 KEYVWGDVMSGLIVGIILVPQAIAYCLLAGLEPIYGLYTSFFANIIYFFMGTSKHVSVGI
Sbjct  13981637  KEYVWGDVMSGLIVGIILVPQAIAYCLLAGLEPIYGLYTSFFANIIYFFMGTSKHVSVGI  13981816

Query  121       FSLMSLMVGQVVDREVYLAGFDLNEDSKPATPGLDGIFNGTGVSSPSTVNLTLGAYNIEC  180
                 FSLMSLMVGQVVDREVYLAGFDLNEDSKPATPGLDGIFNGTGVSSPSTVNLTLGAYNIEC
Sbjct  13981817  FSLMSLMVGQVVDREVYLAGFDLNEDSKPATPGLDGIFNGTGVSSPSTVNLTLGAYNIEC  13981996

Query  181       GKECYAISIAAALTFLAGVYQVL  203
                 GKECYAISIAAALTFLAGVYQVL
Sbjct  13981997  GKECYAISIAAALTFLAGVYQVL  13982065
```

AB West African bonefish *slc26a1*

Query: hypothetical protein AGOR_G00245810 [Albula goreensis] Query ID: KAI1881981.1 Length: 705

>Albula goreensis voucher SM-30Jul2019_1 chromosome 25, whole genome shotgun sequence
Sequence ID: JAERUA010000025.1 Length: 23709570
Range 1: 3018099 to 3019616

Score:1859 bits(3799), Expect:0.0,
Method:,
Identities:506/506(100%), Positives:506/506(100%), Gaps:0/506(0%)

```
Query  200      QILMAVFRLGFVSVYLSAPMLDGFATGASLTILTVQAKYLLGLKIPRHQGAGTVVVTWIN  259
                QILMAVFRLGFVSVYLSAPMLDGFATGASLTILTVQAKYLLGLKIPRHQGAGTVVVTWIN
Sbjct  3018099  QILMAVFRLGFVSVYLSAPMLDGFATGASLTILTVQAKYLLGLKIPRHQGAGTVVVTWIN  3018278

Query  260      IFKNIQNTNFCDMITSAICIAVLVAGKELQDRYKNRLKIPLPTELVVVAVATVVSHFADL  319
                IFKNIQNTNFCDMITSAICIAVLVAGKELQDRYKNRLKIPLPTELVVVAVATVVSHFADL
Sbjct  3018279  IFKNIQNTNFCDMITSAICIAVLVAGKELQDRYKNRLKIPLPTELVVVAVATVVSHFADL  3018458

Query  320      NGKYSSSISGAIPTGFIPPKVPSFDLMPRVAFDAIPLAVISFAFTVSLSEMFAKKHGYTV  379
                NGKYSSSISGAIPTGFIPPKVPSFDLMPRVAFDAIPLAVISFAFTVSLSEMFAKKHGYTV
Sbjct  3018459  NGKYSSSISGAIPTGFIPPKVPSFDLMPRVAFDAIPLAVISFAFTVSLSEMFAKKHGYTV  3018638

Query  380      RANQEMMAIGFCNIIPSFFHCFTTSAALAKTMVKDSTGCQTQVSSVVSAFVVLLVLLFFA  439
                RANQEMMAIGFCNIIPSFFHCFTTSAALAKTMVKDSTGCQTQVSSVVSAFVVLLVLLFFA
Sbjct  3018639  RANQEMMAIGFCNIIPSFFHCFTTSAALAKTMVKDSTGCQTQVSSVVSAFVVLLVLLFFA  3018818

Query  440      PFFYSLQKCVLASIIIVSLRGALRKFRDVPNQWRTNKLDAIVWMVAMSASALISVEIGLV  499
                PFFYSLQKCVLASIIIVSLRGALRKFRDVPNQWRTNKLDAIVWMVAMSASALISVEIGLV
Sbjct  3018819  PFFYSLQKCVLASIIIVSLRGALRKFRDVPNQWRTNKLDAIVWMVAMSASALISVEIGLV  3018998

Query  500      VGVVFSMLCVVAQTQNPKVALLGQIQNTSHYEDLGEYNNLMAVPKVKIFRFQAPLYYANK  559
                VGVVFSMLCVVAQTQNPKVALLGQIQNTSHYEDLGEYNNLMAVPKVKIFRFQAPLYYANK
Sbjct  3018999  VGVVFSMLCVVAQTQNPKVALLGQIQNTSHYEDLGEYNNLMAVPKVKIFRFQAPLYYANK  3019178

Query  560      ESFLKSLYKAVSLEPFLEIAKRKKLEKKAKELAAKQAKSSGSTNGDVAVNLVSRDYDFHT  619
                ESFLKSLYKAVSLEPFLEIAKRKKLEKKAKELAAKQAKSSGSTNGDVAVNLVSRDYDFHT
Sbjct  3019179  ESFLKSLYKAVSLEPFLEIAKRKKLEKKAKELAAKQAKSSGSTNGDVAVNLVSRDYDFHT  3019358

Query  620      IILDCSSMPFIDTTGMLTLKTVMKDYKEIGVRILLACCNPPVIDALKCGSVFGPDDKDVG  679
                IILDCSSMPFIDTTGMLTLKTVMKDYKEIGVRILLACCNPPVIDALKCGSVFGPDDKDVG
Sbjct  3019359  IILDCSSMPFIDTTGMLTLKTVMKDYKEIGVRILLACCNPPVIDALKCGSVFGPDDKDVG  3019538
```

Query 680   SLTFHSVHGAVLFAGDCASTVGDMGV 705
SLTFHSVHGAVLFAGDCASTVGDMGV
Sbjct 3019539 SLTFHSVHGAVLFAGDCASTVGDMGV 3019616

Range 2: 3017305 to 3017907

Score:745 bits(1521), Expect:0.0,
Method:,
Identities:200/201(99%), Positives:201/201(100%), Gaps:0/201(0%)

Query 1   MADCKLAEVGHLERKVRRRRRPKDVILSKLSRSCSCSTAKLKAALTGFFPVVRWLPKYRL 60
MADCKLAEVGHLERKVRRRRRPKDVILSKLSRSCSCSTAKLKAALTGFFPVVRWLPKYRL
Sbjct 3017305 MADCKLAEVGHLERKVRRRRRPKDVILSKLSRSCSCSTAKLKAALTGFFPVVRWLPKYRL 3017484

Query 61   REYAWGDIMSGLIVGIILVPQAIAYCLLAGLEPIYGLYTSFFANIIYFLMGTSKHVSVGI 120
REYAWGDIMSGLIVGIILVPQAIAYCLLAGLEPIYGLYTSFFANIIYFLMGTSKHVSVGI
Sbjct 3017485 REYAWGDIMSGLIVGIILVPQAIAYCLLAGLEPIYGLYTSFFANIIYFLMGTSKHVSVGI 3017664

Query 121   FSLMSLMVGQVVDREVYLAGFEFSEEGQPAAVTLNGIWNGTLHNGSVVNLTMGGYHLECG 180
FSLMSLMVGQVVDREVYLAGFEFSEEGQPAAVTLNGIWNGTLHNGSVVNLTMGGYHLECG
Sbjct 3017665 FSLMSLMVGQVVDREVYLAGFEFSEEGQPAAVTLNGIWNGTLHNGSVVNLTMGGYHLECG 3017844

Query 181   RECYAISIAAALTFLAGVYQI 201
RECYAISIAAALTFLAGVYQ+
Sbjct 3017845 RECYAISIAAALTFLAGVYQV 3017907

AC European eel *slc26a1*

Query: sulfate anion transporter 1 [Anguilla anguilla] Query ID: XP_035245576.1 Length: 702

>Anguilla anguilla isolate fAngAng1 chromosome 14, fAngAng1.pri, whole genome shotgun sequence
Sequence ID: NC_049214.1 Length: 41497830
Range 1: 35178488 to 35179990

Score:1849 bits(3778), Expect:0.0,
Method:,
Identities:501/501(100%), Positives:501/501(100%), Gaps:0/501(0%)

Query 202   VLMAVFRLGFVSVYLSAPMLDGFATGASLTILTVQAKYLLGLKIPRHQGYGTVVVTWINI 261
VLMAVFRLGFVSVYLSAPMLDGFATGASLTILTVQAKYLLGLKIPRHQGYGTVVVTWINI
Sbjct 35178488 VLMAVFRLGFVSVYLSAPMLDGFATGASLTILTVQAKYLLGLKIPRHQGYGTVVVTWINI 35178667

Query 262   FKNIHMTNFCDVITSAICITVLVAGKELQDRYKSRLKIPLPTELVVVAMATLVSHFADLG 321
FKNIHMTNFCDVITSAICITVLVAGKELQDRYKSRLKIPLPTELVVVAMATLVSHFADLG
Sbjct 35178668 FKNIHMTNFCDVITSAICITVLVAGKELQDRYKSRLKIPLPTELVVVAMATLVSHFADLG 35178847

Query 322   GRYSSSISGAIPTGFIQPEMPSFQLMPRVALDAIPLAIISFAFTVSLSEMFAKKHGYTVR 381
GRYSSSISGAIPTGFIQPEMPSFQLMPRVALDAIPLAIISFAFTVSLSEMFAKKHGYTVR
Sbjct 35178848 GRYSSSISGAIPTGFIQPEMPSFQLMPRVALDAIPLAIISFAFTVSLSEMFAKKHGYTVR 35179027

Query 382   ANQEMIAIGFCNIIPSFFHCFTTSAALAKTMVKDSTGCQTQVSSLVSAFVVLLVLLIFAP 441
ANQEMIAIGFCNIIPSFFHCFTTSAALAKTMVKDSTGCQTQVSSLVSAFVVLLVLLIFAP
Sbjct 35179028 ANQEMIAIGFCNIIPSFFHCFTTSAALAKTMVKDSTGCQTQVSSLVSAFVVLLVLLIFAP 35179207

Query 442   FFYSLQKCVLACIIIVSLRGALRKFRDVPEQWRLNNVDAIIWTVTMSASALISVEIGLLV 501
FFYSLQKCVLACIIIVSLRGALRKFRDVPEQWRLNNVDAIIWTVTMSASALISVEIGLLV
Sbjct 35179208 FFYSLQKCVLACIIIVSLRGALRKFRDVPEQWRLNNVDAIIWTVTMSASALISVEIGLLV 35179387

Query 502   GVVFSMLCVVVRTQNPKVALLGQIPDTSHYEDLGEYDNLLAVPKVKIFRFQAPLYYANKD 561
GVVFSMLCVVVRTQNPKVALLGQIPDTSHYEDLGEYDNLLAVPKVKIFRFQAPLYYANKD
Sbjct 35179388 GVVFSMLCVVVRTQNPKVALLGQIPDTSHYEDLGEYDNLLAVPKVKIFRFQAPLYYANKD 35179567

Query 562   SFLKSLYKAVGLEPFLEVAKRRKAEKRAKDPATKQAKSSDSINGNVATVLVTRDIHTIIL 621
SFLKSLYKAVGLEPFLEVAKRRKAEKRAKDPATKQAKSSDSINGNVATVLVTRDIHTIIL
Sbjct 35179568 SFLKSLYKAVGLEPFLEVAKRRKAEKRAKDPATKQAKSSDSINGNVATVLVTRDIHTIIL 35179747

Query 622   DCSAMPFIDTPGMLTLKLVMKDYKGIGVRVLLACCNPPVIDALKRGFVFGPGHKDMDSLT 681
DCSAMPFIDTPGMLTLKLVMKDYKGIGVRVLLACCNPPVIDALKRGFVFGPGHKDMDSLT
Sbjct 35179748 DCSAMPFIDTPGMLTLKLVMKDYKGIGVRVLLACCNPPVIDALKRGFVFGPGHKDMDSLT 35179927

Query 682   FHSVHGAVLYAYDNVDAEMAV 702
FHSVHGAVLYAYDNVDAEMAV
Sbjct 35179928 FHSVHGAVLYAYDNVDAEMAV 35179990

Range 2: 35177722 to 35178336

Score:753 bits(1536), Expect:0.0,
Method:,
Identities:204/205(99%), Positives:204/205(99%), Gaps:0/205(0%)

Query 1   MADRKAAEVEHLERRVRRRRGPKDVILSKLSRSFTCSTARLKVTLTGFFPVVKWLPKYKL 60
MADRKAAEVEHLERRVRRRRGPKDVILSKLSRSFTCSTARLKVTLTGFFPVVKWLPKYKL
Sbjct 35177722 MADRKAAEVEHLERRVRRRRGPKDVILSKLSRSFTCSTARLKVTLTGFFPVVKWLPKYKL 35177901

```
Query  61        KEYAWGDAMSGLIVGIILVPQAIAYCLLAGLEPIYGLYTSFFANIIYFFMGTSKHVSVGI  120
                 KEYAWGDAMSGLIVGIILVPQAIAYCLLAGLEPIYGLYTSFFANIIYFFMGTSKHVSVGI
Sbjct  35177902  KEYAWGDAMSGLIVGIILVPQAIAYCLLAGLEPIYGLYTSFFANIIYFFMGTSKHVSVGI  35178081

Query  121       FSLMSLMVGQVVDREVYLAGFDLSEEGKPAVTSLNDMWNGTVIFNTSAVNLTLGGYSLEC  180
                 FSLMSLMVGQVVDREVYLAGFDLSEEGKPAVTSLNDMWNGTVIFNTSAVNLTLGGYSLEC
Sbjct  35178082  FSLMSLMVGQVVDREVYLAGFDLSEEGKPAVTSLNDMWNGTVIFNTSAVNLTLGGYSLEC  35178261

Query  181       GKECYAISIAATLTFLAGVYQVLMA  205
                 GKECYAISIAATLTFLAGVYQVL A
Sbjct  35178262  GKECYAISIAATLTFLAGVYQVLRA  35178336
```

AD European conger *slc26a1*

Query: hypothetical protein COCON_G00166240 [Conger conger] Query ID: KAJ8260901.1 Length: 705

>Conger conger isolate Concon-B chromosome 12, whole genome shotgun sequence
Sequence ID: JAFJMO010000012.1 Length: 44312402
Range 1: 19494196 to 19495707

Score:1861 bits(3802), Expect:0.0,
Method:,
Identities:504/504(100%), Positives:504/504(100%), Gaps:0/504(0%)

```
Query  202       VLMAVFRLGFVSVYLSSPMLDGFATGASFTILTVQAKYLLGLKIPRHQGYGTVVVTWINI  261
                 VLMAVFRLGFVSVYLSSPMLDGFATGASFTILTVQAKYLLGLKIPRHQGYGTVVVTWINI
Sbjct  19494196  VLMAVFRLGFVSVYLSSPMLDGFATGASFTILTVQAKYLLGLKIPRHQGYGTVVVTWINI  19494375

Query  262       FKNIHKTNFCDMITSAICISVLVAGKELQERYKSRLKIPLPTELVVVAVATVVSHFADLS  321
                 FKNIHKTNFCDMITSAICISVLVAGKELQERYKSRLKIPLPTELVVVAVATVVSHFADLS
Sbjct  19494376  FKNIHKTNFCDMITSAICISVLVAGKELQERYKSRLKIPLPTELVVVAVATVVSHFADLS  19494555

Query  322       GRYSSSISGAIPTGFIPPKVPSFELMPRVALDAIPLAVISFAFTVSLSEMFAKKHGYSVR  381
                 GRYSSSISGAIPTGFIPPKVPSFELMPRVALDAIPLAVISFAFTVSLSEMFAKKHGYSVR
Sbjct  19494556  GRYSSSISGAIPTGFIPPKVPSFELMPRVALDAIPLAVISFAFTVSLSEMFAKKHGYSVR  19494735

Query  382       ANQEMIAIGFCNIIPSFFHCFTTSAALAKTMVKDSTGCQTQVSSLVSAFVILLVLLIFAP  441
                 ANQEMIAIGFCNIIPSFFHCFTTSAALAKTMVKDSTGCQTQVSSLVSAFVILLVLLIFAP
Sbjct  19494736  ANQEMIAIGFCNIIPSFFHCFTTSAALAKTMVKDSTGCQTQVSSLVSAFVILLVLLIFAP  19494915

Query  442       FFYSLQKCVLACIIIVSLRGALRKFRDVPELWRMNNVDAVIWMVTVSASALISVEIGLLV  501
                 FFYSLQKCVLACIIIVSLRGALRKFRDVPELWRMNNVDAVIWMVTVSASALISVEIGLLV
Sbjct  19494916  FFYSLQKCVLACIIIVSLRGALRKFRDVPELWRMNNVDAVIWMVTVSASALISVEIGLLV  19495095

Query  502       GVVFSMLCVVVRTQNPKVALLGQIQDTSHYEDLGEYDNLLVVPKVKILRFQAPLYYANKD  561
                 GVVFSMLCVVVRTQNPKVALLGQIQDTSHYEDLGEYDNLLVVPKVKILRFQAPLYYANKD
Sbjct  19495096  GVVFSMLCVVVRTQNPKVALLGQIQDTSHYEDLGEYDNLLVVPKVKILRFQAPLYYANKD  19495275

Query  562       FFLKSLYKAVGLEPLLEVAKRRKAEKQAKDQAKQAKSLDSINGNAATVLVFREPDFHTII  621
                 FFLKSLYKAVGLEPLLEVAKRRKAEKQAKDQAKQAKSLDSINGNAATVLVFREPDFHTII
Sbjct  19495276  FFLKSLYKAVGLEPLLEVAKRRKAEKQAKDQAKQAKSLDSINGNAATVLVFREPDFHTII  19495455

Query  622       LDCSSMPFIDTPGMMTLKMVMKDYKGVGVRVLLACCNPPVIDALKRGSVFGPGHKEMDSL  681
                 LDCSSMPFIDTPGMMTLKMVMKDYKGVGVRVLLACCNPPVIDALKRGSVFGPGHKEMDSL
Sbjct  19495456  LDCSSMPFIDTPGMMTLKMVMKDYKGVGVRVLLACCNPPVIDALKRGSVFGPGHKEMDSL  19495635

Query  682       TFHSVHGAILFAHDSEEAVCEMGV  705
                 TFHSVHGAILFAHDSEEAVCEMGV
Sbjct  19495636  TFHSVHGAILFAHDSEEAVCEMGV  19495707
```

Range 2: 19492927 to 19493541

Score:751 bits(1533), Expect:0.0,
Method:,
Identities:204/205(99%), Positives:204/205(99%), Gaps:0/205(0%)

```
Query  1         MADCRPAEVEHLERRVRRRRGPKDVIVSKLSRSLSCSTARLKRTLAGFFPVVKWLPKYRL  60
                 MADCRPAEVEHLERRVRRRRGPKDVIVSKLSRSLSCSTARLKRTLAGFFPVVKWLPKYRL
Sbjct  19492927  MADCRPAEVEHLERRVRRRRGPKDVIVSKLSRSLSCSTARLKRTLAGFFPVVKWLPKYRL  19493106

Query  61        KEYAWGDAMSGLIVGIILVPQAIAYCLLAGLEPIYGLYTSFFANIIYFLMGTSRHVSVGI  120
                 KEYAWGDAMSGLIVGIILVPQAIAYCLLAGLEPIYGLYTSFFANIIYFLMGTSRHVSVGI
Sbjct  19493107  KEYAWGDAMSGLIVGIILVPQAIAYCLLAGLEPIYGLYTSFFANIIYFLMGTSRHVSVGI  19493286

Query  121       FSLMSLMVGQVVDREVYLAGFDLNEEGEHAGLSLDGGWNGTVVSNASVVNLTLGGYSMEC  180
                 FSLMSLMVGQVVDREVYLAGFDLNEEGEHAGLSLDGGWNGTVVSNASVVNLTLGGYSMEC
Sbjct  19493287  FSLMSLMVGQVVDREVYLAGFDLNEEGEHAGLSLDGGWNGTVVSNASVVNLTLGGYSMEC  19493466

Query  181       GKECYAISIAASLTFLAGVYQVLMA  205
                 GKECYAISIAASLTFLAGVYQVL A
Sbjct  19493467  GKECYAISIAASLTFLAGVYQVLRA  19493541
```

AE Atlantic herring *slc26a1*

Query: sulfate anion transporter 1 [Clupea harengus] Query ID: XP_012693088.2 Length: 713

```
>Clupea harengus chromosome 12, Ch_v2.0.2, whole genome shotgun sequence
Sequence ID: NC_045163.1 Length: 30022480
Range 1: 25212724 to 25214241

Score:1868 bits(3817), Expect:0.0,
Method:,
Identities:506/506(100%), Positives:506/506(100%), Gaps:0/506(0%)

Query  208       QVLMAVLRLGVVSVYLSGPMLDGFATGASCTILTVQAKYLLGLKIPRYQGYGTVVVTWIN  267
                 QVLMAVLRLGVVSVYLSGPMLDGFATGASCTILTVQAKYLLGLKIPRYQGYGTVVVTWIN
Sbjct  25212724  QVLMAVLRLGVVSVYLSGPMLDGFATGASCTILTVQAKYLLGLKIPRYQGYGTVVVTWIN  25212903

Query  268       IFKNIQNTNFCDMITSAICISILVAGKELQERYKDRLKIPIPTELVVVAGATIASHFADF  327
                 IFKNIQNTNFCDMITSAICISILVAGKELQERYKDRLKIPIPTELVVVAGATIASHFADF
Sbjct  25212904  IFKNIQNTNFCDMITSAICISILVAGKELQERYKDRLKIPIPTELVVVAGATIASHFADF  25213083

Query  328       HGSYSSSISGAIPTGFIPPKVPALDMMSRVVFDAIPLAVISFAFTVSLSEMFAKKHGYSV  387
                 HGSYSSSISGAIPTGFIPPKVPALDMMSRVVFDAIPLAVISFAFTVSLSEMFAKKHGYSV
Sbjct  25213084  HGSYSSSISGAIPTGFIPPKVPALDMMSRVVFDAIPLAVISFAFTVSLSEMFAKKHGYSV  25213263

Query  388       RPNQEMIAIGCCNIIPSFFHCFTTSAALAKTMVKDSTGCQTQVSSIVSAFVVLLVLLFFA  447
                 RPNQEMIAIGCCNIIPSFFHCFTTSAALAKTMVKDSTGCQTQVSSIVSAFVVLLVLLFFA
Sbjct  25213264  RPNQEMIAIGCCNIIPSFFHCFTTSAALAKTMVKDSTGCQTQVSSIVSAFVVLLVLLFFA  25213443

Query  448       PFFYALQKCVLACIIIVSLRGALRKFRDVPQMWRLSKADAVVWIVTMASTALISVELGLV  507
                 PFFYALQKCVLACIIIVSLRGALRKFRDVPQMWRLSKADAVVWIVTMASTALISVELGLV
Sbjct  25213444  PFFYALQKCVLACIIIVSLRGALRKFRDVPQMWRLSKADAVVWIVTMASTALISVELGLV  25213623

Query  508       VGVMFSMMCVVAQTQTPKVSLLGQVDNSSHYDDTEEYHNLQTIPKIKIFRFQSPLYYANK  567
                 VGVMFSMMCVVAQTQTPKVSLLGQVDNSSHYDDTEEYHNLQTIPKIKIFRFQSPLYYANK
Sbjct  25213624  VGVMFSMMCVVAQTQTPKVSLLGQVDNSSHYDDTEEYHNLQTIPKIKIFRFQSPLYYANK  25213803

Query  568       HIFLKSLYKAVELEPFIEMTRRRKATKKAKQLAKKADPTNGEVSIGLVHNDLEFHTIILD  627
                 HIFLKSLYKAVELEPFIEMTRRRKATKKAKQLAKKADPTNGEVSIGLVHNDLEFHTIILD
Sbjct  25213804  HIFLKSLYKAVELEPFIEMTRRRKATKKAKQLAKKADPTNGEVSIGLVHNDLEFHTIILD  25213983

Query  628       GSYIPFIDSTGTTTFKNVQKDYKEVGVTVLLACCNTSVIDSLRRGAFFGPADKDMPSLLF  687
                 GSYIPFIDSTGTTTFKNVQKDYKEVGVTVLLACCNTSVIDSLRRGAFFGPADKDMPSLLF
Sbjct  25213984  GSYIPFIDSTGTTTFKNVQKDYKEVGVTVLLACCNTSVIDSLRRGAFFGPADKDMPSLLF  25214163

Query  688       HTVHSAVVFASDMPSAAAAAAGNTPV  713
                 HTVHSAVVFASDMPSAAAAAAGNTPV
Sbjct  25214164  HTVHSAVVFASDMPSAAAAAAGNTPV  25214241


Range 2: 25211921 to 25212547

Score:773 bits(1577), Expect:0.0,
Method:,
Identities:209/209(100%), Positives:209/209(100%), Gaps:0/209(0%)

Query  1         MGSLLPDNMEDSKSSVIGPLERRVRRRRGPLEVIKSKVNRSLTCSVPRLKGALTGLFPVV  60
                 MGSLLPDNMEDSKSSVIGPLERRVRRRRGPLEVIKSKVNRSLTCSVPRLKGALTGLFPVV
Sbjct  25211921  MGSLLPDNMEDSKSSVIGPLERRVRRRRGPLEVIKSKVNRSLTCSVPRLKGALTGLFPVV  25212100

Query  61        QWLPKYKLKEYVWGDVMSGLIVGIILVPQAIAYCLLAGLEPVYGLYTSFFANIIYFLMGT  120
                 QWLPKYKLKEYVWGDVMSGLIVGIILVPQAIAYCLLAGLEPVYGLYTSFFANIIYFLMGT
Sbjct  25212101  QWLPKYKLKEYVWGDVMSGLIVGIILVPQAIAYCLLAGLEPVYGLYTSFFANIIYFLMGT  25212280

Query  121       SRHVSVGIFSLMSLMVGQVVNREVYLAGFDLDEDTKQGPASGGALNFTEESFGAVMNVSI  180
                 SRHVSVGIFSLMSLMVGQVVNREVYLAGFDLDEDTKQGPASGGALNFTEESFGAVMNVSI
Sbjct  25212281  SRHVSVGIFSLMSLMVGQVVNREVYLAGFDLDEDTKQGPASGGALNFTEESFGAVMNVSI  25212460

Query  181       KVLDMECGKECYAIGIATALTFLAGIYQV  209
                 KVLDMECGKECYAIGIATALTFLAGIYQV
Sbjct  25212461  KVLDMECGKECYAIGIATALTFLAGIYQV  25212547
```

AF Milkfish *slc26a1*

Query: sulfate anion transporter 1-like [Chanos chanos] Query ID: XP_030625776.1 Length: 704

```
>Chanos chanos chromosome 3, fChaCha1.1, whole genome shotgun sequence
Sequence ID: NC_044497.1 Length: 58651890
Range 1: 51486385 to 51487905

Score:1870 bits(3821), Expect:0.0,
Method:,
Identities:506/507(99%), Positives:507/507(100%), Gaps:0/507(0%)

Query  198       YQVLMAVFRMGFVSVYLSAPMLDGFATGASCTILTVQAKYLLGLKIPRHQGLGTVVVTWI  257
                 +QVLMAVFRMGFVSVYLSAPMLDGFATGASCTILTVQAKYLLGLKIPRHQGLGTVVVTWI
```

```
Sbjct  51486385  FQVLMAVFRMGFVSVYLSAPMLDGFATGASCTILTVQAKYLLGLKIPRHQGLGTVVVTWI  51486564

Query  258       NIFKNIHNTNYCDLITSAICIFVLVMGKEIQDKYKDRLKIPLPTELVVVAVATIVSHFAD  317
                 NIFKNIHNTNYCDLITSAICIFVLVMGKEIQDKYKDRLKIPLPTELVVVAVATIVSHFAD
Sbjct  51486565  NIFKNIHNTNYCDLITSAICIFVLVMGKEIQDKYKDRLKIPLPTELVVVAVATIVSHFAD  51486744

Query  318       LNGQYGSSISGSIPTGFIPPKVPSFGLMPRVALDAIPLAVISFAFTVSLSEMFAKKNGYT  377
                 LNGQYGSSISGSIPTGFIPPKVPSFGLMPRVALDAIPLAVISFAFTVSLSEMFAKKNGYT
Sbjct  51486745  LNGQYGSSISGSIPTGFIPPKVPSFGLMPRVALDAIPLAVISFAFTVSLSEMFAKKNGYT  51486924

Query  378       VRPNQEMIAIGFCNIIPSFFHCFTTSAALAKTMVKDSTGCQTQVSSVISALVVLLVLLFF  437
                 VRPNQEMIAIGFCNIIPSFFHCFTTSAALAKTMVKDSTGCQTQVSSVISALVVLLVLLFF
Sbjct  51486925  VRPNQEMIAIGFCNIIPSFFHCFTTSAALAKTMVKDSTGCQTQVSSVISALVVLLVLLFF  51487104

Query  438       APFFYALQKCVLACIIIVSLRGALRKFRDVPKQWHLSKIDAIVWLVSMSSAALISVEIGL  497
                 APFFYALQKCVLACIIIVSLRGALRKFRDVPKQWHLSKIDAIVWLVSMSSAALISVEIGL
Sbjct  51487105  APFFYALQKCVLACIIIVSLRGALRKFRDVPKQWHLSKIDAIVWLVSMSSAALISVEIGL  51487284

Query  498       LIGVVFSMLCVVAQTQKPKVSLLGQVENTSHYDDMEEYNHLLPVPKVKIFRFQAPLYYAN  557
                 LIGVVFSMLCVVAQTQKPKVSLLGQVENTSHYDDMEEYNHLLPVPKVKIFRFQAPLYYAN
Sbjct  51487285  LIGVVFSMLCVVAQTQKPKVSLLGQVENTSHYDDMEEYNHLLPVPKVKIFRFQAPLYYAN  51487464

Query  558       KDFFLKSLYKAVNLEPFLEIARRKKLEKKAKEKKSKQMDETEQANGDDGIGFISTKLDFH  617
                 KDFFLKSLYKAVNLEPFLEIARRKKLEKKAKEKKSKQMDETEQANGDDGIGFISTKLDFH
Sbjct  51487465  KDFFLKSLYKAVNLEPFLEIARRKKLEKKAKEKKSKQMDETEQANGDDGIGFISTKLDFH  51487644

Query  618       TIILDCSSIPFVDTAGMNTLKGLVKDYNGVGVTVLLACCNTSVIDSLRRGSFFGKADIDM  677
                 TIILDCSSIPFVDTAGMNTLKGLVKDYNGVGVTVLLACCNTSVIDSLRRGSFFGKADIDM
Sbjct  51487645  TIILDCSSIPFVDTAGMNTLKGLVKDYNGVGVTVLLACCNTSVIDSLRRGSFFGKADIDM  51487824

Query  678       HSLVFHTVHSAVCFATDTIATVGNTPV  704
                 HSLVFHTVHSAVCFATDTIATVGNTPV
Sbjct  51487825  HSLVFHTVHSAVCFATDTIATVGNTPV  51487905
```

Range 2: 51485413 to 51486012

Score:741 bits(1513), Expect:0.0,
Method:,
Identities:200/200(100%), Positives:200/200(100%), Gaps:0/200(0%)

```
Query  1         MENTKSAMVGPLERKVRKRPRPIATIKTKLCQTLSCSVPKAKSIVTGFFPVVLWLPKYKL  60
                 MENTKSAMVGPLERKVRKRPRPIATIKTKLCQTLSCSVPKAKSIVTGFFPVVLWLPKYKL
Sbjct  51485413  MENTKSAMVGPLERKVRKRPRPIATIKTKLCQTLSCSVPKAKSIVTGFFPVVLWLPKYKL  51485592

Query  61        KEYIWGDIMSGLIVGIILIPQAIAYCLLAGLDPIYGLYTSFFANIIYFFMGTSRHVSVGI  120
                 KEYIWGDIMSGLIVGIILIPQAIAYCLLAGLDPIYGLYTSFFANIIYFFMGTSRHVSVGI
Sbjct  51485593  KEYIWGDIMSGLIVGIILIPQAIAYCLLAGLDPIYGLYTSFFANIIYFFMGTSRHVSVGI  51485772

Query  121       FSLMSLMVGQVVDREVYLAGFDLSEDSKTRTSDGPLNITGHSNSSAINFTINGFGVECGK  180
                 FSLMSLMVGQVVDREVYLAGFDLSEDSKTRTSDGPLNITGHSNSSAINFTINGFGVECGK
Sbjct  51485773  FSLMSLMVGQVVDREVYLAGFDLSEDSKTRTSDGPLNITGHSNSSAINFTINGFGVECGK  51485952

Query  181       ECYAISIATALTLLAGVYQV  200
                 ECYAISIATALTLLAGVYQV
Sbjct  51485953  ECYAISIATALTLLAGVYQV  51486012
```

AG Fathead minnow *slc26a1*

Query: sulfate anion transporter 1 [Pimephales promelas] Query ID: XP_039544144.1 Length: 710

>Pimephales promelas strain EPAAWBERC unplaced genomic scaffold, EPA_FHM_2.0 scaf30, whole genome shotgun sequence
Sequence ID: NW_024121322.1 Length: 8921713
Range 1: 4473079 to 4474596

Score:1859 bits(3799), Expect:0.0,
Method:,
Identities:506/506(100%), Positives:506/506(100%), Gaps:0/506(0%)

```
Query  205      QVLMAVLRLGFVSVYLSAPMLDGFATGASCTILTVQAKYLLGLKIPRHQGYGTVVVTWIN  264
                QVLMAVLRLGFVSVYLSAPMLDGFATGASCTILTVQAKYLLGLKIPRHQGYGTVVVTWIN
Sbjct  4474596  QVLMAVLRLGFVSVYLSAPMLDGFATGASCTILTVQAKYLLGLKIPRHQGYGTVVVTWIN  4474417

Query  265      IFKNIHQTNFCDLITSAICIIVLVAGKEIQDRYKDRLKIPLPTELVVVAVATIVSHFADL  324
                IFKNIHQTNFCDLITSAICIIVLVAGKEIQDRYKDRLKIPLPTELVVVAVATIVSHFADL
Sbjct  4474416  IFKNIHQTNFCDLITSAICIIVLVAGKEIQDRYKDRLKIPLPTELVVVAVATIVSHFADL  4474237

Query  325      NGQFSSSISGAIPTGFIPPKVPSVELMPRVAFDAIPLAVISFAFTVSLSEMFAKKNGYTV  384
                NGQFSSSISGAIPTGFIPPKVPSVELMPRVAFDAIPLAVISFAFTVSLSEMFAKKNGYTV
Sbjct  4474236  NGQFSSSISGAIPTGFIPPKVPSVELMPRVAFDAIPLAVISFAFTVSLSEMFAKKNGYTV  4474057

Query  385      RPNQEMIAIGFCNIIPSFFHSFTTSAALAKTMVKDSTGCQTQVSSVVSALVVLLVLLFFA  444
                RPNQEMIAIGFCNIIPSFFHSFTTSAALAKTMVKDSTGCQTQVSSVVSALVVLLVLLFFA
Sbjct  4474056  RPNQEMIAIGFCNIIPSFFHSFTTSAALAKTMVKDSTGCQTQVSSVVSALVVLLVLLFFA  4473877

Query  445      PFFYALQKCVLACIIIISLRGALRKFRDVPKQWRESKIEAIVWLVTMSSTALISVELGLL  504
                PFFYALQKCVLACIIIISLRGALRKFRDVPKQWRESKIEAIVWLVTMSSTALISVELGLL
Sbjct  4473876  PFFYALQKCVLACIIIISLRGALRKFRDVPKQWRESKIEAIVWLVTMSSTALISVELGLL  4473697
```

```
Query  505      IGVVFSMICVVVQTQNPKVSLLGQIEQTSDYEDMEEYDNLTPIPKVKIFRFQAPLFYANK  564
                IGVVFSMICVVVQTQNPKVSLLGQIEQTSDYEDMEEYDNLTPIPKVKIFRFQAPLFYANK
Sbjct  4473696  IGVVFSMICVVVQTQNPKVSLLGQIEQTSDYEDMEEYDNLTPIPKVKIFRFQAPLFYANK  4473517

Query  565      DFFLKSLYKATDLEPFLEITRRNKLEKKAREKAVKKTGSVDQTNGDVSVSLISKDLDFHT  624
                DFFLKSLYKATDLEPFLEITRRNKLEKKAREKAVKKTGSVDQTNGDVSVSLISKDLDFHT
Sbjct  4473516  DFFLKSLYKATDLEPFLEITRRNKLEKKAREKAVKKTGSVDQTNGDVSVSLISKDLDFHT  4473337

Query  625      IILDCSCISIIDTTGVSTFKAVLKDYKEVGVNVILACCNTTIIDSLSRGSFFGAGDKDME  684
                IILDCSCISIIDTTGVSTFKAVLKDYKEVGVNVILACCNTTIIDSLSRGSFFGAGDKDME
Sbjct  4473336  IILDCSCISIIDTTGVSTFKAVLKDYKEVGVNVILACCNTTIIDSLSRGSFFGAGDKDME  4473157

Query  685      RLSFHTIQSAVRFATGTTQPVNDSSV  710
                RLSFHTIQSAVRFATGTTQPVNDSSV
Sbjct  4473156  RLSFHTIQSAVRFATGTTQPVNDSSV  4473079
```

Range 2: 4475047 to 4475664

Score:767 bits(1566), Expect:0.0,
Method:,
Identities:206/206(100%), Positives:206/206(100%), Gaps:0/206(0%)

```
Query  1        MGLSSLEAMEDLNSVTVGPLKRRVRQRRIHEIIKTKLKRNLSCSGPKVKRTLTGFFPVV  60
                MGLSSLEAMEDLNSVTVGPLKRRVRQRRIHEIIKTKLKRNLSCSGPKVKRTLTGFFPVV
Sbjct  4475664  MGLSSLEAMEDLNSVTVGPLKRRVRQRRIHEIIKTKLKRNLSCSGPKVKRTLTGFFPVV  4475485

Query  61       KWLPKYKVKEYIWGDLMSGLIVGIILIPQAIAYCLLAGLDPIYGLYTSFFANIIYFFMGT  120
                KWLPKYKVKEYIWGDLMSGLIVGIILIPQAIAYCLLAGLDPIYGLYTSFFANIIYFFMGT
Sbjct  4475484  KWLPKYKVKEYIWGDLMSGLIVGIILIPQAIAYCLLAGLDPIYGLYTSFFANIIYFFMGT  4475305

Query  121      SRHVSVGIFSLMSLMIGQVVDREVYLAGFDLNEDNTKNALGLNGTGETNSTVVNLKIMAL  180
                SRHVSVGIFSLMSLMIGQVVDREVYLAGFDLNEDNTKNALGLNGTGETNSTVVNLKIMAL
Sbjct  4475304  SRHVSVGIFSLMSLMIGQVVDREVYLAGFDLNEDNTKNALGLNGTGETNSTVVNLKIMAL  4475125

Query  181      NMECGKECYAISIATALTFLAGVYQV  206
                NMECGKECYAISIATALTFLAGVYQV
Sbjct  4475124  NMECGKECYAISIATALTFLAGVYQV  4475047
```

AH Electric eel *slc26a1*

Query: sulfate anion transporter 1 [Electrophorus electricus] Query ID: XP_026884294.2 Length: 698

>Electrophorus electricus isolate fEleEle1 chromosome 6, fEleEle1.pri, whole genome shotgun sequence
Sequence ID: NC_049540.1 Length: 30780680
Range 1: 24305265 to 24306773

Score:1726 bits(3526), Expect:0.0,
Method:,
Identities:503/503(100%), Positives:503/503(100%), Gaps:0/503(0%)

```
Query  196       ILMAVFRLGFVSVYLSAPMLDGFATGASCTILTVQAKYLLGLKIPRHQGYGTVLVTWINI  255
                 ILMAVFRLGFVSVYLSAPMLDGFATGASCTILTVQAKYLLGLKIPRHQGYGTVLVTWINI
Sbjct  24305265  ILMAVFRLGFVSVYLSAPMLDGFATGASCTILTVQAKYLLGLKIPRHQGYGTVLVTWINI  24305444

Query  256       FKNIHKTNFCDMITSAICITMLLAGKELQDRYKNRLRIPLPTELvvvvvattvSHFADLS  315
                 FKNIHKTNFCDMITSAICITMLLAGKELQDRYKNRLRIPLPTELVVVVVATTVSHFADLS
Sbjct  24305445  FKNIHKTNFCDMITSAICITMLLAGKELQDRYKNRLRIPLPTELVVVVVATTVSHFADLS  24305624

Query  316       GQYssgisgsiPTGFIPPKVPDFGLMPRLVIDAIPLAVISFAFTVSLSEMFAKKNGYTVR  375
                 GQYSSGISGSIPTGFIPPKVPDFGLMPRLVIDAIPLAVISFAFTVSLSEMFAKKNGYTVR
Sbjct  24305625  GQYSSGISGSIPTGFIPPKVPDFGLMPRLVIDAIPLAVISFAFTVSLSEMFAKKNGYTVR  24305804

Query  376       PNQEMLAIGLCNIVPSFFHSFTTSAALAKTMVKDSTGCQTQvssvvsalvvllvllflaP  435
                 PNQEMLAIGLCNIVPSFFHSFTTSAALAKTMVKDSTGCQTQVSSVVSALVVLLVLLFLAP
Sbjct  24305805  PNQEMLAIGLCNIVPSFFHSFTTSAALAKTMVKDSTGCQTQVSSVVSALVVLLVLLFLAP  24305984

Query  436       FFYSLQKCVLACIIIVSLRGALHKFRDVPALWRLSKIDAVVWMVTMCSSALISVEIGLVV  495
                 FFYSLQKCVLACIIIVSLRGALHKFRDVPALWRLSKIDAVVWMVTMCSSALISVEIGLVV
Sbjct  24305985  FFYSLQKCVLACIIIVSLRGALHKFRDVPALWRLSKIDAVVWMVTMCSSALISVEIGLVV  24306164

Query  496       GVVFSMLCVVAQTQNPKVSLLGQIENTNHYEDMDNYNHLVSIPKVKIFRFQAPLYYANKD  555
                 GVVFSMLCVVAQTQNPKVSLLGQIENTNHYEDMDNYNHLVSIPKVKIFRFQAPLYYANKD
Sbjct  24306165  GVVFSMLCVVAQTQNPKVSLLGQIENTNHYEDMDNYNHLVSIPKVKIFRFQAPLYYANKD  24306344

Query  556       FFLKSLYKAVELEPFLEKNRRKKIEKRAKDMVTSRTDKENGDASINLIVSELEFHTIILD  615
                 FFLKSLYKAVELEPFLEKNRRKKIEKRAKDMVTSRTDKENGDASINLIVSELEFHTIILD
Sbjct  24306345  FFLKSLYKAVELEPFLEKNRRKKIEKRAKDMVTSRTDKENGDASINLIVSELEFHTIILD  24306524

Query  616       CSSISFIDTTGISTFKGLLKEYKEVGVNVILACCNTTVLDSLKRSSFFGPDDKEMHTLFF  675
                 CSSISFIDTTGISTFKGLLKEYKEVGVNVILACCNTTVLDSLKRSSFFGPDDKEMHTLFF
Sbjct  24306525  CSSISFIDTTGISTFKGLLKEYKEVGVNVILACCNTTVLDSLKRSSFFGPDDKEMHTLFF  24306704

Query  676       HTVHSAVLFTTHSTVALDSTTPV  698
                 HTVHSAVLFTTHSTVALDSTTPV
Sbjct  24306705  HTVHSAVLFTTHSTVALDSTTPV  24306773
```

Range 2: 24304595 to 24305182

Score:727 bits(1484), Expect:0.0,
Method:,
Identities:195/196(99%), Positives:196/196(100%), Gaps:0/196(0%)

```
Query  1         MEDVKPLPTGLLDRKVQQKKGTVETVKAKLHQKLSCSVPKVKRTLTDFFPVVLWLPKYKL  60
                 MEDVKPLPTGLLDRKVQQKKGTVETVKAKLHQKLSCSVPKVKRTLTDFFPVVLWLPKYKL
Sbjct  24304595  MEDVKPLPTGLLDRKVQQKKGTVETVKAKLHQKLSCSVPKVKRTLTDFFPVVLWLPKYKL  24304774

Query  61        KEYVWGDLMSGLIVGIILVPQAIAYCLLAGLEPVYGLYTSFFSNIIYFILGTSRHVSVGI  120
                 KEYVWGDLMSGLIVGIILVPQAIAYCLLAGLEPVYGLYTSFFSNIIYFILGTSRHVSVGI
Sbjct  24304775  KEYVWGDLMSGLIVGIILVPQAIAYCLLAGLEPVYGLYTSFFSNIIYFILGTSRHVSVGI  24304954

Query  121       FSLMSLMVGQVVDREVYLAGFDLSEDSKRSTGWNGTEDNTTSGEIFMKSFDMECGKECYA  180
                 FSLMSLMVGQVVDREVYLAGFDLSEDSKRSTGWNGTEDNTTSGEIFMKSFDMECGKECYA
Sbjct  24304955  FSLMSLMVGQVVDREVYLAGFDLSEDSKRSTGWNGTEDNTTSGEIFMKSFDMECGKECYA  24305134

Query  181       ISIATALTFLTGTYQI  196
                 ISIATALTFLTGTYQ+
Sbjct  24305135  ISIATALTFLTGTYQV  24305182
```

AI Mexican tetra *slc26a1*

Query: sulfate anion transporter 1 [Astyanax mexicanus] Query ID: XP_049322336.1 Length: 712

>Astyanax mexicanus isolate ESR-SI-001 chromosome 17, AstMex3_surface, whole genome shotgun sequence
Sequence ID: NC_064424.1 Length: 48145406
Range 1: 4223491 to 4225011

Score:1658 bits(3388), Expect:0.0,
Method:,
Identities:506/507(99%), Positives:507/507(100%), Gaps:0/507(0%)

```
Query  206      YQVMMAVFRLGFVSIYLSAPMLDGFATGASCTILTVQAKYLLGLKIPRHQGYGTVVVTWI  265
                +QVMMAVFRLGFVSIYLSAPMLDGFATGASCTILTVQAKYLLGLKIPRHQGYGTVVVTWI
Sbjct  4225011  FQVMMAVFRLGFVSIYLSAPMLDGFATGASCTILTVQAKYLLGLKIPRHQGYGTVVVTWI  4224832

Query  266      NIFKNIHKTNFCDMITSAICIAVLVAGKELQDRYKNRLKMPLPTELVVVGLATVVSHFAD  325
                NIFKNIHKTNFCDMITSAICIAVLVAGKELQDRYKNRLKMPLPTELVVVGLATVVSHFAD
Sbjct  4224831  NIFKNIHKTNFCDMITSAICIAVLVAGKELQDRYKNRLKMPLPTELVVVGLATVVSHFAD  4224652

Query  326      LNGQYSSSISGAIPTGFIPPQVPKFDLMPRIALDAIPLAVISFAFTVSLSEMFAKKNGYT  385
                LNGQYSSSISGAIPTGFIPPQVPKFDLMPRIALDAIPLAVISFAFTVSLSEMFAKKNGYT
Sbjct  4224651  LNGQYSSSISGAIPTGFIPPQVPKFDLMPRIALDAIPLAVISFAFTVSLSEMFAKKNGYT  4224472

Query  386      VKPNQEMLAIGFCNIIPSFFHCFTTSAALAKTMVKDSTGCQTQvssvvsalvvllvllfl  445
                VKPNQEMLAIGFCNIIPSFFHCFTTSAALAKTMVKDSTGCQTQVSSVVSALVVLLVLLFL
Sbjct  4224471  VKPNQEMLAIGFCNIIPSFFHCFTTSAALAKTMVKDSTGCQTQVSSVVSALVVLLVLLFL  4224292

Query  446      aPFFYSLQKCVLACIIIVSLRGALRKFRDVPTLWRLSKIDAAVWIVTMFSAALISVEIGL  505
                APFFYSLQKCVLACIIIVSLRGALRKFRDVPTLWRLSKIDAAVWIVTMFSAALISVEIGL
Sbjct  4224291  APFFYSLQKCVLACIIIVSLRGALRKFRDVPTLWRLSKIDAAVWIVTMFSAALISVEIGL  4224112

Query  506      vigvvvsmicvvvQTQFPKASLLGQIENTNHYEDMDDYSHLVSVPKIKIFRFQAPLYYAN  565
                VIGVVVSMICVVVQTQFPKASLLGQIENTNHYEDMDDYSHLVSVPKIKIFRFQAPLYYAN
Sbjct  4224111  VIGVVVSMICVVVQTQFPKASLLGQIENTNHYEDMDDYSHLVSVPKIKIFRFQAPLYYAN  4223932

Query  566      KEFFLKSLYKAVGLEPFLektrrkkmekkakeraakktmGDNDNGDVSIGLIAPELDFHT  625
                KEFFLKSLYKAVGLEPFLEKTRRKKMEKKAKERAAKKTMGDNDNGDVSIGLIAPELDFHT
Sbjct  4223931  KEFFLKSLYKAVGLEPFLEKTRRKKMEKKAKERAAKKTMGDNDNGDVSIGLIAPELDFHT  4223752

Query  626      IILDCSSMSFIDTTGITTFKGLLKDYKEIGVSIILACCNTNVIDSLRKGSFFGPDDKEMY  685
                IILDCSSMSFIDTTGITTFKGLLKDYKEIGVSIILACCNTNVIDSLRKGSFFGPDDKEMY
Sbjct  4223751  IILDCSSMSFIDTTGITTFKGLLKDYKEIGVSIILACCNTNVIDSLRKGSFFGPDDKEMY  4223572

Query  686      TLSFHTIQSAVSFATDATAASVNSSSV  712
                TLSFHTIQSAVSFATDATAASVNSSSV
Sbjct  4223571  TLSFHTIQSAVSFATDATAASVNSSSV  4223491
```

Range 2: 4226353 to 4226985

Score:773 bits(1577), Expect:0.0,
Method:,
Identities:209/211(99%), Positives:210/211(99%), Gaps:0/211(0%)

```
Query  1        MGSSSAEDMEDVKPLPMGSLERKARKRKGALETLKSKLRNQLSCSVPKVKRMLTGFFPVV  60
                MGSSSAEDMEDVKPLPMGSLERKARKRKGALETLKSKLRNQLSCSVPKVKRMLTGFFPVV
Sbjct  4226985  MGSSSAEDMEDVKPLPMGSLERKARKRKGALETLKSKLRNQLSCSVPKVKRMLTGFFPVV  4226806

Query  61       VWLPKYKINEYVWGDLMSGLIVGIILVPQAIAYCLLAGLEPIYGLYTSFFGNIIYFILGT  120
                VWLPKYKINEYVWGDLMSGLIVGIILVPQAIAYCLLAGLEPIYGLYTSFFGNIIYFILGT
Sbjct  4226805  VWLPKYKINEYVWGDLMSGLIVGIILVPQAIAYCLLAGLEPIYGLYTSFFGNIIYFILGT  4226626
```

```
Query  121      SRHVSVGIFSLMSLMVGQVVDREVYLAGFDLGDESKQSSLDGGWNASEDYSNTALNLTIK  180
                SRHVSVGIFSLMSLMVGQVVDREVYLAGFDLGDESKQSSLDGGWNASEDYSNTALNLTIK
Sbjct  4226625  SRHVSVGIFSLMSLMVGQVVDREVYLAGFDLGDESKQSSLDGGWNASEDYSNTALNLTIK  4226446

Query  181      AFNMECGKECYAISIATALTFLAGIYQVMMA  211
                AFNMECGKECYAISIATALTFLAGIYQV+ A
Sbjct  4226445  AFNMECGKECYAISIATALTFLAGIYQVLSA  4226353
```

AJ Channel catfish *slc26a1*

Query: sulfate anion transporter 1 [Ictalurus punctatus] Query ID: XP_017348765.1 Length: 702

>Ictalurus punctatus breed USDA103 chromosome 18, Coco_2.0, whole genome shotgun sequence
Sequence ID: NC_030433.2 Length: 25178057
Range 1: 20717085 to 20718599

Score:1737 bits(3548), Expect:0.0,
Method:,
Identities:504/505(99%), Positives:505/505(100%), Gaps:0/505(0%)

```
Query  198       YQVLMAVFRLGFVSVYLSAPMLDGFATGASCTILTVQVKYLVGLKIPRHHGYGTVVVTWI  257
                 +QVLMAVFRLGFVSVYLSAPMLDGFATGASCTILTVQVKYLVGLKIPRHHGYGTVVVTWI
Sbjct  20718599  FQVLMAVFRLGFVSVYLSAPMLDGFATGASCTILTVQVKYLVGLKIPRHHGYGTVVVTWI  20718420

Query  258       NIFKNILMTNVCDVITSAICITVLVVGKELQDRYKDRLKIPLPTElvvvalatvvshlaD  317
                 NIFKNILMTNVCDVITSAICITVLVVGKELQDRYKDRLKIPLPTELVVVALATVVSHLAD
Sbjct  20718419  NIFKNILMTNVCDVITSAICITVLVVGKELQDRYKDRLKIPLPTELVVVALATVVSHLAD  20718240

Query  318       LNGQYNSSISGAIPTGFIPPKVPNFELLPRVAIDAIPLAVISFAFTVSLSEMFAKKNGYT  377
                 LNGQYNSSISGAIPTGFIPPKVPNFELLPRVAIDAIPLAVISFAFTVSLSEMFAKKNGYT
Sbjct  20718239  LNGQYNSSISGAIPTGFIPPKVPNFELLPRVAIDAIPLAVISFAFTVSLSEMFAKKNGYT  20718060

Query  378       VRPNQEMLAIGFCNIIPSFFHCFTTSAALAKTMVKDSTGCRTQvssvvsalvvllvllfl  437
                 VRPNQEMLAIGFCNIIPSFFHCFTTSAALAKTMVKDSTGCRTQVSSVVSALVVLLVLLFL
Sbjct  20718059  VRPNQEMLAIGFCNIIPSFFHCFTTSAALAKTMVKDSTGCRTQVSSVVSALVVLLVLLFL  20717880

Query  438       aPFFYSLQKCVLACIIIVSLRGALRKFKDFPRLWRLSKIDAVVWMVTMCSSALISVEIGL  497
                 APFFYSLQKCVLACIIIVSLRGALRKFKDFPRLWRLSKIDAVVWMVTMCSSALISVEIGL
Sbjct  20717879  APFFYSLQKCVLACIIIVSLRGALRKFKDFPRLWRLSKIDAVVWMVTMCSSALISVEIGL  20717700

Query  498       LIGVVFSMICVVAQTQHPKASLLGQIENTNHYEDMDDYDNLVIIPRVKIFRFQAPLYYAN  557
                 LIGVVFSMICVVAQTQHPKASLLGQIENTNHYEDMDDYDNLVIIPRVKIFRFQAPLYYAN
Sbjct  20717699  LIGVVFSMICVVAQTQHPKASLLGQIENTNHYEDMDDYDNLVIIPRVKIFRFQAPLYYAN  20717520

Query  558       KDFFLKSLFKAVGLEPVLEKSRRRKIEKRAKMRTAKQTDKGNGDVSLRLLPSEVDFHTII  617
                 KDFFLKSLFKAVGLEPVLEKSRRRKIEKRAKMRTAKQTDKGNGDVSLRLLPSEVDFHTII
Sbjct  20717519  KDFFLKSLFKAVGLEPVLEKSRRRKIEKRAKMRTAKQTDKGNGDVSLRLLPSEVDFHTII  20717340

Query  618       LDCSSVPFIDTTGINTLKGLIKEYKEVGVNVILACCNTTVIDSLRRGSFFGSDDKEMHTR  677
                 LDCSSVPFIDTTGINTLKGLIKEYKEVGVNVILACCNTTVIDSLRRGSFFGSDDKEMHTR
Sbjct  20717339  LDCSSVPFIDTTGINTLKGLIKEYKEVGVNVILACCNTTVIDSLRRGSFFGSDDKEMHTR  20717160

Query  678       SFHNLPSAVSFATSDSAESVNTTSV  702
                 SFHNLPSAVSFATSDSAESVNTTSV
Sbjct  20717159  SFHNLPSAVSFATSDSAESVNTTSV  20717085
```

Range 2: 20719542 to 20720141

Score:749 bits(1529), Expect:0.0,
Method:,
Identities:200/200(100%), Positives:200/200(100%), Gaps:0/200(0%)

```
Query  1         MEDAKPQYTCHLERKVQQKKGPLKVIRTKLRHQLSCSLPKVKSTLINFFPVVRWLPKYKI  60
                 MEDAKPQYTCHLERKVQQKKGPLKVIRTKLRHQLSCSLPKVKSTLINFFPVVRWLPKYKI
Sbjct  20720141  MEDAKPQYTCHLERKVQQKKGPLKVIRTKLRHQLSCSLPKVKSTLINFFPVVRWLPKYKI  20719962

Query  61        KEYVWGDVMSGLIVGIILVPQAIAYCLLAGLEPVYGLYTSFFANMIYFFLGTSRHVSVGI  120
                 KEYVWGDVMSGLIVGIILVPQAIAYCLLAGLEPVYGLYTSFFANMIYFFLGTSRHVSVGI
Sbjct  20719961  KEYVWGDVMSGLIVGIILVPQAIAYCLLAGLEPVYGLYTSFFANMIYFFLGTSRHVSVGI  20719782

Query  121       FSLMSLMVGQVVDREVYLAGFDLSDDSKQSSLGAGWNGTEDHQDTAVNLTMGAFSMECGK  180
                 FSLMSLMVGQVVDREVYLAGFDLSDDSKQSSLGAGWNGTEDHQDTAVNLTMGAFSMECGK
Sbjct  20719781  FSLMSLMVGQVVDREVYLAGFDLSDDSKQSSLGAGWNGTEDHQDTAVNLTMGAFSMECGK  20719602

Query  181       ECYAISIATVLTFLAGLYQV  200
                 ECYAISIATVLTFLAGLYQV
Sbjct  20719601  ECYAISIATVLTFLAGLYQV  20719542
```

AK Rainbow trout *slc26a1*

Query: sulfate transporter [Oncorhynchus mykiss] Query ID: XP_021458509.1 Length: 705

>Oncorhynchus mykiss isolate Arlee chromosome 5, USDA_OmykA_1.1, whole genome shotgun sequence
Sequence ID: NC_048569.1 Length: 100798064
Range 1: 40252517 to 40254043

Score:1882 bits(3845), Expect:0.0,
Method:,
Identities:508/509(99%), Positives:509/509(100%), Gaps:0/509(0%)

```
Query  197       YQVLMAVFRLGFVSVYLSAPMLDGFATGASFTILTVQAKYLLGLKIPRHQGYGTVVVTWI  256
                 +QVLMAVFRLGFVSVYLSAPMLDGFATGASFTILTVQAKYLLGLKIPRHQGYGTVVVTWI
Sbjct  40252517  FQVLMAVFRLGFVSVYLSAPMLDGFATGASFTILTVQAKYLLGLKIPRHQGYGTVVVTWI  40252696

Query  257       NIFSNIQNTNYCDLITSVICISVLVLGKELQDRYKDRLKIPLPTELVVVAGATLVSHFVD  316
                 NIFSNIQNTNYCDLITSVICISVLVLGKELQDRYKDRLKIPLPTELVVVAGATLVSHFVD
Sbjct  40252697  NIFSNIQNTNYCDLITSVICISVLVLGKELQDRYKDRLKIPLPTELVVVAGATLVSHFVD  40252876

Query  317       FHGRYDSSVSGAIPTGFIPPKVPSFGLMPRVAFDAIPLAVISFAFTVSLSEMFAKKNGYT  376
                 FHGRYDSSVSGAIPTGFIPPKVPSFGLMPRVAFDAIPLAVISFAFTVSLSEMFAKKNGYT
Sbjct  40252877  FHGRYDSSVSGAIPTGFIPPKVPSFGLMPRVAFDAIPLAVISFAFTVSLSEMFAKKNGYT  40253056

Query  377       VRPNQEMLAIGFCNIIPSFFHCFTTSAALAKTMVKDSTGCQTQVSSIVSAFVVLLVLLFF  436
                 VRPNQEMLAIGFCNIIPSFFHCFTTSAALAKTMVKDSTGCQTQVSSIVSAFVVLLVLLFF
Sbjct  40253057  VRPNQEMLAIGFCNIIPSFFHCFTTSAALAKTMVKDSTGCQTQVSSIVSAFVVLLVLLFF  40253236

Query  437       APFFYSLQKCVLACIIIVSLRGALRKFRDVPSKWRVSKMDAVVWMVTMGASALISVEMGL  496
                 APFFYSLQKCVLACIIIVSLRGALRKFRDVPSKWRVSKMDAVVWMVTMGASALISVEMGL
Sbjct  40253237  APFFYSLQKCVLACIIIVSLRGALRKFRDVPSKWRVSKMDAVVWMVTMGASALISVEMGL  40253416

Query  497       VVGVVFSILCIIVQTQKPKVSLLGQVHDTVYYEDLEEYENLMSLPKVKIFRFQAPLYYAN  556
                 VVGVVFSILCIIVQTQKPKVSLLGQVHDTVYYEDLEEYENLMSLPKVKIFRFQAPLYYAN
Sbjct  40253417  VVGVVFSILCIIVQTQKPKVSLLGQVHDTVYYEDLEEYENLMSLPKVKIFRFQAPLYYAN  40253596

Query  557       KDFFLKSLYKAVGVEPFLEMTRRMEAEKKSEKMAAKEVGRDDKTNGEVNVGLVSRELDFH  616
                 KDFFLKSLYKAVGVEPFLEMTRRMEAEKKSEKMAAKEVGRDDKTNGEVNVGLVSRELDFH
Sbjct  40253597  KDFFLKSLYKAVGVEPFLEMTRRMEAEKKSEKMAAKEVGRDDKTNGEVNVGLVSRELDFH  40253776

Query  617       TIILDCSAMPFVDSTGMQTFKGIIKDYKEVGVTVLLASCNTTVIDSLRQGSFFGKADKDM  676
                 TIILDCSAMPFVDSTGMQTFKGIIKDYKEVGVTVLLASCNTTVIDSLRQGSFFGKADKDM
Sbjct  40253777  TIILDCSAMPFVDSTGMQTFKGIIKDYKEVGVTVLLASCNTTVIDSLRQGSFFGKADKDM  40253956

Query  677       ERLAFYTVHTAVQFANDRATSAFIGDTLV  705
                 ERLAFYTVHTAVQFANDRATSAFIGDTLV
Sbjct  40253957  ERLAFYTVHTAVQFANDRATSAFIGDTLV  40254043
```

Range 2: 40251711 to 40252307

Score:742 bits(1515), Expect:0.0,
Method:,
Identities:199/199(100%), Positives:199/199(100%), Gaps:0/199(0%)

```
Query  1         MEEAKVTEVPHLERRVRQRKEPMTVLKTKLSRSLSCSVPRVKNTLTGFFPVVLWLPKYKL  60
                 MEEAKVTEVPHLERRVRQRKEPMTVLKTKLSRSLSCSVPRVKNTLTGFFPVVLWLPKYKL
Sbjct  40251711  MEEAKVTEVPHLERRVRQRKEPMTVLKTKLSRSLSCSVPRVKNTLTGFFPVVLWLPKYKL  40251890

Query  61        KEYIWGDLMSGLIIGIILVPQAIAYCLLAGVDPIYGLYTSFFANIIYFFMGTSRHVSVGI  120
                 KEYIWGDLMSGLIIGIILVPQAIAYCLLAGVDPIYGLYTSFFANIIYFFMGTSRHVSVGI
Sbjct  40251891  KEYIWGDLMSGLIIGIILVPQAIAYCLLAGVDPIYGLYTSFFANIIYFFMGTSRHVSVGI  40252070

Query  121       FSLMSLMVGQVVDREVYMAGFELDDTKAATIDGFLNVTEDSDSSAVNLTIGAFGMECGKE  180
                 FSLMSLMVGQVVDREVYMAGFELDDTKAATIDGFLNVTEDSDSSAVNLTIGAFGMECGKE
Sbjct  40252071  FSLMSLMVGQVVDREVYMAGFELDDTKAATIDGFLNVTEDSDSSAVNLTIGAFGMECGKE  40252250

Query  181       CYAISIAAALTFLAGIYQV  199
                 CYAISIAAALTFLAGIYQV
Sbjct  40252251  CYAISIAAALTFLAGIYQV  40252307
```

AL Argentina silus *slc26a1*

>Argentina silus genome assembly, contig: atg000079l_1, whole genome shotgun sequence
Sequence ID: CATOGY010000076.1 Length: 801731
Range 1: 435050 to 436561

Score:948 bits(2451), Expect:0.0,
Method:Compositional matrix adjust.,
Identities:504/504(100%), Positives:504/504(100%), Gaps:0/504(0%)

```
Query  200     MAVFRLGFVSVYLSAPMLDGFATGASFTILTVQAKYLLGLKIPRHQGYGTVVITWINIFS  259
               MAVFRLGFVSVYLSAPMLDGFATGASFTILTVQAKYLLGLKIPRHQGYGTVVITWINIFS
Sbjct  435050  MAVFRLGFVSVYLSAPMLDGFATGASFTILTVQAKYLLGLKIPRHQGYGTVVITWINIFS  435229

Query  260     NIHKTNMCDLVTSAICISILVAGKELQDRYKDRMKIPLPTELVVVAGATLVSHFGDLNAR  319
               NIHKTNMCDLVTSAICISILVAGKELQDRYKDRMKIPLPTELVVVAGATLVSHFGDLNAR
Sbjct  435230  NIHKTNMCDLVTSAICISILVAGKELQDRYKDRMKIPLPTELVVVAGATLVSHFGDLNAR  435409

Query  320     YGSTVSGHIPTGFIPPEVPSFSLMPRVAFDAIPLAVISFAFTVSLSEMFAKKNGYTVRPN  379
               YGSTVSGHIPTGFIPPEVPSFSLMPRVAFDAIPLAVISFAFTVSLSEMFAKKNGYTVRPN
Sbjct  435410  YGSTVSGHIPTGFIPPEVPSFSLMPRVAFDAIPLAVISFAFTVSLSEMFAKKNGYTVRPN  435589
```

```
Query  380      QEMLAIGLCNIIPSFFHCFTTSAALAKTMVKDSTGCQTQvsslvsafvvllvllffapff  439
                QEMLAIGLCNIIPSFFHCFTTSAALAKTMVKDSTGCQTQVSSLVSAFVVLLVLLFFAPFF
Sbjct  435590  QEMLAIGLCNIIPSFFHCFTTSAALAKTMVKDSTGCQTQVSSLVSAFVVLLVLLFFAPFF  435769

Query  440      YSLQKCVLACIIIVSLRGALRKFRDVPSKWRVSKMDAVVWLVTMSASALISVELGLLVGV  499
                YSLQKCVLACIIIVSLRGALRKFRDVPSKWRVSKMDAVVWLVTMSASALISVELGLLVGV
Sbjct  435770  YSLQKCVLACIIIVSLRGALRKFRDVPSKWRVSKMDAVVWLVTMSASALISVELGLLVGV  435949

Query  500      VFSMLCVVFQTQKPKTSLLGKIANTEHYEDMEEYENLVAPSRVQIFSFQAPLYYANKDFF  559
                VFSMLCVVFQTQKPKTSLLGKIANTEHYEDMEEYENLVAPSRVQIFSFQAPLYYANKDFF
Sbjct  435950  VFSMLCVVFQTQKPKTSLLGKIANTEHYEDMEEYENLVAPSRVQIFSFQAPLYYANKDFF  436129

Query  560      LKSLYKTVGVDPFLEMTRRRkaekkakkelaakqagkNDMSNGEANVSLVAQELDFHTII  619
                LKSLYKTVGVDPFLEMTRRRKAEKKAKKELAAKQAGKNDMSNGEANVSLVAQELDFHTII
Sbjct  436130  LKSLYKTVGVDPFLEMTRRRKAEKKAKKELAAKQAGKNDMSNGEANVSLVAQELDFHTII  436309

Query  620      LDCSAIPFIDSTGMNTFTGIIKDYEKVGVTVLLASCNTTVIDALKRDSFFGKADKDMNRL  679
                LDCSAIPFIDSTGMNTFTGIIKDYEKVGVTVLLASCNTTVIDALKRDSFFGKADKDMNRL
Sbjct  436310  LDCSAIPFIDSTGMNTFTGIIKDYEKVGVTVLLASCNTTVIDALKRDSFFGKADKDMNRL  436489

Query  680      AFYTVHAAVLFANNRATSIGDSVM  703
                AFYTVHAAVLFANNRATSIGDSVM
Sbjct  436490  AFYTVHAAVLFANNRATSIGDSVM  436561
```

Range 2: 433940 to 434569

Score:415 bits(1067), Expect:2e-125,
Method:Compositional matrix adjust.,
Identities:204/213(96%), Positives:205/213(96%), Gaps:3/213(1%)

```
Query  1        MEEGKVTELPPLERRVRQRRQSTAVLRTKLSRSLSCSVPKIKRTLTGFFPVVSWLPRYKL  60
                MEEGKVTELPPLERRVRQRRQSTAVLRTKLSRSLSCSVPKIKRTLTGFFPVVSWLPRYKL
Sbjct  433940  MEEGKVTELPPLERRVRQRRQSTAVLRTKLSRSLSCSVPKIKRTLTGFFPVVSWLPRYKL  434119

Query  61       REYVWGDLMSGLIVGIILVPQAIAYCLLAGVDPIYGLYTSFFANIIYFFMGTSKHVSVGI  120
                REYVWGDLMSGLIVGIILVPQAIAYCLLAGVDPIYGLYTSFFANIIYFFMGTSKHVSVGI
Sbjct  434120  REYVWGDLMSGLIVGIILVPQAIAYCLLAGVDPIYGLYTSFFANIIYFFMGTSKHVSVGI  434299

Query  121      FSLMSLMVGQVVDREVYLAGFDLNDEGQAGTSAWNGTEGSNSSAVSLTVGVLGVECGKEC  180
                FSLMSLMVGQVVDREVYLAGFDLNDEGQAGTSAWNGTEGSNSSAVSLTVGVLGVECGKEC
Sbjct  434300  FSLMSLMVGQVVDREVYLAGFDLNDEGQAGTSAWNGTEGSNSSAVSLTVGVLGVECGKEC  434479

Query  181      YAISIAAAMTFLAGIYQVLMAVFRLGFVSVYLS  213
                YAISIAAAMTFLAGIYQV    FR    S+YLS
Sbjct  434480  YAISIAAAMTFLAGIYQVHFQTFRF---SIYLS  434569
```

AM Large-eye snaggletooth *slc26a1*

Query: unnamed protein product Query ID: lcl|Query_295957 Length: 701

>Borostomias antarcticus genome assembly, contig: atg001449l_1, whole genome shotgun sequence
Sequence ID: CATLJU010001285.1 Length: 124978
Range 1: 64549 to 65862

Score:1430 bits(2922), Expect:0.0,
Method:,
Identities:438/438(100%), Positives:438/438(100%), Gaps:0/438(0%)

```
Query  264    SAICISVLVAGKELQERFKSRLKIPLPTElvvvtvatlaSHFGDLNGQYDSSVSGHIPTG  323
              SAICISVLVAGKELQERFKSRLKIPLPTELVVVTVATLASHFGDLNGQYDSSVSGHIPTG
Sbjct  64549  SAICISVLVAGKELQERFKSRLKIPLPTELVVVTVATLASHFGDLNGQYDSSVSGHIPTG  64728

Query  324    FIPPKVPSFDLMPRVALDAIPLAVISFAFTVSLSEMFAKKNGYTVRPNQEMLAIGCCNII  383
              FIPPKVPSFDLMPRVALDAIPLAVISFAFTVSLSEMFAKKNGYTVRPNQEMLAIGCCNII
Sbjct  64729  FIPPKVPSFDLMPRVALDAIPLAVISFAFTVSLSEMFAKKNGYTVRPNQEMLAIGCCNII  64908

Query  384    PSFFHCFTTSAALAKTMVKDSTGCQTQvsslvsalvvllvllFFAPYFYALQKCVLACII  443
              PSFFHCFTTSAALAKTMVKDSTGCQTQVSSLVSALVVLLVLLFFAPYFYALQKCVLACII
Sbjct  64909  PSFFHCFTTSAALAKTMVKDSTGCQTQVSSLVSALVVLLVLLFFAPYFYALQKCVLACII  65088

Query  444    IVSLRGALRKFLDVPGKWRASKTDAVVWLVTMASSALISVELGLLVGVVFSMLCVIFQTQ  503
              IVSLRGALRKFLDVPGKWRASKTDAVVWLVTMASSALISVELGLLVGVVFSMLCVIFQTQ
Sbjct  65089  IVSLRGALRKFLDVPGKWRASKTDAVVWLVTMASSALISVELGLLVGVVFSMLCVIFQTQ  65268

Query  504    NPAVSLLGRADDTELYEDMEEYQNLSAAPGVRIFRFQAPLYYANKESFLKSLYKAVGLEP  563
              NPAVSLLGRADDTELYEDMEEYQNLSAAPGVRIFRFQAPLYYANKESFLKSLYKAVGLEP
Sbjct  65269  NPAVSLLGRADDTELYEDMEEYQNLSAAPGVRIFRFQAPLYYANKESFLKSLYKAVGLEP  65448

Query  564    FLEVTRRRKAQKKAQELVAKQAKDEGVEGSDKTNGEVSVRLVNGELEFHTIVLDCSAMPF  623
              FLEVTRRRKAQKKAQELVAKQAKDEGVEGSDKTNGEVSVRLVNGELEFHTIVLDCSAMPF
Sbjct  65449  FLEVTRRRKAQKKAQELVAKQAKDEGVEGSDKTNGEVSVRLVNGELEFHTIVLDCSAMPF  65628

Query  624    VDTAGVGTFRSVLKDYQEVGVSVLLAACNTAVIDALSRASYFGKGDADMDSLMFHSvhaa  683
              VDTAGVGTFRSVLKDYQEVGVSVLLAACNTAVIDALSRASYFGKGDADMDSLMFHSVHAA
```

Sbjct 65629 VDTAGVGTFRSVLKDYQEVGVSVLLAACNTAVIDALSRASYFGKGDADMDSLMFHSVHAA 65808

Query 684 vlhagdgadadaaSTSVV 701
VLHAGDGADADAASTSVV
Sbjct 65809 VLHAGDGADADAASTSVV 65862

Range 2: 62593 to 63165

Score:624 bits(1273), Expect:2e-177,
Method:,
Identities:191/191(100%), Positives:191/191(100%), Gaps:0/191(0%)

Query 1 MEEVKTprpaalqrrlrqrkrplqvlrTRVTRGVTCSVPRLRSTLQGFFPVVRWLPRYRL 60
MEEVKTPRPAALQRRLRQRKRPLQVLRTRVTRGVTCSVPRLRSTLQGFFPVVRWLPRYRL
Sbjct 62593 MEEVKTPRPAALQRRLRQRKRPLQVLRTRVTRGVTCSVPRLRSTLQGFFPVVRWLPRYRL 62772

Query 61 REYVWGDAMSGLIVGIILVPQAIAYCLLAGVQPIYGLYTSFFANIIYFFMGTSRHVSVGI 120
REYVWGDAMSGLIVGIILVPQAIAYCLLAGVQPIYGLYTSFFANIIYFFMGTSRHVSVGI
Sbjct 62773 REYVWGDAMSGLIVGIILVPQAIAYCLLAGVQPIYGLYTSFFANIIYFFMGTSRHVSVGI 62952

Query 121 FSLMSLMVGQVVDREVFQAGFDLGEEGAPGNGSVSPSYLSPNGTEMECGKECYAISIAAA 180
FSLMSLMVGQVVDREVFQAGFDLGEEGAPGNGSVSPSYLSPNGTEMECGKECYAISIAAA
Sbjct 62953 FSLMSLMVGQVVDREVFQAGFDLGEEGAPGNGSVSPSYLSPNGTEMECGKECYAISIAAA 63132

Query 181 VTFLAGIYQVL 191
VTFLAGIYQVL
Sbjct 63133 VTFLAGIYQVL 63165

Range 3: 63778 to 64002

Score:282 bits(573), Expect:3e-74,
Method:,
Identities:75/75(100%), Positives:75/75(100%), Gaps:0/75(0%)

Query 189 QVLMAVFRLGFVSVYLSAPMLDGFATGASFTILTVQAKYLLGLRIPRHQGYGTVVVTWFR 248
QVLMAVFRLGFVSVYLSAPMLDGFATGASFTILTVQAKYLLGLRIPRHQGYGTVVVTWFR
Sbjct 63778 QVLMAVFRLGFVSVYLSAPMLDGFATGASFTILTVQAKYLLGLRIPRHQGYGTVVVTWFR 63957

Query 249 IFSNIQHANMCDLVT 263
IFSNIQHANMCDLVT
Sbjct 63958 IFSNIQHANMCDLVT 64002

AN European smelt *slc26a1*

Query: sulfate anion transporter 1 [Osmerus eperlanus] ID: XP_062334655.1(amino acid) Length: 709

>Osmerus eperlanus genome assembly, contig: scf7180003266451, whole genome shotgun sequence
Sequence ID: OMKJ01042897.1 Length: 21657
Range 1: 4789 to 6129

Score:844 bits(2180), Expect:0.0,
Method:Compositional matrix adjust.,
Identities:445/447(99%), Positives:446/447(99%), Gaps:0/447(0%)

Query 263 ITSAICIFVLVAGKELQERYKDRLKIPLPTELLVVAGATLTSHFGDLHGRYDSSVSGHIP 322
+ SAICIFVLVAGKELQERYKDRLKIPLPTELLVVAGATLTSHFGDLHGRYDSSVSGHIP
Sbjct 4789 VPSAICIFVLVAGKELQERYKDRLKIPLPTELLVVAGATLTSHFGDLHGRYDSSVSGHIP 4968

Query 323 TGFIPPTVPNFGLMPRVALDAIPLAVISFAFTVSLSEMFAKKNGYTVRPNQEMLAIGCCN 382
TGFIPPTVPNFGLMPRVALDAIPLAVISFAFTVSLSEMFAKKNGYTVRPNQEMLAIGCCN
Sbjct 4969 TGFIPPTVPNFGLMPRVALDAIPLAVISFAFTVSLSEMFAKKNGYTVRPNQEMLAIGCCN 5148

Query 383 IIPSFFHCFTTSAALAKTMVKDSTGCQTQvsslvsalvvllvllFFAPYFHALQKCVLAC 442
IIPSFFHCFTTSAALAKTMVKDSTGCQTQVSSLVSALVVLLVLLFFAPYFHALQKCVLAC
Sbjct 5149 IIPSFFHCFTTSAALAKTMVKDSTGCQTQVSSLVSALVVLLVLLFFAPYFHALQKCVLAC 5328

Query 443 IIIVSLRGALRKFRDVPSKWRASKTDAVVWLVTVAASALITVELGLLVGVVFSMICVIFQ 502
IIIVSLRGALRKFRDVPSKWRASKTDAVVWLVTVAASALITVELGLLVGVVFSMICVIFQ
Sbjct 5329 IIIVSLRGALRKFRDVPSKWRASKTDAVVWLVTVAASALITVELGLLVGVVFSMICVIFQ 5508

Query 503 TQNPKVSLLGQAGTSELYEDIAEYKDLSAPSGVRIFRFQAPLYYANKETFLKSLYKAVGL 562
TQNPKVSLLGQAGTSELYEDIAEYKDLSAPSGVRIFRFQAPLYYANKETFLKSLYKAVGL
Sbjct 5509 TQNPKVSLLGQAGTSELYEDIAEYKDLSAPSGVRIFRFQAPLYYANKETFLKSLYKAVGL 5688

Query 563 EPFLELTRRRkaqkksllaakqaeasRGDKTNGDAIVGLVNADLLFHTIVLDCSSVPFID 622
EPFLELTRRRKAQKKSLLAAKQAEASRGDKTNGDAIVGLVNADLLFHTIVLDCSSVPFID
Sbjct 5689 EPFLELTRRRKAQKKSLLAAKQAEASRGDKTNGDAIVGLVNADLLFHTIVLDCSSVPFID 5868

Query 623 SAGMGTLKAVLKEYQEVDVSVLLAVCNTNVIDTLRRGSYFGKNDKDIDSLLFHTVHSAVL 682
SAGMGTLKAVLKEYQEVDVSVLLAVCNTNVIDTLRRGSYFGKNDKDIDSLLFHTVHSAVL
Sbjct 5869 SAGMGTLKAVLKEYQEVDVSVLLAVCNTNVIDTLRRGSYFGKNDKDIDSLLFHTVHSAVL 6048

Query 683 YASEVANTADKAAGDTWPGETAEDSVV 709
YASEVANTADKAAGDTWPGETAEDSVV

```
Sbjct  6049  YASEVANTADKAAGDTWPGETAEDSVV  6129
```

Range 2: 3765 to 4709

Score:520 bits(1338), Expect:0.0,
Method:Compositional matrix adjust.,
Identities:265/315(84%), Positives:265/315(84%), Gaps:48/315(15%)

```
Query  1     MEAVMTPRSCVPSLERRARQRKPPLEVLKTKVKQGLACSVPRVRSTLTGFFPVVRWLPRY  60
             MEAVMTPRSCVPSLERRARQRKPPLEVLKTKVKQGLACSVPRVRSTLTGFFPVVRWLPRY
Sbjct  3765  MEAVMTPRSCVPSLERRARQRKPPLEVLKTKVKQGLACSVPRVRSTLTGFFPVVRWLPRY  3944

Query  61    KLREYAWGDAMSGLIVGIILVPQAIAYCLLAGVDPIYGLYTSFFANIIYFFMGTSKHVSV  120
             KLREYAWGDAMSGLIVGIILVPQAIAYCLLAGVDPIYGLYTSFFANIIYFFMGTSKHVSV
Sbjct  3945  KLREYAWGDAMSGLIVGIILVPQAIAYCLLAGVDPIYGLYTSFFANIIYFFMGTSKHVSV  4124

Query  121   GIFSLMSLMVGQVVDREVFLAGYDLNEDGASAVLNDSLLVNGTAGGLKGEKEYYAISIAA  180
             GIFSLMSLMVGQVVDREVFLAGYDLNEDGASAVLNDSLLVNGTAGGLKGEKEYYAISIAA
Sbjct  4125  GIFSLMSLMVGQVVDREVFLAGYDLNEDGASAVLNDSLLVNGTAGGLKGEKEYYAISIAA  4304

Query  181   AVTFLAGIY------------------------------------------------QVL  192
             AVTFLAGIY                                                QVL
Sbjct  4305  AVTFLAGIYQVLSLCLRSCVCVWING*KDGICRWMDG*TEL*MIYDMQTCLALPLPAQVL  4484

Query  193   MAVFRLGFVSVFLSAPMLDGFATGASFTILTVQAKYLLGLKIPRYQGYGTVVVTWVNIFS  252
             MAVFRLGFVSVFLSAPMLDGFATGASFTILTVQAKYLLGLKIPRYQGYGTVVVTWVNIFS
Sbjct  4485  MAVFRLGFVSVFLSAPMLDGFATGASFTILTVQAKYLLGLKIPRYQGYGTVVVTWVNIFS  4664

Query  253   NIHHTNLCDLITSAI  267
             NIHHTNLCDLIT  I
Sbjct  4665  NIHHTNLCDLITRLI  4709
```

AO Ayu *slc26a1*

Query: unnamed protein product Query ID: lcl|Query_332235 Length: 709

>Plecoglossus altivelis isolate Fa20170821-1 scaf41_2, whole genome shotgun sequence
Sequence ID: SDAO01004358.1 Length: 6214464
Range 1: 1270792 to 1272132

Score:1426 bits(2913), Expect:0.0,
Method:,
Identities:446/447(99%), Positives:446/447(99%), Gaps:0/447(0%)

```
Query  263      ITSAICIFVLVAGKELQERYKDRMKIPLPTELVVVAGATLASHFGDLNGRYDSSVSGHIP  322
                I SAICIFVLVAGKELQERYKDRMKIPLPTELVVVAGATLASHFGDLNGRYDSSVSGHIP
Sbjct  1270792  IPSAICIFVLVAGKELQERYKDRMKIPLPTELVVVAGATLASHFGDLNGRYDSSVSGHIP  1270971

Query  323      TGFIAPTVPNFSLMPRVALDAIPLAVISFAFTVSLSEMFAKKNGYTVRPNQEMLAIGCCN  382
                TGFIAPTVPNFSLMPRVALDAIPLAVISFAFTVSLSEMFAKKNGYTVRPNQEMLAIGCCN
Sbjct  1270972  TGFIAPTVPNFSLMPRVALDAIPLAVISFAFTVSLSEMFAKKNGYTVRPNQEMLAIGCCN  1271151

Query  383      IIPSFFHCFTTSAALAKTMVKDSTGCQTQvsslisalvvllvllFFAPYFHALQKCVLAC  442
                IIPSFFHCFTTSAALAKTMVKDSTGCQTQVSSLISALVVLLVLLFFAPYFHALQKCVLAC
Sbjct  1271152  IIPSFFHCFTTSAALAKTMVKDSTGCQTQVSSLISALVVLLVLLFFAPYFHALQKCVLAC  1271331

Query  443      IIIVSLRGALRKFRDVPSKWRASKTDAVVWLVTMASSALITVELGLLVGVVFSMICVIFQ  502
                IIIVSLRGALRKFRDVPSKWRASKTDAVVWLVTMASSALITVELGLLVGVVFSMICVIFQ
Sbjct  1271332  IIIVSLRGALRKFRDVPSKWRASKTDAVVWLVTMASSALITVELGLLVGVVFSMICVIFQ  1271511

Query  503      TQNPKVSLLGQAGSSELYEDIAEYKDLSAPSAIHIFRFQAPLYYANKESFLKSLYKAVGL  562
                TQNPKVSLLGQAGSSELYEDIAEYKDLSAPSAIHIFRFQAPLYYANKESFLKSLYKAVGL
Sbjct  1271512  TQNPKVSLLGQAGSSELYEDIAEYKDLSAPSAIHIFRFQAPLYYANKESFLKSLYKAVGL  1271691

Query  563      EPFLELTrrrkaqkksllaakqaaasrGDKTNGEAIVGLVNAEPPFHTIVLDCSSVPFID  622
                EPFLELTRRRKAQKKSLLAAKQAAASRGDKTNGEAIVGLVNAEPPFHTIVLDCSSVPFID
Sbjct  1271692  EPFLELTRRRKAQKKSLLAAKQAAASRGDKTNGEAIVGLVNAEPPFHTIVLDCSSVPFID  1271871

Query  623      SAGVGTLKAVLKEYLEVGVSVLLAGCNTNIIDSLKRGSYFGKNDKDAQSLLFHTVHSAVL  682
                SAGVGTLKAVLKEYLEVGVSVLLAGCNTNIIDSLKRGSYFGKNDKDAQSLLFHTVHSAVL
Sbjct  1271872  SAGVGTLKAVLKEYLEVGVSVLLAGCNTNIIDSLKRGSYFGKNDKDAQSLLFHTVHSAVL  1272051

Query  683      YankvanaadraagnTSPGEMAEDSVV  709
                YANKVANAADRAAGNTSPGEMAEDSVV
Sbjct  1272052  YANKVANAADRAAGNTSPGEMAEDSVV  1272132
```

Range 2: 1269741 to 1270316

Score:705 bits(1439), Expect:0.0,
Method:,
Identities:191/192(99%), Positives:191/192(99%), Gaps:0/192(0%)

```
Query  1        METVNTPGSCVPSLERRARQRKPPLEVLQTKVKRGLTCSVPRVRSTLTGFFPVVRWLPRY  60
                METVNTPGSCVPSLERRARQRKPPLEVLQTKVKRGLTCSVPRVRSTLTGFFPVVRWLPRY
Sbjct  1269741  METVNTPGSCVPSLERRARQRKPPLEVLQTKVKRGLTCSVPRVRSTLTGFFPVVRWLPRY  1269920
```

```
Query  61       KLQEYAWGDIMSGLIVGIILVPQAIAYCLLAGVDPIYGLYTSFFANIIYFFMGTSKHVSV  120
                KLQEYAWGDIMSGLIVGIILVPQAIAYCLLAGVDPIYGLYTSFFANIIYFFMGTSKHVSV
Sbjct  1269921  KLQEYAWGDIMSGLIVGIILVPQAIAYCLLAGVDPIYGLYTSFFANIIYFFMGTSKHVSV  1270100

Query  121      GIFSLMSLMVGQVVDREVFLAGYDLNDDVASTVLNDSLLVNGTAGVLKGEKEYYAISVAA  180
                GIFSLMSLMVGQVVDREVFLAGYDLNDDVAS VLNDSLLVNGTAGVLKGEKEYYAISVAA
Sbjct  1270101  GIFSLMSLMVGQVVDREVFLAGYDLNDDVASAVLNDSLLVNGTAGVLKGEKEYYAISVAA  1270280

Query  181      AVTFLAGIYQVL  192
                AVTFLAGIYQVL
Sbjct  1270281  AVTFLAGIYQVL  1270316
```

Range 3: 1270473 to 1270697

Score:280 bits(569), Expect:2e-73,
Method:,
Identities:75/75(100%), Positives:75/75(100%), Gaps:0/75(0%)

```
Query  190      QVLMAVFRLGFVSVFLSAPMLDGFATGASFTILTVQAKYLLGLKIPRYQGYGTVVVTWVN  249
                QVLMAVFRLGFVSVFLSAPMLDGFATGASFTILTVQAKYLLGLKIPRYQGYGTVVVTWVN
Sbjct  1270473  QVLMAVFRLGFVSVFLSAPMLDGFATGASFTILTVQAKYLLGLKIPRYQGYGTVVVTWVN  1270652

Query  250      IFSNIHHTNLCDLIT  264
                IFSNIHHTNLCDLIT
Sbjct  1270653  IFSNIHHTNLCDLIT  1270697
```

AP Peladilla *slc26a1*

Query: unnamed protein product Query ID: lcl|Query_5509087 Length: 715

>Aplochiton taeniatus voucher 6995 isolate fAplTae1 001287F_arrow_arrow, whole genome shotgun sequence
Sequence ID: JAGFOW010004266.1 Length: 169766
Range 1: 38345 to 39142

Score:896 bits(1829), Expect:0.0,
Method:,
Identities:266/266(100%), Positives:266/266(100%), Gaps:0/266(0%)

```
Query  450    QKCVLACIIIVSLRGALRKILDVPSKWRASRADAVVWLVAMGATALISVELGLVVGVVFS  509
              QKCVLACIIIVSLRGALRKILDVPSKWRASRADAVVWLVAMGATALISVELGLVVGVVFS
Sbjct  39142  QKCVLACIIIVSLRGALRKILDVPSKWRASRADAVVWLVAMGATALISVELGLVVGVVFS  38963

Query  510    MLCVIVQTQRPKVALLGRVADTDFYEDIKEYQNLTAPEGVQIFRFQAPLYYANKDSFLKA  569
              MLCVIVQTQRPKVALLGRVADTDFYEDIKEYQNLTAPEGVQIFRFQAPLYYANKDSFLKA
Sbjct  38962  MLCVIVQTQRPKVALLGRVADTDFYEDIKEYQNLTAPEGVQIFRFQAPLYYANKDSFLKA  38783

Query  570    LYKAVGLEPFLestrrrkaekkarelasklPKSAGENNNSNGDVSTSLVVGENKFHTIIL  629
              LYKAVGLEPFLESTRRRKAEKKARELASKLPKSAGENNNSNGDVSTSLVVGENKFHTIIL
Sbjct  38782  LYKAVGLEPFLESTRRRKAEKKARELASKLPKSAGENNNSNGDVSTSLVVGENKFHTIIL  38603

Query  630    DCSAMPFMDSTGMGTFKGLVKDYSEVGVNLLLASCNTNVIDTLRKGLFFGKGDKDMDSML  689
              DCSAMPFMDSTGMGTFKGLVKDYSEVGVNLLLASCNTNVIDTLRKGLFFGKGDKDMDSML
Sbjct  38602  DCSAMPFMDSTGMGTFKGLVKDYSEVGVNLLLASCNTNVIDTLRKGLFFGKGDKDMDSML  38423

Query  690    FYTIHAAVLYANSQVAPPALPADSAV  715
              FYTIHAAVLYANSQVAPPALPADSAV
Sbjct  38422  FYTIHAAVLYANSQVAPPALPADSAV  38345
```

Range 2: 39362 to 40027

Score:814 bits(1662), Expect:0.0,
Method:,
Identities:222/222(100%), Positives:222/222(100%), Gaps:0/222(0%)

```
Query  204    QVLMAVFRLGFVSVYLSAPMLDGFATGASFTILTVQAKYLLGLKIARHQGYGTVVVTWVR  263
              QVLMAVFRLGFVSVYLSAPMLDGFATGASFTILTVQAKYLLGLKIARHQGYGTVVVTWVR
Sbjct  40027  QVLMAVFRLGFVSVYLSAPMLDGFATGASFTILTVQAKYLLGLKIARHQGYGTVVVTWVR  39848

Query  264    ILSNIHHTNLCDLVTSAICIFVLVAGKELQERFKDRLKIPIPTELVVVLGATLASHFGDL  323
              ILSNIHHTNLCDLVTSAICIFVLVAGKELQERFKDRLKIPIPTELVVVLGATLASHFGDL
Sbjct  39847  ILSNIHHTNLCDLVTSAICIFVLVAGKELQERFKDRLKIPIPTELVVVLGATLASHFGDL  39668

Query  324    HGLYGSSVSGHIPTGFISPTVPSFGLMPRVALDAVPLAVISFAFTVSLSEMFAKKNGYTV  383
              HGLYGSSVSGHIPTGFISPTVPSFGLMPRVALDAVPLAVISFAFTVSLSEMFAKKNGYTV
Sbjct  39667  HGLYGSSVSGHIPTGFISPTVPSFGLMPRVALDAVPLAVISFAFTVSLSEMFAKKNGYTV  39488

Query  384    RPNQEMLAIGCCNIIPSFFHCFTTSAALAKTMVKDSTGCQTQ  425
              RPNQEMLAIGCCNIIPSFFHCFTTSAALAKTMVKDSTGCQTQ
Sbjct  39487  RPNQEMLAIGCCNIIPSFFHCFTTSAALAKTMVKDSTGCQTQ  39362
```

Range 3: 40823 to 41230

Score:514 bits(1048), Expect:3e-144,
Method:,
Identities:136/136(100%), Positives:136/136(100%), Gaps:0/136(0%)

```
Query  1      MENHDGVTEILPPPPLLERCARQRRPPLSVLRTRVKQGLTCSVPRVKATLTGFFPVVHWL  60
              MENHDGVTEILPPPPLLERCARQRRPPLSVLRTRVKQGLTCSVPRVKATLTGFFPVVHWL
Sbjct  41230  MENHDGVTEILPPPPLLERCARQRRPPLSVLRTRVKQGLTCSVPRVKATLTGFFPVVHWL  41051

Query  61     PKYKLREYVWGDLMSGLIVGIILVPQAIAYCLLAGVDPIYGLYTSFFANIIYFFMGTSRH  120
              PKYKLREYVWGDLMSGLIVGIILVPQAIAYCLLAGVDPIYGLYTSFFANIIYFFMGTSRH
Sbjct  41050  PKYKLREYVWGDLMSGLIVGIILVPQAIAYCLLAGVDPIYGLYTSFFANIIYFFMGTSRH  40871

Query  121    VSVGIFSLMSLMVGQV  136
              VSVGIFSLMSLMVGQV
Sbjct  40870  VSVGIFSLMSLMVGQV  40823
```

Range 4: 40189 to 40401

Score:265 bits(539), Expect:3e-69,
Method:,
Identities:71/71(100%), Positives:71/71(100%), Gaps:0/71(0%)

```
Query  135    QVVDREVYLAGFDMSDGSTVTMAPGMWNDTGPGDTNLTAAGVEVMGMECGKECYAISVAA  194
              QVVDREVYLAGFDMSDGSTVTMAPGMWNDTGPGDTNLTAAGVEVMGMECGKECYAISVAA
Sbjct  40401  QVVDREVYLAGFDMSDGSTVTMAPGMWNDTGPGDTNLTAAGVEVMGMECGKECYAISVAA  40222

Query  195    ATTFLAGIYQV  205
              ATTFLAGIYQV
Sbjct  40221  ATTFLAGIYQV  40189
```

AQ Antarctic jonasfish *slc26a1*

Query: unnamed protein product Query ID: lcl|Query_1298795 Length: 719

>Notolepis coatsorum genome assembly, contig: atg020714l_1, whole genome shotgun sequence
Sequence ID: CAXASL010016664.1 Length: 117332
Range 1: 57594 to 58175

Score:400 bits(1028), Expect:3e-119,
Method:Compositional matrix adjust.,
Identities:193/194(99%), Positives:194/194(100%), Gaps:0/194(0%)

```
Query  526    KVSILGRVTDSELYEDMGEYQNLMPPTRVRIFRFQSPLYYANKDSFLKSLYKTVGLEPFM  585
              +VSILGRVTDSELYEDMGEYQNLMPPTRVRIFRFQSPLYYANKDSFLKSLYKTVGLEPFM
Sbjct  58175  QVSILGRVTDSELYEDMGEYQNLMPPTRVRIFRFQSPLYYANKDSFLKSLYKTVGLEPFM  57996

Query  586    ELTRRSKVEKKAKELTKQAKGEVNKANGEVSIALVHRELDFHTIVLDCSAIPFIDTTGVA  645
              ELTRRSKVEKKAKELTKQAKGEVNKANGEVSIALVHRELDFHTIVLDCSAIPFIDTTGVA
Sbjct  57995  ELTRRSKVEKKAKELTKQAKGEVNKANGEVSIALVHRELDFHTIVLDCSAIPFIDTTGVA  57816

Query  646    TFNGLVKDYKEVGVSVALASCNTSVIDSLKKGAFFGKDDRDTGSVLFYTVHAAVLHADSR  705
              TFNGLVKDYKEVGVSVALASCNTSVIDSLKKGAFFGKDDRDTGSVLFYTVHAAVLHADSR
Sbjct  57815  TFNGLVKDYKEVGVSVALASCNTSVIDSLKKGAFFGKDDRDTGSVLFYTVHAAVLHADSR  57636

Query  706    AAAKMTPMAADSEV  719
              AAAKMTPMAADSEV
Sbjct  57635  AAAKMTPMAADSEV  57594
```

Range 2: 67335 to 67751

Score:229 bits(583), Expect:7e-61,
Method:Compositional matrix adjust.,
Identities:138/139(99%), Positives:139/139(100%), Gaps:0/139(0%)

```
Query  1      MQEAAKVPEPVSApppLhlerrirhrrpplsllrsklkQGVTCSTPRVRATLTGFFPVVR  60
              MQEAAKVPEPVSAPPPLHLERRIRHRRPPLSLLRSKLKQGVTCSTPRVRATLTGFFPVVR
Sbjct  67751  MQEAAKVPEPVSAPPPLHLERRIRHRRPPLSLLRSKLKQGVTCSTPRVRATLTGFFPVVR  67572

Query  61     WLPKYKLREYVWGDVMSGLIVGIILVPQAIAYCLLAGVEPVYGLYTSFFANIIYFFMGTS  120
              WLPKYKLREYVWGDVMSGLIVGIILVPQAIAYCLLAGVEPVYGLYTSFFANIIYFFMGTS
Sbjct  67571  WLPKYKLREYVWGDVMSGLIVGIILVPQAIAYCLLAGVEPVYGLYTSFFANIIYFFMGTS  67392

Query  121    RHVSVGIFSLMSLMVGQVV  139
              RHVSVGIFSLMSLMVGQV+
Sbjct  67391  RHVSVGIFSLMSLMVGQVI  67335
```

Range 3: 58795 to 59196

Score:184 bits(466), Expect:5e-46,
Method:Compositional matrix adjust.,
Identities:92/142(65%), Positives:108/142(76%), Gaps:8/142(5%)

```
Query  446    FFAPCFYALQKCVLACIIIVSLRGALRKFRDVPAKWRDSRIDAVVWLVTMASTALISVEL  505
```

```
              FFAPCFYALQKCVLACIIIVSLRGALRKFRDVPAKWRDSRIDAVVWLVTMASTALISVEL
Sbjct  59196  FFAPCFYALQKCVLACIIIVSLRGALRKFRDVPAKWRDSRIDAVVWLVTMASTALISVEL  59017

Query  506    GLLVGIVFSMLCVICQTQNPKVSILGRVTDSELYEDMGEYQNLMPPTRVRIFRFQSPLYY  565
              GLLVGIVFSMLCVICQTQNPKVS+    T+  +Y  +  Y ++     + I+     +Y
Sbjct  59016  GLLVGIVFSMLCVICQTQNPKVSLF---TNQSIY--LSIYLSIYLSIYLSIYL---SIYL  58861

Query  566    ANKDSFLKSLYKTVGLEPFMEL  587
              +   S   S+Y ++ L  ++ +
Sbjct  58860  SIYLSIYLSIYLSIYLSIYLSI  58795
```

Range 4: 61167 to 61415

Score:174 bits(441), Expect:8e-43,
Method:Compositional matrix adjust.,
Identities:83/83(100%), Positives:83/83(100%), Gaps:0/83(0%)

```
Query  209    QILMAVFRLGFVSVYLSSPMLDGFATGASFTILTVQAKYLLGLKIPRHQGYGTVAVTWFN  268
              QILMAVFRLGFVSVYLSSPMLDGFATGASFTILTVQAKYLLGLKIPRHQGYGTVAVTWFN
Sbjct  61415  QILMAVFRLGFVSVYLSSPMLDGFATGASFTILTVQAKYLLGLKIPRHQGYGTVAVTWFN  61236

Query  269    IFANIHKTNLCDLVTSAICISVL  291
              IFANIHKTNLCDLVTSAICISVL
Sbjct  61235  IFANIHKTNLCDLVTSAICISVL  61167
```

Range 5: 60555 to 60806

Score:162 bits(411), Expect:4e-39,
Method:Compositional matrix adjust.,
Identities:78/84(93%), Positives:81/84(96%), Gaps:0/84(0%)

```
Query  290    VLVAGKEIQERYKDRLKIPLPTELVVVAGATLASHFGDLNSIYGSSVSGHIPTGFIPPKV  349
              +LVAGKEIQERYKDRLKIPLPTELVVVAGATLASHFGDLNSIYGSSVSGHIPTGFIPPKV
Sbjct  60806  LLVAGKEIQERYKDRLKIPLPTELVVVAGATLASHFGDLNSIYGSSVSGHIPTGFIPPKV  60627

Query  350    PSFDLMPRVALDAIPLAVISFAFT  373
              PSFDLMPRVALDAIPLAVI ++
Sbjct  60626  PSFDLMPRVALDAIPLAVIRYSMN  60555
```

Range 6: 66325 to 66573

Score:156 bits(394), Expect:4e-37,
Method:Compositional matrix adjust.,
Identities:78/88(89%), Positives:82/88(93%), Gaps:5/88(5%)

```
Query  137    QVVIREVYMAGFDLDEDPGTAKSTGPDIWNGTEGVNLSSGRLATVEVMGMACNKECYAIS  196
              QVVIREVYMAGFDLDEDPGTAKSTGPDIWNGTEGVNLSSGRLATVEVMGMACNKECYAIS
Sbjct  66573  QVVIREVYMAGFDLDEDPGTAKSTGPDIWNGTEGVNLSSGRLATVEVMGMACNKECYAIS  66394

Query  197    IAAAITFLAGIYQILMAVFRLGFVSVYL  224
              IAAAITFLAGIYQ    V+RL + +VY+
Sbjct  66393  IAAAITFLAGIYQ----VWRLSY-TVYV  66325
```

Range 7: 59507 to 59686

Score:128 bits(322), Expect:3e-28,
Method:Compositional matrix adjust.,
Identities:60/60(100%), Positives:60/60(100%), Gaps:0/60(0%)

```
Query  371    AFTVSLSEMFAKKNGYTVRPNQEMLAIGCCNIIPSFFHCFTTSAALAKTMVKDSTGCQTQ  430
              AFTVSLSEMFAKKNGYTVRPNQEMLAIGCCNIIPSFFHCFTTSAALAKTMVKDSTGCQTQ
Sbjct  59686  AFTVSLSEMFAKKNGYTVRPNQEMLAIGCCNIIPSFFHCFTTSAALAKTMVKDSTGCQTQ  59507
```

AR Minispotted lanternfish *slc26a1*

Query: unnamed protein product Query ID: lcl|Query_3656753 Length: 706

>Gymnoscopelus microlampas genome assembly, contig: atg004017l_1, whole genome shotgun sequence
Sequence ID: CAUOFV010003168.1 Length: 202781
Range 1: 62750 to 63328

Score:353 bits(906), Expect:1e-103,
Method:Compositional matrix adjust.,
Identities:193/193(100%), Positives:193/193(100%), Gaps:0/193(0%)

```
Query  514    KVSLLGRADDTDFYEDMEEYKNLTAPSRVQVFRFQSPLYYANKDSFLKSLYKAVEMDPFl  573
              KVSLLGRADDTDFYEDMEEYKNLTAPSRVQVFRFQSPLYYANKDSFLKSLYKAVEMDPFL
Sbjct  62750  KVSLLGRADDTDFYEDMEEYKNLTAPSRVQVFRFQSPLYYANKDSFLKSLYKAVEMDPFL  62929

Query  574    eltrkrkaekkakelvakQDAAASDKTNGEVSIALVQRELDFHTIVLDCSAIPFMDSAGS  633
              ELTRKRKAEKKAKELVAKQDAAASDKTNGEVSIALVQRELDFHTIVLDCSAIPFMDSAGS
```

```
Sbjct  62930  ELTRKRKAEKKAKELVAKQDAAASDKTNGEVSIALVQRELDFHTIVLDCSAIPFMDSAGS  63109

Query  634    VTFKGLVKDYKEVGVSVLVACCNTSVIDALRRGALFGKDDKDMDSMLFYTIHAAVMHANN  693
              VTFKGLVKDYKEVGVSVLVACCNTSVIDALRRGALFGKDDKDMDSMLFYTIHAAVMHANN
Sbjct  63110  VTFKGLVKDYKEVGVSVLVACCNTSVIDALRRGALFGKDDKDMDSMLFYTIHAAVMHANN  63289

Query  694    RAAVARSTEDSTV  706
              RAAVARSTEDSTV
Sbjct  63290  RAAVARSTEDSTV  63328
```

Range 2: 55064 to 55519

Score:295 bits(756), Expect:5e-84,
Method:Compositional matrix adjust.,
Identities:142/152(93%), Positives:146/152(96%), Gaps:4/152(2%)

```
Query  1      MEDATNKATESTSPPPHPHLERHVRLRHTPLSALRSKVKQGVTCSVPRVRATLTGFFPVV  60
              MEDATNKATESTSPPPHPHLERHVRLRHTPLSALRSKVKQGVTCSVPRVRATLTGFFPVV
Sbjct  55064  MEDATNKATESTSPPPHPHLERHVRLRHTPLSALRSKVKQGVTCSVPRVRATLTGFFPVV  55243

Query  61     RWLPKYKLREYVWGDLMSGLIVGIILVPQAIAYCLLAGVDPIYGLYTSFFANIIYFFMGT  120
              RWLPKYKLREYVWGDLMSGLIVGIILVPQAIAYCLLAGVDPIYGLYTSFFANIIYFFMGT
Sbjct  55244  RWLPKYKLREYVWGDLMSGLIVGIILVPQAIAYCLLAGVDPIYGLYTSFFANIIYFFMGT  55423

Query  121    SRHVSVGIFSLMSLMVGQVVY----REVDLAF  148
              SRHVSVGIFSLMSLMVGQV+     R+++L F
Sbjct  55424  SRHVSVGIFSLMSLMVGQVIVLHGERKLELEF  55519
```

Range 3: 57169 to 57417

Score:175 bits(443), Expect:1e-43,
Method:Compositional matrix adjust.,
Identities:83/83(100%), Positives:83/83(100%), Gaps:0/83(0%)

```
Query  197    QIMMAVFRLGFVSVYLSAPMLDGFATGASFTILTVQAKYLLGLKIPRHQGYGTVIYTWYN  256
              QIMMAVFRLGFVSVYLSAPMLDGFATGASFTILTVQAKYLLGLKIPRHQGYGTVIYTWYN
Sbjct  57169  QIMMAVFRLGFVSVYLSAPMLDGFATGASFTILTVQAKYLLGLKIPRHQGYGTVIYTWYN  57348

Query  257    IFANIHNTNFCDLITSAICIFIL  279
              IFANIHNTNFCDLITSAICIFIL
Sbjct  57349  IFANIHNTNFCDLITSAICIFIL  57417
```

Range 4: 62116 to 62427

Score:169 bits(429), Expect:7e-42,
Method:Compositional matrix adjust.,
Identities:86/104(83%), Positives:90/104(86%), Gaps:4/104(3%)

```
Query  434    FFAPYFYALQKCVLACIIIVSLRGALRKFRDVPSKWRTSKIDAIVWLVTMAASALISVEL  493
              FFAPYFYALQKCVLACIIIVSLRGALRKFRDVPSKWRTSKIDAIVWLVTMAASALISVEL
Sbjct  62116  FFAPYFYALQKCVLACIIIVSLRGALRKFRDVPSKWRTSKIDAIVWLVTMAASALISVEL  62295

Query  494    GLVAGIVFSMLCVIYQTQNAKVSLLG----RADDTDFYEDMEEY  533
              GLVAGIVFSMLCVIYQTQNAKV L+      A    F+  + +Y
Sbjct  62296  GLVAGIVFSMLCVIYQTQNAKVILICFLIRLAVCLSFHVRLSKY  62427
```

Range 5: 57811 to 58071

Score:165 bits(417), Expect:2e-40,
Method:Compositional matrix adjust.,
Identities:79/87(91%), Positives:81/87(93%), Gaps:0/87(0%)

```
Query  277    FILVAGKEIQERYKDRLKIPLPTELVVVAGATLASHFGDLNGQYSSSVSGHIPTGFIAPK  336
                LVAGKEIQERYKDRLKIPLPTELVVVAGATLASHFGDLNGQYSSSVSGHIPTGFIAPK
Sbjct  57811  LFLVAGKEIQERYKDRLKIPLPTELVVVAGATLASHFGDLNGQYSSSVSGHIPTGFIAPK  57990

Query  337    VPDFNLMPRVALDAIPLAVISFAFTVS  363
              VPDFNLMPRVALDAIPLAVI +   +S
Sbjct  57991  VPDFNLMPRVALDAIPLAVIRYGTGMS  58071
```

Range 6: 58937 to 59125

Score:134 bits(336), Expect:2e-30,
Method:Compositional matrix adjust.,
Identities:62/63(98%), Positives:63/63(100%), Gaps:0/63(0%)

```
Query  356    ISFAFTVSLSEMFAKKNGYTVRPNQEMLAIGCCNIIPSFFHCFTTSAALAKTMVKDSTGC  415
              +SFAFTVSLSEMFAKKNGYTVRPNQEMLAIGCCNIIPSFFHCFTTSAALAKTMVKDSTGC
Sbjct  58937  LSFAFTVSLSEMFAKKNGYTVRPNQEMLAIGCCNIIPSFFHCFTTSAALAKTMVKDSTGC  59116

Query  416    QTQ  418
              QTQ
Sbjct  59117  QTQ  59125
```

Range 7: 55884 to 56075

Score:126 bits(317), Expect:5e-28,
Method:Compositional matrix adjust.,
Identities:61/66(92%), Positives:63/66(95%), Gaps:2/66(3%)

```
Query  139    VVYREVDLAFPNEDSSNNAIDPGMLNSTNATDLPGLECGKECYAISVAVAITFLAGIYQI  198
              VVYREVDLAFPNEDSSNNAIDPGMLNSTNATDLPGLECGKECYAISVAVAITFLAGIYQ+
Sbjct  55884  VVYREVDLAFPNEDSSNNAIDPGMLNSTNATDLPGLECGKECYAISVAVAITFLAGIYQV  56063

Query  199    MMAVFR  204
               +  FR
Sbjct  56064  WL--FR  56075
```

AS Bigeye Pacific opah *slc26a1*

Query: unnamed protein product Query ID: lcl|Query_7346801 Length: 714

>Lampris megalopsis isolate JB-2021 Contig2, whole genome shotgun sequence
Sequence ID: JAJHIY020000424.1 Length: 20198553
Range 1: 16054750 to 16055331

Score:401 bits(1030), Expect:6e-120,
Method:Compositional matrix adjust.,
Identities:194/194(100%), Positives:194/194(100%), Gaps:0/194(0%)

```
Query  521       KVSLLGRVNDTTVYEDLEEYENLIPPARVHIFQFQAPLYYANKDSFLKSLYKSVKVEPFL  580
                 KVSLLGRVNDTTVYEDLEEYENLIPPARVHIFQFQAPLYYANKDSFLKSLYKSVKVEPFL
Sbjct  16054750  KVSLLGRVNDTTVYEDLEEYENLIPPARVHIFQFQAPLYYANKDSFLKSLYKSVKVEPFL  16054929

Query  581       ELTKRRKADKIKAKEMKTDKTNGEVVLGLVERELDFHTIVLDCSTIPFIDSTGVATIESL  640
                 ELTKRRKADKIKAKEMKTDKTNGEVVLGLVERELDFHTIVLDCSTIPFIDSTGVATIESL
Sbjct  16054930  ELTKRRKADKIKAKEMKTDKTNGEVVLGLVERELDFHTIVLDCSTIPFIDSTGVATIESL  16055109

Query  641       IKDYKEVGVSILLASCNTSVIDNLRRGSFFRKNDKDIENILFYTVHAAVLHANTRAAAVE  700
                 IKDYKEVGVSILLASCNTSVIDNLRRGSFFRKNDKDIENILFYTVHAAVLHANTRAAAVE
Sbjct  16055110  IKDYKEVGVSILLASCNTSVIDNLRRGSFFRKNDKDIENILFYTVHAAVLHANTRAAAVE  16055289

Query  701       HMMNKEFTVRELKE  714
                 HMMNKEFTVRELKE
Sbjct  16055290  HMMNKEFTVRELKE  16055331
```

Range 2: 16043245 to 16043667

Score:228 bits(580), Expect:6e-61,
Method:Compositional matrix adjust.,
Identities:137/141(97%), Positives:141/141(100%), Gaps:0/141(0%)

```
Query  1         MDAGTKDIETppplplilerrirkrrpplsilrsrVKQEVACSTPRVRSLFSGFFPVVRW  60
                 MDAGTKDIETPPPLPLILERRIRKRRPPLSILRSRVKQEVACSTPRVRSLFSGFFPVVRW
Sbjct  16043245  MDAGTKDIETPPPLPLILERRIRKRRPPLSILRSRVKQEVACSTPRVRSLFSGFFPVVRW  16043424

Query  61        LPKYKLQQYVWGDVMSGLIVGIILVPQAIAYCLLAGVEPIYGLYTSFFANIIYFFMGTSK  120
                 LPKYKLQQYVWGDVMSGLIVGIILVPQAIAYCLLAGVEPIYGLYTSFFANIIYFFMGTSK
Sbjct  16043425  LPKYKLQQYVWGDVMSGLIVGIILVPQAIAYCLLAGVEPIYGLYTSFFANIIYFFMGTSK  16043604

Query  121       HVSVGIFSLMSLMIGQVVDRE  141
                 HVSVGIFSLMSLMIGQV++++
Sbjct  16043605  HVSVGIFSLMSLMIGQVMNQQ  16043667
```

Range 3: 16045688 to 16045936

Score:169 bits(427), Expect:2e-41,
Method:Compositional matrix adjust.,
Identities:82/83(99%), Positives:83/83(100%), Gaps:0/83(0%)

```
Query  204       QVMMAIFRLGFVSVYLSAPMLDGFATGASFTILTVQAKYLLGLKIPRHQGYGTVVVTWIN  263
                 +VMMAIFRLGFVSVYLSAPMLDGFATGASFTILTVQAKYLLGLKIPRHQGYGTVVVTWIN
Sbjct  16045688  KVMMAIFRLGFVSVYLSAPMLDGFATGASFTILTVQAKYLLGLKIPRHQGYGTVVVTWIN  16045867

Query  264       ILSSIHKTNICDLITSAICITIL  286
                 ILSSIHKTNICDLITSAICITIL
Sbjct  16045868  ILSSIHKTNICDLITSAICITIL  16045936
```

Range 4: 16048246 to 16048497

Score:162 bits(410), Expect:2e-39,
Method:Compositional matrix adjust.,
Identities:78/84(93%), Positives:80/84(95%), Gaps:0/84(0%)

```
Query  285       ILVAGKEIQDRYKARLKIPLPTELIVVAGATLISHFGDLHSHYESSVSGHIPTGFMPPTV  344
                 I VAGKEIQDRYKARLKIPLPTELIVVAGATLISHFGDLHSHYESSVSGHIPTGFMPPTV
Sbjct  16048246  IKVAGKEIQDRYKARLKIPLPTELIVVAGATLISHFGDLHSHYESSVSGHIPTGFMPPTV  16048425

Query  345       PSFGLMPRVALDAIPLAIISFAFT  368
                 PSFGLMPRVALDAIPLAII +  +
Sbjct  16048426  PSFGLMPRVALDAIPLAIIRYCIS  16048497
```

Range 5: 16053539 to 16053760

Score:151 bits(382), Expect:6e-36,
Method:Compositional matrix adjust.,
Identities:74/74(100%), Positives:74/74(100%), Gaps:0/74(0%)

```
Query  450       QKCVLASIIIVSLRGALRKFRDVPAKWHTSRIDAIVWMVTMAATALISVELGLLVGVFFS  509
                 QKCVLASIIIVSLRGALRKFRDVPAKWHTSRIDAIVWMVTMAATALISVELGLLVGVFFS
Sbjct  16053539  QKCVLASIIIVSLRGALRKFRDVPAKWHTSRIDAIVWMVTMAATALISVELGLLVGVFFS  16053718

Query  510       MLCIVFLTQKPKVS  523
                 MLCIVFLTQKPKVS
Sbjct  16053719  MLCIVFLTQKPKVS  16053760
```

Range 6: 16044264 to 16044530

Score:145 bits(366), Expect:6e-34,
Method:Compositional matrix adjust.,
Identities:74/89(83%), Positives:77/89(86%), Gaps:2/89(2%)

```
Query  119       SKHVSVGIFSLMSLMIG--QVVDREVYLAGFDLNEDSGISANSLDILNGTQGTNLSATEM  176
                 S+ + +   SLM       QVVDREVYLAGFDLNEDSGISANSLDILNGTQGTNLSATEM
Sbjct  16044264  SECIKITFLSLMGFFFHCLQVVDREVYLAGFDLNEDSGISANSLDILNGTQGTNLSATEM  16044443

Query  177       GSMGIECGKECYAISIAAAVTFLAGIYQV  205
                 GSMGIECGKECYAISIAAAVTFLAGIYQV
Sbjct  16044444  GSMGIECGKECYAISIAAAVTFLAGIYQV  16044530
```

Range 7: 16049689 to 16049886

Score:134 bits(336), Expect:3e-30,
Method:Compositional matrix adjust.,
Identities:63/66(95%), Positives:64/66(96%), Gaps:0/66(0%)

```
Query  360       LAIISFAFTVSLSEMFAKKNGYTVRPNQEMLAIGFCNIIPSFFHCFTTSAALAKTMVKES  419
                 L + SFAFTVSLSEMFAKKNGYTVRPNQEMLAIGFCNIIPSFFHCFTTSAALAKTMVKES
Sbjct  16049689  LFLFSFAFTVSLSEMFAKKNGYTVRPNQEMLAIGFCNIIPSFFHCFTTSAALAKTMVKES  16049868

Query  420       TGCQTQ  425
                 TGCQTQ
Sbjct  16049869  TGCQTQ  16049886
```

AT Pinecone soldierfish *slc26a1*

Query: sulfate anion transporter 1-like [Myripristis murdjan] Query ID: XP_029920955.1 Length: 713

>Myripristis murdjan chromosome 12, fMyrMur1.1, whole genome shotgun sequence
Sequence ID: NC_043991.1 Length: 32414123
Range 1: 1823572 to 1824147

Score:706 bits(1440), Expect:0.0,
Method:,
Identities:192/192(100%), Positives:192/192(100%), Gaps:0/192(0%)

```
Query  522      VSLLGRVSDSDLYEDMEEYKNLTPPSRVHVFRFQSPLYYANKDSFLKSLYRAVGVEPFLE  581
                VSLLGRVSDSDLYEDMEEYKNLTPPSRVHVFRFQSPLYYANKDSFLKSLYRAVGVEPFLE
Sbjct  1823572  VSLLGRVSDSDLYEDMEEYKNLTPPSRVHVFRFQSPLYYANKDSFLKSLYRAVGVEPFLE  1823751

Query  582      LTRRKKAEKARDMAAKQANAAEHKNNGKVSTGLVHRELDFHTIVLDCSAIPFIDTTGMAT  641
                LTRRKKAEKARDMAAKQANAAEHKNNGKVSTGLVHRELDFHTIVLDCSAIPFIDTTGMAT
Sbjct  1823752  LTRRKKAEKARDMAAKQANAAEHKNNGKVSTGLVHRELDFHTIVLDCSAIPFIDTTGMAT  1823931

Query  642      FKGLVKDYKEIGVSVLLANCNTSVIDALKKGAFFGKNDRDMASLLFYTVHAAVLHANGRA  701
                FKGLVKDYKEIGVSVLLANCNTSVIDALKKGAFFGKNDRDMASLLFYTVHAAVLHANGRA
Sbjct  1823932  FKGLVKDYKEIGVSVLLANCNTSVIDALKKGAFFGKNDRDMASLLFYTVHAAVLHANGRA  1824111

Query  702      AAADCMSDDTMV  713
                AAADCMSDDTMV
```

```
Sbjct  1824112  AAADCMSDDTMV  1824147
```

Range 2: 1816280 to 1816696

Score:516 bits(1053), Expect:3e-150,
Method:,
Identities:137/139(99%), Positives:139/139(100%), Gaps:0/139(0%)

```
Query  1        MEEASKVTETTPALPPLLERRVRQRQPQMSVLKSKLKQRAACSVPCVRATLSGFFPVVRW  60
                MEEASKVTETTPALPPLLERRVRQRQPQMSVLKSKLKQRAACSVPCVRATLSGFFPVVRW
Sbjct  1816280  MEEASKVTETTPALPPLLERRVRQRQPQMSVLKSKLKQRAACSVPCVRATLSGFFPVVRW  1816459

Query  61       LPKYKLQEYVWGDLMSGLIVGIILVPQAIAYCLLAGVEPIYGLYTSFFANIIYFFMGTSR  120
                LPKYKLQEYVWGDLMSGLIVGIILVPQAIAYCLLAGVEPIYGLYTSFFANIIYFFMGTSR
Sbjct  1816460  LPKYKLQEYVWGDLMSGLIVGIILVPQAIAYCLLAGVEPIYGLYTSFFANIIYFFMGTSR  1816639

Query  121      HVSVGIFSLMSLMVGQVVN  139
                HVSVGIFSLMSLMVGQV++
Sbjct  1816640  HVSVGIFSLMSLMVGQVID  1816696
```

Range 3: 1820441 to 1820668

Score:278 bits(565), Expect:1e-142,
Method:,
Identities:76/76(100%), Positives:76/76(100%), Gaps:0/76(0%)

```
Query  288      AGKEIQERYKDRLKIPIPTELIVVAGATLASHFGDLNGHYGSSVSGHIPTGFVMPKVPSF  347
                AGKEIQERYKDRLKIPIPTELIVVAGATLASHFGDLNGHYGSSVSGHIPTGFVMPKVPSF
Sbjct  1820441  AGKEIQERYKDRLKIPIPTELIVVAGATLASHFGDLNGHYGSSVSGHIPTGFVMPKVPSF  1820620

Query  348      DLMPRVALDAIPLAVI  363
                DLMPRVALDAIPLAVI
Sbjct  1820621  DLMPRVALDAIPLAVI  1820668
```

Range 4: 1820762 to 1820950

Score:237 bits(482), Expect:1e-142,
Method:,
Identities:63/63(100%), Positives:63/63(100%), Gaps:0/63(0%)

```
Query  364      SFAFTVSLSEMFAKKNGYTVRPNQEMLAIGFCNIIPSFFYCFTTSAALAKTMVKDSTGCQ  423
                SFAFTVSLSEMFAKKNGYTVRPNQEMLAIGFCNIIPSFFYCFTTSAALAKTMVKDSTGCQ
Sbjct  1820762  SFAFTVSLSEMFAKKNGYTVRPNQEMLAIGFCNIIPSFFYCFTTSAALAKTMVKDSTGCQ  1820941

Query  424      TQV  426
                TQV
Sbjct  1820942  TQV  1820950
```

Range 5: 1821614 to 1821907

Score:360 bits(734), Expect:3e-103,
Method:,
Identities:98/98(100%), Positives:98/98(100%), Gaps:0/98(0%)

```
Query  425      QVSSLVSALVVLLVLLFFAPFFYALQKCVLACIIIVSLRGALRKFRDVPAKWRASRNDAI  484
                QVSSLVSALVVLLVLLFFAPFFYALQKCVLACIIIVSLRGALRKFRDVPAKWRASRNDAI
Sbjct  1821614  QVSSLVSALVVLLVLLFFAPFFYALQKCVLACIIIVSLRGALRKFRDVPAKWRASRNDAI  1821793

Query  485      VWLVAMAATALISVELGLLVGIVFSMLCIIFKTQNPKV  522
                VWLVAMAATALISVELGLLVGIVFSMLCIIFKTQNPKV
Sbjct  1821794  VWLVAMAATALISVELGLLVGIVFSMLCIIFKTQNPKV  1821907
```

Range 6: 1819131 to 1819379

Score:310 bits(631), Expect:5e-88,
Method:,
Identities:83/83(100%), Positives:83/83(100%), Gaps:0/83(0%)

```
Query  204      QVMMAVFRLGFVSVYLSGPMLDGFATGASFTILTVQAKYLLGLKIPRHQGYGTVIVTWIN  263
                QVMMAVFRLGFVSVYLSGPMLDGFATGASFTILTVQAKYLLGLKIPRHQGYGTVIVTWIN
Sbjct  1819131  QVMMAVFRLGFVSVYLSGPMLDGFATGASFTILTVQAKYLLGLKIPRHQGYGTVIVTWIN  1819310

Query  264      IFSNIHKTNVCDLITSAICISTL  286
                IFSNIHKTNVCDLITSAICISTL
Sbjct  1819311  IFSNIHKTNVCDLITSAICISTL  1819379
```

Range 7: 1818301 to 1818510

Score:252 bits(512), Expect:2e-70,
Method:,

Identities:70/70(100%), Positives:70/70(100%), Gaps:0/70(0%)

```
Query  136      QVVNREVYLAGFDPNEDSGTKLSGPGMMNGTGGANLSAGSVELLGLQCGKECYAISIAAA  195
                QVVNREVYLAGFDPNEDSGTKLSGPGMMNGTGGANLSAGSVELLGLQCGKECYAISIAAA
Sbjct  1818301  QVVNREVYLAGFDPNEDSGTKLSGPGMMNGTGGANLSAGSVELLGLQCGKECYAISIAAA  1818480

Query  196      LTFLAGIYQV  205
                LTFLAGIYQV
Sbjct  1818481  LTFLAGIYQV  1818510
```

AU Yellowfin tuna *slc26a1*

Query: sulfate anion transporter 1 [Thunnus albacares] Query ID: XP_044190032.1 Length: 713

>Thunnus albacares chromosome 18, fThuAlb1.1, whole genome shotgun sequence
Sequence ID: NC_058123.1 Length: 30510322
Range 1: 20163698 to 20164279

Score:716 bits(1461), Expect:0.0,
Method:,
Identities:194/194(100%), Positives:194/194(100%), Gaps:0/194(0%)

```
Query  520       KVSLLGRANDTDLYEDMEEYKNLMPPPRVQVFRFQAPLYYANKESFLKSLYKTVGVEPFL  579
                 KVSLLGRANDTDLYEDMEEYKNLMPPPRVQVFRFQAPLYYANKESFLKSLYKTVGVEPFL
Sbjct  20163698  KVSLLGRANDTDLYEDMEEYKNLMPPPRVQVFRFQAPLYYANKESFLKSLYKTVGVEPFL  20163877

Query  580       EMTKRRKAEKKAKEMSAQQAKANGDKTNGEVVVGLVPRELDFHTIVLDCSAIPFIDSTGM  639
                 EMTKRRKAEKKAKEMSAQQAKANGDKTNGEVVVGLVPRELDFHTIVLDCSAIPFIDSTGM
Sbjct  20163878  EMTKRRKAEKKAKEMSAQQAKANGDKTNGEVVVGLVPRELDFHTIVLDCSAIPFIDSTGM  20164057

Query  640       ATFKGLVNEYKEIGVSVLFASCNTSVIDTLQKGQFFGKNDKDMSSLLFHTVHAAVLHANS  699
                 ATFKGLVNEYKEIGVSVLFASCNTSVIDTLQKGQFFGKNDKDMSSLLFHTVHAAVLHANS
Sbjct  20164058  ATFKGLVNEYKEIGVSVLFASCNTSVIDTLQKGQFFGKNDKDMSSLLFHTVHAAVLHANS  20164237

Query  700       MPSAAESKSEDSMV  713
                 MPSAAESKSEDSMV
Sbjct  20164238  MPSAAESKSEDSMV  20164279
```

Range 2: 20159230 to 20159640

Score:505 bits(1030), Expect:1e-146,
Method:,
Identities:135/137(99%), Positives:136/137(99%), Gaps:0/137(0%)

```
Query  1         MEEVTKVTETTSQPLLERRVRQRQPTVSVLKSKLKQSVTCSVPRVRSTLSGFFPVVRWLP  60
                 MEEVTKVTETTSQPLLERRVRQRQPTVSVLKSKLKQSVTCSVPRVRSTLSGFFPVVRWLP
Sbjct  20159230  MEEVTKVTETTSQPLLERRVRQRQPTVSVLKSKLKQSVTCSVPRVRSTLSGFFPVVRWLP  20159409

Query  61        KYKLREYVWGDVMSGVIVGIILVPQAIAYCLLAGVEPIYGLYTSFYANIIYFIMGTSRHV  120
                 KYKLREYVWGDVMSGVIVGIILVPQAIAYCLLAGVEPIYGLYTSFYANIIYFIMGTSRHV
Sbjct  20159410  KYKLREYVWGDVMSGVIVGIILVPQAIAYCLLAGVEPIYGLYTSFYANIIYFIMGTSRHV  20159589

Query  121       SVGIFSLMSLMVGQVVD  137
                 SVGIFSLMSLMVGQV +
Sbjct  20159590  SVGIFSLMSLMVGQVMN  20159640
```

Range 3: 20163129 to 20163425

Score:366 bits(745), Expect:1e-104,
Method:,
Identities:99/99(100%), Positives:99/99(100%), Gaps:0/99(0%)

```
Query  424       QVSSLISALVVLLVLLFFAPFFHALQKCVLACIIIVSLRGALRKFMDIPAKWRASRNDAI  483
                 QVSSLISALVVLLVLLFFAPFFHALQKCVLACIIIVSLRGALRKFMDIPAKWRASRNDAI
Sbjct  20163129  QVSSLISALVVLLVLLFFAPFFHALQKCVLACIIIVSLRGALRKFMDIPAKWRASRNDAI  20163308

Query  484       VWLVAMSATALISVELGLLVGIVFSMICIIFKTQNPKVS  522
                 VWLVAMSATALISVELGLLVGIVFSMICIIFKTQNPKVS
Sbjct  20163309  VWLVAMSATALISVELGLLVGIVFSMICIIFKTQNPKVS  20163425
```

Range 4: 20160979 to 20161227

Score:310 bits(632), Expect:7e-88,
Method:,
Identities:83/83(100%), Positives:83/83(100%), Gaps:0/83(0%)

```
Query  203       QVMMALFQLGFVSVYLSSPMLDGFATGASFTILTVQAKYLLGLKIPRHQGYGTVVVTWFN  262
                 QVMMALFQLGFVSVYLSSPMLDGFATGASFTILTVQAKYLLGLKIPRHQGYGTVVVTWFN
Sbjct  20160979  QVMMALFQLGFVSVYLSSPMLDGFATGASFTILTVQAKYLLGLKIPRHQGYGTVVVTWFN  20161158

Query  263       IFANIQNTNLCDLITSTICISVL  285
```

```
                IFANIQNTNLCDLITSTICISVL
Sbjct  20161159  IFANIQNTNLCDLITSTICISVL  20161227
```

Range 5: 20161678 to 20161914

Score:281 bits(572), Expect:5e-79,
Method:,
Identities:77/79(97%), Positives:78/79(98%), Gaps:0/79(0%)

```
Query  284       VLVAGKEIQERYKDRLKIPLPTELIVVAGSTLASHYGMLNSRYGSSVSGHIPTGFIPPQV  343
                 + VAGKEIQERYKDRLKIPLPTELIVVAGSTLASHYGMLNSRYGSSVSGHIPTGFIPPQV
Sbjct  20161678  IIVAGKEIQERYKDRLKIPLPTELIVVAGSTLASHYGMLNSRYGSSVSGHIPTGFIPPQV  20161857

Query  344       PNFSLMSRVALDAIPLAVI  362
                 PNFSLMSRVALDAIPLAVI
Sbjct  20161858  PNFSLMSRVALDAIPLAVI  20161914
```

Range 6: 20159932 to 20160144

Score:256 bits(521), Expect:2e-71,
Method:,
Identities:71/71(100%), Positives:71/71(100%), Gaps:0/71(0%)

```
Query  134       QVVDREVFLAGFDLSEDVSASGPDVLNGSLGINLTEGKLHSVELMGLQCGKECYAISIAA  193
                 QVVDREVFLAGFDLSEDVSASGPDVLNGSLGINLTEGKLHSVELMGLQCGKECYAISIAA
Sbjct  20159932  QVVDREVFLAGFDLSEDVSASGPDVLNGSLGINLTEGKLHSVELMGLQCGKECYAISIAA  20160111

Query  194       AITFLAGIYQV  204
                 AITFLAGIYQV
Sbjct  20160112  AITFLAGIYQV  20160144
```

Range 7: 20162340 to 20162528

Score:237 bits(481), Expect:1e-65,
Method:,
Identities:63/63(100%), Positives:63/63(100%), Gaps:0/63(0%)

```
Query  363       SFAFTVSLSEMFAKKNGYTVRPNQEMLAIGFCNIIPSFFHCFTTSAALAKTMVKDSTGCQ  422
                 SFAFTVSLSEMFAKKNGYTVRPNQEMLAIGFCNIIPSFFHCFTTSAALAKTMVKDSTGCQ
Sbjct  20162340  SFAFTVSLSEMFAKKNGYTVRPNQEMLAIGFCNIIPSFFHCFTTSAALAKTMVKDSTGCQ  20162519

Query  423       TQV  425
                 TQV
Sbjct  20162520  TQV  20162528
```

AV Mandarinfish *slc26a1*

Query: sulfate anion transporter 1 [Synchiropus splendidus] Query ID: XP_053741897.1 Length: 699

>Synchiropus splendidus isolate RoL2022-P1 chromosome 1, RoL_Sspl_1.0, whole genome shotgun sequence
Sequence ID: NC_071334.1 Length: 71620908
Range 1: 59720837 to 59721388

Score:670 bits(1368), Expect:0.0,
Method:,
Identities:184/184(100%), Positives:184/184(100%), Gaps:0/184(0%)

```
Query  516       VSLLGQADDTELYEDLEEYANLTAPPRIKIVRFQAPLYYANKESFLKSLYKTVGVEPFLE  575
                 VSLLGQADDTELYEDLEEYANLTAPPRIKIVRFQAPLYYANKESFLKSLYKTVGVEPFLE
Sbjct  59721388  VSLLGQADDTELYEDLEEYANLTAPPRIKIVRFQAPLYYANKESFLKSLYKTVGVEPFLE  59721209

Query  576       LTARRKADKVAKEMSVNGGGNGELGVGLVNRELDFHTIVLDCSAMPFIDSTGVATFNGLV  635
                 LTARRKADKVAKEMSVNGGGNGELGVGLVNRELDFHTIVLDCSAMPFIDSTGVATFNGLV
Sbjct  59721208  LTARRKADKVAKEMSVNGGGNGELGVGLVNRELDFHTIVLDCSAMPFIDSTGVATFNGLV  59721029

Query  636       KEYREIGIQVLLACCNTSLVDSLQKGQVFGKTDSDMSSMLFHSVHAATVHAGLSGTHRTD  695
                 KEYREIGIQVLLACCNTSLVDSLQKGQVFGKTDSDMSSMLFHSVHAATVHAGLSGTHRTD
Sbjct  59721028  KEYREIGIQVLLACCNTSLVDSLQKGQVFGKTDSDMSSMLFHSVHAATVHAGLSGTHRTD  59720849

Query  696       DSLV  699
                 DSLV
Sbjct  59720848  DSLV  59720837
```

Range 2: 59722117 to 59722413

Score:371 bits(755), Expect:8e-170,
Method:,
Identities:99/99(100%), Positives:99/99(100%), Gaps:0/99(0%)

```
Query  419       QVSSVISALVVLLVLLFFAPYFHDLQKCVLACIIIVSLRGALRKYKNIPAMWRTNRNDAI  478
                 QVSSVISALVVLLVLLFFAPYFHDLQKCVLACIIIVSLRGALRKYKNIPAMWRTNRNDAI
Sbjct  59722413  QVSSVISALVVLLVLLFFAPYFHDLQKCVLACIIIVSLRGALRKYKNIPAMWRTNRNDAI  59722234

Query  479       VWLVAMSATALISIELGLLVGVVFAMMCVIYKTQNPKVS  517
                 VWLVAMSATALISIELGLLVGVVFAMMCVIYKTQNPKVS
Sbjct  59722233  VWLVAMSATALISIELGLLVGVVFAMMCVIYKTQNPKVS  59722117
```

Range 3: 59722475 to 59722663

Score:235 bits(477), Expect:8e-170,
Method:,
Identities:63/63(100%), Positives:63/63(100%), Gaps:0/63(0%)

```
Query  358       SFAFTVSLSEMFAKKNGYTVRPNQEMLAIGFCNIIPSFFHSFTTSAALAKTMVKDSTGCQ  417
                 SFAFTVSLSEMFAKKNGYTVRPNQEMLAIGFCNIIPSFFHSFTTSAALAKTMVKDSTGCQ
Sbjct  59722663  SFAFTVSLSEMFAKKNGYTVRPNQEMLAIGFCNIIPSFFHSFTTSAALAKTMVKDSTGCQ  59722484

Query  418       TQV  420
                 TQV
Sbjct  59722483  TQV  59722475
```

Range 4: 59723256 to 59723504

Score:308 bits(627), Expect:5e-165,
Method:,
Identities:83/83(100%), Positives:83/83(100%), Gaps:0/83(0%)

```
Query  198       QVLMAVFRLGFVSVYLSAPMLDGFATGASFTILTVQAKYLLGLKIPRHQGYGTVVVTWIN  257
                 QVLMAVFRLGFVSVYLSAPMLDGFATGASFTILTVQAKYLLGLKIPRHQGYGTVVVTWIN
Sbjct  59723504  QVLMAVFRLGFVSVYLSAPMLDGFATGASFTILTVQAKYLLGLKIPRHQGYGTVVVTWIN  59723325

Query  258       IFSNIHKTNVCDLITSAICISIL  280
                 IFSNIHKTNVCDLITSAICISIL
Sbjct  59723324  IFSNIHKTNVCDLITSAICISIL  59723256
```

Range 5: 59722944 to 59723174

Score:281 bits(572), Expect:5e-165,
Method:,
Identities:77/77(100%), Positives:77/77(100%), Gaps:0/77(0%)

```
Query  281       VAGKEIQERFKDRLKIPLPTELVVVAGATLASHYGELNSHYGTAVSGHIPTGFIPPQVPS  340
                 VAGKEIQERFKDRLKIPLPTELVVVAGATLASHYGELNSHYGTAVSGHIPTGFIPPQVPS
Sbjct  59723174  VAGKEIQERFKDRLKIPLPTELVVVAGATLASHYGELNSHYGTAVSGHIPTGFIPPQVPS  59722995

Query  341       FGLMPRVAMDAIPLAVI  357
                 FGLMPRVAMDAIPLAVI
Sbjct  59722994  FGLMPRVAMDAIPLAVI  59722944
```

Range 6: 59725167 to 59725577

Score:512 bits(1045), Expect:5e-149,
Method:,
Identities:137/137(100%), Positives:137/137(100%), Gaps:0/137(0%)

```
Query  1         MADHSKETEPTPSPPQLLERRLRQRQSTASVLKKKLKQSATCSIPRVRSTLIGFFPVVQW  60
                 MADHSKETEPTPSPPQLLERRLRQRQSTASVLKKKLKQSATCSIPRVRSTLIGFFPVVQW
Sbjct  59725577  MADHSKETEPTPSPPQLLERRLRQRQSTASVLKKKLKQSATCSIPRVRSTLIGFFPVVQW  59725398

Query  61        LPKYKLKEYVWGDVMSGLIIGIILVPQAIAYCLLAGVDPIYGLYTSFYANIIYFLMGTSR  120
                 LPKYKLKEYVWGDVMSGLIIGIILVPQAIAYCLLAGVDPIYGLYTSFYANIIYFLMGTSR
Sbjct  59725397  LPKYKLKEYVWGDVMSGLIIGIILVPQAIAYCLLAGVDPIYGLYTSFYANIIYFLMGTSR  59725218

Query  121       HVSVGIFSLMSLMVGQV  137
                 HVSVGIFSLMSLMVGQV
Sbjct  59725217  HVSVGIFSLMSLMVGQV  59725167
```

Range 7: 59723932 to 59724129

Score:238 bits(484), Expect:3e-66,
Method:,
Identities:65/66(98%), Positives:65/66(98%), Gaps:0/66(0%)

```
Query  136       QVVDREVFLAGFDLNDESAAFNSSLNLSNAAGQTVELMGMQCDKECYAICIAAALTFLAG  195
                 QVVDREVFLAGFDLNDESAAFNSSLNLSNAAGQTVELMGMQCDKECYAICIAAALTFLAG
Sbjct  59724129  QVVDREVFLAGFDLNDESAAFNSSLNLSNAAGQTVELMGMQCDKECYAICIAAALTFLAG  59723950

Query  196       VYQVLM  201
                 VYQV M
Sbjct  59723949  VYQVNM  59723932
```

AW Straightnose pipefish *slc26a1*

Query: sulfate anion transporter 1 [Nerophis ophidion] Query ID: XP_061732948.1 Length: 696

>Nerophis ophidion isolate RoL-2023_Sa linkage group LG17, whole genome shotgun sequence
Sequence ID: JAWNBE010000017.1 Length: 53015709
Range 1: 40895450 to 40896004

Score:360 bits(925), Expect:5e-106,
Method:Compositional matrix adjust.,
Identities:183/185(99%), Positives:184/185(99%), Gaps:0/185(0%)

```
Query  512       KPKVSLLGRANDSELYEDVEEYQNLVPPLRIRIFRFQAPLYYANKDVFLASLYKAVGVTP  571
                 K +VSLLGRANDSELYEDVEEYQNLVPPLRIRIFRFQAPLYYANKDVFLASLYKAVGVTP
Sbjct  40896004  KLQVSLLGRANDSELYEDVEEYQNLVPPLRIRIFRFQAPLYYANKDVFLASLYKAVGVTP  40895825

Query  572       FLektrrrkaekkakkMSTTQANGDTTNGEVVVGLVRREPDFHTIILDCSAISFIDSAGM  631
                 FLEKTRRRKAEKKAKKMSTTQANGDTTNGEVVVGLVRREPDFHTIILDCSAISFIDSAGM
Sbjct  40895824  FLEKTRRRKAEKKAKKMSTTQANGDTTNGEVVVGLVRREPDFHTIILDCSAISFIDSAGM  40895645

Query  632       AAFKGLLQEFDQIGVRVVLAGCNTSLVDALQKGQFFGKEMRNMLFHSVHHAVLQTNSMAA  691
                 AAFKGLLQEFDQIGVRVVLAGCNTSLVDALQKGQFFGKEMRNMLFHSVHHAVLQTNSMAA
Sbjct  40895644  AAFKGLLQEFDQIGVRVVLAGCNTSLVDALQKGQFFGKEMRNMLFHSVHHAVLQTNSMAA  40895465

Query  692       DDNVV  696
                 DDNVV
Sbjct  40895464  DDNVV  40895450
```

Range 2: 40907706 to 40908203

Score:275 bits(702), Expect:1e-76,
Method:Compositional matrix adjust.,
Identities:142/169(84%), Positives:149/169(88%), Gaps:5/169(2%)

```
Query  1         MEEVIKLKESPPTLLERRVRHRQLALSVLKSKLKQGVSCSVPRVRSTLSGFFPVVRWLPK  60
                 MEEVIKLKESPPTLLERRVRHRQLALSVLKSKLKQGVSCSVPRVRSTLSGFFPVVRWLPK
Sbjct  40908203  MEEVIKLKESPPTLLERRVRHRQLALSVLKSKLKQGVSCSVPRVRSTLSGFFPVVRWLPK  40908024

Query  61        YKLREYVWGDVMSGLIVGIILVPQAIAYCLLAGVEPIYGLYTSFYANIIYFLMGTSRHVS  120
                 YKLREYVWGDVMSGLIVGIILVPQAIAYCLLAGVEPIYGLYTSFYANIIYFLMGTSRHVS
Sbjct  40908023  YKLREYVWGDVMSGLIVGIILVPQAIAYCLLAGVEPIYGLYTSFYANIIYFLMGTSRHVS  40907844

Query  121       VGIFSLMSLMVGQVVDKEVFLAGFDLNEDLLGFN--ASLRTNFTGHDSH  167
                 VGIFSLMSLMVGQV +    ++ F L+  L  F   A L+ N + +  H
Sbjct  40907843  VGIFSLMSLMVGQVSN---CISWFFLSFFLSSFRT*AHLQHNTSENSRH  40907706
```

Range 3: 40899817 to 40900089

Score:178 bits(451), Expect:2e-76,
Method:Compositional matrix adjust.,
Identities:86/91(95%), Positives:88/91(96%), Gaps:0/91(0%)

```
Query  434       FFAPYFHALQKCVLACIIIVSLRGALRKFKDVPSKWRASKNDAVVWLVTVSATALISVEL  493
                 FFAPYFHALQKCVLACIIIVSLRGALRKFKDVPSKWRASKNDAVVWLVTVSATALISVEL
Sbjct  40900089  FFAPYFHALQKCVLACIIIVSLRGALRKFKDVPSKWRASKNDAVVWLVTVSATALISVEL  40899910

Query  494       GLLVGITFSMMCIIYKTQKPKVSLLGRANDS  524
                 GLLVGITFSMMCIIYKTQKPKVSL  + +DS
Sbjct  40899909  GLLVGITFSMMCIIYKTQKPKVSLSHQQDDS  40899817
```

Range 4: 40900203 to 40900406

Score:134 bits(337), Expect:2e-76,
Method:Compositional matrix adjust.,
Identities:64/68(94%), Positives:64/68(94%), Gaps:0/68(0%)

```
Query  351       IPLAVISFAFTVSLSEMFAKKNGYTVRPNQEMLAIGCCNIIPSFFHCFTTSAALAKTMVK  410
                 I L   SFAFTVSLSEMFAKKNGYTVRPNQEMLAIGCCNIIPSFFHCFTTSAALAKTMVK
Sbjct  40900406  IDLLFFSFAFTVSLSEMFAKKNGYTVRPNQEMLAIGCCNIIPSFFHCFTTSAALAKTMVK  40900227

Query  411       DSTGCQTQ  418
                 DSTGCQTQ
Sbjct  40900226  DSTGCQTQ  40900203
```

Range 5: 40901834 to 40902082

Score:170 bits(431), Expect:8e-42,
Method:Compositional matrix adjust.,
Identities:83/83(100%), Positives:83/83(100%), Gaps:0/83(0%)

```
Query  197       QVLMAVFGLGFVSVYLSTPMLDGFATGASFTILTVQAKYLLGLKIPRHQGYGTVLVTWFN  256
                 QVLMAVFGLGFVSVYLSTPMLDGFATGASFTILTVQAKYLLGLKIPRHQGYGTVLVTWFN
Sbjct  40902082  QVLMAVFGLGFVSVYLSTPMLDGFATGASFTILTVQAKYLLGLKIPRHQGYGTVLVTWFN  40901903

Query  257       IFANIHKTNVCDLVTSAICIAIL  279
                 IFANIHKTNVCDLVTSAICIAIL
Sbjct  40901902  IFANIHKTNVCDLVTSAICIAIL  40901834
```

Range 6: 40901533 to 40901814

Score:164 bits(415), Expect:8e-40,
Method:Compositional matrix adjust.,
Identities:83/94(88%), Positives:86/94(91%), Gaps:4/94(4%)

```
Query  267       CDLVT---SAICI-AILVTGKEIQERYKDRLKIPLPTELVVVGGATLASHFGNLNHLYGS  322
                 C  VT   + +C+ A LVTGKEIQERYKDRLKIPLPTELVVVGGATLASHFGNLNHLYGS
Sbjct  40901814  CSPVTQEVTCLCVWAPLVTGKEIQERYKDRLKIPLPTELVVVGGATLASHFGNLNHLYGS  40901635

Query  323       SVSGHIPTGFIPPQVPNFGLMPRVALDAIPLAVI  356
                 SVSGHIPTGFIPPQVPNFGLMPRVALDAIPLAVI
Sbjct  40901634  SVSGHIPTGFIPPQVPNFGLMPRVALDAIPLAVI  40901533
```

Range 7: 40905204 to 40905476

Score:139 bits(350), Expect:1e-31,
Method:Compositional matrix adjust.,
Identities:71/94(76%), Positives:76/94(80%), Gaps:3/94(3%)

```
Query  105       YANIIYFLMGTSRHVSVGIFSLMSLMVGQVVDKEVFLAGFDLNEDLLGFNASLRTNFTGH  164
                 + N++Y  +  S    +  F    L   QVVDKEVFLAGFDLNEDLLGFNASLRTNFTGH
Sbjct  40905476  FTNVVYIKIKQSWFCRIW*FMTACL---QVVDKEVFLAGFDLNEDLLGFNASLRTNFTGH  40905306

Query  165       DSHSVELLGVQCGKECYAISIAAALTFLAGIYQV  198
                 DSHSVELLGVQCGKECYAISIAAALTFLAGIYQV
Sbjct  40905305  DSHSVELLGVQCGKECYAISIAAALTFLAGIYQV  40905204
```

AX Broad-nosed pipefish *slc26a1*

Query: sulfate anion transporter 1 [Syngnathus typhle] Query ID: XP_061150241.1 Length: 698

>Syngnathus typhle isolate RoL2023-S1 linkage group LG12, whole genome shotgun sequence
Sequence ID: JAWIVE010000012.1 Length: 13708153
Range 1: 3282906 to 3283403

Score:277 bits(709), Expect:0.0,
Method:Compositional matrix adjust.,
Identities:139/166(84%), Positives:139/166(83%), Gaps:27/166(16%)

```
Query  275      VAGKEIQERYKDRLKIPLPTELVVVAGATLASHFGDLNHQYGTSVSGHIPTGFIPPQVPS  334
                VAGKEIQERYKDRLKIPLPTELVVVAGATLASHFGDLNHQYGTSVSGHIPTGFIPPQVPS
Sbjct  3282906  VAGKEIQERYKDRLKIPLPTELVVVAGATLASHFGDLNHQYGTSVSGHIPTGFIPPQVPS  3283085

Query  335      LSLMSRVALDAIPLAVI---------------------------SFAFTVSLSEMFAKKN  367
                LSLMSRVALDAIPLAVI                           SFAFTVSLSEMFAKKN
Sbjct  3283086  LSLMSRVALDAIPLAVIR*FLNNVNL*HLAS*S*LVNKNVILSPSFAFTVSLSEMFAKKN  3283265

Query  368      GYTVRPNQEMFAIGCCNIIPSFFHCFTTSAALAKTMVKDSTGCQTQ  413
                GYTVRPNQEMFAIGCCNIIPSFFHCFTTSAALAKTMVKDSTGCQTQ
Sbjct  3283266  GYTVRPNQEMFAIGCCNIIPSFFHCFTTSAALAKTMVKDSTGCQTQ  3283403
```

Range 2: 3281871 to 3282260

Score:266 bits(681), Expect:0.0,
Method:Compositional matrix adjust.,
Identities:130/130(100%), Positives:130/130(100%), Gaps:0/130(0%)

```
Query  1        MEEVSSTPSLLERKAHHAEPTLSVLKSKLQRSLSCSATRVRSTLSGFFPVVRWLPKYKLR  60
                MEEVSSTPSLLERKAHHAEPTLSVLKSKLQRSLSCSATRVRSTLSGFFPVVRWLPKYKLR
Sbjct  3281871  MEEVSSTPSLLERKAHHAEPTLSVLKSKLQRSLSCSATRVRSTLSGFFPVVRWLPKYKLR  3282050

Query  61       EYVWGDGMSGLIVGIILVPQAIAYCLLAGVEPIYGLYTSFYANIIYFLMGTSRHVSVGIF  120
                EYVWGDGMSGLIVGIILVPQAIAYCLLAGVEPIYGLYTSFYANIIYFLMGTSRHVSVGIF
Sbjct  3282051  EYVWGDGMSGLIVGIILVPQAIAYCLLAGVEPIYGLYTSFYANIIYFLMGTSRHVSVGIF  3282230

Query  121      SLMSLMVGQV  130
                SLMSLMVGQV
Sbjct  3282231  SLMSLMVGQV  3282260
```

Range 3: 3282587 to 3282835

Score:172 bits(435), Expect:0.0,
Method:Compositional matrix adjust.,
Identities:83/83(100%), Positives:83/83(100%), Gaps:0/83(0%)

```
Query  192      QVLMAVFRLGFVSVYLSAPMLDGFATGASFTILTVQAKYLLGLKIPRHQGYGTVVVTWFN  251
                QVLMAVFRLGFVSVYLSAPMLDGFATGASFTILTVQAKYLLGLKIPRHQGYGTVVVTWFN
Sbjct  3282587  QVLMAVFRLGFVSVYLSAPMLDGFATGASFTILTVQAKYLLGLKIPRHQGYGTVVVTWFN  3282766

Query  252      IFANIHKTNMCDLITSAICITVL  274
                IFANIHKTNMCDLITSAICITVL
Sbjct  3282767  IFANIHKTNMCDLITSAICITVL  3282835
```

Range 4: 3282301 to 3282513

Score:137 bits(345), Expect:0.0,
Method:Compositional matrix adjust.,
Identities:66/71(93%), Positives:68/71(95%), Gaps:0/71(0%)

```
Query  123      MSLMVGQVVYREVFLAGFDLNEDSPGVPEFNGSFINLTTHTVEIMGVQCGKECYAISVAA  182
                +S +  QVVYREVFLAGFDLNEDSPGVPEFNGSFINLTTHTVEIMGVQCGKECYAISVAA
Sbjct  3282301  LSKLYPQVVYREVFLAGFDLNEDSPGVPEFNGSFINLTTHTVEIMGVQCGKECYAISVAA  3282480

Query  183      ALTLLAGIYQV  193
                ALTLLAGIYQV
Sbjct  3282481  ALTLLAGIYQV  3282513
```

Range 5: 3283845 to 3284414

Score:375 bits(964), Expect:1e-145,
Method:Compositional matrix adjust.,
Identities:189/190(99%), Positives:190/190(100%), Gaps:0/190(0%)

```
Query  509      KVSLLGRAGDSDLYEDVEEYQNLVPPNRVQVFRFQAPLYYANKDTFLKALYQAVGVTPFL  568
                +VSLLGRAGDSDLYEDVEEYQNLVPPNRVQVFRFQAPLYYANKDTFLKALYQAVGVTPFL
Sbjct  3283845  QVSLLGRAGDSDLYEDVEEYQNLVPPNRVQVFRFQAPLYYANKDTFLKALYQAVGVTPFL  3284024

Query  569      EMTRRkkaekkakkMSLTQAQTNGDANHGDVVVALLQGLELDFHTIIVDCSAIPFIDSAG  628
                EMTRRKKAEKKAKKMSLTQAQTNGDANHGDVVVALLQGLELDFHTIIVDCSAIPFIDSAG
Sbjct  3284025  EMTRRKKAEKKAKKMSLTQAQTNGDANHGDVVVALLQGLELDFHTIIVDCSAIPFIDSAG  3284204

Query  629      MAAFKGLLKEFKQIGVEVVLACCNTSIIDTLQKGHFFGKDMNKMLFHTVHHAVLHINIQR  688
                MAAFKGLLKEFKQIGVEVVLACCNTSIIDTLQKGHFFGKDMNKMLFHTVHHAVLHINIQR
Sbjct  3284205  MAAFKGLLKEFKQIGVEVVLACCNTSIIDTLQKGHFFGKDMNKMLFHTVHHAVLHINIQR  3284384

Query  689      PDKSTEDSAL  698
                PDKSTEDSAL
Sbjct  3284385  PDKSTEDSAL  3284414
```

Range 6: 3283531 to 3283770

Score:166 bits(420), Expect:1e-145,
Method:Compositional matrix adjust.,
Identities:80/80(100%), Positives:80/80(100%), Gaps:0/80(0%)

```
Query  432      PFFHALQKCVLACIIIVSLRGALRKFKDVPAKWRASRNDAVVWLVTVSATALIRVELGLV  491
                PFFHALQKCVLACIIIVSLRGALRKFKDVPAKWRASRNDAVVWLVTVSATALIRVELGLV
Sbjct  3283531  PFFHALQKCVLACIIIVSLRGALRKFKDVPAKWRASRNDAVVWLVTVSATALIRVELGLV  3283710

Query  492      VGITFSMICVIYKTQNPKVS  511
                VGITFSMICVIYKTQNPKVS
Sbjct  3283711  VGITFSMICVIYKTQNPKVS  3283770
```

AY Common seadragon *slc26a1*

Query: sulfate anion transporter 1 [Phyllopteryx taeniolatus] Query ID: XP_061655629.1 Length: 712

>Phyllopteryx taeniolatus isolate TA_2022b chromosome 15, whole genome shotgun sequence
Sequence ID: JAMWDW010000015.1 Length: 23884239
Range 1: 9297851 to 9298264

Score:518 bits(1056), Expect:0.0,
Method:,
Identities:138/138(100%), Positives:138/138(100%), Gaps:0/138(0%)

```
Query  1        MEEFGKLKKCPPAHPPSLLERRARPPQHTVTILKSKLKQSVSCSVPRVRSTLSGLFPVLH  60
                MEEFGKLKKCPPAHPPSLLERRARPPQHTVTILKSKLKQSVSCSVPRVRSTLSGLFPVLH
Sbjct  9298264  MEEFGKLKKCPPAHPPSLLERRARPPQHTVTILKSKLKQSVSCSVPRVRSTLSGLFPVLH  9298085
```

```
Query  61       WLPRYNLREYVWGDAMSGLIVGIILVPQAIAYCLLAGVEPIYGLYTSFYANIIYFLMGTS  120
                WLPRYNLREYVWGDAMSGLIVGIILVPQAIAYCLLAGVEPIYGLYTSFYANIIYFLMGTS
Sbjct  9298084  WLPRYNLREYVWGDAMSGLIVGIILVPQAIAYCLLAGVEPIYGLYTSFYANIIYFLMGTS  9297905

Query  121      RHVSVGIFSLMSLMVGQV  138
                RHVSVGIFSLMSLMVGQV
Sbjct  9297904  RHVSVGIFSLMSLMVGQV  9297851
```

Range 2: 9297559 to 9297765

Score:252 bits(513), Expect:0.0,
Method:,
Identities:69/69(100%), Positives:69/69(100%), Gaps:0/69(0%)

```
Query  137      QVVNREVFLAGFDLNEDSSGPPEFNGSFSINLTASQTHCVEILGVQCGKECYAISIAAAL  196
                QVVNREVFLAGFDLNEDSSGPPEFNGSFSINLTASQTHCVEILGVQCGKECYAISIAAAL
Sbjct  9297765  QVVNREVFLAGFDLNEDSSGPPEFNGSFSINLTASQTHCVEILGVQCGKECYAISIAAAL  9297586

Query  197      TFLAGTYQV  205
                TFLAGTYQV
Sbjct  9297585  TFLAGTYQV  9297559
```

Range 3: 9294486 to 9295058

Score:629 bits(1284), Expect:8e-179,
Method:,
Identities:191/191(100%), Positives:191/191(100%), Gaps:0/191(0%)

```
Query  522      VSLLGRANDSDLYEDVEEYQNLVPPTRVRVFRFQAPLYYANKDTFLKALYKAVGVTPFLE  581
                VSLLGRANDSDLYEDVEEYQNLVPPTRVRVFRFQAPLYYANKDTFLKALYKAVGVTPFLE
Sbjct  9295058  VSLLGRANDSDLYEDVEEYQNLVPPTRVRVFRFQAPLYYANKDTFLKALYKAVGVTPFLE  9294879

Query  582      MTRRkkagkkakkMSMTQTQANGDTNNGDVVVALVQRELDFHAIILDCSAIPFIDSTGMA  641
                MTRRKKAGKKAKKMSMTQTQANGDTNNGDVVVALVQRELDFHAIILDCSAIPFIDSTGMA
Sbjct  9294878  MTRRKKAGKKAKKMSMTQTQANGDTNNGDVVVALVQRELDFHAIILDCSAIPFIDSTGMA  9294699

Query  642      AFKGLLTEFEEIGVRVVLACCNITLIDTLqkgqffgkggkDMSNMLFHTVHHAVLQTNIR  701
                AFKGLLTEFEEIGVRVVLACCNITLIDTLQKGQFFGKGGKDMSNMLFHTVHHAVLQTNIR
Sbjct  9294698  AFKGLLTEFEEIGVRVVLACCNITLIDTLQKGQFFGKGGKDMSNMLFHTVHHAVLQTNIR  9294519

Query  702      QTDKGLEDSDL  712
                QTDKGLEDSDL
Sbjct  9294518  QTDKGLEDSDL  9294486
```

Range 4: 9297065 to 9297313

Score:310 bits(632), Expect:1e-160,
Method:,
Identities:83/83(100%), Positives:83/83(100%), Gaps:0/83(0%)

```
Query  204      QVMMAVFRLGFVSVYLSAPMLDGFATGASFTILTVQAKYLLGLKIPRHRGYGTVVVTWFN  263
                QVMMAVFRLGFVSVYLSAPMLDGFATGASFTILTVQAKYLLGLKIPRHRGYGTVVVTWFN
Sbjct  9297313  QVMMAVFRLGFVSVYLSAPMLDGFATGASFTILTVQAKYLLGLKIPRHRGYGTVVVTWFN  9297134

Query  264      IFANIHKTNVCDLITSAICITVL  286
                IFANIHKTNVCDLITSAICITVL
Sbjct  9297133  IFANIHKTNVCDLITSAICITVL  9297065
```

Range 5: 9296746 to 9296976

Score:282 bits(573), Expect:1e-160,
Method:,
Identities:77/77(100%), Positives:77/77(100%), Gaps:0/77(0%)

```
Query  287      VAGKEIQERYKNRLKIPLPTELVVVAGATLASHFGNLNHHYSTSVSGHIPTGFMPPQVPS  346
                VAGKEIQERYKNRLKIPLPTELVVVAGATLASHFGNLNHHYSTSVSGHIPTGFMPPQVPS
Sbjct  9296976  VAGKEIQERYKNRLKIPLPTELVVVAGATLASHFGNLNHHYSTSVSGHIPTGFMPPQVPS  9296797

Query  347      FSLMPRVALDAVPLALI  363
                FSLMPRVALDAVPLALI
Sbjct  9296796  FSLMPRVALDAVPLALI  9296746
```

Range 6: 9295592 to 9295831

Score:299 bits(608), Expect:4e-79,
Method:,
Identities:80/80(100%), Positives:80/80(100%), Gaps:0/80(0%)

```
Query  444      PFFHALQKCVLACIIIVSLRGALRKFKDLPAKWRASRNDAVVWLVTMSATALISVELGLL  503
                PFFHALQKCVLACIIIVSLRGALRKFKDLPAKWRASRNDAVVWLVTMSATALISVELGLL
Sbjct  9295831  PFFHALQKCVLACIIIVSLRGALRKFKDLPAKWRASRNDAVVWLVTMSATALISVELGLL  9295652

Query  504      IGITFSMICIIYKTQNPKVS  523
```

```
             IGITFSMICIIYKTQNPKVS
Sbjct  9295651  IGITFSMICIIYKTQNPKVS  9295592
```

Range 7: 9295977 to 9296162

Score:235 bits(477), Expect:7e-60,
Method:,
Identities:62/62(100%), Positives:62/62(100%), Gaps:0/62(0%)

```
Query  364      SFAFTVSLSEMFAKKNGYTVRPNQEMFAIGCCNIIPSFFHCFTTSAALAKTMVKDSTGCE  423
                SFAFTVSLSEMFAKKNGYTVRPNQEMFAIGCCNIIPSFFHCFTTSAALAKTMVKDSTGCE
Sbjct  9296162  SFAFTVSLSEMFAKKNGYTVRPNQEMFAIGCCNIIPSFFHCFTTSAALAKTMVKDSTGCE  9295983

Query  424      TQ  425
                TQ
Sbjct  9295982  TQ  9295977
```

AZ Big-belly seahorse *slc26a1*

Query: unnamed protein product Query ID: lcl|Query_63477 Length: 712

>Hippocampus abdominalis strain HM12 chromosome 18, whole genome shotgun sequence
Sequence ID: JAGPNR010000010.1 Length: 13991795
Range 1: 4126961 to 4127536

Score:397 bits(1019), Expect:6e-119,
Method:Compositional matrix adjust.,
Identities:191/192(99%), Positives:192/192(100%), Gaps:0/192(0%)

```
Query  521      RVSLLGRVGDSDLYEDVEEYQNLVPPSRVRVFRFQAPLYYANKDTFLKALYKAVGVTPFL  580
                +VSLLGRVGDSDLYEDVEEYQNLVPPSRVRVFRFQAPLYYANKDTFLKALYKAVGVTPFL
Sbjct  4126961  QVSLLGRVGDSDLYEDVEEYQNLVPPSRVRVFRFQAPLYYANKDTFLKALYKAVGVTPFL  4127140

Query  581      EMTSRRKAEKKAKKMSVTQAQAIGNANNGDVVVALVQRQLDFHTIILDCSAIPFLDSTGL  640
                EMTSRRKAEKKAKKMSVTQAQAIGNANNGDVVVALVQRQLDFHTIILDCSAIPFLDSTGL
Sbjct  4127141  EMTSRRKAEKKAKKMSVTQAQAIGNANNGDVVVALVQRQLDFHTIILDCSAIPFLDSTGL  4127320

Query  641      VAFKGLFKEFAEIDVKVVLACCNTSLIDILQKGQLFGKGDKDMSKMLFHTVHHAILYVNI  700
                VAFKGLFKEFAEIDVKVVLACCNTSLIDILQKGQLFGKGDKDMSKMLFHTVHHAILYVNI
Sbjct  4127321  VAFKGLFKEFAEIDVKVVLACCNTSLIDILQKGQLFGKGDKDMSKMLFHTVHHAILYVNI  4127500

Query  701      QQPDKSSEDSVL  712
                QQPDKSSEDSVL
Sbjct  4127501  QQPDKSSEDSVL  4127536
```

Range 2: 4124479 to 4124892

Score:289 bits(739), Expect:3e-113,
Method:Compositional matrix adjust.,
Identities:138/138(100%), Positives:138/138(100%), Gaps:0/138(0%)

```
Query  1        MEEVNLRKESVTDHPPSLLERKARHVQPTLWVLKSKLQRSLSCSMPRVRSTLSGFFPVVR  60
                MEEVNLRKESVTDHPPSLLERKARHVQPTLWVLKSKLQRSLSCSMPRVRSTLSGFFPVVR
Sbjct  4124479  MEEVNLRKESVTDHPPSLLERKARHVQPTLWVLKSKLQRSLSCSMPRVRSTLSGFFPVVR  4124658

Query  61       WLPKYKLREYVWGDAMSGLIVGIILVPQAIAYCLLAGVEPIYGLYTSFYANIIYFLMGTS  120
                WLPKYKLREYVWGDAMSGLIVGIILVPQAIAYCLLAGVEPIYGLYTSFYANIIYFLMGTS
Sbjct  4124659  WLPKYKLREYVWGDAMSGLIVGIILVPQAIAYCLLAGVEPIYGLYTSFYANIIYFLMGTS  4124838

Query  121      RHVSVGIFSLMSLMVGQV  138
                RHVSVGIFSLMSLMVGQV
Sbjct  4124839  RHVSVGIFSLMSLMVGQV  4124892
```

Range 3: 4125003 to 4125209

Score:143 bits(361), Expect:3e-113,
Method:Compositional matrix adjust.,
Identities:69/69(100%), Positives:69/69(100%), Gaps:0/69(0%)

```
Query  137      QVVDREVFLAGFDLNEEPSGPLEFNGSFSINLTSSQTQSVEIMGVQCGKECYAISIAAAL  196
                QVVDREVFLAGFDLNEEPSGPLEFNGSFSINLTSSQTQSVEIMGVQCGKECYAISIAAAL
Sbjct  4125003  QVVDREVFLAGFDLNEEPSGPLEFNGSFSINLTSSQTQSVEIMGVQCGKECYAISIAAAL  4125182

Query  197      TFLAGIYQV  205
                TFLAGIYQV
Sbjct  4125183  TFLAGIYQV  4125209
```

Range 4: 4125434 to 4125682

```
Score:171 bits(434), Expect:2e-83,
Method:Compositional matrix adjust.,
Identities:83/83(100%), Positives:83/83(100%), Gaps:0/83(0%)

Query  204      QVMMAVFRLGFVSVYLSAPMLDGFATGASFTILTVQAKYLLGLKIPRHQGYGTVVITWIN  263
                QVMMAVFRLGFVSVYLSAPMLDGFATGASFTILTVQAKYLLGLKIPRHQGYGTVVITWIN
Sbjct  4125434  QVMMAVFRLGFVSVYLSAPMLDGFATGASFTILTVQAKYLLGLKIPRHQGYGTVVITWIN  4125613

Query  264      IFANIHKTNICDLITSAICITVL  286
                IFANIHKTNICDLITSAICITVL
Sbjct  4125614  IFANIHKTNICDLITSAICITVL  4125682



Range 5: 4125777 to 4126010

Score:161 bits(408), Expect:2e-83,
Method:Compositional matrix adjust.,
Identities:77/78(99%), Positives:78/78(100%), Gaps:0/78(0%)

Query  286      LVAGKEIQERYKDRLKIPLPTELVVVAGATLASHFGNLNHHYSTSVSGHIPTGFIPPQVP  345
                +VAGKEIQERYKDRLKIPLPTELVVVAGATLASHFGNLNHHYSTSVSGHIPTGFIPPQVP
Sbjct  4125777  VVAGKEIQERYKDRLKIPLPTELVVVAGATLASHFGNLNHHYSTSVSGHIPTGFIPPQVP  4125956

Query  346      SFSLMPRVALDAIPVAVI  363
                SFSLMPRVALDAIPVAVI
Sbjct  4125957  SFSLMPRVALDAIPVAVI  4126010



Range 6: 4126093 to 4126731

Score:259 bits(663), Expect:3e-72,
Method:Compositional matrix adjust.,
Identities:163/219(74%), Positives:170/219(77%), Gaps:35/219(15%)

Query  345      PSFSLMPRVALDAIPVAVISFAFTVSLSEMFAKKNGYTVRPNQEMFAIGCCNIIPSFFHC  404
                P   ++ R+ L +      SFAFTVSLSEMFAKKNGYTVRPNQEMFAIGCCNIIPSFFHC
Sbjct  4126093  PEEWILKRIVLSS------SFAFTVSLSEMFAKKNGYTVRPNQEMFAIGCCNIIPSFFHC  4126254

Query  405      FTTSAALAKTMVKDSTGCQTQvss----------------------------lvsalvi  435
                FTTSAALAKTMVKDSTGCQTQV S                            LVSALVI
Sbjct  4126255  FTTSAALAKTMVKDSTGCQTQVMSKQKKCATEQHGLLGM*KSKS*VCFHQVSSLVSALVI  4126434

Query  436      llvllffapffhalQKCVLACIIIVSLRGALRKFKDVPAKWRASRNDATIWLITMSATAL  495
                LLVLLFFAPFFHALQKCVLACIIIVSLRGALRKFKDVPAKWRASRNDATIWLITMSATAL
Sbjct  4126435  LLVLLFFAPFFHALQKCVLACIIIVSLRGALRKFKDVPAKWRASRNDATIWLITMSATAL  4126614

Query  496      ISVELGLLVGITFSLICIIYKTQNPRVSLLGRVGDSDLY  534
                ISVELGLLVGITFSLICIIYKTQNPRVS   R    +++
Sbjct  4126615  ISVELGLLVGITFSLICIIYKTQNPRVSDSNRTESYNIH  4126731
```

BA Mudskipper *slc26a1*

```
Query: sulfate anion transporter 1 [Boleophthalmus pectinirostris] Query ID: XP_055009644.1 Length: 737


>Boleophthalmus pectinirostris isolate CB_2022bp ecotype Guang Dong unplaced genomic scaffold, ASM2622593v1 HiC_scaffold_18, whole genome shotgun sequence
Sequence ID: NW_026571133.1 Length: 47286877
Range 1: 33095077 to 33095652

Score:703 bits(1434), Expect:0.0,
Method:,
Identities:192/192(100%), Positives:192/192(100%), Gaps:0/192(0%)

Query  546       QVSLLGRASDCNLYEDMDEYKNLLPPARVHVFRYQAPLYYANKDAFLKALYKRVGVEPFL  605
                 QVSLLGRASDCNLYEDMDEYKNLLPPARVHVFRYQAPLYYANKDAFLKALYKRVGVEPFL
Sbjct  33095077  QVSLLGRASDCNLYEDMDEYKNLLPPARVHVFRYQAPLYYANKDAFLKALYKRVGVEPFL  33095256

Query  606       ELTRRKKEHKKAKMSAKHTGGDKTNGEVVVGLIKSELDFHSIVLDCSAIPFIDSSGMATL  665
                 ELTRRKKEHKKAKMSAKHTGGDKTNGEVVVGLIKSELDFHSIVLDCSAIPFIDSSGMATL
Sbjct  33095257  ELTRRKKEHKKAKMSAKHTGGDKTNGEVVVGLIKSELDFHSIVLDCSAIPFIDSSGMATL  33095436

Query  666       KSVVKEYKDIGVSVYLASCYASVIDALQKGEFFGKNNQNMGQLLFHTVHTAVEYALACKA  725
                 KSVVKEYKDIGVSVYLASCYASVIDALQKGEFFGKNNQNMGQLLFHTVHTAVEYALACKA
Sbjct  33095437  KSVVKEYKDIGVSVYLASCYASVIDALQKGEFFGKNNQNMGQLLFHTVHTAVEYALACKA  33095616

Query  726       AEGESKSAASVV  737
                 AEGESKSAASVV
Sbjct  33095617  AEGESKSAASVV  33095652



Range 2: 33090259 to 33090735
```

```
Score:601 bits(1226), Expect:2e-175,
Method:,
Identities:159/159(100%), Positives:159/159(100%), Gaps:0/159(0%)

Query  6         NTADHFILPPPPYHHTLLRTMEEDLANTPEPSPLPPPPLLERQLRQRPSAITLLKSKMKH  65
                 NTADHFILPPPPYHHTLLRTMEEDLANTPEPSPLPPPPLLERQLRQRPSAITLLKSKMKH
Sbjct  33090259  NTADHFILPPPPYHHTLLRTMEEDLANTPEPSPLPPPPLLERQLRQRPSAITLLKSKMKH  33090438

Query  66        GVDCSLPKVRSTLTGFFPVVRWLPKYKLKEYIWGDIMSGLIVGIILVPQAIAYCLLAGVD  125
                 GVDCSLPKVRSTLTGFFPVVRWLPKYKLKEYIWGDIMSGLIVGIILVPQAIAYCLLAGVD
Sbjct  33090439  GVDCSLPKVRSTLTGFFPVVRWLPKYKLKEYIWGDIMSGLIVGIILVPQAIAYCLLAGVD  33090618

Query  126       PIYGLYTSFYANIIYFLMGTSRHVSVGIFSLMSLMVGQV  164
                 PIYGLYTSFYANIIYFLMGTSRHVSVGIFSLMSLMVGQV
Sbjct  33090619  PIYGLYTSFYANIIYFLMGTSRHVSVGIFSLMSLMVGQV  33090735



Range 3: 33092987 to 33093220

Score:282 bits(574), Expect:4e-144,
Method:,
Identities:78/78(100%), Positives:78/78(100%), Gaps:0/78(0%)

Query  311       LVAGKELQDRYKDRLKMPLPTELVVVAAATLASHFGDLNSKYGSSVSGHIPTGFIPPKAP  370
                 LVAGKELQDRYKDRLKMPLPTELVVVAAATLASHFGDLNSKYGSSVSGHIPTGFIPPKAP
Sbjct  33092987  LVAGKELQDRYKDRLKMPLPTELVVVAAATLASHFGDLNSKYGSSVSGHIPTGFIPPKAP  33093166

Query  371       SLEIMPQIALDAIPLAVI  388
                 SLEIMPQIALDAIPLAVI
Sbjct  33093167  SLEIMPQIALDAIPLAVI  33093220



Range 4: 33093305 to 33093493

Score:238 bits(484), Expect:4e-144,
Method:,
Identities:63/63(100%), Positives:63/63(100%), Gaps:0/63(0%)

Query  389       SFAFTVSLSEMMAKKHCYTVRPNQEMLAIGFCNIIPSFFHSFTTSAALAKTMVKDSTGCQ  448
                 SFAFTVSLSEMMAKKHCYTVRPNQEMLAIGFCNIIPSFFHSFTTSAALAKTMVKDSTGCQ
Sbjct  33093305  SFAFTVSLSEMMAKKHCYTVRPNQEMLAIGFCNIIPSFFHSFTTSAALAKTMVKDSTGCQ  33093484

Query  449       TQV  451
                 TQV
Sbjct  33093485  TQV  33093493



Range 5: 33094205 to 33094492

Score:349 bits(711), Expect:2e-99,
Method:,
Identities:96/96(100%), Positives:96/96(100%), Gaps:0/96(0%)

Query  450       QVSSLISALVVLLVLLFFAPFFYSLQKCVLASIIIVSLRGALRKFKDVPAKWRASREDAV  509
                 QVSSLISALVVLLVLLFFAPFFYSLQKCVLASIIIVSLRGALRKFKDVPAKWRASREDAV
Sbjct  33094205  QVSSLISALVVLLVLLFFAPFFYSLQKCVLASIIIVSLRGALRKFKDVPAKWRASREDAV  33094384

Query  510       VWLVAMAATALISVELGLLVGIVFSMTVVIYKTQKP  545
                 VWLVAMAATALISVELGLLVGIVFSMTVVIYKTQKP
Sbjct  33094385  VWLVAMAATALISVELGLLVGIVFSMTVVIYKTQKP  33094492



Range 6: 33092487 to 33092735

Score:310 bits(631), Expect:1e-87,
Method:,
Identities:83/83(100%), Positives:83/83(100%), Gaps:0/83(0%)

Query  229       QVLMAVFRLGFVSVYLSAPMLDGFATGASFTILTVQAKYLLGLKIPRHQGPGVVVVTWFN  288
                 QVLMAVFRLGFVSVYLSAPMLDGFATGASFTILTVQAKYLLGLKIPRHQGPGVVVVTWFN
Sbjct  33092487  QVLMAVFRLGFVSVYLSAPMLDGFATGASFTILTVQAKYLLGLKIPRHQGPGVVVVTWFN  33092666

Query  289       IFANIHKTNFCDLITSAICIFIL  311
                 IFANIHKTNFCDLITSAICIFIL
Sbjct  33092667  IFANIHKTNFCDLITSAICIFIL  33092735



Range 7: 33091610 to 33091813

Score:251 bits(511), Expect:5e-70,
Method:,
Identities:68/68(100%), Positives:68/68(100%), Gaps:0/68(0%)

Query  163       QVVDREVFQAGFDTSEDSKISKLNLLNGTLDINMTTKTIELFGVEFEKESYAIMVATTLT  222
                 QVVDREVFQAGFDTSEDSKISKLNLLNGTLDINMTTKTIELFGVEFEKESYAIMVATTLT
Sbjct  33091610  QVVDREVFQAGFDTSEDSKISKLNLLNGTLDINMTTKTIELFGVEFEKESYAIMVATTLT  33091789
```

```
Query  223       CLAGIYQV  230
                 CLAGIYQV
Sbjct  33091790  CLAGIYQV  33091813
```

BB Indian glassy fish *slc26a1*

Query: sulfate anion transporter 1-like isoform X2 [Parambassis ranga] Query ID: XP_028273709.1 Length: 715

>Parambassis ranga chromosome 12, fParRan2.1, whole genome shotgun sequence
Sequence ID: NC_041032.1 Length: 17157085
Range 1: 7514916 to 7515488

Score:700 bits(1429), Expect:0.0,
Method:,
Identities:191/191(100%), Positives:191/191(100%), Gaps:0/191(0%)

```
Query  525      VALLGRLNSTDLYEDLDEYKNLTPPSRVQIFRFQAPLYYANKESFLKSLYRAVGVQPFLE  584
                VALLGRLNSTDLYEDLDEYKNLTPPSRVQIFRFQAPLYYANKESFLKSLYRAVGVQPFLE
Sbjct  7514916  VALLGRLNSTDLYEDLDEYKNLTPPSRVQIFRFQAPLYYANKESFLKSLYRAVGVQPFLE  7515095

Query  585      LTKRKKAEKKAKEMSKKQIKAERYNDDVIVGFVQRDLGFHTIILDCSAIPFIDSTGMGTF  644
                LTKRKKAEKKAKEMSKKQIKAERYNDDVIVGFVQRDLGFHTIILDCSAIPFIDSTGMGTF
Sbjct  7515096  LTKRKKAEKKAKEMSKKQIKAERYNDDVIVGFVQRDLGFHTIILDCSAIPFIDSTGMGTF  7515275

Query  645      KALVKEYKEIGICVLLASCNTSVIDTLQKGQFFGKDGKDMSSLLFHTVHAAVLHANIASA  704
                KALVKEYKEIGICVLLASCNTSVIDTLQKGQFFGKDGKDMSSLLFHTVHAAVLHANIASA
Sbjct  7515276  KALVKEYKEIGICVLLASCNTSVIDTLQKGQFFGKDGKDMSSLLFHTVHAAVLHANIASA  7515455

Query  705      ASEIRSEDSVV  715
                ASEIRSEDSVV
Sbjct  7515456  ASEIRSEDSVV  7515488
```

Range 2: 7512471 to 7512887

Score:521 bits(1062), Expect:0.0,
Method:,
Identities:138/139(99%), Positives:139/139(100%), Gaps:0/139(0%)

```
Query  1        MEEYPKVTETPEAPPPLLERCVRQRQPTISVLKSKLKQGANCSGPKVRSTLTGFFPVVRW  60
                MEEYPKVTETPEAPPPLLERCVRQRQPTISVLKSKLKQGANCSGPKVRSTLTGFFPVVRW
Sbjct  7512471  MEEYPKVTETPEAPPPLLERCVRQRQPTISVLKSKLKQGANCSGPKVRSTLTGFFPVVRW  7512650

Query  61       LPKYKLREYIWGDVMSGVIVGIILVPQAIAYCLLAGVDPIYGLYTSFYANIIYFLMGTSR  120
                LPKYKLREYIWGDVMSGVIVGIILVPQAIAYCLLAGVDPIYGLYTSFYANIIYFLMGTSR
Sbjct  7512651  LPKYKLREYIWGDVMSGVIVGIILVPQAIAYCLLAGVDPIYGLYTSFYANIIYFLMGTSR  7512830

Query  121      HVSVGIFSLMSLMVGQVVD  139
                HVSVGIFSLMSLMVGQV+D
Sbjct  7512831  HVSVGIFSLMSLMVGQVID  7512887
```

Range 3: 7514526 to 7514819

Score:363 bits(739), Expect:0.0,
Method:,
Identities:98/98(100%), Positives:98/98(100%), Gaps:0/98(0%)

```
Query  428      QVSSLVSAMVVLLVLLYLAPFFYALQKCVLACIIIVSLRGALRKFRDAPALWRASKNDAI  487
                QVSSLVSAMVVLLVLLYLAPFFYALQKCVLACIIIVSLRGALRKFRDAPALWRASKNDAI
Sbjct  7514526  QVSSLVSAMVVLLVLLYLAPFFYALQKCVLACIIIVSLRGALRKFRDAPALWRASKNDAI  7514705

Query  488      VWLVTMAATSLISVELGLLVGMIFSMSCIIFKTQNPKV  525
                VWLVTMAATSLISVELGLLVGMIFSMSCIIFKTQNPKV
Sbjct  7514706  VWLVTMAATSLISVELGLLVGMIFSMSCIIFKTQNPKV  7514819
```

Range 4: 7512957 to 7513175

Score:266 bits(541), Expect:0.0,
Method:,
Identities:73/73(100%), Positives:73/73(100%), Gaps:0/73(0%)

```
Query  136      QVVDREVFLAGFDLNEDSTSSSASNNVFNGTLGTNITEGKVYTVELMGMQCGKEYYAISV  195
                QVVDREVFLAGFDLNEDSTSSSASNNVFNGTLGTNITEGKVYTVELMGMQCGKEYYAISV
Sbjct  7512957  QVVDREVFLAGFDLNEDSTSSSASNNVFNGTLGTNITEGKVYTVELMGMQCGKEYYAISV  7513136

Query  196      AAAVTFLAGVYQV  208
                AAAVTFLAGVYQV
Sbjct  7513137  AAAVTFLAGVYQV  7513175
```

Range 5: 7514225 to 7514413

Score:238 bits(484), Expect:0.0,
Method:,
Identities:63/63(100%), Positives:63/63(100%), Gaps:0/63(0%)

```
Query  367      SFAFTVSLSEMFAKKNGYTVRPNQEMLAIGLCNIIPSFFHCFTTSAAMAKTMVRDSTGCQ  426
                SFAFTVSLSEMFAKKNGYTVRPNQEMLAIGLCNIIPSFFHCFTTSAAMAKTMVRDSTGCQ
Sbjct  7514225  SFAFTVSLSEMFAKKNGYTVRPNQEMLAIGLCNIIPSFFHCFTTSAAMAKTMVRDSTGCQ  7514404

Query  427      TQV  429
                TQV
Sbjct  7514405  TQV  7514413
```

Range 6: 7513436 to 7513684

Score:311 bits(633), Expect:5e-165,
Method:,
Identities:83/83(100%), Positives:83/83(100%), Gaps:0/83(0%)

```
Query  207      QVMMAVFRLGFVSVYLSSPMLDGFATGASFTILTVQAKYLLGLKIPRHQGYGTVVVTWIN  266
                QVMMAVFRLGFVSVYLSSPMLDGFATGASFTILTVQAKYLLGLKIPRHQGYGTVVVTWIN
Sbjct  7513436  QVMMAVFRLGFVSVYLSSPMLDGFATGASFTILTVQAKYLLGLKIPRHQGYGTVVVTWIN  7513615

Query  267      IFANIHKTNFCDLITSAICISIL  289
                IFANIHKTNFCDLITSAICISIL
Sbjct  7513616  IFANIHKTNFCDLITSAICISIL  7513684
```

Range 7: 7513779 to 7514015

Score:278 bits(566), Expect:5e-165,
Method:,
Identities:78/79(99%), Positives:78/79(98%), Gaps:0/79(0%)

```
Query  288      ILVAGKEIQERYKDRLKIPLPTELIVVAGATLASHSGELNRRYGSSISGHIPTGFIPPQV  347
                I VAGKEIQERYKDRLKIPLPTELIVVAGATLASHSGELNRRYGSSISGHIPTGFIPPQV
Sbjct  7513779  ITVAGKEIQERYKDRLKIPLPTELIVVAGATLASHSGELNRRYGSSISGHIPTGFIPPQV  7513958

Query  348      PSLSLMPRVALDAIPLSVI  366
                PSLSLMPRVALDAIPLSVI
Sbjct  7513959  PSLSLMPRVALDAIPLSVI  7514015
```

BC Clown anemonefish *slc26a1*

Query: sulfate anion transporter 1 [Amphiprion ocellaris] Query ID: XP_023117813.1 Length: 710

>Amphiprion ocellaris isolate individual 3 ecotype Okinawa chromosome 17, ASM2253959v1, whole genome shotgun sequence
Sequence ID: NC_072782.1 Length: 33922019
Range 1: 2326515 to 2327084

Score:695 bits(1419), Expect:0.0,
Method:,
Identities:190/190(100%), Positives:190/190(100%), Gaps:0/190(0%)

```
Query  521      VSLLGRVSDIDLYEDLEEYKNLTPPPRIQVFRFQAPLYYANKDSFLRSLYKAVGVEPFLE  580
                VSLLGRVSDIDLYEDLEEYKNLTPPPRIQVFRFQAPLYYANKDSFLRSLYKAVGVEPFLE
Sbjct  2327084  VSLLGRVSDIDLYEDLEEYKNLTPPPRIQVFRFQAPLYYANKDSFLRSLYKAVGVEPFLE  2326905

Query  581      LTKRRKAEKKAKEMSLKQAKANGEKNNGDIIVGLVQRELEFHTIVLDCSAIPFIDSTGMA  640
                LTKRRKAEKKAKEMSLKQAKANGEKNNGDIIVGLVQRELEFHTIVLDCSAIPFIDSTGMA
Sbjct  2326904  LTKRRKAEKKAKEMSLKQAKANGEKNNGDIIVGLVQRELEFHTIVLDCSAIPFIDSTGMA  2326725

Query  641      TFKTLVKEYKEIGVSVLLASCSTTVIDTLQKGQFFGKNDVDMSSLLFYTVHAAILYANSA  700
                TFKTLVKEYKEIGVSVLLASCSTTVIDTLQKGQFFGKNDVDMSSLLFYTVHAAILYANSA
Sbjct  2326724  TFKTLVKEYKEIGVSVLLASCSTTVIDTLQKGQFFGKNDVDMSSLLFYTVHAAILYANSA  2326545

Query  701      AESKPEDSVV  710
                AESKPEDSVV
Sbjct  2326544  AESKPEDSVV  2326515
```

Range 2: 2327153 to 2327449

Score:362 bits(737), Expect:0.0,
Method:,
Identities:99/99(100%), Positives:99/99(100%), Gaps:0/99(0%)

```
Query  424      QVSSLISAVVVLLVLLVFAPFFYSLQKCVLACIIIVSLRGALRKFKDVPAKWRASRNDAI  483
                QVSSLISAVVVLLVLLVFAPFFYSLQKCVLACIIIVSLRGALRKFKDVPAKWRASRNDAI
Sbjct  2327449  QVSSLISAVVVLLVLLVFAPFFYSLQKCVLACIIIVSLRGALRKFKDVPAKWRASRNDAI  2327270

Query  484      VWLVTMSATALISVELGLLVGVVFSMSCIIFKTQNPKVS  522
```

```
                 VWLVTMSATALISVELGLLVGVVFSMSCIIFKTQNPKVS
Sbjct  2327269  VWLVTMSATALISVELGLLVGVVFSMSCIIFKTQNPKVS  2327153
```

Range 3: 2329385 to 2329633

Score:309 bits(629), Expect:5e-164,
Method:,
Identities:83/83(100%), Positives:83/83(100%), Gaps:0/83(0%)

```
Query  203      QILMAIFRLGFVSVYLSGPMLDGFATGASFTILTVQAKYLLGLKIPRHQGYGTVVVTWIN  262
                QILMAIFRLGFVSVYLSGPMLDGFATGASFTILTVQAKYLLGLKIPRHQGYGTVVVTWIN
Sbjct  2329633  QILMAIFRLGFVSVYLSGPMLDGFATGASFTILTVQAKYLLGLKIPRHQGYGTVVVTWIN  2329454

Query  263      IFANIHKTNLCDLITSAICISIL  285
                IFANIHKTNLCDLITSAICISIL
Sbjct  2329453  IFANIHKTNLCDLITSAICISIL  2329385
```

Range 4: 2329046 to 2329282

Score:279 bits(567), Expect:5e-164,
Method:,
Identities:78/79(99%), Positives:78/79(98%), Gaps:0/79(0%)

```
Query  284      ILVAGKEIQDRYKDRLKIPLPTELVVVAGATLASHFGDLNGRYGSSVSGHIPTGFIPPQV  343
                I VAGKEIQDRYKDRLKIPLPTELVVVAGATLASHFGDLNGRYGSSVSGHIPTGFIPPQV
Sbjct  2329282  ITVAGKEIQDRYKDRLKIPLPTELVVVAGATLASHFGDLNGRYGSSVSGHIPTGFIPPQV  2329103

Query  344      PAFSLMPRVALDAIPLAVI  362
                PAFSLMPRVALDAIPLAVI
Sbjct  2329102  PAFSLMPRVALDAIPLAVI  2329046
```

Range 5: 2332863 to 2333273

Score:508 bits(1036), Expect:4e-147,
Method:,
Identities:136/137(99%), Positives:136/137(99%), Gaps:0/137(0%)

```
Query  1        MEEVSKETPLAPPPLLERRVRQRQRTASVLRSKLKQGVTCSIPRVRSTLTGFFPVVRWLP  60
                MEEVSKETPLAPPPLLERRVRQRQRTASVLRSKLKQGVTCSIPRVRSTLTGFFPVVRWLP
Sbjct  2333273  MEEVSKETPLAPPPLLERRVRQRQRTASVLRSKLKQGVTCSIPRVRSTLTGFFPVVRWLP  2333094

Query  61       KYKLREYVWGDVMSGLIVGIILVPQAIAYCLLAGVEPIYGLYTSFYANIIYFLMGTSRHV  120
                KYKLREYVWGDVMSGLIVGIILVPQAIAYCLLAGVEPIYGLYTSFYANIIYFLMGTSRHV
Sbjct  2333093  KYKLREYVWGDVMSGLIVGIILVPQAIAYCLLAGVEPIYGLYTSFYANIIYFLMGTSRHV  2332914

Query  121      SVGIFSLMSLMVGQVVD  137
                SVGIFSLMSLMVGQV D
Sbjct  2332913  SVGIFSLMSLMVGQVTD  2332863
```

Range 6: 2331510 to 2331722

Score:259 bits(526), Expect:7e-72,
Method:,
Identities:70/71(99%), Positives:71/71(100%), Gaps:0/71(0%)

```
Query  134      QVVDREVFLAGFDLNEESTKSSPDVLNGTFGFNLTGGTVHTVELMGMQCGKECYAITIAA  193
                QVVDREVFLAGFDLNEESTKSSPDVLNGTFGFNLTGGTVHTVELMGMQCGKECYAITIAA
Sbjct  2331722  QVVDREVFLAGFDLNEESTKSSPDVLNGTFGFNLTGGTVHTVELMGMQCGKECYAITIAA  2331543

Query  194      AVTFLAGIYQI  204
                AVTFLAGIYQ+
Sbjct  2331542  AVTFLAGIYQV  2331510
```

Range 7: 2327834 to 2328022

Score:236 bits(479), Expect:6e-65,
Method:,
Identities:63/63(100%), Positives:63/63(100%), Gaps:0/63(0%)

```
Query  363      SFAFTVSLSEMFAKKNGYTVRPNQEMLAIGLCNIIPSFFHCFTTSAALAKTMVKDSTGCQ  422
                SFAFTVSLSEMFAKKNGYTVRPNQEMLAIGLCNIIPSFFHCFTTSAALAKTMVKDSTGCQ
Sbjct  2328022  SFAFTVSLSEMFAKKNGYTVRPNQEMLAIGLCNIIPSFFHCFTTSAALAKTMVKDSTGCQ  2327843

Query  423      TQV  425
                TQV
Sbjct  2327842  TQV  2327834
```

BD Jewelled blenny *slc26a1*

Query: sulfate anion transporter 1-like [Salarias fasciatus] Query ID: XP_029941310.1 Length: 714

>Salarias fasciatus chromosome 7 unlocalized genomic scaffold, fSalaFa1.1 super_scaffold_4, whole genome shotgun sequence
Sequence ID: NW_021941229.1 Length: 28306296
Range 1: 8403281 to 8403859

Score:698 bits(1425), Expect:0.0,
Method:,
Identities:193/193(100%), Positives:193/193(100%), Gaps:0/193(0%)

```
Query  522      VSLLGRASDTDLYEDLEEYKNLTPPSRVHVFRFQAPLFYANKDSFLKSLYRSVGVEPFLE  581
                VSLLGRASDTDLYEDLEEYKNLTPPSRVHVFRFQAPLFYANKDSFLKSLYRSVGVEPFLE
Sbjct  8403281  VSLLGRASDTDLYEDLEEYKNLTPPSRVHVFRFQAPLFYANKDSFLKSLYRSVGVEPFLE  8403460

Query  582      LTKRRKAEKKAKEMSAKQAAANGDKSNGEVVVGFAQRDLEFHTIVLDCSAVPFIDSTGMA  641
                LTKRRKAEKKAKEMSAKQAAANGDKSNGEVVVGFAQRDLEFHTIVLDCSAVPFIDSTGMA
Sbjct  8403461  LTKRRKAEKKAKEMSAKQAAANGDKSNGEVVVGFAQRDLEFHTIVLDCSAVPFIDSTGMA  8403640

Query  642      TFKGLLKEYKEVGVDVLLAGCNTSVIDTLRKGQFFGKNDSDMSSLLFHTVHAAVLHANSG  701
                TFKGLLKEYKEVGVDVLLAGCNTSVIDTLRKGQFFGKNDSDMSSLLFHTVHAAVLHANSG
Sbjct  8403641  TFKGLLKEYKEVGVDVLLAGCNTSVIDTLRKGQFFGKNDSDMSSLLFHTVHAAVLHANSG  8403820

Query  702      QSAVESGTHDSVV  714
                QSAVESGTHDSVV
Sbjct  8403821  QSAVESGTHDSVV  8403859
```

Range 2: 8402872 to 8403168

Score:365 bits(743), Expect:0.0,
Method:,
Identities:99/99(100%), Positives:99/99(100%), Gaps:0/99(0%)

```
Query  425      QVSSLISALVVLLVLLFFAPFFYSLQKCVLACIIIVSLRGALRKFRDVPAKWRASRNDAI  484
                QVSSLISALVVLLVLLFFAPFFYSLQKCVLACIIIVSLRGALRKFRDVPAKWRASRNDAI
Sbjct  8402872  QVSSLISALVVLLVLLFFAPFFYSLQKCVLACIIIVSLRGALRKFRDVPAKWRASRNDAI  8403051

Query  485      VWLVAMSATALISVEMGLLVGVVFSMSCIIFKTQNPKVS  523
                VWLVAMSATALISVEMGLLVGVVFSMSCIIFKTQNPKVS
Sbjct  8403052  VWLVAMSATALISVEMGLLVGVVFSMSCIIFKTQNPKVS  8403168
```

Range 3: 8401657 to 8401905

Score:310 bits(631), Expect:4e-164,
Method:,
Identities:83/83(100%), Positives:83/83(100%), Gaps:0/83(0%)

```
Query  204      QVLMAVFRLGFVSVYLSAPMLDGFATGASFTILTVQAKYLLGVKFPRHQGYGTVVVTWIN  263
                QVLMAVFRLGFVSVYLSAPMLDGFATGASFTILTVQAKYLLGVKFPRHQGYGTVVVTWIN
Sbjct  8401657  QVLMAVFRLGFVSVYLSAPMLDGFATGASFTILTVQAKYLLGVKFPRHQGYGTVVVTWIN  8401836

Query  264      IFSNIHMTNLCDLVTSAICISIL  286
                IFSNIHMTNLCDLVTSAICISIL
Sbjct  8401837  IFSNIHMTNLCDLVTSAICISIL  8401905
```

Range 4: 8401981 to 8402211

Score:277 bits(563), Expect:4e-164,
Method:,
Identities:77/77(100%), Positives:77/77(100%), Gaps:0/77(0%)

```
Query  287      VAGKELQDRYKDRLKVPLPTELLVVAGATLASHFGELNSKYGSSVSGHIPTGFIPPQVPS  346
                VAGKELQDRYKDRLKVPLPTELLVVAGATLASHFGELNSKYGSSVSGHIPTGFIPPQVPS
Sbjct  8401981  VAGKELQDRYKDRLKVPLPTELLVVAGATLASHFGELNSKYGSSVSGHIPTGFIPPQVPS  8402160

Query  347      FSLMPRVALDAIPLAVI  363
                FSLMPRVALDAIPLAVI
Sbjct  8402161  FSLMPRVALDAIPLAVI  8402211
```

Range 5: 8400211 to 8400621

Score:512 bits(1044), Expect:9e-149,
Method:,
Identities:137/137(100%), Positives:137/137(100%), Gaps:0/137(0%)

```
Query  1        MDETKLTETPLLAPSPLLERRVRQRQSTCSVLKSKLRDSATCSVSRVRSTLTGFFPVVRW  60
                MDETKLTETPLLAPSPLLERRVRQRQSTCSVLKSKLRDSATCSVSRVRSTLTGFFPVVRW
Sbjct  8400211  MDETKLTETPLLAPSPLLERRVRQRQSTCSVLKSKLRDSATCSVSRVRSTLTGFFPVVRW  8400390

Query  61       LPKYRLREYIWGDVMSGLIVGIILVPQAIAYCLLAGVEPIYGLYTSFYANIIYFLMGTSR  120
                LPKYRLREYIWGDVMSGLIVGIILVPQAIAYCLLAGVEPIYGLYTSFYANIIYFLMGTSR
Sbjct  8400391  LPKYRLREYIWGDVMSGLIVGIILVPQAIAYCLLAGVEPIYGLYTSFYANIIYFLMGTSR  8400570
```

```
Query  121      HVSVGIFSLMSLMVGQV  137
                HVSVGIFSLMSLMVGQV
Sbjct  8400571  HVSVGIFSLMSLMVGQV  8400621
```

Range 6: 8400883 to 8401092

Score:258 bits(525), Expect:3e-72,
Method:,
Identities:70/70(100%), Positives:70/70(100%), Gaps:0/70(0%)

```
Query  136      QVVDREVFLAGFDPNEDSTSSPDLFNSTLGANFTDGKVYSVELMGMQCGKECYAISIAAA  195
                QVVDREVFLAGFDPNEDSTSSPDLFNSTLGANFTDGKVYSVELMGMQCGKECYAISIAAA
Sbjct  8400883  QVVDREVFLAGFDPNEDSTSSPDLFNSTLGANFTDGKVYSVELMGMQCGKECYAISIAAA  8401062

Query  196      VTFLAGIYQV  205
                VTFLAGIYQV
Sbjct  8401063  VTFLAGIYQV  8401092
```

Range 7: 8402510 to 8402698

Score:236 bits(479), Expect:2e-65,
Method:,
Identities:63/63(100%), Positives:63/63(100%), Gaps:0/63(0%)

```
Query  364      SFAFTVSLSEMFAKKNGYTVRPNQEMLAIGLCNIIPSFFHCFTTSAALAKTMVKDSTGCQ  423
                SFAFTVSLSEMFAKKNGYTVRPNQEMLAIGLCNIIPSFFHCFTTSAALAKTMVKDSTGCQ
Sbjct  8402510  SFAFTVSLSEMFAKKNGYTVRPNQEMLAIGLCNIIPSFFHCFTTSAALAKTMVKDSTGCQ  8402689

Query  424      TQV  426
                TQV
Sbjct  8402690  TQV  8402698
```

BE Bonti rainbowfish *slc26a1*

Query: unnamed protein product Query ID: lcl|Query_2111681 Length: 705

>Telmatherina bonti genome assembly, contig: atg000131l_1, whole genome shotgun sequence
Sequence ID: CAKOGH010000126.1 Length: 605886
Range 1: 421590 to 422105

Score:310 bits(795), Expect:5e-123,
Method:Compositional matrix adjust.,
Identities:172/172(100%), Positives:172/172(100%), Gaps:0/172(0%)

```
Query  522     VSLLGQVDGTGLYEDLEEYKNLMPPNQAQIFRFHAPLYYANKESFLKSLYKAVGVEPFLE  581
               VSLLGQVDGTGLYEDLEEYKNLMPPNQAQIFRFHAPLYYANKESFLKSLYKAVGVEPFLE
Sbjct  422105  VSLLGQVDGTGLYEDLEEYKNLMPPNQAQIFRFHAPLYYANKESFLKSLYKAVGVEPFLE  421926

Query  582     LTkrrkaekkakemsakqakasGELPQRLLEFHTIILDCSAISFIDSTGLGTFKSLVKEY  641
               LTKRRKAEKKAKEMSAKQAKASGELPQRLLEFHTIILDCSAISFIDSTGLGTFKSLVKEY
Sbjct  421925  LTKRRKAEKKAKEMSAKQAKASGELPQRLLEFHTIILDCSAISFIDSTGLGTFKSLVKEY  421746

Query  642     KEIGVSVVLASCNTSVIDALQKGQFFGKDDKDMSSILFHTVHAAVLHANKVT  693
               KEIGVSVVLASCNTSVIDALQKGQFFGKDDKDMSSILFHTVHAAVLHANKVT
Sbjct  421745  KEIGVSVVLASCNTSVIDALQKGQFFGKDDKDMSSILFHTVHAAVLHANKVT  421590
```

Range 2: 422204 to 422500

Score:155 bits(392), Expect:5e-123,
Method:Compositional matrix adjust.,
Identities:98/99(99%), Positives:99/99(100%), Gaps:0/99(0%)

```
Query  424     TQVSSLITalvvllvllffapffyalQKCVLACIIIVSLRGALRKYKDVPAKWRASRNDA  483
               +QVSSLITALVVLLVLLFFAPFFYALQKCVLACIIIVSLRGALRKYKDVPAKWRASRNDA
Sbjct  422500  SQVSSLITALVVLLVLLFFAPFFYALQKCVLACIIIVSLRGALRKYKDVPAKWRASRNDA  422321

Query  484     IVWLVTMSATALISVELGLLVGIVFSMSCIIFKTQNPKV  522
               IVWLVTMSATALISVELGLLVGIVFSMSCIIFKTQNPKV
Sbjct  422320  IVWLVTMSATALISVELGLLVGIVFSMSCIIFKTQNPKV  422204
```

Range 3: 423175 to 423774

Score:314 bits(805), Expect:1e-90,
Method:Compositional matrix adjust.,
Identities:160/200(80%), Positives:160/200(80%), Gaps:40/200(20%)

```
Query  204     QIMMAVFRLGFVSVYLSAPMLDGFATGASFTILTVQAKYLLGLKIPRHQGYGTVVVTWIN  263
```

```
                QIMMAVFRLGFVSVYLSAPMLDGFATGASFTILTVQAKYLLGLKIPRHQGYGTVVVTWIN
Sbjct  423774  QIMMAVFRLGFVSVYLSAPMLDGFATGASFTILTVQAKYLLGLKIPRHQGYGTVVVTWIN  423595

Query  264     IFANIHKTNICDLISSAICILIL-------------------------------------  286
               IFANIHKTNICDLISSAICILIL
Sbjct  423594  IFANIHKTNICDLISSAICILILGLSFTFSSVLTSVFNVIILTKVFF*YLS*KI*NNTCF  423415

Query  287     ---VTGKEIQERYKDRLKIPLPTELVVVAGATLASHFGDLNTHYGSSISGHIPTGFIPPQ  343
                  VTGKEIQERYKDRLKIPLPTELVVVAGATLASHFGDLNTHYGSSISGHIPTGFIPPQ
Sbjct  423414  *FTVTGKEIQERYKDRLKIPLPTELVVVAGATLASHFGDLNTHYGSSISGHIPTGFIPPQ  423235

Query  344     VPGFSLMPRVALDAIPLAVI  363
               VPGFSLMPRVALDAIPLAVI
Sbjct  423234  VPGFSLMPRVALDAIPLAVI  423175
```

Range 4: 425065 to 425475

Score:284 bits(726), Expect:3e-80,
Method:Compositional matrix adjust.,
Identities:137/137(100%), Positives:137/137(100%), Gaps:0/137(0%)

```
Query  1       MEESSKVIGTPPAPPPILERRVRQRQPTIVSLKSKLKQNVTCSVPRLRSTLTAFFPVVRW  60
               MEESSKVIGTPPAPPPILERRVRQRQPTIVSLKSKLKQNVTCSVPRLRSTLTAFFPVVRW
Sbjct  425475  MEESSKVIGTPPAPPPILERRVRQRQPTIVSLKSKLKQNVTCSVPRLRSTLTAFFPVVRW  425296

Query  61      LPKYRLREYIWGDVMSGMIVGIILVPQAIAYCLLAGVDPIYGLYTSFYANIIYFLMGTSR  120
               LPKYRLREYIWGDVMSGMIVGIILVPQAIAYCLLAGVDPIYGLYTSFYANIIYFLMGTSR
Sbjct  425295  LPKYRLREYIWGDVMSGMIVGIILVPQAIAYCLLAGVDPIYGLYTSFYANIIYFLMGTSR  425116

Query  121     HVSVGIFSLMSLMVGQV  137
               HVSVGIFSLMSLMVGQV
Sbjct  425115  HVSVGIFSLMSLMVGQV  425065
```

Range 5: 424760 to 425014

Score:149 bits(376), Expect:2e-35,
Method:Compositional matrix adjust.,
Identities:73/85(86%), Positives:78/85(91%), Gaps:1/85(1%)

```
Query  127     FSLMSLM-VGQVVDREVFLAGFNLNEDSQSSPDILNSTLGTNITHAAIHTVELMGMQCGK  185
               F L++L    QVVDREVFLAGFNLNEDSQSSPDILNSTLGTNITHAAIHTVELMGMQCGK
Sbjct  425014  FMLLTLCSCDQVVDREVFLAGFNLNEDSQSSPDILNSTLGTNITHAAIHTVELMGMQCGK  424835

Query  186     ACYAISIAAAVTFLAGVYQIMMAVF  210
               ACYAISIAAAVTFLAGVYQ+M  ++
Sbjct  424834  ACYAISIAAAVTFLAGVYQVMKQMY  424760
```

Range 6: 422687 to 422875

Score:133 bits(335), Expect:2e-30,
Method:Compositional matrix adjust.,
Identities:63/63(100%), Positives:63/63(100%), Gaps:0/63(0%)

```
Query  364     SFAFTVSLSEMFAKKNGYSVRPNQEMLAIGLCNVIPSFFHCFTTSAALAKTMVKDSTGCQ  423
               SFAFTVSLSEMFAKKNGYSVRPNQEMLAIGLCNVIPSFFHCFTTSAALAKTMVKDSTGCQ
Sbjct  422875  SFAFTVSLSEMFAKKNGYSVRPNQEMLAIGLCNVIPSFFHCFTTSAALAKTMVKDSTGCQ  422696

Query  424     TQV  426
               TQV
Sbjct  422695  TQV  422687
```

BF Japanese medaka *slc26a1*

Query: sulfate anion transporter 1 [Oryzias latipes] Query ID: XP_011480235.1 Length: 706

>Oryzias latipes chromosome 12, ASM223467v1
Sequence ID: NC_019870.2 Length: 30543476
Range 1: 18442553 to 18443125

Score:695 bits(1418), Expect:0.0,
Method:,
Identities:191/191(100%), Positives:191/191(100%), Gaps:0/191(0%)

```
Query  516       FSLLGRVSDTDLYEDLEEYTGLTPPPQVHIFRFQAPLYYANKESFLKSLYKAVGVEPFLE  575
                 FSLLGRVSDTDLYEDLEEYTGLTPPPQVHIFRFQAPLYYANKESFLKSLYKAVGVEPFLE
Sbjct  18442553  FSLLGRVSDTDLYEDLEEYTGLTPPPQVHIFRFQAPLYYANKESFLKSLYKAVGVEPFLE  18442732

Query  576       LTKRRKAEKAKGKSSKQAKANKKNGDVFIGPIHKELDFHTIVLDCSAIPFIDSSGTATLK  635
                 LTKRRKAEKAKGKSSKQAKANKKNGDVFIGPIHKELDFHTIVLDCSAIPFIDSSGTATLK
Sbjct  18442733  LTKRRKAEKAKGKSSKQAKANKKNGDVFIGPIHKELDFHTIVLDCSAIPFIDSSGTATLK  18442912
```

```
Query  636       VLVKEYKDIGVSVLLACCNASVIDALRKAQVFGKNDREMSSLLFYTVHAAVFHAGQEHAE  695
                 VLVKEYKDIGVSVLLACCNASVIDALRKAQVFGKNDREMSSLLFYTVHAAVFHAGQEHAE
Sbjct  18442913  VLVKEYKDIGVSVLLACCNASVIDALRKAQVFGKNDREMSSLLFYTVHAAVFHAGQEHAE  18443092

Query  696       AAEGKSEDSLF  706
                 AAEGKSEDSLF
Sbjct  18443093  AAEGKSEDSLF  18443125
```

Range 2: 18437175 to 18437585

Score:511 bits(1042), Expect:0.0,
Method:,
Identities:137/137(100%), Positives:137/137(100%), Gaps:0/137(0%)

```
Query  1         MDEVSKVTETPLAPPPLLARRARQPQPAISVVKSKLRKNLTCSASKVQKTLTSFFPVVRW  60
                 MDEVSKVTETPLAPPPLLARRARQPQPAISVVKSKLRKNLTCSASKVQKTLTSFFPVVRW
Sbjct  18437175  MDEVSKVTETPLAPPPLLARRARQPQPAISVVKSKLRKNLTCSASKVQKTLTSFFPVVRW  18437354

Query  61        LPKYKLKEYVWGDVMSGMIVGIILVPQAIAYCLLAGVEPIYGLYTSFYANIIYFLMGTSK  120
                 LPKYKLKEYVWGDVMSGMIVGIILVPQAIAYCLLAGVEPIYGLYTSFYANIIYFLMGTSK
Sbjct  18437355  LPKYKLKEYVWGDVMSGMIVGIILVPQAIAYCLLAGVEPIYGLYTSFYANIIYFLMGTSK  18437534

Query  121       HVSVGIFSLMSLMVGQV  137
                 HVSVGIFSLMSLMVGQV
Sbjct  18437535  HVSVGIFSLMSLMVGQV  18437585
```

Range 3: 18442179 to 18442469

Score:359 bits(731), Expect:0.0,
Method:,
Identities:97/97(100%), Positives:97/97(100%), Gaps:0/97(0%)

```
Query  419       QVSSLISALVVLLVLLFFAPFFHALQKCVLACIIIVSLRGALRKFKEVPAKWRASKNDAI  478
                 QVSSLISALVVLLVLLFFAPFFHALQKCVLACIIIVSLRGALRKFKEVPAKWRASKNDAI
Sbjct  18442179  QVSSLISALVVLLVLLFFAPFFHALQKCVLACIIIVSLRGALRKFKEVPAKWRASKNDAI  18442358

Query  479       VWLITMFATALISVELGLLVGIIFSMVCIIFKTQNPK  515
                 VWLITMFATALISVELGLLVGIIFSMVCIIFKTQNPK
Sbjct  18442359  VWLITMFATALISVELGLLVGIIFSMVCIIFKTQNPK  18442469
```

Range 4: 18437667 to 18437858

Score:235 bits(477), Expect:0.0,
Method:,
Identities:63/64(98%), Positives:64/64(100%), Gaps:0/64(0%)

```
Query  136       QVVDRELFLSGFDLNDDPTSKADSFNLTDDDPHTVDLMGIPCSKECYAISIAAAVTFLAG  195
                 QVVDRELFLSGFDLNDDPTSKADSFNLTDDDPHTVDLMGIPCSKECYAISIAAAVTFLAG
Sbjct  18437667  QVVDRELFLSGFDLNDDPTSKADSFNLTDDDPHTVDLMGIPCSKECYAISIAAAVTFLAG  18437846

Query  196       VYQI  199
                 VYQ+
Sbjct  18437847  VYQV  18437858
```

Range 5: 18440934 to 18441182

Score:312 bits(635), Expect:2e-165,
Method:,
Identities:83/83(100%), Positives:83/83(100%), Gaps:0/83(0%)

```
Query  198       QIMMAVFQLGFVSVYLSAPMLDGFATGASFTILTVQAKYLLGLKIPRHHGYGTVPVTWFN  257
                 QIMMAVFQLGFVSVYLSAPMLDGFATGASFTILTVQAKYLLGLKIPRHHGYGTVPVTWFN
Sbjct  18440934  QIMMAVFQLGFVSVYLSAPMLDGFATGASFTILTVQAKYLLGLKIPRHHGYGTVPVTWFN  18441113

Query  258       IFANIHKTNVCDLITSAICISIL  280
                 IFANIHKTNVCDLITSAICISIL
Sbjct  18441114  IFANIHKTNVCDLITSAICISIL  18441182
```

Range 6: 18441300 to 18441536

Score:280 bits(569), Expect:2e-165,
Method:,
Identities:78/79(99%), Positives:78/79(98%), Gaps:0/79(0%)

```
Query  279       ILVAGKEIQDRYKNRLKIPLPTELVVVAGATLASHFGKLNSRYSSSVSGHIPTGFIPPSV  338
                 I VAGKEIQDRYKNRLKIPLPTELVVVAGATLASHFGKLNSRYSSSVSGHIPTGFIPPSV
Sbjct  18441300  IVVAGKEIQDRYKNRLKIPLPTELVVVAGATLASHFGKLNSRYSSSVSGHIPTGFIPPSV  18441479

Query  339       PAFSLMPRVALDAIPLAVI  357
                 PAFSLMPRVALDAIPLAVI
Sbjct  18441480  PAFSLMPRVALDAIPLAVI  18441536
```

Range 7: 18441817 to 18442008

Score:237 bits(481), Expect:2e-65,
Method:,
Identities:64/64(100%), Positives:64/64(100%), Gaps:0/64(0%)

```
Query  358       SFAFTVSLSEMFAKKHGYTVRPNQEMVAIGLCNVIPSFFHSFTTSAALAKTMVKDSTGCQ  417
                 SFAFTVSLSEMFAKKHGYTVRPNQEMVAIGLCNVIPSFFHSFTTSAALAKTMVKDSTGCQ
Sbjct  18441817  SFAFTVSLSEMFAKKHGYTVRPNQEMVAIGLCNVIPSFFHSFTTSAALAKTMVKDSTGCQ  18441996

Query  418       TQVS  421
                 TQVS
Sbjct  18441997  TQVS  18442008
```

BG Mangrove rivulus *slc26a1*

Query: sulfate anion transporter 1 [Kryptolebias marmoratus] Query ID: XP_017266248.1 Length: 715

>Kryptolebias marmoratus isolate JLee-2015 linkage group LG14, ASM164957v2, whole genome shotgun sequence
Sequence ID: NC_051443.1 Length: 26915974
Range 1: 20804301 to 20804885

Score:711 bits(1451), Expect:0.0,
Method:,
Identities:194/195(99%), Positives:194/195(99%), Gaps:0/195(0%)

```
Query  521       PKVSLLGRVSDTDLYEDLEEYKNLTPPPRIKIFRFQAPLYYANKDSFLKSLYKAVGVEPF  580
                 P VSLLGRVSDTDLYEDLEEYKNLTPPPRIKIFRFQAPLYYANKDSFLKSLYKAVGVEPF
Sbjct  20804301  PQVSLLGRVSDTDLYEDLEEYKNLTPPPRIKIFRFQAPLYYANKDSFLKSLYKAVGVEPF  20804480

Query  581       LELTKRKKAEKKGKDMSKKQAKTNGEKNNCDVFIGLVQRELEFHTIVLDCSAIPFIDSTG  640
                 LELTKRKKAEKKGKDMSKKQAKTNGEKNNCDVFIGLVQRELEFHTIVLDCSAIPFIDSTG
Sbjct  20804481  LELTKRKKAEKKGKDMSKKQAKTNGEKNNCDVFIGLVQRELEFHTIVLDCSAIPFIDSTG  20804660

Query  641       MATIKGLVKEYKEIGVSTLLAGCNTLVIDILQKGQIFGNNEKNMSNYLFYTVHAAVLHAN  700
                 MATIKGLVKEYKEIGVSTLLAGCNTLVIDILQKGQIFGNNEKNMSNYLFYTVHAAVLHAN
Sbjct  20804661  MATIKGLVKEYKEIGVSTLLAGCNTLVIDILQKGQIFGNNEKNMSNYLFYTVHAAVLHAN  20804840

Query  701       QASTAAESRSEDSTV  715
                 QASTAAESRSEDSTV
Sbjct  20804841  QASTAAESRSEDSTV  20804885
```

Range 2: 20803910 to 20804206

Score:366 bits(746), Expect:0.0,
Method:,
Identities:99/99(100%), Positives:99/99(100%), Gaps:0/99(0%)

```
Query  426       QLSSLISALVVLLVLLFFAPFFSALQKCVLACIIIVSLRGALRKFKDVPAKWRASWNDAI  485
                 QLSSLISALVVLLVLLFFAPFFSALQKCVLACIIIVSLRGALRKFKDVPAKWRASWNDAI
Sbjct  20803910  QLSSLISALVVLLVLLFFAPFFSALQKCVLACIIIVSLRGALRKFKDVPAKWRASWNDAI  20804089

Query  486       VWLVTMSATALISVEIGLIIGIVFSMVCIIYKTQNPKVS  524
                 VWLVTMSATALISVEIGLIIGIVFSMVCIIYKTQNPKVS
Sbjct  20804090  VWLVTMSATALISVEIGLIIGIVFSMVCIIYKTQNPKVS  20804206
```

Range 3: 20802471 to 20802716

Score:305 bits(620), Expect:3e-166,
Method:,
Identities:82/82(100%), Positives:82/82(100%), Gaps:0/82(0%)

```
Query  206       ILMAVFRLGFVSVYLSGPMLDGFATGASFTILTVQAKYLLGLKIPRHQGYGTVVVTWINI  265
                 ILMAVFRLGFVSVYLSGPMLDGFATGASFTILTVQAKYLLGLKIPRHQGYGTVVVTWINI
Sbjct  20802471  ILMAVFRLGFVSVYLSGPMLDGFATGASFTILTVQAKYLLGLKIPRHQGYGTVVVTWINI  20802650

Query  266       FANIHKTNLCDLITSAICIAIL  287
                 FANIHKTNLCDLITSAICIAIL
Sbjct  20802651  FANIHKTNLCDLITSAICIAIL  20802716
```

Range 4: 20802809 to 20803045

Score:289 bits(589), Expect:3e-166,
Method:,
Identities:79/79(100%), Positives:79/79(100%), Gaps:0/79(0%)

```
Query  286       ILVAGKEIQDRYKDRLKIPLPTELIVVAGATLASHFGEFNTNYHSSISGHIPTGFIPPQV  345
                 ILVAGKEIQDRYKDRLKIPLPTELIVVAGATLASHFGEFNTNYHSSISGHIPTGFIPPQV
Sbjct  20802809  ILVAGKEIQDRYKDRLKIPLPTELIVVAGATLASHFGEFNTNYHSSISGHIPTGFIPPQV  20802988
```

```
Query  346       PAFSLMPRVALDAIPLAVI  364
                 PAFSLMPRVALDAIPLAVI
Sbjct  20802989  PAFSLMPRVALDAIPLAVI  20803045
```

Range 5: 20799252 to 20799662

Score:515 bits(1051), Expect:1e-149,
Method:,
Identities:137/137(100%), Positives:137/137(100%), Gaps:0/137(0%)

```
Query  1         MEEVSPVMETPMAQPFLLERRVHQRKSTVSVLKSKLKQNVTCSGPRVRSTLTGFFPVVRW  60
                 MEEVSPVMETPMAQPFLLERRVHQRKSTVSVLKSKLKQNVTCSGPRVRSTLTGFFPVVRW
Sbjct  20799252  MEEVSPVMETPMAQPFLLERRVHQRKSTVSVLKSKLKQNVTCSGPRVRSTLTGFFPVVRW  20799431

Query  61        LPKYKLKEYIWGDIMSGLIVGIILVPQAIAYCLLAGVQPIYGLLTSFYANIIYFLMGTSR  120
                 LPKYKLKEYIWGDIMSGLIVGIILVPQAIAYCLLAGVQPIYGLLTSFYANIIYFLMGTSR
Sbjct  20799432  LPKYKLKEYIWGDIMSGLIVGIILVPQAIAYCLLAGVQPIYGLLTSFYANIIYFLMGTSR  20799611

Query  121       HVSVGIFSLMSLMVGQV  137
                 HVSVGIFSLMSLMVGQV
Sbjct  20799612  HVSVGIFSLMSLMVGQV  20799662
```

Range 6: 20800637 to 20800846

Score:254 bits(516), Expect:9e-71,
Method:,
Identities:69/70(99%), Positives:70/70(100%), Gaps:0/70(0%)

```
Query  137       VVVREVFLAGFDPNEDSTPSGLVVLNDTLDTNLTDDTVLTVEVMGMQCGKECYAISVAAA  196
                 VVVREVFLAGFDPNEDSTPSGLVVLNDTLDTNLTDDTVLTVEVMGMQCGKECYAISVAAA
Sbjct  20800637  VVVREVFLAGFDPNEDSTPSGLVVLNDTLDTNLTDDTVLTVEVMGMQCGKECYAISVAAA  20800816

Query  197       VTFLAGVYQI  206
                 VTFLAGVYQ+
Sbjct  20800817  VTFLAGVYQV  20800846
```

Range 7: 20803346 to 20803531

Score:233 bits(473), Expect:2e-64,
Method:,
Identities:62/62(100%), Positives:62/62(100%), Gaps:0/62(0%)

```
Query  365       SFAFTVSLSEMFAKKNGYTVRPNQEMLAIGCCNVVPSFFHCFTTSAALAKTMVKDSTGCQ  424
                 SFAFTVSLSEMFAKKNGYTVRPNQEMLAIGCCNVVPSFFHCFTTSAALAKTMVKDSTGCQ
Sbjct  20803346  SFAFTVSLSEMFAKKNGYTVRPNQEMLAIGCCNVVPSFFHCFTTSAALAKTMVKDSTGCQ  20803525

Query  425       TQ  426
                 TQ
Sbjct  20803526  TQ  20803531
```

BH Platyfish *slc26a1*

Query: sulfate anion transporter 1-like [Xiphophorus maculatus] Query ID: XP_014326062.1 Length: 715

>Xiphophorus maculatus strain JP 163 A chromosome 8, X_maculatus-5.0-male, whole genome shotgun sequence
Sequence ID: NC_036450.1 Length: 27836836
Range 1: 10663084 to 10663656

Score:699 bits(1426), Expect:0.0,
Method:,
Identities:191/191(100%), Positives:191/191(100%), Gaps:0/191(0%)

```
Query  525       VSLLGRVNDIDLYEDVEEYKNLIAPSRVKIFRFQAPLYFANKNSFLKSLYKAVGVEPFLE  584
                 VSLLGRVNDIDLYEDVEEYKNLIAPSRVKIFRFQAPLYFANKNSFLKSLYKAVGVEPFLE
Sbjct  10663084  VSLLGRVNDIDLYEDVEEYKNLIAPSRVKIFRFQAPLYFANKNSFLKSLYKAVGVEPFLE  10663263

Query  585       LTKRKKAEKKEKDKSKKQAKTNGETTNFDVFVGLVQKELEFHTIVLDCSAIPFIDSTGMA  644
                 LTKRKKAEKKEKDKSKKQAKTNGETTNFDVFVGLVQKELEFHTIVLDCSAIPFIDSTGMA
Sbjct  10663264  LTKRKKAEKKEKDKSKKQAKTNGETTNFDVFVGLVQKELEFHTIVLDCSAIPFIDSTGMA  10663443

Query  645       TFKTLVKEYEEIGVSVLLACCNTSVIDVLQKEEFFGKNNKDISSLLFYTVHAAVLHANRA  704
                 TFKTLVKEYEEIGVSVLLACCNTSVIDVLQKEEFFGKNNKDISSLLFYTVHAAVLHANRA
Sbjct  10663444  TFKTLVKEYEEIGVSVLLACCNTSVIDVLQKEEFFGKNNKDISSLLFYTVHAAVLHANRA  10663623

Query  705       AEEKKAEDSVV  715
                 AEEKKAEDSVV
Sbjct  10663624  AEEKKAEDSVV  10663656
```

Range 2: 10659183 to 10659599

Score:522 bits(1065), Expect:0.0,
Method:,
Identities:139/139(100%), Positives:139/139(100%), Gaps:0/139(0%)

```
Query  1         MEEVSPDTETPLAPQPPRLLERRVRQQQPAVAVLKSKLKNGLTCSGPKVRSTLMGFFPVV  60
                 MEEVSPDTETPLAPQPPRLLERRVRQQQPAVAVLKSKLKNGLTCSGPKVRSTLMGFFPVV
Sbjct  10659183  MEEVSPDTETPLAPQPPRLLERRVRQQQPAVAVLKSKLKNGLTCSGPKVRSTLMGFFPVV  10659362

Query  61        RWLPKYKLKEYIWGDVMSGVIIGIILIPQAIAYCLLAGVPPIYGLYTSFYANIIYFLMGT  120
                 RWLPKYKLKEYIWGDVMSGVIIGIILIPQAIAYCLLAGVPPIYGLYTSFYANIIYFLMGT
Sbjct  10659363  RWLPKYKLKEYIWGDVMSGVIIGIILIPQAIAYCLLAGVPPIYGLYTSFYANIIYFLMGT  10659542

Query  121       SRHVSVGIFSLMSLMVGQV  139
                 SRHVSVGIFSLMSLMVGQV
Sbjct  10659543  SRHVSVGIFSLMSLMVGQV  10659599
```

Range 3: 10659698 to 10659907

Score:256 bits(521), Expect:0.0,
Method:,
Identities:69/70(99%), Positives:70/70(100%), Gaps:0/70(0%)

```
Query  139       VVVREVFRAGFDLNEDSTPSPHELFNDTLDLNFTRKPELTVELMGMQLGKDSYAITVAAA  198
                 VVVREVFRAGFDLNEDSTPSPHELFNDTLDLNFTRKPELTVELMGMQLGKDSYAITVAAA
Sbjct  10659698  VVVREVFRAGFDLNEDSTPSPHELFNDTLDLNFTRKPELTVELMGMQLGKDSYAITVAAA  10659877

Query  199       ITFLAGVYQI  208
                 ITFLAGVYQ+
Sbjct  10659878  ITFLAGVYQV  10659907
```

Range 4: 10660410 to 10660676

Score:318 bits(647), Expect:5e-168,
Method:,
Identities:86/89(97%), Positives:86/89(96%), Gaps:0/89(0%)

```
Query  201       FLAGVYQILMAVFRLGFVSIYLSAPMLDGFATGASVTILTVQAKYLLGLKIPRHQGYGTV  260
                 FL   YQILMAVFRLGFVSIYLSAPMLDGFATGASVTILTVQAKYLLGLKIPRHQGYGTV
Sbjct  10660410  FLSPLYQILMAVFRLGFVSIYLSAPMLDGFATGASVTILTVQAKYLLGLKIPRHQGYGTV  10660589

Query  261       VVTWFNIFANIHKTNLCDLITSAICITIL  289
                 VVTWFNIFANIHKTNLCDLITSAICITIL
Sbjct  10660590  VVTWFNIFANIHKTNLCDLITSAICITIL  10660676
```

Range 5: 10660760 to 10660996

Score:282 bits(573), Expect:5e-168,
Method:,
Identities:78/79(99%), Positives:78/79(98%), Gaps:0/79(0%)

```
Query  288       ILVAGKEIQDRYKDRLKIPLPTELVVVAGATLASHFGEFNTRYHSSVSGHIPTGFTPPQV  347
                 I VAGKEIQDRYKDRLKIPLPTELVVVAGATLASHFGEFNTRYHSSVSGHIPTGFTPPQV
Sbjct  10660760  ITVAGKEIQDRYKDRLKIPLPTELVVVAGATLASHFGEFNTRYHSSVSGHIPTGFTPPQV  10660939

Query  348       PAFSIMPQVALDAIPLAVI  366
                 PAFSIMPQVALDAIPLAVI
Sbjct  10660940  PAFSIMPQVALDAIPLAVI  10660996
```

Range 6: 10662496 to 10662792

Score:365 bits(744), Expect:1e-104,
Method:,
Identities:99/99(100%), Positives:99/99(100%), Gaps:0/99(0%)

```
Query  428       QVSSLISALVVLLVLLFFAPYFQALQKCVLACIIIVSLRGALRKFRDVPAKWRASRMDTI  487
                 QVSSLISALVVLLVLLFFAPYFQALQKCVLACIIIVSLRGALRKFRDVPAKWRASRMDTI
Sbjct  10662496  QVSSLISALVVLLVLLFFAPYFQALQKCVLACIIIVSLRGALRKFRDVPAKWRASRMDTI  10662675

Query  488       VWLVAMSATALISVELGLLVGIVFSMSCIIYKTQNPKVS  526
                 VWLVAMSATALISVELGLLVGIVFSMSCIIYKTQNPKVS
Sbjct  10662676  VWLVAMSATALISVELGLLVGIVFSMSCIIYKTQNPKVS  10662792
```

Range 7: 10661591 to 10661782

Score:238 bits(483), Expect:4e-66,
Method:,
Identities:64/64(100%), Positives:64/64(100%), Gaps:0/64(0%)

```
Query  367       SFAFTVSLSEMFAKKHGYSVRPNQEMVAIGLCNVVPSFFHCFTTSAALAKTMVKDSTGCQ  426
                 SFAFTVSLSEMFAKKHGYSVRPNQEMVAIGLCNVVPSFFHCFTTSAALAKTMVKDSTGCQ
```

```
Sbjct  10661591  SFAFTVSLSEMFAKKHGYSVRPNQEMVAIGLCNVVPSFFHCFTTSAALAKTMVKDSTGCQ  10661770

Query  427       TQVS  430
                 TQVS
Sbjct  10661771  TQVS  10661782
```

BI Northern snakehead *slc26a1*

Query: sulfate anion transporter 1 [Channa argus] Query ID: XP_067340996.1 Length: 708

>Channa argus isolate OARG1902GOOAL chr17_contig_1, whole genome shotgun sequence
Sequence ID: SIWR01000140.1 Length: 16312606
Range 1: 14283279 to 14283860

Score:402 bits(1033), Expect:4e-120,
Method:Compositional matrix adjust.,
Identities:193/194(99%), Positives:194/194(100%), Gaps:0/194(0%)

```
Query  515       KVSLLGRANNSDIYEDVDEYKNLTLTPRVQVLRFQAPLYYANKDSFLKSLHKAVGVEPFL  574
                 +VSLLGRANNSDIYEDVDEYKNLTLTPRVQVLRFQAPLYYANKDSFLKSLHKAVGVEPFL
Sbjct  14283279  QVSLLGRANNSDIYEDVDEYKNLTLTPRVQVLRFQAPLYYANKDSFLKSLHKAVGVEPFL  14283458

Query  575       EMSMRKKAEKQAKETSLRQAKANGDKNNGEVAIGLVQRELDFHTIVLDCSAIPFIDSAGM  634
                 EMSMRKKAEKQAKETSLRQAKANGDKNNGEVAIGLVQRELDFHTIVLDCSAIPFIDSAGM
Sbjct  14283459  EMSMRKKAEKQAKETSLRQAKANGDKNNGEVAIGLVQRELDFHTIVLDCSAIPFIDSAGM  14283638

Query  635       AAFKGLVKEYKEIGVNVLLASCNTSVIDTLQKGQFFGKNGKDMSNLLFHTVHGAVLHAIS  694
                 AAFKGLVKEYKEIGVNVLLASCNTSVIDTLQKGQFFGKNGKDMSNLLFHTVHGAVLHAIS
Sbjct  14283639  AAFKGLVKEYKEIGVNVLLASCNTSVIDTLQKGQFFGKNGKDMSNLLFHTVHGAVLHAIS  14283818

Query  695       MHASADSSPEDSMV  708
                 MHASADSSPEDSMV
Sbjct  14283819  MHASADSSPEDSMV  14283860
```

Range 2: 14280076 to 14280513

Score:267 bits(683), Expect:5e-74,
Method:Compositional matrix adjust.,
Identities:139/146(95%), Positives:142/146(97%), Gaps:4/146(2%)

```
Query  1         MEKVNKVTEITTAPPLLLERRVHKQEPklsllksklRQSATCSIPRVRSTLTGVFPVVHW  60
                 MEKVNKVTEITTAPPLLLERRVHKQEPKLSLLKSKLRQSATCSIPRVRSTLTGVFPVVHW
Sbjct  14280076  MEKVNKVTEITTAPPLLLERRVHKQEPKLSLLKSKLRQSATCSIPRVRSTLTGVFPVVHW  14280255

Query  61        LPKYKVREYICGDVMSGMIIGIILVPQAIAYCLLAGVEPIYGLYTSFYANIIYFLMGTSR  120
                 LPKYKVREYICGDVMSGMIIGIILVPQAIAYCLLAGVEPIYGLYTSFYANIIYFLMGTSR
Sbjct  14280256  LPKYKVREYICGDVMSGMIIGIILVPQAIAYCLLAGVEPIYGLYTSFYANIIYFLMGTSR  14280435

Query  121       HVSVGIFSLMSLMVGQVV----DKEM  142
                 HVSVGIFSLMSLMVGQV+    DK++
Sbjct  14280436  HVSVGIFSLMSLMVGQVIRNTNDKQL  14280513
```

Range 3: 14281250 to 14281507

Score:174 bits(442), Expect:4e-43,
Method:Compositional matrix adjust.,
Identities:86/88(98%), Positives:86/88(97%), Gaps:2/88(2%)

```
Query  198       QVMMALFQLGFVSVYLSAPMLDGFATGASFTILTVQAKYLLGIKIPRHQGYGTVIITWIN  257
                 QVMMALFQLGFVSVYLSAPMLDGFATGASFTILTVQAKYLLGIKIPRHQGYGTVIITWIN
Sbjct  14281250  QVMMALFQLGFVSVYLSAPMLDGFATGASFTILTVQAKYLLGIKIPRHQGYGTVIITWIN  14281429

Query  258       IFYNIHKTNMCDLITSAICISILVAGKE  285
                 IFYNIHKTNMCDLITSAICISIL  GKE
Sbjct  14281430  IFYNIHKTNMCDLITSAICISIL--GKE  14281507
```

Range 4: 14281628 to 14281945

Score:163 bits(413), Expect:2e-39,
Method:Compositional matrix adjust.,
Identities:83/106(78%), Positives:87/106(82%), Gaps:8/106(7%)

```
Query  260       YNIHKTNMCD--------LITSAICISILVAGKEIQERYKDRLKIPLPTELVVVAGATLA  311
                 ++IH+ N  D         I       I+VAGKEIQERYKDRLKIPLPTELVVVAGATLA
Sbjct  14281628  FDIHQINTGDETAIQYFFYIKQHYVF*IVVAGKEIQERYKDRLKIPLPTELVVVAGATLA  14281807

Query  312       SHFGELNSHYGSSVSGHIPTGFIPPQVPAFSLMSRVALDAIPLAVI  357
                 SHFGELNSHYGSSVSGHIPTGFIPPQVPAFSLMSRVALDAIPLAVI
Sbjct  14281808  SHFGELNSHYGSSVSGHIPTGFIPPQVPAFSLMSRVALDAIPLAVI  14281945
```

```
Range 5: 14282883 to 14283125

Score:154 bits(388), Expect:2e-36,
Method:Compositional matrix adjust.,
Identities:76/81(94%), Positives:76/81(93%), Gaps:0/81(0%)

Query  444       QKCVLACIIIVSLRGALRKFKDIPAKWQSSKNDAVVWLVTMSATALISVELGLLIGIVFS  503
                 QKCVLACIIIVSLRGALRKFKDIPAKWQSSKNDAVVWLVTMSATALISVELGLLIGIVFS
Sbjct  14282883  QKCVLACIIIVSLRGALRKFKDIPAKWQSSKNDAVVWLVTMSATALISVELGLLIGIVFS  14283062

Query  504       MICIIFKTHKPKVSLLGRANN  524
                 MICIIFKTHKPKVS L   N
Sbjct  14283063  MICIIFKTHKPKVSQLLDVNQ  14283125
```

```
Range 6: 14280695 to 14280916

Score:135 bits(341), Expect:1e-30,
Method:Compositional matrix adjust.,
Identities:67/74(91%), Positives:69/74(93%), Gaps:0/74(0%)

Query  131       SLMVGQVVDKEMFLAGFDLSEDVLNGSMSTNVTAGHVHSVELMGLQCEKECYAISIAAAL  190
                 +L + QVVDKEMFLAGFDLSEDVLNGSMSTNVTAGHVHSVELMGLQCEKECYAISIAAAL
Sbjct  14280695  NLFLVQVVDKEMFLAGFDLSEDVLNGSMSTNVTAGHVHSVELMGLQCEKECYAISIAAAL  14280874

Query  191       TFLAGVYQVMMALF  204
                 TFLAGVYQV   LF
Sbjct  14280875  TFLAGVYQVNEQLF  14280916
```

```
Range 7: 14282188 to 14282373

Score:133 bits(334), Expect:1e-29,
Method:Compositional matrix adjust.,
Identities:62/62(100%), Positives:62/62(100%), Gaps:0/62(0%)

Query  358       SFAFTVSLSEMFAKKNGYTVRPNQEMLAIGFCNIIPSFFHCFTTSAALAKTMVKDSTGCK  417
                 SFAFTVSLSEMFAKKNGYTVRPNQEMLAIGFCNIIPSFFHCFTTSAALAKTMVKDSTGCK
Sbjct  14282188  SFAFTVSLSEMFAKKNGYTVRPNQEMLAIGFCNIIPSFFHCFTTSAALAKTMVKDSTGCK  14282367

Query  418       TQ  419
                 TQ
Sbjct  14282368  TQ  14282373
```

BJ Swamp eel *slc26a1*

```
Query: sulfate transporter-like, partial [Monopterus albus] Query ID: XP_020465534.1 Length: 700


>Monopterus albus unplaced genomic scaffold, M_albus_1.0 scaffold102.1, whole genome shotgun sequence
Sequence ID: NW_018127982.1 Length: 1772076
Range 1: 224899 to 225477

Score:703 bits(1435), Expect:0.0,
Method:,
Identities:193/193(100%), Positives:193/193(100%), Gaps:0/193(0%)

Query  508     VSLLGQANDTALYEDVSEYKNLMLPPRVHVLRFQAPLYYANKDSFLKSLYKAVGVEPFLE  567
               VSLLGQANDTALYEDVSEYKNLMLPPRVHVLRFQAPLYYANKDSFLKSLYKAVGVEPFLE
Sbjct  225477  VSLLGQANDTALYEDVSEYKNLMLPPRVHVLRFQAPLYYANKDSFLKSLYKAVGVEPFLE  225298

Query  568     ITKRRKAEKKAKEMSIKQAKANGNKNDGEVITVLVKRELGFHTIVLDCSAIPFMDSTGMA  627
               ITKRRKAEKKAKEMSIKQAKANGNKNDGEVITVLVKRELGFHTIVLDCSAIPFMDSTGMA
Sbjct  225297  ITKRRKAEKKAKEMSIKQAKANGNKNDGEVITVLVKRELGFHTIVLDCSAIPFMDSTGMA  225118

Query  628     TFKALVKEYKEIGVSVLLASCNTSVIDTLQKGQFFGKNNEDMSSLLFHTVHAAVLHARNT  687
               TFKALVKEYKEIGVSVLLASCNTSVIDTLQKGQFFGKNNEDMSSLLFHTVHAAVLHARNT
Sbjct  225117  TFKALVKEYKEIGVSVLLASCNTSVIDTLQKGQFFGKNNEDMSSLLFHTVHAAVLHARNT  224938

Query  688     SAAAESRSNDSVV  700
               SAAAESRSNDSVV
Sbjct  224937  SAAAESRSNDSVV  224899
```

```
Range 2: 229117 to 229479

Score:457 bits(932), Expect:2e-132,
Method:,
Identities:121/121(100%), Positives:121/121(100%), Gaps:0/121(0%)

Query  1       HLLERQVRQRQPAVSVLKYKLKRGVTCSVPKVRSTLTGFFPVLRWLPKYKLREYVWGDVM  60
               HLLERQVRQRQPAVSVLKYKLKRGVTCSVPKVRSTLTGFFPVLRWLPKYKLREYVWGDVM
Sbjct  229479  HLLERQVRQRQPAVSVLKYKLKRGVTCSVPKVRSTLTGFFPVLRWLPKYKLREYVWGDVM  229300
```

```
Query  61      SGMIIGIILVPQAIAYCLLAGVEPIYGLYTSFYANIIYFLMGTSRHVSVGIFSLMSLMVG  120
               SGMIIGIILVPQAIAYCLLAGVEPIYGLYTSFYANIIYFLMGTSRHVSVGIFSLMSLMVG
Sbjct  229299  SGMIIGIILVPQAIAYCLLAGVEPIYGLYTSFYANIIYFLMGTSRHVSVGIFSLMSLMVG  229120

Query  121     Q  121
               Q
Sbjct  229119  Q  229117
```

Range 3: 225633 to 225929

Score:363 bits(740), Expect:4e-104,
Method:,
Identities:99/99(100%), Positives:99/99(100%), Gaps:0/99(0%)

```
Query  411     QVSSLISALVVLFVLLFLAPFFYSLQKCVLACIIIVSLRGALRKLKDIPAKCCAGQNDAI  470
               QVSSLISALVVLFVLLFLAPFFYSLQKCVLACIIIVSLRGALRKLKDIPAKCCAGQNDAI
Sbjct  225929  QVSSLISALVVLFVLLFLAPFFYSLQKCVLACIIIVSLRGALRKLKDIPAKCCAGQNDAI  225750

Query  471     VWLVTMSATALISVELGILIGIVFSMSCFIFKTQKPKVS  509
               VWLVTMSATALISVELGILIGIVFSMSCFIFKTQKPKVS
Sbjct  225749  VWLVTMSATALISVELGILIGIVFSMSCFIFKTQKPKVS  225633
```

Range 4: 227455 to 227703

Score:312 bits(636), Expect:1e-88,
Method:,
Identities:83/83(100%), Positives:83/83(100%), Gaps:0/83(0%)

```
Query  190     QVMMAVFRLGFVSVYLSAPMLDGFATGASFTILTVQAKYLLGLKFPRHQGYGIVIITWIN  249
               QVMMAVFRLGFVSVYLSAPMLDGFATGASFTILTVQAKYLLGLKFPRHQGYGIVIITWIN
Sbjct  227703  QVMMAVFRLGFVSVYLSAPMLDGFATGASFTILTVQAKYLLGLKFPRHQGYGIVIITWIN  227524

Query  250     IFTNIHKTNLCDLITSAICISIL  272
               IFTNIHKTNLCDLITSAICISIL
Sbjct  227523  IFTNIHKTNLCDLITSAICISIL  227455
```

Range 5: 227011 to 227247

Score:285 bits(581), Expect:1e-80,
Method:,
Identities:78/79(99%), Positives:78/79(98%), Gaps:0/79(0%)

```
Query  271     ILVAGKEIQEHYKDRLKIPLPTELVVMAGATLASHFGQLNSHYDSSISGHIPTGFIPPRQ  330
               I VAGKEIQEHYKDRLKIPLPTELVVMAGATLASHFGQLNSHYDSSISGHIPTGFIPPRQ
Sbjct  227247  IIVAGKEIQEHYKDRLKIPLPTELVVMAGATLASHFGQLNSHYDSSISGHIPTGFIPPRQ  227068

Query  331     PSFSLMSQVALDAIPLAVI  349
               PSFSLMSQVALDAIPLAVI
Sbjct  227067  PSFSLMSQVALDAIPLAVI  227011
```

Range 6: 228545 to 228775

Score:263 bits(536), Expect:6e-74,
Method:,
Identities:75/78(96%), Positives:75/78(96%), Gaps:1/78(1%)

```
Query  121     QVVDREVFLAGFDLNEDSKPSGPDVLNGSMDINFTVGQLHSVELMGLQCSKECYAISIAA  180
               QVVDREVFLAGFDLNEDSKPSGPDVLNGSMDINFTVGQLHSVELMGLQCSKECYAISIAA
Sbjct  228775  QVVDREVFLAGFDLNEDSKPSGPDVLNGSMDINFTVGQLHSVELMGLQCSKECYAISIAA  228596

Query  181     AVTFLAGVYQVMMAVFRL  198
               AVTFLAGVYQV  AVF L
Sbjct  228595  AVTFLAGVYQV-TAVFWL  228545
```

Range 7: 226462 to 226650

Score:235 bits(478), Expect:2e-65,
Method:,
Identities:63/63(100%), Positives:63/63(100%), Gaps:0/63(0%)

```
Query  350     SFAFTVSLSEMFAKKNGYTVRPNQEMLAIGFCNIIPSSFHCFTTSAALAKTMVKDSTGCQ  409
               SFAFTVSLSEMFAKKNGYTVRPNQEMLAIGFCNIIPSSFHCFTTSAALAKTMVKDSTGCQ
Sbjct  226650  SFAFTVSLSEMFAKKNGYTVRPNQEMLAIGFCNIIPSSFHCFTTSAALAKTMVKDSTGCQ  226471

Query  410     TQV  412
               TQV
Sbjct  226470  TQV  226462
```

BK Greater amberjack *slc26a1*

Query: sulfate anion transporter 1-like isoform X2 [Seriola dumerili] Query ID: XP_022622298.1 Length: 715

>Seriola dumerili isolate Sdu_G_001 unplaced genomic scaffold, Sdu_1.0 BDQW01000874.1, whole genome shotgun sequence
Sequence ID: NW_019175129.1 Length: 938565
Range 1: 817060 to 817635

Score:707 bits(1442), Expect:0.0,
Method:,
Identities:192/192(100%), Positives:192/192(100%), Gaps:0/192(0%)

```
Query  524     VSLLGRAKDSDLYEDMDEYKNLMPPPRVQVFRFQAPLYFANKDTFLKSLYKAVGVEPFLE  583
               VSLLGRAKDSDLYEDMDEYKNLMPPPRVQVFRFQAPLYFANKDTFLKSLYKAVGVEPFLE
Sbjct  817635  VSLLGRAKDSDLYEDMDEYKNLMPPPRVQVFRFQAPLYFANKDTFLKSLYKAVGVEPFLE  817456

Query  584     MTKRRKAEKKAKEMSLKQAKANGDKNNGEVVIGLVKRELEFHTIVLDCAAIPFIDSTGMA  643
               MTKRRKAEKKAKEMSLKQAKANGDKNNGEVVIGLVKRELEFHTIVLDCAAIPFIDSTGMA
Sbjct  817455  MTKRRKAEKKAKEMSLKQAKANGDKNNGEVVIGLVKRELEFHTIVLDCAAIPFIDSTGMA  817276

Query  644     TLKGLVKEYKEIGACILLASCNTSVIDTLQKGQFFGKDDKDMSSLLFHTVHTAVLYANST  703
               TLKGLVKEYKEIGACILLASCNTSVIDTLQKGQFFGKDDKDMSSLLFHTVHTAVLYANST
Sbjct  817275  TLKGLVKEYKEIGACILLASCNTSVIDTLQKGQFFGKDDKDMSSLLFHTVHTAVLYANST  817096

Query  704     FAALERSEDSVV  715
               FAALERSEDSVV
Sbjct  817095  FAALERSEDSVV  817060
```

Range 2: 819960 to 820370

Score:512 bits(1044), Expect:0.0,
Method:,
Identities:137/137(100%), Positives:137/137(100%), Gaps:0/137(0%)

```
Query  1       MEEVTNVTETTPALPPLLERRVRQRQPTVSVLKSKLKQGVTCSVPRVRSTLTGFFPVVKW  60
               MEEVTNVTETTPALPPLLERRVRQRQPTVSVLKSKLKQGVTCSVPRVRSTLTGFFPVVKW
Sbjct  820370  MEEVTNVTETTPALPPLLERRVRQRQPTVSVLKSKLKQGVTCSVPRVRSTLTGFFPVVKW  820191

Query  61      LPKYKLREYVWGDVMSGVIVGIILVPQAIAYCLLAGVEPIYGLYTSFYANIIYFLMGTSR  120
               LPKYKLREYVWGDVMSGVIVGIILVPQAIAYCLLAGVEPIYGLYTSFYANIIYFLMGTSR
Sbjct  820190  LPKYKLREYVWGDVMSGVIVGIILVPQAIAYCLLAGVEPIYGLYTSFYANIIYFLMGTSR  820011

Query  121     HVSVGIFSLMSLMVGQV  137
               HVSVGIFSLMSLMVGQV
Sbjct  820010  HVSVGIFSLMSLMVGQV  819960
```

Range 3: 819666 to 819878

Score:260 bits(528), Expect:0.0,
Method:,
Identities:71/71(100%), Positives:71/71(100%), Gaps:0/71(0%)

```
Query  137     VVDREVFLAGFDLNDDFKASALPDVFNGSIDTNLTAGKLHTVELMGQQCGKECYAISIAA  196
               VVDREVFLAGFDLNDDFKASALPDVFNGSIDTNLTAGKLHTVELMGQQCGKECYAISIAA
Sbjct  819878  VVDREVFLAGFDLNDDFKASALPDVFNGSIDTNLTAGKLHTVELMGQQCGKECYAISIAA  819699

Query  197     AVTFLAGIYQV  207
               AVTFLAGIYQV
Sbjct  819698  AVTFLAGIYQV  819666
```

Range 4: 817760 to 818053

Score:364 bits(741), Expect:4e-104,
Method:,
Identities:98/98(100%), Positives:98/98(100%), Gaps:0/98(0%)

```
Query  428     VSSLISAMVVLLVLLFFAPFFYALQKCVLACIIIVSLRGALRKFKDVPAKWRASRNDAIV  487
               VSSLISAMVVLLVLLFFAPFFYALQKCVLACIIIVSLRGALRKFKDVPAKWRASRNDAIV
Sbjct  818053  VSSLISAMVVLLVLLFFAPFFYALQKCVLACIIIVSLRGALRKFKDVPAKWRASRNDAIV  817874

Query  488     WLVAMSATALISVELGLLVGIVFSMMCIIYKTQNPKVS  525
               WLVAMSATALISVELGLLVGIVFSMMCIIYKTQNPKVS
Sbjct  817873  WLVAMSATALISVELGLLVGIVFSMMCIIYKTQNPKVS  817760
```

Range 5: 819291 to 819539

Score:310 bits(631), Expect:7e-88,
Method:,
Identities:83/83(100%), Positives:83/83(100%), Gaps:0/83(0%)

```
Query  206     QVMMAVFRLGFVSVYLSAPMLDGFATGASFTILTVQAKYLLGLKIPRHQGYGTVVVTWIN  265
               QVMMAVFRLGFVSVYLSAPMLDGFATGASFTILTVQAKYLLGLKIPRHQGYGTVVVTWIN
Sbjct  819539  QVMMAVFRLGFVSVYLSAPMLDGFATGASFTILTVQAKYLLGLKIPRHQGYGTVVVTWIN  819360
```

```
Query  266     IFANIHKTNLCDLITSAICISIL  288
               IFANIHKTNLCDLITSAICISIL
Sbjct  819359  IFANIHKTNLCDLITSAICISIL  819291
```

Range 6: 818897 to 819133

Score:281 bits(572), Expect:3e-79,
Method:,
Identities:78/79(99%), Positives:78/79(98%), Gaps:0/79(0%)

```
Query  287     ILVAGKEIQERYKDRLKIPLPTELVVVAGATLASHFGELNSLYGSSVSGHIPTGFIPPQV  346
               I VAGKEIQERYKDRLKIPLPTELVVVAGATLASHFGELNSLYGSSVSGHIPTGFIPPQV
Sbjct  819133  IIVAGKEIQERYKDRLKIPLPTELVVVAGATLASHFGELNSLYGSSVSGHIPTGFIPPQV  818954

Query  347     PRFGLMSRVALDAIPLAVI  365
               PRFGLMSRVALDAIPLAVI
Sbjct  818953  PRFGLMSRVALDAIPLAVI  818897
```

Range 7: 818510 to 818698

Score:237 bits(482), Expect:7e-66,
Method:,
Identities:63/63(100%), Positives:63/63(100%), Gaps:0/63(0%)

```
Query  366     SFAFTVSLSEMFAKKNGYTVRPNQEMLAIGCCNIIPSFFHCFTTSAALAKTMVKDSTGCQ  425
               SFAFTVSLSEMFAKKNGYTVRPNQEMLAIGCCNIIPSFFHCFTTSAALAKTMVKDSTGCQ
Sbjct  818698  SFAFTVSLSEMFAKKNGYTVRPNQEMLAIGCCNIIPSFFHCFTTSAALAKTMVKDSTGCQ  818519

Query  426     TQV  428
               TQV
Sbjct  818518  TQV  818510
```

BL Japanese lates *slc26a1*

Query: sulfate anion transporter 1-like protein [Lates japonicus] Query ID: GLD55153.1 Length: 712

>Lates japonicus DNA, scaffold_20, whole genome shotgun sequence
Sequence ID: BRZM01000021.1 Length: 9467204
Range 1: 1883910 to 1884479

Score:697 bits(1422), Expect:0.0,
Method:,
Identities:190/190(100%), Positives:190/190(100%), Gaps:0/190(0%)

```
Query  523      VSLLGRVNDCDLYEDVDEYKNLMPPPRVKIFRFQAPLYFANKDSFLKSLYKAVGVEPFLE  582
                VSLLGRVNDCDLYEDVDEYKNLMPPPRVKIFRFQAPLYFANKDSFLKSLYKAVGVEPFLE
Sbjct  1884479  VSLLGRVNDCDLYEDVDEYKNLMPPPRVKIFRFQAPLYFANKDSFLKSLYKAVGVEPFLE  1884300

Query  583      LTKRRKAEKKAKEISSKQAKENGETNNGEVVIGLVLEFHTIVLDCSAIPFIDSTGMAMFN  642
                LTKRRKAEKKAKEISSKQAKENGETNNGEVVIGLVLEFHTIVLDCSAIPFIDSTGMAMFN
Sbjct  1884299  LTKRRKAEKKAKEISSKQAKENGETNNGEVVIGLVLEFHTIVLDCSAIPFIDSTGMAMFN  1884120

Query  643      RLVKEYKEIGVSVLLASCNTSVIDTLQKGQFFGKNDKDMSSLLFHTVHAAVLYAKSTHAA  702
                RLVKEYKEIGVSVLLASCNTSVIDTLQKGQFFGKNDKDMSSLLFHTVHAAVLYAKSTHAA
Sbjct  1884119  RLVKEYKEIGVSVLLASCNTSVIDTLQKGQFFGKNDKDMSSLLFHTVHAAVLYAKSTHAA  1883940

Query  703      AESRLEDSGV  712
                AESRLEDSGV
Sbjct  1883939  AESRLEDSGV  1883910
```

Range 2: 1887084 to 1887497

Score:513 bits(1047), Expect:0.0,
Method:,
Identities:137/138(99%), Positives:138/138(100%), Gaps:0/138(0%)

```
Query  1        MEEVPKPTETTPSLPPLLERRVRQRQPKVSVLKSKLKQGVTCSVPKVRSTLTGFFPVVRW  60
                MEEVPKPTETTPSLPPLLERRVRQRQPKVSVLKSKLKQGVTCSVPKVRSTLTGFFPVVRW
Sbjct  1887497  MEEVPKPTETTPSLPPLLERRVRQRQPKVSVLKSKLKQGVTCSVPKVRSTLTGFFPVVRW  1887318

Query  61       LPKYKLREYVWGDVMSGLIVGIILVPQAIAYCLLAGVEPIYGLYTSFYANIIYFLMGTSR  120
                LPKYKLREYVWGDVMSGLIVGIILVPQAIAYCLLAGVEPIYGLYTSFYANIIYFLMGTSR
Sbjct  1887317  LPKYKLREYVWGDVMSGLIVGIILVPQAIAYCLLAGVEPIYGLYTSFYANIIYFLMGTSR  1887138

Query  121      HVSVGIFSLMSLMVGQVV  138
                HVSVGIFSLMSLMVGQV+
Sbjct  1887137  HVSVGIFSLMSLMVGQVI  1887084
```

Range 3: 1886789 to 1887001

Score:259 bits(526), Expect:0.0,
Method:,
Identities:71/71(100%), Positives:71/71(100%), Gaps:0/71(0%)

```
Query  136      QVVDREVFLAGFDLNEDSKASGPDVFNGSMGTNLTAGELHSVELMGLQCGKECYAISIAA  195
                QVVDREVFLAGFDLNEDSKASGPDVFNGSMGTNLTAGELHSVELMGLQCGKECYAISIAA
Sbjct  1887001  QVVDREVFLAGFDLNEDSKASGPDVFNGSMGTNLTAGELHSVELMGLQCGKECYAISIAA  1886822

Query  196      ALTFLTGVYQV  206
                ALTFLTGVYQV
Sbjct  1886821  ALTFLTGVYQV  1886789
```

Range 4: 1884656 to 1884952

Score:365 bits(743), Expect:5e-105,
Method:,
Identities:99/99(100%), Positives:99/99(100%), Gaps:0/99(0%)

```
Query  426      QVSSLISALVVLLVLLFFAPFFYSLQKCVLACIIIVSLRGALRKFKDVPAKWRASQNDAI  485
                QVSSLISALVVLLVLLFFAPFFYSLQKCVLACIIIVSLRGALRKFKDVPAKWRASQNDAI
Sbjct  1884952  QVSSLISALVVLLVLLFFAPFFYSLQKCVLACIIIVSLRGALRKFKDVPAKWRASQNDAI  1884773

Query  486      VWLVAMSATALISVELGLLVGIIFSMICIIYKTQNPKVS  524
                VWLVAMSATALISVELGLLVGIIFSMICIIYKTQNPKVS
Sbjct  1884772  VWLVAMSATALISVELGLLVGIIFSMICIIYKTQNPKVS  1884656
```

Range 5: 1886397 to 1886645

Score:312 bits(636), Expect:3e-89,
Method:,
Identities:83/83(100%), Positives:83/83(100%), Gaps:0/83(0%)

```
Query  205      QVMMAVFQLGFVSVYLSAPMLDGFATGASFTILTVQAKYLLGLKIPRHQGYGTVIVTWIN  264
                QVMMAVFQLGFVSVYLSAPMLDGFATGASFTILTVQAKYLLGLKIPRHQGYGTVIVTWIN
Sbjct  1886645  QVMMAVFQLGFVSVYLSAPMLDGFATGASFTILTVQAKYLLGLKIPRHQGYGTVIVTWIN  1886466

Query  265      IFTNIHKTNLCDLITSAICIFIL  287
                IFTNIHKTNLCDLITSAICIFIL
Sbjct  1886465  IFTNIHKTNLCDLITSAICIFIL  1886397
```

Range 6: 1885977 to 1886213

Score:280 bits(570), Expect:2e-79,
Method:,
Identities:78/79(99%), Positives:78/79(98%), Gaps:0/79(0%)

```
Query  286      ILVAGKEIQERYKDRLKIPLPTELVVVAGATLASHFGELNSVYGSSVSGHIPTGFIPPQV  345
                I VAGKEIQERYKDRLKIPLPTELVVVAGATLASHFGELNSVYGSSVSGHIPTGFIPPQV
Sbjct  1886213  IIVAGKEIQERYKDRLKIPLPTELVVVAGATLASHFGELNSVYGSSVSGHIPTGFIPPQV  1886034

Query  346      PSFGLMSRVALDAIPLAVI  364
                PSFGLMSRVALDAIPLAVI
Sbjct  1886033  PSFGLMSRVALDAIPLAVI  1885977
```

Range 7: 1885341 to 1885541

Score:239 bits(485), Expect:7e-67,
Method:,
Identities:66/68(97%), Positives:66/68(97%), Gaps:1/68(1%)

```
Query  365      SFAFTVSLSEMFAKKNGYTVRPNQEMLAIGCCNIIPSFFHCFTTSAALAKTMVKDSTGCQ  424
                SFAFTVSLSEMFAKKNGYTVRPNQEMLAIGCCNIIPSFFHCFTTSAALAKTMVKDSTGCQ
Sbjct  1885541  SFAFTVSLSEMFAKKNGYTVRPNQEMLAIGCCNIIPSFFHCFTTSAALAKTMVKDSTGCQ  1885362

Query  425      TQVSSLIS  432
                TQV  LIS
Sbjct  1885361  TQV-NLIS  1885341
```

BM Turbot *slc26a1*

Query: sulfate anion transporter 1 [Scophthalmus maximus] Query ID: XP_035463328.2 Length: 711

>Scophthalmus maximus strain ysfricsl-2021 chromosome 20, ASM2237912v1, whole genome shotgun sequence
Sequence ID: NC_061534.1 Length: 18467213
Range 1: 5701275 to 5701850

Score:706 bits(1441), Expect:0.0,
Method:,
Identities:192/192(100%), Positives:192/192(100%), Gaps:0/192(0%)

```
Query  520      VSLMGRVNNSDLYEDMDEYKNLVPPPGVQVFCFQAPLYYANKDFFLKSLYGTVGVEPFLE  579
                VSLMGRVNNSDLYEDMDEYKNLVPPPGVQVFCFQAPLYYANKDFFLKSLYGTVGVEPFLE
Sbjct  5701850  VSLMGRVNNSDLYEDMDEYKNLVPPPGVQVFCFQAPLYYANKDFFLKSLYGTVGVEPFLE  5701671

Query  580      MARRRKAEKKAKEMFSKQAKANGDKNTGEVVIGLVQRELEFHTIVLDCSAIPFLDSAGLA  639
                MARRRKAEKKAKEMFSKQAKANGDKNTGEVVIGLVQRELEFHTIVLDCSAIPFLDSAGLA
Sbjct  5701670  MARRRKAEKKAKEMFSKQAKANGDKNTGEVVIGLVQRELEFHTIVLDCSAIPFLDSAGLA  5701491

Query  640      TFKGLVKEYKEVGVSVLLASCYTSVVDTLQKGQFFGKNNTDMGSLLFHTVHSAVLYANSH  699
                TFKGLVKEYKEVGVSVLLASCYTSVVDTLQKGQFFGKNNTDMGSLLFHTVHSAVLYANSH
Sbjct  5701490  TFKGLVKEYKEVGVSVLLASCYTSVVDTLQKGQFFGKNNTDMGSLLFHTVHSAVLYANSH  5701311

Query  700      RLLGNTAREANA  711
                RLLGNTAREANA
Sbjct  5701310  RLLGNTAREANA  5701275
```

Range 2: 5702009 to 5702305

Score:362 bits(738), Expect:3e-168,
Method:,
Identities:99/99(100%), Positives:99/99(100%), Gaps:0/99(0%)

```
Query  423      QVSSLISALVVLLVLLFFAPYFYALQKCVLACIIIVSLRGALRKFKDLPAKWRASRNDGI  482
                QVSSLISALVVLLVLLFFAPYFYALQKCVLACIIIVSLRGALRKFKDLPAKWRASRNDGI
Sbjct  5702305  QVSSLISALVVLLVLLFFAPYFYALQKCVLACIIIVSLRGALRKFKDLPAKWRASRNDGI  5702126

Query  483      VCLVAMSATALISVELGLLVGVVFSMICIIFKTQNPKVS  521
                VCLVAMSATALISVELGLLVGVVFSMICIIFKTQNPKVS
Sbjct  5702125  VCLVAMSATALISVELGLLVGVVFSMICIIFKTQNPKVS  5702009
```

Range 3: 5702427 to 5702615

Score:239 bits(486), Expect:3e-168,
Method:,
Identities:63/63(100%), Positives:63/63(100%), Gaps:0/63(0%)

```
Query  362      SFAFTVSLSEMFAKKNGYMVRPNQEMLAIGCCNIIPSFFHCFTTSAALAKTMVKDSTGCQ  421
                SFAFTVSLSEMFAKKNGYMVRPNQEMLAIGCCNIIPSFFHCFTTSAALAKTMVKDSTGCQ
Sbjct  5702615  SFAFTVSLSEMFAKKNGYMVRPNQEMLAIGCCNIIPSFFHCFTTSAALAKTMVKDSTGCQ  5702436

Query  422      TQV  424
                TQV
Sbjct  5702435  TQV  5702427
```

Range 4: 5703916 to 5704326

Score:514 bits(1049), Expect:3e-149,
Method:,
Identities:137/137(100%), Positives:137/137(100%), Gaps:0/137(0%)

```
Query  1        MEDVPKVSEAAMAPPPLLERRVRQRQPTVSVLKSKLKQGMTCSVPRVRSTLSGFFPVVRW  60
                MEDVPKVSEAAMAPPPLLERRVRQRQPTVSVLKSKLKQGMTCSVPRVRSTLSGFFPVVRW
Sbjct  5704326  MEDVPKVSEAAMAPPPLLERRVRQRQPTVSVLKSKLKQGMTCSVPRVRSTLSGFFPVVRW  5704147

Query  61       LPKYKLREYVWGDVMSGLIVGIILVPQAIAYCLLAGVEPIYGLYTSFYANIIYFLMGTSR  120
                LPKYKLREYVWGDVMSGLIVGIILVPQAIAYCLLAGVEPIYGLYTSFYANIIYFLMGTSR
Sbjct  5704146  LPKYKLREYVWGDVMSGLIVGIILVPQAIAYCLLAGVEPIYGLYTSFYANIIYFLMGTSR  5703967

Query  121      HVSVGIFSLMSLMVGQV  137
                HVSVGIFSLMSLMVGQV
Sbjct  5703966  HVSVGIFSLMSLMVGQV  5703916
```

Range 5: 5703178 to 5703426

Score:310 bits(632), Expect:1e-87,
Method:,
Identities:83/83(100%), Positives:83/83(100%), Gaps:0/83(0%)

```
Query  202      QVLMAVFRLGFVSVYLSAPMLDGFAMGASFTILTVQAKYLLGLKIPRHQGYGTVVVTWFN  261
                QVLMAVFRLGFVSVYLSAPMLDGFAMGASFTILTVQAKYLLGLKIPRHQGYGTVVVTWFN
Sbjct  5703426  QVLMAVFRLGFVSVYLSAPMLDGFAMGASFTILTVQAKYLLGLKIPRHQGYGTVVVTWFN  5703247

Query  262      IFANIHKTNLCDLITSAICISIL  284
                IFANIHKTNLCDLITSAICISIL
Sbjct  5703246  IFANIHKTNLCDLITSAICISIL  5703178
```

Range 6: 5702818 to 5703054

```
Score:278 bits(565), Expect:8e-78,
Method:,
Identities:78/79(99%), Positives:78/79(98%), Gaps:0/79(0%)

Query  283      ILVAGKEIQERFKDRLKIPLPTELVVVAGATLASHFGDLNSLYDSSVSGHIPTGFIAPQV  342
                I VAGKEIQERFKDRLKIPLPTELVVVAGATLASHFGDLNSLYDSSVSGHIPTGFIAPQV
Sbjct  5703054  ITVAGKEIQERFKDRLKIPLPTELVVVAGATLASHFGDLNSLYDSSVSGHIPTGFIAPQV  5702875

Query  343      PNFGLMSRVALDAIPLAVI  361
                PNFGLMSRVALDAIPLAVI
Sbjct  5702874  PNFGLMSRVALDAIPLAVI  5702818
```

```
Range 7: 5703565 to 5703768

Score:246 bits(501), Expect:2e-68,
Method:,
Identities:68/68(100%), Positives:68/68(100%), Gaps:0/68(0%)

Query  136      QVVFREVFLAGFDLNDDSIASVAGVLNSSVGTNVTLHSVELMGLQCGKECYAISVAACVT  195
                QVVFREVFLAGFDLNDDSIASVAGVLNSSVGTNVTLHSVELMGLQCGKECYAISVAACVT
Sbjct  5703768  QVVFREVFLAGFDLNDDSIASVAGVLNSSVGTNVTLHSVELMGLQCGKECYAISVAACVT  5703589

Query  196      FLAGVYQV  203
                FLAGVYQV
Sbjct  5703588  FLAGVYQV  5703565
```

BN Humphead wrasse *slc26a1*

```
Query: sulfate anion transporter 1 [Cheilinus undulatus] Query ID: XP_041666234.1 Length: 710

>Cheilinus undulatus linkage group 17, ASM1832078v1, whole genome shotgun sequence
Sequence ID: NC_054881.1 Length: 44424328
Range 1: 26794497 to 26795075

Score:715 bits(1459), Expect:0.0,
Method:,
Identities:193/193(100%), Positives:193/193(100%), Gaps:0/193(0%)

Query  518       VSLLGRANDTDLYEDIEEYKNLLPPPRIKVFRFQSPLYYANKDTFLKSLYKAVGVEPFLE  577
                 VSLLGRANDTDLYEDIEEYKNLLPPPRIKVFRFQSPLYYANKDTFLKSLYKAVGVEPFLE
Sbjct  26794497  VSLLGRANDTDLYEDIEEYKNLLPPPRIKVFRFQSPLYYANKDTFLKSLYKAVGVEPFLE  26794676

Query  578       MTKRKKAEKKAEVMSFKQAKANGDTNNGEVIVGLVQRELDFHTIVLDCSAIPFIDSTGMA  637
                 MTKRKKAEKKAEVMSFKQAKANGDTNNGEVIVGLVQRELDFHTIVLDCSAIPFIDSTGMA
Sbjct  26794677  MTKRKKAEKKAEVMSFKQAKANGDTNNGEVIVGLVQRELDFHTIVLDCSAIPFIDSTGMA  26794856

Query  638       TFKMLVREYKDIRVSVLLANCNTSVTDILQKGLFFGNNDEDMSGLLFHTVHAAVLHANSM  697
                 TFKMLVREYKDIRVSVLLANCNTSVTDILQKGLFFGNNDEDMSGLLFHTVHAAVLHANSM
Sbjct  26794857  TFKMLVREYKDIRVSVLLANCNTSVTDILQKGLFFGNNDEDMSGLLFHTVHAAVLHANSM  26795036

Query  698       FAVAENRSDDSVV  710
                 FAVAENRSDDSVV
Sbjct  26795037  FAVAENRSDDSVV  26795075
```

```
Range 2: 26789985 to 26790395

Score:511 bits(1042), Expect:2e-148,
Method:,
Identities:137/137(100%), Positives:137/137(100%), Gaps:0/137(0%)

Query  1         MEEVTKVAESTVAPQPLLERRFRQRQPAVSVLKSKLKKGVTCSGPRVRSTLTGFFPVVCW  60
                 MEEVTKVAESTVAPQPLLERRFRQRQPAVSVLKSKLKKGVTCSGPRVRSTLTGFFPVVCW
Sbjct  26789985  MEEVTKVAESTVAPQPLLERRFRQRQPAVSVLKSKLKKGVTCSGPRVRSTLTGFFPVVCW  26790164

Query  61        LPKYKLKEYVWGDVMSGLIVGIILVPQAIAYCLLAGVEPIYGLYTSFYANIIYFLMGTSR  120
                 LPKYKLKEYVWGDVMSGLIVGIILVPQAIAYCLLAGVEPIYGLYTSFYANIIYFLMGTSR
Sbjct  26790165  LPKYKLKEYVWGDVMSGLIVGIILVPQAIAYCLLAGVEPIYGLYTSFYANIIYFLMGTSR  26790344

Query  121       HVSVGIFSLMSLMVGQV  137
                 HVSVGIFSLMSLMVGQV
Sbjct  26790345  HVSVGIFSLMSLMVGQV  26790395
```

```
Range 3: 26794077 to 26794373

Score:367 bits(747), Expect:7e-105,
Method:,
Identities:99/99(100%), Positives:99/99(100%), Gaps:0/99(0%)

Query  421       QVSSLISALVVLLVLLFFAPYFYSLQKCVLACIIIVSLRGALRKFRDVPSKWRASQNDAI  480
                 QVSSLISALVVLLVLLFFAPYFYSLQKCVLACIIIVSLRGALRKFRDVPSKWRASQNDAI
Sbjct  26794077  QVSSLISALVVLLVLLFFAPYFYSLQKCVLACIIIVSLRGALRKFRDVPSKWRASQNDAI  26794256
```

```
Query  481       VWLVTMAATALISVEMGLLVGVVFSMICVIYKTQNPKVS  519
                 VWLVTMAATALISVEMGLLVGVVFSMICVIYKTQNPKVS
Sbjct  26794257  VWLVTMAATALISVEMGLLVGVVFSMICVIYKTQNPKVS  26794373
```

Range 4: 26791413 to 26791661

Score:311 bits(633), Expect:5e-88,
Method:,
Identities:83/83(100%), Positives:83/83(100%), Gaps:0/83(0%)

```
Query  200       QVLMAVFRLGFVSVYLSAPMLDGFATGASFTILTVQAKYLLGLKIPRHQGYGTVVITWIN  259
                 QVLMAVFRLGFVSVYLSAPMLDGFATGASFTILTVQAKYLLGLKIPRHQGYGTVVITWIN
Sbjct  26791413  QVLMAVFRLGFVSVYLSAPMLDGFATGASFTILTVQAKYLLGLKIPRHQGYGTVVITWIN  26791592

Query  260       IFRNIHDTNFCDVITSAICISIL  282
                 IFRNIHDTNFCDVITSAICISIL
Sbjct  26791593  IFRNIHDTNFCDVITSAICISIL  26791661
```

Range 5: 26791807 to 26792043

Score:280 bits(570), Expect:9e-79,
Method:,
Identities:78/79(99%), Positives:78/79(98%), Gaps:0/79(0%)

```
Query  281       ILVAGKEIQERYKNRLKIPLPTELVVVAGATLASHFGDLNNRYLSSVSGHIPTGFIPPQV  340
                 I VAGKEIQERYKNRLKIPLPTELVVVAGATLASHFGDLNNRYLSSVSGHIPTGFIPPQV
Sbjct  26791807  ITVAGKEIQERYKNRLKIPLPTELVVVAGATLASHFGDLNNRYLSSVSGHIPTGFIPPQV  26791986

Query  341       PSFSLMPQVALDAIPLAVI  359
                 PSFSLMPQVALDAIPLAVI
Sbjct  26791987  PSFSLMPQVALDAIPLAVI  26792043
```

Range 6: 26790551 to 26790748

Score:240 bits(489), Expect:8e-67,
Method:,
Identities:66/66(100%), Positives:66/66(100%), Gaps:0/66(0%)

```
Query  136       QVVDKELYLAGFDLNEDSKASGPDVINDTMGTNLTAVELFGVQCGKECYAISIAAALTFL  195
                 QVVDKELYLAGFDLNEDSKASGPDVINDTMGTNLTAVELFGVQCGKECYAISIAAALTFL
Sbjct  26790551  QVVDKELYLAGFDLNEDSKASGPDVINDTMGTNLTAVELFGVQCGKECYAISIAAALTFL  26790730

Query  196       AGVYQV  201
                 AGVYQV
Sbjct  26790731  AGVYQV  26790748
```

Range 7: 26793411 to 26793599

Score:237 bits(481), Expect:1e-65,
Method:,
Identities:63/63(100%), Positives:63/63(100%), Gaps:0/63(0%)

```
Query  360       SFAFTVSLSEMFAKKNGYTVRPNQEMLAIGFCNIIPSFFHCFTTSAALAKTMVKDSTGCQ  419
                 SFAFTVSLSEMFAKKNGYTVRPNQEMLAIGFCNIIPSFFHCFTTSAALAKTMVKDSTGCQ
Sbjct  26793411  SFAFTVSLSEMFAKKNGYTVRPNQEMLAIGFCNIIPSFFHCFTTSAALAKTMVKDSTGCQ  26793590

Query  420       TQV  422
                 TQV
Sbjct  26793591  TQV  26793599
```

BO Chinese perch *slc26a1*

Query: sulfate anion transporter 1 isoform X4 [Siniperca chuatsi] Query ID: XP_044029392.1 Length: 715

>Siniperca chuatsi isolate FFG_IHB_CAS linkage group LG18, ASM2008510v1, whole genome shotgun sequence
Sequence ID: NC_058059.1 Length: 28316384
Range 1: 17253617 to 17254195

Score:708 bits(1444), Expect:0.0,
Method:,
Identities:193/193(100%), Positives:193/193(100%), Gaps:0/193(0%)

```
Query  523       VSLLGRANDTDLYEDVDEYKNLMPPPRVQVFRFQAPLYYANRDSFLKSLYKAVGVEPFLE  582
                 VSLLGRANDTDLYEDVDEYKNLMPPPRVQVFRFQAPLYYANRDSFLKSLYKAVGVEPFLE
Sbjct  17253617  VSLLGRANDTDLYEDVDEYKNLMPPPRVQVFRFQAPLYYANRDSFLKSLYKAVGVEPFLE  17253796

Query  583       LTKRRKAEKKAKEMSSKQAKAKVDKNNGEVVIGLVQRELDFHTIVLDCSAIPFIDSTGMA  642
```

```
                LTKRRKAEKKAKEMSSKQAKAKVDKNNGEVVIGLVQRELDFHTIVLDCSAIPFIDSTGMA
Sbjct  17253797  LTKRRKAEKKAKEMSSKQAKAKVDKNNGEVVIGLVQRELDFHTIVLDCSAIPFIDSTGMA  17253976

Query  643       TFKGLVKEYKEIRVSVLFANCNTSVIDTLQKGQFFGKNDKEMSSLLFHTVHAAVLHANST  702
                TFKGLVKEYKEIRVSVLFANCNTSVIDTLQKGQFFGKNDKEMSSLLFHTVHAAVLHANST
Sbjct  17253977  TFKGLVKEYKEIRVSVLFANCNTSVIDTLQKGQFFGKNDKEMSSLLFHTVHAAVLHANST  17254156

Query  703       FAAAESRSEDSVV  715
                FAAAESRSEDSVV
Sbjct  17254157  FAAAESRSEDSVV  17254195
```

Range 2: 17249109 to 17249525

Score:516 bits(1052), Expect:8e-150,
Method:,
Identities:138/139(99%), Positives:138/139(99%), Gaps:0/139(0%)

```
Query  1         MEEVTKVTETTQAPPPLLERQVRQRQPTVSVLKSKLKQSVTCSVPKVRSTLTGFFPVVRW  60
                MEEVTKVTETTQAPPPLLERQVRQRQPTVSVLKSKLKQSVTCSVPKVRSTLTGFFPVVRW
Sbjct  17249109  MEEVTKVTETTQAPPPLLERQVRQRQPTVSVLKSKLKQSVTCSVPKVRSTLTGFFPVVRW  17249288

Query  61        LPKYKLREYIWGDVMSGVIVGIILVPQAIAYCLLAGVQPIYGLYTSFYANIIYFLMGTSR  120
                LPKYKLREYIWGDVMSGVIVGIILVPQAIAYCLLAGVQPIYGLYTSFYANIIYFLMGTSR
Sbjct  17249289  LPKYKLREYIWGDVMSGVIVGIILVPQAIAYCLLAGVQPIYGLYTSFYANIIYFLMGTSR  17249468

Query  121       HVSVGIFSLMGLMVGQVVD  139
                HVSVGIFSLMGLMVGQV D
Sbjct  17249469  HVSVGIFSLMGLMVGQVMD  17249525
```

Range 3: 17253127 to 17253423

Score:364 bits(742), Expect:4e-104,
Method:,
Identities:99/99(100%), Positives:99/99(100%), Gaps:0/99(0%)

```
Query  426       QVSSLISAVVVLLVLLFFAPYFYALQKCVLACIIIVSLRGALRKFKDVPGKWRASRNDAI  485
                QVSSLISAVVVLLVLLFFAPYFYALQKCVLACIIIVSLRGALRKFKDVPGKWRASRNDAI
Sbjct  17253127  QVSSLISAVVVLLVLLFFAPYFYALQKCVLACIIIVSLRGALRKFKDVPGKWRASRNDAI  17253306

Query  486       VWLVTMSATALISVELGLLVGIVFSMICVIFKTQNPKVS  524
                VWLVTMSATALISVELGLLVGIVFSMICVIFKTQNPKVS
Sbjct  17253307  VWLVTMSATALISVELGLLVGIVFSMICVIFKTQNPKVS  17253423
```

Range 4: 17251223 to 17251471

Score:307 bits(626), Expect:5e-87,
Method:,
Identities:83/83(100%), Positives:83/83(100%), Gaps:0/83(0%)

```
Query  205       QVLMAVFQLGFVSVYLSAPMLDGFATGASFTILTVQAKYLLGLKIPRHQGYGTVVVTWIN  264
                QVLMAVFQLGFVSVYLSAPMLDGFATGASFTILTVQAKYLLGLKIPRHQGYGTVVVTWIN
Sbjct  17251223  QVLMAVFQLGFVSVYLSAPMLDGFATGASFTILTVQAKYLLGLKIPRHQGYGTVVVTWIN  17251402

Query  265       IFANINETNLCDLITSAICISVL  287
                IFANINETNLCDLITSAICISVL
Sbjct  17251403  IFANINETNLCDLITSAICISVL  17251471
```

Range 5: 17251631 to 17251867

Score:280 bits(569), Expect:1e-78,
Method:,
Identities:77/79(97%), Positives:78/79(98%), Gaps:0/79(0%)

```
Query  286       VLVAGKEIQERYKDRLKIPLPTELIVVAGATLASHFGELNSRYSSSVSGHIPTGFIPPQV  345
                + VAGKEIQERYKDRLKIPLPTELIVVAGATLASHFGELNSRYSSSVSGHIPTGFIPPQV
Sbjct  17251631  IIVAGKEIQERYKDRLKIPLPTELIVVAGATLASHFGELNSRYSSSVSGHIPTGFIPPQV  17251810

Query  346       PSFFLMSRVALDAIPLAVI  364
                PSFFLMSRVALDAIPLAVI
Sbjct  17251811  PSFFLMSRVALDAIPLAVI  17251867
```

Range 6: 17249919 to 17250131

Score:262 bits(533), Expect:3e-73,
Method:,
Identities:71/71(100%), Positives:71/71(100%), Gaps:0/71(0%)

```
Query  136       QVVDKEMFLAGFDLNEDSTVYGPDVFNATLDTNLTAAKLHSVELMGQQCGRECYSISIAA  195
                QVVDKEMFLAGFDLNEDSTVYGPDVFNATLDTNLTAAKLHSVELMGQQCGRECYSISIAA
Sbjct  17249919  QVVDKEMFLAGFDLNEDSTVYGPDVFNATLDTNLTAAKLHSVELMGQQCGRECYSISIAA  17250098

Query  196       ALTFLAGVYQV  206
                ALTFLAGVYQV
```

```
Sbjct  17250099  ALTFLAGVYQV  17250131
```

```
Range 7: 17252337 to 17252525

Score:236 bits(480), Expect:2e-65,
Method:,
Identities:63/63(100%), Positives:63/63(100%), Gaps:0/63(0%)

Query  365       SFAFTVSLSEMFAKKNGYSVRPNQEMLAIGFCNIIPSFFHCFTTSAALAKTMVKDSTGCQ  424
                 SFAFTVSLSEMFAKKNGYSVRPNQEMLAIGFCNIIPSFFHCFTTSAALAKTMVKDSTGCQ
Sbjct  17252337  SFAFTVSLSEMFAKKNGYSVRPNQEMLAIGFCNIIPSFFHCFTTSAALAKTMVKDSTGCQ  17252516

Query  425       TQV  427
                 TQV
Sbjct  17252517  TQV  17252525
```

BP European seabass *slc26a1*

```
Query: sulfate anion transporter 1 [Dicentrarchus labrax] Query ID: XP_051268920.1 Length: 711


>Dicentrarchus labrax unplaced genomic scaffold, dlabrax2021, whole genome shotgun sequence
Sequence ID: NW_026136710.1 Length: 25258443
Range 1: 15857892 to 15858464

Score:708 bits(1445), Expect:0.0,
Method:,
Identities:191/191(100%), Positives:191/191(100%), Gaps:0/191(0%)

Query  521       VSLLGRANDTDLYEDMDEYKNLLPPPRVQVFRFQAPLYYANKDSFLKSLYKAVGVEPFLE  580
                 VSLLGRANDTDLYEDMDEYKNLLPPPRVQVFRFQAPLYYANKDSFLKSLYKAVGVEPFLE
Sbjct  15857892  VSLLGRANDTDLYEDMDEYKNLLPPPRVQVFRFQAPLYYANKDSFLKSLYKAVGVEPFLE  15858071

Query  581       MTIRRKAEKKAKDMSSKQAKANGDKNNGEVTIGSVELDFHTIVLDCSAIPFIDSTGMATF  640
                 MTIRRKAEKKAKDMSSKQAKANGDKNNGEVTIGSVELDFHTIVLDCSAIPFIDSTGMATF
Sbjct  15858072  MTIRRKAEKKAKDMSSKQAKANGDKNNGEVTIGSVELDFHTIVLDCSAIPFIDSTGMATF  15858251

Query  641       KGLVKEYKEIGVSVVLANCNTLVIDTLQKGQFFGKNDKDMSSMLFYTVHAAVLYANSTFA  700
                 KGLVKEYKEIGVSVVLANCNTLVIDTLQKGQFFGKNDKDMSSMLFYTVHAAVLYANSTFA
Sbjct  15858252  KGLVKEYKEIGVSVVLANCNTLVIDTLQKGQFFGKNDKDMSSMLFYTVHAAVLYANSTFA  15858431

Query  701       AAENWSENSML  711
                 AAENWSENSML
Sbjct  15858432  AAENWSENSML  15858464
```

```
Range 2: 15853199 to 15853609

Score:505 bits(1030), Expect:0.0,
Method:,
Identities:136/137(99%), Positives:136/137(99%), Gaps:0/137(0%)

Query  1         MEEVTKETTPALLPLLERRVRQRKPTVSVLKSKLKQGVTCSVPRVRSTLTGFFPVVSWLP  60
                 MEEVTKETTPALLPLLERRVRQRKPTVSVLKSKLKQGVTCSVPRVRSTLTGFFPVVSWLP
Sbjct  15853199  MEEVTKETTPALLPLLERRVRQRKPTVSVLKSKLKQGVTCSVPRVRSTLTGFFPVVSWLP  15853378

Query  61        KYKLTEYVWGDAMSGVIVGIILVPQAIAYCLLAGVKPIYGLYTSFYANIIYFLMGTSRHV  120
                 KYKLTEYVWGDAMSGVIVGIILVPQAIAYCLLAGVKPIYGLYTSFYANIIYFLMGTSRHV
Sbjct  15853379  KYKLTEYVWGDAMSGVIVGIILVPQAIAYCLLAGVKPIYGLYTSFYANIIYFLMGTSRHV  15853558

Query  121       SVGIFSLMSLMVGQVVD  137
                 SVGIFSLMSLMVGQV D
Sbjct  15853559  SVGIFSLMSLMVGQVMD  15853609
```

```
Range 3: 15853704 to 15853916

Score:260 bits(528), Expect:0.0,
Method:,
Identities:71/71(100%), Positives:71/71(100%), Gaps:0/71(0%)

Query  134       QVVDKELFLAGFDLNEESTLSGPDMFNATLESNLTAGTLHSVELMGVQCGKECYAISVAT  193
                 QVVDKELFLAGFDLNEESTLSGPDMFNATLESNLTAGTLHSVELMGVQCGKECYAISVAT
Sbjct  15853704  QVVDKELFLAGFDLNEESTLSGPDMFNATLESNLTAGTLHSVELMGVQCGKECYAISVAT  15853883

Query  194       ALTFLVGIYQV  204
                 ALTFLVGIYQV
Sbjct  15853884  ALTFLVGIYQV  15853916
```

```
Range 4: 15857416 to 15857712
```

Score:368 bits(749), Expect:8e-105,
Method:,
Identities:99/99(100%), Positives:99/99(100%), Gaps:0/99(0%)

```
Query  424       QVSSLISALVVLLVLLFFAPFFYALQKCVLASIIIVSLRGALRKFKDVPDKWRMSRNDAI  483
                 QVSSLISALVVLLVLLFFAPFFYALQKCVLASIIIVSLRGALRKFKDVPDKWRMSRNDAI
Sbjct  15857416  QVSSLISALVVLLVLLFFAPFFYALQKCVLASIIIVSLRGALRKFKDVPDKWRMSRNDAI  15857595

Query  484       VWLVTMSATALISVEMGLLVGIVFSMICIIFKTQNPKVS  522
                 VWLVTMSATALISVEMGLLVGIVFSMICIIFKTQNPKVS
Sbjct  15857596  VWLVTMSATALISVEMGLLVGIVFSMICIIFKTQNPKVS  15857712
```

Range 5: 15854574 to 15854822

Score:310 bits(632), Expect:1e-87,
Method:,
Identities:83/83(100%), Positives:83/83(100%), Gaps:0/83(0%)

```
Query  203       QVMMAVFRLGFVSVYLSAPMLDGFATGASFTILTVQAKYLLGLKIPRYQGYGTVVITWIN  262
                 QVMMAVFRLGFVSVYLSAPMLDGFATGASFTILTVQAKYLLGLKIPRYQGYGTVVITWIN
Sbjct  15854574  QVMMAVFRLGFVSVYLSAPMLDGFATGASFTILTVQAKYLLGLKIPRYQGYGTVVITWIN  15854753

Query  263       IFSNIHKTNLCDLITSAICISVL  285
                 IFSNIHKTNLCDLITSAICISVL
Sbjct  15854754  IFSNIHKTNLCDLITSAICISVL  15854822
```

Range 6: 15855078 to 15855314

Score:276 bits(562), Expect:3e-77,
Method:,
Identities:77/79(97%), Positives:78/79(98%), Gaps:0/79(0%)

```
Query  284       VLVAGKEIQERYKDRLKIPLPTELLVVAGATLASHFGELNSRYSSSVSGHIPTGFIAPQV  343
                 + VAGKEIQERYKDRLKIPLPTELLVVAGATLASHFGELNSRYSSSVSGHIPTGFIAPQV
Sbjct  15855078  IIVAGKEIQERYKDRLKIPLPTELLVVAGATLASHFGELNSRYSSSVSGHIPTGFIAPQV  15855257

Query  344       PSFVLISRVALDAIPLAII  362
                 PSFVLISRVALDAIPLAII
Sbjct  15855258  PSFVLISRVALDAIPLAII  15855314
```

Range 7: 15856768 to 15856956

Score:237 bits(482), Expect:2e-65,
Method:,
Identities:63/63(100%), Positives:63/63(100%), Gaps:0/63(0%)

```
Query  363       SFAFTVSLSEMFAKKNGYTVRPNQEMLAIGCCNIIPSFFHCFTTSAALAKTMVKDSTGCQ  422
                 SFAFTVSLSEMFAKKNGYTVRPNQEMLAIGCCNIIPSFFHCFTTSAALAKTMVKDSTGCQ
Sbjct  15856768  SFAFTVSLSEMFAKKNGYTVRPNQEMLAIGCCNIIPSFFHCFTTSAALAKTMVKDSTGCQ  15856947

Query  423       TQV  425
                 TQV
Sbjct  15856948  TQV  15856956
```

BQ Large yellow croaker *slc26a1*

Query: sulfate anion transporter 1 [Larimichthys crocea] Query ID: XP_019115085.2 Length: 713

>Larimichthys crocea isolate SSNF chromosome IV, L_crocea_2.0, whole genome shotgun sequence
Sequence ID: NC_040014.1 Length: 6444570
Range 1: 531552 to 532124

Score:703 bits(1434), Expect:0.0,
Method:,
Identities:191/191(100%), Positives:191/191(100%), Gaps:0/191(0%)

```
Query  523     VSLLGRANDTDLYEDMDEYKNLIPPPRVQVFRFQAPLYYANKDSFLKSLYKAVGVEPFLE  582
               VSLLGRANDTDLYEDMDEYKNLIPPPRVQVFRFQAPLYYANKDSFLKSLYKAVGVEPFLE
Sbjct  531552  VSLLGRANDTDLYEDMDEYKNLIPPPRVQVFRFQAPLYYANKDSFLKSLYKAVGVEPFLE  531731

Query  583     MTIRRKAEKKAKDMSLKQAKANGDRNNGEVVIGLVQRELDFHTIVLDCSAIPFIDSTGMA  642
               MTIRRKAEKKAKDMSLKQAKANGDRNNGEVVIGLVQRELDFHTIVLDCSAIPFIDSTGMA
Sbjct  531732  MTIRRKAEKKAKDMSLKQAKANGDRNNGEVVIGLVQRELDFHTIVLDCSAIPFIDSTGMA  531911

Query  643     AFEGLVKEYKEIGVSVLLANCNTSVIDTLQKGQFFGKNDKDMSSLLFHTVHAAVLHANST  702
               AFEGLVKEYKEIGVSVLLANCNTSVIDTLQKGQFFGKNDKDMSSLLFHTVHAAVLHANST
Sbjct  531912  AFEGLVKEYKEIGVSVLLANCNTSVIDTLQKGQFFGKNDKDMSSLLFHTVHAAVLHANST  532091

Query  703     FAAAETKLEVV  713
               FAAAETKLEVV
Sbjct  532092  FAAAETKLEVV  532124
```

Range 2: 528194 to 528610

Score:518 bits(1056), Expect:3e-150,
Method:,
Identities:138/139(99%), Positives:138/139(99%), Gaps:0/139(0%)

```
Query  1       MEEVTKITETTLAPPLLLERRVRQRQPKATVLKSKLKQSMTCSVPRVRSTLTGFFPVVRW  60
               MEEVTKITETTLAPPLLLERRVRQRQPKATVLKSKLKQSMTCSVPRVRSTLTGFFPVVRW
Sbjct  528194  MEEVTKITETTLAPPLLLERRVRQRQPKATVLKSKLKQSMTCSVPRVRSTLTGFFPVVRW  528373

Query  61      LPKYKLREYVWGDVMSGLIIGIILVPQAIAYCLLAGVEPIYGLYTSFYANIIYFLMGTSR  120
               LPKYKLREYVWGDVMSGLIIGIILVPQAIAYCLLAGVEPIYGLYTSFYANIIYFLMGTSR
Sbjct  528374  LPKYKLREYVWGDVMSGLIIGIILVPQAIAYCLLAGVEPIYGLYTSFYANIIYFLMGTSR  528553

Query  121     HVSVGIFSLMSLMVGQVVD  139
               HVSVGIFSLMSLMVGQV D
Sbjct  528554  HVSVGIFSLMSLMVGQVMD  528610
```

Range 3: 531082 to 531378

Score:366 bits(746), Expect:1e-104,
Method:,
Identities:99/99(100%), Positives:99/99(100%), Gaps:0/99(0%)

```
Query  426     QVSSLISALVVLLVLLFFAPFFYDLQKCVLACIIIVSLRGALRKFKDVPAKWRASRNDAI  485
               QVSSLISALVVLLVLLFFAPFFYDLQKCVLACIIIVSLRGALRKFKDVPAKWRASRNDAI
Sbjct  531082  QVSSLISALVVLLVLLFFAPFFYDLQKCVLACIIIVSLRGALRKFKDVPAKWRASRNDAI  531261

Query  486     VWLVTMSATALISVEMGLLVGIVFSMICVIFKTQKPKVS  524
               VWLVTMSATALISVEMGLLVGIVFSMICVIFKTQKPKVS
Sbjct  531262  VWLVTMSATALISVEMGLLVGIVFSMICVIFKTQKPKVS  531378
```

Range 4: 529614 to 529862

Score:309 bits(630), Expect:2e-87,
Method:,
Identities:83/83(100%), Positives:83/83(100%), Gaps:0/83(0%)

```
Query  205     QVMMAVFRLGFVSVYLSTPMLDGFATGASFTILTVQAKYLLGLKIARHQGYGTVAVTWIN  264
               QVMMAVFRLGFVSVYLSTPMLDGFATGASFTILTVQAKYLLGLKIARHQGYGTVAVTWIN
Sbjct  529614  QVMMAVFRLGFVSVYLSTPMLDGFATGASFTILTVQAKYLLGLKIARHQGYGTVAVTWIN  529793

Query  265     IFINIHKTNLCDLITSAICISVL  287
               IFINIHKTNLCDLITSAICISVL
Sbjct  529794  IFINIHKTNLCDLITSAICISVL  529862
```

Range 5: 530070 to 530306

Score:281 bits(572), Expect:7e-79,
Method:,
Identities:78/79(99%), Positives:79/79(100%), Gaps:0/79(0%)

```
Query  286     VLVAGKEIQERYKNRLKIPLPTELIVVAGATLASHFGELNSRYSSSVSGHIPTGFIPPQV  345
               +LVAGKEIQERYKNRLKIPLPTELIVVAGATLASHFGELNSRYSSSVSGHIPTGFIPPQV
Sbjct  530070  ILVAGKEIQERYKNRLKIPLPTELIVVAGATLASHFGELNSRYSSSVSGHIPTGFIPPQV  530249

Query  346     PNFGLVSRLALDAIPLAVI  364
               PNFGLVSRLALDAIPLAVI
Sbjct  530250  PNFGLVSRLALDAIPLAVI  530306
```

Range 6: 529163 to 529375

Score:258 bits(524), Expect:8e-72,
Method:,
Identities:71/71(100%), Positives:71/71(100%), Gaps:0/71(0%)

```
Query  136     QVVDKEMFLAGFDLNEDSTASSSDLFNVTLGTNLTAGKLHSVELMGVQCGKDCYSISIAS  195
               QVVDKEMFLAGFDLNEDSTASSSDLFNVTLGTNLTAGKLHSVELMGVQCGKDCYSISIAS
Sbjct  529163  QVVDKEMFLAGFDLNEDSTASSSDLFNVTLGTNLTAGKLHSVELMGVQCGKDCYSISIAS  529342

Query  196     ALTFLAGIYQV  206
               ALTFLAGIYQV
Sbjct  529343  ALTFLAGIYQV  529375
```

Range 7: 530657 to 530845

Score:237 bits(481), Expect:2e-65,
Method:,
Identities:63/63(100%), Positives:63/63(100%), Gaps:0/63(0%)

```
Query  365     SFAFTVSLSEMFAKKNGYTVRPNQEMLAIGFCNIIPSFFHCFTTSAALAKTMVKDSTGCQ  424
               SFAFTVSLSEMFAKKNGYTVRPNQEMLAIGFCNIIPSFFHCFTTSAALAKTMVKDSTGCQ
Sbjct  530657  SFAFTVSLSEMFAKKNGYTVRPNQEMLAIGFCNIIPSFFHCFTTSAALAKTMVKDSTGCQ  530836

Query  425     TQV  427
               TQV
Sbjct  530837  TQV  530845
```

BR Gilthead seabream *slc26a1*

```
Query: sulfate anion transporter 1-like isoform X7 [Sparus aurata] Query ID: XP_030292621.1 Length: 715


>Sparus aurata chromosome 12, fSpaAur1.1, whole genome shotgun sequence
Sequence ID: NC_044198.1 Length: 30193437
Range 1: 11405482 to 11406060

Score:711 bits(1451), Expect:0.0,
Method:,
Identities:193/193(100%), Positives:193/193(100%), Gaps:0/193(0%)

Query  523       VSLLGRATDTDIYEDMDEYKDLTPPPRVQVFRFQAPLYYANKDSFLKSLYKAVGVEPFLE  582
                 VSLLGRATDTDIYEDMDEYKDLTPPPRVQVFRFQAPLYYANKDSFLKSLYKAVGVEPFLE
Sbjct  11406060  VSLLGRATDTDIYEDMDEYKDLTPPPRVQVFRFQAPLYYANKDSFLKSLYKAVGVEPFLE  11405881

Query  583       MTKRSKAEKKAKDMSSKQTKANGDKNNGEVIIALVQRELDFHTIVLDCSAMPFIDSTGMA  642
                 MTKRSKAEKKAKDMSSKQTKANGDKNNGEVIIALVQRELDFHTIVLDCSAMPFIDSTGMA
Sbjct  11405880  MTKRSKAEKKAKDMSSKQTKANGDKNNGEVIIALVQRELDFHTIVLDCSAMPFIDSTGMA  11405701

Query  643       TFKGLVKEYNEINVSVLLANCNTSVIDTLQKGQFFGKNDQEISSMLFHTVHAAVLHANST  702
                 TFKGLVKEYNEINVSVLLANCNTSVIDTLQKGQFFGKNDQEISSMLFHTVHAAVLHANST
Sbjct  11405700  TFKGLVKEYNEINVSVLLANCNTSVIDTLQKGQFFGKNDQEISSMLFHTVHAAVLHANST  11405521

Query  703       FAAAESRSEDSVV  715
                 FAAAESRSEDSVV
Sbjct  11405520  FAAAESRSEDSVV  11405482



Range 2: 11406135 to 11406431

Score:364 bits(742), Expect:0.0,
Method:,
Identities:99/99(100%), Positives:99/99(100%), Gaps:0/99(0%)

Query  426       QVSSLISALVVLLVLLFFAPFFYDLQKCVLACIIIVSLRGALRKFKDVPAKWRASKNDAI  485
                 QVSSLISALVVLLVLLFFAPFFYDLQKCVLACIIIVSLRGALRKFKDVPAKWRASKNDAI
Sbjct  11406431  QVSSLISALVVLLVLLFFAPFFYDLQKCVLACIIIVSLRGALRKFKDVPAKWRASKNDAI  11406252

Query  486       VWLVAMSATALISVELGLLFGVVFSMTCIIFKTQNPKVS  524
                 VWLVAMSATALISVELGLLFGVVFSMTCIIFKTQNPKVS
Sbjct  11406251  VWLVAMSATALISVELGLLFGVVFSMTCIIFKTQNPKVS  11406135



Range 3: 11408044 to 11408292

Score:308 bits(627), Expect:3e-166,
Method:,
Identities:83/83(100%), Positives:83/83(100%), Gaps:0/83(0%)

Query  205       QLLMAVFRLGFVSVYLSSPMLDGFATGASFTILTVQAKYLLGLKIPRHQGYGTVAITWIN  264
                 QLLMAVFRLGFVSVYLSSPMLDGFATGASFTILTVQAKYLLGLKIPRHQGYGTVAITWIN
Sbjct  11408292  QLLMAVFRLGFVSVYLSSPMLDGFATGASFTILTVQAKYLLGLKIPRHQGYGTVAITWIN  11408113

Query  265       IFANIHKTNLCDLITSAICISIL  287
                 IFANIHKTNLCDLITSAICISIL
Sbjct  11408112  IFANIHKTNLCDLITSAICISIL  11408044



Range 4: 11407728 to 11407964

Score:285 bits(581), Expect:3e-166,
Method:,
Identities:79/79(100%), Positives:79/79(100%), Gaps:0/79(0%)

Query  286       ILVAGKEIQERYKDRLKIPLPTELVVVAGATLASHFGELNSRYSSSVSGHIPTGFIPPQV  345
                 ILVAGKEIQERYKDRLKIPLPTELVVVAGATLASHFGELNSRYSSSVSGHIPTGFIPPQV
Sbjct  11407964  ILVAGKEIQERYKDRLKIPLPTELVVVAGATLASHFGELNSRYSSSVSGHIPTGFIPPQV  11407785

Query  346       PSFGLMQRVALDAIPLAVI  364
                 PSFGLMQRVALDAIPLAVI
Sbjct  11407784  PSFGLMQRVALDAIPLAVI  11407728
```

Range 5: 11409010 to 11409426

Score:515 bits(1050), Expect:1e-149,
Method:,
Identities:138/139(99%), Positives:138/139(99%), Gaps:0/139(0%)

```
Query  1         MEEATKVTDTVPALPPLLERRVRQRQPTVSVLKSKLKQSATCSVPRVRSTLTGFFPVVRW  60
                 MEEATKVTDTVPALPPLLERRVRQRQPTVSVLKSKLKQSATCSVPRVRSTLTGFFPVVRW
Sbjct  11409426  MEEATKVTDTVPALPPLLERRVRQRQPTVSVLKSKLKQSATCSVPRVRSTLTGFFPVVRW  11409247

Query  61        LPKYKLREYIWGDLMSGVIVGIILVPQAIAYCLLAGVEPIYGLYTSFYANIIYFLMGTSR  120
                 LPKYKLREYIWGDLMSGVIVGIILVPQAIAYCLLAGVEPIYGLYTSFYANIIYFLMGTSR
Sbjct  11409246  LPKYKLREYIWGDLMSGVIVGIILVPQAIAYCLLAGVEPIYGLYTSFYANIIYFLMGTSR  11409067

Query  121       HVSVGIFSLMSLMVGQVVD  139
                 HVSVGIFSLMSLMVGQV D
Sbjct  11409066  HVSVGIFSLMSLMVGQVMD  11409010
```

Range 6: 11408660 to 11408869

Score:257 bits(522), Expect:8e-72,
Method:,
Identities:70/70(100%), Positives:70/70(100%), Gaps:0/70(0%)

```
Query  136       QVVDKELYLAGFDLNEDSKASVPDVFNATLGTNLTIGKPHSVELMGLQCEKECYAISIAT  195
                 QVVDKELYLAGFDLNEDSKASVPDVFNATLGTNLTIGKPHSVELMGLQCEKECYAISIAT
Sbjct  11408869  QVVDKELYLAGFDLNEDSKASVPDVFNATLGTNLTIGKPHSVELMGLQCEKECYAISIAT  11408690

Query  196       ALTFLAGIYQ  205
                 ALTFLAGIYQ
Sbjct  11408689  ALTFLAGIYQ  11408660
```

Range 7: 11407062 to 11407250

Score:236 bits(479), Expect:2e-65,
Method:,
Identities:63/63(100%), Positives:63/63(100%), Gaps:0/63(0%)

```
Query  365       SFAFTVSLSEMFAKKNGYTVRPNQEMLAISLCNIIPSFFHCFTTSAALAKTMVKDSTGCQ  424
                 SFAFTVSLSEMFAKKNGYTVRPNQEMLAISLCNIIPSFFHCFTTSAALAKTMVKDSTGCQ
Sbjct  11407250  SFAFTVSLSEMFAKKNGYTVRPNQEMLAISLCNIIPSFFHCFTTSAALAKTMVKDSTGCQ  11407071

Query  425       TQV  427
                 TQV
Sbjct  11407070  TQV  11407062
```

BS Japanese pufferfish *slc26a1*

Query: sulfate anion transporter 1-like [Takifugu rubripes] Query ID: XP_029693916.1 Length: 734

>Takifugu rubripes chromosome 6, fTakRub1.2, whole genome shotgun sequence
Sequence ID: NC_042290.1 Length: 12913240
Range 1: 11905876 to 11906451

Score:689 bits(1407), Expect:0.0,
Method:,
Identities:192/192(100%), Positives:192/192(100%), Gaps:0/192(0%)

```
Query  543       ASLLGRVHDSQLYEDVEEYRNLTPPPGVRVFRFQAPLYYANKDVFLRSLYRAVGVEPFAE  602
                 ASLLGRVHDSQLYEDVEEYRNLTPPPGVRVFRFQAPLYYANKDVFLRSLYRAVGVEPFAE
Sbjct  11906451  ASLLGRVHDSQLYEDVEEYRNLTPPPGVRVFRFQAPLYYANKDVFLRSLYRAVGVEPFAE  11906272

Query  603       LTRRKKEEKKAASLSLKHQKANGAKTNGEAAVQLKTELEFHTLVLDLSAVPFVDSSGVST  662
                 LTRRKKEEKKAASLSLKHQKANGAKTNGEAAVQLKTELEFHTLVLDLSAVPFVDSSGVST
Sbjct  11906271  LTRRKKEEKKAASLSLKHQKANGAKTNGEAAVQLKTELEFHTLVLDLSAVPFVDSSGVST  11906092

Query  663       VKGTLKEYKDIGVSVLLASCNASVIDAMREAQVFGKNDKDMSSLLFHTVHAAVLHANASF  722
                 VKGTLKEYKDIGVSVLLASCNASVIDAMREAQVFGKNDKDMSSLLFHTVHAAVLHANASF
Sbjct  11906091  VKGTLKEYKDIGVSVLLASCNASVIDAMREAQVFGKNDKDMSSLLFHTVHAAVLHANASF  11905912

Query  723       AESQSSLGDSEV  734
                 AESQSSLGDSEV
Sbjct  11905911  AESQSSLGDSEV  11905876
```

Range 2: 11908069 to 11908500

Score:534 bits(1089), Expect:0.0,
Method:,
Identities:144/144(100%), Positives:144/144(100%), Gaps:0/144(0%)

```
Query  15        SAALSRLPETMDDSAETTPALPPLLERQVRQRPSAVTALKSKLKRGVSCSVPRVRSTLTG  74
                 SAALSRLPETMDDSAETTPALPPLLERQVRQRPSAVTALKSKLKRGVSCSVPRVRSTLTG
Sbjct  11908500  SAALSRLPETMDDSAETTPALPPLLERQVRQRPSAVTALKSKLKRGVSCSVPRVRSTLTG  11908321

Query  75        LFPVVRWLPKYKLREYIWGDVMSGMIVGIILVPQAIAYCLLAGVEPIYGLYTSFYANIIY  134
                 LFPVVRWLPKYKLREYIWGDVMSGMIVGIILVPQAIAYCLLAGVEPIYGLYTSFYANIIY
Sbjct  11908320  LFPVVRWLPKYKLREYIWGDVMSGMIVGIILVPQAIAYCLLAGVEPIYGLYTSFYANIIY  11908141

Query  135       FLMGTSRHVSVGIFSLMSLMVGQV  158
                 FLMGTSRHVSVGIFSLMSLMVGQV
Sbjct  11908140  FLMGTSRHVSVGIFSLMSLMVGQV  11908069
```

Range 3: 11906527 to 11906823

Score:362 bits(738), Expect:0.0,
Method:,
Identities:98/99(99%), Positives:98/99(98%), Gaps:0/99(0%)

```
Query  446       QVSSLISALVILFILLFFAPLFYDLQKCVLACIIIVSLRGALRKFKDIPAKWRSSRNDAV  505
                 QVSSLISALVILFILLFFAPLFYDLQKCVLACIIIVSLRGALRKFKDIPAKWRSSRNDAV
Sbjct  11906823  QVSSLISALVILFILLFFAPLFYDLQKCVLACIIIVSLRGALRKFKDIPAKWRSSRNDAV  11906644

Query  506       VWLVTMAATALISVELGLLAGIVFSMICIIFKFQNPKAS  544
                 VWLVTMAATALISVELGLLAGIVFSMICIIFKFQNPK S
Sbjct  11906643  VWLVTMAATALISVELGLLAGIVFSMICIIFKFQNPKVS  11906527
```

Range 4: 11907451 to 11907699

Score:308 bits(627), Expect:0.0,
Method:,
Identities:83/83(100%), Positives:83/83(100%), Gaps:0/83(0%)

```
Query  225       QVLMAIFRLGFVSVFLSSPMLDGFATGASFTILTVQAKYLLGLKIPRHQGYGTVVITWIN  284
                 QVLMAIFRLGFVSVFLSSPMLDGFATGASFTILTVQAKYLLGLKIPRHQGYGTVVITWIN
Sbjct  11907699  QVLMAIFRLGFVSVFLSSPMLDGFATGASFTILTVQAKYLLGLKIPRHQGYGTVVITWIN  11907520

Query  285       IFSNIHKTNLCDLITSAICISVL  307
                 IFSNIHKTNLCDLITSAICISVL
Sbjct  11907519  IFSNIHKTNLCDLITSAICISVL  11907451
```

Range 5: 11907156 to 11907386

Score:279 bits(567), Expect:0.0,
Method:,
Identities:77/77(100%), Positives:77/77(100%), Gaps:0/77(0%)

```
Query  308       VAGKELQERYKDRLKIPLPTELVVVAGATLASHYGGQLNLRYSSSVSGHIPTGFIPPQVPS  367
                 VAGKELQERYKDRLKIPLPTELVVVAGATLASHYGGQLNLRYSSSVSGHIPTGFIPPQVPS
Sbjct  11907386  VAGKELQERYKDRLKIPLPTELVVVAGATLASHYGGQLNLRYSSSVSGHIPTGFIPPQVPS  11907207

Query  368       FSLMPRVALDAIPLAVI  384
                 FSLMPRVALDAIPLAVI
Sbjct  11907206  FSLMPRVALDAIPLAVI  11907156
```

Range 6: 11907795 to 11908004

Score:254 bits(516), Expect:0.0,
Method:,
Identities:70/70(100%), Positives:70/70(100%), Gaps:0/70(0%)

```
Query  157       QVVDKELFLAGFDLNEDSSPTVSEAFNATLGANLTSQVHTVELMGVPCGKECYAIGVASV  216
                 QVVDKELFLAGFDLNEDSSPTVSEAFNATLGANLTSQVHTVELMGVPCGKECYAIGVASV
Sbjct  11908004  QVVDKELFLAGFDLNEDSSPTVSEAFNATLGANLTSQVHTVELMGVPCGKECYAIGVASV  11907825

Query  217       LTFLAGIYQV  226
                 LTFLAGIYQV
Sbjct  11907824  LTFLAGIYQV  11907795
```

Range 7: 11906873 to 11907073

Score:239 bits(486), Expect:0.0,
Method:,
Identities:65/67(97%), Positives:65/67(97%), Gaps:0/67(0%)

```
Query  385       SFAFTVSLSEMFAKKNGYTVRPNQEMLAIGFCNIIPSFFHCFTTSAALAKTMVKDSTGCQ  444
                 SFAFTVSLSEMFAKKNGYTVRPNQEMLAIGFCNIIPSFFHCFTTSAALAKTMVKDSTGCQ
Sbjct  11907073  SFAFTVSLSEMFAKKNGYTVRPNQEMLAIGFCNIIPSFFHCFTTSAALAKTMVKDSTGCQ  11906894

Query  445       TQVSSLI  451
                 TQV S I
Sbjct  11906893  TQVRSHI  11906873
```

Range 8: 11910413 to 11910454

Score:52.7 bits(104), Expect:2e-10,
Method:,
Identities:14/14(100%), Positives:14/14(100%), Gaps:0/14(0%)

```
Query  1         MCRGASQVDLRLSG  14
                 MCRGASQVDLRLSG
Sbjct  11910454  MCRGASQVDLRLSG  11910413
```

BT Three-spined stickleback *slc26a1*

Query: LOW QUALITY PROTEIN: sulfate anion transporter 1 [Gasterosteus aculeatus aculeatus] Query ID: XP_040052945.1 Length: 773

>Gasterosteus aculeatus aculeatus strain Lake Benthic chromosome 14, GAculeatus_UGA_version5, whole genome shotgun sequence
Sequence ID: NC_053225.1 Length: 16147532
Range 1: 9613316 to 9613861

Score:664 bits(1355), Expect:0.0,
Method:,
Identities:182/182(100%), Positives:182/182(100%), Gaps:0/182(0%)

```
Query  592      VSLLGRASGSDLYEDLDEYENLMPPPRVQVFRFQAPLYYANKDSFLKSLYKAVGVEPFLE  651
                VSLLGRASGSDLYEDLDEYENLMPPPRVQVFRFQAPLYYANKDSFLKSLYKAVGVEPFLE
Sbjct  9613316  VSLLGRASGSDLYEDLDEYENLMPPPRVQVFRFQAPLYYANKDSFLKSLYKAVGVEPFLE  9613495

Query  652      LTKRRKAEKKAKKMSSKMVVIGLGLVQRELAFHTIVLDCSAIPFIDSTGTGTFKGLVSEF  711
                LTKRRKAEKKAKKMSSKMVVIGLGLVQRELAFHTIVLDCSAIPFIDSTGTGTFKGLVSEF
Sbjct  9613496  LTKRRKAEKKAKKMSSKMVVIGLGLVQRELAFHTIVLDCSAIPFIDSTGTGTFKGLVSEF  9613675

Query  712      KEIGVSVLLAHCNTSVIDALQKGQFFGKDDRDMSSLLFHTVHMAVLHASSASAEGKSEDC  771
                KEIGVSVLLAHCNTSVIDALQKGQFFGKDDRDMSSLLFHTVHMAVLHASSASAEGKSEDC
Sbjct  9613676  KEIGVSVLLAHCNTSVIDALQKGQFFGKDDRDMSSLLFHTVHMAVLHASSASAEGKSEDC  9613855

Query  772      LV  773
                LV
Sbjct  9613856  LV  9613861
```

Range 2: 9610508 to 9610939

Score:536 bits(1094), Expect:1e-161,
Method:,
Identities:144/144(100%), Positives:144/144(100%), Gaps:0/144(0%)

```
Query  63       FLSPRRPPLLPKTMEEVNLVTPPALERRARRRQPAVSVLKSKLKKSVTCSVPRVRSALTG  122
                FLSPRRPPLLPKTMEEVNLVTPPALERRARRRQPAVSVLKSKLKKSVTCSVPRVRSALTG
Sbjct  9610508  FLSPRRPPLLPKTMEEVNLVTPPALERRARRRQPAVSVLKSKLKKSVTCSVPRVRSALTG  9610687

Query  123      FFPVVRWLPKYKLREYIWGDLMSGVIVGIILVPQAIAYCLLAGVKPIHGLYTSFYANIIY  182
                FFPVVRWLPKYKLREYIWGDLMSGVIVGIILVPQAIAYCLLAGVKPIHGLYTSFYANIIY
Sbjct  9610688  FFPVVRWLPKYKLREYIWGDLMSGVIVGIILVPQAIAYCLLAGVKPIHGLYTSFYANIIY  9610867

Query  183      FLMGTSRHVSVGIFSLMSLMVGQV  206
                FLMGTSRHVSVGIFSLMSLMVGQV
Sbjct  9610868  FLMGTSRHVSVGIFSLMSLMVGQV  9610939
```

Range 3: 9610470 to 9610505

Score:42.4 bits(83), Expect:1e-161,
Method:,
Identities:12/12(100%), Positives:12/12(100%), Gaps:0/12(0%)

```
Query  50       GAELTTLPTSVT  61
                GAELTTLPTSVT
Sbjct  9610470  GAELTTLPTSVT  9610505
```

Range 4: 9612872 to 9613168

Score:366 bits(746), Expect:9e-105,
Method:,
Identities:99/99(100%), Positives:99/99(100%), Gaps:0/99(0%)

```
Query  495      QISSLVSALVVLLVLLFFAPCFYALQKCVLACIIIVSLRGALRKFRDVPAKWRASRTDAT  554
                QISSLVSALVVLLVLLFFAPCFYALQKCVLACIIIVSLRGALRKFRDVPAKWRASRTDAT
Sbjct  9612872  QISSLVSALVVLLVLLFFAPCFYALQKCVLACIIIVSLRGALRKFRDVPAKWRASRTDAT  9613051

Query  555      VWLVTMSATALISVELGLVVGIFFSMFCIIFETQNPKVS  593
                VWLVTMSATALISVELGLVVGIFFSMFCIIFETQNPKVS
Sbjct  9613052  VWLVTMSATALISVELGLVVGIFFSMFCIIFETQNPKVS  9613168
```

Range 5: 9611499 to 9611747

Score:309 bits(630), Expect:1e-87,
Method:,
Identities:83/83(100%), Positives:83/83(100%), Gaps:0/83(0%)

```
Query  274      QVLMAAFRLGFVSVYLSAPMLDGFATGASFTILTVQAKYLLGLKIPRHQGYGTVVVTWFN  333
                QVLMAAFRLGFVSVYLSAPMLDGFATGASFTILTVQAKYLLGLKIPRHQGYGTVVVTWFN
Sbjct  9611499  QVLMAAFRLGFVSVYLSAPMLDGFATGASFTILTVQAKYLLGLKIPRHQGYGTVVVTWFN  9611678

Query  334      IFANIHQTNMCDLITSAISIFVL  356
                IFANIHQTNMCDLITSAISIFVL
Sbjct  9611679  IFANIHQTNMCDLITSAISIFVL  9611747
```

Range 6: 9611886 to 9612116

Score:278 bits(565), Expect:4e-78,
Method:,
Identities:77/77(100%), Positives:77/77(100%), Gaps:0/77(0%)

```
Query  357      VAGKEIQERYKDRLKIPLPTELIVVAGATLASHFGELNSNYGSSVSGHIPTGFSPPQVPS  416
                VAGKEIQERYKDRLKIPLPTELIVVAGATLASHFGELNSNYGSSVSGHIPTGFSPPQVPS
Sbjct  9611886  VAGKEIQERYKDRLKIPLPTELIVVAGATLASHFGELNSNYGSSVSGHIPTGFSPPQVPS  9612065

Query  417      FSLMSRLILDAIPLAVI  433
                FSLMSRLILDAIPLAVI
Sbjct  9612066  FSLMSRLILDAIPLAVI  9612116
```

Range 7: 9611067 to 9611279

Score:260 bits(528), Expect:1e-72,
Method:,
Identities:71/71(100%), Positives:71/71(100%), Gaps:0/71(0%)

```
Query  205      QVVYKEMFLAGFDLNEDSPASGPDVVNATLGNNLTAGKLHTVELMGLQCGKECYSISIAA  264
                QVVYKEMFLAGFDLNEDSPASGPDVVNATLGNNLTAGKLHTVELMGLQCGKECYSISIAA
Sbjct  9611067  QVVYKEMFLAGFDLNEDSPASGPDVVNATLGNNLTAGKLHTVELMGLQCGKECYSISIAA  9611246

Query  265      SLTFLVGVYQV  275
                SLTFLVGVYQV
Sbjct  9611247  SLTFLVGVYQV  9611279
```

Range 8: 9612488 to 9612676

Score:234 bits(476), Expect:6e-65,
Method:,
Identities:62/63(98%), Positives:63/63(100%), Gaps:0/63(0%)

```
Query  434      SFAFTVSLSEMFAKKNGYTVRPNQEMLAIGFCNIIPSFFHCFTTSAALAKTMVKDSTGCQ  493
                SFAFTVSLSEMFAKKNGYTVRPNQEMLAIGFCNIIPSFFHCFTTSAALAKTMVKDSTGCQ
Sbjct  9612488  SFAFTVSLSEMFAKKNGYTVRPNQEMLAIGFCNIIPSFFHCFTTSAALAKTMVKDSTGCQ  9612667

Query  494      TQI  496
                TQ+
Sbjct  9612668  TQV  9612676
```

Range 9: 9605281 to 9605430

Score:187 bits(380), Expect:9e-51,
Method:,
Identities:50/50(100%), Positives:50/50(100%), Gaps:0/50(0%)

```
Query  1        MLCGMKQEEEHEHPSGRLHRQTQSDAACRGTNRGAMADVASRSLLWASLG  50
                MLCGMKQEEEHEHPSGRLHRQTQSDAACRGTNRGAMADVASRSLLWASLG
Sbjct  9605281  MLCGMKQEEEHEHPSGRLHRQTQSDAACRGTNRGAMADVASRSLLWASLG  9605430
```

BU Tristan klipfish *slc26a1*

Query: unnamed protein product Query ID: lcl|Query_6755959 Length: 709

>Bovichtus diacanthus isolate 2004_03 flattened_line_11004, whole genome shotgun sequence
Sequence ID: QZNB01005511.1 Length: 27969
Range 1: 2859 to 3437

Score:709 bits(1447), Expect:0.0,
Method:,
Identities:193/193(100%), Positives:193/193(100%), Gaps:0/193(0%)

```
Query  517   VSLLGRTNDTDLYEDVDEYQNLMPPPRVQVFRFQAPLYYANKDSFLRSLYKAVGVEPFLE  576
             VSLLGRTNDTDLYEDVDEYQNLMPPPRVQVFRFQAPLYYANKDSFLRSLYKAVGVEPFLE
Sbjct  3437  VSLLGRTNDTDLYEDVDEYQNLMPPPRVQVFRFQAPLYYANKDSFLRSLYKAVGVEPFLE  3258

Query  577   LTKRRKAEKKAKEMSSKQAKANGDKSNGEVVIGLVQRELDFHTIVLDCSAIPFIDSTGMS  636
             LTKRRKAEKKAKEMSSKQAKANGDKSNGEVVIGLVQRELDFHTIVLDCSAIPFIDSTGMS
Sbjct  3257  LTKRRKAEKKAKEMSSKQAKANGDKSNGEVVIGLVQRELDFHTIVLDCSAIPFIDSTGMS  3078

Query  637   TFGGLVKEYKEIRVNVLFANCNTSVIDILQKGQLFGENDKDMSSRLFYTVHAAVVHANSS  696
             TFGGLVKEYKEIRVNVLFANCNTSVIDILQKGQLFGENDKDMSSRLFYTVHAAVVHANSS
Sbjct  3077  TFGGLVKEYKEIRVNVLFANCNTSVIDILQKGQLFGENDKDMSSRLFYTVHAAVVHANSS  2898

Query  697   YAAAESRSEDSVV  709
             YAAAESRSEDSVV
Sbjct  2897  YAAAESRSEDSVV  2859
```

Range 2: 3555 to 3851

Score:366 bits(745), Expect:0.0,
Method:,
Identities:99/99(100%), Positives:99/99(100%), Gaps:0/99(0%)

```
Query  420   QVSSLISALVVLLVLLFFAPFFYALQKCVLACIIIVSLRGALRKFKDVPAKCRASRNDGI  479
             QVSSLISALVVLLVLLFFAPFFYALQKCVLACIIIVSLRGALRKFKDVPAKCRASRNDGI
Sbjct  3851  QVSSLISALVVLLVLLFFAPFFYALQKCVLACIIIVSLRGALRKFKDVPAKCRASRNDGI  3672

Query  480   VWLVTMSATALISVEMGLLVGIVFSMMCVIFKTQNPKVS  518
             VWLVTMSATALISVEMGLLVGIVFSMMCVIFKTQNPKVS
Sbjct  3671  VWLVTMSATALISVEMGLLVGIVFSMMCVIFKTQNPKVS  3555
```

Range 3: 4802 to 5050

Score:308 bits(627), Expect:2e-160,
Method:,
Identities:83/83(100%), Positives:83/83(100%), Gaps:0/83(0%)

```
Query  199   QVLMAVFRLGFVSVYLSAPMLDGFATGASFTILTVQAKYLLGLKIPRHQGYGTVVVTWIN  258
             QVLMAVFRLGFVSVYLSAPMLDGFATGASFTILTVQAKYLLGLKIPRHQGYGTVVVTWIN
Sbjct  5050  QVLMAVFRLGFVSVYLSAPMLDGFATGASFTILTVQAKYLLGLKIPRHQGYGTVVVTWIN  4871

Query  259   IFANIHNTNLCDLITSAICISVL  281
             IFANIHNTNLCDLITSAICISVL
Sbjct  4870  IFANIHNTNLCDLITSAICISVL  4802
```

Range 4: 4487 to 4723

Score:283 bits(576), Expect:2e-160,
Method:,
Identities:78/79(99%), Positives:79/79(100%), Gaps:0/79(0%)

```
Query  280   VLVAGKEIQERYKDRLKIPLPTELVIVAGATLASHFGELNSHYGSSVSGHIPTGFITPQV  339
             +LVAGKEIQERYKDRLKIPLPTELVIVAGATLASHFGELNSHYGSSVSGHIPTGFITPQV
Sbjct  4723  ILVAGKEIQERYKDRLKIPLPTELVIVAGATLASHFGELNSHYGSSVSGHIPTGFITPQV  4544

Query  340   PSFSLMPRLALDAIPLAVI  358
             PSFSLMPRLALDAIPLAVI
Sbjct  4543  PSFSLMPRLALDAIPLAVI  4487
```

Range 5: 5937 to 6335

Score:494 bits(1008), Expect:3e-138,
Method:,
Identities:132/133(99%), Positives:132/133(99%), Gaps:0/133(0%)

```
Query  1     MEEVTKVPLPVLERRARQRQPTVSVLKSKLKHGVTCSVPRVRSTLSGFFPVVRWLPKYKL  60
             MEEVTKVPLPVLERRARQRQPTVSVLKSKLKHGVTCSVPRVRSTLSGFFPVVRWLPKYKL
Sbjct  6335  MEEVTKVPLPVLERRARQRQPTVSVLKSKLKHGVTCSVPRVRSTLSGFFPVVRWLPKYKL  6156

Query  61    REYVWGDVMSGVIIGIILVPQAIAYCLLAGVEPIYGLYTSFYANIIYFLMGTSRHVSVGI  120
             REYVWGDVMSGVIIGIILVPQAIAYCLLAGVEPIYGLYTSFYANIIYFLMGTSRHVSVGI
Sbjct  6155  REYVWGDVMSGVIIGIILVPQAIAYCLLAGVEPIYGLYTSFYANIIYFLMGTSRHVSVGI  5976

Query  121   FSLMSLMVGQVVD  133
             FSLMSLMVGQV D
Sbjct  5975  FSLMSLMVGQVMD  5937
```

Range 6: 5245 to 5457

Score:261 bits(530), Expect:8e-68,

```
Method:,
Identities:71/71(100%), Positives:71/71(100%), Gaps:0/71(0%)

Query  130   QVVDKEMFLAGFDLNDDSTASGPDVFNATLGTNVTTGTLHSVELMGLQCGKECYSISIAA  189
             QVVDKEMFLAGFDLNDDSTASGPDVFNATLGTNVTTGTLHSVELMGLQCGKECYSISIAA
Sbjct  5457  QVVDKEMFLAGFDLNDDSTASGPDVFNATLGTNVTTGTLHSVELMGLQCGKECYSISIAA  5278

Query  190   ALTFMVGVYQV  200
             ALTFMVGVYQV
Sbjct  5277  ALTFMVGVYQV  5245
```

```
Range 7: 4042 to 4236

Score:240 bits(488), Expect:1e-61,
Method:,
Identities:64/65(98%), Positives:64/65(98%), Gaps:0/65(0%)

Query  359   SFAFTVSLSEMFAKKNGYTVRPNQEMLAIGFCNIIPSFFHCFTTSAALAKTMVKDSTGCH  418
             SFAFTVSLSEMFAKKNGYTVRPNQEMLAIGFCNIIPSFFHCFTTSAALAKTMVKDSTGCH
Sbjct  4236  SFAFTVSLSEMFAKKNGYTVRPNQEMLAIGFCNIIPSFFHCFTTSAALAKTMVKDSTGCH  4057

Query  419   TQVSS  423
             TQV S
Sbjct  4056  TQVNS  4042
```

BV Patagonian blennie *slc26a1*

```
Query: unnamed protein product Query ID: lcl|Query_7966423 Length: 708


>Eleginops maclovinus isolate 2004_01 flattened_line_17216, whole genome shotgun sequence
Sequence ID: QZNA01008631.1 Length: 15525
Range 1: 14630 to 15208

Score:711 bits(1451), Expect:0.0,
Method:,
Identities:193/193(100%), Positives:193/193(100%), Gaps:0/193(0%)

Query  516    VSLLGKASEIDLYEDVEEYKNLMPPPRVQIFRFQAPLYYANKDSFLRSLYKAVGVEPFLE  575
              VSLLGKASEIDLYEDVEEYKNLMPPPRVQIFRFQAPLYYANKDSFLRSLYKAVGVEPFLE
Sbjct  14630  VSLLGKASEIDLYEDVEEYKNLMPPPRVQIFRFQAPLYYANKDSFLRSLYKAVGVEPFLE  14809

Query  576    LTKRRKEEKKAQEMSSKQIKANGDKSNGEVVIGLVQRELDFHTIVLDCSAIPFIDSTGMA  635
              LTKRRKEEKKAQEMSSKQIKANGDKSNGEVVIGLVQRELDFHTIVLDCSAIPFIDSTGMA
Sbjct  14810  LTKRRKEEKKAQEMSSKQIKANGDKSNGEVVIGLVQRELDFHTIVLDCSAIPFIDSTGMA  14989

Query  636    TFEGLVKEFGEIRVNILLANCNTSVIDILQKGQFFGKNDKDMSSRLFHTVHAAVLHAKEL  695
              TFEGLVKEFGEIRVNILLANCNTSVIDILQKGQFFGKNDKDMSSRLFHTVHAAVLHAKEL
Sbjct  14990  TFEGLVKEFGEIRVNILLANCNTSVIDILQKGQFFGKNDKDMSSRLFHTVHAAVLHAKEL  15169

Query  696    YAAAESRSEDSEV  708
              YAAAESRSEDSEV
Sbjct  15170  YAAAESRSEDSEV  15208
```

```
Range 2: 14211 to 14507

Score:363 bits(739), Expect:4e-162,
Method:,
Identities:99/99(100%), Positives:99/99(100%), Gaps:0/99(0%)

Query  419    QISSLISALVVLLVLLFFAPYFFALQKCVLACIIIVSLRGALRKFKDVPAKLRASRDDGI  478
              QISSLISALVVLLVLLFFAPYFFALQKCVLACIIIVSLRGALRKFKDVPAKLRASRDDGI
Sbjct  14211  QISSLISALVVLLVLLFFAPYFFALQKCVLACIIIVSLRGALRKFKDVPAKLRASRDDGI  14390

Query  479    VWMVAMSATALISVELGLVVGVVFSMICVIYKTQNPKVS  517
              VWMVAMSATALISVELGLVVGVVFSMICVIYKTQNPKVS
Sbjct  14391  VWMVAMSATALISVELGLVVGVVFSMICVIYKTQNPKVS  14507
```

```
Range 3: 13907 to 14095

Score:234 bits(476), Expect:4e-162,
Method:,
Identities:62/63(98%), Positives:63/63(100%), Gaps:0/63(0%)

Query  358    SFAFTVSLSEMFAKKNGYTVRPNQEMLAISFCNIIPSFFHCFTTSAALAKTMVKDSTGCQ  417
              SFAFTVSLSEMFAKKNGYTVRPNQEMLAISFCNIIPSFFHCFTTSAALAKTMVKDSTGCQ
Sbjct  13907  SFAFTVSLSEMFAKKNGYTVRPNQEMLAISFCNIIPSFFHCFTTSAALAKTMVKDSTGCQ  14086

Query  418    TQI  420
              TQ+
Sbjct  14087  TQV  14095
```

Range 4: 11952 to 12350

Score:492 bits(1003), Expect:1e-137,
Method:,
Identities:131/133(98%), Positives:132/133(99%), Gaps:0/133(0%)

```
Query  1      MEEVTKVPPPILERRVRQRQTTVSVLKSKLKQGVTCSVPRVRSTLTGFFPVVRWLPKYKL  60
              MEEVTKVPPPILERRVRQRQTTVSVLKSKLKQGVTCSVPRVRSTLTGFFPVVRWLPKYKL
Sbjct  11952  MEEVTKVPPPILERRVRQRQTTVSVLKSKLKQGVTCSVPRVRSTLTGFFPVVRWLPKYKL  12131

Query  61     QEYVWGDLMSGVIVGIILVPQAIAYCLLAGVQPIYGLYTSFYANIIYFLMGTSRHVSVGI  120
              QEYVWGDLMSGVIVGIILVPQAIAYCLLAGVQPIYGLYTSFYANIIYFLMGTSRHVSVGI
Sbjct  12132  QEYVWGDLMSGVIVGIILVPQAIAYCLLAGVQPIYGLYTSFYANIIYFLMGTSRHVSVGI  12311

Query  121    FSLMSLMVGQVVD  133
              FSLMSLMVGQV +
Sbjct  12312  FSLMSLMVGQVME  12350
```

Range 5: 12923 to 13171

Score:309 bits(630), Expect:1e-82,
Method:,
Identities:83/83(100%), Positives:83/83(100%), Gaps:0/83(0%)

```
Query  198    QLLMAVFRLGFVSVYLSAPMLDGFATGASFTILTVQAKYLLGIKIPRHQGYGTVVVTWFN  257
              QLLMAVFRLGFVSVYLSAPMLDGFATGASFTILTVQAKYLLGIKIPRHQGYGTVVVTWFN
Sbjct  12923  QLLMAVFRLGFVSVYLSAPMLDGFATGASFTILTVQAKYLLGIKIPRHQGYGTVVVTWFN  13102

Query  258    IFANIHNTNLCDLITSAICISIL  280
              IFANIHNTNLCDLITSAICISIL
Sbjct  13103  IFANIHNTNLCDLITSAICISIL  13171
```

Range 6: 13326 to 13556

Score:279 bits(568), Expect:2e-73,
Method:,
Identities:77/77(100%), Positives:77/77(100%), Gaps:0/77(0%)

```
Query  281    VAGKEIQERYKDRLKIPLPTELIIVAGATLASHFGELNSKYGSSVSGHIPTGFIPPQVPS  340
              VAGKEIQERYKDRLKIPLPTELIIVAGATLASHFGELNSKYGSSVSGHIPTGFIPPQVPS
Sbjct  13326  VAGKEIQERYKDRLKIPLPTELIIVAGATLASHFGELNSKYGSSVSGHIPTGFIPPQVPS  13505

Query  341    FTLMPRLALDAIPLAVI  357
              FTLMPRLALDAIPLAVI
Sbjct  13506  FTLMPRLALDAIPLAVI  13556
```

Range 7: 12561 to 12764

Score:250 bits(508), Expect:1e-64,
Method:,
Identities:68/68(100%), Positives:68/68(100%), Gaps:0/68(0%)

```
Query  131    VVDKEMFMAGFDLDEDSKASGLVFNASLGTNLTIGTPHSVELMGVQCGKECYSITIAAAL  190
              VVDKEMFMAGFDLDEDSKASGLVFNASLGTNLTIGTPHSVELMGVQCGKECYSITIAAAL
Sbjct  12561  VVDKEMFMAGFDLDEDSKASGLVFNASLGTNLTIGTPHSVELMGVQCGKECYSITIAAAL  12740

Query  191    TFLVGVYQ  198
              TFLVGVYQ
Sbjct  12741  TFLVGVYQ  12764
```

BW Emerald rockcod *slc26a1*

Query: sulfate anion transporter 1 [Trematomus bernacchii] Query ID: XP_033979372.1 Length: 708

>Trematomus bernacchii unplaced genomic scaffold, fTreBer1.1, whole genome shotgun sequence
Sequence ID: NW_022987547.1 Length: 14399040
Range 1: 305712 to 306290

Score:709 bits(1446), Expect:0.0,
Method:,
Identities:193/193(100%), Positives:193/193(100%), Gaps:0/193(0%)

```
Query  516     VSLLGKASDVDLYEDVEEYKNLMPPPRVQIFRFQAPLYYANKDSFLRSLYKAVGVEPFLE  575
               VSLLGKASDVDLYEDVEEYKNLMPPPRVQIFRFQAPLYYANKDSFLRSLYKAVGVEPFLE
Sbjct  306290  VSLLGKASDVDLYEDVEEYKNLMPPPRVQIFRFQAPLYYANKDSFLRSLYKAVGVEPFLE  306111

Query  576     LTKRRKEEKKAKEMSSKQIKANGDKSNGEVVIGLVERELDFHTIVLDCSVIPFIDSTGMA  635
               LTKRRKEEKKAKEMSSKQIKANGDKSNGEVVIGLVERELDFHTIVLDCSVIPFIDSTGMA
```

Sbjct 306110 LTKRRKEEKKAKEMSSKQIKANGDKSNGEVVIGLVERELDFHTIVLDCSVIPFIDSTGMA 305931

Query 636   TFEGLVKEYGEIRVNVLLANCNTSVIDLLQKGQFFGKNDKDMSSRLFHTVHAAVLHAKDS 695
               TFEGLVKEYGEIRVNVLLANCNTSVIDLLQKGQFFGKNDKDMSSRLFHTVHAAVLHAKDS
Sbjct 305930 TFEGLVKEYGEIRVNVLLANCNTSVIDLLQKGQFFGKNDKDMSSRLFHTVHAAVLHAKDS 305751

Query 696   YAAAESRSEDSGV 708
               YAAAESRSEDSGV
Sbjct 305750 YAAAESRSEDSGV 305712

Range 2: 306368 to 306667

Score:368 bits(749), Expect:0.0,
Method:,
Identities:100/100(100%), Positives:100/100(100%), Gaps:0/100(0%)

Query 418   TQISSLISALVVLLVLLFFAPYFYALQKCVLACIIIVSLRGALRKFKDVPAKLRASRDDG 477
               TQISSLISALVVLLVLLFFAPYFYALQKCVLACIIIVSLRGALRKFKDVPAKLRASRDDG
Sbjct 306667 TQISSLISALVVLLVLLFFAPYFYALQKCVLACIIIVSLRGALRKFKDVPAKLRASRDDG 306488

Query 478   IVWLVAMFATTLISVEIGLVVGVVFSMICIIYKTQNPKVS 517
               IVWLVAMFATTLISVEIGLVVGVVFSMICIIYKTQNPKVS
Sbjct 306487 IVWLVAMFATTLISVEIGLVVGVVFSMICIIYKTQNPKVS 306368

Range 3: 309706 to 310104

Score:488 bits(995), Expect:3e-141,
Method:,
Identities:131/133(98%), Positives:132/133(99%), Gaps:0/133(0%)

Query 1   MEEVTKVPPPVLERQVRQRQTTVSVLKSKLKHGVTCSVPKVRSTLTGFFPVVRWLPKYKL 60
               MEEVTKVPPPVLERQVRQRQTTVSVLKSKLKHGVTCSVPKVRSTLTGFFPVVRWLPKYKL
Sbjct 310104 MEEVTKVPPPVLERQVRQRQTTVSVLKSKLKHGVTCSVPKVRSTLTGFFPVVRWLPKYKL 309925

Query 61   KEYVLGDLMSGVIVGIILVPQAIAYCLLAGVPPIYGLYTSFYANIIYFLMGTSRHVSVGI 120
               KEYVLGDLMSGVIVGIILVPQAIAYCLLAGVPPIYGLYTSFYANIIYFLMGTSRHVSVGI
Sbjct 309924 KEYVLGDLMSGVIVGIILVPQAIAYCLLAGVPPIYGLYTSFYANIIYFLMGTSRHVSVGI 309745

Query 121   FSLMSLMVGQVVN 133
               FSLMSLMVGQV +
Sbjct 309744 FSLMSLMVGQVMD 309706

Range 4: 308790 to 309038

Score:309 bits(630), Expect:2e-87,
Method:,
Identities:83/83(100%), Positives:83/83(100%), Gaps:0/83(0%)

Query 198   QLLMAVFRLGFVSVYLSSPMLDGFATGASFTILTVQAKYLLGIKIPRHQGYGTVVVTWFN 257
               QLLMAVFRLGFVSVYLSSPMLDGFATGASFTILTVQAKYLLGIKIPRHQGYGTVVVTWFN
Sbjct 309038 QLLMAVFRLGFVSVYLSSPMLDGFATGASFTILTVQAKYLLGIKIPRHQGYGTVVVTWFN 308859

Query 258   IFANIHNTNLCDLITSAICISIL 280
               IFANIHNTNLCDLITSAICISIL
Sbjct 308858 IFANIHNTNLCDLITSAICISIL 308790

Range 5: 308410 to 308646

Score:287 bits(585), Expect:9e-81,
Method:,
Identities:79/79(100%), Positives:79/79(100%), Gaps:0/79(0%)

Query 279   ILVAGKEIQERYKDRLKIPFPTELIIVAGATLASHFGDLNSKYGSSVSGHIPTGFIPPEV 338
               ILVAGKEIQERYKDRLKIPFPTELIIVAGATLASHFGDLNSKYGSSVSGHIPTGFIPPEV
Sbjct 308646 ILVAGKEIQERYKDRLKIPFPTELIIVAGATLASHFGDLNSKYGSSVSGHIPTGFIPPEV 308467

Query 339   PSFTLMPRLALDAIPLAVI 357
               PSFTLMPRLALDAIPLAVI
Sbjct 308466 PSFTLMPRLALDAIPLAVI 308410

Range 6: 309195 to 309398

Score:247 bits(503), Expect:1e-68,
Method:,
Identities:68/68(100%), Positives:68/68(100%), Gaps:0/68(0%)

Query 131   VVNKEMFLAGFDLNEDSKASGLVLNASLGSNLTIGSPHSVELMGVPCGKECYSISIAAAL 190
               VVNKEMFLAGFDLNEDSKASGLVLNASLGSNLTIGSPHSVELMGVPCGKECYSISIAAAL
Sbjct 309398 VVNKEMFLAGFDLNEDSKASGLVLNASLGSNLTIGSPHSVELMGVPCGKECYSISIAAAL 309219

Query 191   TFMVGVYQ 198
               TFMVGVYQ
Sbjct 309218 TFMVGVYQ 309195

Range 7: 306868 to 307056

Score:234 bits(476), Expect:1e-64,
Method:,
Identities:62/63(98%), Positives:63/63(100%), Gaps:0/63(0%)

```
Query  358     SFAFTVSLSEMFAKKNGYTVRPNQEMLAISFCNIIPSFFHCFTTSAALAKTMVKDSTGCQ  417
               SFAFTVSLSEMFAKKNGYTVRPNQEMLAISFCNIIPSFFHCFTTSAALAKTMVKDSTGCQ
Sbjct  307056  SFAFTVSLSEMFAKKNGYTVRPNQEMLAISFCNIIPSFFHCFTTSAALAKTMVKDSTGCQ  306877

Query  418     TQI  420
               TQ+
Sbjct  306876  TQV  306868
```

BX Marbled rockcod *slc26a1*

Query: unnamed protein product Query ID: lcl|Query_8069441 Length: 708

>Notothenia rossii genome assembly, contig: atg002301l_1, whole genome shotgun sequence
Sequence ID: CATIUU010002033.1 Length: 150056
Range 1: 48574 to 49152

Score:708 bits(1444), Expect:0.0,
Method:,
Identities:193/193(100%), Positives:193/193(100%), Gaps:0/193(0%)

```
Query  516    VSLLGKASDVDLYEDVEEYKNLMPPPRVQIFRFQAPLYYANKDSFLRSLYKAVGVEPFLE  575
              VSLLGKASDVDLYEDVEEYKNLMPPPRVQIFRFQAPLYYANKDSFLRSLYKAVGVEPFLE
Sbjct  48574  VSLLGKASDVDLYEDVEEYKNLMPPPRVQIFRFQAPLYYANKDSFLRSLYKAVGVEPFLE  48753

Query  576    LTKRKKEEKKAKEMSSKQIKANGDKSNGEVVIGLVERELDFHTIVLDCSAIPFIDSTGMA  635
              LTKRKKEEKKAKEMSSKQIKANGDKSNGEVVIGLVERELDFHTIVLDCSAIPFIDSTGMA
Sbjct  48754  LTKRKKEEKKAKEMSSKQIKANGDKSNGEVVIGLVERELDFHTIVLDCSAIPFIDSTGMA  48933

Query  636    TFEGLVKEYGEIRVNVLLANCNTSVIDLLQKGQFFGKNDKDMSSRLFHTVHAAVVHAKDS  695
              TFEGLVKEYGEIRVNVLLANCNTSVIDLLQKGQFFGKNDKDMSSRLFHTVHAAVVHAKDS
Sbjct  48934  TFEGLVKEYGEIRVNVLLANCNTSVIDLLQKGQFFGKNDKDMSSRLFHTVHAAVVHAKDS  49113

Query  696    YAAAESRSEDSGV  708
              YAAAESRSEDSGV
Sbjct  49114  YAAAESRSEDSGV  49152
```

Range 2: 48194 to 48493

Score:367 bits(748), Expect:0.0,
Method:,
Identities:100/100(100%), Positives:100/100(100%), Gaps:0/100(0%)

```
Query  418    TQISSLISALVVLLVLLFFAPYFYALQKCVLACIIIVSLRGALRKFTDVPAKLRASRDDG  477
              TQISSLISALVVLLVLLFFAPYFYALQKCVLACIIIVSLRGALRKFTDVPAKLRASRDDG
Sbjct  48194  TQISSLISALVVLLVLLFFAPYFYALQKCVLACIIIVSLRGALRKFTDVPAKLRASRDDG  48373

Query  478    IVWLVAMFATTLISVEIGLVVGVVFSMICVIYKTQNPKVS  517
              IVWLVAMFATTLISVEIGLVVGVVFSMICVIYKTQNPKVS
Sbjct  48374  IVWLVAMFATTLISVEIGLVVGVVFSMICVIYKTQNPKVS  48493
```

Range 3: 43476 to 43874

Score:488 bits(995), Expect:3e-136,
Method:,
Identities:131/133(98%), Positives:132/133(99%), Gaps:0/133(0%)

```
Query  1      MEEVTKVPPPVLERRVRQRQTTVSVLKSKLKHGVTCSVPKVRSTLTGFFPVVRWLPKYKL  60
              MEEVTKVPPPVLERRVRQRQTTVSVLKSKLKHGVTCSVPKVRSTLTGFFPVVRWLPKYKL
Sbjct  43476  MEEVTKVPPPVLERRVRQRQTTVSVLKSKLKHGVTCSVPKVRSTLTGFFPVVRWLPKYKL  43655

Query  61     KEYVLGDLMSGVIVGIILVPQAIAYCLLAGVPPIYGLYTSFYANIIYFLMGTSRHVSVGI  120
              KEYVLGDLMSGVIVGIILVPQAIAYCLLAGVPPIYGLYTSFYANIIYFLMGTSRHVSVGI
Sbjct  43656  KEYVLGDLMSGVIVGIILVPQAIAYCLLAGVPPIYGLYTSFYANIIYFLMGTSRHVSVGI  43835

Query  121    FSLMSLMVGQVVN  133
              FSLMSLMVGQV +
Sbjct  43836  FSLMSLMVGQVMD  43874
```

Range 4: 44542 to 44790

Score:309 bits(630), Expect:2e-82,

Method:,
Identities:83/83(100%), Positives:83/83(100%), Gaps:0/83(0%)

```
Query  198    QLLMAVFRLGFVSVYLSSPMLDGFATGASFTILTVQAKYLLGIKIPRHQGYGTVVVTWFN  257
              QLLMAVFRLGFVSVYLSSPMLDGFATGASFTILTVQAKYLLGIKIPRHQGYGTVVVTWFN
Sbjct  44542  QLLMAVFRLGFVSVYLSSPMLDGFATGASFTILTVQAKYLLGIKIPRHQGYGTVVVTWFN  44721

Query  258    IFANIHNTNLCDLITSAICISIL  280
              IFANIHNTNLCDLITSAICISIL
Sbjct  44722  IFANIHNTNLCDLITSAICISIL  44790
```

Range 5: 44937 to 45173

Score:287 bits(585), Expect:9e-76,
Method:,
Identities:79/79(100%), Positives:79/79(100%), Gaps:0/79(0%)

```
Query  279    ILVAGKEIQERYKDRLKIPFPTELIIVAGATLASHFGDLNSKYGSSVSGHIPTGFIPPQV  338
              ILVAGKEIQERYKDRLKIPFPTELIIVAGATLASHFGDLNSKYGSSVSGHIPTGFIPPQV
Sbjct  44937  ILVAGKEIQERYKDRLKIPFPTELIIVAGATLASHFGDLNSKYGSSVSGHIPTGFIPPQV  45116

Query  339    PSFTLMPRLALDAIPLAVI  357
              PSFTLMPRLALDAIPLAVI
Sbjct  45117  PSFTLMPRLALDAIPLAVI  45173
```

Range 6: 44181 to 44384

Score:247 bits(503), Expect:1e-63,
Method:,
Identities:68/68(100%), Positives:68/68(100%), Gaps:0/68(0%)

```
Query  131    VVNKEMFLAGFDLNEDSKASGLVLNASLASNLTIGSPHSVELMGVLCGKDCYSISIATAL  190
              VVNKEMFLAGFDLNEDSKASGLVLNASLASNLTIGSPHSVELMGVLCGKDCYSISIATAL
Sbjct  44181  VVNKEMFLAGFDLNEDSKASGLVLNASLASNLTIGSPHSVELMGVLCGKDCYSISIATAL  44360

Query  191    TFMVGVYQ  198
              TFMVGVYQ
Sbjct  44361  TFMVGVYQ  44384
```

Range 7: 47805 to 47993

Score:234 bits(476), Expect:1e-59,
Method:,
Identities:62/63(98%), Positives:63/63(100%), Gaps:0/63(0%)

```
Query  358    SFAFTVSLSEMFAKKNGYTVRPNQEMLAISFCNIIPSFFHCFTTSAALAKTMVKDSTGCQ  417
              SFAFTVSLSEMFAKKNGYTVRPNQEMLAISFCNIIPSFFHCFTTSAALAKTMVKDSTGCQ
Sbjct  47805  SFAFTVSLSEMFAKKNGYTVRPNQEMLAISFCNIIPSFFHCFTTSAALAKTMVKDSTGCQ  47984

Query  418    TQI  420
              TQ+
Sbjct  47985  TQV  47993
```

BY Blackfin icefish *slc26a1*

Query: unnamed protein product Query ID: lcl|Query_8114437 Length: 708

>Chaenocephalus aceratus isolate KU_202001 chromosome 12, whole genome shotgun sequence
Sequence ID: JAMFTG010000012.1 Length: 28277769
Range 1: 19888236 to 19888814

Score:709 bits(1447), Expect:0.0,
Method:,
Identities:193/193(100%), Positives:193/193(100%), Gaps:0/193(0%)

```
Query  516       VSLLGKASNIDLYEDVEEYKNLMPPLRVQIFRFQAPLYYANKNSFLRSLYKAVGVEPFLE  575
                 VSLLGKASNIDLYEDVEEYKNLMPPLRVQIFRFQAPLYYANKNSFLRSLYKAVGVEPFLE
Sbjct  19888236  VSLLGKASNIDLYEDVEEYKNLMPPLRVQIFRFQAPLYYANKNSFLRSLYKAVGVEPFLE  19888415

Query  576       LTKRRKEEKKAKEMSSKQIKANGDKSNGEVVIGLVERELDFHTIVLDCSAIPFIDSTGMA  635
                 LTKRRKEEKKAKEMSSKQIKANGDKSNGEVVIGLVERELDFHTIVLDCSAIPFIDSTGMA
Sbjct  19888416  LTKRRKEEKKAKEMSSKQIKANGDKSNGEVVIGLVERELDFHTIVLDCSAIPFIDSTGMA  19888595

Query  636       TFEGLVKEYGEIRVNVFLANCNTSVIDLLQKGQFFGKNDKDMSSRLFHTVHAAVLHAKDS  695
                 TFEGLVKEYGEIRVNVFLANCNTSVIDLLQKGQFFGKNDKDMSSRLFHTVHAAVLHAKDS
Sbjct  19888596  TFEGLVKEYGEIRVNVFLANCNTSVIDLLQKGQFFGKNDKDMSSRLFHTVHAAVLHAKDS  19888775

Query  696       YAAAESRSENSGV  708
                 YAAAESRSENSGV
Sbjct  19888776  YAAAESRSENSGV  19888814
```

```
Range 2: 19887859 to 19888158

Score:367 bits(747), Expect:0.0,
Method:,
Identities:100/100(100%), Positives:100/100(100%), Gaps:0/100(0%)

Query  418       TQISSLISALVVLLVLLFFAPYFYALQKCVLACIIIVSLRGALRKFKDVPAKLRASRDDG  477
                 TQISSLISALVVLLVLLFFAPYFYALQKCVLACIIIVSLRGALRKFKDVPAKLRASRDDG
Sbjct  19887859  TQISSLISALVVLLVLLFFAPYFYALQKCVLACIIIVSLRGALRKFKDVPAKLRASRDDG  19888038

Query  478       IVWLVAMFATALISVEIGLVVGVVFSMICVIYKTQNPKVS  517
                 IVWLVAMFATALISVEIGLVVGVVFSMICVIYKTQNPKVS
Sbjct  19888039  IVWLVAMFATALISVEIGLVVGVVFSMICVIYKTQNPKVS  19888158



Range 3: 19882345 to 19882743

Score:488 bits(996), Expect:2e-136,
Method:,
Identities:131/133(98%), Positives:132/133(99%), Gaps:0/133(0%)

Query  1         MEEVTQVPPPVLERRVRQRQTTVSVLKSKLKHGVTCSVPKVRSTLTGFFPVVRWLPKYKL  60
                 MEEVTQVPPPVLERRVRQRQTTVSVLKSKLKHGVTCSVPKVRSTLTGFFPVVRWLPKYKL
Sbjct  19882345  MEEVTQVPPPVLERRVRQRQTTVSVLKSKLKHGVTCSVPKVRSTLTGFFPVVRWLPKYKL  19882524

Query  61        KEYVLGDLMSGVIVGIILVPQAIAYCLLAGVPPIYGLYTSFYANIIYFLMGTSRHVSVGI  120
                 KEYVLGDLMSGVIVGIILVPQAIAYCLLAGVPPIYGLYTSFYANIIYFLMGTSRHVSVGI
Sbjct  19882525  KEYVLGDLMSGVIVGIILVPQAIAYCLLAGVPPIYGLYTSFYANIIYFLMGTSRHVSVGI  19882704

Query  121       FSLMSLMVGQVVN  133
                 FSLMSLMVGQV +
Sbjct  19882705  FSLMSLMVGQVMD  19882743



Range 4: 19883408 to 19883656

Score:309 bits(630), Expect:2e-82,
Method:,
Identities:83/83(100%), Positives:83/83(100%), Gaps:0/83(0%)

Query  198       QLLMAVFRLGFVSVYLSSPMLDGFATGASFTILTVQAKYLLGIKIPRHQGYGTVVVTWFN  257
                 QLLMAVFRLGFVSVYLSSPMLDGFATGASFTILTVQAKYLLGIKIPRHQGYGTVVVTWFN
Sbjct  19883408  QLLMAVFRLGFVSVYLSSPMLDGFATGASFTILTVQAKYLLGIKIPRHQGYGTVVVTWFN  19883587

Query  258       IFANIHNTNLCDLITSAICISIL  280
                 IFANIHNTNLCDLITSAICISIL
Sbjct  19883588  IFANIHNTNLCDLITSAICISIL  19883656



Range 5: 19883799 to 19884035

Score:289 bits(588), Expect:3e-76,
Method:,
Identities:79/79(100%), Positives:79/79(100%), Gaps:0/79(0%)

Query  279       ILVAGKEIQERYKDRLKIPFPTELIIVAGATLASHFGDLNSKYDSSVSGHIPTGFIPPQV  338
                 ILVAGKEIQERYKDRLKIPFPTELIIVAGATLASHFGDLNSKYDSSVSGHIPTGFIPPQV
Sbjct  19883799  ILVAGKEIQERYKDRLKIPFPTELIIVAGATLASHFGDLNSKYDSSVSGHIPTGFIPPQV  19883978

Query  339       PSFTLMPRLALDAIPLAII  357
                 PSFTLMPRLALDAIPLAII
Sbjct  19883979  PSFTLMPRLALDAIPLAII  19884035



Range 6: 19883051 to 19883254

Score:247 bits(503), Expect:9e-64,
Method:,
Identities:68/68(100%), Positives:68/68(100%), Gaps:0/68(0%)

Query  131       VVNKEMFLAGFDLNEDSKASGLVLNASLGSNLTIGSPHSVELMGVQCGKECYSISIAAAL  190
                 VVNKEMFLAGFDLNEDSKASGLVLNASLGSNLTIGSPHSVELMGVQCGKECYSISIAAAL
Sbjct  19883051  VVNKEMFLAGFDLNEDSKASGLVLNASLGSNLTIGSPHSVELMGVQCGKECYSISIAAAL  19883230

Query  191       TFMVGVYQ  198
                 TFMVGVYQ
Sbjct  19883231  TFMVGVYQ  19883254



Range 7: 19887467 to 19887655

Score:234 bits(476), Expect:9e-60,
Method:,
Identities:62/63(98%), Positives:63/63(100%), Gaps:0/63(0%)
```

```
Query  358       SFAFTVSLSEMFAKKNGYTVRPNQEMLAISFCNIIPSFFHCFTTSAALAKTMVKDSTGCQ  417
                 SFAFTVSLSEMFAKKNGYTVRPNQEMLAISFCNIIPSFFHCFTTSAALAKTMVKDSTGCQ
Sbjct  19887467  SFAFTVSLSEMFAKKNGYTVRPNQEMLAISFCNIIPSFFHCFTTSAALAKTMVKDSTGCQ  19887646

Query  418       TQI  420
                 TQ+
Sbjct  19887647  TQV  19887655
```

BZ Antarctic spiny plunderfish *slc26a1*

```
Query: unnamed protein product Query ID: lcl|Query_6994901 Length: 709


>Harpagifer antarcticus genome assembly, contig: scaffold_6, whole genome shotgun sequence
Sequence ID: CADEHL010000394.1 Length: 14544978
Range 1: 6894710 to 6895291

Score:710 bits(1449), Expect:0.0,
Method:,
Identities:194/194(100%), Positives:194/194(100%), Gaps:0/194(0%)

Query  516      VSLLGKASDVDLYEDVEEYKNLMPPPRVQIFRFQAPLYYANKDSFLRSLYKAVGVEPFLE  575
                VSLLGKASDVDLYEDVEEYKNLMPPPRVQIFRFQAPLYYANKDSFLRSLYKAVGVEPFLE
Sbjct  6895291  VSLLGKASDVDLYEDVEEYKNLMPPPRVQIFRFQAPLYYANKDSFLRSLYKAVGVEPFLE  6895112

Query  576      LTKRIKEEKKAKEMSSKQIKANGDKSNGEVVIGLVERELDFHTIVLDCSAIPFIDSTGMA  635
                LTKRIKEEKKAKEMSSKQIKANGDKSNGEVVIGLVERELDFHTIVLDCSAIPFIDSTGMA
Sbjct  6895111  LTKRIKEEKKAKEMSSKQIKANGDKSNGEVVIGLVERELDFHTIVLDCSAIPFIDSTGMA  6894932

Query  636      TFEGLVKEYGEIRVNVFLANCNTSVIDLLQKGQFFGKNDKDMSSRLFHTVHAAVLHAKDS  695
                TFEGLVKEYGEIRVNVFLANCNTSVIDLLQKGQFFGKNDKDMSSRLFHTVHAAVLHAKDS
Sbjct  6894931  TFEGLVKEYGEIRVNVFLANCNTSVIDLLQKGQFFGKNDKDMSSRLFHTVHAAVLHAKDS  6894752

Query  696      YAATESRSEDSGV*  709
                YAATESRSEDSGV*
Sbjct  6894751  YAATESRSEDSGV*  6894710



Range 2: 6895366 to 6895665

Score:366 bits(746), Expect:0.0,
Method:,
Identities:100/100(100%), Positives:100/100(100%), Gaps:0/100(0%)

Query  418      TQISSLISALVVLLVLLFFAPYFYALQKCVLACIIIVSLRGALRKFKDVPAKLRASRDDG  477
                TQISSLISALVVLLVLLFFAPYFYALQKCVLACIIIVSLRGALRKFKDVPAKLRASRDDG
Sbjct  6895665  TQISSLISALVVLLVLLFFAPYFYALQKCVLACIIIVSLRGALRKFKDVPAKLRASRDDG  6895486

Query  478      IVWLVAMFATALISVEIGLVVGVVFSMICVVYKTQNPKVS  517
                IVWLVAMFATALISVEIGLVVGVVFSMICVVYKTQNPKVS
Sbjct  6895485  IVWLVAMFATALISVEIGLVVGVVFSMICVVYKTQNPKVS  6895366



Range 3: 6898433 to 6898831

Score:488 bits(996), Expect:2e-136,
Method:,
Identities:131/133(98%), Positives:132/133(99%), Gaps:0/133(0%)

Query  1        MEEVTKVPPPVLERRVRQRQTTVSVLKSKLKHGVTCSVPKVRSTLTGFFPVVRWLPKYKL  60
                MEEVTKVPPPVLERRVRQRQTTVSVLKSKLKHGVTCSVPKVRSTLTGFFPVVRWLPKYKL
Sbjct  6898831  MEEVTKVPPPVLERRVRQRQTTVSVLKSKLKHGVTCSVPKVRSTLTGFFPVVRWLPKYKL  6898652

Query  61       KEYVLGDLMSGVIIGIILVPQAIAYCLLAGVPPIYGLYTSFYANIIYFLMGTSRHVSVGI  120
                KEYVLGDLMSGVIIGIILVPQAIAYCLLAGVPPIYGLYTSFYANIIYFLMGTSRHVSVGI
Sbjct  6898651  KEYVLGDLMSGVIIGIILVPQAIAYCLLAGVPPIYGLYTSFYANIIYFLMGTSRHVSVGI  6898472

Query  121      FSLMSLMVGQVVN  133
                FSLMSLMVGQV +
Sbjct  6898471  FSLMSLMVGQVMD  6898433



Range 4: 6897514 to 6897762

Score:309 bits(630), Expect:2e-82,
Method:,
Identities:83/83(100%), Positives:83/83(100%), Gaps:0/83(0%)

Query  198      QLLMAVFRLGFVSVYLSSPMLDGFATGASFTILTVQAKYLLGIKIPRHQGYGTVVVTWFN  257
                QLLMAVFRLGFVSVYLSSPMLDGFATGASFTILTVQAKYLLGIKIPRHQGYGTVVVTWFN
Sbjct  6897762  QLLMAVFRLGFVSVYLSSPMLDGFATGASFTILTVQAKYLLGIKIPRHQGYGTVVVTWFN  6897583

Query  258      IFANIHNTNLCDLITSAICISIL  280
                IFANIHNTNLCDLITSAICISIL
Sbjct  6897582  IFANIHNTNLCDLITSAICISIL  6897514
```

Range 5: 6897137 to 6897373

Score:287 bits(585), Expect:8e-76,
Method:,
Identities:79/79(100%), Positives:79/79(100%), Gaps:0/79(0%)

```
Query  279      ILVAGKEIQERYKDRLKIPFPTELIIVAGATLASHFGDLNSKYGSSVSGHIPTGFIPPQV  338
                ILVAGKEIQERYKDRLKIPFPTELIIVAGATLASHFGDLNSKYGSSVSGHIPTGFIPPQV
Sbjct  6897373  ILVAGKEIQERYKDRLKIPFPTELIIVAGATLASHFGDLNSKYGSSVSGHIPTGFIPPQV  6897194

Query  339      PSFTLMPRLALDAIPLAVI  357
                PSFTLMPRLALDAIPLAVI
Sbjct  6897193  PSFTLMPRLALDAIPLAVI  6897137
```

Range 6: 6897922 to 6898125

Score:247 bits(503), Expect:1e-63,
Method:,
Identities:68/68(100%), Positives:68/68(100%), Gaps:0/68(0%)

```
Query  131      VVNKEMFLAGFDLNEDSKASGLVLNASLGSNLTIGSPHSVELMGVQCGKECYSISIAAAL  190
                VVNKEMFLAGFDLNEDSKASGLVLNASLGSNLTIGSPHSVELMGVQCGKECYSISIAAAL
Sbjct  6898125  VVNKEMFLAGFDLNEDSKASGLVLNASLGSNLTIGSPHSVELMGVQCGKECYSISIAAAL  6897946

Query  191      TFMVGVYQ  198
                TFMVGVYQ
Sbjct  6897945  TFMVGVYQ  6897922
```

Range 7: 6895869 to 6896057

Score:234 bits(476), Expect:1e-59,
Method:,
Identities:62/63(98%), Positives:63/63(100%), Gaps:0/63(0%)

```
Query  358      SFAFTVSLSEMFAKKNGYTVRPNQEMLAISFCNIIPSFFHCFTTSAALAKTMVKDSTGCQ  417
                SFAFTVSLSEMFAKKNGYTVRPNQEMLAISFCNIIPSFFHCFTTSAALAKTMVKDSTGCQ
Sbjct  6896057  SFAFTVSLSEMFAKKNGYTVRPNQEMLAISFCNIIPSFFHCFTTSAALAKTMVKDSTGCQ  6895878

Query  418      TQI  420
                TQ+
Sbjct  6895877  TQV  6895869
```

CA White-fin plunderfish *slc26a1*

Query: hypothetical protein JOQ06_008693 [Pogonophryne albipinna] Query ID: KAJ4926520.1 Length: 708

>Pogonophryne albipinna isolate SGF0006 chromosome 20, whole genome shotgun sequence
Sequence ID: JAPTMU010000020.1 Length: 27672119
Range 1: 19931028 to 19931606

Score:709 bits(1446), Expect:0.0,
Method:,
Identities:193/193(100%), Positives:193/193(100%), Gaps:0/193(0%)

```
Query  516       VSLLGKASDVDLYEDVEEYKNLMPPPRVQIFRFQAPLYYANKDSFLRSLYKAVGVEPFLE  575
                 VSLLGKASDVDLYEDVEEYKNLMPPPRVQIFRFQAPLYYANKDSFLRSLYKAVGVEPFLE
Sbjct  19931028  VSLLGKASDVDLYEDVEEYKNLMPPPRVQIFRFQAPLYYANKDSFLRSLYKAVGVEPFLE  19931207

Query  576       LTKRRKEEKKAKEMSSKQIKANGDKSNGEVVIGLVERKLDFHTIVLDCSAIPFIDSTGMA  635
                 LTKRRKEEKKAKEMSSKQIKANGDKSNGEVVIGLVERKLDFHTIVLDCSAIPFIDSTGMA
Sbjct  19931208  LTKRRKEEKKAKEMSSKQIKANGDKSNGEVVIGLVERKLDFHTIVLDCSAIPFIDSTGMA  19931387

Query  636       TFEGLVKEYGEIRVNVFLANCNTSVIDLLQKGQFFGKNDKDMSSRLFHTVHAAVLHAKDS  695
                 TFEGLVKEYGEIRVNVFLANCNTSVIDLLQKGQFFGKNDKDMSSRLFHTVHAAVLHAKDS
Sbjct  19931388  TFEGLVKEYGEIRVNVFLANCNTSVIDLLQKGQFFGKNDKDMSSRLFHTVHAAVLHAKDS  19931567

Query  696       YAAAESRSEDSGV  708
                 YAAAESRSEDSGV
Sbjct  19931568  YAAAESRSEDSGV  19931606
```

Range 2: 19930651 to 19930950

Score:367 bits(747), Expect:0.0,
Method:,
Identities:100/100(100%), Positives:100/100(100%), Gaps:0/100(0%)

```
Query  418       TQISSLISALVVLLVLLFFAPYFYALQKCVLACIIIVSLRGALRKFKDVPAKLRASRDDG  477
```

```
                TQISSLISALVVLLVLLFFAPYFYALQKCVLACIIIVSLRGALRKFKDVPAKLRASRDDG
Sbjct  19930651  TQISSLISALVVLLVLLFFAPYFYALQKCVLACIIIVSLRGALRKFKDVPAKLRASRDDG  19930830

Query  478       IVWLVAMFATALISVEIGLVVGVVFSMICVIYKTQNPKVS  517
                IVWLVAMFATALISVEIGLVVGVVFSMICVIYKTQNPKVS
Sbjct  19930831  IVWLVAMFATALISVEIGLVVGVVFSMICVIYKTQNPKVS  19930950
```

Range 3: 19926446 to 19926844

Score:488 bits(996), Expect:4e-142,
Method:,
Identities:131/133(98%), Positives:132/133(99%), Gaps:0/133(0%)

```
Query  1         MEEVTKVPPPVLERRVRQRQTTVSVLKSKLKHGVTCSVPKVRSTLTGFFPVVRWLPKYKL  60
                MEEVTKVPPPVLERRVRQRQTTVSVLKSKLKHGVTCSVPKVRSTLTGFFPVVRWLPKYKL
Sbjct  19926446  MEEVTKVPPPVLERRVRQRQTTVSVLKSKLKHGVTCSVPKVRSTLTGFFPVVRWLPKYKL  19926625

Query  61        KEYVLGDLMSGVIIGIILVPQAIAYCLLAGVPPIYGLYTSFYANIIYFLMGTSRHVSVGI  120
                KEYVLGDLMSGVIIGIILVPQAIAYCLLAGVPPIYGLYTSFYANIIYFLMGTSRHVSVGI
Sbjct  19926626  KEYVLGDLMSGVIIGIILVPQAIAYCLLAGVPPIYGLYTSFYANIIYFLMGTSRHVSVGI  19926805

Query  121       FSLMSLMVGQVVN  133
                FSLMSLMVGQV +
Sbjct  19926806  FSLMSLMVGQVMD  19926844
```

Range 4: 19927491 to 19927739

Score:309 bits(630), Expect:4e-88,
Method:,
Identities:83/83(100%), Positives:83/83(100%), Gaps:0/83(0%)

```
Query  198       QLLMAVFRLGFVSVYLSSPMLDGFATGASFTILTVQAKYLLGIKIPRHQGYGTVVVTWFN  257
                QLLMAVFRLGFVSVYLSSPMLDGFATGASFTILTVQAKYLLGIKIPRHQGYGTVVVTWFN
Sbjct  19927491  QLLMAVFRLGFVSVYLSSPMLDGFATGASFTILTVQAKYLLGIKIPRHQGYGTVVVTWFN  19927670

Query  258       IFANIHNTNLCDLITSAICISIL  280
                IFANIHNTNLCDLITSAICISIL
Sbjct  19927671  IFANIHNTNLCDLITSAICISIL  19927739
```

Range 5: 19927882 to 19928118

Score:287 bits(585), Expect:2e-81,
Method:,
Identities:79/79(100%), Positives:79/79(100%), Gaps:0/79(0%)

```
Query  279       ILVAGKEIQERYKDRLKIPFPTELIIVAGATLASHFGDLNSKYGSSVSGHIPTGFIPPQV  338
                ILVAGKEIQERYKDRLKIPFPTELIIVAGATLASHFGDLNSKYGSSVSGHIPTGFIPPQV
Sbjct  19927882  ILVAGKEIQERYKDRLKIPFPTELIIVAGATLASHFGDLNSKYGSSVSGHIPTGFIPPQV  19928061

Query  339       PSFTLMPRLALDAIPLAVI  357
                PSFTLMPRLALDAIPLAVI
Sbjct  19928062  PSFTLMPRLALDAIPLAVI  19928118
```

Range 6: 19927152 to 19927355

Score:247 bits(503), Expect:2e-69,
Method:,
Identities:68/68(100%), Positives:68/68(100%), Gaps:0/68(0%)

```
Query  131       VVNKEMFLAGFDLNEDSTASGLVLNASLGSNLTIGSPHSVELMGVQCGKECYSISIAAAL  190
                VVNKEMFLAGFDLNEDSTASGLVLNASLGSNLTIGSPHSVELMGVQCGKECYSISIAAAL
Sbjct  19927152  VVNKEMFLAGFDLNEDSTASGLVLNASLGSNLTIGSPHSVELMGVQCGKECYSISIAAAL  19927331

Query  191       TFMVGVYQ  198
                TFMVGVYQ
Sbjct  19927332  TFMVGVYQ  19927355
```

Range 7: 19930259 to 19930447

Score:234 bits(476), Expect:2e-65,
Method:,
Identities:62/63(98%), Positives:63/63(100%), Gaps:0/63(0%)

```
Query  358       SFAFTVSLSEMFAKKNGYTVRPNQEMLAISFCNIIPSFFHCFTTSAALAKTMVKDSTGCQ  417
                SFAFTVSLSEMFAKKNGYTVRPNQEMLAISFCNIIPSFFHCFTTSAALAKTMVKDSTGCQ
Sbjct  19930259  SFAFTVSLSEMFAKKNGYTVRPNQEMLAISFCNIIPSFFHCFTTSAALAKTMVKDSTGCQ  19930438

Query  418       TQI  420
                TQ+
Sbjct  19930439  TQV  19930447
```

CB Elephant shark *slc26a2*

```
Query: sulfate transporter [Callorhinchus milii] Query ID: XP_007910486.2 Length: 690


>Callorhinchus milii isolate IMCB2004 unplaced genomic scaffold, IMCB_Cmil_1.0 Scaffold13, whole genome shotgun sequence
Sequence ID: NW_024704754.1 Length: 19629525
Range 1: 4172531 to 4174033

Score:936 bits(2419), Expect:0.0,
Method:Compositional matrix adjust.,
Identities:500/501(99%), Positives:501/501(100%), Gaps:0/501(0%)

Query  190      YQVAFGLFQIGFVSVYLSDSLLSGFVTGASFTILTSQVKYLLGIKINQPRGPGSLILTWV  249
                +QVAFGLFQIGFVSVYLSDSLLSGFVTGASFTILTSQVKYLLGIKINQPRGPGSLILTWV
Sbjct  4174033  FQVAFGLFQIGFVSVYLSDSLLSGFVTGASFTILTSQVKYLLGIKINQPRGPGSLILTWV  4173854

Query  250      EIFRNIHKTNLCDLITSILCLLVLVPIKEVNARCKPKLKGPIPGELLVVIVATLISHYGN  309
                EIFRNIHKTNLCDLITSILCLLVLVPIKEVNARCKPKLKGPIPGELLVVIVATLISHYGN
Sbjct  4173853  EIFRNIHKTNLCDLITSILCLLVLVPIKEVNARCKPKLKGPIPGELLVVIVATLISHYGN  4173674

Query  310      LNVKYDSSVSGDIPTGFQPPSFPDWSILGRVapgafpiaiigfaiTVSLSEMFAKKHSYN  369
                LNVKYDSSVSGDIPTGFQPPSFPDWSILGRVAPGAFPIAIIGFAITVSLSEMFAKKHSYN
Sbjct  4173673  LNVKYDSSVSGDIPTGFQPPSFPDWSILGRVAPGAFPIAIIGFAITVSLSEMFAKKHSYN  4173494

Query  370      VRANQEMFAIGFCNIIPSFFHCFTTSAALAKTLVKESTGCKTQlssivtslvlllvllvl  429
                VRANQEMFAIGFCNIIPSFFHCFTTSAALAKTLVKESTGCKTQLSSIVTSLVLLLVLLVL
Sbjct  4173493  VRANQEMFAIGFCNIIPSFFHCFTTSAALAKTLVKESTGCKTQLSSIVTSLVLLLVLLVL  4173314

Query  430      aplfyslQKCVLGVVTIVNLRGALRKFIDLPKMWHLSKVDTCIWFITMLCSALISTELGL  489
                APLFYSLQKCVLGVVTIVNLRGALRKFIDLPKMWHLSKVDTCIWFITMLCSALISTELGL
Sbjct  4173313  APLFYSLQKCVLGVVTIVNLRGALRKFIDLPKMWHLSKVDTCIWFITMLCSALISTELGL  4173134

Query  490      LIGVVLSVFCVIIRTQLPTASLLGRLDGTEIYQDLGVYKHLKMVPGIKIFRFEAALYYAN  549
                LIGVVLSVFCVIIRTQLPTASLLGRLDGTEIYQDLGVYKHLKMVPGIKIFRFEAALYYAN
Sbjct  4173133  LIGVVLSVFCVIIRTQLPTASLLGRLDGTEIYQDLGVYKHLKMVPGIKIFRFEAALYYAN  4172954

Query  550      KEGFKSALYKQTGVNPPQVTMAQKKAERKIHLREKKQKDAETCKSYEVTMQLFKPEFDFQ  609
                KEGFKSALYKQTGVNPPQVTMAQKKAERKIHLREKKQKDAETCKSYEVTMQLFKPEFDFQ
Sbjct  4172953  KEGFKSALYKQTGVNPPQVTMAQKKAERKIHLREKKQKDAETCKSYEVTMQLFKPEFDFQ  4172774

Query  610      TLIIDCSAIQFLDSAGIATVKETFRDYKLVGIQILLANCNASVINSLQRGNYFEGSDSNT  669
                TLIIDCSAIQFLDSAGIATVKETFRDYKLVGIQILLANCNASVINSLQRGNYFEGSDSNT
Sbjct  4172773  TLIIDCSAIQFLDSAGIATVKETFRDYKLVGIQILLANCNASVINSLQRGNYFEGSDSNT  4172594

Query  670      LAFCSVHDAVTYVLNKGQSNG  690
                LAFCSVHDAVTYVLNKGQSNG
Sbjct  4172593  LAFCSVHDAVTYVLNKGQSNG  4172531



Range 2: 4174723 to 4175298

Score:370 bits(950), Expect:5e-117,
Method:Compositional matrix adjust.,
Identities:192/192(100%), Positives:192/192(100%), Gaps:0/192(0%)

Query  1        MTSTEANNGEITSEVETQVSPGPRHQQYSPIVLEKYEKKPidikkiavkklkkYCACTPQ  60
                MTSTEANNGEITSEVETQVSPGPRHQQYSPIVLEKYEKKPIDIKKIAVKKLKKYCACTPQ
Sbjct  4175298  MTSTEANNGEITSEVETQVSPGPRHQQYSPIVLEKYEKKPIDIKKIAVKKLKKYCACTPQ  4175119

Query  61       NAKQYVTGFFPVLQWFPKYRWREWIFGDIMSGLIVGILLVPQSIAYSLLAGQEPKYGLYT  120
                NAKQYVTGFFPVLQWFPKYRWREWIFGDIMSGLIVGILLVPQSIAYSLLAGQEPKYGLYT
Sbjct  4175118  NAKQYVTGFFPVLQWFPKYRWREWIFGDIMSGLIVGILLVPQSIAYSLLAGQEPKYGLYT  4174939

Query  121      SFFACIIYFLMGTSKHISVGIFGVLCLMIGEVVDAELQFEAVQNSTASDCDVSCRSIAIA  180
                SFFACIIYFLMGTSKHISVGIFGVLCLMIGEVVDAELQFEAVQNSTASDCDVSCRSIAIA
Sbjct  4174938  SFFACIIYFLMGTSKHISVGIFGVLCLMIGEVVDAELQFEAVQNSTASDCDVSCRSIAIA  4174759

Query  181      STVTFMAGIYQV  192
                STVTFMAGIYQV
Sbjct  4174758  STVTFMAGIYQV  4174723
```

CC Little skate *slc26a2*

```
Query: sulfate transporter [Leucoraja erinacea] Query ID: XP_055498994.1 Length: 713


>Leucoraja erinacea ecotype New England chromosome 11, Leri_hhj_1, whole genome shotgun sequence
Sequence ID: NC_073387.1 Length: 60429722
Range 1: 35694396 to 35695934

Score:1888 bits(3858), Expect:0.0,
Method:,
Identities:512/513(99%), Positives:513/513(100%), Gaps:0/513(0%)
```

```
Query  201       YQVAMGIFQVGFISVYLSDSLLSGFATGASFTIITSQIKYILGIRIPRVNTPGSLVKTWV  260
                 +QVAMGIFQVGFISVYLSDSLLSGFATGASFTIITSQIKYILGIRIPRVNTPGSLVKTWV
Sbjct  35695934  FQVAMGIFQVGFISVYLSDSLLSGFATGASFTIITSQIKYILGIRIPRVNTPGSLVKTWV  35695755

Query  261       HIFKNIHETNICDLVTSILSLLFLVPIKEINECYKQKLKMPIPGELLVVIIATLISHFGQ  320
                 HIFKNIHETNICDLVTSILSLLFLVPIKEINECYKQKLKMPIPGELLVVIIATLISHFGQ
Sbjct  35695754  HIFKNIHETNICDLVTSILSLLFLVPIKEINECYKQKLKMPIPGELLVVIIATLISHFGQ  35695575

Query  321       LNAKFNSTIAGDIPTGFIAPTSPDWSLIPRIAIDAIPIAIIGFAITVTLSEMFAKKHGYT  380
                 LNAKFNSTIAGDIPTGFIAPTSPDWSLIPRIAIDAIPIAIIGFAITVTLSEMFAKKHGYT
Sbjct  35695574  LNAKFNSTIAGDIPTGFIAPTSPDWSLIPRIAIDAIPIAIIGFAITVTLSEMFAKKHGYT  35695395

Query  381       VKANQEMFAIGACNVIPAFFHCFTSSAALAKSLVKESTSCKTQLSSLVTALVLLLVLLVI  440
                 VKANQEMFAIGACNVIPAFFHCFTSSAALAKSLVKESTSCKTQLSSLVTALVLLLVLLVI
Sbjct  35695394  VKANQEMFAIGACNVIPAFFHCFTSSAALAKSLVKESTSCKTQLSSLVTALVLLLVLLVI  35695215

Query  441       APLFYSLQKCVLAVIIIVNLRGALRKFLELPKMWNTSKVDTVVWFITMLATALVSTELGL  500
                 APLFYSLQKCVLAVIIIVNLRGALRKFLELPKMWNTSKVDTVVWFITMLATALVSTELGL
Sbjct  35695214  APLFYSLQKCVLAVIIIVNLRGALRKFLELPKMWNTSKVDTVVWFITMLATALVSTELGL  35695035

Query  501       LIGVCFSVICVIVRTQVPRTTVLGHLKGTEIYEDLRKYRNLQSLPGIKIFRFEAPLYYAN  560
                 LIGVCFSVICVIVRTQVPRTTVLGHLKGTEIYEDLRKYRNLQSLPGIKIFRFEAPLYYAN
Sbjct  35695034  LIGVCFSVICVIVRTQVPRTTVLGHLKGTEIYEDLRKYRNLQSLPGIKIFRFEAPLYYAN  35694855

Query  561       KELFKEALYKQTGINPTLVVLARKKAEKKNIRELKQKQTAHSQAGVKPEVTLQLSYGYQE  620
                 KELFKEALYKQTGINPTLVVLARKKAEKKNIRELKQKQTAHSQAGVKPEVTLQLSYGYQE
Sbjct  35694854  KELFKEALYKQTGINPTLVVLARKKAEKKNIRELKQKQTAHSQAGVKPEVTLQLSYGYQE  35694675

Query  621       FDIHTIIIDCSVIQFLDTAGIATMKEAFKDYKEIGILVLLSNCNASVIDSLRNGNYFDNG  680
                 FDIHTIIIDCSVIQFLDTAGIATMKEAFKDYKEIGILVLLSNCNASVIDSLRNGNYFDNG
Sbjct  35694674  FDIHTIIIDCSVIQFLDTAGIATMKEAFKDYKEIGILVLLSNCNASVIDSLRNGNYFDNG  35694495

Query  681       YISGLLFYSVHDAVKFASNVHQKNGDCEANTPC  713
                 YISGLLFYSVHDAVKFASNVHQKNGDCEANTPC
Sbjct  35694494  YISGLLFYSVHDAVKFASNVHQKNGDCEANTPC  35694396
```

Range 2: 35700811 to 35701419

Score:760 bits(1551), Expect:0.0,
Method:,
Identities:203/203(100%), Positives:203/203(100%), Gaps:0/203(0%)

```
Query  1         MTSADVNNAESMLVLNNVVLCDMKSDKYSPIILERQEKEPLDVKACAVKKLKKHLSCTPA  60
                 MTSADVNNAESMLVLNNVVLCDMKSDKYSPIILERQEKEPLDVKACAVKKLKKHLSCTPA
Sbjct  35701419  MTSADVNNAESMLVLNNVVLCDMKSDKYSPIILERQEKEPLDVKACAVKKLKKHLSCTPA  35701240

Query  61        KAKDLLVDLFPIIRWFPKYKWKKWILGDIMSGLVVGILIVPQSIAYSLLAGQDPVYGLYT  120
                 KAKDLLVDLFPIIRWFPKYKWKKWILGDIMSGLVVGILIVPQSIAYSLLAGQDPVYGLYT
Sbjct  35701239  KAKDLLVDLFPIIRWFPKYKWKKWILGDIMSGLVVGILIVPQSIAYSLLAGQDPVYGLYT  35701060

Query  121       SFFACIIYSLMGTSKHISVGIFGVLCLMIGQVVERELQFAGFDISDDINTTALPNQAANV  180
                 SFFACIIYSLMGTSKHISVGIFGVLCLMIGQVVERELQFAGFDISDDINTTALPNQAANV
Sbjct  35701059  SFFACIIYSLMGTSKHISVGIFGVLCLMIGQVVERELQFAGFDISDDINTTALPNQAANV  35700880

Query  181       CGRSCFAIRIGSTLTFMAGIYQV  203
                 CGRSCFAIRIGSTLTFMAGIYQV
Sbjct  35700879  CGRSCFAIRIGSTLTFMAGIYQV  35700811
```

CD Smaller spotted catshark *slc26a2*

Query: sulfate transporter [Scyliorhinus canicula] Query ID: XP_038651483.1 Length: 713

>Scyliorhinus canicula chromosome 4, sScyCan1.1, whole genome shotgun sequence
Sequence ID: NC_052149.1 Length: 244324502
Range 1: 173758949 to 173760481

Score:1882 bits(3845), Expect:0.0,
Method:,
Identities:510/511(99%), Positives:511/511(100%), Gaps:0/511(0%)

```
Query  203        YQVAMGIFQVGFISVYLSDSLLSGFATGASFTILTSQVKYLLGISIPRAHGPGSLVQTWV  262
                  +QVAMGIFQVGFISVYLSDSLLSGFATGASFTILTSQVKYLLGISIPRAHGPGSLVQTWV
Sbjct  173760481  FQVAMGIFQVGFISVYLSDSLLSGFATGASFTILTSQVKYLLGISIPRAHGPGSLVQTWV  173760302

Query  263        HIFKNIHTTNICDLVTSLVCLLILVPLKEINECYKSKLKAPIPGELLVVVFATLVSHYGH  322
                  HIFKNIHTTNICDLVTSLVCLLILVPLKEINECYKSKLKAPIPGELLVVVFATLVSHYGH
Sbjct  173760301  HIFKNIHTTNICDLVTSLVCLLILVPLKEINECYKSKLKAPIPGELLVVVFATLVSHYGH  173760122

Query  323        LNAKFNSTIAGNIPTGFMAPTSPDWSLIPRVAADALPIAIIGFAITVSLSEMFAKKHGYT  382
                  LNAKFNSTIAGNIPTGFMAPTSPDWSLIPRVAADALPIAIIGFAITVSLSEMFAKKHGYT
Sbjct  173760121  LNAKFNSTIAGNIPTGFMAPTSPDWSLIPRVAADALPIAIIGFAITVSLSEMFAKKHGYT  173759942

Query  383        VRANQEMFAIGTCNIIPAFFHCFTTSAALAKTLVKESTGCRTQFSSIVTALVLLLVLLVI  442
                  VRANQEMFAIGTCNIIPAFFHCFTTSAALAKTLVKESTGCRTQFSSIVTALVLLLVLLVI
Sbjct  173759941  VRANQEMFAIGTCNIIPAFFHCFTTSAALAKTLVKESTGCRTQFSSIVTALVLLLVLLVI  173759762
```

```
Query  443        APLFYSLQKCVLGVITIVNLRGALRKFLELPKMWKVSKVDTVIWFVTMLSSALISTELGL  502
                  APLFYSLQKCVLGVITIVNLRGALRKFLELPKMWKVSKVDTVIWFVTMLSSALISTELGL
Sbjct  173759761  APLFYSLQKCVLGVITIVNLRGALRKFLELPKMWKVSKVDTVIWFVTMLSSALISTELGL  173759582

Query  503        LIGVSFSVICVIARTQMPRATMLGHLEGTEIYEDLGRYKNLQNLSGVKIFRFEAPLYYAN  562
                  LIGVSFSVICVIARTQMPRATMLGHLEGTEIYEDLGRYKNLQNLSGVKIFRFEAPLYYAN
Sbjct  173759581  LIGVSFSVICVIARTQMPRATMLGHLEGTEIYEDLGRYKNLQNLSGVKIFRFEAPLYYAN  173759402

Query  563        KESFKASLYKQVGVNPTLVIMAKKKAERKLIREMKQKVTVNACASMKIEATVQLSKQEFE  622
                  KESFKASLYKQVGVNPTLVIMAKKKAERKLIREMKQKVTVNACASMKIEATVQLSKQEFE
Sbjct  173759401  KESFKASLYKQVGVNPTLVIMAKKKAERKLIREMKQKVTVNACASMKIEATVQLSKQEFE  173759222

Query  623        FHTVIIDFSVIQFLDTAGIATVKEVYKDYKQIGIQILLSNCNALVIDSLRNGGYFENDSI  682
                  FHTVIIDFSVIQFLDTAGIATVKEVYKDYKQIGIQILLSNCNALVIDSLRNGGYFENDSI
Sbjct  173759221  FHTVIIDFSVIQFLDTAGIATVKEVYKDYKQIGIQILLSNCNALVIDSLRNGGYFENDSI  173759042

Query  683        HTLVFYSVHDAVMFASNVQQKNGDWEGSNLC  713
                  HTLVFYSVHDAVMFASNVQQKNGDWEGSNLC
Sbjct  173759041  HTLVFYSVHDAVMFASNVQQKNGDWEGSNLC  173758949
```

Range 2: 173766330 to 173766944

Score:768 bits(1568), Expect:0.0,
Method:,
Identities:205/205(100%), Positives:205/205(100%), Gaps:0/205(0%)

```
Query  1          MTSTEANTADLLALSTPASPAMKNRKYSPIILEEQERNTFDVKKVAAKRLKKCCSCTPTK  60
                  MTSTEANTADLLALSTPASPAMKNRKYSPIILEEQERNTFDVKKVAAKRLKKCCSCTPTK
Sbjct  173766944  MTSTEANTADLLALSTPASPAMKNRKYSPIILEEQERNTFDVKKVAAKRLKKCCSCTPTK  173766765

Query  61         AKDFVVDFFPAIRWFPKYKWKEWILGDIMSGLIVGILLVPQSIAYSLLAGQEPIYGLYTS  120
                  AKDFVVDFFPAIRWFPKYKWKEWILGDIMSGLIVGILLVPQSIAYSLLAGQEPIYGLYTS
Sbjct  173766764  AKDFVVDFFPAIRWFPKYKWKEWILGDIMSGLIVGILLVPQSIAYSLLAGQEPIYGLYTS  173766585

Query  121        FFACIIYFLMGTSKHISVGIFGVLCLMIGQVVDRELQFAGFDIYDDSNSTGQAEMFNQSA  180
                  FFACIIYFLMGTSKHISVGIFGVLCLMIGQVVDRELQFAGFDIYDDSNSTGQAEMFNQSA
Sbjct  173766584  FFACIIYFLMGTSKHISVGIFGVLCLMIGQVVDRELQFAGFDIYDDSNSTGQAEMFNQSA  173766405

Query  181        GACGRSCFAIRVGSTLTFMAGVYQV  205
                  GACGRSCFAIRVGSTLTFMAGVYQV
Sbjct  173766404  GACGRSCFAIRVGSTLTFMAGVYQV  173766330
```

CE Human *slc26a2*

Query: sulfate transporter [Homo sapiens] Query ID: NP_000103.2 Length: 739

>Homo sapiens chromosome 5, GRCh38.p14 Primary Assembly
Sequence ID: NC_000005.10 Length: 181538259
Range 1: 149980287 to 149981810

Score:1864 bits(3809), Expect:0.0,
Method:,
Identities:507/508(99%), Positives:508/508(100%), Gaps:0/508(0%)

```
Query  232        YQVAMGFFQVGFVSVYLSDALLSGFVTGASFTILTSQAKYLLGLNLPRTNGVGSLITTWI  291
                  +QVAMGFFQVGFVSVYLSDALLSGFVTGASFTILTSQAKYLLGLNLPRTNGVGSLITTWI
Sbjct  149980287  FQVAMGFFQVGFVSVYLSDALLSGFVTGASFTILTSQAKYLLGLNLPRTNGVGSLITTWI  149980466

Query  292        HVFRNIHKTNLCDLITSLLCLLVLLPTKELNEHFKSKLKAPIPIELVVVVAATLASHFGK  351
                  HVFRNIHKTNLCDLITSLLCLLVLLPTKELNEHFKSKLKAPIPIELVVVVAATLASHFGK
Sbjct  149980467  HVFRNIHKTNLCDLITSLLCLLVLLPTKELNEHFKSKLKAPIPIELVVVVAATLASHFGK  149980646

Query  352        LHENYNSSIAGHIPTGFMPPKVPEWNLIPSVAVDAIAISIIGFAITVSLSEMFAKKHGYT  411
                  LHENYNSSIAGHIPTGFMPPKVPEWNLIPSVAVDAIAISIIGFAITVSLSEMFAKKHGYT
Sbjct  149980647  LHENYNSSIAGHIPTGFMPPKVPEWNLIPSVAVDAIAISIIGFAITVSLSEMFAKKHGYT  149980826

Query  412        VKANQEMYAIGFCNIIPSFFHCFTTSAALAKTLVKESTGCHTQLSGVVTALVLLLVLLVI  471
                  VKANQEMYAIGFCNIIPSFFHCFTTSAALAKTLVKESTGCHTQLSGVVTALVLLLVLLVI
Sbjct  149980827  VKANQEMYAIGFCNIIPSFFHCFTTSAALAKTLVKESTGCHTQLSGVVTALVLLLVLLVI  149981006

Query  472        APLFYSLQKSVLGVITIVNLRGALRKFRDLPKMWSISRMDTVIWFVTMLSSALLSTEIGL  531
                  APLFYSLQKSVLGVITIVNLRGALRKFRDLPKMWSISRMDTVIWFVTMLSSALLSTEIGL
Sbjct  149981007  APLFYSLQKSVLGVITIVNLRGALRKFRDLPKMWSISRMDTVIWFVTMLSSALLSTEIGL  149981186

Query  532        LVGVCFSIFCVILRTQKPKSSLLGLVEESEVFESVSAYKNLQIKPGIKIFRFVAPLYYIN  591
                  LVGVCFSIFCVILRTQKPKSSLLGLVEESEVFESVSAYKNLQIKPGIKIFRFVAPLYYIN
Sbjct  149981187  LVGVCFSIFCVILRTQKPKSSLLGLVEESEVFESVSAYKNLQIKPGIKIFRFVAPLYYIN  149981366

Query  592        KECFKSALYKQTVNPILIKVAWKKAAKRKIKEKVVTLGGIQDEMSVQLSHDPLELHTIVI  651
                  KECFKSALYKQTVNPILIKVAWKKAAKRKIKEKVVTLGGIQDEMSVQLSHDPLELHTIVI
Sbjct  149981367  KECFKSALYKQTVNPILIKVAWKKAAKRKIKEKVVTLGGIQDEMSVQLSHDPLELHTIVI  149981546

Query  652        DCSAIQFLDTAGIHTLKEVRRDYEAIGIQVLLAQCNPTVRDSLTNGEYCKKEEENLLFYS  711
                  DCSAIQFLDTAGIHTLKEVRRDYEAIGIQVLLAQCNPTVRDSLTNGEYCKKEEENLLFYS
Sbjct  149981547  DCSAIQFLDTAGIHTLKEVRRDYEAIGIQVLLAQCNPTVRDSLTNGEYCKKEEENLLFYS  149981726

Query  712        VYEAMAFAEVSKNQKGVCVPNGLSLSSD  739
```

VYEAMAFAEVSKNQKGVCVPNGLSLSSD
Sbjct 149981727 VYEAMAFAEVSKNQKGVCVPNGLSLSSD 149981810

Range 2: 149977653 to 149978354

Score:869 bits(1774), Expect:0.0,
Method:,
Identities:234/234(100%), Positives:234/234(100%), Gaps:0/234(0%)

Query 1 MSSESKEQHNVSPRDSAEGNDSYPSGIHLELQRESSTDFKQFETNDQCRPYHRILIERQE 60
MSSESKEQHNVSPRDSAEGNDSYPSGIHLELQRESSTDFKQFETNDQCRPYHRILIERQE
Sbjct 149977653 MSSESKEQHNVSPRDSAEGNDSYPSGIHLELQRESSTDFKQFETNDQCRPYHRILIERQE 149977832

Query 61 KSDTNFKEFVIKKLQKNCQCSPAKAKNMILGFLPVLQWLPKYDLKKNILGDVMSGLIVGI 120
KSDTNFKEFVIKKLQKNCQCSPAKAKNMILGFLPVLQWLPKYDLKKNILGDVMSGLIVGI
Sbjct 149977833 KSDTNFKEFVIKKLQKNCQCSPAKAKNMILGFLPVLQWLPKYDLKKNILGDVMSGLIVGI 149978012

Query 121 LLVPQSIAYSLLAGQEPVYGLYTSFFASIIYFLLGTSRHISVGIFGVLCLMIGETVDREL 180
LLVPQSIAYSLLAGQEPVYGLYTSFFASIIYFLLGTSRHISVGIFGVLCLMIGETVDREL
Sbjct 149978013 LLVPQSIAYSLLAGQEPVYGLYTSFFASIIYFLLGTSRHISVGIFGVLCLMIGETVDREL 149978192

Query 181 QKAGYDNAHSAPSLGMVSNGSTLLNHTSDRICDKSCYAIMVGSTVTFIAGVYQV 234
QKAGYDNAHSAPSLGMVSNGSTLLNHTSDRICDKSCYAIMVGSTVTFIAGVYQV
Sbjct 149978193 QKAGYDNAHSAPSLGMVSNGSTLLNHTSDRICDKSCYAIMVGSTVTFIAGVYQV 149978354

CF Dog *slc26a2*

Query: sulfate transporter [Canis lupus familiaris] Query ID: XP_038519588.1 Length: 742

>Canis lupus familiaris isolate SID07034 breed Labrador retriever chromosome 4, ROS_Cfam_1.0, whole genome shotgun sequence
Sequence ID: NC_051808.1 Length: 89007665
Range 1: 59554584 to 59556107

Score:1858 bits(3797), Expect:0.0,
Method:,
Identities:507/508(99%), Positives:508/508(100%), Gaps:0/508(0%)

Query 235 YQVAMGFFQVGFVSVYLSDALLSGFVTGASFTILTSQAKYLLGLSLPRSNGVGSLITTWI 294
+QVAMGFFQVGFVSVYLSDALLSGFVTGASFTILTSQAKYLLGLSLPRSNGVGSLITTWI
Sbjct 59556107 FQVAMGFFQVGFVSVYLSDALLSGFVTGASFTILTSQAKYLLGLSLPRSNGVGSLITTWI 59555928

Query 295 HIFKNIHKTNICDLITSLLCLLVLLPTKELNEHFKSKLKAPIPTELIVVVAATLASHFGK 354
HIFKNIHKTNICDLITSLLCLLVLLPTKELNEHFKSKLKAPIPTELIVVVAATLASHFGK
Sbjct 59555927 HIFKNIHKTNICDLITSLLCLLVLLPTKELNEHFKSKLKAPIPTELIVVVAATLASHFGK 59555748

Query 355 LNEKYNTSIAGSIPTGFMPPTAPDWNLIPSLAVDAIAISIIGFAITVSLSEMFAKKHGYS 414
LNEKYNTSIAGSIPTGFMPPTAPDWNLIPSLAVDAIAISIIGFAITVSLSEMFAKKHGYS
Sbjct 59555747 LNEKYNTSIAGSIPTGFMPPTAPDWNLIPSLAVDAIAISIIGFAITVSLSEMFAKKHGYS 59555568

Query 415 VKANQEMYAIGFCNIIPSFFHCFTTSAALAKTLVKESTGCQTQLSGVITALVLLLVLLVI 474
VKANQEMYAIGFCNIIPSFFHCFTTSAALAKTLVKESTGCQTQLSGVITALVLLLVLLVI
Sbjct 59555567 VKANQEMYAIGFCNIIPSFFHCFTTSAALAKTLVKESTGCQTQLSGVITALVLLLVLLVI 59555388

Query 475 APLFYSLQKSVLGVITIVNLRGALRKFKDLPKMWKVSRMDTVIWFVTMLSSALISTEIGL 534
APLFYSLQKSVLGVITIVNLRGALRKFKDLPKMWKVSRMDTVIWFVTMLSSALISTEIGL
Sbjct 59555387 APLFYSLQKSVLGVITIVNLRGALRKFKDLPKMWKVSRMDTVIWFVTMLSSALISTEIGL 59555208

Query 535 LIGVCFSMFCVILRTQKPKTSLLGLVEESEIFESMSAYKNLQTKPGIKIFRFVAPLYYIN 594
LIGVCFSMFCVILRTQKPKTSLLGLVEESEIFESMSAYKNLQTKPGIKIFRFVAPLYYIN
Sbjct 59555207 LIGVCFSMFCVILRTQKPKTSLLGLVEESEIFESMSAYKNLQTKPGIKIFRFVAPLYYIN 59555028

Query 595 KECFKSALYKKTLNPVLVKAAQKKAAKRKITKETVILSGVQDEVSVQLSHDPLELQTIVI 654
KECFKSALYKKTLNPVLVKAAQKKAAKRKITKETVILSGVQDEVSVQLSHDPLELQTIVI
Sbjct 59555027 KECFKSALYKKTLNPVLVKAAQKKAAKRKITKETVILSGVQDEVSVQLSHDPLELQTIVI 59554848

Query 655 DCSAIQFLDTAGIHTLKEVRRDYEAIGIQVLLAQCNPSVRDSLARGEYCKKEEENLLFYS 714
DCSAIQFLDTAGIHTLKEVRRDYEAIGIQVLLAQCNPSVRDSLARGEYCKKEEENLLFYS
Sbjct 59554847 DCSAIQFLDTAGIHTLKEVRRDYEAIGIQVLLAQCNPSVRDSLARGEYCKKEEENLLFYS 59554668

Query 715 VYEAMAFAEESQNQKGVCIPNGLSLSSD 742
VYEAMAFAEESQNQKGVCIPNGLSLSSD
Sbjct 59554667 VYEAMAFAEESQNQKGVCIPNGLSLSSD 59554584

Range 2: 59557644 to 59558354

Score:881 bits(1799), Expect:0.0,
Method:,
Identities:237/237(100%), Positives:237/237(100%), Gaps:0/237(0%)

Query 1 MSLESKQQHDLSLKNSVEGNDQHSPLSNMHPELGKQSSTDFKQFEANDQCTLYRRIHMEP 60
MSLESKQQHDLSLKNSVEGNDQHSPLSNMHPELGKQSSTDFKQFEANDQCTLYRRIHMEP
Sbjct 59558354 MSLESKQQHDLSLKNSVEGNDQHSPLSNMHPELGKQSSTDFKQFEANDQCTLYRRIHMEP 59558175

Query 61 REKSSTNFKQFVIRKLQKSCQCSPAKAKNMIFDFLPVLRWLPKYDLKKNILGDVMSGLIV 120

```
                    REKSSTNFKQFVIRKLQKSCQCSPAKAKNMIFDFLPVLRWLPKYDLKKNILGDVMSGLIV
Sbjct  59558174  REKSSTNFKQFVIRKLQKSCQCSPAKAKNMIFDFLPVLRWLPKYDLKKNILGDVMSGLIV  59557995

Query  121       GILLVPQSIAYSLLAGQEPIYGLYTSFFASIIYFLLGTSRHISVGIFGILCLMIGEVVDR  180
                    GILLVPQSIAYSLLAGQEPIYGLYTSFFASIIYFLLGTSRHISVGIFGILCLMIGEVVDR
Sbjct  59557994  GILLVPQSIAYSLLAGQEPIYGLYTSFFASIIYFLLGTSRHISVGIFGILCLMIGEVVDR  59557815

Query  181       ELHKAGYDTADNAPSDLGLVLNGSTLLNQTSDRICDRSCYAIAVGSTVTFMAGVYQV  237
                    ELHKAGYDTADNAPSDLGLVLNGSTLLNQTSDRICDRSCYAIAVGSTVTFMAGVYQV
Sbjct  59557814  ELHKAGYDTADNAPSDLGLVLNGSTLLNQTSDRICDRSCYAIAVGSTVTFMAGVYQV  59557644
```

CG Nine-banded armadillo *slc26a2*

```
Query: unnamed protein product Query ID: lcl|Query_3064223 Length: 741

>Dasypus novemcinctus isolate mDasNov1 chromosome 2, whole genome shotgun sequence
Sequence ID: JAUJUB010000002.1 Length: 206848192
Range 1: 160604502 to 160606025

Score:926 bits(2392), Expect:0.0,
Method:Compositional matrix adjust.,
Identities:507/508(99%), Positives:508/508(100%), Gaps:0/508(0%)

Query  234        YQVAMGFFQVGFVSVYLSDALLSGFVTGASFTILTSQAKYLLGLSIPRSNGVGSLITTWI  293
                  +QVAMGFFQVGFVSVYLSDALLSGFVTGASFTILTSQAKYLLGLSIPRSNGVGSLITTWI
Sbjct  160604502  FQVAMGFFQVGFVSVYLSDALLSGFVTGASFTILTSQAKYLLGLSIPRSNGVGSLITTWI  160604681

Query  294        HIFRNIHKTNlcdlitsllcllvllptkelNEHFKSKLKAPIPTELIVVVAATLSSHFGK  353
                  HIFRNIHKTNLCDLITSLLCLLVLLPTKELNEHFKSKLKAPIPTELIVVVAATLSSHFGK
Sbjct  160604682  HIFRNIHKTNLCDLITSLLCLLVLLPTKELNEHFKSKLKAPIPTELIVVVAATLSSHFGK  160604861

Query  354        LNERYNSSISGHIPTGFMPPKAPDWNLIPNVAVDAIAISIIGFAITVSLSEMFAKKHGYT  413
                  LNERYNSSISGHIPTGFMPPKAPDWNLIPNVAVDAIAISIIGFAITVSLSEMFAKKHGYT
Sbjct  160604862  LNERYNSSISGHIPTGFMPPKAPDWNLIPNVAVDAIAISIIGFAITVSLSEMFAKKHGYT  160605041

Query  414        VKANQEMYAIGFCNIIPSFFHCFATSAALAKTLVKESTGCQTQlssvvtalvlllvllvI  473
                  VKANQEMYAIGFCNIIPSFFHCFATSAALAKTLVKESTGCQTQLSSVVTALVLLLVLLVI
Sbjct  160605042  VKANQEMYAIGFCNIIPSFFHCFATSAALAKTLVKESTGCQTQLSSVVTALVLLLVLLVI  160605221

Query  474        APLFYSLQKSVLGVITIVNLRGALRKFRDLPKMWRLSRMDTVIWFVTMLSSALISTEIGL  533
                  APLFYSLQKSVLGVITIVNLRGALRKFRDLPKMWRLSRMDTVIWFVTMLSSALISTEIGL
Sbjct  160605222  APLFYSLQKSVLGVITIVNLRGALRKFRDLPKMWRLSRMDTVIWFVTMLSSALISTEIGL  160605401

Query  534        LVGVCFSMFCVILRTQKPNVSLLGMEEESEVFESTSAYKNLRTMPGIKIFRFIAPLYYIN  593
                  LVGVCFSMFCVILRTQKPNVSLLGMEEESEVFESTSAYKNLRTMPGIKIFRFIAPLYYIN
Sbjct  160605402  LVGVCFSMFCVILRTQKPNVSLLGMEEESEVFESTSAYKNLRTMPGIKIFRFIAPLYYIN  160605581

Query  594        KECFKSALYKKTLNPILvkavqkkavkrkikEETVTFSRIQDEVSVSLPHDQVELHTIVI  653
                  KECFKSALYKKTLNPILVKAVQKKAVKRKIKEETVTFSRIQDEVSVSLPHDQVELHTIVI
Sbjct  160605582  KECFKSALYKKTLNPILVKAVQKKAVKRKIKEETVTFSRIQDEVSVSLPHDQVELHTIVI  160605761

Query  654        DCSAIQFLDTAGIHTLKEVRRDYNAIGIQVLLAQCNPSVRDSLARGEYCKKEEENLLFYS  713
                  DCSAIQFLDTAGIHTLKEVRRDYNAIGIQVLLAQCNPSVRDSLARGEYCKKEEENLLFYS
Sbjct  160605762  DCSAIQFLDTAGIHTLKEVRRDYNAIGIQVLLAQCNPSVRDSLARGEYCKKEEENLLFYS  160605941

Query  714        LHEAMAFAVDSQIQKEICIPNGESLASE  741
                  LHEAMAFAVDSQIQKEICIPNGESLASE
Sbjct  160605942  LHEAMAFAVDSQIQKEICIPNGESLASE  160606025



Range 2: 160602397 to 160603104

Score:496 bits(1278), Expect:1e-151,
Method:Compositional matrix adjust.,
Identities:236/236(100%), Positives:236/236(100%), Gaps:0/236(0%)

Query  1          MSSESKEQHDLSCRDSSEENDQYNLQSRIHPEPQKEPSPDLKQFWANNQHRPHSRIHIES  60
                  MSSESKEQHDLSCRDSSEENDQYNLQSRIHPEPQKEPSPDLKQFWANNQHRPHSRIHIES
Sbjct  160602397  MSSESKEQHDLSCRDSSEENDQYNLQSRIHPEPQKEPSPDLKQFWANNQHRPHSRIHIES  160602576

Query  61         QEQSHTNCKQFVIKKLQKNCQCSPAKAKNMIFGFLPVLQWLPKYDLKKNILGDVMSGLIV  120
                  QEQSHTNCKQFVIKKLQKNCQCSPAKAKNMIFGFLPVLQWLPKYDLKKNILGDVMSGLIV
Sbjct  160602577  QEQSHTNCKQFVIKKLQKNCQCSPAKAKNMIFGFLPVLQWLPKYDLKKNILGDVMSGLIV  160602756

Query  121        GILLVPQSIAYSLLAGQEPIYGLYTSFFASIIYFLLGTSRHISVGIFGVLCLMIGEVVDR  180
                  GILLVPQSIAYSLLAGQEPIYGLYTSFFASIIYFLLGTSRHISVGIFGVLCLMIGEVVDR
Sbjct  160602757  GILLVPQSIAYSLLAGQEPIYGLYTSFFASIIYFLLGTSRHISVGIFGVLCLMIGEVVDR  160602936

Query  181        ELQEAGYETVHSIPSLGMVSNGSTLLNQTLGEICDKNCYAIRVGSTVTFMAGVYQV  236
                  ELQEAGYETVHSIPSLGMVSNGSTLLNQTLGEICDKNCYAIRVGSTVTFMAGVYQV
Sbjct  160602937  ELQEAGYETVHSIPSLGMVSNGSTLLNQTLGEICDKNCYAIRVGSTVTFMAGVYQV  160603104
```

CH Linnaeus's two-toed sloth *slc26a2*

```
Query: sulfate transporter isoform X1 [Choloepus didactylus] Query ID: XP_037657756.1 Length: 739


>Choloepus didactylus isolate mChoDid1 chromosome 13, mChoDid1.pri, whole genome shotgun sequence
Sequence ID: NC_051319.1 Length: 100229138
Range 1: 94520864 to 94522384

Score:1857 bits(3795), Expect:0.0,
Method:,
Identities:506/507(99%), Positives:507/507(100%), Gaps:0/507(0%)

Query  233       YQVAMGFFQVGFVSVYLSDALLSGFATGASFTILTSQVKYLLGLSIPRSKGVGSLITTWI  292
                 +QVAMGFFQVGFVSVYLSDALLSGFATGASFTILTSQVKYLLGLSIPRSKGVGSLITTWI
Sbjct  94520864  FQVAMGFFQVGFVSVYLSDALLSGFATGASFTILTSQVKYLLGLSIPRSKGVGSLITTWI  94521043

Query  293       HIFRNIHKTNLCDLITSLLCLLVLVPSKELNERFKSKLKAPVPTELIVVVAATLASHFGK  352
                 HIFRNIHKTNLCDLITSLLCLLVLVPSKELNERFKSKLKAPVPTELIVVVAATLASHFGK
Sbjct  94521044  HIFRNIHKTNLCDLITSLLCLLVLVPSKELNERFKSKLKAPVPTELIVVVAATLASHFGK  94521223

Query  353       LNEKYNSSISGHIPTGFMLPKAPDWNLIPSVAVDAIAISIIGFAITVSLSEMFAKKHGYT  412
                 LNEKYNSSISGHIPTGFMLPKAPDWNLIPSVAVDAIAISIIGFAITVSLSEMFAKKHGYT
Sbjct  94521224  LNEKYNSSISGHIPTGFMLPKAPDWNLIPSVAVDAIAISIIGFAITVSLSEMFAKKHGYT  94521403

Query  413       VRANQEMYAIGFCNIIPSFFHCFTTSAALAKTLVKESTGCQTQLSSVVTALVLLLVLLVI  472
                 VRANQEMYAIGFCNIIPSFFHCFTTSAALAKTLVKESTGCQTQLSSVVTALVLLLVLLVI
Sbjct  94521404  VRANQEMYAIGFCNIIPSFFHCFTTSAALAKTLVKESTGCQTQLSSVVTALVLLLVLLVI  94521583

Query  473       APLFYSLQKSVLGVITLVNLRGALRKFRDLPKMWRLSRVDTVIWFVTMLSSALISTEIGL  532
                 APLFYSLQKSVLGVITLVNLRGALRKFRDLPKMWRLSRVDTVIWFVTMLSSALISTEIGL
Sbjct  94521584  APLFYSLQKSVLGVITLVNLRGALRKFRDLPKMWRLSRVDTVIWFVTMLSSALISTEIGL  94521763

Query  533       LVGVCFSMFCVILRTQKPKVSLLGMVEESEVFESTSAYKNLQIKPGIKIFRFVAPLYYIN  592
                 LVGVCFSMFCVILRTQKPKVSLLGMVEESEVFESTSAYKNLQIKPGIKIFRFVAPLYYIN
Sbjct  94521764  LVGVCFSMFCVILRTQKPKVSLLGMVEESEVFESTSAYKNLQIKPGIKIFRFVAPLYYIN  94521943

Query  593       KEYFKSALYKKTLNPILVKAAQKKAEKRKIKEEIVTFSGTQDKVSMHLSRDHVELHTIVI  652
                 KEYFKSALYKKTLNPILVKAAQKKAEKRKIKEEIVTFSGTQDKVSMHLSRDHVELHTIVI
Sbjct  94521944  KEYFKSALYKKTLNPILVKAAQKKAEKRKIKEEIVTFSGTQDKVSMHLSRDHVELHTIVI  94522123

Query  653       DCSAIQYLDTAGIHTLKEVRRDYDAIGIQVLLAQCNPSVRDSLARGEYCKNEEENLFYSL  712
                 DCSAIQYLDTAGIHTLKEVRRDYDAIGIQVLLAQCNPSVRDSLARGEYCKNEEENLFYSL
Sbjct  94522124  DCSAIQYLDTAGIHTLKEVRRDYDAIGIQVLLAQCNPSVRDSLARGEYCKNEEENLFYSL  94522303

Query  713       HEAVAFAVESQTQKGICVPNDVNLASD  739
                 HEAVAFAVESQTQKGICVPNDVNLASD
Sbjct  94522304  HEAVAFAVESQTQKGICVPNDVNLASD  94522384



Range 2: 94518722 to 94519426

Score:875 bits(1787), Expect:0.0,
Method:,
Identities:235/235(100%), Positives:235/235(100%), Gaps:0/235(0%)

Query  1         MSLESKEQHDLSPRDSSEENDQYSLQSRIHPEPQEESTTHFKQFGASDQHRPHSRIHIEP  60
                 MSLESKEQHDLSPRDSSEENDQYSLQSRIHPEPQEESTTHFKQFGASDQHRPHSRIHIEP
Sbjct  94518722  MSLESKEQHDLSPRDSSEENDQYSLQSRIHPEPQEESTTHFKQFGASDQHRPHSRIHIEP  94518901

Query  61        QEQLHTNIKQFVIKKLQKNCQCSPTKAKNTIFGFLPVLHWLPKYDLKKNILGDLMSGLIV  120
                 QEQLHTNIKQFVIKKLQKNCQCSPTKAKNTIFGFLPVLHWLPKYDLKKNILGDLMSGLIV
Sbjct  94518902  QEQLHTNIKQFVIKKLQKNCQCSPTKAKNTIFGFLPVLHWLPKYDLKKNILGDLMSGLIV  94519081

Query  121       GILLVPQSIAYSLLAGQEPVYGLYTSFFASIIYFLLGTSRHISVGIFGVLCLMIGEVVDR  180
                 GILLVPQSIAYSLLAGQEPVYGLYTSFFASIIYFLLGTSRHISVGIFGVLCLMIGEVVDR
Sbjct  94519082  GILLVPQSIAYSLLAGQEPVYGLYTSFFASIIYFLLGTSRHISVGIFGVLCLMIGEVVDR  94519261

Query  181       ELHKAGYETVHIASVGMDSNGSVLLNQTLDGICDKSCYAIRVGSTVTFMAGIYQV  235
                 ELHKAGYETVHIASVGMDSNGSVLLNQTLDGICDKSCYAIRVGSTVTFMAGIYQV
Sbjct  94519262  ELHKAGYETVHIASVGMDSNGSVLLNQTLDGICDKSCYAIRVGSTVTFMAGIYQV  94519426
```

CI African savanna elephant *slc26a2*

```
Query: sulfate transporter [Loxodonta africana] Query ID: XP_023408461.1 Length: 654


>Loxodonta africana isolate ISIS603380 unplaced genomic scaffold, Loxafr3.0 scaffold_1, whole genome shotgun sequence
Sequence ID: NW_003573421.1 Length: 111286942
Range 1: 69470538 to 69471797

Score:1544 bits(3154), Expect:0.0,
Method:,
Identities:419/420(99%), Positives:420/420(100%), Gaps:0/420(0%)

Query  234       YQVAMGFFQVGFVSVYLSDALLSGFVTGASFTILTSQAKYLLGLSLPRSNGVGSLITAWI  293
                 +QVAMGFFQVGFVSVYLSDALLSGFVTGASFTILTSQAKYLLGLSLPRSNGVGSLITAWI
```

```
Sbjct  69470538  FQVAMGFFQVGFVSVYLSDALLSGFVTGASFTILTSQAKYLLGLSLPRSNGVGSLITAWI  69470717

Query  294       HIFRNIHKTNLCDLITSFLCLLVLLPTKELNEHFKSKLKAPIPTELIVVIAATLASHFGK  353
                 HIFRNIHKTNLCDLITSFLCLLVLLPTKELNEHFKSKLKAPIPTELIVVIAATLASHFGK
Sbjct  69470718  HIFRNIHKTNLCDLITSFLCLLVLLPTKELNEHFKSKLKAPIPTELIVVIAATLASHFGK  69470897

Query  354       LNENYNSSIAGHIPTGFMPPKSPDWNLIPSVAVDAIAISIIGFAITVSLSEMFAKKHGYT  413
                 LNENYNSSIAGHIPTGFMPPKSPDWNLIPSVAVDAIAISIIGFAITVSLSEMFAKKHGYT
Sbjct  69470898  LNENYNSSIAGHIPTGFMPPKSPDWNLIPSVAVDAIAISIIGFAITVSLSEMFAKKHGYT  69471077

Query  414       VKANQEMYAIGFCNIIPSFFHCFTTSAALAKTLVKESTGCQTQLSGVVTALVLLLVLLVI  473
                 VKANQEMYAIGFCNIIPSFFHCFTTSAALAKTLVKESTGCQTQLSGVVTALVLLLVLLVI
Sbjct  69471078  VKANQEMYAIGFCNIIPSFFHCFTTSAALAKTLVKESTGCQTQLSGVVTALVLLLVLLVI  69471257

Query  474       APLFFSLQKCVLGVITIVNLRGALLKFRDLPQMWRLSRMDTVIWFVTMLSSALLSTEIGL  533
                 APLFFSLQKCVLGVITIVNLRGALLKFRDLPQMWRLSRMDTVIWFVTMLSSALLSTEIGL
Sbjct  69471258  APLFFSLQKCVLGVITIVNLRGALLKFRDLPQMWRLSRMDTVIWFVTMLSSALLSTEIGL  69471437

Query  534       LVGVCFSMFCVILRTQKPKSSLLGLVEDSEIFESTSVYQNLQTKPGIKIFRFVAPLYYIN  593
                 LVGVCFSMFCVILRTQKPKSSLLGLVEDSEIFESTSVYQNLQTKPGIKIFRFVAPLYYIN
Sbjct  69471438  LVGVCFSMFCVILRTQKPKSSLLGLVEDSEIFESTSVYQNLQTKPGIKIFRFVAPLYYIN  69471617

Query  594       KECFKSALYKKTLNPVLIKAAQRKAAKRKIKEETFRRMQDEVSVHLSHELLELHTIVIDC  653
                 KECFKSALYKKTLNPVLIKAAQRKAAKRKIKEETFRRMQDEVSVHLSHELLELHTIVIDC
Sbjct  69471618  KECFKSALYKKTLNPVLIKAAQRKAAKRKIKEETFRRMQDEVSVHLSHELLELHTIVIDC  69471797
```

Range 2: 69468036 to 69468743

Score:882 bits(1800), Expect:0.0,
Method:,
Identities:236/236(100%), Positives:236/236(100%), Gaps:0/236(0%)

```
Query  1         MSLESKEQQVLSPRDSSEENDQYSLQSRIHLEPQEESSTDFKQFRTNNQCRPHSRIHMEP  60
                 MSLESKEQQVLSPRDSSEENDQYSLQSRIHLEPQEESSTDFKQFRTNNQCRPHSRIHMEP
Sbjct  69468036  MSLESKEQQVLSPRDSSEENDQYSLQSRIHLEPQEESSTDFKQFRTNNQCRPHSRIHMEP  69468215

Query  61        REKSNTDFKHFVIKKLRKTCQCSPAKAKNMIFGFLPVLQWLPKYDLKKNILGDIMSGLIV  120
                 REKSNTDFKHFVIKKLRKTCQCSPAKAKNMIFGFLPVLQWLPKYDLKKNILGDIMSGLIV
Sbjct  69468216  REKSNTDFKHFVIKKLRKTCQCSPAKAKNMIFGFLPVLQWLPKYDLKKNILGDIMSGLIV  69468395

Query  121       GILLVPQSIAYSLLAGQEPIYGLYTSFFASIIYFLLGTSRHISVGIFGVLCLMIGEVVDR  180
                 GILLVPQSIAYSLLAGQEPIYGLYTSFFASIIYFLLGTSRHISVGIFGVLCLMIGEVVDR
Sbjct  69468396  GILLVPQSIAYSLLAGQEPIYGLYTSFFASIIYFLLGTSRHISVGIFGVLCLMIGEVVDR  69468575

Query  181       ELHKAGYDTAHIAPSSGVFSNESTSLNHTLHETCDRSCYAIRVGSTVTFMAGVYQV  236
                 ELHKAGYDTAHIAPSSGVFSNESTSLNHTLHETCDRSCYAIRVGSTVTFMAGVYQV
Sbjct  69468576  ELHKAGYDTAHIAPSSGVFSNESTSLNHTLHETCDRSCYAIRVGSTVTFMAGVYQV  69468743
```

CJ Gray short-tailed opossum *slc26a2*

Query: sulfate transporter [Monodelphis domestica] Query ID: XP_007473924.1 Length: 725

>Monodelphis domestica isolate mMonDom1 chromosome 1, mMonDom1.pri, whole genome shotgun sequence
Sequence ID: NC_077227.1 Length: 760810273
Range 1: 349527610 to 349529133

Score:1863 bits(3807), Expect:0.0,
Method:,
Identities:507/508(99%), Positives:508/508(100%), Gaps:0/508(0%)

```
Query  218        YQVAMGLFQVGFVSVYLSDALLSGFVTGTSFTILTSQAKYLLGLNIPRSSGVGSLITTWI  277
                  +QVAMGLFQVGFVSVYLSDALLSGFVTGTSFTILTSQAKYLLGLNIPRSSGVGSLITTWI
Sbjct  349527610  FQVAMGLFQVGFVSVYLSDALLSGFVTGTSFTILTSQAKYLLGLNIPRSSGVGSLITTWI  349527789

Query  278        YIFKNIHKTNLCDLVTSLLCLLVLVPTKELNEHFKSKLKAPIPTELVVVVAATLASHFGK  337
                  YIFKNIHKTNLCDLVTSLLCLLVLVPTKELNEHFKSKLKAPIPTELVVVVAATLASHFGK
Sbjct  349527790  YIFKNIHKTNLCDLVTSLLCLLVLVPTKELNEHFKSKLKAPIPTELVVVVAATLASHFGK  349527969

Query  338        LNEKYGSSIAGHIPTGFLPPKAPDWNLIPNVAVDAIAISIIGFAITVSLSEMFAKKHGYI  397
                  LNEKYGSSIAGHIPTGFLPPKAPDWNLIPNVAVDAIAISIIGFAITVSLSEMFAKKHGYI
Sbjct  349527970  LNEKYGSSIAGHIPTGFLPPKAPDWNLIPNVAVDAIAISIIGFAITVSLSEMFAKKHGYI  349528149

Query  398        VKANQEMYAIGFCNIIPSFFHCFTTSAALAKTLVKESTGCQTQVSSVVTAMVLLLVLLVV  457
                  VKANQEMYAIGFCNIIPSFFHCFTTSAALAKTLVKESTGCQTQVSSVVTAMVLLLVLLVV
Sbjct  349528150  VKANQEMYAIGFCNIIPSFFHCFTTSAALAKTLVKESTGCQTQVSSVVTAMVLLLVLLVV  349528329

Query  458        APLFYSLQKCVLGVITIVNLRGALRKFGDLPNMWRLSKMDTAIWFITMLSSALISTEIGL  517
                  APLFYSLQKCVLGVITIVNLRGALRKFGDLPNMWRLSKMDTAIWFITMLSSALISTEIGL
Sbjct  349528330  APLFYSLQKCVLGVITIVNLRGALRKFGDLPNMWRLSKMDTAIWFITMLSSALISTEIGL  349528509

Query  518        LIGVCFSMFCVILRTQKPETPILGHVEETEIYESLCAYKNLQTEPGIKIFRFVAPLYYIN  577
                  LIGVCFSMFCVILRTQKPETPILGHVEETEIYESLCAYKNLQTEPGIKIFRFVAPLYYIN
Sbjct  349528510  LIGVCFSMFCVILRTQKPETPILGHVEETEIYESLCAYKNLQTEPGIKIFRFVAPLYYIN  349528689

Query  578        KEGFKSALYKKTVSPILVKAARKKAAKRKLREKMVTFSGCQDDVSMNLSHEPPELHTIVI  637
                  KEGFKSALYKKTVSPILVKAARKKAAKRKLREKMVTFSGCQDDVSMNLSHEPPELHTIVI
Sbjct  349528690  KEGFKSALYKKTVSPILVKAARKKAAKRKLREKMVTFSGCQDDVSMNLSHEPPELHTIVI  349528869
```

```
Query  638        DCSALQFLDTAGIQTLKEVRKDYADIGIQVLLAQCNLSVRSSLYSGEYCTKEEENLLFYS  697
                  DCSALQFLDTAGIQTLKEVRKDYADIGIQVLLAQCNLSVRSSLYSGEYCTKEEENLLFYS
Sbjct  349528870  DCSALQFLDTAGIQTLKEVRKDYADIGIQVLLAQCNLSVRSSLYSGEYCTKEEENLLFYS  349529049

Query  698        VHEALSFALDLQKQKVLNASNGLNTSTD  725
                  VHEALSFALDLQKQKVLNASNGLNTSTD
Sbjct  349529050  VHEALSFALDLQKQKVLNASNGLNTSTD  349529133
```

Range 2: 349524144 to 349524803

Score:830 bits(1694), Expect:0.0,
Method:,
Identities:220/220(100%), Positives:220/220(100%), Gaps:0/220(0%)

```
Query  1          MKDSSPEMSDKEEDHDVDSSFDLAKGDNQYHHYHRILLEPQEKNSTDFKDYVVQKLKKTC  60
                  MKDSSPEMSDKEEDHDVDSSFDLAKGDNQYHHYHRILLEPQEKNSTDFKDYVVQKLKKTC
Sbjct  349524144  MKDSSPEMSDKEEDHDVDSSFDLAKGDNQYHHYHRILLEPQEKNSTDFKDYVVQKLKKTC  349524323

Query  61         CCSPAKAKNCFFGFMPVLQWLPKYDLKKYLLGDIMSGLIVGILLVPQSIAYSLLAGQEPI  120
                  CCSPAKAKNCFFGFMPVLQWLPKYDLKKYLLGDIMSGLIVGILLVPQSIAYSLLAGQEPI
Sbjct  349524324  CCSPAKAKNCFFGFMPVLQWLPKYDLKKYLLGDIMSGLIVGILLVPQSIAYSLLAGQEPI  349524503

Query  121        YGLYTSFFAGIIYFLFGTSRHISVGIFGVLCLMIGEVVDREVHKAGYGMEHKAHPVLFDH  180
                  YGLYTSFFAGIIYFLFGTSRHISVGIFGVLCLMIGEVVDREVHKAGYGMEHKAHPVLFDH
Sbjct  349524504  YGLYTSFFAGIIYFLFGTSRHISVGIFGVLCLMIGEVVDREVHKAGYGMEHKAHPVLFDH  349524683

Query  181        INESIIIPAVNHTSGQICDKSCYAITVGTTVTFIAGVYQV  220
                  INESIIIPAVNHTSGQICDKSCYAITVGTTVTFIAGVYQV
Sbjct  349524684  INESIIIPAVNHTSGQICDKSCYAITVGTTVTFIAGVYQV  349524803
```

CK Platypus *slc26a2*

Query: sulfate transporter [Ornithorhynchus anatinus] Query ID: XP_007671512.1 Length: 717

>Ornithorhynchus anatinus isolate Pmale09 chromosome X1, mOrnAna1.pri.v4, whole genome shotgun sequence
Sequence ID: NC_041749.1 Length: 125040914
Range 1: 17430346 to 17431869

Score:1853 bits(3786), Expect:0.0,
Method:,
Identities:508/508(100%), Positives:508/508(100%), Gaps:0/508(0%)

```
Query  210       QVAMGFFQVGFVSVYLSDALLSGFVTGASFTILTSQAKYLLGLNIPRSNGVGSLVTTWIH  269
                 QVAMGFFQVGFVSVYLSDALLSGFVTGASFTILTSQAKYLLGLNIPRSNGVGSLVTTWIH
Sbjct  17430346  QVAMGFFQVGFVSVYLSDALLSGFVTGASFTILTSQAKYLLGLNIPRSNGVGSLVTTWIH  17430525

Query  270       IFQNIHKTNLCDLITSILSLLVLVPTKELNEHFKAKLKAPIPIELVVIVAATLASHFGQL  329
                 IFQNIHKTNLCDLITSILSLLVLVPTKELNEHFKAKLKAPIPIELVVIVAATLASHFGQL
Sbjct  17430526  IFQNIHKTNLCDLITSILSLLVLVPTKELNEHFKAKLKAPIPIELVVIVAATLASHFGQL  17430705

Query  330       KEKYGSSVAGHIPTGFLPPKAPDWNLIPNVAVDAIAIAVIGFAITVSLSEMFAKKHGYTV  389
                 KEKYGSSVAGHIPTGFLPPKAPDWNLIPNVAVDAIAIAVIGFAITVSLSEMFAKKHGYTV
Sbjct  17430706  KEKYGSSVAGHIPTGFLPPKAPDWNLIPNVAVDAIAIAVIGFAITVSLSEMFAKKHGYTV  17430885

Query  390       KANQEMYAIGFCNIVPAFFHCFTTSAALAKTLVKESTGCQTQVSGVVTALVLLLVLLVIA  449
                 KANQEMYAIGFCNIVPAFFHCFTTSAALAKTLVKESTGCQTQVSGVVTALVLLLVLLVIA
Sbjct  17430886  KANQEMYAIGFCNIVPAFFHCFTTSAALAKTLVKESTGCQTQVSGVVTALVLLLVLLVIA  17431065

Query  450       PLFYSLQKCVLGVITIVNLRGALRKFSDLPRMWKLSRIDTVIWFVTMLASALISTEIGLL  509
                 PLFYSLQKCVLGVITIVNLRGALRKFSDLPRMWKLSRIDTVIWFVTMLASALISTEIGLL
Sbjct  17431066  PLFYSLQKCVLGVITIVNLRGALRKFSDLPRMWKLSRIDTVIWFVTMLASALISTEIGLL  17431245

Query  510       VGVCFSMFCVILRTQKPEASSLGLVEESETYESTAAYKNLRTPPGIKIFRFVAPLYYVNK  569
                 VGVCFSMFCVILRTQKPEASSLGLVEESETYESTAAYKNLRTPPGIKIFRFVAPLYYVNK
Sbjct  17431246  VGVCFSMFCVILRTQKPEASSLGLVEESETYESTAAYKNLRTPPGIKIFRFVAPLYYVNK  17431425

Query  570       ESFKSALFRTTGIDPASVTAERKAAAKRRLREDAVTFSGSQEKVSAHLVHEPLAHHTVVV  629
                 ESFKSALFRTTGIDPASVTAERKAAAKRRLREDAVTFSGSQEKVSAHLVHEPLAHHTVVV
Sbjct  17431426  ESFKSALFRTTGIDPASVTAERKAAAKRRLREDAVTFSGSQEKVSAHLVHEPLAHHTVVV  17431605

Query  630       DCGAVQFLDTAGIATLKAVRKDYEEIGIQLLLAQCNPSVRNSLSSGEYCKQGEEDLLFYS  689
                 DCGAVQFLDTAGIATLKAVRKDYEEIGIQLLLAQCNPSVRNSLSSGEYCKQGEEDLLFYS
Sbjct  17431606  DCGAVQFLDTAGIATLKAVRKDYEEIGIQLLLAQCNPSVRNSLSSGEYCKQGEEDLLFYS  17431785

Query  690       VHDAVCFALESHPQRGQCASNGMYVPGD  717
                 VHDAVCFALESHPQRGQCASNGMYVPGD
Sbjct  17431786  VHDAVCFALESHPQRGQCASNGMYVPGD  17431869
```

Range 2: 17425827 to 17426459

Score:778 bits(1589), Expect:0.0,
Method:,
Identities:211/211(100%), Positives:211/211(100%), Gaps:0/211(0%)

```
Query  1         MSEEGEDNLVHAPDAQVRGSDQWCLHPRILLEPLEKEPVDPKKLAIQKLKGPCRCSAAKV  60
                 MSEEGEDNLVHAPDAQVRGSDQWCLHPRILLEPLEKEPVDPKKLAIQKLKGPCRCSAAKV
Sbjct  17425827  MSEEGEDNLVHAPDAQVRGSDQWCLHPRILLEPLEKEPVDPKKLAIQKLKGPCRCSAAKV  17426006

Query  61        KERVFSFLPVLQWLPKYKFKKYILGDVMSGLIVGILLVPQSIAYSLLAGQEPIYGLYTSF  120
                 KERVFSFLPVLQWLPKYKFKKYILGDVMSGLIVGILLVPQSIAYSLLAGQEPIYGLYTSF
Sbjct  17426007  KERVFSFLPVLQWLPKYKFKKYILGDVMSGLIVGILLVPQSIAYSLLAGQEPIYGLYTSF  17426186

Query  121       FASIIYFLLGTSRHISVGIFGVLCLMIGQVVDREVHRAGYDAEQAVRAAGPVNGTAGPWA  180
                 FASIIYFLLGTSRHISVGIFGVLCLMIGQVVDREVHRAGYDAEQAVRAAGPVNGTAGPWA
Sbjct  17426187  FASIIYFLLGTSRHISVGIFGVLCLMIGQVVDREVHRAGYDAEQAVRAAGPVNGTAGPWA  17426366

Query  181       ANQTSGLGCDRSCYAITVGATVTFVAGVYQV  211
                 ANQTSGLGCDRSCYAITVGATVTFVAGVYQV
Sbjct  17426367  ANQTSGLGCDRSCYAITVGATVTFVAGVYQV  17426459
```

CL Chicken *slc26a2*

```
Query: sulfate transporter [Gallus gallus] Query ID: NP_001376667.1 Length: 715


>Gallus gallus isolate bGalGal1 chromosome 13, bGalGal1.mat.broiler.GRCg7b, whole genome shotgun sequence
Sequence ID: NC_052544.1 Length: 17905061
Range 1: 7962969 to 7964474

Score:1844 bits(3767), Expect:0.0,
Method:,
Identities:502/502(100%), Positives:502/502(100%), Gaps:0/502(0%)

Query  214      QVAMGFFQVGFVSVYLSDSLLSGFVTGASFTILTSQAKYLLGLDIPRSSGVGSLITTWIN  273
                QVAMGFFQVGFVSVYLSDSLLSGFVTGASFTILTSQAKYLLGLDIPRSSGVGSLITTWIN
Sbjct  7964474  QVAMGFFQVGFVSVYLSDSLLSGFVTGASFTILTSQAKYLLGLDIPRSSGVGSLITTWIN  7964295

Query  274      IFRNIHKTNICDVITSFLCFLVLIPTKELNERFKSRLKAPIPVELVVVVAATLASHLGKL  333
                IFRNIHKTNICDVITSFLCFLVLIPTKELNERFKSRLKAPIPVELVVVVAATLASHLGKL
Sbjct  7964294  IFRNIHKTNICDVITSFLCFLVLIPTKELNERFKSRLKAPIPVELVVVVAATLASHLGKL  7964115

Query  334      KETYGSSVAGHIPTGFLPPSPPEWNLIPNVALDAIPIAVIGFAITVSLSEMFAKKHGYTV  393
                KETYGSSVAGHIPTGFLPPSPPEWNLIPNVALDAIPIAVIGFAITVSLSEMFAKKHGYTV
Sbjct  7964114  KETYGSSVAGHIPTGFLPPSPPEWNLIPNVALDAIPIAVIGFAITVSLSEMFAKKHGYTV  7963935

Query  394      KANQEMYAIGFCNIFPSFFHCFTTSAALAKTLIKESTGCRTQVSGIVTSLLILIVLLVIA  453
                KANQEMYAIGFCNIFPSFFHCFTTSAALAKTLIKESTGCRTQVSGIVTSLLILIVLLVIA
Sbjct  7963934  KANQEMYAIGFCNIFPSFFHCFTTSAALAKTLIKESTGCRTQVSGIVTSLLILIVLLVIA  7963755

Query  454      PLFYSLQKCVLAVITIVNLRGALRKFRDLPKMWHLSRVDTVIWLVTMASSALISTEIGLL  513
                PLFYSLQKCVLAVITIVNLRGALRKFRDLPKMWHLSRVDTVIWLVTMASSALISTEIGLL
Sbjct  7963754  PLFYSLQKCVLAVITIVNLRGALRKFRDLPKMWHLSRVDTVIWLVTMASSALISTEIGLL  7963575

Query  514      TGVCFSMLCVIFRTQKPEAPLLGWVAESETYESLSAYKNLETKPGVVVLRFEAPLYYINK  573
                TGVCFSMLCVIFRTQKPEAPLLGWVAESETYESLSAYKNLETKPGVVVLRFEAPLYYINK
Sbjct  7963574  TGVCFSMLCVIFRTQKPEAPLLGWVAESETYESLSAYKNLETKPGVVVLRFEAPLYYINK  7963395

Query  574      ECFKSALYKQTGVNPALVKAAKKKAAKRMLREKEAGSGGNQTSISMELVSEPLGFHTIVI  633
                ECFKSALYKQTGVNPALVKAAKKKAAKRMLREKEAGSGGNQTSISMELVSEPLGFHTIVI
Sbjct  7963394  ECFKSALYKQTGVNPALVKAAKKKAAKRMLREKEAGSGGNQTSISMELVSEPLGFHTIVI  7963215

Query  634      DCCAVQFLDTAGIRTLKEVCKDYNEIDVQVLLAQCNPSVRSSLMRGEFFKEGEDHLLFHS  693
                DCCAVQFLDTAGIRTLKEVCKDYNEIDVQVLLAQCNPSVRSSLMRGEFFKEGEDHLLFHS
Sbjct  7963214  DCCAVQFLDTAGIRTLKEVCKDYNEIDVQVLLAQCNPSVRSSLMRGEFFKEGEDHLLFHS  7963035

Query  694      VHQAVDFALGAQEHSRICASKN  715
                VHQAVDFALGAQEHSRICASKN
Sbjct  7963034  VHQAVDFALGAQEHSRICASKN  7962969



Range 2: 7966271 to 7966915

Score:803 bits(1639), Expect:0.0,
Method:,
Identities:215/215(100%), Positives:215/215(100%), Gaps:0/215(0%)

Query  1        MTAMAEFSNVQSESEMPEGGDAKRGFHHRMFLEPQEEKRNLKALVVKQVKKTCSCTPAKV  60
                MTAMAEFSNVQSESEMPEGGDAKRGFHHRMFLEPQEEKRNLKALVVKQVKKTCSCTPAKV
Sbjct  7966915  MTAMAEFSNVQSESEMPEGGDAKRGFHHRMFLEPQEEKRNLKALVVKQVKKTCSCTPAKV  7966736

Query  61       KDCVLSFFPILQWLPKYNLKECLLGDIMSGVIVGVLLVPQSIAYSLLAGQEPIYGLYTSF  120
                KDCVLSFFPILQWLPKYNLKECLLGDIMSGVIVGVLLVPQSIAYSLLAGQEPIYGLYTSF
Sbjct  7966735  KDCVLSFFPILQWLPKYNLKECLLGDIMSGVIVGVLLVPQSIAYSLLAGQEPIYGLYTSF  7966556

Query  121      FAGIIYCIFGTSHHISVGIFGALCLMVGQVVDREVLRAGYDLEPAAFSDHMDTAMHVNST  180
                FAGIIYCIFGTSHHISVGIFGALCLMVGQVVDREVLRAGYDLEPAAFSDHMDTAMHVNST
Sbjct  7966555  FAGIIYCIFGTSHHISVGIFGALCLMVGQVVDREVLRAGYDLEPAAFSDHMDTAMHVNST  7966376

Query  181      IAPVNQTSQKLLCDKTCYAIKVGATVTFIAGVYQV  215
                IAPVNQTSQKLLCDKTCYAIKVGATVTFIAGVYQV
Sbjct  7966375  IAPVNQTSQKLLCDKTCYAIKVGATVTFIAGVYQV  7966271
```

CM American alligator *slc26a2*

Query: sulfate transporter [Alligator mississippiensis] Query ID: XP_014453123.1 Length: 716

>Alligator mississippiensis isolate rAllMis1 chromosome 9, rAllMis1, whole genome shotgun sequence
Sequence ID: NC_081832.1 Length: 79974352
Range 1: 60154220 to 60155728

Score:1854 bits(3789), Expect:0.0,
Method:,
Identities:502/503(99%), Positives:503/503(100%), Gaps:0/503(0%)

Query 214 YQVAMGFFQVGFVSVYLSDSLLSGFVTGASFTILTSQAKYLLGLDIPRSNGIGSLVTTWI 273
+QVAMGFFQVGFVSVYLSDSLLSGFVTGASFTILTSQAKYLLGLDIPRSNGIGSLVTTWI
Sbjct 60155728 FQVAMGFFQVGFVSVYLSDSLLSGFVTGASFTILTSQAKYLLGLDIPRSNGIGSLVTTWI 60155549

Query 274 NIFRNIHKTNICDLTTSVLCLLVLVPTKEMNEYFKSKLKAPIPIELVVIVAATLASHFGK 333
NIFRNIHKTNICDLTTSVLCLLVLVPTKEMNEYFKSKLKAPIPIELVVIVAATLASHFGK
Sbjct 60155548 NIFRNIHKTNICDLTTSVLCLLVLVPTKEMNEYFKSKLKAPIPIELVVIVAATLASHFGK 60155369

Query 334 LKENYGSGIAGHIPTGFLPPRPPDWDLIPSVALDAVAIAVIGFAITVSLSEMFAKKHGYT 393
LKENYGSGIAGHIPTGFLPPRPPDWDLIPSVALDAVAIAVIGFAITVSLSEMFAKKHGYT
Sbjct 60155368 LKENYGSGIAGHIPTGFLPPRPPDWDLIPSVALDAVAIAVIGFAITVSLSEMFAKKHGYT 60155189

Query 394 VKANQEMYAIGFCNIIPSFFHCFTTSAALAKTLVKESTGCRTQISSVVTALIILLVLLVI 453
VKANQEMYAIGFCNIIPSFFHCFTTSAALAKTLVKESTGCRTQISSVVTALIILLVLLVI
Sbjct 60155188 VKANQEMYAIGFCNIIPSFFHCFTTSAALAKTLVKESTGCRTQISSVVTALIILLVLLVI 60155009

Query 454 APLFYSLQKCVLAVITIVNLRGALRKFRDLPKMWCLSKVDTLIWFVTMIASALVSTEIGL 513
APLFYSLQKCVLAVITIVNLRGALRKFRDLPKMWCLSKVDTLIWFVTMIASALVSTEIGL
Sbjct 60155008 APLFYSLQKCVLAVITIVNLRGALRKFRDLPKMWCLSKVDTLIWFVTMIASALVSTEIGL 60154829

Query 514 LVGVCFSMLCVIFRTQRPEAALLGWAAESEAYESLSSYKNLQTKLGIRVFRFEAPLYYIN 573
LVGVCFSMLCVIFRTQRPEAALLGWAAESEAYESLSSYKNLQTKLGIRVFRFEAPLYYIN
Sbjct 60154828 LVGVCFSMLCVIFRTQRPEAALLGWAAESEAYESLSSYKNLQTKLGIRVFRFEAPLYYIN 60154649

Query 574 KECFKSALYKRTGVNPAWVKTAKKKAEKQMLKEKMMNSEGKQDDISVQLVSEPLDFHTIV 633
KECFKSALYKRTGVNPAWVKTAKKKAEKQMLKEKMMNSEGKQDDISVQLVSEPLDFHTIV
Sbjct 60154648 KECFKSALYKRTGVNPAWVKTAKKKAEKQMLKEKMMNSEGKQDDISVQLVSEPLDFHTIV 60154469

Query 634 VDCCAVQFLDTAGIRTLKEVCRDYEEIGIQVLLAQCNPSVRSSLQRGEYFKKGKRHLLFH 693
VDCCAVQFLDTAGIRTLKEVCRDYEEIGIQVLLAQCNPSVRSSLQRGEYFKKGKRHLLFH
Sbjct 60154468 VDCCAVQFLDTAGIRTLKEVCRDYEEIGIQVLLAQCNPSVRSSLQRGEYFKKGKRHLLFH 60154289

Query 694 SVHQAVDFALGAHMQNGSCASDS 716
SVHQAVDFALGAHMQNGSCASDS
Sbjct 60154288 SVHQAVDFALGAHMQNGSCASDS 60154220

Range 2: 60157590 to 60158237

Score:797 bits(1627), Expect:0.0,
Method:,
Identities:216/216(100%), Positives:216/216(100%), Gaps:0/216(0%)

Query 1 MAAVVELNHILSTSESAEGDGNKLKFHPGMFLEPQEKKIDVKALVVKKAKKACSCTPAKV 60
MAAVVELNHILSTSESAEGDGNKLKFHPGMFLEPQEKKIDVKALVVKKAKKACSCTPAKV
Sbjct 60158237 MAAVVELNHILSTSESAEGDGNKLKFHPGMFLEPQEKKIDVKALVVKKAKKACSCTPAKV 60158058

Query 61 KELIFSFFPVLQWLPKYKLKEYILGDVMSGLIVGILLVPQSIAYSLLAGQEPIYGLYTSF 120
KELIFSFFPVLQWLPKYKLKEYILGDVMSGLIVGILLVPQSIAYSLLAGQEPIYGLYTSF
Sbjct 60158057 KELIFSFFPVLQWLPKYKLKEYILGDVMSGLIVGILLVPQSIAYSLLAGQEPIYGLYTSF 60157878

Query 121 FASIIYFLFGTSRHISVGIFGVLCLMVGQVVDREVQRAGYDLESNIHSSLHRDLVIDINI 180
FASIIYFLFGTSRHISVGIFGVLCLMVGQVVDREVQRAGYDLESNIHSSLHRDLVIDINI
Sbjct 60157877 FASIIYFLFGTSRHISVGIFGVLCLMVGQVVDREVQRAGYDLESNIHSSLHRDLVIDINI 60157698

Query 181 TALPMNQTSQQLLCDKSCYAIAVGSTLTFIAGVYQV 216
TALPMNQTSQQLLCDKSCYAIAVGSTLTFIAGVYQV
Sbjct 60157697 TALPMNQTSQQLLCDKSCYAIAVGSTLTFIAGVYQV 60157590

CN Painted turtle *slc26a2*

Query: sulfate transporter isoform X2 [Chrysemys picta bellii] Query ID: XP_005298074.1 Length: 718

>Chrysemys picta bellii isolate RCT428 chromosome 8, Chrysemys_picta_BioNano-3.0.4, whole genome shotgun sequence
Sequence ID: NC_024225.2 Length: 90858768
Range 1: 14212772 to 14214286

Score:1862 bits(3804), Expect:0.0,

```
Method:,
Identities:504/505(99%), Positives:505/505(100%), Gaps:0/505(0%)

Query  214       YQVAMGFFQVGFVSVYLSDSLLGGFVTGASFTILTSQAKYLLGLDIPRSNGIGSLITTWI  273
                 +QVAMGFFQVGFVSVYLSDSLLGGFVTGASFTILTSQAKYLLGLDIPRSNGIGSLITTWI
Sbjct  14214286  FQVAMGFFQVGFVSVYLSDSLLGGFVTGASFTILTSQAKYLLGLDIPRSNGIGSLITTWI  14214107

Query  274       NIFKNIHKTNICDLITSFLCLLVLVPTKELNEHFKSRLKAPIPIELVVVVAATLASHFGK  333
                 NIFKNIHKTNICDLITSFLCLLVLVPTKELNEHFKSRLKAPIPIELVVVVAATLASHFGK
Sbjct  14214106  NIFKNIHKTNICDLITSFLCLLVLVPTKELNEHFKSRLKAPIPIELVVVVAATLASHFGK  14213927

Query  334       LKENYGSSVAGHIPTGFLPPQPPKWDLIPSVAMDAVAIAIIGFAITVSLSEMFAKKHGYT  393
                 LKENYGSSVAGHIPTGFLPPQPPKWDLIPSVAMDAVAIAIIGFAITVSLSEMFAKKHGYT
Sbjct  14213926  LKENYGSSVAGHIPTGFLPPQPPKWDLIPSVAMDAVAIAIIGFAITVSLSEMFAKKHGYT  14213747

Query  394       VKANQEMYAIGFCNIIPSFFHCFTTSAALAKTLVKESTGCRTQISGVVTALVILLVLLI  453
                 VKANQEMYAIGFCNIIPSFFHCFTTSAALAKTLVKESTGCRTQISGVVTALVILLVLLI
Sbjct  14213746  VKANQEMYAIGFCNIIPSFFHCFTTSAALAKTLVKESTGCRTQISGVVTALVILLVLLI  14213567

Query  454       APLFYSLQKCVLGVITIVNLRGALRKFRDLPKMWHLSKVDTVIWLVTMAASALLSTEIGL  513
                 APLFYSLQKCVLGVITIVNLRGALRKFRDLPKMWHLSKVDTVIWLVTMAASALLSTEIGL
Sbjct  14213566  APLFYSLQKCVLGVITIVNLRGALRKFRDLPKMWHLSKVDTVIWLVTMAASALLSTEIGL  14213387

Query  514       LIGVCFSMLCVIVRTQRPEAPLLGWVVETEMYESLSAYKNLKTKPGIVVFRFEAPLYYIN  573
                 LIGVCFSMLCVIVRTQRPEAPLLGWVVETEMYESLSAYKNLKTKPGIVVFRFEAPLYYIN
Sbjct  14213386  LIGVCFSMLCVIVRTQRPEAPLLGWVVETEMYESLSAYKNLKTKPGIVVFRFEAPLYYIN  14213207

Query  574       KECFKSTLYKRTGVNPAWVKAAKKKAAKRMLKKKMVNSGGSQADVAVQLVTEPLVFHTLV  633
                 KECFKSTLYKRTGVNPAWVKAAKKKAAKRMLKKKMVNSGGSQADVAVQLVTEPLVFHTLV
Sbjct  14213206  KECFKSTLYKRTGVNPAWVKAAKKKAAKRMLKKKMVNSGGSQADVAVQLVTEPLVFHTLV  14213027

Query  634       IDCCAIQFLDTAGIHTLKEICKDYGEIGIQVLLAQCNASVRSSLHRGEYFKKEEQNLLFH  693
                 IDCCAIQFLDTAGIHTLKEICKDYGEIGIQVLLAQCNASVRSSLHRGEYFKKEEQNLLFH
Sbjct  14213026  IDCCAIQFLDTAGIHTLKEICKDYGEIGIQVLLAQCNASVRSSLHRGEYFKKEEQNLLFH  14212847

Query  694       SVHQAVDFALGAHKQNGCWEGNNCV  718
                 SVHQAVDFALGAHKQNGCWEGNNCV
Sbjct  14212846  SVHQAVDFALGAHKQNGCWEGNNCV  14212772
```

```
Range 2: 14216022 to 14216669

Score:807 bits(1647), Expect:0.0,
Method:,
Identities:216/216(100%), Positives:216/216(100%), Gaps:0/216(0%)

Query  1         MAAEVELNHVQSTSEMAEGEDDEHIFQPMMFLEPQQTSIDMKALLVKKVKKTCSCTPAKV  60
                 MAAEVELNHVQSTSEMAEGEDDEHIFQPMMFLEPQQTSIDMKALLVKKVKKTCSCTPAKV
Sbjct  14216669  MAAEVELNHVQSTSEMAEGEDDEHIFQPMMFLEPQQTSIDMKALLVKKVKKTCSCTPAKV  14216490

Query  61        KDLIFSFLPVLQWLPKYKLREYILGDIMSGVIVGILLVPQSIAYSLLAGQEPIYGLYTSF  120
                 KDLIFSFLPVLQWLPKYKLREYILGDIMSGVIVGILLVPQSIAYSLLAGQEPIYGLYTSF
Sbjct  14216489  KDLIFSFLPVLQWLPKYKLREYILGDIMSGVIVGILLVPQSIAYSLLAGQEPIYGLYTSF  14216310

Query  121       FASIIYFLFGTSRHISVGIFGVLCLMVGQVVDREVQRAGYDIEPSVHSGLQRDMVSYVNI  180
                 FASIIYFLFGTSRHISVGIFGVLCLMVGQVVDREVQRAGYDIEPSVHSGLQRDMVSYVNI
Sbjct  14216309  FASIIYFLFGTSRHISVGIFGVLCLMVGQVVDREVQRAGYDIEPSVHSGLQRDMVSYVNI  14216130

Query  181       TTLAVNQTSQPLLCDRSCYAITVGATVTFIAGVYQV  216
                 TTLAVNQTSQPLLCDRSCYAITVGATVTFIAGVYQV
Sbjct  14216129  TTLAVNQTSQPLLCDRSCYAITVGATVTFIAGVYQV  14216022
```

CO Green anole *slc26a2*

```
Query: PREDICTED: sulfate transporter [Anolis carolinensis] Query ID: XP_003217385.1 Length: 714


>Anolis carolinensis chromosome 2, AnoCar2.0, whole genome shotgun sequence
Sequence ID: NC_014777.1 Length: 199619895
Range 1: 122090529 to 122092055

Score:1878 bits(3838), Expect:0.0,
Method:,
Identities:509/509(100%), Positives:509/509(100%), Gaps:0/509(0%)

Query  206        IAMGFFQVGFISVYLSDSLLSGFVTGASFTILTSQAKYLLGLDIPRSNGIGSFITTWINI  265
                  IAMGFFQVGFISVYLSDSLLSGFVTGASFTILTSQAKYLLGLDIPRSNGIGSFITTWINI
Sbjct  122092055  IAMGFFQVGFISVYLSDSLLSGFVTGASFTILTSQAKYLLGLDIPRSNGIGSFITTWINI  122091876

Query  266        FKNIHKTNFCDLITSFLCLLVLIPTKELNERYKSKLKAPLPTELFVVIVATLVSHFGKLK  325
                  FKNIHKTNFCDLITSFLCLLVLIPTKELNERYKSKLKAPLPTELFVVIVATLVSHFGKLK
Sbjct  122091875  FKNIHKTNFCDLITSFLCLLVLIPTKELNERYKSKLKAPLPTELFVVIVATLVSHFGKLK  122091696

Query  326        EKYGSSVSGHIPTGFLPPQPPDWGLIPSIALDAVAIAIIGFAITVSLSEMFAKKHGYTVK  385
                  EKYGSSVSGHIPTGFLPPQPPDWGLIPSIALDAVAIAIIGFAITVSLSEMFAKKHGYTVK
Sbjct  122091695  EKYGSSVSGHIPTGFLPPQPPDWGLIPSIALDAVAIAIIGFAITVSLSEMFAKKHGYTVK  122091516

Query  386        PNQEMYAIGFCNIIPSFFHCITTSAALAKTLVKESTGCRTQVSGVVTALVILLVLLVIAP  445
                  PNQEMYAIGFCNIIPSFFHCITTSAALAKTLVKESTGCRTQVSGVVTALVILLVLLVIAP
```

```
Sbjct  122091515  PNQEMYAIGFCNIIPSFFHCITTSAALAKTLVKESTGCRTQVSGVVTALVILLVLLVIAP  122091336

Query  446        LFYSLQKCVLGVITIVNLRGALRKFGDLPKMWQLGKVDTVIWTITMLSSALISTELGLLI  505
                  LFYSLQKCVLGVITIVNLRGALRKFGDLPKMWQLGKVDTVIWTITMLSSALISTELGLLI
Sbjct  122091335  LFYSLQKCVLGVITIVNLRGALRKFGDLPKMWQLGKVDTVIWTITMLSSALISTELGLLI  122091156

Query  506        GVCFSLLCVVLRTQRPEGQLLGWVPDSEIYEPLPAYKDLQTKPSIKVFRFEAPIYYANKE  565
                  GVCFSLLCVVLRTQRPEGQLLGWVPDSEIYEPLPAYKDLQTKPSIKVFRFEAPIYYANKE
Sbjct  122091155  GVCFSLLCVVLRTQRPEGQLLGWVPDSEIYEPLPAYKDLQTKPSIKVFRFEAPIYYANKE  122090976

Query  566        SFKSMLYKQTGVNPVWELAAKRKTEKRTKKTASANGNQAEVSVQLFTQDFEFHTIVIDCC  625
                  SFKSMLYKQTGVNPVWELAAKRKTEKRTKKTASANGNQAEVSVQLFTQDFEFHTIVIDCC
Sbjct  122090975  SFKSMLYKQTGVNPVWELAAKRKTEKRTKKTASANGNQAEVSVQLFTQDFEFHTIVIDCC  122090796

Query  626        AVHFLDTAGIHTLKEIHKDYEEIGIQVLLAQCNPSVRDSLHRGEYIKKGEKKFLFHSVHQ  685
                  AVHFLDTAGIHTLKEIHKDYEEIGIQVLLAQCNPSVRDSLHRGEYIKKGEKKFLFHSVHQ
Sbjct  122090795  AVHFLDTAGIHTLKEIHKDYEEIGIQVLLAQCNPSVRDSLHRGEYIKKGEKKFLFHSVHQ  122090616

Query  686        AVEYALCSSKQNGCCAPEGQCKGKDLAES  714
                  AVEYALCSSKQNGCCAPEGQCKGKDLAES
Sbjct  122090615  AVEYALCSSKQNGCCAPEGQCKGKDLAES  122090529
```

Range 2: 122095223 to 122095840

Score:769 bits(1570), Expect:0.0,
Method:,
Identities:205/206(99%), Positives:206/206(100%), Gaps:0/206(0%)

```
Query  1          MAEANHVNSVFELAEVHDGERNHHPRIILEPIDKRTDIKAWIVKKVKKKCSCTPAIAKDL  60
                  MAEANHVNSVFELAEVHDGERNHHPRIILEPIDKRTDIKAWIVKKVKKKCSCTPAIAKDL
Sbjct  122095840  MAEANHVNSVFELAEVHDGERNHHPRIILEPIDKRTDIKAWIVKKVKKKCSCTPAIAKDL  122095661

Query  61         VFSFLPVLKWLPKYNVKEDLLGDLMSGLIVGILLVPQSIAYSLLAGQEPIYGLYTSFFAS  120
                  VFSFLPVLKWLPKYNVKEDLLGDLMSGLIVGILLVPQSIAYSLLAGQEPIYGLYTSFFAS
Sbjct  122095660  VFSFLPVLKWLPKYNVKEDLLGDLMSGLIVGILLVPQSIAYSLLAGQEPIYGLYTSFFAS  122095481

Query  121        IIYFLFGTSRHISVGIFGVLCLMIGEVVDREVQKAGYDLDIHVYINSSSTMGPLNMNQTS  180
                  IIYFLFGTSRHISVGIFGVLCLMIGEVVDREVQKAGYDLDIHVYINSSSTMGPLNMNQTS
Sbjct  122095480  IIYFLFGTSRHISVGIFGVLCLMIGEVVDREVQKAGYDLDIHVYINSSSTMGPLNMNQTS  122095301

Query  181        QTFCDKSCYAIIVGSTVTFMAGIYQI  206
                  QTFCDKSCYAIIVGSTVTFMAGIYQ+
Sbjct  122095300  QTFCDKSCYAIIVGSTVTFMAGIYQV  122095223
```

CP Western clawed frog *slc26a2*

Query: sulfate transporter [Xenopus tropicalis] Query ID: XP_002943195.1 Length: 711

>Xenopus tropicalis strain Nigerian chromosome 3, UCB_Xtro_10.0, whole genome shotgun sequence
Sequence ID: NC_030679.2 Length: 153873357
Range 1: 44122411 to 44123919

Score:1859 bits(3798), Expect:0.0,
Method:,
Identities:503/503(100%), Positives:503/503(100%), Gaps:0/503(0%)

```
Query  209       QLAMGFLQVGFVSVYLSDSLLSGFATGASFTILTSQVKYLLGISIPRANGIGSLIYTWIY  268
                 QLAMGFLQVGFVSVYLSDSLLSGFATGASFTILTSQVKYLLGISIPRANGIGSLIYTWIY
Sbjct  44122411  QLAMGFLQVGFVSVYLSDSLLSGFATGASFTILTSQVKYLLGISIPRANGIGSLIYTWIY  44122590

Query  269       IFQNIHLTNICDLVTSILCLLVLIPAKELNECYKSKLKAPIPIELLVVVAATLASHFGHL  328
                 IFQNIHLTNICDLVTSILCLLVLIPAKELNECYKSKLKAPIPIELLVVVAATLASHFGHL
Sbjct  44122591  IFQNIHLTNICDLVTSILCLLVLIPAKELNECYKSKLKAPIPIELLVVVAATLASHFGHL  44122770

Query  329       KENYGSSIAGTIPTGFLMPKSPDWSLIPSIALPALSIAIIGFAITVSLSEMFAKKHGYEV  388
                 KENYGSSIAGTIPTGFLMPKSPDWSLIPSIALPALSIAIIGFAITVSLSEMFAKKHGYEV
Sbjct  44122771  KENYGSSIAGTIPTGFLMPKSPDWSLIPSIALPALSIAIIGFAITVSLSEMFAKKHGYEV  44122950

Query  389       KANQEMYAIGFCNIIPAFFHCFTTSAALAKTLVKESTGCKTQVSGIMTSLVLLLVLLVIA  448
                 KANQEMYAIGFCNIIPAFFHCFTTSAALAKTLVKESTGCKTQVSGIMTSLVLLLVLLVIA
Sbjct  44122951  KANQEMYAIGFCNIIPAFFHCFTTSAALAKTLVKESTGCKTQVSGIMTSLVLLLVLLVIA  44123130

Query  449       PLFFSLQKCVLGVITIVNLRGALRKFLDLPKMWKVNKVDTTIWLVTMFSSALISTELGLL  508
                 PLFFSLQKCVLGVITIVNLRGALRKFLDLPKMWKVNKVDTTIWLVTMFSSALISTELGLL
Sbjct  44123131  PLFFSLQKCVLGVITIVNLRGALRKFLDLPKMWKVNKVDTTIWLVTMFSSALISTELGLL  44123310

Query  509       IGIAFSMFCVIVRSQKPVSTLLGQVDNTEVYESMNAYKNLNIPQGIKIFHFEAPIYYVNK  568
                 IGIAFSMFCVIVRSQKPVSTLLGQVDNTEVYESMNAYKNLNIPQGIKIFHFEAPIYYVNK
Sbjct  44123311  IGIAFSMFCVIVRSQKPVSTLLGQVDNTEVYESMNAYKNLNIPQGIKIFHFEAPIYYVNK  44123490

Query  569       QHFKMTLYKYSGVDPVSVATAKRKKAKKIEKLNRQDGSMEIKTTECLYKEQQPVHTLIID  628
                 QHFKMTLYKYSGVDPVSVATAKRKKAKKIEKLNRQDGSMEIKTTECLYKEQQPVHTLIID
Sbjct  44123491  QHFKMTLYKYSGVDPVSVATAKRKKAKKIEKLNRQDGSMEIKTTECLYKEQQPVHTLIID  44123670

Query  629       CGAIQFIDTAGMNTLKEVLKDYSDIGIQLLLAQCNVTVKNSLRNGDFFKREENTVMFHSV  688
                 CGAIQFIDTAGMNTLKEVLKDYSDIGIQLLLAQCNVTVKNSLRNGDFFKREENTVMFHSV
Sbjct  44123671  CGAIQFIDTAGMNTLKEVLKDYSDIGIQLLLAQCNVTVKNSLRNGDFFKREENTVMFHSV  44123850
```

```
Query  689       HQAVQFALYVQQQSDAHLNGSAV  711
                 HQAVQFALYVQQQSDAHLNGSAV
Sbjct  44123851  HQAVQFALYVQQQSDAHLNGSAV  44123919
```

Range 2: 44116684 to 44117310

Score:786 bits(1604), Expect:0.0,
Method:,
Identities:209/209(100%), Positives:209/209(100%), Gaps:0/209(0%)

```
Query  1         MDTESNEDTKAVYSCSYPPGTIYLEEKEKKPIRLKRRLAEKIRKTCCCSSGRAKELLLSF  60
                 MDTESNEDTKAVYSCSYPPGTIYLEEKEKKPIRLKRRLAEKIRKTCCCSSGRAKELLLSF
Sbjct  44116684  MDTESNEDTKAVYSCSYPPGTIYLEEKEKKPIRLKRRLAEKIRKTCCCSSGRAKELLLSF  44116863

Query  61        FPVLQWLPSYNLKEYLLGDMMSGLIVAILLVPQSIAYSLLAGQEPIFGLYTSFFACIIYF  120
                 FPVLQWLPSYNLKEYLLGDMMSGLIVAILLVPQSIAYSLLAGQEPIFGLYTSFFACIIYF
Sbjct  44116864  FPVLQWLPSYNLKEYLLGDMMSGLIVAILLVPQSIAYSLLAGQEPIFGLYTSFFACIIYF  44117043

Query  121       LMGTSRHIHVGIFGVLCLMVGEVVDRELQVAGYDSLDSHKGQMAVLDALHENSTHSPFDH  180
                 LMGTSRHIHVGIFGVLCLMVGEVVDRELQVAGYDSLDSHKGQMAVLDALHENSTHSPFDH
Sbjct  44117044  LMGTSRHIHVGIFGVLCLMVGEVVDRELQVAGYDSLDSHKGQMAVLDALHENSTHSPFDH  44117223

Query  181       NHTVAMTCDRSCYAITVGATVTFMAGVYQ  209
                 NHTVAMTCDRSCYAITVGATVTFMAGVYQ
Sbjct  44117224  NHTVAMTCDRSCYAITVGATVTFMAGVYQ  44117310
```

CQ Two-lined caecilian *slc26a2*

Query: sulfate transporter [Rhinatrema bivittatum] Query ID: XP_029439133.1 Length: 731

>Rhinatrema bivittatum chromosome 18, aRhiBiv1.1, whole genome shotgun sequence
Sequence ID: NC_042632.1 Length: 61353747
Range 1: 56873827 to 56875362

Score:1874 bits(3829), Expect:0.0,
Method:,
Identities:511/512(99%), Positives:512/512(100%), Gaps:0/512(0%)

```
Query  220       YQVAMGLLQVGFVSVYLSDSLLSGFATGASFTILTSQVKYLLGISIPRSGGVGSLLRTWI  279
                 +QVAMGLLQVGFVSVYLSDSLLSGFATGASFTILTSQVKYLLGISIPRSGGVGSLLRTWI
Sbjct  56873827  FQVAMGLLQVGFVSVYLSDSLLSGFATGASFTILTSQVKYLLGISIPRSGGVGSLLRTWI  56874006

Query  280       YIFQNIHKTNICDLVTSTLCLLVLIPTKEINECYKSKLKAPIPIELFVVIAATLTSHFGH  339
                 YIFQNIHKTNICDLVTSTLCLLVLIPTKEINECYKSKLKAPIPIELFVVIAATLTSHFGH
Sbjct  56874007  YIFQNIHKTNICDLVTSTLCLLVLIPTKEINECYKSKLKAPIPIELFVVIAATLTSHFGH  56874186

Query  340       LNERYGSSVAGDIPTGFMLPKTPDWGLIPNVAVGAFSIAIIGFAITVTLSEMFAKKHGYT  399
                 LNERYGSSVAGDIPTGFMLPKTPDWGLIPNVAVGAFSIAIIGFAITVTLSEMFAKKHGYT
Sbjct  56874187  LNERYGSSVAGDIPTGFMLPKTPDWGLIPNVAVGAFSIAIIGFAITVTLSEMFAKKHGYT  56874366

Query  400       VKANQEMYAIGFCNIIPAFFHCFTTSAALAKTLVKESTGCKTQISGVVTALVILLVLLVV  459
                 VKANQEMYAIGFCNIIPAFFHCFTTSAALAKTLVKESTGCKTQISGVVTALVILLVLLVV
Sbjct  56874367  VKANQEMYAIGFCNIIPAFFHCFTTSAALAKTLVKESTGCKTQISGVVTALVILLVLLVV  56874546

Query  460       APLFYSLQKCVLGVITIVNLRGALRKFVDLPNMWKLSKVDTAIWLVTMASTALISTELGL  519
                 APLFYSLQKCVLGVITIVNLRGALRKFVDLPNMWKLSKVDTAIWLVTMASTALISTELGL
Sbjct  56874547  APLFYSLQKCVLGVITIVNLRGALRKFVDLPNMWKLSKVDTAIWLVTMASTALISTELGL  56874726

Query  520       LIGVCFSMLCIILRTQMPAAMLLGQVDGTEIYESLSAYKNLKTMPDIQIFRFEAPLCYVN  579
                 LIGVCFSMLCIILRTQMPAAMLLGQVDGTEIYESLSAYKNLKTMPDIQIFRFEAPLCYVN
Sbjct  56874727  LIGVCFSMLCIILRTQMPAAMLLGQVDGTEIYESLSAYKNLKTMPDIQIFRFEAPLCYVN  56874906

Query  580       KHHFKSALYKQTGLNPASVLAAEKKAAKSRLREEKKEAVVSFSSGQQEGEVSVQLFQERL  639
                 KHHFKSALYKQTGLNPASVLAAEKKAAKSRLREEKKEAVVSFSSGQQEGEVSVQLFQERL
Sbjct  56874907  KHHFKSALYKQTGLNPASVLAAEKKAAKSRLREEKKEAVVSFSSGQQEGEVSVQLFQERL  56875086

Query  640       PFHTLVIDCAAIQFLDTVGISTLREVLREYKELGIRVLLAQCSPSVRASLRSGDYFKSEE  699
                 PFHTLVIDCAAIQFLDTVGISTLREVLREYKELGIRVLLAQCSPSVRASLRSGDYFKSEE
Sbjct  56875087  PFHTLVIDCAAIQFLDTVGISTLREVLREYKELGIRVLLAQCSPSVRASLRSGDYFKSEE  56875266

Query  700       DNLLFYSLHQAICFVQSEQQQNGSCAAGDASC  731
                 DNLLFYSLHQAICFVQSEQQQNGSCAAGDASC
Sbjct  56875267  DNLLFYSLHQAICFVQSEQQQNGSCAAGDASC  56875362
```

Range 2: 56869413 to 56870078

Score:813 bits(1660), Expect:0.0,
Method:,
Identities:222/222(100%), Positives:222/222(100%), Gaps:0/222(0%)

```
Query  1         MAGESPDESSFSPVLAGAAAQNGEGLGRHRAGKIFLEKREVACAGVKKLLAETVKENVSC  60
                 MAGESPDESSFSPVLAGAAAQNGEGLGRHRAGKIFLEKREVACAGVKKLLAETVKENVSC
Sbjct  56869413  MAGESPDESSFSPVLAGAAAQNGEGLGRHRAGKIFLEKREVACAGVKKLLAETVKENVSC  56869592
```

```
Query  61        TPARFKDLLFSFFPVLQWLPKYRKEYVLGDVMSGLIVGILLVPQSIAYSLLAGQEPIYGL  120
                 TPARFKDLLFSFFPVLQWLPKYRKEYVLGDVMSGLIVGILLVPQSIAYSLLAGQEPIYGL
Sbjct  56869593  TPARFKDLLFSFFPVLQWLPKYRKEYVLGDVMSGLIVGILLVPQSIAYSLLAGQEPIYGL  56869772

Query  121       YTSFFACLIYFLMGTSRHISVGIFGVLCLMVGEVVEQELEAAGYNAFHESQDGLHANITS  180
                 YTSFFACLIYFLMGTSRHISVGIFGVLCLMVGEVVEQELEAAGYNAFHESQDGLHANITS
Sbjct  56869773  YTSFFACLIYFLMGTSRHISVGIFGVLCLMVGEVVEQELEAAGYNAFHESQDGLHANITS  56869952

Query  181       YERNSTLSPATANQSAGLPCDRSCYAITVGVTVTFMAGVYQV  222
                 YERNSTLSPATANQSAGLPCDRSCYAITVGVTVTFMAGVYQV
Sbjct  56869953  YERNSTLSPATANQSAGLPCDRSCYAITVGVTVTFMAGVYQV  56870078
```

CR West african lungfish *slc26a2*

Query: sulfate transporter [Protopterus annectens] Query ID: XP_043925149.1 Length: 727

>Protopterus annectens isolate FY-2018-M chromosome 4.part0, PAN1.0, whole genome shotgun sequence
Sequence ID: NC_056733.1 Length: 2000000000
Range 1: 1353410115 to 1353411638

Score:1879 bits(3840), Expect:0.0,
Method:,
Identities:508/508(100%), Positives:508/508(100%), Gaps:0/508(0%)

```
Query  220         VVMGLFQVGFISVYLSDSLLSGFATGASLTILTSQVKYLLGISIPRSHGVGSLIFTWYHI  279
                   VVMGLFQVGFISVYLSDSLLSGFATGASLTILTSQVKYLLGISIPRSHGVGSLIFTWYHI
Sbjct  1353411638  VVMGLFQVGFISVYLSDSLLSGFATGASLTILTSQVKYLLGISIPRSHGVGSLIFTWYHI  1353411459

Query  280         FKNIYKTNICDLITSLLCLALLVPAKEINELYKSKLKAPIPMELFVVIAATLASHFGNLK  339
                   FKNIYKTNICDLITSLLCLALLVPAKEINELYKSKLKAPIPMELFVVIAATLASHFGNLK
Sbjct  1353411458  FKNIYKTNICDLITSLLCLALLVPAKEINELYKSKLKAPIPMELFVVIAATLASHFGNLK  1353411279

Query  340         EKYGSSIAGTIPTGFLPPSPPDWSLIPRIAGDAIAIAIIGFAITVSLSEMFAKKHGYTVR  399
                   EKYGSSIAGTIPTGFLPPSPPDWSLIPRIAGDAIAIAIIGFAITVSLSEMFAKKHGYTVR
Sbjct  1353411278  EKYGSSIAGTIPTGFLPPSPPDWSLIPRIAGDAIAIAIIGFAITVSLSEMFAKKHGYTVR  1353411099

Query  400         ANQEMYAIGFCNIIPAFFHCFTTSAALAKTLVKESTGCQTQISGVITSGLLLLVLLVIAP  459
                   ANQEMYAIGFCNIIPAFFHCFTTSAALAKTLVKESTGCQTQISGVITSGLLLLVLLVIAP
Sbjct  1353411098  ANQEMYAIGFCNIIPAFFHCFTTSAALAKTLVKESTGCQTQISGVITSGLLLLVLLVIAP  1353410919

Query  460         LFYSLQKCVLGVITIVNLRGALRKFLDLPKMWQVNRTDTMIWLVTMASSAFISTELGLVI  519
                   LFYSLQKCVLGVITIVNLRGALRKFLDLPKMWQVNRTDTMIWLVTMASSAFISTELGLVI
Sbjct  1353410918  LFYSLQKCVLGVITIVNLRGALRKFLDLPKMWQVNRTDTMIWLVTMASSAFISTELGLVI  1353410739

Query  520         GVCFSMLCVIIRTQQARSTLLGWVDGTEIYEDQAVYKNLSSVQGVEIFRFEAPLYYANKE  579
                   GVCFSMLCVIIRTQQARSTLLGWVDGTEIYEDQAVYKNLSSVQGVEIFRFEAPLYYANKE
Sbjct  1353410738  GVCFSMLCVIIRTQQARSTLLGWVDGTEIYEDQAVYKNLSSVQGVEIFRFEAPLYYANKE  1353410559

Query  580         YFRSSLYKRTGINPISLISARKKIEKKLREQNQTDTANHDSSKKVDVVLPLCHDEAKFHT  639
                   YFRSSLYKRTGINPISLISARKKIEKKLREQNQTDTANHDSSKKVDVVLPLCHDEAKFHT
Sbjct  1353410558  YFRSSLYKRTGINPISLISARKKIEKKLREQNQTDTANHDSSKKVDVVLPLCHDEAKFHT  1353410379

Query  640         LIIDCSSVTFVDTVGVNILKEILKDFKEIGIQVLLAHCNPSIIDSLRRGGYLQKEDHSLL  699
                   LIIDCSSVTFVDTVGVNILKEILKDFKEIGIQVLLAHCNPSIIDSLRRGGYLQKEDHSLL
Sbjct  1353410378  LIIDCSSVTFVDTVGVNILKEILKDFKEIGIQVLLAHCNPSIIDSLRRGGYLQKEDHSLL  1353410199

Query  700         FHNIHEAVCFCKIKDVENGYCPGDETRC  727
                   FHNIHEAVCFCKIKDVENGYCPGDETRC
Sbjct  1353410198  FHNIHEAVCFCKIKDVENGYCPGDETRC  1353410115
```

Range 2: 1353519321 to 1353519983

Score:812 bits(1657), Expect:0.0,
Method:,
Identities:220/221(99%), Positives:221/221(100%), Gaps:0/221(0%)

```
Query  1           MTTSGRSDNTSKMTELVAVGSVKDPAICTKTVLQEQDKETVPLKKYITKTLKKHCSCAPS  60
                   MTTSGRSDNTSKMTELVAVGSVKDPAICTKTVLQEQDKETVPLKKYITKTLKKHCSCAPS
Sbjct  1353519983  MTTSGRSDNTSKMTELVAVGSVKDPAICTKTVLQEQDKETVPLKKYITKTLKKHCSCAPS  1353519804

Query  61          QAKRLLFDFFPVLKWLPKYRFRDWILGDVMSGLIVGILLVPQSIAYSLLASQEPVFGLYT  120
                   QAKRLLFDFFPVLKWLPKYRFRDWILGDVMSGLIVGILLVPQSIAYSLLASQEPVFGLYT
Sbjct  1353519803  QAKRLLFDFFPVLKWLPKYRFRDWILGDVMSGLIVGILLVPQSIAYSLLASQEPVFGLYT  1353519624

Query  121         SFFACLIYFLLGTSRHISVGIFGVLCLMVGQVVDRELQFAGYDLDSTDQNSLTHVELGNS  180
                   SFFACLIYFLLGTSRHISVGIFGVLCLMVGQVVDRELQFAGYDLDSTDQNSLTHVELGNS
Sbjct  1353519623  SFFACLIYFLLGTSRHISVGIFGVLCLMVGQVVDRELQFAGYDLDSTDQNSLTHVELGNS  1353519444

Query  181         TNVTEGVQTLNASAGILCNKSCYAITVGATLTFMAGVYQVV  221
                   TNVTEGVQTLNASAGILCNKSCYAITVGATLTFMAGVYQV+
Sbjct  1353519443  TNVTEGVQTLNASAGILCNKSCYAITVGATLTFMAGVYQVI  1353519321
```

CS Coelacanth *slc26a2*

Query: PREDICTED: sulfate transporter isoform X2 [Latimeria chalumnae] Query ID: XP_006003846.1 Length: 723

>Latimeria chalumnae isolate SAIAB 97564 unplaced genomic scaffold, LatCha1 scaffold00970, whole genome shotgun sequence
Sequence ID: NW_005819980.1 Length: 831416
Range 1: 749772 to 751301

Score:1880 bits(3841), Expect:0.0,
Method:,
Identities:509/510(99%), Positives:510/510(100%), Gaps:0/510(0%)

```
Query  214     YQVTMGFCQVGFVSVYLSDSLLSGFATGASLTILTSQVKYLLGISIPRNHGLGSLIFTWI  273
               +QVTMGFCQVGFVSVYLSDSLLSGFATGASLTILTSQVKYLLGISIPRNHGLGSLIFTWI
Sbjct  749772  FQVTMGFCQVGFVSVYLSDSLLSGFATGASLTILTSQVKYLLGISIPRNHGLGSLIFTWI  749951

Query  274     DIFKNIHRTNFCDVITSLLCLLVLVPAKEINECYKAKLKAPIPAELFVVIIATLASHFGK  333
               DIFKNIHRTNFCDVITSLLCLLVLVPAKEINECYKAKLKAPIPAELFVVIIATLASHFGK
Sbjct  749952  DIFKNIHRTNFCDVITSLLCLLVLVPAKEINECYKAKLKAPIPAELFVVIIATLASHFGK  750131

Query  334     LKEKYGSSIAGNIPTGFLPPVPPNWSLIPRIAADALPIAIIGFAITVSLSEMFAKKHGYK  393
               LKEKYGSSIAGNIPTGFLPPVPPNWSLIPRIAADALPIAIIGFAITVSLSEMFAKKHGYK
Sbjct  750132  LKEKYGSSIAGNIPTGFLPPVPPNWSLIPRIAADALPIAIIGFAITVSLSEMFAKKHGYK  750311

Query  394     VNANQEMYAIGFCNIIPSFFHCFTTSAALAKTLVKESTGCRTQVSGVVTALALLLVLLVI  453
               VNANQEMYAIGFCNIIPSFFHCFTTSAALAKTLVKESTGCRTQVSGVVTALALLLVLLVI
Sbjct  750312  VNANQEMYAIGFCNIIPSFFHCFTTSAALAKTLVKESTGCRTQVSGVVTALALLLVLLVI  750491

Query  454     APLFYSLQKCVLGVITIVNLRGALRKFGDLPKMWQVNKVDTLIWLVTMVSSAFISTELGL  513
               APLFYSLQKCVLGVITIVNLRGALRKFGDLPKMWQVNKVDTLIWLVTMVSSAFISTELGL
Sbjct  750492  APLFYSLQKCVLGVITIVNLRGALRKFGDLPKMWQVNKVDTLIWLVTMVSSAFISTELGL  750671

Query  514     VIGVSFSVFCVIVRTQRPRATLLGWVENTEVYEDLDAYKNLKILPGIKVFHFEAALYYAN  573
               VIGVSFSVFCVIVRTQRPRATLLGWVENTEVYEDLDAYKNLKILPGIKVFHFEAALYYAN
Sbjct  750672  VIGVSFSVFCVIVRTQRPRATLLGWVENTEVYEDLDAYKNLKILPGIKVFHFEAALYYAN  750851

Query  574     KDYFKSELYKQTGVNPALVMVTRKKAEKKRLQEEKQAKKENSNYIKDEVTAQLCEDQFEF  633
               KDYFKSELYKQTGVNPALVMVTRKKAEKKRLQEEKQAKKENSNYIKDEVTAQLCEDQFEF
Sbjct  750852  KDYFKSELYKQTGVNPALVMVTRKKAEKKRLQEEKQAKKENSNYIKDEVTAQLCEDQFEF  751031

Query  634     HTIIIDCSAVLFLDTVGTNTLKDLFNDYSKIGIRVLLAHCNLAVADSLRRGAYITNNDNN  693
               HTIIIDCSAVLFLDTVGTNTLKDLFNDYSKIGIRVLLAHCNLAVADSLRRGAYITNNDNN
Sbjct  751032  HTIIIDCSAVLFLDTVGTNTLKDLFNDYSKIGIRVLLAHCNLAVADSLRRGAYITNNDNN  751211

Query  694     LLFCSVHEAVHFAKNIQKQNGCCTAAESLC  723
               LLFCSVHEAVHFAKNIQKQNGCCTAAESLC
Sbjct  751212  LLFCSVHEAVHFAKNIQKQNGCCTAAESLC  751301
```

Range 2: 745262 to 745909

Score:802 bits(1638), Expect:0.0,
Method:,
Identities:216/216(100%), Positives:216/216(100%), Gaps:0/216(0%)

```
Query  1       MTCKMSAKEVHNCLSSPLGMGETGDGEGRNTTRIILEEKEKKSVDVKALIIRKLKKQCKC  60
               MTCKMSAKEVHNCLSSPLGMGETGDGEGRNTTRIILEEKEKKSVDVKALIIRKLKKQCKC
Sbjct  745262  MTCKMSAKEVHNCLSSPLGMGETGDGEGRNTTRIILEEKEKKSVDVKALIIRKLKKQCKC  745441

Query  61      TPTRVKKAFLDFFPVLQWLPRYKFKDWILGDLLSGLIVGVLLVPQSIAYSLLAGQEPVYG  120
               TPTRVKKAFLDFFPVLQWLPRYKFKDWILGDLLSGLIVGVLLVPQSIAYSLLAGQEPVYG
Sbjct  745442  TPTRVKKAFLDFFPVLQWLPRYKFKDWILGDLLSGLIVGVLLVPQSIAYSLLAGQEPVYG  745621

Query  121     LYTSFFASLIYFLMGTSRHISVGIFGVLCLMVGQVVDRELQFAGYDTKYEDHSLDYLAVV  180
               LYTSFFASLIYFLMGTSRHISVGIFGVLCLMVGQVVDRELQFAGYDTKYEDHSLDYLAVV
Sbjct  745622  LYTSFFASLIYFLMGTSRHISVGIFGVLCLMVGQVVDRELQFAGYDTKYEDHSLDYLAVV  745801

Query  181     NSSVNANNTEAFACDKGCYAITVGATLTFMAGVYQV  216
               NSSVNANNTEAFACDKGCYAITVGATLTFMAGVYQV
Sbjct  745802  NSSVNANNTEAFACDKGCYAITVGATLTFMAGVYQV  745909
```

CT Reedfish *slc26a2*

Query: sulfate transporter [Erpetoichthys calabaricus] Query ID: XP_028668740.1 Length: 713

>Erpetoichthys calabaricus chromosome 11, fErpCal1.3, whole genome shotgun sequence
Sequence ID: NC_041404.2 Length: 166547183
Range 1: 31794812 to 31795603

Score:983 bits(2007), Expect:0.0,
Method:,
Identities:264/264(100%), Positives:264/264(100%), Gaps:0/264(0%)

```
Query  450       CVLAVIIIVNLRGALRKFGELPQMWRVNRIDAIIWLVTMSTSAFLNTELGLLIGVVFSAL  509
```

```
               CVLAVIIIVNLRGALRKFGELPQMWRVNRIDAIIWLVTMSTSAFLNTELGLLIGVVFSAL
Sbjct  31795603  CVLAVIIIVNLRGALRKFGELPQMWRVNRIDAIIWLVTMSTSAFLNTELGLLIGVVFSAL  31795424

Query  510       CVLVRTQVARAVQLGRAGDLELYEDLNSYNSLHVESGIFIMRFEAPVYYANQSLFKKAVY  569
               CVLVRTQVARAVQLGRAGDLELYEDLNSYNSLHVESGIFIMRFEAPVYYANQSLFKKAVY
Sbjct  31795423  CVLVRTQVARAVQLGRAGDLELYEDLNSYNSLHVESGIFIMRFEAPVYYANQSLFKKAVY  31795244

Query  570       RCTGFEPGKETILRKKQQKAKVKKHVETNIAVENDHEIATAVQQFMPEEPHLNTLIIDCA  629
               RCTGFEPGKETILRKKQQKAKVKKHVETNIAVENDHEIATAVQQFMPEEPHLNTLIIDCA
Sbjct  31795243  RCTGFEPGKETILRKKQQKAKVKKHVETNIAVENDHEIATAVQQFMPEEPHLNTLIIDCA  31795064

Query  630       PMFFLDTTGVNALKELYVDYKNFGVQVLLAQCNSSVLDSLRRGGYLQQKGQEFTERVFFT  689
               PMFFLDTTGVNALKELYVDYKNFGVQVLLAQCNSSVLDSLRRGGYLQQKGQEFTERVFFT
Sbjct  31795063  PMFFLDTTGVNALKELYVDYKNFGVQVLLAQCNSSVLDSLRRGGYLQQKGQEFTERVFFT  31794884

Query  690       IGEAVRFAKTLHMENGVNETLSSC  713
               IGEAVRFAKTLHMENGVNETLSSC
Sbjct  31794883  IGEAVRFAKTLHMENGVNETLSSC  31794812
```

Range 2: 31796811 to 31797551

Score:909 bits(1855), Expect:0.0,
Method:,
Identities:247/247(100%), Positives:247/247(100%), Gaps:0/247(0%)

```
Query  203       ILMGLFQIGFVSVYLSDALLSGFATGASLTILTSQIKYLLGLRLPRANGIGSLIRTWIYL  262
               ILMGLFQIGFVSVYLSDALLSGFATGASLTILTSQIKYLLGLRLPRANGIGSLIRTWIYL
Sbjct  31797551  ILMGLFQIGFVSVYLSDALLSGFATGASLTILTSQIKYLLGLRLPRANGIGSLIRTWIYL  31797372

Query  263       FQNLHDTNLCDLITSLLCLLVLVPTKELNEHFKSKMKVPFPMELLVVIVATLASHFGHFK  322
               FQNLHDTNLCDLITSLLCLLVLVPTKELNEHFKSKMKVPFPMELLVVIVATLASHFGHFK
Sbjct  31797371  FQNLHDTNLCDLITSLLCLLVLVPTKELNEHFKSKMKVPFPMELLVVIVATLASHFGHFK  31797192

Query  323       DRFGSSVAETIPTGFLPPQMPNWSLIPNIALDALSIAIIGFAITVSLSEMFAKKHGYTVD  382
               DRFGSSVAETIPTGFLPPQMPNWSLIPNIALDALSIAIIGFAITVSLSEMFAKKHGYTVD
Sbjct  31797191  DRFGSSVAETIPTGFLPPQMPNWSLIPNIALDALSIAIIGFAITVSLSEMFAKKHGYTVD  31797012

Query  383       ANQEMYAIGFCNIIPAFFHCFTTSAALTKTLVKESTGCHSQLSGLVTALVLLLVLLVIAP  442
               ANQEMYAIGFCNIIPAFFHCFTTSAALTKTLVKESTGCHSQLSGLVTALVLLLVLLVIAP
Sbjct  31797011  ANQEMYAIGFCNIIPAFFHCFTTSAALTKTLVKESTGCHSQLSGLVTALVLLLVLLVIAP  31796832

Query  443       VFYSLQK  449
               VFYSLQK
Sbjct  31796831  VFYSLQK  31796811
```

Range 3: 31798205 to 31798813

Score:753 bits(1536), Expect:0.0,
Method:,
Identities:202/203(99%), Positives:203/203(100%), Gaps:0/203(0%)

```
Query  1         MMADEVNENSPDQLTSLTLPSEKKPYKPIVLEKIQKEDEDLKSAIIKKLRLFCACSPVKT  60
               MMADEVNENSPDQLTSLTLPSEKKPYKPIVLEKIQKEDEDLKSAIIKKLRLFCACSPVKT
Sbjct  31798813  MMADEVNENSPDQLTSLTLPSEKKPYKPIVLEKIQKEDEDLKSAIIKKLRLFCACSPVKT  31798634

Query  61        KKAVFDFFPILNWLPHYELKKWLLGDIMSGLIVGILLVPQSIAYSLLAGQDPIYGLYTSF  120
               KKAVFDFFPILNWLPHYELKKWLLGDIMSGLIVGILLVPQSIAYSLLAGQDPIYGLYTSF
Sbjct  31798633  KKAVFDFFPILNWLPHYELKKWLLGDIMSGLIVGILLVPQSIAYSLLAGQDPIYGLYTSF  31798454

Query  121       FSCIIYAVLGTSRHVSVGIFGVLSLLIGQVVDRELQNVDFFIQKSVNQTGISKDSNYNVT  180
               FSCIIYAVLGTSRHVSVGIFGVLSLLIGQVVDRELQNVDFFIQKSVNQTGISKDSNYNVT
Sbjct  31798453  FSCIIYAVLGTSRHVSVGIFGVLSLLIGQVVDRELQNVDFFIQKSVNQTGISKDSNYNVT  31798274

Query  181       CDRGCYAIVVGSTVTFMAGVYQI  203
               CDRGCYAIVVGSTVTFMAGVYQ+
Sbjct  31798273  CDRGCYAIVVGSTVTFMAGVYQV  31798205
```

CU Gray bichir *slc26a2*

Query: sulfate transporter [Polypterus senegalus] Query ID: XP_039630796.1 Length: 711

>Polypterus senegalus isolate Bchr_013 chromosome 13, ASM1683550v1, whole genome shotgun sequence
Sequence ID: NC_053166.1 Length: 163732318
Range 1: 34457499 to 34458290

Score:988 bits(2018), Expect:0.0,
Method:,
Identities:264/264(100%), Positives:264/264(100%), Gaps:0/264(0%)

```
Query  448       CVLAVIIIVNLRGALRKFGELPQMWRVNRIDAIIWMVTMSTSAFLNTELGLLIGVVFSAL  507
               CVLAVIIIVNLRGALRKFGELPQMWRVNRIDAIIWMVTMSTSAFLNTELGLLIGVVFSAL
Sbjct  34457499  CVLAVIIIVNLRGALRKFGELPQMWRVNRIDAIIWMVTMSTSAFLNTELGLLIGVVFSAL  34457678

Query  508       CVLVRTQMARAVQLGRAGDLELYEDLNSYNSLHVESGIFIMRFEAPVYYANQSLFKKAVY  567
```

```
              CVLVRTQMARAVQLGRAGDLELYEDLNSYNSLHVESGIFIMRFEAPVYYANQSLFKKAVY
Sbjct  34457679  CVLVRTQMARAVQLGRAGDLELYEDLNSYNSLHVESGIFIMRFEAPVYYANQSLFKKAVY  34457858

Query  568       RCTGFEPGKETILKKKQQKAKVKQHVETNIAVENDHEIATAVQHFMPEEPHLNTLIIDCA  627
              RCTGFEPGKETILKKKQQKAKVKQHVETNIAVENDHEIATAVQHFMPEEPHLNTLIIDCA
Sbjct  34457859  RCTGFEPGKETILKKKQQKAKVKQHVETNIAVENDHEIATAVQHFMPEEPHLNTLIIDCA  34458038

Query  628       PMFFIDTAGLNALKELYVDYKNFGVRVLLAQCNSSVLDSLHRGGYLKQKEQEFTERVFFT  687
              PMFFIDTAGLNALKELYVDYKNFGVRVLLAQCNSSVLDSLHRGGYLKQKEQEFTERVFFT
Sbjct  34458039  PMFFIDTAGLNALKELYVDYKNFGVRVLLAQCNSSVLDSLHRGGYLKQKEQEFTERVFFT  34458218

Query  688       ISEAVRFAKTLHMENGVNETLSSC  711
              ISEAVRFAKTLHMENGVNETLSSC
Sbjct  34458219  ISEAVRFAKTLHMENGVNETLSSC  34458290
```

Range 2: 34455535 to 34456275

Score:907 bits(1852), Expect:0.0,
Method:,
Identities:247/247(100%), Positives:247/247(100%), Gaps:0/247(0%)

```
Query  201       ILMGLFQVGFVSVYLSDALLSGFATGASLTILTSQIKYLLGLRLPRANGIGSLIRTWIYL  260
              ILMGLFQVGFVSVYLSDALLSGFATGASLTILTSQIKYLLGLRLPRANGIGSLIRTWIYL
Sbjct  34455535  ILMGLFQVGFVSVYLSDALLSGFATGASLTILTSQIKYLLGLRLPRANGIGSLIRTWIYL  34455714

Query  261       FQNLLDTNLCDLITSLLCLLVLVPTKELNEHFKSKMKVPFPMELLVVIVATLASHFGHFK  320
              FQNLLDTNLCDLITSLLCLLVLVPTKELNEHFKSKMKVPFPMELLVVIVATLASHFGHFK
Sbjct  34455715  FQNLLDTNLCDLITSLLCLLVLVPTKELNEHFKSKMKVPFPMELLVVIVATLASHFGHFK  34455894

Query  321       DRFGSSVAETIPTGFLPPQMPNWSLIPNIALDALSIAIIGFAITVSLSEMFAKKHGYTVD  380
              DRFGSSVAETIPTGFLPPQMPNWSLIPNIALDALSIAIIGFAITVSLSEMFAKKHGYTVD
Sbjct  34455895  DRFGSSVAETIPTGFLPPQMPNWSLIPNIALDALSIAIIGFAITVSLSEMFAKKHGYTVD  34456074

Query  381       ANQEMYAIGFCNIIPAFFHCFTTSAALTKTLVKESTGCHSQLSGLVTALVLLLVLLVIAP  440
              ANQEMYAIGFCNIIPAFFHCFTTSAALTKTLVKESTGCHSQLSGLVTALVLLLVLLVIAP
Sbjct  34456075  ANQEMYAIGFCNIIPAFFHCFTTSAALTKTLVKESTGCHSQLSGLVTALVLLLVLLVIAP  34456254

Query  441       VFYSLQK  447
              VFYSLQK
Sbjct  34456255  VFYSLQK  34456275
```

Range 3: 34454301 to 34454903

Score:744 bits(1519), Expect:0.0,
Method:,
Identities:200/201(99%), Positives:201/201(100%), Gaps:0/201(0%)

```
Query  1         MADEVNENSPDQLTSLTLPSEKPYKPIVLEKIQKEDEDLKSAVIKKLCLFCACNPVKTKK  60
              MADEVNENSPDQLTSLTLPSEKPYKPIVLEKIQKEDEDLKSAVIKKLCLFCACNPVKTKK
Sbjct  34454301  MADEVNENSPDQLTSLTLPSEKPYKPIVLEKIQKEDEDLKSAVIKKLCLFCACNPVKTKK  34454480

Query  61        AVFDFFPILNWLPRYELKKWLLGDLMSGLIVGILLVPQSIAYSLLAGQDPIYGLYTSFFS  120
              AVFDFFPILNWLPRYELKKWLLGDLMSGLIVGILLVPQSIAYSLLAGQDPIYGLYTSFFS
Sbjct  34454481  AVFDFFPILNWLPRYELKKWLLGDLMSGLIVGILLVPQSIAYSLLAGQDPIYGLYTSFFS  34454660

Query  121       CIIYAVLGTSRHVSVGIFGVLSLLIGQVVDRELQNVDFFIQTSVNQTGISKDSNYNVTCD  180
              CIIYAVLGTSRHVSVGIFGVLSLLIGQVVDRELQNVDFFIQTSVNQTGISKDSNYNVTCD
Sbjct  34454661  CIIYAVLGTSRHVSVGIFGVLSLLIGQVVDRELQNVDFFIQTSVNQTGISKDSNYNVTCD  34454840

Query  181       RGCYAIVVGSTVTFMAGVYQI  201
              RGCYAIVVGSTVTFMAGVYQ+
Sbjct  34454841  RGCYAIVVGSTVTFMAGVYQV  34454903
```

CV Sterlet *slc26a2*

Query: sulfate transporter-like [Acipenser ruthenus] Query ID: XP_058853166.1 Length: 732

>Acipenser ruthenus chromosome 23, fAciRut3.2 maternal haplotype, whole genome shotgun sequence
Sequence ID: NC_081211.1 Length: 30298233
Range 1: 29151547 to 29152374

Score:1006 bits(2054), Expect:0.0,
Method:,
Identities:276/276(100%), Positives:276/276(100%), Gaps:0/276(0%)

```
Query  457       CVLGVIIIVNLRGALRKFGEIPQMWRVNRVDAAIWLVTMATSALANTELGLLVGVAFSAF  516
              CVLGVIIIVNLRGALRKFGEIPQMWRVNRVDAAIWLVTMATSALANTELGLLVGVAFSAF
Sbjct  29152374  CVLGVIIIVNLRGALRKFGEIPQMWRVNRVDAAIWLVTMATSALANTELGLLVGVAFSAF  29152195

Query  517       CVLARTQRARAAKLGRAGESGPYEDLSAYRGLQAHAGFIVFRFEAPVYYANQSLFKKALY  576
              CVLARTQRARAAKLGRAGESGPYEDLSAYRGLQAHAGFIVFRFEAPVYYANQSLFKKALY
Sbjct  29152194  CVLARTQRARAAKLGRAGESGPYEDLSAYRGLQAHAGFIVFRFEAPVYYANQSLFKKALY  29152015

Query  577       RCTELDPVQEAALRRKEQQQQNKKRKKEKDKEKEEEAGLEGRGGASPDSGVATVAQLIVP  636
```

```
                 RCTELDPVQEAALRRKEQQQQNKKRKKEKDKEKEEEAGLEGRGGASPDSGVATVAQLIVP
Sbjct  29152014  RCTELDPVQEAALRRKEQQQQNKKRKKEKDKEKEEEAGLEGRGGASPDSGVATVAQLIVP  29151835

Query  637       EKRSFHTLVIDCGPVLFLDTAGVNALKEVYRDYKELGVGVLLAQCNPSVLDSLRRGGYLQ  696
                 EKRSFHTLVIDCGPVLFLDTAGVNALKEVYRDYKELGVGVLLAQCNPSVLDSLRRGGYLQ
Sbjct  29151834  EKRSFHTLVIDCGPVLFLDTAGVNALKEVYRDYKELGVGVLLAQCNPSVLDSLRRGGYLQ  29151655

Query  697       QGSDEAQLVFCTISDAVGSTQSYCTENGGCEIDSHC  732
                 QGSDEAQLVFCTISDAVGSTQSYCTENGGCEIDSHC
Sbjct  29151654  QGSDEAQLVFCTISDAVGSTQSYCTENGGCEIDSHC  29151547
```

Range 2: 29152732 to 29153472

Score:898 bits(1834), Expect:0.0,
Method:,
Identities:247/247(100%), Positives:247/247(100%), Gaps:0/247(0%)

```
Query  210       VLMGLFQVGFVSVYLSDSLLSGFATGASLTILTSQVRHLLGLKLPRVQGLGSLVKTWVSL  269
                 VLMGLFQVGFVSVYLSDSLLSGFATGASLTILTSQVRHLLGLKLPRVQGLGSLVKTWVSL
Sbjct  29153472  VLMGLFQVGFVSVYLSDSLLSGFATGASLTILTSQVRHLLGLKLPRVQGLGSLVKTWVSL  29153293

Query  270       FQNLKDTNLCDLVTSLLCLLVLVPTKELNDRYKAKLKAPIPTELLVVIVATLASHYGRFE  329
                 FQNLKDTNLCDLVTSLLCLLVLVPTKELNDRYKAKLKAPIPTELLVVIVATLASHYGRFE
Sbjct  29153292  FQNLKDTNLCDLVTSLLCLLVLVPTKELNDRYKAKLKAPIPTELLVVIVATLASHYGRFE  29153113

Query  330       ERFHSGVAGTIPTGFQPPQMPDWSLIPNVALDAFSIAIIGFAITVSLSEMFAKKHGYTVD  389
                 ERFHSGVAGTIPTGFQPPQMPDWSLIPNVALDAFSIAIIGFAITVSLSEMFAKKHGYTVD
Sbjct  29153112  ERFHSGVAGTIPTGFQPPQMPDWSLIPNVALDAFSIAIIGFAITVSLSEMFAKKHGYTVD  29152933

Query  390       ANQEMYAIGFCNILPSFFRCFTTSAALTKTLVKDSTGCHTQLSSLVTALVLLLVLLVIAP  449
                 ANQEMYAIGFCNILPSFFRCFTTSAALTKTLVKDSTGCHTQLSSLVTALVLLLVLLVIAP
Sbjct  29152932  ANQEMYAIGFCNILPSFFRCFTTSAALTKTLVKDSTGCHTQLSSLVTALVLLLVLLVIAP  29152753

Query  450       LFYSLQK  456
                 LFYSLQK
Sbjct  29152752  LFYSLQK  29152732
```

Range 3: 29153810 to 29154451

Score:772 bits(1575), Expect:0.0,
Method:,
Identities:212/214(99%), Positives:212/214(99%), Gaps:0/214(0%)

```
Query  1         MAGVETMAREENGQAGDQVTSLSRAVERKRYTPVLLEEQEKEAEDLRSVLTRKLKKKCLC  60
                 MAGVETMAREENGQAGDQVTSLSRAVERKRYTPVLLEEQEKEAEDLRSVLTRKLKKKCLC
Sbjct  29154451  MAGVETMAREENGQAGDQVTSLSRAVERKRYTPVLLEEQEKEAEDLRSVLTRKLKKKCLC  29154272

Query  61        SPSRAKELVLGFFPILSWLPQYRPKECLLGDVMSGLIVAILLVPQSIAYSLLAGQEPVYG  120
                 SPSRAKELVLGFFPILSWLPQYRPKECLLGDVMSGLIVAILLVPQSIAYSLLAGQEPVYG
Sbjct  29154271  SPSRAKELVLGFFPILSWLPQYRPKECLLGDVMSGLIVAILLVPQSIAYSLLAGQEPVYG  29154092

Query  121       LYTSFFSCIIYAVLASSRHVSVGIFGVLCLLVGQVVDRELQAAGYTSEGGRNQTGMTGAG  180
                 LYTSFFSCIIYAVLASSRHVSVGIFGVLCLLVGQVVDRELQAAGYTSEGGRNQTGMTGAG
Sbjct  29154091  LYTSFFSCIIYAVLASSRHVSVGIFGVLCLLVGQVVDRELQAAGYTSEGGRNQTGMTGAG  29153912

Query  181       NLTAGMTCDQSCYAISVATMLTFMAGLYQVLMGL  214
                 NLTAGMTCDQSCYAISVATMLTFMAGLYQV  GL
Sbjct  29153911  NLTAGMTCDQSCYAISVATMLTFMAGLYQVSVGL  29153810
```

CW Spotted gar *slc26a2*

Query: PREDICTED: sulfate transporter-like isoform X1 [Lepisosteus oculatus] Query ID: XP_006632145.2 Length: 710

>Lepisosteus oculatus isolate Spotted Gar 1 linkage group LG6 genomic scaffold, LepOcu1, whole genome shotgun sequence
Sequence ID: NW_006269931.1 Length: 10849474
Range 1: 7940315 to 7941094

Score:944 bits(1928), Expect:0.0,
Method:,
Identities:260/260(100%), Positives:260/260(100%), Gaps:0/260(0%)

```
Query  451      CVLAVIIVVNLRGALRKFLDVPRMWRVNHVDAAIWLVTMGTSALLNTELGLLVGVLFSAL  510
                CVLAVIIVVNLRGALRKFLDVPRMWRVNHVDAAIWLVTMGTSALLNTELGLLVGVLFSAL
Sbjct  7941094  CVLAVIIVVNLRGALRKFLDVPRMWRVNHVDAAIWLVTMGTSALLNTELGLLVGVLFSAL  7940915

Query  511      CVLARTQGAPAAELGRAGELELYEDLGAYKGLRTQPGVSVFRFGAPIYYANQALFKRALY  570
                CVLARTQGAPAAELGRAGELELYEDLGAYKGLRTQPGVSVFRFGAPIYYANQALFKRALY
Sbjct  7940914  CVLARTQGAPAAELGRAGELELYEDLGAYKGLRTQPGVSVFRFGAPIYYANQALFKRALY  7940735

Query  571      RHLGLDPVKEKSRRRKLERRRRKEEEGGAAGDQNKAEESPARIFIPQRPGCHTLVLDCAP  630
                RHLGLDPVKEKSRRRKLERRRRKEEEGGAAGDQNKAEESPARIFIPQRPGCHTLVLDCAP
Sbjct  7940734  RHLGLDPVKEKSRRRKLERRRRKEEEGGAAGDQNKAEESPARIFIPQRPGCHTLVLDCAP  7940555

Query  631      VLFLDTAGVNALQEVRKDYSELGLRVLLARCNASVLDSLRRGGYLEEAGSSAGKVFFTIG  690
```

```
              VLFLDTAGVNALQEVRKDYSELGLRVLLARCNASVLDSLRRGGYLEEAGSSAGKVFFTIG
Sbjct  7940554  VLFLDTAGVNALQEVRKDYSELGLRVLLARCNASVLDSLRRGGYLEEAGSSAGKVFFTIG  7940375

Query  691      DAVGYALALCSQNGNGETCC  710
                DAVGYALALCSQNGNGETCC
Sbjct  7940374  DAVGYALALCSQNGNGETCC  7940315
```

Range 2: 7941864 to 7942607

Score:906 bits(1850), Expect:0.0,
Method:,
Identities:248/248(100%), Positives:248/248(100%), Gaps:0/248(0%)

```
Query  203      QVLMGVFQVGFVSVYLSDSLLSGFATGASLTILTSQVKYLLGIRLPRTQGLGSLVKTWFY  262
                QVLMGVFQVGFVSVYLSDSLLSGFATGASLTILTSQVKYLLGIRLPRTQGLGSLVKTWFY
Sbjct  7942607  QVLMGVFQVGFVSVYLSDSLLSGFATGASLTILTSQVKYLLGIRLPRTQGLGSLVKTWFY  7942428

Query  263      LFQNIKDTNVCDLVTSLLCLLVLVPTKELNDRFKAKLKAPIPMELFVVIVATLASHYGRF  322
                LFQNIKDTNVCDLVTSLLCLLVLVPTKELNDRFKAKLKAPIPMELFVVIVATLASHYGRF
Sbjct  7942427  LFQNIKDTNVCDLVTSLLCLLVLVPTKELNDRFKAKLKAPIPMELFVVIVATLASHYGRF  7942248

Query  323      HEVYGSDISGTIPTGFQPPMLPAWSLIPNIALDALSIAIIGFAITVSLSEMFAKKHGYVV  382
                HEVYGSDISGTIPTGFQPPMLPAWSLIPNIALDALSIAIIGFAITVSLSEMFAKKHGYVV
Sbjct  7942247  HEVYGSDISGTIPTGFQPPMLPAWSLIPNIALDALSIAIIGFAITVSLSEMFAKKHGYVV  7942068

Query  383      DANQEMYAIGFCNILPSFFRCFTTSAALTKTLVKESTGCQTQLSGVVTALVLLLVLLVVA  442
                DANQEMYAIGFCNILPSFFRCFTTSAALTKTLVKESTGCQTQLSGVVTALVLLLVLLVVA
Sbjct  7942067  DANQEMYAIGFCNILPSFFRCFTTSAALTKTLVKESTGCQTQLSGVVTALVLLLVLLVVA  7941888

Query  443      PLFFSLQK  450
                PLFFSLQK
Sbjct  7941887  PLFFSLQK  7941864
```

Range 3: 7942912 to 7943523

Score:751 bits(1532), Expect:0.0,
Method:,
Identities:204/204(100%), Positives:204/204(100%), Gaps:0/204(0%)

```
Query  1        MDKVEAETMAGEDQGDSSGDQAGRKRFNPVVLEEQEKETRSLKSLFARKVKKKCSCGPAR  60
                MDKVEAETMAGEDQGDSSGDQAGRKRFNPVVLEEQEKETRSLKSLFARKVKKKCSCGPAR
Sbjct  7943523  MDKVEAETMAGEDQGDSSGDQAGRKRFNPVVLEEQEKETRSLKSLFARKVKKKCSCGPAR  7943344

Query  61       IKGLLFGFFPILSWLPGYKLKEWIIGDMMSGLIVGILLVPQSIAYSLLAGQDPIYGLYTS  120
                IKGLLFGFFPILSWLPGYKLKEWIIGDMMSGLIVGILLVPQSIAYSLLAGQDPIYGLYTS
Sbjct  7943343  IKGLLFGFFPILSWLPGYKLKEWIIGDMMSGLIVGILLVPQSIAYSLLAGQDPIYGLYTS  7943164

Query  121      FFSCIIYALLGTSRHISVGIFGVLCLLVGQVVDKELMAAGYLTEGNQTAIPGPGNATAAL  180
                FFSCIIYALLGTSRHISVGIFGVLCLLVGQVVDKELMAAGYLTEGNQTAIPGPGNATAAL
Sbjct  7943163  FFSCIIYALLGTSRHISVGIFGVLCLLVGQVVDKELMAAGYLTEGNQTAIPGPGNATAAL  7942984

Query  181      DCDRSCYAILVGTTVTFMAGVYQV  204
                DCDRSCYAILVGTTVTFMAGVYQV
Sbjct  7942983  DCDRSCYAILVGTTVTFMAGVYQV  7942912
```

CX Asian bonytongue *slc26a2*

Query: sulfate transporter isoform X2 [Scleropages formosus] Query ID: XP_018588710.2 Length: 694

>Scleropages formosus chromosome 13, fSclFor1.1, whole genome shotgun sequence
Sequence ID: NC_041818.1 Length: 29668380
Range 1: 27296087 to 27296857

Score:947 bits(1934), Expect:0.0,
Method:,
Identities:257/257(100%), Positives:257/257(100%), Gaps:0/257(0%)

```
Query  438       CVLAVIIVVNLRGALTKICEVPQMWRVNHVDTATWLITMGTSALVNTELGLVVGVLVSAF  497
                 CVLAVIIVVNLRGALTKICEVPQMWRVNHVDTATWLITMGTSALVNTELGLVVGVLVSAF
Sbjct  27296857  CVLAVIIVVNLRGALTKICEVPQMWRVNHVDTATWLITMGTSALVNTELGLVVGVLVSAF  27296678

Query  498       CVLGRTQRAPAVQLGQADLPELYEDLASYKGLHTHRGVVVFRYEAPIYYANQVLFKRMLY  557
                 CVLGRTQRAPAVQLGQADLPELYEDLASYKGLHTHRGVVVFRYEAPIYYANQVLFKRMLY
Sbjct  27296677  CVLGRTQRAPAVQLGQADLPELYEDLASYKGLHTHRGVVVFRYEAPIYYANQVLFKRMLY  27296498

Query  558       RRVGLDPLVEKARRMKQEKQDRKRDMLKPSAGKKEVPTCVFLPQHTSFHTLVLDCAPVLF  617
                 RRVGLDPLVEKARRMKQEKQDRKRDMLKPSAGKKEVPTCVFLPQHTSFHTLVLDCAPVLF
Sbjct  27296497  RRVGLDPLVEKARRMKQEKQDRKRDMLKPSAGKKEVPTCVFLPQHTSFHTLVLDCAPVLF  27296318

Query  618       LDTAGVNALKEVAKDYNELGIRLVLARCSASVLDSLRRGGYLDAEAEGPPKVFFTIGDAV  677
                 LDTAGVNALKEVAKDYNELGIRLVLARCSASVLDSLRRGGYLDAEAEGPPKVFFTIGDAV
Sbjct  27296317  LDTAGVNALKEVAKDYNELGIRLVLARCSASVLDSLRRGGYLDAEAEGPPKVFFTIGDAV  27296138

Query  678       QYAQSLASQNGNCEASC  694
```

```
              QYAQSLASQNGNCEASC
Sbjct  27296137  QYAQSLASQNGNCEASC  27296087
```

Range 2: 27297072 to 27297821

Score:908 bits(1854), Expect:0.0,
Method:,
Identities:249/250(99%), Positives:250/250(100%), Gaps:0/250(0%)

```
Query  188       IFQVLMGLLQVGFVSVFLSDALLSGFATGASLTILTSQVKYLLGLNLPRAQSPGSLIKTW  247
                 +FQVLMGLLQVGFVSVFLSDALLSGFATGASLTILTSQVKYLLGLNLPRAQSPGSLIKTW
Sbjct  27297821  VFQVLMGLLQVGFVSVFLSDALLSGFATGASLTILTSQVKYLLGLNLPRAQSPGSLIKTW  27297642

Query  248       VLLAQNIGQTNICDLVTSLLCMLVLVPAKELNDRFKAKLRAPIPFELFVVIAATLASHFG  307
                 VLLAQNIGQTNICDLVTSLLCMLVLVPAKELNDRFKAKLRAPIPFELFVVIAATLASHFG
Sbjct  27297641  VLLAQNIGQTNICDLVTSLLCMLVLVPAKELNDRFKAKLRAPIPFELFVVIAATLASHFG  27297462

Query  308       HFEERFGSSVAGNIPTGFMPPQLPAWSLIPNVAMDALSMAIIGFVITVSLSEMFAKKHGY  367
                 HFEERFGSSVAGNIPTGFMPPQLPAWSLIPNVAMDALSMAIIGFVITVSLSEMFAKKHGY
Sbjct  27297461  HFEERFGSSVAGNIPTGFMPPQLPAWSLIPNVAMDALSMAIIGFVITVSLSEMFAKKHGY  27297282

Query  368       TVVANQEMYAIGFCNILSSFFRCFTTSAALTKTLVKESTGCQTQLSSLVTALVLLLVLLV  427
                 TVVANQEMYAIGFCNILSSFFRCFTTSAALTKTLVKESTGCQTQLSSLVTALVLLLVLLV
Sbjct  27297281  TVVANQEMYAIGFCNILSSFFRCFTTSAALTKTLVKESTGCQTQLSSLVTALVLLLVLLV  27297102

Query  428       IAPLFYSLQK  437
                 IAPLFYSLQK
Sbjct  27297101  IAPLFYSLQK  27297072
```

Range 3: 27298027 to 27298599

Score:708 bits(1445), Expect:0.0,
Method:,
Identities:191/191(100%), Positives:191/191(100%), Gaps:0/191(0%)

```
Query  1         MADEDQDGVAEDDLTTVEPHVPFVLEAWEKEEESWRMLVSRKLRKQCACSSARTKGLLLG  60
                 MADEDQDGVAEDDLTTVEPHVPFVLEAWEKEEESWRMLVSRKLRKQCACSSARTKGLLLG
Sbjct  27298599  MADEDQDGVAEDDLTTVEPHVPFVLEAWEKEEESWRMLVSRKLRKQCACSSARTKGLLLG  27298420

Query  61        FFPVLSWLPRYRFREWLLGDVMSGVIVGILLVPQSIAYSLLAGQDPIYGLYTSFFAGIIY  120
                 FFPVLSWLPRYRFREWLLGDVMSGVIVGILLVPQSIAYSLLAGQDPIYGLYTSFFAGIIY
Sbjct  27298419  FFPVLSWLPRYRFREWLLGDVMSGVIVGILLVPQSIAYSLLAGQDPIYGLYTSFFAGIIY  27298240

Query  121       MVLGTSRHVSVGIFGVLCLLVGQVVDRELDAAGFNVSAPSGNLTAGLACDRSCYSIIVGA  180
                 MVLGTSRHVSVGIFGVLCLLVGQVVDRELDAAGFNVSAPSGNLTAGLACDRSCYSIIVGA
Sbjct  27298239  MVLGTSRHVSVGIFGVLCLLVGQVVDRELDAAGFNVSAPSGNLTAGLACDRSCYSIIVGA  27298060

Query  181       TVTFMAGIFQV  191
                 TVTFMAGIFQV
Sbjct  27298059  TVTFMAGIFQV  27298027
```

CY Zebrafish *slc26a2*

Query: sulfate transporter [Danio rerio] Query ID: XP_685114.1 Length: 699

>Danio rerio strain Tuebingen chromosome 14, GRCz11 Primary Assembly
Sequence ID: NC_007125.7 Length: 52660232
Range 1: 25440590 to 25441360

Score:946 bits(1931), Expect:0.0,
Method:,
Identities:257/257(100%), Positives:257/257(100%), Gaps:0/257(0%)

```
Query  443       CVLAVIIVVNLRGALRKFGDIPQMWRVNRVDTVIWLLTMATSALVNTELGLLVGVVVSAF  502
                 CVLAVIIVVNLRGALRKFGDIPQMWRVNRVDTVIWLLTMATSALVNTELGLLVGVVVSAF
Sbjct  25441360  CVLAVIIVVNLRGALRKFGDIPQMWRVNRVDTVIWLLTMATSALVNTELGLLVGVVVSAF  25441181

Query  503       CVLGRTQFAQALQLGQAGDRELFEDIASYNGLQTQPDVAVFRYEAPIYYANQSLFKKSLY  562
                 CVLGRTQFAQALQLGQAGDRELFEDIASYNGLQTQPDVAVFRYEAPIYYANQSLFKKSLY
Sbjct  25441180  CVLGRTQFAQALQLGQAGDRELFEDIASYNGLQTQPDVAVFRYEAPIYYANQSLFKKSLY  25441001

Query  563       RNVGLDPLKEKAKQRKLEKQRKQKHSREDQKQEMDASTNVNLLQHSSFHTLVIDCSSVLF  622
                 RNVGLDPLKEKAKQRKLEKQRKQKHSREDQKQEMDASTNVNLLQHSSFHTLVIDCSSVLF
Sbjct  25441000  RNVGLDPLKEKAKQRKLEKQRKQKHSREDQKQEMDASTNVNLLQHSSFHTLVIDCSSVLF  25440821

Query  623       LDTAGVNALKEVFKDYKELGVNVLLAQCNTSVIDSLRRGDYYDPNKGTKDIQFHTIGEAI  682
                 LDTAGVNALKEVFKDYKELGVNVLLAQCNTSVIDSLRRGDYYDPNKGTKDIQFHTIGEAI
Sbjct  25440820  LDTAGVNALKEVFKDYKELGVNVLLAQCNTSVIDSLRRGDYYDPNKGTKDIQFHTIGEAI  25440641

Query  683       LYGQSLKSQNGNCDTSV  699
                 LYGQSLKSQNGNCDTSV
Sbjct  25440640  LYGQSLKSQNGNCDTSV  25440590
```

Range 2: 25443329 to 25444078

Score:903 bits(1843), Expect:0.0,
Method:,
Identities:249/250(99%), Positives:249/250(99%), Gaps:0/250(0%)

```
Query  195       QVLMGLLQIGFVSVFLSDSLLSGFATGASLTILTSQIKYFLGLHLPRVQGWGSLIKTWIS  254
                 QVLMGLLQIGFVSVFLSDSLLSGFATGASLTILTSQIKYFLGLHLPRVQGWGSLIKTWIS
Sbjct  25444078  QVLMGLLQIGFVSVFLSDSLLSGFATGASLTILTSQIKYFLGLHLPRVQGWGSLIKTWIS  25443899

Query  255       LFKNLGHTNLCDLITSLICLLVLVPAKELNDRFKAKLKAPIPFELFVVIAATLASHFGHF  314
                 LFKNLGHTNLCDLITSLICLLVLVPAKELNDRFKAKLKAPIPFELFVVIAATLASHFGHF
Sbjct  25443898  LFKNLGHTNLCDLITSLICLLVLVPAKELNDRFKAKLKAPIPFELFVVIAATLASHFGHF  25443719

Query  315       KDTYGSDVAGTIPTGFMAPQSPNLSLVPNIAIDALSIAIVGFAITVSLSEMFAKKHGYIV  374
                 KDTYGSDVAGTIPTGFMAPQSPNLSLVPNIAIDALSIAIVGFAITVSLSEMFAKKHGYIV
Sbjct  25443718  KDTYGSDVAGTIPTGFMAPQSPNLSLVPNIAIDALSIAIVGFAITVSLSEMFAKKHGYIV  25443539

Query  375       NANQELNAIGNCNIISSYFHCFTVSAALTKTLVKESTGCHTQISGLVTALVLLLVMLVIA  434
                 NANQELNAIGNCNIISSYFHCFTVSAALTKTLVKESTGCHTQISGLVTALVLLLVMLVIA
Sbjct  25443538  NANQELNAIGNCNIISSYFHCFTVSAALTKTLVKESTGCHTQISGLVTALVLLLVMLVIA  25443359

Query  435       PAFYSLQKCV  444
                 PAFYSLQK V
Sbjct  25443358  PAFYSLQKYV  25443329
```

Range 3: 25444169 to 25444756

Score:729 bits(1488), Expect:0.0,
Method:,
Identities:196/196(100%), Positives:196/196(100%), Gaps:0/196(0%)

```
Query  1         MPTEGSCNNLSAEEDAKREPNLPFRLEECVKEKVSWKEALRKKVKKCSCSTARVKSQIMK  60
                 MPTEGSCNNLSAEEDAKREPNLPFRLEECVKEKVSWKEALRKKVKKCSCSTARVKSQIMK
Sbjct  25444756  MPTEGSCNNLSAEEDAKREPNLPFRLEECVKEKVSWKEALRKKVKKCSCSTARVKSQIMK  25444577

Query  61        SFPIVKWLPRYRFKDWIIGDAMSGLIVGILLVPQSIAYSLLAGQDPIYGLYTSFFANIIY  120
                 SFPIVKWLPRYRFKDWIIGDAMSGLIVGILLVPQSIAYSLLAGQDPIYGLYTSFFANIIY
Sbjct  25444576  SFPIVKWLPRYRFKDWIIGDAMSGLIVGILLVPQSIAYSLLAGQDPIYGLYTSFFANIIY  25444397

Query  121       ALLGTSRHISVGMFGVLCLLVGQVVDRELTLAGYSSDINQTTLGNVDNSTGPICDRSCYA  180
                 ALLGTSRHISVGMFGVLCLLVGQVVDRELTLAGYSSDINQTTLGNVDNSTGPICDRSCYA
Sbjct  25444396  ALLGTSRHISVGMFGVLCLLVGQVVDRELTLAGYSSDINQTTLGNVDNSTGPICDRSCYA  25444217

Query  181       IMVGATLTFTAGIYQV  196
                 IMVGATLTFTAGIYQV
Sbjct  25444216  IMVGATLTFTAGIYQV  25444169
```

CZ Northern pike *slc26a2*

Query: sulfate transporter [Esox lucius] Query ID: XP_010897256.2 Length: 724

>Esox lucius isolate fEsoLuc1 chromosome 4, fEsoLuc1.pri, whole genome shotgun sequence
Sequence ID: NC_047572.1 Length: 34969986
Range 1: 21290233 to 21291036

Score:992 bits(2025), Expect:0.0,
Method:,
Identities:268/268(100%), Positives:268/268(100%), Gaps:0/268(0%)

```
Query  457       CVLAVIIVVNLRGALRKFTDFPRMWRINHVDAVIWLVTMATSAFVNTELGLLVGVMVSAF  516
                 CVLAVIIVVNLRGALRKFTDFPRMWRINHVDAVIWLVTMATSAFVNTELGLLVGVMVSAF
Sbjct  21290233  CVLAVIIVVNLRGALRKFTDFPRMWRINHVDAVIWLVTMATSAFVNTELGLLVGVMVSAF  21290412

Query  517       CVLGRTQRAQASELGKAGDHELYQDLASYKGLQTQPGVAIFRYEAPIYYANQSLFKKALY  576
                 CVLGRTQRAQASELGKAGDHELYQDLASYKGLQTQPGVAIFRYEAPIYYANQSLFKKALY
Sbjct  21290413  CVLGRTQRAQASELGKAGDHELYQDLASYKGLQTQPGVAIFRYEAPIYYANQSLFKKALY  21290592

Query  577       RCLGLDPVKEKAQRRKKEKLGRKQEAAADMKIVGVEILGKDTELATKTKVFLPNQVNFHS  636
                 RCLGLDPVKEKAQRRKKEKLGRKQEAAADMKIVGVEILGKDTELATKTKVFLPNQVNFHS
Sbjct  21290593  RCLGLDPVKEKAQRRKKEKLGRKQEAAADMKIVGVEILGKDTELATKTKVFLPNQVNFHS  21290772

Query  637       VVIDCSPVLFLDTAGVNALKEVCKDYKQLGVQVFLAQCNTSVLESLDRGDYYEEKSKGEK  696
                 VVIDCSPVLFLDTAGVNALKEVCKDYKQLGVQVFLAQCNTSVLESLDRGDYYEEKSKGEK
Sbjct  21290773  VVIDCSPVLFLDTAGVNALKEVCKDYKQLGVQVFLAQCNTSVLESLDRGDYYEEKSKGEK  21290952

Query  697       EKVFFTISDAVHYTQSLCPQNGDCQTFC  724
                 EKVFFTISDAVHYTQSLCPQNGDCQTFC
Sbjct  21290953  EKVFFTISDAVHYTQSLCPQNGDCQTFC  21291036
```

Range 2: 21289303 to 21290043

Score:896 bits(1830), Expect:0.0,
Method:,
Identities:247/247(100%), Positives:247/247(100%), Gaps:0/247(0%)

```
Query  210       VLMGLLQVGFVSVYLSDSLLSGFATGASLTILTSQVKYLLGLKLPRIQGWGSLIKTWVSV  269
                 VLMGLLQVGFVSVYLSDSLLSGFATGASLTILTSQVKYLLGLKLPRIQGWGSLIKTWVSV
Sbjct  21289303  VLMGLLQVGFVSVYLSDSLLSGFATGASLTILTSQVKYLLGLKLPRIQGWGSLIKTWVSV  21289482

Query  270       FQNLGQTNICDLLTSLVCLAVLVPTKEFSDRFKAKLKAPIPFELFVVIAATLASHFGHFK  329
                 FQNLGQTNICDLLTSLVCLAVLVPTKEFSDRFKAKLKAPIPFELFVVIAATLASHFGHFK
Sbjct  21289483  FQNLGQTNICDLLTSLVCLAVLVPTKEFSDRFKAKLKAPIPFELFVVIAATLASHFGHFK  21289662

Query  330       EEYGSDVAGAIPTGFQSPQLPSWSLIPNVAVDAFTIAIVGFAITVSLSEMFAKKHGYTVD  389
                 EEYGSDVAGAIPTGFQSPQLPSWSLIPNVAVDAFTIAIVGFAITVSLSEMFAKKHGYTVD
Sbjct  21289663  EEYGSDVAGAIPTGFQSPQLPSWSLIPNVAVDAFTIAIVGFAITVSLSEMFAKKHGYTVD  21289842

Query  390       ANQEMYAIGFCNILPSFFRCFTTSAALTKTLVKESTGCQTQLSGLVTALVLLLVLLVIAP  449
                 ANQEMYAIGFCNILPSFFRCFTTSAALTKTLVKESTGCQTQLSGLVTALVLLLVLLVIAP
Sbjct  21289843  ANQEMYAIGFCNILPSFFRCFTTSAALTKTLVKESTGCQTQLSGLVTALVLLLVLLVIAP  21290022

Query  450       LFYSLQK  456
                 LFYSLQK
Sbjct  21290023  LFYSLQK  21290043
```

Range 3: 21288601 to 21289206

Score:749 bits(1529), Expect:0.0,
Method:,
Identities:202/202(100%), Positives:202/202(100%), Gaps:0/202(0%)

```
Query  9         ATRDEEDDCVSADQGAQSALHTPVMLARREREKENWRTGLSHQLKKQCKCSSERAKAKLL  68
                 ATRDEEDDCVSADQGAQSALHTPVMLARREREKENWRTGLSHQLKKQCKCSSERAKAKLL
Sbjct  21288601  ATRDEEDDCVSADQGAQSALHTPVMLARREREKENWRTGLSHQLKKQCKCSSERAKAKLL  21288780

Query  69        GFVPIIKWLPHYQFKEWILGDVMSGLIVGILLVPQSIAYSLLAGQDPIYGLYTSFFSSII  128
                 GFVPIIKWLPHYQFKEWILGDVMSGLIVGILLVPQSIAYSLLAGQDPIYGLYTSFFSSII
Sbjct  21288781  GFVPIIKWLPHYQFKEWILGDVMSGLIVGILLVPQSIAYSLLAGQDPIYGLYTSFFSSII  21288960

Query  129       YTLLGTSRHISVGIFGVLCLLVGQVVDRELVLAGYLTETNRNNATMGLDNWNASATESVC  188
                 YTLLGTSRHISVGIFGVLCLLVGQVVDRELVLAGYLTETNRNNATMGLDNWNASATESVC
Sbjct  21288961  YTLLGTSRHISVGIFGVLCLLVGQVVDRELVLAGYLTETNRNNATMGLDNWNASATESVC  21289140

Query  189       DRSCYAIMVGATLTFTAGVYQV  210
                 DRSCYAIMVGATLTFTAGVYQV
Sbjct  21289141  DRSCYAIMVGATLTFTAGVYQV  21289206
```

DA Atlantic cod *slc26a2*

Query: sulfate transporter isoform X1 [Gadus morhua] Query ID: XP_030224398.1 Length: 746

>Gadus morhua chromosome 10, gadMor3.0, whole genome shotgun sequence
Sequence ID: NC_044057.1 Length: 27234273
Range 1: 24021035 to 24021928

Score:1068 bits(2181), Expect:0.0,
Method:,
Identities:298/298(100%), Positives:298/298(100%), Gaps:0/298(0%)

```
Query  449       CVLAVIIVVNLRGALRKFADVPRMWLANRIDACVWLITMATSALVNTELGLVVGVLVSAF  508
                 CVLAVIIVVNLRGALRKFADVPRMWLANRIDACVWLITMATSALVNTELGLVVGVLVSAF
Sbjct  24021035  CVLAVIIVVNLRGALRKFADVPRMWLANRIDACVWLITMATSALVNTELGLVVGVLVSAF  24021214

Query  509       CVLGRTQRAQAVALGRSSSGRELYEDLQAYEGLRAAPGGVAVFRYQAPVYYANQSLFKSS  568
                 CVLGRTQRAQAVALGRSSSGRELYEDLQAYEGLRAAPGGVAVFRYQAPVYYANQSLFKSS
Sbjct  24021215  CVLGRTQRAQAVALGRSSSGRELYEDLQAYEGLRAAPGGVAVFRYQAPVYYANQSLFKSS  24021394

Query  569       LFRAAGLDPVKEKARRRKLEKKKKDRSDPGKEEEAAATTADVAALGRSDDAENGAVGKQG  628
                 LFRAAGLDPVKEKARRRKLEKKKKDRSDPGKEEEAAATTADVAALGRSDDAENGAVGKQG
Sbjct  24021395  LFRAAGLDPVKEKARRRKLEKKKKDRSDPGKEEEAAATTADVAALGRSDDAENGAVGKQG  24021574

Query  629       AGESDAGVTKVLMPAGKRKEETAPGGGVHSVVLDCSAVLFLDTAGVNALKEVRKDYQDVG  688
                 AGESDAGVTKVLMPAGKRKEETAPGGGVHSVVLDCSAVLFLDTAGVNALKEVRKDYQDVG
Sbjct  24021575  AGESDAGVTKVLMPAGKRKEETAPGGGVHSVVLDCSAVLFLDTAGVNALKEVRKDYQDVG  24021754

Query  689       VRLLLARCSASVLDTLERGGYSPLNADREEPVFFSIGDAVRYAQNVSNGGEDKCDTYC  746
                 VRLLLARCSASVLDTLERGGYSPLNADREEPVFFSIGDAVRYAQNVSNGGEDKCDTYC
Sbjct  24021755  VRLLLARCSASVLDTLERGGYSPLNADREEPVFFSIGDAVRYAQNVSNGGEDKCDTYC  24021928
```

Range 2: 24016941 to 24017543

Score:736 bits(1502), Expect:0.0,
Method:,
Identities:201/201(100%), Positives:201/201(100%), Gaps:0/201(0%)

```
Query  1         MTLDEEPSDTEDPHHPLVLHRIKKDEEVSWRTSASRQIRGHCSCTKDKARAKLLGFFPIL  60
                 MTLDEEPSDTEDPHHPLVLHRIKKDEEVSWRTSASRQIRGHCSCTKDKARAKLLGFFPIL
Sbjct  24016941  MTLDEEPSDTEDPHHPLVLHRIKKDEEVSWRTSASRQIRGHCSCTKDKARAKLLGFFPIL  24017120

Query  61        KWLPRYQLRDWILGDVMSGVIVGILLVPQSIAYSLLAGQEPIYGLYTSFFSAIIYAFLGT  120
                 KWLPRYQLRDWILGDVMSGVIVGILLVPQSIAYSLLAGQEPIYGLYTSFFSAIIYAFLGT
Sbjct  24017121  KWLPRYQLRDWILGDVMSGVIVGILLVPQSIAYSLLAGQEPIYGLYTSFFSAIIYAFLGT  24017300

Query  121       SRHISVGIFGVLCLLVGQVVERELAAAGYPSDTVLVAGGGYNTSAALLVAQGNSSLGRAC  180
                 SRHISVGIFGVLCLLVGQVVERELAAAGYPSDTVLVAGGGYNTSAALLVAQGNSSLGRAC
Sbjct  24017301  SRHISVGIFGVLCLLVGQVVERELAAAGYPSDTVLVAGGGYNTSAALLVAQGNSSLGRAC  24017480

Query  181       DKSCYAIVVGATVTFTAGVYQ  201
                 DKSCYAIVVGATVTFTAGVYQ
Sbjct  24017481  DKSCYAIVVGATVTFTAGVYQ  24017543
```

Range 3: 24018840 to 24019289

Score:545 bits(1112), Expect:5e-159,
Method:,
Identities:150/150(100%), Positives:150/150(100%), Gaps:0/150(0%)

```
Query  299       APIPFELFVVIIATLASHVGRFNAEYGSGVAGAIPTGFLPPQLPDWSLIPSVAVDAFSIA  358
                 APIPFELFVVIIATLASHVGRFNAEYGSGVAGAIPTGFLPPQLPDWSLIPSVAVDAFSIA
Sbjct  24018840  APIPFELFVVIIATLASHVGRFNAEYGSGVAGAIPTGFLPPQLPDWSLIPSVAVDAFSIA  24019019

Query  359       VVGFAITVSLSEMFAKKHGYAVDANQEMYAIGFCNVLPSFFRCFTTSAALTKTLVKESTG  418
                 VVGFAITVSLSEMFAKKHGYAVDANQEMYAIGFCNVLPSFFRCFTTSAALTKTLVKESTG
Sbjct  24019020  VVGFAITVSLSEMFAKKHGYAVDANQEMYAIGFCNVLPSFFRCFTTSAALTKTLVKESTG  24019199

Query  419       CQSQVSGLVTALVLLLVLLFIAPFFYSLQK  448
                 CQSQVSGLVTALVLLLVLLFIAPFFYSLQK
Sbjct  24019200  CQSQVSGLVTALVLLLVLLFIAPFFYSLQK  24019289
```

Range 4: 24018048 to 24018338

Score:353 bits(719), Expect:6e-101,
Method:,
Identities:97/97(100%), Positives:97/97(100%), Gaps:0/97(0%)

```
Query  202       LLMGLLQVGFVSVYLSDSLLSGFATGASLTILTSQLKYLLGLRIPRPQGWFTLIKTWYAL  261
                 LLMGLLQVGFVSVYLSDSLLSGFATGASLTILTSQLKYLLGLRIPRPQGWFTLIKTWYAL
Sbjct  24018048  LLMGLLQVGFVSVYLSDSLLSGFATGASLTILTSQLKYLLGLRIPRPQGWFTLIKTWYAL  24018227

Query  262       LTNLANTNVCDLVTSLVCLLVLVPSKELNDRFKAKLK  298
                 LTNLANTNVCDLVTSLVCLLVLVPSKELNDRFKAKLK
Sbjct  24018228  LTNLANTNVCDLVTSLVCLLVLVPSKELNDRFKAKLK  24018338
```

DB Nile tilapia *slc26a2*

Query: sulfate transporter [Oreochromis niloticus] Query ID: XP_019211041.1 Length: 716

>Oreochromis niloticus isolate F11D_XX linkage group LG2, O_niloticus_UMD_NMBU, whole genome shotgun sequence
Sequence ID: NC_031966.2 Length: 36523203
Range 1: 3090103 to 3090915

Score:989 bits(2019), Expect:0.0,
Method:,
Identities:269/271(99%), Positives:269/271(99%), Gaps:0/271(0%)

```
Query  446      LQKCVLAAIIVVNLRGALRKFTDIPCMWRANHIDAFIWLITMATSALVNTELGLLVGVLV  505
                L  CVLAAIIVVNLRGALRKFTDIPCMWRANHIDAFIWLITMATSALVNTELGLLVGVLV
Sbjct  3090103  LLRCVLAAIIVVNLRGALRKFTDIPCMWRANHIDAFIWLITMATSALVNTELGLLVGVLV  3090282

Query  506      SAFCVLGRTQRVQVLELGRAMTREHYEDLASYKGLQTHPDVAIFRYEAPIYYANQNLFKK  565
                SAFCVLGRTQRVQVLELGRAMTREHYEDLASYKGLQTHPDVAIFRYEAPIYYANQNLFKK
Sbjct  3090283  SAFCVLGRTQRVQVLELGRAMTREHYEDLASYKGLQTHPDVAIFRYEAPIYYANQNLFKK  3090462

Query  566      SLYRCVGLDPVKEKSRRVKFQNSKRQEEAAGVPTSNSDEKEAEAPPAVTLMMNTASRQLR  625
                SLYRCVGLDPVKEKSRRVKFQNSKRQEEAAGVPTSNSDEKEAEAPPAVTLMMNTASRQLR
Sbjct  3090463  SLYRCVGLDPVKEKSRRVKFQNSKRQEEAAGVPTSNSDEKEAEAPPAVTLMMNTASRQLR  3090642

Query  626      RVVIDCSAVLFLDTAGVNALKEVRKDYGELGVVVELAQCNTSVLDTLERGGYYSNQKGGN  685
                RVVIDCSAVLFLDTAGVNALKEVRKDYGELGVVVELAQCNTSVLDTLERGGYYSNQKGGN
Sbjct  3090643  RVVIDCSAVLFLDTAGVNALKEVRKDYGELGVVVELAQCNTSVLDTLERGGYYSNQKGGN  3090822

Query  686      AGENRTIFFTIEDAVLHVQSPSTPNGDCDKS  716
                AGENRTIFFTIEDAVLHVQSPSTPNGDCDKS
Sbjct  3090823  AGENRTIFFTIEDAVLHVQSPSTPNGDCDKS  3090915
```

Range 2: 3088258 to 3088863

Score:742 bits(1514), Expect:0.0,
Method:,
Identities:202/202(100%), Positives:202/202(100%), Gaps:0/202(0%)

```
Query  1        MLHGEDSCAAVEDGGEEDQHRPLILERIEKETGRTWRTVLSQRLRKHCSCTPEKAKSRIL  60
                MLHGEDSCAAVEDGGEEDQHRPLILERIEKETGRTWRTVLSQRLRKHCSCTPEKAKSRIL
Sbjct  3088258  MLHGEDSCAAVEDGGEEDQHRPLILERIEKETGRTWRTVLSQRLRKHCSCTPEKAKSRIL  3088437

Query  61       SFFPVLQWLPRYKLRDWILGDAMSGVIVGILLVPQSIAYSLLANQDPIYGLYTSFFASII  120
                SFFPVLQWLPRYKLRDWILGDAMSGVIVGILLVPQSIAYSLLANQDPIYGLYTSFFASII
Sbjct  3088438  SFFPVLQWLPRYKLRDWILGDAMSGVIVGILLVPQSIAYSLLANQDPIYGLYTSFFASII  3088617

Query  121      YALLGTSRHISVGIFGVLCLLIGQVVDRELALAGYLPESSLSGNDSSVPLAGNDSGVVGC  180
                YALLGTSRHISVGIFGVLCLLIGQVVDRELALAGYLPESSLSGNDSSVPLAGNDSGVVGC
Sbjct  3088618  YALLGTSRHISVGIFGVLCLLIGQVVDRELALAGYLPESSLSGNDSSVPLAGNDSGVVGC  3088797

Query  181      DQSCYAIAVGATVTFTAGIYQV  202
                DQSCYAIAVGATVTFTAGIYQV
Sbjct  3088798  DQSCYAIAVGATVTFTAGIYQV  3088863
```

Range 3: 3088934 to 3089230

Score:359 bits(731), Expect:0.0,
Method:,
Identities:98/99(99%), Positives:99/99(100%), Gaps:0/99(0%)

```
Query  200      YQVLMGLLQVGFVSVYLSDSLLSGFATGASLTILTSQIKYLLGLKIPRPQGWFTLFKTWY  259
                +QVLMGLLQVGFVSVYLSDSLLSGFATGASLTILTSQIKYLLGLKIPRPQGWFTLFKTWY
Sbjct  3088934  FQVLMGLLQVGFVSVYLSDSLLSGFATGASLTILTSQIKYLLGLKIPRPQGWFTLFKTWY  3089113

Query  260      GLLSNLGNTNVCDLITSLVCLAVLIPTKELNDRFKSKLK  298
                GLLSNLGNTNVCDLITSLVCLAVLIPTKELNDRFKSKLK
Sbjct  3089114  GLLSNLGNTNVCDLITSLVCLAVLIPTKELNDRFKSKLK  3089230
```

Range 4: 3089374 to 3089823

Score:551 bits(1124), Expect:1e-160,
Method:,
Identities:150/150(100%), Positives:150/150(100%), Gaps:0/150(0%)

```
Query  299      APIPFELFVVIIATLASHFGHFNAKYGSGVAGVIPTGFLPPQMPMWSLIPNVAVDAFSIA  358
                APIPFELFVVIIATLASHFGHFNAKYGSGVAGVIPTGFLPPQMPMWSLIPNVAVDAFSIA
Sbjct  3089374  APIPFELFVVIIATLASHFGHFNAKYGSGVAGVIPTGFLPPQMPMWSLIPNVAVDAFSIA  3089553

Query  359      IVGFAITVSLSEMFAKKHGYTVDANQEMYAIGFCNILPSFFHCFTSSAALTKTLVKESTG  418
                IVGFAITVSLSEMFAKKHGYTVDANQEMYAIGFCNILPSFFHCFTSSAALTKTLVKESTG
Sbjct  3089554  IVGFAITVSLSEMFAKKHGYTVDANQEMYAIGFCNILPSFFHCFTSSAALTKTLVKESTG  3089733

Query  419      CQTQLSGLISALLLLLVLLVIAPLFYSLQK  448
                CQTQLSGLISALLLLLVLLVIAPLFYSLQK
Sbjct  3089734  CQTQLSGLISALLLLLVLLVIAPLFYSLQK  3089823
```

DC Tarpon *slc26a2*

Query: unnamed protein product ID: lcl|Query_560587(amino acid) Length: 703

>Megalops atlanticus isolate YG-15Mar2019-1 chromosome 14, whole genome shotgun sequence
Sequence ID: JAFDVH010000014.1 Length: 34728576
Range 1: 21004385 to 21005911

Score:771 bits(1990), Expect:0.0,
Method:Compositional matrix adjust.,
Identities:444/509(87%), Positives:447/509(87%), Gaps:52/509(10%)

```
Query  1         MAEADHQASAEAEPGPYVPIVLeewqkkeedwkevvkrkLRRQCTCSPGRVKALLLGFFP  60
                 MAEADHQASAEAEPGPYVPIVLEEWQKKEEDWKEVVKRKLRRQCTCSPGRVKALLLGFFP
Sbjct  21005911  MAEADHQASAEAEPGPYVPIVLEEWQKKEEDWKEVVKRKLRRQCTCSPGRVKALLLGFFP  21005732

Query  61        VISWLPHYRPKEWLLGDTMSGLIVGILLVPQSIAYSLLAGQDPIYGLYTSFFSSIIYTLL  120
                 VISWLPHYRPKEWLLGDTMSGLIVGILLVPQSIAYSLLAGQDPIYGLYTSFFSSIIYTLL
Sbjct  21005731  VISWLPHYRPKEWLLGDTMSGLIVGILLVPQSIAYSLLAGQDPIYGLYTSFFSSIIYTLL  21005552

Query  121       GTSRHISVGIFGVLCLLVGQVVDRELSLAGYLTDANQTALADAGNATAGPVCDRSCYAIV  180
                 GTSRHISVGIFGVLCLLVGQVVDRELSLAGYLTDANQTALADAGNATAGPVCDRSCYAIV
Sbjct  21005551  GTSRHISVGIFGVLCLLVGQVVDRELSLAGYLTDANQTALADAGNATAGPVCDRSCYAIV  21005372

Query  181       VGATVTFMAGVYQV----------------------------------------------  194
                 VGATVTFMAGVYQV
Sbjct  21005371  VGATVTFMAGVYQVTINLKCGK**K*RIKVVS*LLIGTVNTAEIGNVWLV**YRCGFTCL  21005192

Query  195       ------LMGLFQVGFVAVYLSDSLLSGFATGASLTILTSQVKYLLGIKIPRAQGLGSLIK  248
                       LMGLFQVGFVAVYLSDSLLSGFATGASLTILTSQVKYLLGIKIPRAQGLGSLIK
Sbjct  21005191  SLWL*VLMGLFQVGFVAVYLSDSLLSGFATGASLTILTSQVKYLLGIKIPRAQGLGSLIK  21005012
```

```
Query  249       TWVSLFKNIGQTNVCDlltsllcllvlVPSKELNNRFKAKLKAPIPFELLVVIVATLASH  308
                 TWVSLFKNIGQTNVCDLLTSLLCLLVLVPSKELNNRFKAKLKAPIPFELLVVIVATLASH
Sbjct  21005011  TWVSLFKNIGQTNVCDLLTSLLCLLVLVPSKELNNRFKAKLKAPIPFELLVVIVATLASH  21004832

Query  309       FGRFHERFGSDVSGTIPTGFLPPQLPDWSLIPNVALDALSIAIVGFAITISLSEMFAKKH  368
                 FGRFHERFGSDVSGTIPTGFLPPQLPDWSLIPNVALDALSIAIVGFAITISLSEMFAKKH
Sbjct  21004831  FGRFHERFGSDVSGTIPTGFLPPQLPDWSLIPNVALDALSIAIVGFAITISLSEMFAKKH  21004652

Query  369       GYMVDPNQEMYAIGFCNILPSFFRCFSTSAALTKTLVKESTGCHTQvsglvtalvlllvm  428
                 GYMVDPNQEMYAIGFCNILPSFFRCFSTSAALTKTLVKESTGCHTQVSGLVTALVLLLVM
Sbjct  21004651  GYMVDPNQEMYAIGFCNILPSFFRCFSTSAALTKTLVKESTGCHTQVSGLVTALVLLLVM  21004472

Query  429       lvIAPLFYSLQKCVLAVIIVVNLRGALHK  457
                 LVIAPLFYSLQK V   +  +   G L+K
Sbjct  21004471  LVIAPLFYSLQK*VNKTLTWLPFCGTLYK  21004385
```

Range 2: 21003537 to 21004328

Score:499 bits(1286), Expect:2e-154,
Method:Compositional matrix adjust.,
Identities:263/264(99%), Positives:264/264(100%), Gaps:0/264(0%)

```
Query  440       KCVLAVIIVVNLRGALHKFSDVPRMWRVNRVDAVIWLVTMATSAFVNTELGLLMGVLVSA  499
                 +CVLAVIIVVNLRGALHKFSDVPRMWRVNRVDAVIWLVTMATSAFVNTELGLLMGVLVSA
Sbjct  21004328  RCVLAVIIVVNLRGALHKFSDVPRMWRVNRVDAVIWLVTMATSAFVNTELGLLMGVLVSA  21004149

Query  500       LCVLGRTQRASALQLGQAGHLDLYLDMATYKGLSTQPGIVVFRYEAPIYYANQGLFKKAL  559
                 LCVLGRTQRASALQLGQAGHLDLYLDMATYKGLSTQPGIVVFRYEAPIYYANQGLFKKAL
Sbjct  21004148  LCVLGRTQRASALQLGQAGHLDLYLDMATYKGLSTQPGIVVFRYEAPIYYANQGLFKKAL  21003969

Query  560       YRRVGLDPIkekafrrkqekqkrkqqaDLVADEAATRSKGTEESTNVFLPQGSSFHTLVI  619
                 YRRVGLDPIKEKAFRRKQEKQKRKQQADLVADEAATRSKGTEESTNVFLPQGSSFHTLVI
Sbjct  21003968  YRRVGLDPIKEKAFRRKQEKQKRKQQADLVADEAATRSKGTEESTNVFLPQGSSFHTLVI  21003789

Query  620       DCGPVLFLDTAGVGAMKEVYKDYKALGVRVLLARCNTSVLDSLQRGGYLEHKGGDSEMVF  679
                 DCGPVLFLDTAGVGAMKEVYKDYKALGVRVLLARCNTSVLDSLQRGGYLEHKGGDSEMVF
Sbjct  21003788  DCGPVLFLDTAGVGAMKEVYKDYKALGVRVLLARCNTSVLDSLQRGGYLEHKGGDSEMVF  21003609

Query  680       FTISDAISYARSLPSQNGDCDTIC  703
                 FTISDAISYARSLPSQNGDCDTIC
Sbjct  21003608  FTISDAISYARSLPSQNGDCDTIC  21003537
```

DD West African bonefish *slc26a2*

Query: hypothetical protein AGOR_G00078660 [Albula goreensis] Query ID: KAI1899047.1 Length: 708

>Albula goreensis voucher SM-30Jul2019_1 chromosome 6, whole genome shotgun sequence
Sequence ID: JAERUA010000006.1 Length: 41944991
Range 1: 40756416 to 40757219

Score:972 bits(1984), Expect:0.0,
Method:,
Identities:268/268(100%), Positives:268/268(100%), Gaps:0/268(0%)

```
Query  441       CVLAVIIVVNLRGALRKFTDVPRMWRVNRVDASIWLVTMATSALVNTELGLLVGVLASAL  500
                 CVLAVIIVVNLRGALRKFTDVPRMWRVNRVDASIWLVTMATSALVNTELGLLVGVLASAL
Sbjct  40756416  CVLAVIIVVNLRGALRKFTDVPRMWRVNRVDASIWLVTMATSALVNTELGLLVGVLASAL  40756595

Query  501       SVLGRTQRAAALQLGQAGPQHPELYADLAAYKGLNTQPGVVVFRYEAPIYYANQALFKKA  560
                 SVLGRTQRAAALQLGQAGPQHPELYADLAAYKGLNTQPGVVVFRYEAPIYYANQALFKKA
Sbjct  40756596  SVLGRTQRAAALQLGQAGPQHPELYADLAAYKGLNTQPGVVVFRYEAPIYYANQALFKKA  40756775

Query  561       LYRRVGLDPLKEKTRRKKQEKQRRKQEAAGAEGEKVAARAQDGEVSTNIFLPQGKPGFHT  620
                 LYRRVGLDPLKEKTRRKKQEKQRRKQEAAGAEGEKVAARAQDGEVSTNIFLPQGKPGFHT
Sbjct  40756776  LYRRVGLDPLKEKTRRKKQEKQRRKQEAAGAEGEKVAARAQDGEVSTNIFLPQGKPGFHT  40756955

Query  621       LVVDCGPVLFLDTAGVGALKEVRKDYKELGVRVLLAQCNTSVLDSLHRGDYLEHKGGDSE  680
                 LVVDCGPVLFLDTAGVGALKEVRKDYKELGVRVLLAQCNTSVLDSLHRGDYLEHKGGDSE
Sbjct  40756956  LVVDCGPVLFLDTAGVGALKEVRKDYKELGVRVLLAQCNTSVLDSLHRGDYLEHKGGDSE  40757135

Query  681       TVFFTIDDAVGYACSLSAQNGGCGDTYC  708
                 TVFFTIDDAVGYACSLSAQNGGCGDTYC
Sbjct  40757136  TVFFTIDDAVGYACSLSAQNGGCGDTYC  40757219
```

Range 2: 40755463 to 40756203

Score:895 bits(1828), Expect:0.0,
Method:,
Identities:247/247(100%), Positives:247/247(100%), Gaps:0/247(0%)

```
Query  194       VLMGLLQVGFVSVYLSDSLLSGFATGASLTILTSQVKYLLGLKLPRAQGLGSLLKTWYSL  253
                 VLMGLLQVGFVSVYLSDSLLSGFATGASLTILTSQVKYLLGLKLPRAQGLGSLLKTWYSL
Sbjct  40755463  VLMGLLQVGFVSVYLSDSLLSGFATGASLTILTSQVKYLLGLKLPRAQGLGSLLKTWYSL  40755642

Query  254       FSNIGQTNICDLLTSLVCLLLLVPSKELNDRFKSKLKAPIPFELFVVIVATLASHYGQFN  313
```

```
              FSNIGQTNICDLLTSLVCLLLLVPSKELNDRFKSKLKAPIPFELFVVIVATLASHYGQFN
Sbjct  40755643  FSNIGQTNICDLLTSLVCLLLLVPSKELNDRFKSKLKAPIPFELFVVIVATLASHYGQFN  40755822

Query  314       EKFGSDISGTIPTGFLPPQLPMWSLIPNIAVDALSIAIVGFAITVSLSEMFAKKHGYSVD  373
              EKFGSDISGTIPTGFLPPQLPMWSLIPNIAVDALSIAIVGFAITVSLSEMFAKKHGYSVD
Sbjct  40755823  EKFGSDISGTIPTGFLPPQLPMWSLIPNIAVDALSIAIVGFAITVSLSEMFAKKHGYSVD  40756002

Query  374       ANQEMYAIGFCNILPSFFRCFTTSAALTKTLVKESTGCQTQVSGLVTALVLLLVLLVIAP  433
              ANQEMYAIGFCNILPSFFRCFTTSAALTKTLVKESTGCQTQVSGLVTALVLLLVLLVIAP
Sbjct  40756003  ANQEMYAIGFCNILPSFFRCFTTSAALTKTLVKESTGCQTQVSGLVTALVLLLVLLVIAP  40756182

Query  434       LFYSLQK  440
              LFYSLQK
Sbjct  40756183  LFYSLQK  40756203
```

Range 3: 40754568 to 40755149

Score:712 bits(1454), Expect:0.0,
Method:,
Identities:194/194(100%), Positives:194/194(100%), Gaps:0/194(0%)

```
Query  1         MDEEEREASAGGGAMPAAYVPISLEEQERREESWKELVKRKVRKQCVCSPARVKSLLLGF  60
              MDEEEREASAGGGAMPAAYVPISLEEQERREESWKELVKRKVRKQCVCSPARVKSLLLGF
Sbjct  40754568  MDEEEREASAGGGAMPAAYVPISLEEQERREESWKELVKRKVRKQCVCSPARVKSLLLGF  40754747

Query  61        CPALSWLPRYRLREWFLGDAMSGLIVGILLVPQSIAYSLLAGQDPIYGLYTSFFSSIIYT  120
              CPALSWLPRYRLREWFLGDAMSGLIVGILLVPQSIAYSLLAGQDPIYGLYTSFFSSIIYT
Sbjct  40754748  CPALSWLPRYRLREWFLGDAMSGLIVGILLVPQSIAYSLLAGQDPIYGLYTSFFSSIIYT  40754927

Query  121       LLGTSRHISVGIFGVLCLLVGQVVDRELALAGYLNNNQTAALPANGTVGPACDRSCYAIT  180
              LLGTSRHISVGIFGVLCLLVGQVVDRELALAGYLNNNQTAALPANGTVGPACDRSCYAIT
Sbjct  40754928  LLGTSRHISVGIFGVLCLLVGQVVDRELALAGYLNNNQTAALPANGTVGPACDRSCYAIT  40755107

Query  181       VGATVTFTAGVYQV  194
              VGATVTFTAGVYQV
Sbjct  40755108  VGATVTFTAGVYQV  40755149
```

DE European eel *slc26a2*

Query: sulfate transporter [Anguilla anguilla] Query ID: XP_035266276.1 Length: 712

>Anguilla anguilla isolate fAngAng1 chromosome 3, fAngAng1.pri, whole genome shotgun sequence
Sequence ID: NC_049203.1 Length: 71675879
Range 1: 49845362 to 49846159

Score:974 bits(1988), Expect:0.0,
Method:,
Identities:266/266(100%), Positives:266/266(100%), Gaps:0/266(0%)

```
Query  447       CVLAVIIVVNLRGALRKFADIPRMWRENHVDATIWLVTMGTSALVNTELGLLVGVLFSAF  506
              CVLAVIIVVNLRGALRKFADIPRMWRENHVDATIWLVTMGTSALVNTELGLLVGVLFSAF
Sbjct  49845362  CVLAVIIVVNLRGALRKFADIPRMWRENHVDATIWLVTMGTSALVNTELGLLVGVLFSAF  49845541

Query  507       CVLGRTQRAPALQLGQAGHLDLYEDTAAYKGLKTLPGILVFRYEAPVYYANQTLFKKALY  566
              CVLGRTQRAPALQLGQAGHLDLYEDTAAYKGLKTLPGILVFRYEAPVYYANQTLFKKALY
Sbjct  49845542  CVLGRTQRAPALQLGQAGHLDLYEDTAAYKGLKTLPGILVFRYEAPVYYANQTLFKKALY  49845721

Query  567       RCAGLDPVQEKARRRKQEKQKRKREADANAEAGNADAQGGDAEVSTKIFLPQGAGLHTLV  626
              RCAGLDPVQEKARRRKQEKQKRKREADANAEAGNADAQGGDAEVSTKIFLPQGAGLHTLV
Sbjct  49845722  RCAGLDPVQEKARRRKQEKQKRKREADANAEAGNADAQGGDAEVSTKIFLPQGAGLHTLV  49845901

Query  627       IDCGPVLFLDTAGVGAMKELQKDYREMGVRMLLARCNTSVLDSLHRGGYLERGGGDAEKV  686
              IDCGPVLFLDTAGVGAMKELQKDYREMGVRMLLARCNTSVLDSLHRGGYLERGGGDAEKV
Sbjct  49845902  IDCGPVLFLDTAGVGAMKELQKDYREMGVRMLLARCNTSVLDSLHRGGYLERGGGDAEKV  49846081

Query  687       FFTIDDAVSYARSLPCQNGDCDASLL  712
              FFTIDDAVSYARSLPCQNGDCDASLL
Sbjct  49846082  FFTIDDAVSYARSLPCQNGDCDASLL  49846159
```

Range 2: 49844078 to 49844821

Score:896 bits(1830), Expect:0.0,
Method:,
Identities:248/248(100%), Positives:248/248(100%), Gaps:0/248(0%)

```
Query  199       QVLMGLLQVGFVSVYLSDSLLSGFATGASLTILTSQVKYLLGLKLPRAHGLGSLVQTWVS  258
              QVLMGLLQVGFVSVYLSDSLLSGFATGASLTILTSQVKYLLGLKLPRAHGLGSLVQTWVS
Sbjct  49844078  QVLMGLLQVGFVSVYLSDSLLSGFATGASLTILTSQVKYLLGLKLPRAHGLGSLVQTWVS  49844257

Query  259       LFRNIGQTNVCDLVTSLVCLLVLVPSKELNDRFKARLKAPVPFELFVVIGATLASHYGRF  318
              LFRNIGQTNVCDLVTSLVCLLVLVPSKELNDRFKARLKAPVPFELFVVIGATLASHYGRF
Sbjct  49844258  LFRNIGQTNVCDLVTSLVCLLVLVPSKELNDRFKARLKAPVPFELFVVIGATLASHYGRF  49844437

Query  319       QERFGSDVSGTIPTGFLPPQLPSWSLIPNVALDALSIAIVGFAITVSLSEMFAKKHGYLV  378
```

```
                 QERFGSDVSGTIPTGFLPPQLPSWSLIPNVALDALSIAIVGFAITVSLSEMFAKKHGYLV
Sbjct  49844438  QERFGSDVSGTIPTGFLPPQLPSWSLIPNVALDALSIAIVGFAITVSLSEMFAKKHGYLV  49844617

Query  379       DANQEMYAIGFCNILPSFFRCFTTSAALTKTLVKESTGCQTQVSGLVTALVLLLVLLVIA  438
                 DANQEMYAIGFCNILPSFFRCFTTSAALTKTLVKESTGCQTQVSGLVTALVLLLVLLVIA
Sbjct  49844618  DANQEMYAIGFCNILPSFFRCFTTSAALTKTLVKESTGCQTQVSGLVTALVLLLVLLVIA  49844797

Query  439       PLFYSLQK  446
                 PLFYSLQK
Sbjct  49844798  PLFYSLQK  49844821
```

Range 3: 49843134 to 49843733

Score:733 bits(1497), Expect:0.0,
Method:,
Identities:200/200(100%), Positives:200/200(100%), Gaps:0/200(0%)

```
Query  1         MAGEESEALAESPAESPAVPGAYVPFVLEEREKRREGWKELARREAGKRCACSADRAKAL  60
                 MAGEESEALAESPAESPAVPGAYVPFVLEEREKRREGWKELARREAGKRCACSADRAKAL
Sbjct  49843134  MAGEESEALAESPAESPAVPGAYVPFVLEEREKRREGWKELARREAGKRCACSADRAKAL  49843313

Query  61        VLGFFPILSWLPGYRPRDWLVGDMMSGLIVGILLVPQSIAYSLLAGQDPVYGLYTSFFSS  120
                 VLGFFPILSWLPGYRPRDWLVGDMMSGLIVGILLVPQSIAYSLLAGQDPVYGLYTSFFSS
Sbjct  49843314  VLGFFPILSWLPGYRPRDWLVGDMMSGLIVGILLVPQSIAYSLLAGQDPVYGLYTSFFSS  49843493

Query  121       IIYTLLGTSRHISVGIFGVLCLLVGQVVDRELMLAGYLGETNQTALAGAGNLTVGPICDR  180
                 IIYTLLGTSRHISVGIFGVLCLLVGQVVDRELMLAGYLGETNQTALAGAGNLTVGPICDR
Sbjct  49843494  IIYTLLGTSRHISVGIFGVLCLLVGQVVDRELMLAGYLGETNQTALAGAGNLTVGPICDR  49843673

Query  181       SCYAIMVGATVTFTAGVYQV  200
                 SCYAIMVGATVTFTAGVYQV
Sbjct  49843674  SCYAIMVGATVTFTAGVYQV  49843733
```

DF European conger *slc26a2*

Query: hypothetical protein COCON_G00046610 [Conger conger] Query ID: KAJ8282142.1 Length: 703

>Conger conger isolate Concon-B chromosome 3, whole genome shotgun sequence
Sequence ID: JAFJMO010000003.1 Length: 73127133
Range 1: 23893504 to 23894298

Score:966 bits(1973), Expect:0.0,
Method:,
Identities:265/265(100%), Positives:265/265(100%), Gaps:0/265(0%)

```
Query  439       CVLAVIIVVNLRGALRKFADVPRMWRVNHIDATIWLLTMATSALVNTELGLLVGVLVSAF  498
                 CVLAVIIVVNLRGALRKFADVPRMWRVNHIDATIWLLTMATSALVNTELGLLVGVLVSAF
Sbjct  23894298  CVLAVIIVVNLRGALRKFADVPRMWRVNHIDATIWLLTMATSALVNTELGLLVGVLVSAF  23894119

Query  499       CVLGRTQRAAALQLGQAGHLDLYEDPTAYKGLNTVPGTVIFRYEAPVYYANQTLFKKALY  558
                 CVLGRTQRAAALQLGQAGHLDLYEDPTAYKGLNTVPGTVIFRYEAPVYYANQTLFKKALY
Sbjct  23894118  CVLGRTQRAAALQLGQAGHLDLYEDPTAYKGLNTVPGTVIFRYEAPVYYANQTLFKKALY  23893939

Query  559       RSVGLDPVQEKAKRRKQEKQKRKQEVNANAELESVGTQVKDVEVSKAFVPAGAGFHTLVI  618
                 RSVGLDPVQEKAKRRKQEKQKRKQEVNANAELESVGTQVKDVEVSKAFVPAGAGFHTLVI
Sbjct  23893938  RSVGLDPVQEKAKRRKQEKQKRKQEVNANAELESVGTQVKDVEVSKAFVPAGAGFHTLVI  23893759

Query  619       DCGPVLFLDTAGVGAMKELLKDYKELGVRVLLARCNTSVLDSLHRGGYLEHNSSDAEKVF  678
                 DCGPVLFLDTAGVGAMKELLKDYKELGVRVLLARCNTSVLDSLHRGGYLEHNSSDAEKVF
Sbjct  23893758  DCGPVLFLDTAGVGAMKELLKDYKELGVRVLLARCNTSVLDSLHRGGYLEHNSSDAEKVF  23893579

Query  679       FTIDDAVSYAHSLPCQNGDCDASRL  703
                 FTIDDAVSYAHSLPCQNGDCDASRL
Sbjct  23893578  FTIDDAVSYAHSLPCQNGDCDASRL  23893504
```

Range 2: 23894986 to 23895729

Score:898 bits(1834), Expect:0.0,
Method:,
Identities:248/248(100%), Positives:248/248(100%), Gaps:0/248(0%)

```
Query  191       QVLMGLLQVGFVSVYLSDSLLSGFATGASLTILTSQVKYLLGLKLPRAQGLGSLVKTWIS  250
                 QVLMGLLQVGFVSVYLSDSLLSGFATGASLTILTSQVKYLLGLKLPRAQGLGSLVKTWIS
Sbjct  23895729  QVLMGLLQVGFVSVYLSDSLLSGFATGASLTILTSQVKYLLGLKLPRAQGLGSLVKTWIS  23895550

Query  251       LFTNIGKTNVCDLLTSLVCLLLLVPSKELNDRFKAKLKAPIPFELFVVIAATLASHYGHF  310
                 LFTNIGKTNVCDLLTSLVCLLLLVPSKELNDRFKAKLKAPIPFELFVVIAATLASHYGHF
Sbjct  23895549  LFTNIGKTNVCDLLTSLVCLLLLVPSKELNDRFKAKLKAPIPFELFVVIAATLASHYGHF  23895370

Query  311       HDRFGSEVSGTIPTGFLPPQLPDWSLIPNIALDALSIAIVGFAITVSLSEMFAKKHGYVV  370
                 HDRFGSEVSGTIPTGFLPPQLPDWSLIPNIALDALSIAIVGFAITVSLSEMFAKKHGYVV
Sbjct  23895369  HDRFGSEVSGTIPTGFLPPQLPDWSLIPNIALDALSIAIVGFAITVSLSEMFAKKHGYVV  23895190

Query  371       DPNQEMYAIGFCNILPSFFRCFTTSAALTKTLVKESTGCQTQVSGLVTALVLLLVLLVIA  430
```

```
              DPNQEMYAIGFCNILPSFFRCFTTSAALTKTLVKESTGCQTQVSGLVTALVLLLVLLVIA
Sbjct  23895189  DPNQEMYAIGFCNILPSFFRCFTTSAALTKTLVKESTGCQTQVSGLVTALVLLLVLLVIA  23895010

Query  431       PLFYSLQK  438
                 PLFYSLQK
Sbjct  23895009  PLFYSLQK  23894986
```

Range 3: 23896133 to 23896708

Score:705 bits(1439), Expect:0.0,
Method:,
Identities:192/192(100%), Positives:192/192(100%), Gaps:0/192(0%)

```
Query  1         MAAEESEALTEPSAYMPFALEEQERKEESWKELARRKVGKQCACSADRAKALVLGFFPIL  60
                 MAAEESEALTEPSAYMPFALEEQERKEESWKELARRKVGKQCACSADRAKALVLGFFPIL
Sbjct  23896708  MAAEESEALTEPSAYMPFALEEQERKEESWKELARRKVGKQCACSADRAKALVLGFFPIL  23896529

Query  61        SWLPRYRLKDWLLGDMMSGLIVGILLVPQSIAYSLLAGQDPVYGLYTSFFSSIIYTLLGT  120
                 SWLPRYRLKDWLLGDMMSGLIVGILLVPQSIAYSLLAGQDPVYGLYTSFFSSIIYTLLGT
Sbjct  23896528  SWLPRYRLKDWLLGDMMSGLIVGILLVPQSIAYSLLAGQDPVYGLYTSFFSSIIYTLLGT  23896349

Query  121       SRHISVGIFGVLCLLVGQVVDRELLLAGYITEGNQTALAGPGNVTVGQTCDRSCYAIAVG  180
                 SRHISVGIFGVLCLLVGQVVDRELLLAGYITEGNQTALAGPGNVTVGQTCDRSCYAIAVG
Sbjct  23896348  SRHISVGIFGVLCLLVGQVVDRELLLAGYITEGNQTALAGPGNVTVGQTCDRSCYAIAVG  23896169

Query  181       ATVTFTAGVYQV  192
                 ATVTFTAGVYQV
Sbjct  23896168  ATVTFTAGVYQV  23896133
```

DG Atlantic herring *slc26a2*

Query: sulfate transporter [Clupea harengus] Query ID: XP_031424802.1 Length: 732

>Clupea harengus chromosome 6, Ch_v2.0.2, whole genome shotgun sequence
Sequence ID: NC_045157.1 Length: 31461554
Range 1: 9688030 to 9688878

Score:1029 bits(2101), Expect:0.0,
Method:,
Identities:283/283(100%), Positives:283/283(100%), Gaps:0/283(0%)

```
Query  450      CVLAVIIVVNLRGALRKFTDVPRMWRANHVDASIWLITMATSALVNTELGLLVGVLVSAF  509
                CVLAVIIVVNLRGALRKFTDVPRMWRANHVDASIWLITMATSALVNTELGLLVGVLVSAF
Sbjct  9688878  CVLAVIIVVNLRGALRKFTDVPRMWRANHVDASIWLITMATSALVNTELGLLVGVLVSAF  9688699

Query  510      CVLGRTQRAGAVQLGRSGEADLYEDMGWYRGLRAQPGVAVFRYEAPIYYANQVLFKRALY  569
                CVLGRTQRAGAVQLGRSGEADLYEDMGWYRGLRAQPGVAVFRYEAPIYYANQVLFKRALY
Sbjct  9688698  CVLGRTQRAGAVQLGRSGEADLYEDMGWYRGLRAQPGVAVFRYEAPIYYANQVLFKRALY  9688519

Query  570      HAVGLDPVHEKARRRRQEKRRMKLEEGALAAAGAQRGSGDERGAPVSGGGTCPPESQEVG  629
                HAVGLDPVHEKARRRRQEKRRMKLEEGALAAAGAQRGSGDERGAPVSGGGTCPPESQEVG
Sbjct  9688518  HAVGLDPVHEKARRRRQEKRRMKLEEGALAAAGAQRGSGDERGAPVSGGGTCPPESQEVG  9688339

Query  630      VCSGEAILPQRANFHSLVLDCSPVLFLDSAGVGALQEVRKDYAEVGVRLLLTRCRPSVTE  689
                VCSGEAILPQRANFHSLVLDCSPVLFLDSAGVGALQEVRKDYAEVGVRLLLTRCRPSVTE
Sbjct  9688338  VCSGEAILPQRANFHSLVLDCSPVLFLDSAGVGALQEVRKDYAEVGVRLLLTRCRPSVTE  9688159

Query  690      SLQRAGYLEDKGGHSEVVFLTIADAVLYAQSLSSQNGGCENSC  732
                SLQRAGYLEDKGGHSEVVFLTIADAVLYAQSLSSQNGGCENSC
Sbjct  9688158  SLQRAGYLEDKGGHSEVVFLTIADAVLYAQSLSSQNGGCENSC  9688030
```

Range 2: 9689112 to 9689855

Score:896 bits(1829), Expect:0.0,
Method:,
Identities:248/248(100%), Positives:248/248(100%), Gaps:0/248(0%)

```
Query  202      QVLMGLLQVGFVSVFLSDSLLSGFATGASLTILTSQLKYLLGLKLPRAQGWGSLMKTWAS  261
                QVLMGLLQVGFVSVFLSDSLLSGFATGASLTILTSQLKYLLGLKLPRAQGWGSLMKTWAS
Sbjct  9689855  QVLMGLLQVGFVSVFLSDSLLSGFATGASLTILTSQLKYLLGLKLPRAQGWGSLMKTWAS  9689676

Query  262      LLSNLGSTNLCDLLTSLLCILVLVPAKELNDRFKAKLPAPIPFELFVVIAATLASHFGGF  321
                LLSNLGSTNLCDLLTSLLCILVLVPAKELNDRFKAKLPAPIPFELFVVIAATLASHFGGF
Sbjct  9689675  LLSNLGSTNLCDLLTSLLCILVLVPAKELNDRFKAKLPAPIPFELFVVIAATLASHFGGF  9689496

Query  322      RELYGSEVAGAIPTGFLPPQLPAWHLIPNVAVDAFSIALVGFAITVSLSEMFAKKHGYAV  381
                RELYGSEVAGAIPTGFLPPQLPAWHLIPNVAVDAFSIALVGFAITVSLSEMFAKKHGYAV
Sbjct  9689495  RELYGSEVAGAIPTGFLPPQLPAWHLIPNVAVDAFSIALVGFAITVSLSEMFAKKHGYAV  9689316

Query  382      DANQEMFAIGFCNILPSFFRCFTTSAALTKTLVKESTGCQTQLSALVTALVLLLVLLLIA  441
                DANQEMFAIGFCNILPSFFRCFTTSAALTKTLVKESTGCQTQLSALVTALVLLLVLLLIA
Sbjct  9689315  DANQEMFAIGFCNILPSFFRCFTTSAALTKTLVKESTGCQTQLSALVTALVLLLVLLLIA  9689136

Query  442      PLFYSLQK  449
```

```
             PLFYSLQK
Sbjct  9689135  PLFYSLQK  9689112
```

Range 3: 9690591 to 9691199

Score:744 bits(1519), Expect:0.0,
Method:,
Identities:203/203(100%), Positives:203/203(100%), Gaps:0/203(0%)

```
Query  1        MAEEAGEAGQVPDGDRICPLHTPLTLEEWEHDEEPLSAQVRRRAKKLCTCSHARAKALLL  60
                MAEEAGEAGQVPDGDRICPLHTPLTLEEWEHDEEPLSAQVRRRAKKLCTCSHARAKALLL
Sbjct  9691199  MAEEAGEAGQVPDGDRICPLHTPLTLEEWEHDEEPLSAQVRRRAKKLCTCSHARAKALLL  9691020

Query  61       DSVPILRWLPRYRLREWLLGDVMSGVIVGILLVPQSIAYSLLAGQDPIYGLYTSFFSSII  120
                DSVPILRWLPRYRLREWLLGDVMSGVIVGILLVPQSIAYSLLAGQDPIYGLYTSFFSSII
Sbjct  9691019  DSVPILRWLPRYRLREWLLGDVMSGVIVGILLVPQSIAYSLLAGQDPIYGLYTSFFSSII  9690840

Query  121      YALLGSSRHISVGIFGVLCLLVGQVVDRELALAGYPSENSANLTADVTGAAVENGTMGLV  180
                YALLGSSRHISVGIFGVLCLLVGQVVDRELALAGYPSENSANLTADVTGAAVENGTMGLV
Sbjct  9690839  YALLGSSRHISVGIFGVLCLLVGQVVDRELALAGYPSENSANLTADVTGAAVENGTMGLV  9690660

Query  181      CDRSCYAILVGATVTFTAGIYQV  203
                CDRSCYAILVGATVTFTAGIYQV
Sbjct  9690659  CDRSCYAILVGATVTFTAGIYQV  9690591
```

DH Milkfish *slc26a2*

Query: sulfate transporter-like [Chanos chanos] Query ID: XP_030620530.1 Length: 700

>Chanos chanos chromosome 2, fChaCha1.1, whole genome shotgun sequence
Sequence ID: NC_044496.1 Length: 59736555
Range 1: 57884599 to 57885378

Score:951 bits(1942), Expect:0.0,
Method:,
Identities:260/260(100%), Positives:260/260(100%), Gaps:0/260(0%)

```
Query  441       CVLAVIIVVNLRGALRKFRDVPRMWRVNRVDAAIWLVTMATSALVNTELGLLVGVLVSAF  500
                 CVLAVIIVVNLRGALRKFRDVPRMWRVNRVDAAIWLVTMATSALVNTELGLLVGVLVSAF
Sbjct  57884599  CVLAVIIVVNLRGALRKFRDVPRMWRVNRVDAAIWLVTMATSALVNTELGLLVGVLVSAF  57884778

Query  501       CVLGRTQSARALQLGKAGELEVYEDLASYKGLQTQPGVAIFRYEAPIYYANQALFKKCLY  560
                 CVLGRTQSARALQLGKAGELEVYEDLASYKGLQTQPGVAIFRYEAPIYYANQALFKKCLY
Sbjct  57884779  CVLGRTQSARALQLGKAGELEVYEDLASYKGLQTQPGVAIFRYEAPIYYANQALFKKCLY  57884958

Query  561       RSVGLDPLKEKARRKKLERQKQKQEKSEEGGAKGKEPESSTSVFLAHCVSFHTLILDCSP  620
                 RSVGLDPLKEKARRKKLERQKQKQEKSEEGGAKGKEPESSTSVFLAHCVSFHTLILDCSP
Sbjct  57884959  RSVGLDPLKEKARRKKLERQKQKQEKSEEGGAKGKEPESSTSVFLAHCVSFHTLILDCSP  57885138

Query  621       VLFLDTAGVNALKEVCKDYRELGVRLLLAQCNISVIESLRKGGYYNQKDGNTEKLFFTIG  680
                 VLFLDTAGVNALKEVCKDYRELGVRLLLAQCNISVIESLRKGGYYNQKDGNTEKLFFTIG
Sbjct  57885139  VLFLDTAGVNALKEVCKDYRELGVRLLLAQCNISVIESLRKGGYYNQKDGNTEKLFFTIG  57885318

Query  681       DAVCYAKSLSSQNGDCDTVC  700
                 DAVCYAKSLSSQNGDCDTVC
Sbjct  57885319  DAVCYAKSLSSQNGDCDTVC  57885378
```

Range 2: 57883601 to 57884350

Score:913 bits(1864), Expect:0.0,
Method:,
Identities:249/250(99%), Positives:250/250(100%), Gaps:0/250(0%)

```
Query  191       VYQVLMGLLQVGFVSVFLSDSLLSGFATGASLTILTSQLKYLLGIKVPRAQGWFTLFKTW  250
                 V+QVLMGLLQVGFVSVFLSDSLLSGFATGASLTILTSQLKYLLGIKVPRAQGWFTLFKTW
Sbjct  57883601  VFQVLMGLLQVGFVSVFLSDSLLSGFATGASLTILTSQLKYLLGIKVPRAQGWFTLFKTW  57883780

Query  251       ISLLRNIHKTNVCDLVTSIICLLVLVPTKELNDRFKSKLKAPIPFELFVVIVATLASHFG  310
                 ISLLRNIHKTNVCDLVTSIICLLVLVPTKELNDRFKSKLKAPIPFELFVVIVATLASHFG
Sbjct  57883781  ISLLRNIHKTNVCDLVTSIICLLVLVPTKELNDRFKSKLKAPIPFELFVVIVATLASHFG  57883960

Query  311       HFQEEYGSDVSGDIPTGFMPPQLPAWSLIPNVAVDAFSIAIVGFAITVSLSEMFAKKHGY  370
                 HFQEEYGSDVSGDIPTGFMPPQLPAWSLIPNVAVDAFSIAIVGFAITVSLSEMFAKKHGY
Sbjct  57883961  HFQEEYGSDVSGDIPTGFMPPQLPAWSLIPNVAVDAFSIAIVGFAITVSLSEMFAKKHGY  57884140

Query  371       VVDANQEMYAIGFCNIFPSFFRCFTTSAALTKTLVKESTGCQTQLSGLVTALVLLLVLLV  430
                 VVDANQEMYAIGFCNIFPSFFRCFTTSAALTKTLVKESTGCQTQLSGLVTALVLLLVLLV
Sbjct  57884141  VVDANQEMYAIGFCNIFPSFFRCFTTSAALTKTLVKESTGCQTQLSGLVTALVLLLVLLV  57884320

Query  431       IAPLFYSLQK  440
                 IAPLFYSLQK
Sbjct  57884321  IAPLFYSLQK  57884350
```

Range 3: 57882490 to 57883071

Score:714 bits(1457), Expect:0.0,
Method:,
Identities:194/194(100%), Positives:194/194(100%), Gaps:0/194(0%)

```
Query  1         MMTGDDCASEDGAQSGPFTPLILEEQEKEAESWTNVLIRKVKKRCSCSRAQAKALLLDSM  60
                 MMTGDDCASEDGAQSGPFTPLILEEQEKEAESWTNVLIRKVKKRCSCSRAQAKALLLDSM
Sbjct  57882490  MMTGDDCASEDGAQSGPFTPLILEEQEKEAESWTNVLIRKVKKRCSCSRAQAKALLLDSM  57882669

Query  61        PILRWLPRYQLRAWLLGDVMSGLIVGILLVPQSIAYSLLAGQDPIYGLYTSFFSSIIYTV  120
                 PILRWLPRYQLRAWLLGDVMSGLIVGILLVPQSIAYSLLAGQDPIYGLYTSFFSSIIYTV
Sbjct  57882670  PILRWLPRYQLRAWLLGDVMSGLIVGILLVPQSIAYSLLAGQDPIYGLYTSFFSSIIYTV  57882849

Query  121       LGSSRHISVGIFGVLCLLVGQVVDRELVAAGYPTETNQTAVDGLQNVTGPLCDRSCYAIT  180
                 LGSSRHISVGIFGVLCLLVGQVVDRELVAAGYPTETNQTAVDGLQNVTGPLCDRSCYAIT
Sbjct  57882850  LGSSRHISVGIFGVLCLLVGQVVDRELVAAGYPTETNQTAVDGLQNVTGPLCDRSCYAIT  57883029

Query  181       VGATVTFTAGVYQV  194
                 VGATVTFTAGVYQV
Sbjct  57883030  VGATVTFTAGVYQV  57883071
```

DI Fathead minnow *slc26a2*

Query: sulfate transporter [Pimephales promelas] Query ID: XP_039510420.1 Length: 700

>Pimephales promelas strain EPAAWBERC unplaced genomic scaffold, EPA_FHM_2.0 scaf66, whole genome shotgun sequence
Sequence ID: NW_024121721.1 Length: 3149979
Range 1: 2178848 to 2179618

Score:945 bits(1929), Expect:0.0,
Method:,
Identities:257/257(100%), Positives:257/257(100%), Gaps:0/257(0%)

```
Query  444      CVLAVIIVVNLRGALRKFVDIPKMWRVNRVDAIIWLVTMSTSALVNTELGLLVGVLLSAF  503
                CVLAVIIVVNLRGALRKFVDIPKMWRVNRVDAIIWLVTMSTSALVNTELGLLVGVLLSAF
Sbjct  2178848  CVLAVIIVVNLRGALRKFVDIPKMWRVNRVDAIIWLVTMSTSALVNTELGLLVGVLLSAF  2179027

Query  504      CVLGRTQCAQVLQLGQAGDRELFEDIGFYKGLQTQPDVAVFRYEAPIYYANQTLFKKSLY  563
                CVLGRTQCAQVLQLGQAGDRELFEDIGFYKGLQTQPDVAVFRYEAPIYYANQTLFKKSLY
Sbjct  2179028  CVLGRTQCAQVLQLGQAGDRELFEDIGFYKGLQTQPDVAVFRYEAPIYYANQTLFKKSLY  2179207

Query  564      RSVGLDPLKEKARRRKLEKQRKQKQDGEDPKQEIEVSTNVYLLKHTSLHALVIDCSPVLF  623
                RSVGLDPLKEKARRRKLEKQRKQKQDGEDPKQEIEVSTNVYLLKHTSLHALVIDCSPVLF
Sbjct  2179208  RSVGLDPLKEKARRRKLEKQRKQKQDGEDPKQEIEVSTNVYLLKHTSLHALVIDCSPVLF  2179387

Query  624      LDTAGVNALKEVCKDYKELGVNVLLAQCNISVIDSLRRGGYYDPKKGTKEIKFHTVGDAI  683
                LDTAGVNALKEVCKDYKELGVNVLLAQCNISVIDSLRRGGYYDPKKGTKEIKFHTVGDAI
Sbjct  2179388  LDTAGVNALKEVCKDYKELGVNVLLAQCNISVIDSLRRGGYYDPKKGTKEIKFHTVGDAI  2179567

Query  684      SYAQSLKSKNGDCDTVV  700
                SYAQSLKSKNGDCDTVV
Sbjct  2179568  SYAQSLKSKNGDCDTVV  2179618
```

Range 2: 2177470 to 2178222

Score:915 bits(1869), Expect:0.0,
Method:,
Identities:250/251(99%), Positives:250/251(99%), Gaps:0/251(0%)

```
Query  196      QVLMGLLQVGFVSVFLSDSLLSGFATGASLTILTSQMKYFLGLHLPRVQGWGSLIKTWIS  255
                QVLMGLLQVGFVSVFLSDSLLSGFATGASLTILTSQMKYFLGLHLPRVQGWGSLIKTWIS
Sbjct  2177470  QVLMGLLQVGFVSVFLSDSLLSGFATGASLTILTSQMKYFLGLHLPRVQGWGSLIKTWIS  2177649

Query  256      LLKNLGHTNICDLITSGICLLVLVPTKELNNRFKAKLKAPIPFELFVVIAATLASHFGKF  315
                LLKNLGHTNICDLITSGICLLVLVPTKELNNRFKAKLKAPIPFELFVVIAATLASHFGKF
Sbjct  2177650  LLKNLGHTNICDLITSGICLLVLVPTKELNNRFKAKLKAPIPFELFVVIAATLASHFGKF  2177829

Query  316      KENYGSDVAGNIPTGFMPPQLPNWSLIPNIAVDAFSIAIVGFAITVSLSEMFAKKHGYMV  375
                KENYGSDVAGNIPTGFMPPQLPNWSLIPNIAVDAFSIAIVGFAITVSLSEMFAKKHGYMV
Sbjct  2177830  KENYGSDVAGNIPTGFMPPQLPNWSLIPNIAVDAFSIAIVGFAITVSLSEMFAKKHGYMV  2178009

Query  376      DPNQEMYAIGFCNIIPSFFHCFTTSAALTKTLVKESTGCQTQVSGLVTALVLLLALLVIA  435
                DPNQEMYAIGFCNIIPSFFHCFTTSAALTKTLVKESTGCQTQVSGLVTALVLLLALLVIA
Sbjct  2178010  DPNQEMYAIGFCNIIPSFFHCFTTSAALTKTLVKESTGCQTQVSGLVTALVLLLALLVIA  2178189

Query  436      PLFYSLQKCVL  446
                PLFYSLQK VL
Sbjct  2178190  PLFYSLQKYVL  2178222
```

Range 3: 2176801 to 2177391

Score:732 bits(1493), Expect:0.0,
Method:,
Identities:197/197(100%), Positives:197/197(100%), Gaps:0/197(0%)

Query 1   MPVEEVCGSVSAEDDANLGLDVPFLLEECVKEKVPWKEVLKRKVVKKCSCSHARVKALLV 60
                MPVEEVCGSVSAEDDANLGLDVPFLLEECVKEKVPWKEVLKRKVVKKCSCSHARVKALLV
Sbjct 2176801 MPVEEVCGSVSAEDDANLGLDVPFLLEECVKEKVPWKEVLKRKVVKKCSCSHARVKALLV 2176980

Query 61   DSIPIVKWLPRYQFKDWIIGDVMSGLIVAILLVPQSIAYSLLAEQDPIYGLYTSFFASII 120
                DSIPIVKWLPRYQFKDWIIGDVMSGLIVAILLVPQSIAYSLLAEQDPIYGLYTSFFASII
Sbjct 2176981 DSIPIVKWLPRYQFKDWIIGDVMSGLIVAILLVPQSIAYSLLAEQDPIYGLYTSFFASII 2177160

Query 121   YALLGTSRHISVGMFGVLCLLVGQVVDRELTLAGYTSDTNQTIMGNLDNSTGLICDRSCY 180
                YALLGTSRHISVGMFGVLCLLVGQVVDRELTLAGYTSDTNQTIMGNLDNSTGLICDRSCY
Sbjct 2177161 YALLGTSRHISVGMFGVLCLLVGQVVDRELTLAGYTSDTNQTIMGNLDNSTGLICDRSCY 2177340

Query 181   AIMVGATLTFTAGVYQV 197
                AIMVGATLTFTAGVYQV
Sbjct 2177341 AIMVGATLTFTAGVYQV 2177391

DJ Electric eel *slc26a2*

Query: sulfate transporter [Electrophorus electricus] Query ID: XP_026859357.2 Length: 701

>Electrophorus electricus isolate fEleEle1 chromosome 12, fEleEle1.pri, whole genome shotgun sequence
Sequence ID: NC_049546.1 Length: 23225763
Range 1: 6788554 to 6789324

Score:942 bits(1924), Expect:0.0,
Method:,
Identities:257/257(100%), Positives:257/257(100%), Gaps:0/257(0%)

Query 445   CVLAVIIMVNLRGALRKFCDVPRMWRANRVDAGVWLLTMATSALVNTELGLLLGVLASAF 504
                CVLAVIIMVNLRGALRKFCDVPRMWRANRVDAGVWLLTMATSALVNTELGLLLGVLASAF
Sbjct 6789324 CVLAVIIMVNLRGALRKFCDVPRMWRANRVDAGVWLLTMATSALVNTELGLLLGVLASAF 6789145

Query 505   CVLARTQRAQTLQLGQSGDRDVFKELTAYKGLQTLPGVAAFRYEAPIYYANQTLFKKSLY 564
                CVLARTQRAQTLQLGQSGDRDVFKELTAYKGLQTLPGVAAFRYEAPIYYANQTLFKKSLY
Sbjct 6789144 CVLARTQRAQTLQLGQSGDRDVFKELTAYKGLQTLPGVAAFRYEAPIYYANQTLFKKSLY 6788965

Query 565   RNTGLDPVKERARRKRLERGKKQEEERKGAEQSPEASSGNFLPHALPFHTLIIDCGPVLF 624
                RNTGLDPVKERARRKRLERGKKQEEERKGAEQSPEASSGNFLPHALPFHTLIIDCGPVLF
Sbjct 6788964 RNTGLDPVKERARRKRLERGKKQEEERKGAEQSPEASSGNFLPHALPFHTLIIDCGPVLF 6788785

Query 625   LDTAGVAALKEVRKDFQEFGVQLLLAQCNTSTIDSLRRGGYYDPETGNVERIFHTVGDAV 684
                LDTAGVAALKEVRKDFQEFGVQLLLAQCNTSTIDSLRRGGYYDPETGNVERIFHTVGDAV
Sbjct 6788784 LDTAGVAALKEVRKDFQEFGVQLLLAQCNTSTIDSLRRGGYYDPETGNVERIFHTVGDAV 6788605

Query 685   RYGQSLLSQNGDCDTSF 701
                RYGQSLLSQNGDCDTSF
Sbjct 6788604 RYGQSLLSQNGDCDTSF 6788554

Range 2: 6789539 to 6790291

Score:917 bits(1872), Expect:0.0,
Method:,
Identities:250/251(99%), Positives:251/251(100%), Gaps:0/251(0%)

Query 196   YQVLMGLLQVGFVSVFLSDSLLSGFATGASLTILTSQLKYLLGLKLPRTQGWGSLIKTWI 255
                +QVLMGLLQVGFVSVFLSDSLLSGFATGASLTILTSQLKYLLGLKLPRTQGWGSLIKTWI
Sbjct 6790291 FQVLMGLLQVGFVSVFLSDSLLSGFATGASLTILTSQLKYLLGLKLPRTQGWGSLIKTWI 6790112

Query 256   GLLGSLDQTNMCDLITSVLCLLVLVPTKELNERFKSKLKAPIPFELFVVIATTLASHFGH 315
                GLLGSLDQTNMCDLITSVLCLLVLVPTKELNERFKSKLKAPIPFELFVVIATTLASHFGH
Sbjct 6790111 GLLGSLDQTNMCDLITSVLCLLVLVPTKELNERFKSKLKAPIPFELFVVIATTLASHFGH 6789932

Query 316   FKEQYGSEVSGHIPTGFMPPQLPAWSLIPRIALDAFSIAIVGFAITVSLSEMFAKKHGYV 375
                FKEQYGSEVSGHIPTGFMPPQLPAWSLIPRIALDAFSIAIVGFAITVSLSEMFAKKHGYV
Sbjct 6789931 FKEQYGSEVSGHIPTGFMPPQLPAWSLIPRIALDAFSIAIVGFAITVSLSEMFAKKHGYV 6789752

Query 376   VDPNQEMYAIGFCNIIPSFFRCFVTSAALTKTLVKESTGCQTQLSSLVTALVLLLILLVI 435
                VDPNQEMYAIGFCNIIPSFFRCFVTSAALTKTLVKESTGCQTQLSSLVTALVLLLILLVI
Sbjct 6789751 VDPNQEMYAIGFCNIIPSFFRCFVTSAALTKTLVKESTGCQTQLSSLVTALVLLLILLVI 6789572

Query 436   APFFYSLQKCV 446
                APFFYSLQKCV
Sbjct 6789571 APFFYSLQKCV 6789539

Range 3: 6791255 to 6791848

Score:725 bits(1480), Expect:0.0,
Method:,
Identities:198/198(100%), Positives:198/198(100%), Gaps:0/198(0%)

```
Query  1        MPGGGSNVAARAPGPEGDLHVPLILEERGKEEEPLKVVLGRKLKKRCSWSPARAAALLLD  60
                MPGGGSNVAARAPGPEGDLHVPLILEERGKEEEPLKVVLGRKLKKRCSWSPARAAALLLD
Sbjct  6791848  MPGGGSNVAARAPGPEGDLHVPLILEERGKEEEPLKVVLGRKLKKRCSWSPARAAALLLD  6791669

Query  61       SVPILKWLPQYRIKDWLLGDVMSGVIVGILLAPQSIAYSLLAAQDPIYGLYTSFFSCIIY  120
                SVPILKWLPQYRIKDWLLGDVMSGVIVGILLAPQSIAYSLLAAQDPIYGLYTSFFSCIIY
Sbjct  6791668  SVPILKWLPQYRIKDWLLGDVMSGVIVGILLAPQSIAYSLLAAQDPIYGLYTSFFSCIIY  6791489

Query  121      TFMGTSRHISVGIFGVLCLLVGQVVERELAVAGYVTDVQGNRTGLGSLENGTFPDCDRSC  180
                TFMGTSRHISVGIFGVLCLLVGQVVERELAVAGYVTDVQGNRTGLGSLENGTFPDCDRSC
Sbjct  6791488  TFMGTSRHISVGIFGVLCLLVGQVVERELAVAGYVTDVQGNRTGLGSLENGTFPDCDRSC  6791309

Query  181      YAIIVGSTVTFTAGVYQV  198
                YAIIVGSTVTFTAGVYQV
Sbjct  6791308  YAIIVGSTVTFTAGVYQV  6791255
```

DK Mexican tetra *slc26a2*

Query: sulfate transporter [Astyanax mexicanus] Query ID: XP_007246475.2 Length: 698

>Astyanax mexicanus isolate ESR-SI-001 chromosome 10, AstMex3_surface, whole genome shotgun sequence
Sequence ID: NC_064417.1 Length: 54663907
Range 1: 38524928 to 38525698

Score:945 bits(1929), Expect:0.0,
Method:,
Identities:257/257(100%), Positives:257/257(100%), Gaps:0/257(0%)

```
Query  442       CVLAVIIVVNLRGALRKFRDVPQMWRTNRVDATIWLVTMATSALVNTELGLLVGVLVSAF  501
                 CVLAVIIVVNLRGALRKFRDVPQMWRTNRVDATIWLVTMATSALVNTELGLLVGVLVSAF
Sbjct  38524928  CVLAVIIVVNLRGALRKFRDVPQMWRTNRVDATIWLVTMATSALVNTELGLLVGVLVSAF  38525107

Query  502       SVLGRTQRAQAFKLGQAGDYEIFEDLASYKGLHTHPGVAVFRYEAPIYYANQTLFKKSLY  561
                 SVLGRTQRAQAFKLGQAGDYEIFEDLASYKGLHTHPGVAVFRYEAPIYYANQTLFKKSLY
Sbjct  38525108  SVLGRTQRAQAFKLGQAGDYEIFEDLASYKGLHTHPGVAVFRYEAPIYYANQTLFKKSLY  38525287

Query  562       RNVGLDPVKEKARRKKLEKQNKQKTLEDGVKQDHEASTNMFLSQGPAFHTLIIDCSAVLF  621
                 RNVGLDPVKEKARRKKLEKQNKQKTLEDGVKQDHEASTNMFLSQGPAFHTLIIDCSAVLF
Sbjct  38525288  RNVGLDPVKEKARRKKLEKQNKQKTLEDGVKQDHEASTNMFLSQGPAFHTLIIDCSAVLF  38525467

Query  622       LDTAGVGALKEVHKDYKELGVRLLLAQCNTSVIDSLRRAGYYDHKTGSPENIFHTISDAV  681
                 LDTAGVGALKEVHKDYKELGVRLLLAQCNTSVIDSLRRAGYYDHKTGSPENIFHTISDAV
Sbjct  38525468  LDTAGVGALKEVHKDYKELGVRLLLAQCNTSVIDSLRRAGYYDHKTGSPENIFHTISDAV  38525647

Query  682       RYSQSLLSQNGDCDTFC  698
                 RYSQSLLSQNGDCDTFC
Sbjct  38525648  RYSQSLLSQNGDCDTFC  38525698
```

Range 2: 38522692 to 38523438

Score:906 bits(1849), Expect:0.0,
Method:,
Identities:248/249(99%), Positives:249/249(100%), Gaps:0/249(0%)

```
Query  193       YQVLMGLLQVGFVSVFLSDSLLSGFATGASLTILTSQLKYLLGLKLPRAQGWGSLIKTWI  252
                 +QVLMGLLQVGFVSVFLSDSLLSGFATGASLTILTSQLKYLLGLKLPRAQGWGSLIKTWI
Sbjct  38522692  FQVLMGLLQVGFVSVFLSDSLLSGFATGASLTILTSQLKYLLGLKLPRAQGWGSLIKTWI  38522871

Query  253       SLLTNLGQTNICDLITSLICLLVLIPTKELNDRFKSKLKAPIPFELFVVIIATLASHFGQ  312
                 SLLTNLGQTNICDLITSLICLLVLIPTKELNDRFKSKLKAPIPFELFVVIIATLASHFGQ
Sbjct  38522872  SLLTNLGQTNICDLITSLICLLVLIPTKELNDRFKSKLKAPIPFELFVVIIATLASHFGQ  38523051

Query  313       FQEKYGSGVAGAIPTGFMPPQLPDWSLIPNVAVDAFSIAIVGFAITVSLSEMFAKKHGYV  372
                 FQEKYGSGVAGAIPTGFMPPQLPDWSLIPNVAVDAFSIAIVGFAITVSLSEMFAKKHGYV
Sbjct  38523052  FQEKYGSGVAGAIPTGFMPPQLPDWSLIPNVAVDAFSIAIVGFAITVSLSEMFAKKHGYV  38523231

Query  373       VDPNQEMYAIGFCNIFPSFFRCFTTSAALTKTLVKESTGCQTQVSGLITALVLLLVLLVI  432
                 VDPNQEMYAIGFCNIFPSFFRCFTTSAALTKTLVKESTGCQTQVSGLITALVLLLVLLVI
Sbjct  38523232  VDPNQEMYAIGFCNIFPSFFRCFTTSAALTKTLVKESTGCQTQVSGLITALVLLLVLLVI  38523411

Query  433       APLFYSLQK  441
                 APLFYSLQK
Sbjct  38523412  APLFYSLQK  38523438
```

Range 3: 38521913 to 38522497

Score:717 bits(1464), Expect:0.0,
Method:,
Identities:195/195(100%), Positives:195/195(100%), Gaps:0/195(0%)

```
Query  1         MAAGEGNGAAAEAGPESDLHVPFILEERQREEEPLKKVLSQKLKEQCSCSPTRAKALLFD  60
                 MAAGEGNGAAAEAGPESDLHVPFILEERQREEEPLKKVLSQKLKEQCSCSPTRAKALLFD
Sbjct  38521913  MAAGEGNGAAAEAGPESDLHVPFILEERQREEEPLKKVLSQKLKEQCSCSPTRAKALLFD  38522092
```

```
Query  61        SIPILKWLPRYQIKDWIIGDMMSGVIVGILLVPQSIAYSLLAGQDPIYGLYTSFFSSIIY  120
                 SIPILKWLPRYQIKDWIIGDMMSGVIVGILLVPQSIAYSLLAGQDPIYGLYTSFFSSIIY
Sbjct  38522093  SIPILKWLPRYQIKDWIIGDMMSGVIVGILLVPQSIAYSLLAGQDPIYGLYTSFFSSIIY  38522272

Query  121       ALLGTSRHISVGIFGVLCLLVGQVVDRELAVAGYLTDLPSNMTSLENSTAPYCDRSCYAI  180
                 ALLGTSRHISVGIFGVLCLLVGQVVDRELAVAGYLTDLPSNMTSLENSTAPYCDRSCYAI
Sbjct  38522273  ALLGTSRHISVGIFGVLCLLVGQVVDRELAVAGYLTDLPSNMTSLENSTAPYCDRSCYAI  38522452

Query  181       IVGATVTFTAGVYQV  195
                 IVGATVTFTAGVYQV
Sbjct  38522453  IVGATVTFTAGVYQV  38522497
```

DL Channel catfish *slc26a2*

```
Query: sulfate transporter [Ictalurus punctatus] Query ID: XP_017330565.1 Length: 688


>Ictalurus punctatus breed USDA103 chromosome 8, Coco_2.0, whole genome shotgun sequence
Sequence ID: NC_030423.2 Length: 31480639
Range 1: 15070104 to 15070841

Score:913 bits(1864), Expect:0.0,
Method:,
Identities:246/246(100%), Positives:246/246(100%), Gaps:0/246(0%)

Query  443       CVLAVIIVVNLRGALRKFRDVPQMWRVNRIDAAVWLVTMATSALVNTELGLLVGVLVSAF  502
                 CVLAVIIVVNLRGALRKFRDVPQMWRVNRIDAAVWLVTMATSALVNTELGLLVGVLVSAF
Sbjct  15070841  CVLAVIIVVNLRGALRKFRDVPQMWRVNRIDAAVWLVTMATSALVNTELGLLVGVLVSAF  15070662

Query  503       CVLGRTQCAQAIELGQAGHRDLFKDMESYKNLHKHPGVAVFRYEAPIYYANQALFKKFLY  562
                 CVLGRTQCAQAIELGQAGHRDLFKDMESYKNLHKHPGVAVFRYEAPIYYANQALFKKFLY
Sbjct  15070661  CVLGRTQCAQAIELGQAGHRDLFKDMESYKNLHKHPGVAVFRYEAPIYYANQALFKKFLY  15070482

Query  563       RSVGLDPQKEKARLKKLEKKQKKMKEDAEVPTSGFLPSFHTLILDCSAVLFVDTAGVSAL  622
                 RSVGLDPQKEKARLKKLEKKQKKMKEDAEVPTSGFLPSFHTLILDCSAVLFVDTAGVSAL
Sbjct  15070481  RSVGLDPQKEKARLKKLEKKQKKMKEDAEVPTSGFLPSFHTLILDCSAVLFVDTAGVSAL  15070302

Query  623       KEMYKDYKEFAVHLLLAQCSPSVIESLRRGGYYDPESTEIIFHSLGDAVRYAQSCQSQNG  682
                 KEMYKDYKEFAVHLLLAQCSPSVIESLRRGGYYDPESTEIIFHSLGDAVRYAQSCQSQNG
Sbjct  15070301  KEMYKDYKEFAVHLLLAQCSPSVIESLRRGGYYDPESTEIIFHSLGDAVRYAQSCQSQNG  15070122

Query  683       DCETPC  688
                 DCETPC
Sbjct  15070121  DCETPC  15070104



Range 2: 15071376 to 15072122

Score:912 bits(1863), Expect:0.0,
Method:,
Identities:248/249(99%), Positives:249/249(100%), Gaps:0/249(0%)

Query  194       YQVLMGLLQVGFVSVFLSDSLLSGFATGASLTILTSQLKYLLGLKLPRAQGWGSLIKTWI  253
                 +QVLMGLLQVGFVSVFLSDSLLSGFATGASLTILTSQLKYLLGLKLPRAQGWGSLIKTWI
Sbjct  15072122  FQVLMGLLQVGFVSVFLSDSLLSGFATGASLTILTSQLKYLLGLKLPRAQGWGSLIKTWI  15071943

Query  254       RLFKNLGQTNMCDLITSILCLLVLVPAKELNDFFKSRLKAPIPFELFVVIIATLASHFGH  313
                 RLFKNLGQTNMCDLITSILCLLVLVPAKELNDFFKSRLKAPIPFELFVVIIATLASHFGH
Sbjct  15071942  RLFKNLGQTNMCDLITSILCLLVLVPAKELNDFFKSRLKAPIPFELFVVIIATLASHFGH  15071763

Query  314       FQEKYGSEVSGAIPTGFMPPQLPAWDLIPNIALDAFSIAIVGFAITVSLSEMFAQKHGYM  373
                 FQEKYGSEVSGAIPTGFMPPQLPAWDLIPNIALDAFSIAIVGFAITVSLSEMFAQKHGYM
Sbjct  15071762  FQEKYGSEVSGAIPTGFMPPQLPAWDLIPNIALDAFSIAIVGFAITVSLSEMFAQKHGYM  15071583

Query  374       VDANQEMYAIGFCNIFPSFFHCFTTSAALTKTLVKESTGCQTQLSSLVTALVLLLVLLVI  433
                 VDANQEMYAIGFCNIFPSFFHCFTTSAALTKTLVKESTGCQTQLSSLVTALVLLLVLLVI
Sbjct  15071582  VDANQEMYAIGFCNIFPSFFHCFTTSAALTKTLVKESTGCQTQLSSLVTALVLLLVLLVI  15071403

Query  434       APLFYSLQK  442
                 APLFYSLQK
Sbjct  15071402  APLFYSLQK  15071376



Range 3: 15073860 to 15074447

Score:721 bits(1471), Expect:0.0,
Method:,
Identities:196/196(100%), Positives:196/196(100%), Gaps:0/196(0%)

Query  1         MMSGVEAAQVAGEGESKNELQTPFILEECERKDQSLRVLLSQKLKKQCVCSPARAKALLL  60
                 MMSGVEAAQVAGEGESKNELQTPFILEECERKDQSLRVLLSQKLKKQCVCSPARAKALLL
Sbjct  15074447  MMSGVEAAQVAGEGESKNELQTPFILEECERKDQSLRVLLSQKLKKQCVCSPARAKALLL  15074268

Query  61        DSVPILKWLPKYQVKDWLLGDMMSGLIVGILLVPQSIAYSLLAGQDPIYGLYTSFFSSII  120
                 DSVPILKWLPKYQVKDWLLGDMMSGLIVGILLVPQSIAYSLLAGQDPIYGLYTSFFSSII
Sbjct  15074267  DSVPILKWLPKYQVKDWLLGDMMSGLIVGILLVPQSIAYSLLAGQDPIYGLYTSFFSSII  15074088
```

```
Query  121       YTLLGTSRHISVGIFGVLCLLVGQVVDRELTLAGYPTDQSGNITSLENNTIPQCDRSCYA  180
                 YTLLGTSRHISVGIFGVLCLLVGQVVDRELTLAGYPTDQSGNITSLENNTIPQCDRSCYA
Sbjct  15074087  YTLLGTSRHISVGIFGVLCLLVGQVVDRELTLAGYPTDQSGNITSLENNTIPQCDRSCYA  15073908

Query  181       IIIGATVTFTAGVYQV  196
                 IIIGATVTFTAGVYQV
Sbjct  15073907  IIIGATVTFTAGVYQV  15073860
```

DM Rainbow trout *slc26a2*

Query: sulfate transporter isoform X1 [Oncorhynchus mykiss] Query ID: XP_036826124.1 Length: 725

>Oncorhynchus mykiss isolate Arlee chromosome 31, USDA_OmykA_1.1, whole genome shotgun sequence
Sequence ID: NC_050571.1 Length: 44108611
Range 1: 22965453 to 22966253

Score:986 bits(2013), Expect:0.0,
Method:,
Identities:267/267(100%), Positives:267/267(100%), Gaps:0/267(0%)

```
Query  459       RCVLAVIILVNLRGALLKFTDVPRMWRVNRLDTAIWLVTMATSALINTELGLLVGVMVSA  518
                 RCVLAVIILVNLRGALLKFTDVPRMWRVNRLDTAIWLVTMATSALINTELGLLVGVMVSA
Sbjct  22965453  RCVLAVIILVNLRGALLKFTDVPRMWRVNRLDTAIWLVTMATSALINTELGLLVGVMVSA  22965632

Query  519       FCVLGRTQRAQALELGRAGHRELYQDLASYNGLQSQPGVAIFRYEAPIYYANQSLFKKAL  578
                 FCVLGRTQRAQALELGRAGHRELYQDLASYNGLQSQPGVAIFRYEAPIYYANQSLFKKAL
Sbjct  22965633  FCVLGRTQRAQALELGRAGHRELYQDLASYNGLQSQPGVAIFRYEAPIYYANQSLFKKAL  22965812

Query  579       YRCLGLDPVKEKARRRKLEKHRRKQEEVAMMTGGEGGTKGKEKELTTKVFLPNQVNFHSV  638
                 YRCLGLDPVKEKARRRKLEKHRRKQEEVAMMTGGEGGTKGKEKELTTKVFLPNQVNFHSV
Sbjct  22965813  YRCLGLDPVKEKARRRKLEKHRRKQEEVAMMTGGEGGTKGKEKELTTKVFLPNQVNFHSV  22965992

Query  639       VIDCSPVLFLDTAGVNALKEVYKDYKENGVQVFLAQCNTSVLESLNRGGYYPEKGMGEKE  698
                 VIDCSPVLFLDTAGVNALKEVYKDYKENGVQVFLAQCNTSVLESLNRGGYYPEKGMGEKE
Sbjct  22965993  VIDCSPVLFLDTAGVNALKEVYKDYKENGVQVFLAQCNTSVLESLNRGGYYPEKGMGEKE  22966172

Query  699       RVFFTISDAVLYAQSLSSQNGDCDTSC  725
                 RVFFTISDAVLYAQSLSSQNGDCDTSC
Sbjct  22966173  RVFFTISDAVLYAQSLSSQNGDCDTSC  22966253
```

Range 2: 22964549 to 22965289

Score:900 bits(1838), Expect:0.0,
Method:,
Identities:247/247(100%), Positives:247/247(100%), Gaps:0/247(0%)

```
Query  213       VLMGLLQVGFVSVYLSDSLLSGFATGASLTILTSQVKYLLGLELPRAQGWGSLVKTWVSL  272
                 VLMGLLQVGFVSVYLSDSLLSGFATGASLTILTSQVKYLLGLELPRAQGWGSLVKTWVSL
Sbjct  22964549  VLMGLLQVGFVSVYLSDSLLSGFATGASLTILTSQVKYLLGLELPRAQGWGSLVKTWVSL  22964728

Query  273       FQNLGQTNLCDLVTSMVCLAVLVPTKELNDRFKAKLKAPIPFELFVVIAATLASHFGHFE  332
                 FQNLGQTNLCDLVTSMVCLAVLVPTKELNDRFKAKLKAPIPFELFVVIAATLASHFGHFE
Sbjct  22964729  FQNLGQTNLCDLVTSMVCLAVLVPTKELNDRFKAKLKAPIPFELFVVIAATLASHFGHFE  22964908

Query  333       QEYGSKVAGAIPTGFLPPQLPSWSLIPNVAVDAFSIAIVGFAITVSLSEMFAKKHGYVVD  392
                 QEYGSKVAGAIPTGFLPPQLPSWSLIPNVAVDAFSIAIVGFAITVSLSEMFAKKHGYVVD
Sbjct  22964909  QEYGSKVAGAIPTGFLPPQLPSWSLIPNVAVDAFSIAIVGFAITVSLSEMFAKKHGYVVD  22965088

Query  393       PNQEMYAIGFCNILPSFFRCFTTSAALTKTLVKESTGCQTQMSGLVTALVLLLVLLVISP  452
                 PNQEMYAIGFCNILPSFFRCFTTSAALTKTLVKESTGCQTQMSGLVTALVLLLVLLVISP
Sbjct  22965089  PNQEMYAIGFCNILPSFFRCFTTSAALTKTLVKESTGCQTQMSGLVTALVLLLVLLVISP  22965268

Query  453       LFYSLQR  459
                 LFYSLQR
Sbjct  22965269  LFYSLQR  22965289
```

Range 3: 22963483 to 22964100

Score:761 bits(1553), Expect:0.0,
Method:,
Identities:206/206(100%), Positives:206/206(100%), Gaps:0/206(0%)

```
Query  8         SEGGPAMMAQEYCCASADEGAESGPHVPFVLERREKEEENWRTAVSHQLKKQCTCSSERA  67
                 SEGGPAMMAQEYCCASADEGAESGPHVPFVLERREKEEENWRTAVSHQLKKQCTCSSERA
Sbjct  22963483  SEGGPAMMAQEYCCASADEGAESGPHVPFVLERREKEEENWRTAVSHQLKKQCTCSSERA  22963662

Query  68        KAQVLGFVPILKWLPRYQLKDWILGDVMSGLIVGILLVPQSIAYSLLAGQDPIYGLYTSF  127
                 KAQVLGFVPILKWLPRYQLKDWILGDVMSGLIVGILLVPQSIAYSLLAGQDPIYGLYTSF
Sbjct  22963663  KAQVLGFVPILKWLPRYQLKDWILGDVMSGLIVGILLVPQSIAYSLLAGQDPIYGLYTSF  22963842

Query  128       FSSIIYTLLGTSRHISVGIFGVLCLLVGQVVDRELALAGYITESIRNNATLGLGMGNSTG  187
                 FSSIIYTLLGTSRHISVGIFGVLCLLVGQVVDRELALAGYITESIRNNATLGLGMGNSTG
Sbjct  22963843  FSSIIYTLLGTSRHISVGIFGVLCLLVGQVVDRELALAGYITESIRNNATLGLGMGNSTG  22964022
```

```
Query  188       GPVCDRSCYAIMVGTTVTFTAGVYQV  213
                 GPVCDRSCYAIMVGTTVTFTAGVYQV
Sbjct  22964023  GPVCDRSCYAIMVGTTVTFTAGVYQV  22964100
```

DN Argentina silus *slc26a2*

```
>Argentina silus genome assembly, contig: atg000157l_1, whole genome shotgun sequence
Sequence ID: CATOGY010000148.1 Length: 667433
Range 1: 569272 to 570078

Score:494 bits(1272), Expect:2e-152,
Method:Compositional matrix adjust.,
Identities:268/269(99%), Positives:269/269(100%), Gaps:0/269(0%)

Query  444     KCVLAVIIVVNLRGALRKFMDVPRMWRVNHVDAFIWLVTMATSALVNTELGLLVGVLVSA  503
               +CVLAVIIVVNLRGALRKFMDVPRMWRVNHVDAFIWLVTMATSALVNTELGLLVGVLVSA
Sbjct  570078  RCVLAVIIVVNLRGALRKFMDVPRMWRVNHVDAFIWLVTMATSALVNTELGLLVGVLVSA  569899

Query  504     FCVLGRTQRAQALELGRATGHRDLYEDLASYKGLQTQPGVAIFRYEAPIYYANQTLFKKA  563
               FCVLGRTQRAQALELGRATGHRDLYEDLASYKGLQTQPGVAIFRYEAPIYYANQTLFKKA
Sbjct  569898  FCVLGRTQRAQALELGRATGHRDLYEDLASYKGLQTQPGVAIFRYEAPIYYANQTLFKKA  569719

Query  564     LYRCLGLDPVkekaqrkklekqrrkqeVAEgtgsgsgvggtQSKEQEVTTKVFLPNKVNF  623
               LYRCLGLDPVKEKAQRKKLEKQRRKQEVAEGTGSGSGVGGTQSKEQEVTTKVFLPNKVNF
Sbjct  569718  LYRCLGLDPVKEKAQRKKLEKQRRKQEVAEGTGSGSGVGGTQSKEQEVTTKVFLPNKVNF  569539

Query  624     HTLVIDCSPILFLDTAGVNALKEVHKDYKELGVQVLLAQCNTSVLECLQRGGYYPEKEGE  683
               HTLVIDCSPILFLDTAGVNALKEVHKDYKELGVQVLLAQCNTSVLECLQRGGYYPEKEGE
Sbjct  569538  HTLVIDCSPILFLDTAGVNALKEVHKDYKELGVQVLLAQCNTSVLECLQRGGYYPEKEGE  569359

Query  684     RERVFFTIGDAVQYAQNLSSQNGDCDTFC  712
               RERVFFTIGDAVQYAQNLSSQNGDCDTFC
Sbjct  569358  RERVFFTIGDAVQYAQNLSSQNGDCDTFC  569272



Range 2: 570295 to 571092

Score:473 bits(1216), Expect:5e-145,
Method:Compositional matrix adjust.,
Identities:254/276(92%), Positives:258/276(93%), Gaps:10/276(3%)

Query  197     QVLMGLLQVGFVSVYLSDSLLSGFATGASLTILTSQLKYLLGLKLPRAQGWGTLIKTWVS  256
               QVLMGLLQVGFVSVYLSDSLLSGFATGASLTILTSQLKYLLGLKLPRAQGWGTLIKTWVS
Sbjct  571092  QVLMGLLQVGFVSVYLSDSLLSGFATGASLTILTSQLKYLLGLKLPRAQGWGTLIKTWVS  570913

Query  257     LFQNLPNTNLCDLVTSLICLLVLVPTKELNDRFKAKLKAPIPFELFVVIGATLASHFGHF  316
               LFQNLPNTNLCDLVTSLICLLVLVPTKELNDRFKAKLKAPIPFELFVVIGATLASHFGHF
Sbjct  570912  LFQNLPNTNLCDLVTSLICLLVLVPTKELNDRFKAKLKAPIPFELFVVIGATLASHFGHF  570733

Query  317     KEVYSSEVAGAIPTGFLPPQLPSWSLIPNVAVDAFSIAIVGFAITVSLSEMFAKKHGYVV  376
               KEVYSSEVAGAIPTGFLPPQLPSWSLIPNVAVDAFSIAIVGFAITVSLSEMFAKKHGYVV
Sbjct  570732  KEVYSSEVAGAIPTGFLPPQLPSWSLIPNVAVDAFSIAIVGFAITVSLSEMFAKKHGYVV  570553

Query  377     DPNQEMYAIGFCNILPSFFRCFTTSAALTKTLVKESTGCQTQLSGlvtalvlllvllvIA  436
               DPNQEMYAIGFCNILPSFFRCFTTSAALTKTLVKESTGCQTQLSGLVTALVLLLVLLVIA
Sbjct  570552  DPNQEMYAIGFCNILPSFFRCFTTSAALTKTLVKESTGCQTQLSGLVTALVLLLVLLVIA  570373

Query  437     PLFYSLQKCVLAVIIVVNLRGALRKFMDVPRMWRVN  472
               PLFYSLQK V          G L   +++ + W VN
Sbjct  570372  PLFYSLQK*V----------GGLLY*LNIEKGWLVN  570295
```

DO Large-eye snaggletooth *slc26a2*

```
Query: unnamed protein product Query ID: lcl|Query_8160881 Length: 713


>Borostomias antarcticus genome assembly, contig: atg000317l_1, whole genome shotgun sequence
Sequence ID: CATLJU010000294.1 Length: 765169
Range 1: 628065 to 628838

Score:935 bits(1909), Expect:0.0,
Method:,
Identities:258/258(100%), Positives:258/258(100%), Gaps:0/258(0%)

Query  456     CVLAVIIIVNLRGALRKFADVPQMWRVNRVDACVWLVTAATSALVNTELGLLVGVLVAAF  515
               CVLAVIIIVNLRGALRKFADVPQMWRVNRVDACVWLVTAATSALVNTELGLLVGVLVAAF
Sbjct  628838  CVLAVIIIVNLRGALRKFADVPQMWRVNRVDACVWLVTAATSALVNTELGLLVGVLVAAF  628659

Query  516     CVLARTQRARGSALGRAGTRDLYQDLARYRGLHTQPGVAVFRYEAPIYYANQSLFKKCLY  575
               CVLARTQRARGSALGRAGTRDLYQDLARYRGLHTQPGVAVFRYEAPIYYANQSLFKKCLY
Sbjct  628658  CVLARTQRARGSALGRAGTRDLYQDLARYRGLHTQPGVAVFRYEAPIYYANQSLFKKCLY  628479

Query  576     LRLGLDPVKEKACRRKLETRKRKQEAETTSGEGGAEGRAKATAAAEPAKVSLAVKASFHS  635
```

```
               LRLGLDPVKEKACRRKLETRKRKQEAETTSGEGGAEGRAKATAAAEPAKVSLAVKASFHS
Sbjct  628478  LRLGLDPVKEKACRRKLETRKRKQEAETTSGEGGAEGRAKATAAAEPAKVSLAVKASFHS  628299

Query  636     VVIDCSPVLFVDTAGVNALKEVRKDYQDLGLQVLLAQCNESVLESLRRGGYYPEKGGEGE  695
               VVIDCSPVLFVDTAGVNALKEVRKDYQDLGLQVLLAQCNESVLESLRRGGYYPEKGGEGE
Sbjct  628298  VVIDCSPVLFVDTAGVNALKEVRKDYQDLGLQVLLAQCNESVLESLRRGGYYPEKGGEGE  628119

Query  696     QLFFTITDAVHYALKNFS  713
               QLFFTITDAVHYALKNFS
Sbjct  628118  QLFFTITDAVHYALKNFS  628065
```

```
Range 2: 630530 to 631156

Score:760 bits(1551), Expect:0.0,
Method:,
Identities:209/209(100%), Positives:209/209(100%), Gaps:0/209(0%)

Query  1       MAGEEDCCSSASEGVESAPPLPVVLERRETPGEPWRTALARRMRRSCSCSPERAREAVLG  60
               MAGEEDCCSSASEGVESAPPLPVVLERRETPGEPWRTALARRMRRSCSCSPERAREAVLG
Sbjct  631156  MAGEEDCCSSASEGVESAPPLPVVLERRETPGEPWRTALARRMRRSCSCSPERAREAVLG  630977

Query  61      FLPILKWLPHYRLRDWLLGDVMSGLIVGVLLVPQSIAYSLLAGQEPIYGLYTSFFSSIIY  120
               FLPILKWLPHYRLRDWLLGDVMSGLIVGVLLVPQSIAYSLLAGQEPIYGLYTSFFSSIIY
Sbjct  630976  FLPILKWLPHYRLRDWLLGDVMSGLIVGVLLVPQSIAYSLLAGQEPIYGLYTSFFSSIIY  630797

Query  121     ALLGTSRHISVGIFGVLCLLVGQVVDREMAAAGFLSEEGGGGGNDTAAAVLLASLGNNDS  180
               ALLGTSRHISVGIFGVLCLLVGQVVDREMAAAGFLSEEGGGGGNDTAAAVLLASLGNNDS
Sbjct  630796  ALLGTSRHISVGIFGVLCLLVGQVVDREMAAAGFLSEEGGGGGNDTAAAVLLASLGNNDS  630617

Query  181     VGAGPVCDKSCYAIVVAATVTFTAGVYQV  209
               VGAGPVCDKSCYAIVVAATVTFTAGVYQV
Sbjct  630616  VGAGPVCDKSCYAIVVAATVTFTAGVYQV  630530
```

```
Range 3: 630143 to 630430

Score:347 bits(707), Expect:0.0,
Method:,
Identities:96/96(100%), Positives:96/96(100%), Gaps:0/96(0%)

Query  208     QVLMGLLQVGFLSVYLSDSLLSGFATGASLTILTSQLKYLLGLKLPRAQGWFTLGKTWLS  267
               QVLMGLLQVGFLSVYLSDSLLSGFATGASLTILTSQLKYLLGLKLPRAQGWFTLGKTWLS
Sbjct  630430  QVLMGLLQVGFLSVYLSDSLLSGFATGASLTILTSQLKYLLGLKLPRAQGWFTLGKTWLS  630251

Query  268     LLQNLAHANPCDLVTSLLCLLVLVPAKELNERFKAR  303
               LLQNLAHANPCDLVTSLLCLLVLVPAKELNERFKAR
Sbjct  630250  LLQNLAHANPCDLVTSLLCLLVLVPAKELNERFKAR  630143
```

```
Range 4: 629128 to 629586

Score:556 bits(1134), Expect:7e-157,
Method:,
Identities:153/153(100%), Positives:153/153(100%), Gaps:0/153(0%)

Query  303     RLKAPVPFELFVVIAATLASHFGRFQEVYGSEVAGAIPTGFLPPQLPSWSLIPSVAVDAF  362
               RLKAPVPFELFVVIAATLASHFGRFQEVYGSEVAGAIPTGFLPPQLPSWSLIPSVAVDAF
Sbjct  629586  RLKAPVPFELFVVIAATLASHFGRFQEVYGSEVAGAIPTGFLPPQLPSWSLIPSVAVDAF  629407

Query  363     SIAVVGFAITVSLSEMFAKKHGYQVDANQEMYAIGFCNILPSFFRCFTTSAALTKTLVKE  422
               SIAVVGFAITVSLSEMFAKKHGYQVDANQEMYAIGFCNILPSFFRCFTTSAALTKTLVKE
Sbjct  629406  SIAVVGFAITVSLSEMFAKKHGYQVDANQEMYAIGFCNILPSFFRCFTTSAALTKTLVKE  629227

Query  423     STGCQTQLSGLVTALVLLLVLLLIAPLFYSLQK  455
               STGCQTQLSGLVTALVLLLVLLLIAPLFYSLQK
Sbjct  629226  STGCQTQLSGLVTALVLLLVLLLIAPLFYSLQK  629128
```

DP European smelt *slc26a2*

```
Query: sulfate transporter [Osmerus eperlanus] ID: XP_062332156.1(amino acid) Length: 724


>Osmerus eperlanus genome assembly, contig: scf7180003263726, whole genome shotgun sequence
Sequence ID: OMKJ01040172.1 Length: 46262
Range 1: 33235 to 34023

Score:551 bits(1420), Expect:5e-172,
Method:Compositional matrix adjust.,
Identities:262/263(99%), Positives:263/263(100%), Gaps:0/263(0%)

Query  462    KCVLAVIIIVNLSGALRKFTEIPQMWHVNRVDASIWLITAATSALINTELGLLVGVLVSA  521
              +CVLAVIIIVNLSGALRKFTEIPQMWHVNRVDASIWLITAATSALINTELGLLVGVLVSA
Sbjct  33235  RCVLAVIIIVNLSGALRKFTEIPQMWHVNRVDASIWLITAATSALINTELGLLVGVLVSA  33414
```

```
Query  522    FCVLVRTQRAQGLELGRVGTSELYEDLAGYHGIQSQPGIAIFRYESPIYYANQSLFKKCL  581
              FCVLVRTQRAQGLELGRVGTSELYEDLAGYHGIQSQPGIAIFRYESPIYYANQSLFKKCL
Sbjct  33415  FCVLVRTQRAQGLELGRVGTSELYEDLAGYHGIQSQPGIAIFRYESPIYYANQSLFKKCL  33594

Query  582    YHRLGLDPVKEKVHRKKLEKNRKREGQMRAGENRTKGQEAEMTTNVFLPVKSSFHSVVID  641
              YHRLGLDPVKEKVHRKKLEKNRKREGQMRAGENRTKGQEAEMTTNVFLPVKSSFHSVVID
Sbjct  33595  YHRLGLDPVKEKVHRKKLEKNRKREGQMRAGENRTKGQEAEMTTNVFLPVKSSFHSVVID  33774

Query  642    CSPVLFLDTAGVNSLKEVYEDYKELGVKVLLAQCNFSVRASLQRGGYYPDNDGEREHVFF  701
              CSPVLFLDTAGVNSLKEVYEDYKELGVKVLLAQCNFSVRASLQRGGYYPDNDGEREHVFF
Sbjct  33775  CSPVLFLDTAGVNSLKEVYEDYKELGVKVLLAQCNFSVRASLQRGGYYPDNDGEREHVFF  33954

Query  702    TISDAVHYAQCLSGENGACDPHC  724
              TISDAVHYAQCLSGENGACDPHC
Sbjct  33955  TISDAVHYAQCLSGENGACDPHC  34023
```

Range 2: 31361 to 32392

Score:531 bits(1369), Expect:4e-165,
Method:Compositional matrix adjust.,
Identities:305/344(89%), Positives:306/344(88%), Gaps:38/344(11%)

```
Query  5      SEYMMATLRPAVDENDSYSVAKDSDLQLAIVLERREKTEEPWNTVFARRLQKHCFCSSAQ  64
              +EYMMATLRPAVDENDSYSVAKDSDLQLAIVLERREKTEEPWNTVFARRLQKHCFCSSAQ
Sbjct  31361  AEYMMATLRPAVDENDSYSVAKDSDLQLAIVLERREKTEEPWNTVFARRLQKHCFCSSAQ  31540

Query  65     IAKASVLRFIPILQWLPRYHLKEWLLGDIMSGLIVGILLVPQSIAYSLLAGQEPIYGLYT  124
              IAKASVLRFIPILQWLPRYHLKEWLLGDIMSGLIVGILLVPQSIAYSLLAGQEPIYGLYT
Sbjct  31541  IAKASVLRFIPILQWLPRYHLKEWLLGDIMSGLIVGILLVPQSIAYSLLAGQEPIYGLYT  31720

Query  125    sffssiiysfLGTSRHVSVGIFGVLCLLVGQVVDREVAAAGYITEGYSNKTTSFILGRLG  184
              SFFSSIIYSFLGTSRHVSVGIFGVLCLLVGQVVDREVAAAGYITEGYSNKTTSFILGRLG
Sbjct  31721  SFFSSIIYSFLGTSRHVSVGIFGVLCLLVGQVVDREVAAAGYITEGYSNKTTSFILGRLG  31900

Query  185    NDTAGAGLVCDKSCYAIMVGATVTFTAGVYQ-----------------------------  215
              NDTAGAGLVCDKSCYAIMVGATVTFTAGVYQ
Sbjct  31901  NDTAGAGLVCDKSCYAIMVGATVTFTAGVYQVRLWPRVNKPQSSGMITKYPQILYR*LPL  32080

Query  216    ---------VMMGLLQVGFVSVYLSDSLLSGFATGASLTILTSQLKYLLGLKLPRAQGWF  266
                       VMMGLLQVGFVSVYLSDSLLSGFATGASLTILTSQLKYLLGLKLPRAQGWF
Sbjct  32081  PLLPSCPFKVMMGLLQVGFVSVYLSDSLLSGFATGASLTILTSQLKYLLGLKLPRAQGWF  32260

Query  267    TLIKTWFSLLQNMADTNlcdlftsllcllvlvptkelNERFKTR  310
              TLIKTWFSLLQNMADTNLCDLFTSLLCLLVLVPTKELNERFKTR
Sbjct  32261  TLIKTWFSLLQNMADTNLCDLFTSLLCLLVLVPTKELNERFKTR  32392
```

Range 3: 32532 to 33041

Score:281 bits(720), Expect:2e-79,
Method:Compositional matrix adjust.,
Identities:157/170(92%), Positives:163/170(95%), Gaps:5/170(2%)

```
Query  310    RLKAPIPWELFVVIAATVASHFGCFQEVYGSDVAGTIPTGFLPPQMPSWSLIPAVAVDAF  369
              RLKAPIPWELFVVIAATVASHFGCFQEVYGSDVAGTIPTGFLPPQMPSWSLIPAVAVDAF
Sbjct  32532  RLKAPIPWELFVVIAATVASHFGCFQEVYGSDVAGTIPTGFLPPQMPSWSLIPAVAVDAF  32711

Query  370    SIAVVGFAITVSLSEMFAKKHGYQVDPNQEMYAIGFCNILPSFFRCFTTSAALTKTLVKE  429
              SIAVVGFAITVSLSEMFAKKHGYQVDPNQEMYAIGFCNILPSFFRCFTTSAALTKTLVKE
Sbjct  32712  SIAVVGFAITVSLSEMFAKKHGYQVDPNQEMYAIGFCNILPSFFRCFTTSAALTKTLVKE  32891

Query  430    STGCKTQlsglvtalvlllvlllIAPLFYSLQK-CV----LAVIIIVNLS  474
              STGCKTQLSGLVTALVLLLVLLLIAPLFYSLQK C+    L + ++++LS
Sbjct  32892  STGCKTQLSGLVTALVLLLVLLLIAPLFYSLQK*CIHFFQLLICLLIDLS  33041
```

Range 4: 4307370 to 4307828

Score:563 bits(1149), Expect:4e-165,
Method:,
Identities:153/153(100%), Positives:153/153(100%), Gaps:0/153(0%)

```
Query  303      RLKAPIPWELFVVIAATVASHFGCFQEVYGSDVAGTIPTGFLPPQMPSWSLIPAVAIDAL  362
                RLKAPIPWELFVVIAATVASHFGCFQEVYGSDVAGTIPTGFLPPQMPSWSLIPAVAIDAL
Sbjct  4307828  RLKAPIPWELFVVIAATVASHFGCFQEVYGSDVAGTIPTGFLPPQMPSWSLIPAVAIDAL  4307649

Query  363      PIAIVGFAITVSLSEMFAKKHGYQVDPNQEMYAIGFCNILPSFFRCFTTSAALTKTLVKE  422
                PIAIVGFAITVSLSEMFAKKHGYQVDPNQEMYAIGFCNILPSFFRCFTTSAALTKTLVKE
Sbjct  4307648  PIAIVGFAITVSLSEMFAKKHGYQVDPNQEMYAIGFCNILPSFFRCFTTSAALTKTLVKE  4307469

Query  423      STGCKTQLSGLVTALVLLLVLLLIAPLFYSLQK  455
                STGCKTQLSGLVTALVLLLVLLLIAPLFYSLQK
Sbjct  4307468  STGCKTQLSGLVTALVLLLVLLLIAPLFYSLQK  4307370
```

DQ Ayu *slc26a2*

Query: unnamed protein product Query ID: lcl|Query_976675 Length: 709

>Plecoglossus altivelis isolate Fa20170821-1 scaf41_2, whole genome shotgun sequence
Sequence ID: SDAO01004358.1 Length: 6214464
Range 1: 1270792 to 1272132

Score:1614 bits(3298), Expect:0.0,
Method:,
Identities:446/447(99%), Positives:446/447(99%), Gaps:0/447(0%)

```
Query  263      ITSAICIFVLVAGKELQERYKDRMKIPLPTELVVVAGATLASHFGDLNGRYDSSVSGHIP  322
                I SAICIFVLVAGKELQERYKDRMKIPLPTELVVVAGATLASHFGDLNGRYDSSVSGHIP
Sbjct  1270792  IPSAICIFVLVAGKELQERYKDRMKIPLPTELVVVAGATLASHFGDLNGRYDSSVSGHIP  1270971

Query  323      TGFIAPTVPNFSLMPRVALDAIPLAVISFAFTVSLSEMFAKKNGYTVRPNQEMLAIGCCN  382
                TGFIAPTVPNFSLMPRVALDAIPLAVISFAFTVSLSEMFAKKNGYTVRPNQEMLAIGCCN
Sbjct  1270972  TGFIAPTVPNFSLMPRVALDAIPLAVISFAFTVSLSEMFAKKNGYTVRPNQEMLAIGCCN  1271151

Query  383      IIPSFFHCFTTSAALAKTMVKDSTGCQTQVSSLISALVVLLVLLFFAPYFHALQKCVLAC  442
                IIPSFFHCFTTSAALAKTMVKDSTGCQTQVSSLISALVVLLVLLFFAPYFHALQKCVLAC
Sbjct  1271152  IIPSFFHCFTTSAALAKTMVKDSTGCQTQVSSLISALVVLLVLLFFAPYFHALQKCVLAC  1271331

Query  443      IIIVSLRGALRKFRDVPSKWRASKTDAVVWLVTMASSALITVELGLLVGVVFSMICVIFQ  502
                IIIVSLRGALRKFRDVPSKWRASKTDAVVWLVTMASSALITVELGLLVGVVFSMICVIFQ
Sbjct  1271332  IIIVSLRGALRKFRDVPSKWRASKTDAVVWLVTMASSALITVELGLLVGVVFSMICVIFQ  1271511

Query  503      TQNPKVSLLGQAGSSELYEDIAEYKDLSAPSAIHIFRFQAPLYYANKESFLKSLYKAVGL  562
                TQNPKVSLLGQAGSSELYEDIAEYKDLSAPSAIHIFRFQAPLYYANKESFLKSLYKAVGL
Sbjct  1271512  TQNPKVSLLGQAGSSELYEDIAEYKDLSAPSAIHIFRFQAPLYYANKESFLKSLYKAVGL  1271691

Query  563      EPFLELTRRRKAQKKSLLAAKQAAASRGDKTNGEAIVGLVNAEPPFHTIVLDCSSVPFID  622
                EPFLELTRRRKAQKKSLLAAKQAAASRGDKTNGEAIVGLVNAEPPFHTIVLDCSSVPFID
Sbjct  1271692  EPFLELTRRRKAQKKSLLAAKQAAASRGDKTNGEAIVGLVNAEPPFHTIVLDCSSVPFID  1271871

Query  623      SAGVGTLKAVLKEYLEVGVSVLLAGCNTNIIDSLKRGSYFGKNDKDAQSLLFHTVHSAVL  682
                SAGVGTLKAVLKEYLEVGVSVLLAGCNTNIIDSLKRGSYFGKNDKDAQSLLFHTVHSAVL
Sbjct  1271872  SAGVGTLKAVLKEYLEVGVSVLLAGCNTNIIDSLKRGSYFGKNDKDAQSLLFHTVHSAVL  1272051

Query  683      YANKVANAADRAAGNTSPGEMAEDSVV  709
                YANKVANAADRAAGNTSPGEMAEDSVV
Sbjct  1272052  YANKVANAADRAAGNTSPGEMAEDSVV  1272132
```

Range 2: 1269741 to 1270316

Score:705 bits(1439), Expect:0.0,
Method:,
Identities:191/192(99%), Positives:191/192(99%), Gaps:0/192(0%)

```
Query  1        METVNTPGSCVPSLERRARQRKPPLEVLQTKVKRGLTCSVPRVRSTLTGFFPVVRWLPRY  60
                METVNTPGSCVPSLERRARQRKPPLEVLQTKVKRGLTCSVPRVRSTLTGFFPVVRWLPRY
Sbjct  1269741  METVNTPGSCVPSLERRARQRKPPLEVLQTKVKRGLTCSVPRVRSTLTGFFPVVRWLPRY  1269920

Query  61       KLQEYAWGDIMSGLIVGIILVPQAIAYCLLAGVDPIYGLYTSFFANIIYFFMGTSKHVSV  120
                KLQEYAWGDIMSGLIVGIILVPQAIAYCLLAGVDPIYGLYTSFFANIIYFFMGTSKHVSV
Sbjct  1269921  KLQEYAWGDIMSGLIVGIILVPQAIAYCLLAGVDPIYGLYTSFFANIIYFFMGTSKHVSV  1270100

Query  121      GIFSLMSLMVGQVVDREVFLAGYDLNDDVASTVLNDSLLVNGTAGVLKGEKEYYAISVAA  180
                GIFSLMSLMVGQVVDREVFLAGYDLNDDVAS VLNDSLLVNGTAGVLKGEKEYYAISVAA
Sbjct  1270101  GIFSLMSLMVGQVVDREVFLAGYDLNDDVASAVLNDSLLVNGTAGVLKGEKEYYAISVAA  1270280

Query  181      AVTFLAGIYQVL  192
                AVTFLAGIYQVL
Sbjct  1270281  AVTFLAGIYQVL  1270316
```

Range 3: 1270473 to 1270697

Score:280 bits(569), Expect:2e-73,
Method:,
Identities:75/75(100%), Positives:75/75(100%), Gaps:0/75(0%)

```
Query  190      QVLMAVFRLGFVSVFLSAPMLDGFATGASFTILTVQAKYLLGLKIPRYQGYGTVVVTWVN  249
                QVLMAVFRLGFVSVFLSAPMLDGFATGASFTILTVQAKYLLGLKIPRYQGYGTVVVTWVN
Sbjct  1270473  QVLMAVFRLGFVSVFLSAPMLDGFATGASFTILTVQAKYLLGLKIPRYQGYGTVVVTWVN  1270652

Query  250      IFSNIHHTNLCDLIT  264
                IFSNIHHTNLCDLIT
Sbjct  1270653  IFSNIHHTNLCDLIT  1270697
```

DR Peladilla *slc26a2*

Query: unnamed protein product Query ID: lcl|Query_127871 Length: 715

>Aplochiton taeniatus voucher 6995 isolate fAplTae1 001287F_arrow_arrow, whole genome shotgun sequence
Sequence ID: JAGFOW010004266.1 Length: 169766

```
Range 1: 38345 to 39217

Score:1060 bits(2165), Expect:0.0,
Method:,
Identities:291/291(100%), Positives:291/291(100%), Gaps:0/291(0%)

Query  425    QVSSLVSAVVVLLVLLFFAPFFYALQKCVLACIIIVSLRGALRKILDVPSKWRASRADAV  484
              QVSSLVSAVVVLLVLLFFAPFFYALQKCVLACIIIVSLRGALRKILDVPSKWRASRADAV
Sbjct  39217  QVSSLVSAVVVLLVLLFFAPFFYALQKCVLACIIIVSLRGALRKILDVPSKWRASRADAV  39038

Query  485    VWLVAMGATALISVELGLVVGVVFSMLCVIVQTQRPKVALLGRVADTDFYEDIKEYQNLT  544
              VWLVAMGATALISVELGLVVGVVFSMLCVIVQTQRPKVALLGRVADTDFYEDIKEYQNLT
Sbjct  39037  VWLVAMGATALISVELGLVVGVVFSMLCVIVQTQRPKVALLGRVADTDFYEDIKEYQNLT  38858

Query  545    APEGVQIFRFQAPLYYANKDSFLKALYKAVGLEPFLESTRRRKAEKKARELASKLPKSAG  604
              APEGVQIFRFQAPLYYANKDSFLKALYKAVGLEPFLESTRRRKAEKKARELASKLPKSAG
Sbjct  38857  APEGVQIFRFQAPLYYANKDSFLKALYKAVGLEPFLESTRRRKAEKKARELASKLPKSAG  38678

Query  605    ENNNSNGDVSTSLVVGENKFHTIILDCSAMPFMDSTGMGTFKGLVKDYSEVGVNLLLASC  664
              ENNNSNGDVSTSLVVGENKFHTIILDCSAMPFMDSTGMGTFKGLVKDYSEVGVNLLLASC
Sbjct  38677  ENNNSNGDVSTSLVVGENKFHTIILDCSAMPFMDSTGMGTFKGLVKDYSEVGVNLLLASC  38498

Query  665    NTNVIDTLRKGLFFGKGDKDMDSMLFYTIHAAVLYANSQVAPPALPADSAV  715
              NTNVIDTLRKGLFFGKGDKDMDSMLFYTIHAAVLYANSQVAPPALPADSAV
Sbjct  38497  NTNVIDTLRKGLFFGKGDKDMDSMLFYTIHAAVLYANSQVAPPALPADSAV  38345



Range 2: 39359 to 40027

Score:818 bits(1669), Expect:0.0,
Method:,
Identities:223/223(100%), Positives:223/223(100%), Gaps:0/223(0%)

Query  204    QVLMAVFRLGFVSVYLSAPMLDGFATGASFTILTVQAKYLLGLKIARHQGYGTVVVTWVR  263
              QVLMAVFRLGFVSVYLSAPMLDGFATGASFTILTVQAKYLLGLKIARHQGYGTVVVTWVR
Sbjct  40027  QVLMAVFRLGFVSVYLSAPMLDGFATGASFTILTVQAKYLLGLKIARHQGYGTVVVTWVR  39848

Query  264    ILSNIHHTNLCDLVTSAICIFVLVAGKELQERFKDRLKIPIPTELVVVLGATLASHFGDL  323
              ILSNIHHTNLCDLVTSAICIFVLVAGKELQERFKDRLKIPIPTELVVVLGATLASHFGDL
Sbjct  39847  ILSNIHHTNLCDLVTSAICIFVLVAGKELQERFKDRLKIPIPTELVVVLGATLASHFGDL  39668

Query  324    HGLYGSSVSGHIPTGFISPTVPSFGLMPRVALDAVPLAVISFAFTVSLSEMFAKKNGYTV  383
              HGLYGSSVSGHIPTGFISPTVPSFGLMPRVALDAVPLAVISFAFTVSLSEMFAKKNGYTV
Sbjct  39667  HGLYGSSVSGHIPTGFISPTVPSFGLMPRVALDAVPLAVISFAFTVSLSEMFAKKNGYTV  39488

Query  384    RPNQEMLAIGCCNIIPSFFHCFTTSAALAKTMVKDSTGCQTQV  426
              RPNQEMLAIGCCNIIPSFFHCFTTSAALAKTMVKDSTGCQTQV
Sbjct  39487  RPNQEMLAIGCCNIIPSFFHCFTTSAALAKTMVKDSTGCQTQV  39359



Range 3: 40823 to 41230

Score:514 bits(1048), Expect:3e-144,
Method:,
Identities:136/136(100%), Positives:136/136(100%), Gaps:0/136(0%)

Query  1      MENHDGVTEILPPPPLLERCARQRRPPLSVLRTRVKQGLTCSVPRVKATLTGFFPVVHWL  60
              MENHDGVTEILPPPPLLERCARQRRPPLSVLRTRVKQGLTCSVPRVKATLTGFFPVVHWL
Sbjct  41230  MENHDGVTEILPPPPLLERCARQRRPPLSVLRTRVKQGLTCSVPRVKATLTGFFPVVHWL  41051

Query  61     PKYKLREYVWGDLMSGLIVGIILVPQAIAYCLLAGVDPIYGLYTSFFANIIYFFMGTSRH  120
              PKYKLREYVWGDLMSGLIVGIILVPQAIAYCLLAGVDPIYGLYTSFFANIIYFFMGTSRH
Sbjct  41050  PKYKLREYVWGDLMSGLIVGIILVPQAIAYCLLAGVDPIYGLYTSFFANIIYFFMGTSRH  40871

Query  121    VSVGIFSLMSLMVGQV  136
              VSVGIFSLMSLMVGQV
Sbjct  40870  VSVGIFSLMSLMVGQV  40823



Range 4: 40189 to 40401

Score:265 bits(539), Expect:3e-69,
Method:,
Identities:71/71(100%), Positives:71/71(100%), Gaps:0/71(0%)

Query  135    QVVDREVYLAGFDMSDGSTVTMAPGMWNDTGPGDTNLTAAGVEVMGMECGKECYAISVAA  194
              QVVDREVYLAGFDMSDGSTVTMAPGMWNDTGPGDTNLTAAGVEVMGMECGKECYAISVAA
Sbjct  40401  QVVDREVYLAGFDMSDGSTVTMAPGMWNDTGPGDTNLTAAGVEVMGMECGKECYAISVAA  40222

Query  195    ATTFLAGIYQV  205
              ATTFLAGIYQV
Sbjct  40221  ATTFLAGIYQV  40189
```

DS Antarctic jonasfish *slc26a2*

Query: unnamed protein product Query ID: lcl|Query_4692645 Length: 731

>Notolepis coatsorum genome assembly, contig: hap_ptg006377l_1_1, whole genome shotgun sequence

```
Sequence ID: CAXASL010030251.1 Length: 157208
Range 1: 27670 to 28503

Score:504 bits(1297), Expect:9e-155,
Method:Compositional matrix adjust.,
Identities:277/278(99%), Positives:278/278(100%), Gaps:0/278(0%)

Query  454    KCVLAVIIVVNLRGALRKFTDVPRMWRVNRVDASIWLVTMATSALVNTELGLLVGVLVSA  513
              +CVLAVIIVVNLRGALRKFTDVPRMWRVNRVDASIWLVTMATSALVNTELGLLVGVLVSA
Sbjct  28503  RCVLAVIIVVNLRGALRKFTDVPRMWRVNRVDASIWLVTMATSALVNTELGLLVGVLVSA  28324

Query  514    FCVLGRTQRAQALELGHAGARELYEDVASYQGLRTQPGVAVFRYEAPIYYANQRLFKASL  573
              FCVLGRTQRAQALELGHAGARELYEDVASYQGLRTQPGVAVFRYEAPIYYANQRLFKASL
Sbjct  28323  FCVLGRTQRAQALELGHAGARELYEDVASYQGLRTQPGVAVFRYEAPIYYANQRLFKASL  28144

Query  574    YRRLGLDPVkekarrrklekkkamreeVGAARKEEQGIAGKEGETEVAVTKGLMPEQKGP  633
              YRRLGLDPVKEKARRRKLEKKKAMREEVGAARKEEQGIAGKEGETEVAVTKGLMPEQKGP
Sbjct  28143  YRRLGLDPVKEKARRRKLEKKKAMREEVGAARKEEQGIAGKEGETEVAVTKGLMPEQKGP  27964

Query  634    GSFHSVVMDCSAVLFLDTAGVNALKEVRKDYKELGVKVLLAQCNTSVRDSLERGGYFPev  693
              GSFHSVVMDCSAVLFLDTAGVNALKEVRKDYKELGVKVLLAQCNTSVRDSLERGGYFPEV
Sbjct  27963  GSFHSVVMDCSAVLFLDTAGVNALKEVRKDYKELGVKVLLAQCNTSVRDSLERGGYFPEV  27784

Query  694    kvkeeeggeREMVFFTITDAVRYAQSLATPNGDCDTYC  731
              KVKEEEGGEREMVFFTITDAVRYAQSLATPNGDCDTYC
Sbjct  27783  KVKEEEGGEREMVFFTITDAVRYAQSLATPNGDCDTYC  27670
```

```
Range 2: 35321 to 35944

Score:384 bits(986), Expect:1e-113,
Method:Compositional matrix adjust.,
Identities:208/208(100%), Positives:208/208(100%), Gaps:0/208(0%)

Query  1      MTSGEDCCTaaaaeeeeeGVESDHHHPLILERAEKEEENWKSAVSHQVKKHCSCTKEKAK  60
              MTSGEDCCTAAAAEEEEEGVESDHHHPLILERAEKEEENWKSAVSHQVKKHCSCTKEKAK
Sbjct  35944  MTSGEDCCTAAAAEEEEEGVESDHHHPLILERAEKEEENWKSAVSHQVKKHCSCTKEKAK  35765

Query  61     NKILGFVPIIKWLPRYQLRDWILGDLMSGLIVGILLVPQSIAYSLLAGQDPIYGLYTSFF  120
              NKILGFVPIIKWLPRYQLRDWILGDLMSGLIVGILLVPQSIAYSLLAGQDPIYGLYTSFF
Sbjct  35764  NKILGFVPIIKWLPRYQLRDWILGDLMSGLIVGILLVPQSIAYSLLAGQDPIYGLYTSFF  35585

Query  121    ASIIYALLGTSRHISVGIFGVLCLLVGQVVDRELAVAGYLTEDSSGGNDTAALLAGMGKD  180
              ASIIYALLGTSRHISVGIFGVLCLLVGQVVDRELAVAGYLTEDSSGGNDTAALLAGMGKD
Sbjct  35584  ASIIYALLGTSRHISVGIFGVLCLLVGQVVDRELAVAGYLTEDSSGGNDTAALLAGMGKD  35405

Query  181    GALMECDKSCYAIvvgatvtftagvYQV  208
              GALMECDKSCYAIVVGATVTFTAGVYQV
Sbjct  35404  GALMECDKSCYAIVVGATVTFTAGVYQV  35321
```

```
Range 3: 29263 to 29724

Score:273 bits(699), Expect:8e-76,
Method:Compositional matrix adjust.,
Identities:152/154(99%), Positives:153/154(99%), Gaps:0/154(0%)

Query  304    KAPIPFELFVVIIATLASHFGHFNTTYLSDVAGAIPTGFLPPQMPAWSLIPSVAVDAFSI  363
              +APIPFELFVVIIATLASHFGHFNTTYLSDVAGAIPTGFLPPQMPAWSLIPSVAVDAFSI
Sbjct  29724  QAPIPFELFVVIIATLASHFGHFNTTYLSDVAGAIPTGFLPPQMPAWSLIPSVAVDAFSI  29545

Query  364    AIVGFAITVSLSEMFAKKHGYVVDPNQEMYAIGFCNILPAFFRCFTTSAALTKTLVKEST  423
              AIVGFAITVSLSEMFAKKHGYVVDPNQEMYAIGFCNILPAFFRCFTTSAALTKTLVKEST
Sbjct  29544  AIVGFAITVSLSEMFAKKHGYVVDPNQEMYAIGFCNILPAFFRCFTTSAALTKTLVKEST  29365

Query  424    GCQTQvsglvsalvlllvllvIAPLFYSLQKCVL  457
              GCQTQVSGLVSALVLLLVLLVIAPLFYSLQK VL
Sbjct  29364  GCQTQVSGLVSALVLLLVLLVIAPLFYSLQK*VL  29263
```

```
Range 4: 31654 to 31947

Score:161 bits(407), Expect:1e-38,
Method:Compositional matrix adjust.,
Identities:98/98(100%), Positives:98/98(100%), Gaps:0/98(0%)

Query  207    QVLMGLLQVGFVSVYLSDSLLSGFATGASLTILTSQLKYLLGLKIPRPQGWFTLGKTWFS  266
              QVLMGLLQVGFVSVYLSDSLLSGFATGASLTILTSQLKYLLGLKIPRPQGWFTLGKTWFS
Sbjct  31947  QVLMGLLQVGFVSVYLSDSLLSGFATGASLTILTSQLKYLLGLKIPRPQGWFTLGKTWFS  31768

Query  267    LLANLGNTNlcdlvtsllcllvllPAKEFNDRFKANLK  304
              LLANLGNTNLCDLVTSLLCLLVLLPAKEFNDRFKANLK
Sbjct  31767  LLANLGNTNLCDLVTSLLCLLVLLPAKEFNDRFKANLK  31654
```

DT Minispotted lanternfish *slc26a2*

```
Query: unnamed protein product Query ID: lcl|Query_6846471 Length: 734
```

```
>Gymnoscopelus microlampas genome assembly, contig: atg000397l_1, whole genome shotgun sequence
Sequence ID: CAUOFV010000354.1 Length: 682658
Range 1: 581469 to 582350

Score:498 bits(1282), Expect:3e-153,
Method:Compositional matrix adjust.,
Identities:291/296(98%), Positives:293/296(98%), Gaps:2/296(0%)

Query  439     PLFYSLQKCVLAVIIVVNLRGALRRFTDVPRMWRANRIDAAVWLVTMASSALVNTELGLL  498
               P+F  L +CVLAVIIVVNLRGALRRFTDVPRMWRANRIDAAVWLVTMASSALVNTELGLL
Sbjct  581469  PVF--LSRCVLAVIIVVNLRGALRRFTDVPRMWRANRIDAAVWLVTMASSALVNTELGLL  581642

Query  499     VGVLVSAFSILGRTQRARAVELGQAGACEESREVYEDLETYRGLRRQPGVAVFRYEAPIY  558
               VGVLVSAFSILGRTQRARAVELGQAGACEESREVYEDLETYRGLRRQPGVAVFRYEAPIY
Sbjct  581643  VGVLVSAFSILGRTQRARAVELGQAGACEESREVYEDLETYRGLRRQPGVAVFRYEAPIY  581822

Query  559     YANQSLFKAALYRRLGLDPVkekawrrklekrkkkqekpkeaaTMGRVEETGDMGKEINP  618
               YANQSLFKAALYRRLGLDPVKEKAWRRKLEKRKKKQEKPKEAATMGRVEETGDMGKEINP
Sbjct  581823  YANQSLFKAALYRRLGLDPVKEKAWRRKLEKRKKKQEKPKEAATMGRVEETGDMGKEINP  582002

Query  619     EAGVTLNLMHEKDTSGGFHSVVIDCGPVLFLDTAGVNALKEVRKDYKEVGVRVLLARCSI  678
               EAGVTLNLMHEKDTSGGFHSVVIDCGPVLFLDTAGVNALKEVRKDYKEVGVRVLLARCSI
Sbjct  582003  EAGVTLNLMHEKDTSGGFHSVVIDCGPVLFLDTAGVNALKEVRKDYKEVGVRVLLARCSI  582182

Query  679     SVLDSLERGGYFPetkggeeekkeirerkMVFFTITDAIRYAQSLSSANGECDTYC  734
               SVLDSLERGGYFPETKGGEEEKKEIRERKMVFFTITDAIRYAQSLSSANGECDTYC
Sbjct  582183  SVLDSLERGGYFPETKGGEEEKKEIRERKMVFFTITDAIRYAQSLSSANGECDTYC  582350



Range 2: 576037 to 576636

Score:414 bits(1064), Expect:3e-124,
Method:Compositional matrix adjust.,
Identities:200/200(100%), Positives:200/200(100%), Gaps:0/200(0%)

Query  1       MTSGEVCCSLADNRTNRDQCQPLILERPQTEEENWKTLAARKLKRHCTCTPQKARAKVLG  60
               MTSGEVCCSLADNRTNRDQCQPLILERPQTEEENWKTLAARKLKRHCTCTPQKARAKVLG
Sbjct  576037  MTSGEVCCSLADNRTNRDQCQPLILERPQTEEENWKTLAARKLKRHCTCTPQKARAKVLG  576216

Query  61      FFPILRWLPRYQLREWILGDVMSGLIVGVLLVPQSIAYSLLAGQDPIYGLYTSFFSSIIY  120
               FFPILRWLPRYQLREWILGDVMSGLIVGVLLVPQSIAYSLLAGQDPIYGLYTSFFSSIIY
Sbjct  576217  FFPILRWLPRYQLREWILGDVMSGLIVGVLLVPQSIAYSLLAGQDPIYGLYTSFFSSIIY  576396

Query  121     TLLGTSRHISVGIFGVLCLLVGQVVDRELAVAGYLTESSSNGSALLEGLGNGSFGPVCDR  180
               TLLGTSRHISVGIFGVLCLLVGQVVDRELAVAGYLTESSSNGSALLEGLGNGSFGPVCDR
Sbjct  576397  TLLGTSRHISVGIFGVLCLLVGQVVDRELAVAGYLTESSSNGSALLEGLGNGSFGPVCDR  576576

Query  181     SCYAIMVGSTVTFTAGVYQV  200
               SCYAIMVGSTVTFTAGVYQV
Sbjct  576577  SCYAIMVGSTVTFTAGVYQV  576636



Range 3: 578989 to 579462

Score:273 bits(699), Expect:3e-76,
Method:Compositional matrix adjust.,
Identities:152/158(96%), Positives:154/158(97%), Gaps:0/158(0%)

Query  291     FKDKLKAPIPFELVVVIAATLASHFGHFNSEYGSAVAGSIPTGFLPPQLPSWSLIPNVAL  350
               F   ++APIPFELVVVIAATLASHFGHFNSEYGSAVAGSIPTGFLPPQLPSWSLIPNVAL
Sbjct  578989  FVCPIQAPIPFELVVVIAATLASHFGHFNSEYGSAVAGSIPTGFLPPQLPSWSLIPNVAL  579168

Query  351     DAFSIAIVGFAITVSLSEMFAKKHGYTVDANQEMYAIGFCNILPSFFRCFTTSAALTKTL  410
               DAFSIAIVGFAITVSLSEMFAKKHGYTVDANQEMYAIGFCNILPSFFRCFTTSAALTKTL
Sbjct  579169  DAFSIAIVGFAITVSLSEMFAKKHGYTVDANQEMYAIGFCNILPSFFRCFTTSAALTKTL  579348

Query  411     VKESTGCQTQvsglvtslvlllvllvIAPLFYSLQKCV  448
               VKESTGCQTQVSGLVTSLVLLLVLLVIAPLFYSLQK V
Sbjct  579349  VKESTGCQTQVSGLVTSLVLLLVLLVIAPLFYSLQKLV  579462



Range 4: 577038 to 577334

Score:173 bits(439), Expect:5e-43,
Method:Compositional matrix adjust.,
Identities:98/99(99%), Positives:98/99(98%), Gaps:0/99(0%)

Query  199     QVLMGLFQVGFVSVYLSDSLLSGFATGASLTILTSQLKYLLGLNIPRPQGWFTLAKTWYS  258
               QVLMGLFQVGFVSVYLSDSLLSGFATGASLTILTSQLKYLLGLNIPRPQGWFTLAKTWYS
Sbjct  577038  QVLMGLFQVGFVSVYLSDSLLSGFATGASLTILTSQLKYLLGLNIPRPQGWFTLAKTWYS  577217

Query  259     LLANLASTNICDlvtsllcllvlvPAKELNYHFKDKLKA  297
               LLANLASTNICDLVTSLLCLLVLVPAKELNYHFKDKLK
Sbjct  577218  LLANLASTNICDLVTSLLCLLVLVPAKELNYHFKDKLKV  577334
```

DU Bigeye Pacific opah *slc26a2*

Query: unnamed protein product Query ID: lcl|Query_7520011 Length: 730

>Lampris megalopsis isolate JB-2021 Contig146, whole genome shotgun sequence
Sequence ID: JAJHIY020000054.1 Length: 2315043
Range 1: 91180 to 92043

Score:545 bits(1405), Expect:1e-169,
Method:Compositional matrix adjust.,
Identities:283/290(98%), Positives:286/290(98%), Gaps:2/290(0%)

```
Query  441    IAPLFYSLQKCVLAVIIVVNLRGALRKFKDVPRMWRVNRIDTSIWLVTMATSALVNTELG  500
              +APL    ++CVLAVIIVVNLRGALRKFKDVPRMWRVNRIDTSIWLVTMATSALVNTELG
Sbjct  91180  LAPL--CPRRCVLAVIIVVNLRGALRKFKDVPRMWRVNRIDTSIWLVTMATSALVNTELG  91353

Query  501    LLIGVLVSAFCVLGRTQQAQAVELGRAESREFYEDLASYNGLRTQPGVTVFRYRAPIYYA  560
              LLIGVLVSAFCVLGRTQQAQAVELGRAESREFYEDLASYNGLRTQPGVTVFRYRAPIYYA
Sbjct  91354  LLIGVLVSAFCVLGRTQQAQAVELGRAESREFYEDLASYNGLRTQPGVTVFRYRAPIYYA  91533

Query  561    NQSLFKSALYQRLGLNPvkekvrrrkleknkGKKQALAEPEDVKTQRSGVTGVTGKKDET  620
              NQSLFKSALYQRLGLNPVKEKVRRRKLEKNKGKKQALAEPEDVKTQRSGVTGVTGKKDET
Sbjct  91534  NQSLFKSALYQRLGLNPVKEKVRRRKLEKNKGKKQALAEPEDVKTQRSGVTGVTGKKDET  91713

Query  621    KVNITKILMPEKDASSSLHSVVIDCSAVLFLDTAGVNALKEVRKDYEELGVRVLLAQCNT  680
              KVNITKILMPEKDASSSLHSVVIDCSAVLFLDTAGVNALKEVRKDYEELGVRVLLAQCNT
Sbjct  91714  KVNITKILMPEKDASSSLHSVVIDCSAVLFLDTAGVNALKEVRKDYEELGVRVLLAQCNT  91893

Query  681    SVLDSLERGGYFYENKAETGEGKMVFFTITDAVHLAQSLSTPNGDCDTYC  730
              SVLDSLERGGYFYENKAETGEGKMVFFTITDAVHLAQSLSTPNGDCDTYC
Sbjct  91894  SVLDSLERGGYFYENKAETGEGKMVFFTITDAVHLAQSLSTPNGDCDTYC  92043
```

Range 2: 86912 to 87523

Score:401 bits(1030), Expect:1e-119,
Method:Compositional matrix adjust.,
Identities:204/204(100%), Positives:204/204(100%), Gaps:0/204(0%)

```
Query  1      MASGEDCCTTAEDGAEGNQHHLLILervekeeeeNWKTVASRQLKQHCSCTPAKAKHKLT  60
              MASGEDCCTTAEDGAEGNQHHLLILERVEKEEEENWKTVASRQLKQHCSCTPAKAKHKLT
Sbjct  86912  MASGEDCCTTAEDGAEGNQHHLLILERVEKEEEENWKTVASRQLKQHCSCTPAKAKHKLT  87091

Query  61     GFLPILKWLPHYQLRDWLLGDVMSGIIVGILLVPQSIAYSLLAGQDPIYGLYTSFFSSII  120
              GFLPILKWLPHYQLRDWLLGDVMSGIIVGILLVPQSIAYSLLAGQDPIYGLYTSFFSSII
Sbjct  87092  GFLPILKWLPHYQLRDWLLGDVMSGIIVGILLVPQSIAYSLLAGQDPIYGLYTSFFSSII  87271

Query  121    YAFLGTSHHISVGIFGVLCLLVGQVVDRELAVAGYLTESSSSGNLSYLLVAGQGNGSIEM  180
              YAFLGTSHHISVGIFGVLCLLVGQVVDRELAVAGYLTESSSSGNLSYLLVAGQGNGSIEM
Sbjct  87272  YAFLGTSHHISVGIFGVLCLLVGQVVDRELAVAGYLTESSSSGNLSYLLVAGQGNGSIEM  87451

Query  181    ECSRSCYAILVGATLTFTAGIYQV  204
              ECSRSCYAILVGATLTFTAGIYQV
Sbjct  87452  ECSRSCYAILVGATLTFTAGIYQV  87523
```

Range 3: 89038 to 89490

Score:279 bits(714), Expect:3e-78,
Method:Compositional matrix adjust.,
Identities:150/151(99%), Positives:151/151(100%), Gaps:0/151(0%)

```
Query  300    KAPIPFELFVVIAATLASHFGHFNTEYGSEVAGAIPTGFLPPQLPSWSLIPNVAVDAFTV  359
              +APIPFELFVVIAATLASHFGHFNTEYGSEVAGAIPTGFLPPQLPSWSLIPNVAVDAFTV
Sbjct  89038  QAPIPFELFVVIAATLASHFGHFNTEYGSEVAGAIPTGFLPPQLPSWSLIPNVAVDAFTV  89217

Query  360    AIVGFAITVSLSEMFAKKHGYVVDPNQEMYAIGFCNILPSFFRCFTTSAALTKTLVKEST  419
              AIVGFAITVSLSEMFAKKHGYVVDPNQEMYAIGFCNILPSFFRCFTTSAALTKTLVKEST
Sbjct  89218  AIVGFAITVSLSEMFAKKHGYVVDPNQEMYAIGFCNILPSFFRCFTTSAALTKTLVKEST  89397

Query  420    GCQTQLSGlvtalvlllvllvIAPLFYSLQK  450
              GCQTQLSGLVTALVLLLVLLVIAPLFYSLQK
Sbjct  89398  GCQTQLSGLVTALVLLLVLLVIAPLFYSLQK  89490
```

Range 4: 88107 to 88403

Score:175 bits(443), Expect:2e-43,
Method:Compositional matrix adjust.,
Identities:98/99(99%), Positives:98/99(98%), Gaps:0/99(0%)

```
Query  203    QVLMGMLQVGFVSVYLSDSLLSGFATGASLTILTSQLKYLLGLKIPRPQGWFTLGKTWYS  262
              QVLMGMLQVGFVSVYLSDSLLSGFATGASLTILTSQLKYLLGLKIPRPQGWFTLGKTWYS
Sbjct  88107  QVLMGMLQVGFVSVYLSDSLLSGFATGASLTILTSQLKYLLGLKIPRPQGWFTLGKTWYS  88286

Query  263    LITNLRNTNICDlvtsllcllvlvPTKELNDHFKAKLKA  301
              LITNLRNTNICDLVTSLLCLLVLVPTKELNDHFKAKLK
Sbjct  88287  LITNLRNTNICDLVTSLLCLLVLVPTKELNDHFKAKLKV  88403
```

DV Pinecone soldierfish *slc26a2*

Query: sulfate anion transporter 1 [Myripristis murdjan] Query ID: XP_029918692.1 Length: 728

>Myripristis murdjan chromosome 10, fMyrMur1.1, whole genome shotgun sequence
Sequence ID: NC_043989.1 Length: 34950760
Range 1: 5969257 to 5970099

Score:1016 bits(2074), Expect:0.0,
Method:,
Identities:279/281(99%), Positives:279/281(99%), Gaps:0/281(0%)

```
Query  448      LQKCVLAVIIVVNLRGALRKFADVPRMWRINRIDAFIWLITMATSALVNTELGLLVGVLV  507
                L  CVLAVIIVVNLRGALRKFADVPRMWRINRIDAFIWLITMATSALVNTELGLLVGVLV
Sbjct  5969257  LPRCVLAVIIVVNLRGALRKFADVPRMWRINRIDAFIWLITMATSALVNTELGLLVGVLV  5969436

Query  508      SAFCVLGRTQRAQALELGKAGTREHYEDLAAYRGLQRQPGVAVFRYEAPIYYANQSLFKT  567
                SAFCVLGRTQRAQALELGKAGTREHYEDLAAYRGLQRQPGVAVFRYEAPIYYANQSLFKT
Sbjct  5969437  SAFCVLGRTQRAQALELGKAGTREHYEDLAAYRGLQRQPGVAVFRYEAPIYYANQSLFKT  5969616

Query  568      SLYRRLGLDPVKEKALRRKMEKKKKKQKGKELERAEEGATGKSEEREDGVEAGMTKTLMN  627
                SLYRRLGLDPVKEKALRRKMEKKKKKQKGKELERAEEGATGKSEEREDGVEAGMTKTLMN
Sbjct  5969617  SLYRRLGLDPVKEKALRRKMEKKKKKQKGKELERAEEGATGKSEEREDGVEAGMTKTLMN  5969796

Query  628      EEKVPGSLHSLVIDCSAILFLDTAGVNALKEVRKDYGELGVRVVLAQCNTSVLDSLQRGG  687
                EEKVPGSLHSLVIDCSAILFLDTAGVNALKEVRKDYGELGVRVVLAQCNTSVLDSLQRGG
Sbjct  5969797  EEKVPGSLHSLVIDCSAILFLDTAGVNALKEVRKDYGELGVRVVLAQCNTSVLDSLQRGG  5969976

Query  688      YYSENKGGGGGESDATFFTITDAVRYTQSLSTPNGDCDSRC  728
                YYSENKGGGGGESDATFFTITDAVRYTQSLSTPNGDCDSRC
Sbjct  5969977  YYSENKGGGGGESDATFFTITDAVRYTQSLSTPNGDCDSRC  5970099
```

Range 2: 5961367 to 5961978

Score:748 bits(1526), Expect:0.0,
Method:,
Identities:204/204(100%), Positives:204/204(100%), Gaps:0/204(0%)

```
Query  1        MTRGEDCFPAAGDGAESDQQHPLILERVEKDDGEEGNWKAAASHHLKKHCSCSAEKARAK  60
                MTRGEDCFPAAGDGAESDQQHPLILERVEKDDGEEGNWKAAASHHLKKHCSCSAEKARAK
Sbjct  5961367  MTRGEDCFPAAGDGAESDQQHPLILERVEKDDGEEGNWKAAASHHLKKHCSCSAEKARAK  5961546

Query  61       ILGFFPILKWLPRYQLRDWLLGDVMSGLIVGILLVPQSIAYSLLASQDPIYGLYTSFFSS  120
                ILGFFPILKWLPRYQLRDWLLGDVMSGLIVGILLVPQSIAYSLLASQDPIYGLYTSFFSS
Sbjct  5961547  ILGFFPILKWLPRYQLRDWLLGDVMSGLIVGILLVPQSIAYSLLASQDPIYGLYTSFFSS  5961726

Query  121      IIYTLLGTSRHISVGIFGVLCLLVGQVVDRELAEAGYLTEGSGNDSAVLLAGQGNSSFAS  180
                IIYTLLGTSRHISVGIFGVLCLLVGQVVDRELAEAGYLTEGSGNDSAVLLAGQGNSSFAS
Sbjct  5961727  IIYTLLGTSRHISVGIFGVLCLLVGQVVDRELAEAGYLTEGSGNDSAVLLAGQGNSSFAS  5961906

Query  181      ECDRSCYAIMVGATVTFTAGVYQV  204
                ECDRSCYAIMVGATVTFTAGVYQV
Sbjct  5961907  ECDRSCYAIMVGATVTFTAGVYQV  5961978
```

Range 3: 5964783 to 5965232

Score:547 bits(1116), Expect:3e-159,
Method:,
Identities:150/150(100%), Positives:150/150(100%), Gaps:0/150(0%)

```
Query  301      APIPFELFVVIAATAASHFGNFNTEYGSSVAGTIPTGFLPPQLPLWSLIPNVAVDAFSIA  360
                APIPFELFVVIAATAASHFGNFNTEYGSSVAGTIPTGFLPPQLPLWSLIPNVAVDAFSIA
Sbjct  5964783  APIPFELFVVIAATAASHFGNFNTEYGSSVAGTIPTGFLPPQLPLWSLIPNVAVDAFSIA  5964962

Query  361      IVGFVITVSLSEMFAKKHGYTVDANQEMYAIGFCNILPSFFRCFTTSAALTKTLVKESTG  420
                IVGFVITVSLSEMFAKKHGYTVDANQEMYAIGFCNILPSFFRCFTTSAALTKTLVKESTG
Sbjct  5964963  IVGFVITVSLSEMFAKKHGYTVDANQEMYAIGFCNILPSFFRCFTTSAALTKTLVKESTG  5965142

Query  421      CQTQVSGLITALVLLLVLLVIAPLFYSLQK  450
                CQTQVSGLITALVLLLVLLVIAPLFYSLQK
Sbjct  5965143  CQTQVSGLITALVLLLVLLVIAPLFYSLQK  5965232
```

Range 4: 5963810 to 5964103

Score:356 bits(725), Expect:2e-101,
Method:,
Identities:98/98(100%), Positives:98/98(100%), Gaps:0/98(0%)

```
Query  203      QVLMGVLQVGFVSVYLSDSLLSGFATGASLTILTSQLKYLLGLRIARPQGWFTLIKTWYS  262
                QVLMGVLQVGFVSVYLSDSLLSGFATGASLTILTSQLKYLLGLRIARPQGWFTLIKTWYS
```

```
Sbjct  5963810  QVLMGVLQVGFVSVYLSDSLLSGFATGASLTILTSQLKYLLGLRIARPQGWFTLIKTWYS  5963989

Query  263      LLTNLGSTNICDLVTSLVCLLVLVPTKELNDRFKAKLK  300
                LLTNLGSTNICDLVTSLVCLLVLVPTKELNDRFKAKLK
Sbjct  5963990  LLTNLGSTNICDLVTSLVCLLVLVPTKELNDRFKAKLK  5964103
```

DW Yellowfin tuna *slc26a2*

Query: sulfate transporter [Thunnus albacares] Query ID: XP_044219674.1 Length: 727

>Thunnus albacares chromosome 10, fThuAlb1.1, whole genome shotgun sequence
Sequence ID: NC_058115.1 Length: 35265753
Range 1: 30246063 to 30246899

Score:1021 bits(2084), Expect:0.0,
Method:,
Identities:277/279(99%), Positives:277/279(99%), Gaps:0/279(0%)

```
Query  449       LQKCVLAVIIVVNLRGALQKFTDVPRMWRANHVDTSIWLITMATSALVNTELGLLVGVIV  508
                 L  CVLAVIIVVNLRGALQKFTDVPRMWRANHVDTSIWLITMATSALVNTELGLLVGVIV
Sbjct  30246063  LPRCVLAVIIVVNLRGALQKFTDVPRMWRANHVDTSIWLITMATSALVNTELGLLVGVIV  30246242

Query  509       SAFCVLGRTQQAQVLELGRAPNTEHYEDLSAYRGLQTHPGVAVFRYEAPIYYANQSLFKK  568
                 SAFCVLGRTQQAQVLELGRAPNTEHYEDLSAYRGLQTHPGVAVFRYEAPIYYANQSLFKK
Sbjct  30246243  SAFCVLGRTQQAQVLELGRAPNTEHYEDLSAYRGLQTHPGVAVFRYEAPIYYANQSLFKK  30246422

Query  569       SLYKSVGLDPLKEKTRRMKFKRKQSKQPKEVPNMKSKENEVAGKEDGGPDATVTLMLDEK  628
                 SLYKSVGLDPLKEKTRRMKFKRKQSKQPKEVPNMKSKENEVAGKEDGGPDATVTLMLDEK
Sbjct  30246423  SLYKSVGLDPLKEKTRRMKFKRKQSKQPKEVPNMKSKENEVAGKEDGGPDATVTLMLDEK  30246602

Query  629       SACRLHSLVMDCSAILFLDTAGVNAMKEVRKDYAELGVKVVLAQCNTSVLDALERGGYYP  688
                 SACRLHSLVMDCSAILFLDTAGVNAMKEVRKDYAELGVKVVLAQCNTSVLDALERGGYYP
Sbjct  30246603  SACRLHSLVMDCSAILFLDTAGVNAMKEVRKDYAELGVKVVLAQCNTSVLDALERGGYYP  30246782

Query  689       DKKKEGDGGESKMVFFTIEDAIHYVQSLSVPNGNYDSKC  727
                 DKKKEGDGGESKMVFFTIEDAIHYVQSLSVPNGNYDSKC
Sbjct  30246783  DKKKEGDGGESKMVFFTIEDAIHYVQSLSVPNGNYDSKC  30246899
```

Range 2: 30241629 to 30242243

Score:751 bits(1533), Expect:0.0,
Method:,
Identities:205/205(100%), Positives:205/205(100%), Gaps:0/205(0%)

```
Query  1         MTHDDDCTTGEDGAEGDQQHHPLILERVEKDTEKNWKTVVSNRLKKHCSCTSKKAKSKIL  60
                 MTHDDDCTTGEDGAEGDQQHHPLILERVEKDTEKNWKTVVSNRLKKHCSCTSKKAKSKIL
Sbjct  30241629  MTHDDDCTTGEDGAEGDQQHHPLILERVEKDTEKNWKTVVSNRLKKHCSCTSKKAKSKIL  30241808

Query  61        GFVPILKWLPQYQLRDWLLGDVMSGVIVGILLVPQSIAYSLLASQDPVYGLYTSFFASII  120
                 GFVPILKWLPQYQLRDWLLGDVMSGVIVGILLVPQSIAYSLLASQDPVYGLYTSFFASII
Sbjct  30241809  GFVPILKWLPQYQLRDWLLGDVMSGVIVGILLVPQSIAYSLLASQDPVYGLYTSFFASII  30241988

Query  121       YALLGTSRHISVGIFAVLCLLVGQVVDRELALAGYLTESSSISGNDSTVLLAGQGNNSYA  180
                 YALLGTSRHISVGIFAVLCLLVGQVVDRELALAGYLTESSSISGNDSTVLLAGQGNNSYA
Sbjct  30241989  YALLGTSRHISVGIFAVLCLLVGQVVDRELALAGYLTESSSISGNDSTVLLAGQGNNSYA  30242168

Query  181       VDCDRSCYAITVGATVTFTAGVYQV  205
                 VDCDRSCYAITVGATVTFTAGVYQV
Sbjct  30242169  VDCDRSCYAITVGATVTFTAGVYQV  30242243
```

Range 3: 30244122 to 30244571

Score:550 bits(1122), Expect:2e-160,
Method:,
Identities:150/150(100%), Positives:150/150(100%), Gaps:0/150(0%)

```
Query  302       APIPFELFVVIIATLASHFGRFNTEYGSGVAGDIPTGFLPPQMPLWSLIPSVAVDAFSVA  361
                 APIPFELFVVIIATLASHFGRFNTEYGSGVAGDIPTGFLPPQMPLWSLIPSVAVDAFSVA
Sbjct  30244122  APIPFELFVVIIATLASHFGRFNTEYGSGVAGDIPTGFLPPQMPLWSLIPSVAVDAFSVA  30244301

Query  362       IVGFVITVSLSEMFAKKHGYKVDANQEMYAIGICNILPSFFHCFTTSAALTKTLVKESTG  421
                 IVGFVITVSLSEMFAKKHGYKVDANQEMYAIGICNILPSFFHCFTTSAALTKTLVKESTG
Sbjct  30244302  IVGFVITVSLSEMFAKKHGYKVDANQEMYAIGICNILPSFFHCFTTSAALTKTLVKESTG  30244481

Query  422       CQTQISGLISALVLLLVLLVIAPIFYSLQK  451
                 CQTQISGLISALVLLLVLLVIAPIFYSLQK
Sbjct  30244482  CQTQISGLISALVLLLVLLVIAPIFYSLQK  30244571
```

Range 4: 30242593 to 30242886

Score:361 bits(736), Expect:2e-103,

Method:,
Identities:98/98(100%), Positives:98/98(100%), Gaps:0/98(0%)

```
Query  204       QVLMGFLQVGFVSVYLSDSLLSGFATGASLTILTSQFKYLLGLKIPRPQGWFTLFKTWYS  263
                 QVLMGFLQVGFVSVYLSDSLLSGFATGASLTILTSQFKYLLGLKIPRPQGWFTLFKTWYS
Sbjct  30242593  QVLMGFLQVGFVSVYLSDSLLSGFATGASLTILTSQFKYLLGLKIPRPQGWFTLFKTWYS  30242772

Query  264       VFTNLGNTNICDLVTSLVCLVVLIPTKELNDRFKSKLK  301
                 VFTNLGNTNICDLVTSLVCLVVLIPTKELNDRFKSKLK
Sbjct  30242773  VFTNLGNTNICDLVTSLVCLVVLIPTKELNDRFKSKLK  30242886
```

DX Mandarinfish *slc26a2*

Query: sulfate transporter [Synchiropus splendidus] Query ID: XP_053732140.1 Length: 726

>Synchiropus splendidus isolate RoL2022-P1 chromosome 10, RoL_Sspl_1.0, whole genome shotgun sequence
Sequence ID: NC_071343.1 Length: 25476212
Range 1: 11381756 to 11382523

Score:928 bits(1895), Expect:0.0,
Method:,
Identities:254/256(99%), Positives:254/256(99%), Gaps:0/256(0%)

```
Query  471       LQKCVLAVIIVVNLRGALRKFTEIPSMWHLNRIDTGIWLLTMATSALVNTELGLLVGVLA  530
                 L  CVLAVIIVVNLRGALRKFTEIPSMWHLNRIDTGIWLLTMATSALVNTELGLLVGVLA
Sbjct  11381756  LSRCVLAVIIVVNLRGALRKFTEIPSMWHLNRIDTGIWLLTMATSALVNTELGLLVGVLA  11381935

Query  531       SAFSVIGRTQRAQMLELGCASSREHYEDLTAYYGLHTHPGVAVFRYQAPVYYANQSLFKK  590
                 SAFSVIGRTQRAQMLELGCASSREHYEDLTAYYGLHTHPGVAVFRYQAPVYYANQSLFKK
Sbjct  11381936  SAFSVIGRTQRAQMLELGCASSREHYEDLTAYYGLHTHPGVAVFRYQAPVYYANQSLFKK  11382115

Query  591       SLYKCTGLNPGKARRRMLKLKRQNGEKDAVEADASTSVTLDGNPPRRLHSVVMDCSAVLF  650
                 SLYKCTGLNPGKARRRMLKLKRQNGEKDAVEADASTSVTLDGNPPRRLHSVVMDCSAVLF
Sbjct  11382116  SLYKCTGLNPGKARRRMLKLKRQNGEKDAVEADASTSVTLDGNPPRRLHSVVMDCSAVLF  11382295

Query  651       LDSAGVNAVKEVHKDYGELGVKVILAQCSTSVVDALERGGYFSDQNGGERIFFTIADAVR  710
                 LDSAGVNAVKEVHKDYGELGVKVILAQCSTSVVDALERGGYFSDQNGGERIFFTIADAVR
Sbjct  11382296  LDSAGVNAVKEVHKDYGELGVKVILAQCSTSVVDALERGGYFSDQNGGERIFFTIADAVR  11382475

Query  711       YAQSHSVANGDCDSKC  726
                 YAQSHSVANGDCDSKC
Sbjct  11382476  YAQSHSVANGDCDSKC  11382523
```

Range 2: 11378155 to 11378769

Score:753 bits(1536), Expect:0.0,
Method:,
Identities:205/205(100%), Positives:205/205(100%), Gaps:0/205(0%)

```
Query  23        EPSWIMTHGDASRPPSEDGAESDQPLVLERLPKTTDPSWRTCMSRRLKKHCSCTSDKVKS  82
                 EPSWIMTHGDASRPPSEDGAESDQPLVLERLPKTTDPSWRTCMSRRLKKHCSCTSDKVKS
Sbjct  11378155  EPSWIMTHGDASRPPSEDGAESDQPLVLERLPKTTDPSWRTCMSRRLKKHCSCTSDKVKS  11378334

Query  83        KIQGFVPILKWLPNYRLKDWILGDAMSGLIVGVLLVPQSIAYSLLASQDPIYGLYTSFFS  142
                 KIQGFVPILKWLPNYRLKDWILGDAMSGLIVGVLLVPQSIAYSLLASQDPIYGLYTSFFS
Sbjct  11378335  KIQGFVPILKWLPNYRLKDWILGDAMSGLIVGVLLVPQSIAYSLLASQDPIYGLYTSFFS  11378514

Query  143       SIIYALLGTSRHISVGIFGVLCLLVGQVVDRELASAGYLTEATSNNSAAPLAGLVNNSVD  202
                 SIIYALLGTSRHISVGIFGVLCLLVGQVVDRELASAGYLTEATSNNSAAPLAGLVNNSVD
Sbjct  11378515  SIIYALLGTSRHISVGIFGVLCLLVGQVVDRELASAGYLTEATSNNSAAPLAGLVNNSVD  11378694

Query  203       PACDRSCYAITVGATVTFTAGVYQV  227
                 PACDRSCYAITVGATVTFTAGVYQV
Sbjct  11378695  PACDRSCYAITVGATVTFTAGVYQV  11378769
```

Range 3: 11380196 to 11380645

Score:549 bits(1119), Expect:0.0,
Method:,
Identities:150/150(100%), Positives:150/150(100%), Gaps:0/150(0%)

```
Query  324       APIPFELFVVIIATLASHFGHFNADYGSGVAGDIPTGFLPPQLPSWSLIPNVAVDAFSIA  383
                 APIPFELFVVIIATLASHFGHFNADYGSGVAGDIPTGFLPPQLPSWSLIPNVAVDAFSIA
Sbjct  11380196  APIPFELFVVIIATLASHFGHFNADYGSGVAGDIPTGFLPPQLPSWSLIPNVAVDAFSIA  11380375

Query  384       IVGFAITVSLSEMFAKKHGYSVDANQEMYAIGFCNIIPSFFRCFANSAALTKTLVRDSTG  443
                 IVGFAITVSLSEMFAKKHGYSVDANQEMYAIGFCNIIPSFFRCFANSAALTKTLVRDSTG
Sbjct  11380376  IVGFAITVSLSEMFAKKHGYSVDANQEMYAIGFCNIIPSFFRCFANSAALTKTLVRDSTG  11380555

Query  444       CHTQLSGLVSALVLLLVLLIIAPLFYSLQK  473
                 CHTQLSGLVSALVLLLVLLIIAPLFYSLQK
Sbjct  11380556  CHTQLSGLVSALVLLLVLLIIAPLFYSLQK  11380645
```

```
Range 4: 11378866 to 11379159

Score:362 bits(737), Expect:0.0,
Method:,
Identities:98/98(100%), Positives:98/98(100%), Gaps:0/98(0%)

Query  226       QVLMGVFQVGFVSVYLSDSLLSGFATGASLTILTSQVKYLLGLKIPRPTGWFTLIKTWYG  285
                  QVLMGVFQVGFVSVYLSDSLLSGFATGASLTILTSQVKYLLGLKIPRPTGWFTLIKTWYG
Sbjct  11378866  QVLMGVFQVGFVSVYLSDSLLSGFATGASLTILTSQVKYLLGLKIPRPTGWFTLIKTWYG  11379045

Query  286       VFTNLGKTNICDLVTSLVCLLILIPTKEINYRFKDKLK  323
                  VFTNLGKTNICDLVTSLVCLLILIPTKEINYRFKDKLK
Sbjct  11379046  VFTNLGKTNICDLVTSLVCLLILIPTKEINYRFKDKLK  11379159
```

```
Range 5: 11377232 to 11377297

Score:86.4 bits(173), Expect:1e-20,
Method:,
Identities:22/22(100%), Positives:22/22(100%), Gaps:0/22(0%)

Query  1         MKVPLCGRNFTFSSQTLHFRNP  22
                  MKVPLCGRNFTFSSQTLHFRNP
Sbjct  11377232  MKVPLCGRNFTFSSQTLHFRNP  11377297
```

DY Straightnose pipefish *slc26a2*

```
Query: sulfate transporter [Nerophis ophidion] Query ID: XP_061747953.1 Length: 704


>Nerophis ophidion isolate RoL-2023_Sa linkage group LG29, whole genome shotgun sequence
Sequence ID: JAWNBE010000029.1 Length: 31224652
Range 1: 1576930 to 1577715

Score:523 bits(1347), Expect:0.0,
Method:Compositional matrix adjust.,
Identities:256/262(98%), Positives:258/262(98%), Gaps:0/262(0%)

Query  443      PLFYSLQKCVLAVVIVVNLRGALRKFAEVPGMWRANRVDASIWLITMTTSALVNTELGLL  502
                P+  S  +CVLAVVIVVNLRGALRKFAEVPGMWRANRVDASIWLITMTTSALVNTELGLL
Sbjct  1576930  PVLSSRSRCVLAVVIVVNLRGALRKFAEVPGMWRANRVDASIWLITMTTSALVNTELGLL  1577109

Query  503      VGVLVSAFSVLVRTQRAQVLELGRTPGRDHYEALASYQGLHAHPGVVVVRYPAPIYYANQ  562
                VGVLVSAFSVLVRTQRAQVLELGRTPGRDHYEALASYQGLHAHPGVVVVRYPAPIYYANQ
Sbjct  1577110  VGVLVSAFSVLVRTQRAQVLELGRTPGRDHYEALASYQGLHAHPGVVVVRYPAPIYYANQ  1577289

Query  563      TLFRKSLYRRVGLDPVEEKTLRVKLGKKSKDKEGVQEDGESASSVTLRLDKKSQCALRGL  622
                TLFRKSLYRRVGLDPVEEKTLRVKLGKKSKDKEGVQEDGESASSVTLRLDKKSQCALRGL
Sbjct  1577290  TLFRKSLYRRVGLDPVEEKTLRVKLGKKSKDKEGVQEDGESASSVTLRLDKKSQCALRGL  1577469

Query  623      VLDCSAILFLDTVGVNALKEVRKDYAEIGVTVVLAQCNTAVLDALQQGEYIPERDDRAFF  682
                VLDCSAILFLDTVGVNALKEVRKDYAEIGVTVVLAQCNTAVLDALQQGEYIPERDDRAFF
Sbjct  1577470  VLDCSAILFLDTVGVNALKEVRKDYAEIGVTVVLAQCNTAVLDALQQGEYIPERDDRAFF  1577649

Query  683      FTIADAVRHVQHVSTPNGDAGL  704
                FTIADAVRHVQHVSTPNGDAGL
Sbjct  1577650  FTIADAVRHVQHVSTPNGDAGL  1577715
```

```
Range 2: 1576361 to 1576837

Score:275 bits(703), Expect:0.0,
Method:Compositional matrix adjust.,
Identities:152/159(96%), Positives:154/159(96%), Gaps:0/159(0%)

Query  292      NYRFKAKLKAPIPFELFVVIIATLASHFGRFHLEYGSEVAGEIPTGFMPPQLPSWSLIPN  351
                 + F   L+APIPFELFVVIIATLASHFGRFHLEYGSEVAGEIPTGFMPPQLPSWSLIPN
Sbjct  1576361  GFDFLFLLQAPIPFELFVVIIATLASHFGRFHLEYGSEVAGEIPTGFMPPQLPSWSLIPN  1576540

Query  352      VAVDALAIALVGFAITVSLSEMFAKKHGYQVDANQELYAIGFCNVLPSFFRCFTTSAALT  411
                VAVDALAIALVGFAITVSLSEMFAKKHGYQVDANQELYAIGFCNVLPSFFRCFTTSAALT
Sbjct  1576541  VAVDALAIALVGFAITVSLSEMFAKKHGYQVDANQELYAIGFCNVLPSFFRCFTTSAALT  1576720

Query  412      KTLVKESTGCQTQvsglvtavvlllvllvIAPLFYSLQK  450
                KTLVKESTGCQTQVSGLVTAVVLLLVLLVIAPLFYSLQK
Sbjct  1576721  KTLVKESTGCQTQVSGLVTAVVLLLVLLVIAPLFYSLQK  1576837
```

```
Range 3: 1569430 to 1570041

Score:394 bits(1013), Expect:1e-117,
Method:Compositional matrix adjust.,
Identities:204/204(100%), Positives:204/204(100%), Gaps:0/204(0%)
```

```
Query  1        MHHGDGRRSTASEDKAESEQHRPLILERVEERTSPGCWTVASRRLKKHCSCSQQKVKAKI  60
                MHHGDGRRSTASEDKAESEQHRPLILERVEERTSPGCWTVASRRLKKHCSCSQQKVKAKI
Sbjct  1569430  MHHGDGRRSTASEDKAESEQHRPLILERVEERTSPGCWTVASRRLKKHCSCSQQKVKAKI  1569609

Query  61       LDFLPVLKWLPHYQLKDWILGDVMSGLVVGILLVPQSIAYSLLAGQDPIYGLYTSFFSSI  120
                LDFLPVLKWLPHYQLKDWILGDVMSGLVVGILLVPQSIAYSLLAGQDPIYGLYTSFFSSI
Sbjct  1569610  LDFLPVLKWLPHYQLKDWILGDVMSGLVVGILLVPQSIAYSLLAGQDPIYGLYTSFFSSI  1569789

Query  121      IYTLLGTSRHISVGIFGVLCLLVGQVVDRELALAGYLSESISTNNSAALLADSGNGSAVV  180
                IYTLLGTSRHISVGIFGVLCLLVGQVVDRELALAGYLSESISTNNSAALLADSGNGSAVV
Sbjct  1569790  IYTLLGTSRHISVGIFGVLCLLVGQVVDRELALAGYLSESISTNNSAALLADSGNGSAVV  1569969

Query  181      LCDRSCYAIvvgatvtftagvYQV  204
                LCDRSCYAIVVGATVTFTAGVYQV
Sbjct  1569970  LCDRSCYAIVVGATVTFTAGVYQV  1570041
```

Range 4: 1571617 to 1571910

Score:162 bits(410), Expect:4e-39,
Method:Compositional matrix adjust.,
Identities:98/98(100%), Positives:98/98(100%), Gaps:0/98(0%)

```
Query  203      QVLMAVFQVGFISVYLSNSLLSGFATGASLTILTSQVKYLLGLKLRRPQGWFTLIKTWMG  262
                QVLMAVFQVGFISVYLSNSLLSGFATGASLTILTSQVKYLLGLKLRRPQGWFTLIKTWMG
Sbjct  1571617  QVLMAVFQVGFISVYLSNSLLSGFATGASLTILTSQVKYLLGLKLRRPQGWFTLIKTWMG  1571796

Query  263      LLANVGQTNlcdlltslvcllllvPTKELNYRFKAKLK  300
                LLANVGQTNLCDLLTSLVCLLLLVPTKELNYRFKAKLK
Sbjct  1571797  LLANVGQTNLCDLLTSLVCLLLLVPTKELNYRFKAKLK  1571910
```

DZ Broad-nosed pipefish *slc26a2*

Query: sulfate transporter [Syngnathus typhle] Query ID: XP_061142173.1 Length: 709

>Syngnathus typhle isolate RoL2023-S1 linkage group LG1, whole genome shotgun sequence
Sequence ID: JAWIVE010000001.1 Length: 29860936
Range 1: 9886582 to 9887382

Score:543 bits(1398), Expect:0.0,
Method:Compositional matrix adjust.,
Identities:263/267(99%), Positives:264/267(98%), Gaps:0/267(0%)

```
Query  443      FYSLQKCVLAVIIVVNLRGALRKFAEVASMWQANRVDAAVWLITMATSALINTELGLLVG  502
                F  L +CVLAVIIVVNLRGALRKFAEVASMWQANRVDAAVWLITMATSALINTELGLLVG
Sbjct  9887382  FAFLFRCVLAVIIVVNLRGALRKFAEVASMWQANRVDAAVWLITMATSALINTELGLLVG  9887203

Query  503      ILVSAFSVLARTQQAQALELGRTFDRDHYDVMSAYRGLHAHPGVAVFRYPAPIYYANQSL  562
                ILVSAFSVLARTQQAQALELGRTFDRDHYDVMSAYRGLHAHPGVAVFRYPAPIYYANQSL
Sbjct  9887202  ILVSAFSVLARTQQAQALELGRTFDRDHYDVMSAYRGLHAHPGVAVFRYPAPIYYANQSL  9887023

Query  563      FKKSLYRRMGLDPVKEKARRDKFEKKLKRQNAGQESAHKKDGECTASVTLTLDQKSFRSL  622
                FKKSLYRRMGLDPVKEKARRDKFEKKLKRQNAGQESAHKKDGECTASVTLTLDQKSFRSL
Sbjct  9887022  FKKSLYRRMGLDPVKEKARRDKFEKKLKRQNAGQESAHKKDGECTASVTLTLDQKSFRSL  9886843

Query  623      VLDCSAVLFLDCAGVNALKEMRNDYGELGVTVVLAQCNTVILDALRRGGYFTEEQGGVEG  682
                VLDCSAVLFLDCAGVNALKEMRNDYGELGVTVVLAQCNTVILDALRRGGYFTEEQGGVEG
Sbjct  9886842  VLDCSAVLFLDCAGVNALKEMRNDYGELGVTVVLAQCNTVILDALRRGGYFTEEQGGVEG  9886663

Query  683      LHFFTIADAVHHLQRALPPNGDCNGTR  709
                LHFFTIADAVHHLQRALPPNGDCNGTR
Sbjct  9886662  LHFFTIADAVHHLQRALPPNGDCNGTR  9886582
```

Range 2: 9887441 to 9887893

Score:266 bits(680), Expect:0.0,
Method:Compositional matrix adjust.,
Identities:150/151(99%), Positives:151/151(100%), Gaps:0/151(0%)

```
Query  298      KTPIPFELFVVIIATLASHFGRFNADYGSAVAGEIPTGFMPPQLPAWSLIPSVALDAFSI  357
                +TPIPFELFVVIIATLASHFGRFNADYGSAVAGEIPTGFMPPQLPAWSLIPSVALDAFSI
Sbjct  9887893  QTPIPFELFVVIIATLASHFGRFNADYGSAVAGEIPTGFMPPQLPAWSLIPSVALDAFSI  9887714

Query  358      AIVGFAITVSLSEMFAKKHGYRVDANQEMFAIGFCNILPSFFRCFTTSAALTKTLVKEST  417
                AIVGFAITVSLSEMFAKKHGYRVDANQEMFAIGFCNILPSFFRCFTTSAALTKTLVKEST
Sbjct  9887713  AIVGFAITVSLSEMFAKKHGYRVDANQEMFAIGFCNILPSFFRCFTTSAALTKTLVKEST  9887534

Query  418      GCQTQVSalvtaavlllvllviaplFYSLQK  448
                GCQTQVSALVTAAVLLLVLLVIAPLFYSLQK
Sbjct  9887533  GCQTQVSALVTAAVLLLVLLVIAPLFYSLQK  9887441
```

Range 3: 9888373 to 9889266

Score:543 bits(1398), Expect:3e-169,

```
Method:Compositional matrix adjust.,
Identities:298/298(100%), Positives:298/298(100%), Gaps:0/298(0%)

Query  1        MPHRDACCATSGDETNNEQCGPLILERVPEEEGQGCRTVLSRHFRKYCSCSPQKIKYKVL  60
                MPHRDACCATSGDETNNEQCGPLILERVPEEEGQGCRTVLSRHFRKYCSCSPQKIKYKVL
Sbjct  9889266  MPHRDACCATSGDETNNEQCGPLILERVPEEEGQGCRTVLSRHFRKYCSCSPQKIKYKVL  9889087

Query  61       GFIPILKWLPRYQLRDWLLGDIMSGLIVGILLVPQSIAYSLLAGQDPIYGLYTSFFASII  120
                GFIPILKWLPRYQLRDWLLGDIMSGLIVGILLVPQSIAYSLLAGQDPIYGLYTSFFASII
Sbjct  9889086  GFIPILKWLPRYQLRDWLLGDIMSGLIVGILLVPQSIAYSLLAGQDPIYGLYTSFFASII  9888907

Query  121      YTLLGTSKHISVGIFGVLCLLVGQVVDRELVLAGYLSEGGLGSNGNATVEGSNNSMVEAC  180
                YTLLGTSKHISVGIFGVLCLLVGQVVDRELVLAGYLSEGGLGSNGNATVEGSNNSMVEAC
Sbjct  9888906  YTLLGTSKHISVGIFGVLCLLVGQVVDRELVLAGYLSEGGLGSNGNATVEGSNNSMVEAC  9888727

Query  181      DRSCYAItvgatvtfvagvYQVLMGIFQVGFVSVYLSDSLLSGFATGASLTILTSQVKYM  240
                DRSCYAITVGATVTFVAGVYQVLMGIFQVGFVSVYLSDSLLSGFATGASLTILTSQVKYM
Sbjct  9888726  DRSCYAITVGATVTFVAGVYQVLMGIFQVGFVSVYLSDSLLSGFATGASLTILTSQVKYM  9888547

Query  241      LGLKIPRPQGWFTLIKTWFSVLANLGNSNlcdlitslvcllllvptkelNNRFKAKLK  298
                LGLKIPRPQGWFTLIKTWFSVLANLGNSNLCDLITSLVCLLLLVPTKELNNRFKAKLK
Sbjct  9888546  LGLKIPRPQGWFTLIKTWFSVLANLGNSNLCDLITSLVCLLLLVPTKELNNRFKAKLK  9888373
```

EA Common seadragon *slc26a2*

```
Query: sulfate transporter isoform X2 [Phyllopteryx taeniolatus] Query ID: XP_061643192.1 Length: 698


>Phyllopteryx taeniolatus isolate TA_2022b chromosome 10, whole genome shotgun sequence
Sequence ID: JAMWDW010000010.1 Length: 31005324
Range 1: 27996820 to 27997680

Score:1044 bits(2132), Expect:0.0,
Method:,
Identities:287/287(100%), Positives:287/287(100%), Gaps:0/287(0%)

Query  1         MPHGDAGGAESERHEPLVLESVPEEAGRSCQTALCRRLQKQRSRSAREIKSEVLGFVPIL  60
                 MPHGDAGGAESERHEPLVLESVPEEAGRSCQTALCRRLQKQRSRSAREIKSEVLGFVPIL
Sbjct  27996820  MPHGDAGGAESERHEPLVLESVPEEAGRSCQTALCRRLQKQRSRSAREIKSEVLGFVPIL  27996999

Query  61        QWLPRYQPRDWLLGDVMSGLIVGILLVPQSIAYSLLAGQDPIYGLYTSFFSSIIYTLLGT  120
                 QWLPRYQPRDWLLGDVMSGLIVGILLVPQSIAYSLLAGQDPIYGLYTSFFSSIIYTLLGT
Sbjct  27997000  QWLPRYQPRDWLLGDVMSGLIVGILLVPQSIAYSLLAGQDPIYGLYTSFFSSIIYTLLGT  27997179

Query  121       SRHISVGIFGVLCLLVGQVVDREVASAGYLTEGGGDGLAGVNASALETCDRSCYAITVAA  180
                 SRHISVGIFGVLCLLVGQVVDREVASAGYLTEGGGDGLAGVNASALETCDRSCYAITVAA
Sbjct  27997180  SRHISVGIFGVLCLLVGQVVDREVASAGYLTEGGGDGLAGVNASALETCDRSCYAITVAA  27997359

Query  181       TVTFTAGVYQVLMGVFQVGFVSVYLSDSLLSGFATGASLTILTSQVKHLLGLKIQRPRGW  240
                 TVTFTAGVYQVLMGVFQVGFVSVYLSDSLLSGFATGASLTILTSQVKHLLGLKIQRPRGW
Sbjct  27997360  TVTFTAGVYQVLMGVFQVGFVSVYLSDSLLSGFATGASLTILTSQVKHLLGLKIQRPRGW  27997539

Query  241       FTLFKTWFSLLANLAHANLCDLITSLVCLLLLLPTKELNNRFKNKLK  287
                 FTLFKTWFSLLANLAHANLCDLITSLVCLLLLLPTKELNNRFKNKLK
Sbjct  27997540  FTLFKTWFSLLANLAHANLCDLITSLVCLLLLLPTKELNNRFKNKLK  27997680




Range 2: 27999181 to 27999963

Score:945 bits(1929), Expect:0.0,
Method:,
Identities:261/261(100%), Positives:261/261(100%), Gaps:0/261(0%)

Query  438       CVLAVIIVVNLGGALRKFAEVPRMWRADRVDAAVWLVTMATSALVNTELGLLAGVLVSAF  497
                 CVLAVIIVVNLGGALRKFAEVPRMWRADRVDAAVWLVTMATSALVNTELGLLAGVLVSAF
Sbjct  27999181  CVLAVIIVVNLGGALRKFAEVPRMWRADRVDAAVWLVTMATSALVNTELGLLAGVLVSAF  27999360

Query  498       CVLGRTQRAQALELGRTQDGDHYEVLSAYRGLRSHPAVAVFRYPAPIYYANWSLFKKSLY  557
                 CVLGRTQRAQALELGRTQDGDHYEVLSAYRGLRSHPAVAVFRYPAPIYYANWSLFKKSLY
Sbjct  27999361  CVLGRTQRAQALELGRTQDGDHYEVLSAYRGLRSHPAVAVFRYPAPIYYANWSLFKKSLY  27999540

Query  558       RRAGLDPLKEKTRRVKFEKKLKRQNAERESVRKEDGEAAAGVTLAPEQKPLRGLVLDCGA  617
                 RRAGLDPLKEKTRRVKFEKKLKRQNAERESVRKEDGEAAAGVTLAPEQKPLRGLVLDCGA
Sbjct  27999541  RRAGLDPLKEKTRRVKFEKKLKRQNAERESVRKEDGEAAAGVTLAPEQKPLRGLVLDCGA  27999720

Query  618       VLFLDGAGVDALKEVRKDYGELGVAVVLAQCSPAVLDALRRGGYLPEKQGGDRRLTFFTI  677
                 VLFLDGAGVDALKEVRKDYGELGVAVVLAQCSPAVLDALRRGGYLPEKQGGDRRLTFFTI
Sbjct  27999721  VLFLDGAGVDALKEVRKDYGELGVAVVLAQCSPAVLDALRRGGYLPEKQGGDRRLTFFTI  27999900

Query  678       ADAVRHVQRASAPNGDDNGAC  698
                 ADAVRHVQRASAPNGDDNGAC
Sbjct  27999901  ADAVRHVQRASAPNGDDNGAC  27999963




Range 3: 27998663 to 27999112

Score:545 bits(1112), Expect:0.0,
Method:,
```

Identities:150/150(100%), Positives:150/150(100%), Gaps:0/150(0%)

```
Query  288       APIPFELFVVVIATLASHFGRFNADYGSDVAGEIPTGFLPPRLPAWSLIPAVAVDAFSVA  347
                 APIPFELFVVVIATLASHFGRFNADYGSDVAGEIPTGFLPPRLPAWSLIPAVAVDAFSVA
Sbjct  27998663  APIPFELFVVVIATLASHFGRFNADYGSDVAGEIPTGFLPPRLPAWSLIPAVAVDAFSVA  27998842

Query  348       LVGFAITVSLSEMFAKKHGYRVDANQEMYAIGFCNILPSFFRCFATSAALTKTLVKESTG  407
                 LVGFAITVSLSEMFAKKHGYRVDANQEMYAIGFCNILPSFFRCFATSAALTKTLVKESTG
Sbjct  27998843  LVGFAITVSLSEMFAKKHGYRVDANQEMYAIGFCNILPSFFRCFATSAALTKTLVKESTG  27999022

Query  408       CQTQVSGLVSAVVLLLVLLVIAPLFYSLQK  437
                 CQTQVSGLVSAVVLLLVLLVIAPLFYSLQK
Sbjct  27999023  CQTQVSGLVSAVVLLLVLLVIAPLFYSLQK  27999112
```

EB Big-belly seahorse *slc26a2*

Query: sulfate transporter [Hippocampus zosterae] Query ID: XP_051927998.1 Length: 706

>Hippocampus zosterae strain Florida chromosome 1, ASM2543408v3, whole genome shotgun sequence
Sequence ID: NC_067451.1 Length: 42937977
Range 1: 16134151 to 16135047

Score:995 bits(2256), Expect:0.0,
Method:,
Identities:299/299(100%), Positives:299/299(100%), Gaps:0/299(0%)

```
Query  1         MMPHEDACCTASGDRTKNEHYEPLILERVPEEAGQSCQVVLSRYFRENCSCSPQKIKSKI  60
                 MMPHEDACCTASGDRTKNEHYEPLILERVPEEAGQSCQVVLSRYFRENCSCSPQKIKSKI
Sbjct  16135047  MMPHEDACCTASGDRTKNEHYEPLILERVPEEAGQSCQVVLSRYFRENCSCSPQKIKSKI  16134868

Query  61        LGFVPILRWLPHYQLRDWLLGDVMSGLIVGILLVPQSIAYSLLAGQDPIYGLYTSFFSSI  120
                 LGFVPILRWLPHYQLRDWLLGDVMSGLIVGILLVPQSIAYSLLAGQDPIYGLYTSFFSSI
Sbjct  16134867  LGFVPILRWLPHYQLRDWLLGDVMSGLIVGILLVPQSIAYSLLAGQDPIYGLYTSFFSSI  16134688

Query  121       IYTLLGTSKHISVGIFGVLCLLVGQVVDRELAVAGYLVDGGVGGNGSANVMGSNNSMVVA  180
                 IYTLLGTSKHISVGIFGVLCLLVGQVVDRELAVAGYLVDGGVGGNGSANVMGSNNSMVVA
Sbjct  16134687  IYTLLGTSKHISVGIFGVLCLLVGQVVDRELAVAGYLVDGGVGGNGSANVMGSNNSMVVA  16134508

Query  181       CDRSCYAITVGATVTFIAGVYQVLMAIFQVGFVSVYLSNSLLSGFATGASLTILTSQVKY  240
                 CDRSCYAITVGATVTFIAGVYQVLMAIFQVGFVSVYLSNSLLSGFATGASLTILTSQVKY
Sbjct  16134507  CDRSCYAITVGATVTFIAGVYQVLMAIFQVGFVSVYLSNSLLSGFATGASLTILTSQVKY  16134328

Query  241       MLGLKITRPQGWFTFFKTWFGVLSNLGKSNLCDLITSLVCLLLLLPTKELNDHYKAKLK  299
                 MLGLKITRPQGWFTFFKTWFGVLSNLGKSNLCDLITSLVCLLLLLPTKELNDHYKAKLK
Sbjct  16134327  MLGLKITRPQGWFTFFKTWFGVLSNLGKSNLCDLITSLVCLLLLLPTKELNDHYKAKLK  16134151
```

Range 2: 16132095 to 16132865

Score:847 bits(1920), Expect:0.0,
Method:,
Identities:257/257(100%), Positives:257/257(100%), Gaps:0/257(0%)

```
Query  450       CVLAVIIVVNLRGALRKFADVPSMWRTNRIDAAVWLITMATSALINTELGLLVGILVSAF  509
                 CVLAVIIVVNLRGALRKFADVPSMWRTNRIDAAVWLITMATSALINTELGLLVGILVSAF
Sbjct  16132865  CVLAVIIVVNLRGALRKFADVPSMWRTNRIDAAVWLITMATSALINTELGLLVGILVSAF  16132686

Query  510       CVLGRTQRAQAQELGRTAVRDHYEVLSAYAGLRAHPGVAVVRYPAPIYYANQSLFKKSLY  569
                 CVLGRTQRAQAQELGRTAVRDHYEVLSAYAGLRAHPGVAVVRYPAPIYYANQSLFKKSLY
Sbjct  16132685  CVLGRTQRAQAQELGRTAVRDHYEVLSAYAGLRAHPGVAVVRYPAPIYYANQSLFKKSLY  16132506

Query  570       RCAGLDPVKEKSRRIKFEKKLKRQNTEPELPCKEDGEGTAGIMLSLDQKPLRSLVLDCSA  629
                 RCAGLDPVKEKSRRIKFEKKLKRQNTEPELPCKEDGEGTAGIMLSLDQKPLRSLVLDCSA
Sbjct  16132505  RCAGLDPVKEKSRRIKFEKKLKRQNTEPELPCKEDGEGTAGIMLSLDQKPLRSLVLDCSA  16132326

Query  630       VFFLDSAGVNALKEVHSDYAELGVAVVLARCNTAVLDALRRGGYLSEKKGEDDRMTFFTI  689
                 VFFLDSAGVNALKEVHSDYAELGVAVVLARCNTAVLDALRRGGYLSEKKGEDDRMTFFTI
Sbjct  16132325  VFFLDSAGVNALKEVHSDYAELGVAVVLARCNTAVLDALRRGGYLSEKKGEDDRMTFFTI  16132146

Query  690       ADAVHHLQRAFVPNGTC  706
                 ADAVHHLQRAFVPNGTC
Sbjct  16132145  ADAVHHLQRAFVPNGTC  16132095
```

Range 3: 16132945 to 16133394

Score:495 bits(1119), Expect:0.0,
Method:,
Identities:150/150(100%), Positives:150/150(100%), Gaps:0/150(0%)

```
Query  300       APIPFELFVVIIATLASHFGRFNGDYGSDVAGEIPTGFLPPQLPAWSLIPSVAMDAFSIA  359
                 APIPFELFVVIIATLASHFGRFNGDYGSDVAGEIPTGFLPPQLPAWSLIPSVAMDAFSIA
Sbjct  16133394  APIPFELFVVIIATLASHFGRFNGDYGSDVAGEIPTGFLPPQLPAWSLIPSVAMDAFSIA  16133215

Query  360       LVGFAITVSLSEMFAKKHGYRVDANQEMYAIGFCNILPSFFRCFTTSAALTKTLVKESTG  419
                 LVGFAITVSLSEMFAKKHGYRVDANQEMYAIGFCNILPSFFRCFTTSAALTKTLVKESTG
Sbjct  16133214  LVGFAITVSLSEMFAKKHGYRVDANQEMYAIGFCNILPSFFRCFTTSAALTKTLVKESTG  16133035
```

```
Query  420       CQTQVSGLVSAVVLLLVLLLIAPLFYSLQK  449
                 CQTQVSGLVSAVVLLLVLLLIAPLFYSLQK
Sbjct  16133034  CQTQVSGLVSAVVLLLVLLLIAPLFYSLQK  16132945
```

EC Mudskipper *slc26a2*

Query: sulfate transporter isoform X2 [Boleophthalmus pectinirostris] Query ID: XP_020781126.1 Length: 704

>Boleophthalmus pectinirostris isolate CB_2022bp ecotype Guang Dong unplaced genomic scaffold, ASM2622593v1 HiC_scaffold_23, whole genome shotgun sequence
Sequence ID: NW_026571207.1 Length: 39819760
Range 1: 34592098 to 34592892

Score:953 bits(1946), Expect:0.0,
Method:,
Identities:262/265(99%), Positives:262/265(98%), Gaps:0/265(0%)

```
Query  440       LFYSLQKCVLAVIIVVNLRGAMRKFLDVPRMWRANRIDASIWLITMATSALVNTELGLLV  499
                 LF SL  CVLAVIIVVNLRGAMRKFLDVPRMWRANRIDASIWLITMATSALVNTELGLLV
Sbjct  34592892  LFLSLLRCVLAVIIVVNLRGAMRKFLDVPRMWRANRIDASIWLITMATSALVNTELGLLV  34592713

Query  500       GVLVSAFCVLGRTQQARANELGRASDREHYEDLKEYLNLHKQDGVPVFRYNSPIYYANQS  559
                 GVLVSAFCVLGRTQQARANELGRASDREHYEDLKEYLNLHKQDGVPVFRYNSPIYYANQS
Sbjct  34592712  GVLVSAFCVLGRTQQARANELGRASDREHYEDLKEYLNLHKQDGVPVFRYNSPIYYANQS  34592533

Query  560       LFKKSLYKSAGLDPVREIARRLKESKKKLAAEAKVLQNNENGKGVTVNLMLEDKAEPKIS  619
                 LFKKSLYKSAGLDPVREIARRLKESKKKLAAEAKVLQNNENGKGVTVNLMLEDKAEPKIS
Sbjct  34592532  LFKKSLYKSAGLDPVREIARRLKESKKKLAAEAKVLQNNENGKGVTVNLMLEDKAEPKIS  34592353

Query  620       SLVLDCSSVLFVDTAGVSALKEVRKDYAEVGVTIVLARCSTSVLDALEKGGFFPENQPNK  679
                 SLVLDCSSVLFVDTAGVSALKEVRKDYAEVGVTIVLARCSTSVLDALEKGGFFPENQPNK
Sbjct  34592352  SLVLDCSSVLFVDTAGVSALKEVRKDYAEVGVTIVLARCSTSVLDALEKGGFFPENQPNK  34592173

Query  680       MVFFTIDDAVHFVQNLLVSNGSYEI  704
                 MVFFTIDDAVHFVQNLLVSNGSYEI
Sbjct  34592172  MVFFTIDDAVHFVQNLLVSNGSYEI  34592098
```

Range 2: 34602079 to 34602678

Score:734 bits(1499), Expect:0.0,
Method:,
Identities:200/200(100%), Positives:200/200(100%), Gaps:0/200(0%)

```
Query  1         MQDDDCSPESHLHQPLILERVEKEPPKTWKNILSQRFKKHCTCSSQKVKSKILGFVPILT  60
                 MQDDDCSPESHLHQPLILERVEKEPPKTWKNILSQRFKKHCTCSSQKVKSKILGFVPILT
Sbjct  34602678  MQDDDCSPESHLHQPLILERVEKEPPKTWKNILSQRFKKHCTCSSQKVKSKILGFVPILT  34602499

Query  61        WLPRYQLKNWILGDVMSGLIVGILLAPQSIAYSLLASQDPIYGLYTSFFASIIYAILGTS  120
                 WLPRYQLKNWILGDVMSGLIVGILLAPQSIAYSLLASQDPIYGLYTSFFASIIYAILGTS
Sbjct  34602498  WLPRYQLKNWILGDVMSGLIVGILLAPQSIAYSLLASQDPIYGLYTSFFASIIYAILGTS  34602319

Query  121       RHISVGIFGVLCLLVGQVVDRELALAGYPPESSSSGSINISSELVSGLSTNTSTGEGCDR  180
                 RHISVGIFGVLCLLVGQVVDRELALAGYPPESSSSGSINISSELVSGLSTNTSTGEGCDR
Sbjct  34602318  RHISVGIFGVLCLLVGQVVDRELALAGYPPESSSSGSINISSELVSGLSTNTSTGEGCDR  34602139

Query  181       SCYAITVGTTVTFTAGVYQV  200
                 SCYAITVGTTVTFTAGVYQV
Sbjct  34602138  SCYAITVGTTVTFTAGVYQV  34602079
```

Range 3: 34596691 to 34597140

Score:551 bits(1123), Expect:5e-160,
Method:,
Identities:150/150(100%), Positives:150/150(100%), Gaps:0/150(0%)

```
Query  297       APIPFELFVVIIATVASHFGHFNTEYGSGVSGNIPTGFMPPQLPSWSLIPNVAVDAFSIA  356
                 APIPFELFVVIIATVASHFGHFNTEYGSGVSGNIPTGFMPPQLPSWSLIPNVAVDAFSIA
Sbjct  34597140  APIPFELFVVIIATVASHFGHFNTEYGSGVSGNIPTGFMPPQLPSWSLIPNVAVDAFSIA  34596961

Query  357       IVGFAITVSLSEMFAKKHGYTVDANQEMYAIGFCNILPSFFHCFTTSAALTKTLVKESTG  416
                 IVGFAITVSLSEMFAKKHGYTVDANQEMYAIGFCNILPSFFHCFTTSAALTKTLVKESTG
Sbjct  34596960  IVGFAITVSLSEMFAKKHGYTVDANQEMYAIGFCNILPSFFHCFTTSAALTKTLVKESTG  34596781

Query  417       CQTQISGLVSGLVLLLVLLVIAPLFYSLQK  446
                 CQTQISGLVSGLVLLLVLLVIAPLFYSLQK
Sbjct  34596780  CQTQISGLVSGLVLLLVLLVIAPLFYSLQK  34596691
```

Range 4: 34598870 to 34599163

Score:364 bits(742), Expect:7e-104,
Method:,
Identities:98/98(100%), Positives:98/98(100%), Gaps:0/98(0%)

```
Query  199       QVLMGIFQVGFVSVYLSDSMLSGFATGASLTIFTSQFKYMLGLKIPRAQGWFTLFKTWVA  258
                 QVLMGIFQVGFVSVYLSDSMLSGFATGASLTIFTSQFKYMLGLKIPRAQGWFTLFKTWVA
Sbjct  34599163  QVLMGIFQVGFVSVYLSDSMLSGFATGASLTIFTSQFKYMLGLKIPRAQGWFTLFKTWVA  34598984

Query  259       IFSNLGQTNLCDLITSLVCLAVLIPTKEINDRFKSKLK  296
                 IFSNLGQTNLCDLITSLVCLAVLIPTKEINDRFKSKLK
Sbjct  34598983  IFSNLGQTNLCDLITSLVCLAVLIPTKEINDRFKSKLK  34598870
```

ED Indian glassy fish *slc26a2*

Query: sulfate transporter-like [Parambassis ranga] Query ID: XP_028272633.1 Length: 723

>Parambassis ranga chromosome 10, fParRan2.1, whole genome shotgun sequence
Sequence ID: NC_041031.1 Length: 24485510
Range 1: 21415358 to 21416167

Score:993 bits(2028), Expect:0.0,
Method:,
Identities:270/270(100%), Positives:270/270(100%), Gaps:0/270(0%)

```
Query  454       CVLAVIIIVNLRGALQKFTDIPRMWRANRIDASIWLITMVSSALVNTEQGLLVGILVSAF  513
                 CVLAVIIIVNLRGALQKFTDIPRMWRANRIDASIWLITMVSSALVNTEQGLLVGILVSAF
Sbjct  21416167  CVLAVIIIVNLRGALQKFTDIPRMWRANRIDASIWLITMVSSALVNTEQGLLVGILVSAF  21415988

Query  514       CVLGRTQKAQVVELGRVTTREHYEDMSSYRGLQTHPGVAVFRYVAPIYYANQSLFKKSLY  573
                 CVLGRTQKAQVVELGRVTTREHYEDMSSYRGLQTHPGVAVFRYVAPIYYANQSLFKKSLY
Sbjct  21415987  CVLGRTQKAQVVELGRVTTREHYEDMSSYRGLQTHPGVAVFRYVAPIYYANQSLFKKSLY  21415808

Query  574       KCVGLDPVEEKARRIKFTKKKRQKEAAENQSKDKEDACKENDADASATVTLMLDKKSFHS  633
                 KCVGLDPVEEKARRIKFTKKKRQKEAAENQSKDKEDACKENDADASATVTLMLDKKSFHS
Sbjct  21415807  KCVGLDPVEEKARRIKFTKKKRQKEAAENQSKDKEDACKENDADASATVTLMLDKKSFHS  21415628

Query  634       VVIDSSAILFLDTAGVDALKEVHKDYAEFGVRVVLAQCNTSVLDTLERGGYYTDKKGEGV  693
                 VVIDSSAILFLDTAGVDALKEVHKDYAEFGVRVVLAQCNTSVLDTLERGGYYTDKKGEGV
Sbjct  21415627  VVIDSSAILFLDTAGVDALKEVHKDYAEFGVRVVLAQCNTSVLDTLERGGYYTDKKGEGV  21415448

Query  694       GENKVFFTIADAVRYVQSLCTPNGDYDSKC  723
                 GENKVFFTIADAVRYVQSLCTPNGDYDSKC
Sbjct  21415447  GENKVFFTIADAVRYVQSLCTPNGDYDSKC  21415358
```

Range 2: 21417817 to 21418443

Score:760 bits(1551), Expect:0.0,
Method:,
Identities:208/209(99%), Positives:208/209(99%), Gaps:0/209(0%)

```
Query  1         MLNGDDCCTAAEERGASDQQQQQQPLILERIEKETGKTWKTIASHRLKKHCSCTPAKVKS  60
                 MLNGDDCCTAAEERGASDQQQQQQPLILERIEKETGKTWKTIASHRLKKHCSCTPAKVKS
Sbjct  21418443  MLNGDDCCTAAEERGASDQQQQQQPLILERIEKETGKTWKTIASHRLKKHCSCTPAKVKS  21418264

Query  61        KLLGFFPILKWLPHYKLREWLLGDAMSGLIVGILLVPQSIAYSLLASQDPIYGLYTSFFA  120
                 KLLGFFPILKWLPHYKLREWLLGDAMSGLIVGILLVPQSIAYSLLASQDPIYGLYTSFFA
Sbjct  21418263  KLLGFFPILKWLPHYKLREWLLGDAMSGLIVGILLVPQSIAYSLLASQDPIYGLYTSFFA  21418084

Query  121       SIIYALLGTSRHVSVGMFGVLCLLVGQVVDRELALAGYLPESSSSNSSDVLVTGQGNTSS  180
                 SIIYALLGTSRHVSVGMFGVLCLLVGQVVDRELALAGYLPESSSSNSSDVLVTGQGNTSS
Sbjct  21418083  SIIYALLGTSRHVSVGMFGVLCLLVGQVVDRELALAGYLPESSSSNSSDVLVTGQGNTSS  21417904

Query  181       TGIDCDRSCYAITVGATVTFTAGVYQVLM  209
                 TGIDCDRSCYAITVGATVTFTAGVYQV M
Sbjct  21417903  TGIDCDRSCYAITVGATVTFTAGVYQVNM  21417817
```

Range 3: 21417453 to 21417752

Score:364 bits(742), Expect:0.0,
Method:,
Identities:99/100(99%), Positives:100/100(100%), Gaps:0/100(0%)

```
Query  204       VYQVLMGFLQVGFVSVYLSDSLLSGFATGASLTILTSQFKYLLGLKIPRPQGWFSLFKTW  263
                 V+QVLMGFLQVGFVSVYLSDSLLSGFATGASLTILTSQFKYLLGLKIPRPQGWFSLFKTW
Sbjct  21417752  VFQVLMGFLQVGFVSVYLSDSLLSGFATGASLTILTSQFKYLLGLKIPRPQGWFSLFKTW  21417573

Query  264       YSLLINLGNTNICDLITSLVCLALLIPTKELNDRIKSKLK  303
                 YSLLINLGNTNICDLITSLVCLALLIPTKELNDRIKSKLK
Sbjct  21417572  YSLLINLGNTNICDLITSLVCLALLIPTKELNDRIKSKLK  21417453
```

Range 4: 21416799 to 21417248

Score:553 bits(1127), Expect:1e-160,
Method:,
Identities:150/150(100%), Positives:150/150(100%), Gaps:0/150(0%)

```
Query  304       APIPFELFVVIIATLASHFGHFNVDYGSDIAGHIPTGFLPPQLPAWSLIPNLAVDAFSIA  363
                 APIPFELFVVIIATLASHFGHFNVDYGSDIAGHIPTGFLPPQLPAWSLIPNLAVDAFSIA
Sbjct  21417248  APIPFELFVVIIATLASHFGHFNVDYGSDIAGHIPTGFLPPQLPAWSLIPNLAVDAFSIA  21417069

Query  364       LVGFAITISLSEMFAKKHGYTVDPNQEMYAIGFCNILPSFFRCFTTSAALTKTLVKESTG  423
                 LVGFAITISLSEMFAKKHGYTVDPNQEMYAIGFCNILPSFFRCFTTSAALTKTLVKESTG
Sbjct  21417068  LVGFAITISLSEMFAKKHGYTVDPNQEMYAIGFCNILPSFFRCFTTSAALTKTLVKESTG  21416889

Query  424       CQTQMSGLVSALVLLLVLLVIAPLFYSLQK  453
                 CQTQMSGLVSALVLLLVLLVIAPLFYSLQK
Sbjct  21416888  CQTQMSGLVSALVLLLVLLVIAPLFYSLQK  21416799
```

EE Clown anemonefish *slc26a2*

Query: sulfate transporter [Amphiprion ocellaris] Query ID: XP_023143603.2 Length: 723

>Amphiprion ocellaris isolate individual 3 ecotype Okinawa chromosome 13, ASM2253959v1, whole genome shotgun sequence
Sequence ID: NC_072778.1 Length: 36729457
Range 1: 4571617 to 4572435

Score:1007 bits(2056), Expect:0.0,
Method:,
Identities:273/273(100%), Positives:273/273(100%), Gaps:0/273(0%)

```
Query  451      CVLAVIIVVNLRGALRKFTDIPRMWRANRIDTSIWLVTMATSALVNTELGLLVGVLVSAF  510
                CVLAVIIVVNLRGALRKFTDIPRMWRANRIDTSIWLVTMATSALVNTELGLLVGVLVSAF
Sbjct  4572435  CVLAVIIVVNLRGALRKFTDIPRMWRANRIDTSIWLVTMATSALVNTELGLLVGVLVSAF  4572256

Query  511      CVLGRTQQARVLELGRATTREHYEGLSCYRGLQTHPGVAVFRYEAPIYYANQSLFKKSLY  570
                CVLGRTQQARVLELGRATTREHYEGLSCYRGLQTHPGVAVFRYEAPIYYANQSLFKKSLY
Sbjct  4572255  CVLGRTQQARVLELGRATTREHYEGLSCYRGLQTHPGVAVFRYEAPIYYANQSLFKKSLY  4572076

Query  571      KCVGLDPVKEKSRRIKFKKNKRQEEAARVLNVKSDEKEDACKDNGAAVTLMLDQNSSHSL  630
                KCVGLDPVKEKSRRIKFKKNKRQEEAARVLNVKSDEKEDACKDNGAAVTLMLDQNSSHSL
Sbjct  4572075  KCVGLDPVKEKSRRIKFKKNKRQEEAARVLNVKSDEKEDACKDNGAAVTLMLDQNSSHSL  4571896

Query  631      RNVVIDCSAVLFLDTAGMNVLKEVHKDYAELGVKVVLAQCNTSVLDTLERGGYCPDKNGA  690
                RNVVIDCSAVLFLDTAGMNVLKEVHKDYAELGVKVVLAQCNTSVLDTLERGGYCPDKNGA
Sbjct  4571895  RNVVIDCSAVLFLDTAGMNVLKEVHKDYAELGVKVVLAQCNTSVLDTLERGGYCPDKNGA  4571716

Query  691      KTGENKMIFFTIADAVHYIQSLRTANGDYDTKC  723
                KTGENKMIFFTIADAVHYIQSLRTANGDYDTKC
Sbjct  4571715  KTGENKMIFFTIADAVHYIQSLRTANGDYDTKC  4571617
```

Range 2: 4576175 to 4576786

Score:740 bits(1511), Expect:0.0,
Method:,
Identities:204/204(100%), Positives:204/204(100%), Gaps:0/204(0%)

```
Query  1        MAHGNGCLTVAAEDGAEQQQPLILERVERETAKKWQTVVSQRLKKHCSCTSKKAKSKILG  60
                MAHGNGCLTVAAEDGAEQQQPLILERVERETAKKWQTVVSQRLKKHCSCTSKKAKSKILG
Sbjct  4576786  MAHGNGCLTVAAEDGAEQQQPLILERVERETAKKWQTVVSQRLKKHCSCTSKKAKSKILG  4576607

Query  61       FIPILKWLPRYQLRDWLLGDVMSGVIVGILLVPQSIAYSLLASQDPIYGLYTSFFASIIY  120
                FIPILKWLPRYQLRDWLLGDVMSGVIVGILLVPQSIAYSLLASQDPIYGLYTSFFASIIY
Sbjct  4576606  FIPILKWLPRYQLRDWLLGDVMSGVIVGILLVPQSIAYSLLASQDPIYGLYTSFFASIIY  4576427

Query  121      ALLGTSRHISVGIFGVLCLLVGQVVDRELALAGYLTESSSISSNSSDILLAGPGNGSVGV  180
                ALLGTSRHISVGIFGVLCLLVGQVVDRELALAGYLTESSSISSNSSDILLAGPGNGSVGV
Sbjct  4576426  ALLGTSRHISVGIFGVLCLLVGQVVDRELALAGYLTESSSISSNSSDILLAGPGNGSVGV  4576247

Query  181      GCDRSCYAITVGATVTFTAGVYQV  204
                GCDRSCYAITVGATVTFTAGVYQV
Sbjct  4576246  GCDRSCYAITVGATVTFTAGVYQV  4576175
```

Range 3: 4574160 to 4574615

Score:553 bits(1128), Expect:3e-161,
Method:,
Identities:151/152(99%), Positives:151/152(99%), Gaps:0/152(0%)

```
Query  299      LKAPIPFELFVVIIATLASHFGHFNTDYGSDVAGDIPTGFLPPQLPLWSLIPNVAVDAFS  358
                L APIPFELFVVIIATLASHFGHFNTDYGSDVAGDIPTGFLPPQLPLWSLIPNVAVDAFS
Sbjct  4574615  LQAPIPFELFVVIIATLASHFGHFNTDYGSDVAGDIPTGFLPPQLPLWSLIPNVAVDAFS  4574436

Query  359      IAIVGFAITVSLSEMFAKKHGYTVDANQEMYAIGFCNILPSFFHCFTTSAALTKTLVKES  418
                IAIVGFAITVSLSEMFAKKHGYTVDANQEMYAIGFCNILPSFFHCFTTSAALTKTLVKES
Sbjct  4574435  IAIVGFAITVSLSEMFAKKHGYTVDANQEMYAIGFCNILPSFFHCFTTSAALTKTLVKES  4574256

Query  419      TGCQTQISGLISALILLLVLLVIAPLFYSLQK  450
                TGCQTQISGLISALILLLVLLVIAPLFYSLQK
Sbjct  4574255  TGCQTQISGLISALILLLVLLVIAPLFYSLQK  4574160
```

Range 4: 4575127 to 4575420

Score:359 bits(732), Expect:8e-103,
Method:,
Identities:98/98(100%), Positives:98/98(100%), Gaps:0/98(0%)

```
Query  203      QVLMGLLQIGFVSVYLSDSLLSGFATGASLTILTSQFKYLLGLKIPRPQGWFTLFKTWYS  262
                QVLMGLLQIGFVSVYLSDSLLSGFATGASLTILTSQFKYLLGLKIPRPQGWFTLFKTWYS
Sbjct  4575420  QVLMGLLQIGFVSVYLSDSLLSGFATGASLTILTSQFKYLLGLKIPRPQGWFTLFKTWYS  4575241

Query  263      LLTNLGNTNICDLVTSLVCLAILVPTKELNDRFKSKLK  300
                LLTNLGNTNICDLVTSLVCLAILVPTKELNDRFKSKLK
Sbjct  4575240  LLTNLGNTNICDLVTSLVCLAILVPTKELNDRFKSKLK  4575127
```

EF Jewelled blenny *slc26a2*

Query: sulfate transporter-like [Salarias fasciatus] Query ID: XP_029966977.1 Length: 723

>Salarias fasciatus chromosome 2, fSalaFa1.1, whole genome shotgun sequence
Sequence ID: NC_043746.1 Length: 35747288
Range 1: 26208814 to 26209638

Score:999 bits(2040), Expect:0.0,
Method:,
Identities:273/275(99%), Positives:274/275(99%), Gaps:0/275(0%)

```
Query  449       QKCVLAVIIVVNLRGALQKFADIPKMWRSNRVDASIWLITMATSALVNTELGLLVGVLVS  508
                 + CVLAVIIVVNLRGALQKFADIPKMWRSNRVDASIWLITMATSALVNTELGLLVGVLVS
Sbjct  26208814  HRCVLAVIIVVNLRGALQKFADIPKMWRSNRVDASIWLITMATSALVNTELGLLVGVLVS  26208993

Query  509       AYCVLGRTQRVQVVELGRAGDREHYEDLSSYHGLQRHPGVAVFKYQAPIYYANQSLFKKS  568
                 AYCVLGRTQRVQVVELGRAGDREHYEDLSSYHGLQRHPGVAVFKYQAPIYYANQSLFKKS
Sbjct  26208994  AYCVLGRTQRVQVVELGRAGDREHYEDLSSYHGLQRHPGVAVFKYQAPIYYANQSLFKKS  26209173

Query  569       LYKSVGLNPVKEKARRIKFKKKQQQEAAGKSEEKEDASRENDVEAAAASVTLMHDKTSSR  628
                 LYKSVGLNPVKEKARRIKFKKKQQQEAAGKSEEKEDASRENDVEAAAASVTLMHDKTSSR
Sbjct  26209174  LYKSVGLNPVKEKARRIKFKKKQQQEAAGKSEEKEDASRENDVEAAAASVTLMHDKTSSR  26209353

Query  629       SFHSLVMDCGAVLFLDTAGVNALKEIHKDYGELGVKVFLARCNPSVLDTLERGGYYPDGK  688
                 SFHSLVMDCGAVLFLDTAGVNALKEIHKDYGELGVKVFLARCNPSVLDTLERGGYYPDGK
Sbjct  26209354  SFHSLVMDCGAVLFLDTAGVNALKEIHKDYGELGVKVFLARCNPSVLDTLERGGYYPDGK  26209533

Query  689       GGNVGENKMVFFTIPDAVRYAQSLGAPNGNPDSRC  723
                 GGNVGENKMVFFTIPDAVRYAQSLGAPNGNPDSRC
Sbjct  26209534  GGNVGENKMVFFTIPDAVRYAQSLGAPNGNPDSRC  26209638
```

Range 2: 26205161 to 26205772

Score:750 bits(1530), Expect:0.0,
Method:,
Identities:204/204(100%), Positives:204/204(100%), Gaps:0/204(0%)

```
Query  1         MLHADDGCTAAEDGGETDQHQPLILERVKKEADKNWKTVVSDRLKKHCTCTAQKAKSKIL  60
                 MLHADDGCTAAEDGGETDQHQPLILERVKKEADKNWKTVVSDRLKKHCTCTAQKAKSKIL
Sbjct  26205161  MLHADDGCTAAEDGGETDQHQPLILERVKKEADKNWKTVVSDRLKKHCTCTAQKAKSKIL  26205340

Query  61        GFVPILSWLPRYQCRDWILGDAMSGLIVGILLVPQSIAYSLLAGQDPIYGLYTSFFASII  120
                 GFVPILSWLPRYQCRDWILGDAMSGLIVGILLVPQSIAYSLLAGQDPIYGLYTSFFASII
Sbjct  26205341  GFVPILSWLPRYQCRDWILGDAMSGLIVGILLVPQSIAYSLLAGQDPIYGLYTSFFASII  26205520

Query  121       YAILGSSRHISVGIFGVLCLLVGQVVDRELAVAGYLAERSSVGIGNFSEMSDQGNSSVEI  180
                 YAILGSSRHISVGIFGVLCLLVGQVVDRELAVAGYLAERSSVGIGNFSEMSDQGNSSVEI
Sbjct  26205521  YAILGSSRHISVGIFGVLCLLVGQVVDRELAVAGYLAERSSVGIGNFSEMSDQGNSSVEI  26205700

Query  181       QCDKSCYAITVGATVTFTAGVYQV  204
                 QCDKSCYAITVGATVTFTAGVYQV
Sbjct  26205701  QCDKSCYAITVGATVTFTAGVYQV  26205772
```

Range 3: 26207164 to 26207613

Score:547 bits(1116), Expect:6e-160,
Method:,
Identities:150/150(100%), Positives:150/150(100%), Gaps:0/150(0%)

```
Query  301       APIPFELFVVIIATVASHFGRFNTEYGSGVAGDIPTGFLPPQLPSWSLIPNVAVDAFSIA  360
                 APIPFELFVVIIATVASHFGRFNTEYGSGVAGDIPTGFLPPQLPSWSLIPNVAVDAFSIA
Sbjct  26207164  APIPFELFVVIIATVASHFGRFNTEYGSGVAGDIPTGFLPPQLPSWSLIPNVAVDAFSIA  26207343

Query  361       IVGFAITVSLSEMFAKKHGYTVDANQEMYAIGFCNILPSFFRCFTTSAALTKTLVKESTG  420
                 IVGFAITVSLSEMFAKKHGYTVDANQEMYAIGFCNILPSFFRCFTTSAALTKTLVKESTG
Sbjct  26207344  IVGFAITVSLSEMFAKKHGYTVDANQEMYAIGFCNILPSFFRCFTTSAALTKTLVKESTG  26207523
```

```
Query  421       CQTQVSGLVSGLVLLLVLLVIAPLFYSLQK  450
                 CQTQVSGLVSGLVLLLVLLVIAPLFYSLQK
Sbjct  26207524  CQTQVSGLVSGLVLLLVLLVIAPLFYSLQK  26207613
```

Range 4: 26205954 to 26206247

Score:359 bits(732), Expect:4e-103,
Method:,
Identities:98/98(100%), Positives:98/98(100%), Gaps:0/98(0%)

```
Query  203       QVLMGLLQVGFVSVYLSDSLLSGFATGASLTILTSQVKYLLGLKIPRPQGWFTLFKTWYS  262
                 QVLMGLLQVGFVSVYLSDSLLSGFATGASLTILTSQVKYLLGLKIPRPQGWFTLFKTWYS
Sbjct  26205954  QVLMGLLQVGFVSVYLSDSLLSGFATGASLTILTSQVKYLLGLKIPRPQGWFTLFKTWYS  26206133

Query  263       LFNNIGKTNICDLVTSLVCLAVLIPAKEINNRFKAKLK  300
                 LFNNIGKTNICDLVTSLVCLAVLIPAKEINNRFKAKLK
Sbjct  26206134  LFNNIGKTNICDLVTSLVCLAVLIPAKEINNRFKAKLK  26206247
```

EG Bonti rainbowfish *slc26a2*

Query: unnamed protein product Query ID: lcl|Query_2439459 Length: 723

>Telmatherina bonti genome assembly, contig: atg001107l_1, whole genome shotgun sequence
Sequence ID: CAKOGH010001035.1 Length: 297109
Range 1: 40639 to 41466

Score:514 bits(1324), Expect:4e-159,
Method:Compositional matrix adjust.,
Identities:273/276(99%), Positives:274/276(99%), Gaps:0/276(0%)

```
Query  448    SLQKCVLAAIIVVNLRGALRKFIDLPRMWRVNCIDASIWLVTMATSAlvntelgllvgvl  507
              S  +CVLAAIIVVNLRGALRKFIDLPRMWRVNCIDASIWLVTMATSALVNTELGLLVGVL
Sbjct  40639  SFFRCVLAAIIVVNLRGALRKFIDLPRMWRVNCIDASIWLVTMATSALVNTELGLLVGVL  40818

Query  508    vsalcvlgRTQQAQVRELGRAATREHYEDMLSYHGLQKHPGVAIFRYEAPIYYANQTLFK  567
              VSALCVLGRTQQAQVRELGRAATREHYEDMLSYHGLQKHPGVAIFRYEAPIYYANQTLFK
Sbjct  40819  VSALCVLGRTQQAQVRELGRAATREHYEDMLSYHGLQKHPGVAIFRYEAPIYYANQTLFK  40998

Query  568    KSLYKCAGLDPVREKARHVKFKRSKEESGEIPKMKSEDRACKEDEAAATVTLMLGEKPSQ  627
              KSLYKCAGLDPVREKARHVKFKRSKEESGEIPKMKSEDRACKEDEAAATVTLMLGEKPSQ
Sbjct  40999  KSLYKCAGLDPVREKARHVKFKRSKEESGEIPKMKSEDRACKEDEAAATVTLMLGEKPSQ  41178

Query  628    KLRSLIIDCSAILFLDTSGVNALKEVRKDYSELGVEVLLTQCSTSVLDTLERGGYYSSEK  687
              KLRSLIIDCSAILFLDTSGVNALKEVRKDYSELGVEVLLTQCSTSVLDTLERGGYYSSEK
Sbjct  41179  KLRSLIIDCSAILFLDTSGVNALKEVRKDYSELGVEVLLTQCSTSVLDTLERGGYYSSEK  41358

Query  688    DGHVGQNKITFFTIEDAVHYIQSLSSPNGDFNNKI*  723
              DGHVGQNKITFFTIEDAVHYIQSLSSPNGDFNNKI*
Sbjct  41359  DGHVGQNKITFFTIEDAVHYIQSLSSPNGDFNNKI*  41466
```

Range 2: 36165 to 36779

Score:400 bits(1028), Expect:9e-120,
Method:Compositional matrix adjust.,
Identities:205/205(100%), Positives:205/205(100%), Gaps:0/205(0%)

```
Query  1      MHHGDESCPAEDDGTKTHIHQPLILERVEKETEKEWQTIVSHRLKKHCSCTPNKVKSKIL  60
              MHHGDESCPAEDDGTKTHIHQPLILERVEKETEKEWQTIVSHRLKKHCSCTPNKVKSKIL
Sbjct  36165  MHHGDESCPAEDDGTKTHIHQPLILERVEKETEKEWQTIVSHRLKKHCSCTPNKVKSKIL  36344

Query  61     DFVPILKWLPQYKLREWILGDIMSGVIVGILLVPQSIAYSLLAGQDPIYGLYTSFFASII  120
              DFVPILKWLPQYKLREWILGDIMSGVIVGILLVPQSIAYSLLAGQDPIYGLYTSFFASII
Sbjct  36345  DFVPILKWLPQYKLREWILGDIMSGVIVGILLVPQSIAYSLLAGQDPIYGLYTSFFASII  36524

Query  121    YALLGTSRHNSVGIFGVLCLLVGQVVDRELALAGYLTESSSLASNNSDVLLTGQGNGSSG  180
              YALLGTSRHNSVGIFGVLCLLVGQVVDRELALAGYLTESSSLASNNSDVLLTGQGNGSSG
Sbjct  36525  YALLGTSRHNSVGIFGVLCLLVGQVVDRELALAGYLTESSSLASNNSDVLLTGQGNGSSG  36704

Query  181    LVCDKSCYAItvgatvtftagvYQV  205
              LVCDKSCYAITVGATVTFTAGVYQV
Sbjct  36705  LVCDKSCYAITVGATVTFTAGVYQV  36779
```

Range 3: 37843 to 38298

Score:273 bits(699), Expect:1e-76,
Method:Compositional matrix adjust.,
Identities:150/152(99%), Positives:151/152(99%), Gaps:0/152(0%)

```
Query  300    LKAPIPFELFVVIIATLASHFGHFNTDYGSSVAGDIPTGFLPPQPPAWSLIPNVAVDAFS  359
               +APIPFELFVVIIATLASHFGHFNTDYGSSVAGDIPTGFLPPQPPAWSLIPNVAVDAFS
Sbjct  37843  FQAPIPFELFVVIIATLASHFGHFNTDYGSSVAGDIPTGFLPPQPPAWSLIPNVAVDAFS  38022
```

```
Query  360    IAIVGFAITVSLSEMFAKKHGYTVDANQEMYAIGFCNILPSFFRCFTTSAALLKTLVKES  419
              IAIVGFAITVSLSEMFAKKHGYTVDANQEMYAIGFCNILPSFFRCFTTSAALLKTLVKES
Sbjct  38023  IAIVGFAITVSLSEMFAKKHGYTVDANQEMYAIGFCNILPSFFRCFTTSAALLKTLVKES  38202

Query  420    TGCQTQvsglvsalvlllvllvIAPLFYSLQK  451
              TGCQTQVSGLVSALVLLLVLLVIAPLFYSLQK
Sbjct  38203  TGCQTQVSGLVSALVLLLVLLVIAPLFYSLQK  38298
```

Range 4: 36957 to 37253

Score:196 bits(499), Expect:9e-51,
Method:Compositional matrix adjust.,
Identities:98/99(99%), Positives:98/99(98%), Gaps:0/99(0%)

```
Query  204    QVLMGILQVGFVSVYLSDSLLSGFATGASLTILTSQLKYLLGLKIPRPQGWFTLFKIWYS  263
              QVLMGILQVGFVSVYLSDSLLSGFATGASLTILTSQLKYLLGLKIPRPQGWFTLFKIWYS
Sbjct  36957  QVLMGILQVGFVSVYLSDSLLSGFATGASLTILTSQLKYLLGLKIPRPQGWFTLFKIWYS  37136

Query  264    VLTNLGSTNICDLITSLVCLAILIPSKELNDRFKAKLKA  302
              VLTNLGSTNICDLITSLVCLAILIPSKELNDRFKAKLK
Sbjct  37137  VLTNLGSTNICDLITSLVCLAILIPSKELNDRFKAKLKV  37253
```

EH Japanese medaka *slc26a2*

Query: sulfate transporter [Oryzias latipes] Query ID: XP_011478862.1 Length: 717

>Oryzias latipes chromosome 10, ASM223467v1
Sequence ID: NC_019868.2 Length: 31218526
Range 1: 28349656 to 28350456

Score:976 bits(1993), Expect:0.0,
Method:,
Identities:267/267(100%), Positives:267/267(100%), Gaps:0/267(0%)

```
Query  451       CVLAVIIVVNLRGALRKFLDVPRMWRVNRVDASVWLITMGTSALVNTELGLLVGVLASAF  510
                 CVLAVIIVVNLRGALRKFLDVPRMWRVNRVDASVWLITMGTSALVNTELGLLVGVLASAF
Sbjct  28349656  CVLAVIIVVNLRGALRKFLDVPRMWRVNRVDASVWLITMGTSALVNTELGLLVGVLASAF  28349835

Query  511       FVLGRTQRAQILELGRADTREHYEDASSYRGLQVHPKVAVFRYAAPIYYANQSLFKKCLY  570
                 FVLGRTQRAQILELGRADTREHYEDASSYRGLQVHPKVAVFRYAAPIYYANQSLFKKCLY
Sbjct  28349836  FVLGRTQRAQILELGRADTREHYEDASSYRGLQVHPKVAVFRYAAPIYYANQSLFKKCLY  28350015

Query  571       KRIGLDPVKEKARRVKFSKSKHQEENGGKSNMKPEENRPEAVTLMLDQESSRKLHSLVID  630
                 KRIGLDPVKEKARRVKFSKSKHQEENGGKSNMKPEENRPEAVTLMLDQESSRKLHSLVID
Sbjct  28350016  KRIGLDPVKEKARRVKFSKSKHQEENGGKSNMKPEENRPEAVTLMLDQESSRKLHSLVID  28350195

Query  631       CSGVLFLDTAGVNALKEVLKDYGEVGIKVVLVQCNPSVLDSLERGGYFSSKNDGDVGKNN  690
                 CSGVLFLDTAGVNALKEVLKDYGEVGIKVVLVQCNPSVLDSLERGGYFSSKNDGDVGKNN
Sbjct  28350196  CSGVLFLDTAGVNALKEVLKDYGEVGIKVVLVQCNPSVLDSLERGGYFSSKNDGDVGKNN  28350375

Query  691       MIFFTISDGVQYAQSLATANGDFGSQG  717
                 MIFFTISDGVQYAQSLATANGDFGSQG
Sbjct  28350376  MIFFTISDGVQYAQSLATANGDFGSQG  28350456
```

Range 2: 28346282 to 28346893

Score:754 bits(1538), Expect:0.0,
Method:,
Identities:204/204(100%), Positives:204/204(100%), Gaps:0/204(0%)

```
Query  1         MLQEDGGCTAEPDRTEARLREPLILERAEGEPQTKWHVAVSQRLKKHCSCTPQKAKSKIL  60
                 MLQEDGGCTAEPDRTEARLREPLILERAEGEPQTKWHVAVSQRLKKHCSCTPQKAKSKIL
Sbjct  28346282  MLQEDGGCTAEPDRTEARLREPLILERAEGEPQTKWHVAVSQRLKKHCSCTPQKAKSKIL  28346461

Query  61        GFVPILQWLPRYQLRDWILGDVMSGVIVGILLVPQSIAYSLLASQDPIYGLYTSFFSSII  120
                 GFVPILQWLPRYQLRDWILGDVMSGVIVGILLVPQSIAYSLLASQDPIYGLYTSFFSSII
Sbjct  28346462  GFVPILQWLPRYQLRDWILGDVMSGVIVGILLVPQSIAYSLLASQDPIYGLYTSFFSSII  28346641

Query  121       YTLLGSSRHISVGIFGVLCLLVGQVVDRELALAGYLTEGGVSGDNATVFLSGWGNNSDGQ  180
                 YTLLGSSRHISVGIFGVLCLLVGQVVDRELALAGYLTEGGVSGDNATVFLSGWGNNSDGQ
Sbjct  28346642  YTLLGSSRHISVGIFGVLCLLVGQVVDRELALAGYLTEGGVSGDNATVFLSGWGNNSDGQ  28346821

Query  181       ICDRSCYAITVGATVTFTAGIYQV  204
                 ICDRSCYAITVGATVTFTAGIYQV
Sbjct  28346822  ICDRSCYAITVGATVTFTAGIYQV  28346893
```

Range 3: 28348496 to 28348951

Score:550 bits(1121), Expect:2e-160,
Method:,
Identities:151/152(99%), Positives:151/152(99%), Gaps:0/152(0%)

```
Query  299       LKAPIPFELFVVIIATLASHFGHFNAEYGSGVAGAIPTGFLPPQMPSWSLIPSVAVDAFS  358
                 L APIPFELFVVIIATLASHFGHFNAEYGSGVAGAIPTGFLPPQMPSWSLIPSVAVDAFS
Sbjct  28348496  LQAPIPFELFVVIIATLASHFGHFNAEYGSGVAGAIPTGFLPPQMPSWSLIPSVAVDAFS  28348675
```

```
Query  359       IAIVGFAITVSLSEMFAKKHGYKVDANQEMYAIGFCNILPSFFRCFTTSAALTKTLVKES  418
                 IAIVGFAITVSLSEMFAKKHGYKVDANQEMYAIGFCNILPSFFRCFTTSAALTKTLVKES
Sbjct  28348676  IAIVGFAITVSLSEMFAKKHGYKVDANQEMYAIGFCNILPSFFRCFTTSAALTKTLVKES  28348855

Query  419       TGCQTQISGLVTALVLLLVLLVIAPLFFSLQK  450
                 TGCQTQISGLVTALVLLLVLLVIAPLFFSLQK
Sbjct  28348856  TGCQTQISGLVTALVLLLVLLVIAPLFFSLQK  28348951
```

Range 4: 28347864 to 28348169

Score:360 bits(734), Expect:3e-103,
Method:,
Identities:99/102(97%), Positives:100/102(98%), Gaps:0/102(0%)

```
Query  199       AGIYQVLMGLLQIGFVSVYLSDSLLSGFATGASLTILTSQLKYLLGLKIPRPQGWFALFK  258
                 A  +QVLMGLLQIGFVSVYLSDSLLSGFATGASLTILTSQLKYLLGLKIPRPQGWFALFK
Sbjct  28347864  ASLFQVLMGLLQIGFVSVYLSDSLLSGFATGASLTILTSQLKYLLGLKIPRPQGWFALFK  28348043

Query  259       TWYGLLTNIGKTNICDLITSLLCLAVLIPAKELNDRFKAKLK  300
                 TWYGLLTNIGKTNICDLITSLLCLAVLIPAKELNDRFKAKLK
Sbjct  28348044  TWYGLLTNIGKTNICDLITSLLCLAVLIPAKELNDRFKAKLK  28348169
```

El Mangrove rivulus *slc26a2*

Query: sulfate transporter isoform X2 [Kryptolebias marmoratus] Query ID: XP_024864726.1 Length: 716

>Kryptolebias marmoratus isolate JLee-2015 linkage group LG9, ASM164957v2, whole genome shotgun sequence
Sequence ID: NC_051438.1 Length: 31381257
Range 1: 28605940 to 28606734

Score:970 bits(1981), Expect:0.0,
Method:,
Identities:265/265(100%), Positives:265/265(100%), Gaps:0/265(0%)

```
Query  452       CVLAVIIVVNLRGALRKFLDIPRMWRANRIDASIWLVTMATSALVNTELGLLVGVLVSAL  511
                 CVLAVIIVVNLRGALRKFLDIPRMWRANRIDASIWLVTMATSALVNTELGLLVGVLVSAL
Sbjct  28605940  CVLAVIIVVNLRGALRKFLDIPRMWRANRIDASIWLVTMATSALVNTELGLLVGVLVSAL  28606119

Query  512       CILGRTQKAQVLELGKAGSREHYEHLSSYHGLQTHSGVAVFRYEAPIYYANQSLFKKSLY  571
                 CILGRTQKAQVLELGKAGSREHYEHLSSYHGLQTHSGVAVFRYEAPIYYANQSLFKKSLY
Sbjct  28606120  CILGRTQKAQVLELGKAGSREHYEHLSSYHGLQTHSGVAVFRYEAPIYYANQSLFKKSLY  28606299

Query  572       KCVGLNPVKERAQCLKRRDMTKMKSGVKAWEENNEAALAVTLVLDEKSSRRVRSLVIDCS  631
                 KCVGLNPVKERAQCLKRRDMTKMKSGVKAWEENNEAALAVTLVLDEKSSRRVRSLVIDCS
Sbjct  28606300  KCVGLNPVKERAQCLKRRDMTKMKSGVKAWEENNEAALAVTLVLDEKSSRRVRSLVIDCS  28606479

Query  632       AILFLDTAGVNALKEVRKDYGEVGIDVILTQCNTSVLDTLERGGYYSSEKDGQVGKNKLI  691
                 AILFLDTAGVNALKEVRKDYGEVGIDVILTQCNTSVLDTLERGGYYSSEKDGQVGKNKLI
Sbjct  28606480  AILFLDTAGVNALKEVRKDYGEVGIDVILTQCNTSVLDTLERGGYYSSEKDGQVGKNKLI  28606659

Query  692       FFTITDAVHHVQSLSVPNGGCDNQS  716
                 FFTITDAVHHVQSLSVPNGGCDNQS
Sbjct  28606660  FFTITDAVHHVQSLSVPNGGCDNQS  28606734
```

Range 2: 28601804 to 28602415

Score:746 bits(1522), Expect:0.0,
Method:,
Identities:204/204(100%), Positives:204/204(100%), Gaps:0/204(0%)

```
Query  1         MDVSENDGCAPEEDGPETDLKQPLFLERVEKEPAKKWQTVVSNRLKKHCSCTTKKVKSKI  60
                 MDVSENDGCAPEEDGPETDLKQPLFLERVEKEPAKKWQTVVSNRLKKHCSCTTKKVKSKI
Sbjct  28601804  MDVSENDGCAPEEDGPETDLKQPLFLERVEKEPAKKWQTVVSNRLKKHCSCTTKKVKSKI  28601983

Query  61        LGFVPILKWLPNYKLRDWILGDSMSGLIVGILLVPQSIAYSLLASQDPIYGLYTSFFSSI  120
                 LGFVPILKWLPNYKLRDWILGDSMSGLIVGILLVPQSIAYSLLASQDPIYGLYTSFFSSI
Sbjct  28601984  LGFVPILKWLPNYKLRDWILGDSMSGLIVGILLVPQSIAYSLLASQDPIYGLYTSFFSSI  28602163

Query  121       IYTLLGTSRHISVGIFGVLCLLVGQVVDRELALAGYPSESSSVGGNSTGVLLASLGNETN  180
                 IYTLLGTSRHISVGIFGVLCLLVGQVVDRELALAGYPSESSSVGGNSTGVLLASLGNETN
Sbjct  28602164  IYTLLGTSRHISVGIFGVLCLLVGQVVDRELALAGYPSESSSVGGNSTGVLLASLGNETN  28602343

Query  181       VECDRSCYAITVGATVTFTAGVYQ  204
                 VECDRSCYAITVGATVTFTAGVYQ
Sbjct  28602344  VECDRSCYAITVGATVTFTAGVYQ  28602415
```

Range 3: 28603930 to 28604379

Score:550 bits(1121), Expect:1e-160,
Method:,
Identities:150/150(100%), Positives:150/150(100%), Gaps:0/150(0%)

```
Query  302       APIPFELFVVIIATLASHFGHFNSEYGSGVAGHIPTGFLPPQLPSWTLIPNVAVDAFSIA  361
                 APIPFELFVVIIATLASHFGHFNSEYGSGVAGHIPTGFLPPQLPSWTLIPNVAVDAFSIA
Sbjct  28603930  APIPFELFVVIIATLASHFGHFNSEYGSGVAGHIPTGFLPPQLPSWTLIPNVAVDAFSIA  28604109

Query  362       IVGFAITVSLSEMFAKKHGYSVDANQEMYAIGFCNILPSFFRCFTTSAALTKTLVKESTG  421
                 IVGFAITVSLSEMFAKKHGYSVDANQEMYAIGFCNILPSFFRCFTTSAALTKTLVKESTG
Sbjct  28604110  IVGFAITVSLSEMFAKKHGYSVDANQEMYAIGFCNILPSFFRCFTTSAALTKTLVKESTG  28604289

Query  422       CQTQMSGLVSALVLLLVLLVIAPLFYSLQK  451
                 CQTQMSGLVSALVLLLVLLVIAPLFYSLQK
Sbjct  28604290  CQTQMSGLVSALVLLLVLLVIAPLFYSLQK  28604379
```

Range 4: 28602617 to 28602910

Score:364 bits(742), Expect:2e-104,
Method:,
Identities:98/98(100%), Positives:98/98(100%), Gaps:0/98(0%)

```
Query  204       QMLMGLFQVGFVSVYLSDSLLSGFATGASLTILTSQFKYLLGLKIPRPQGWFTLFKTWYG  263
                 QMLMGLFQVGFVSVYLSDSLLSGFATGASLTILTSQFKYLLGLKIPRPQGWFTLFKTWYG
Sbjct  28602617  QMLMGLFQVGFVSVYLSDSLLSGFATGASLTILTSQFKYLLGLKIPRPQGWFTLFKTWYG  28602796

Query  264       IFTNLGKTNICDLVTSLVCLAILIPAKEFNDRFKTKLK  301
                 IFTNLGKTNICDLVTSLVCLAILIPAKEFNDRFKTKLK
Sbjct  28602797  IFTNLGKTNICDLVTSLVCLAILIPAKEFNDRFKTKLK  28602910
```

EJ Platyfish *slc26a2*

Query: sulfate transporter [Xiphophorus maculatus] Query ID: XP_005808685.1 Length: 718

>Xiphophorus maculatus strain JP 163 A chromosome 23, X_maculatus-5.0-male, whole genome shotgun sequence
Sequence ID: NC_036465.1 Length: 32170657
Range 1: 5631883 to 5632701

Score:1004 bits(2051), Expect:0.0,
Method:,
Identities:273/273(100%), Positives:273/273(100%), Gaps:0/273(0%)

```
Query  446      CVLAVIIVVNLRGALRKFRDVPRMWRVNKIDASIWLITMATSALVNTELGLLVGVLVSAF  505
                CVLAVIIVVNLRGALRKFRDVPRMWRVNKIDASIWLITMATSALVNTELGLLVGVLVSAF
Sbjct  5631883  CVLAVIIVVNLRGALRKFRDVPRMWRVNKIDASIWLITMATSALVNTELGLLVGVLVSAF  5632062

Query  506      FVLGRTQQAQVLELGRAASREHYENVSSYHGLQTHFGVAVFRYGAPIYYANQSLFKKSLY  565
                FVLGRTQQAQVLELGRAASREHYENVSSYHGLQTHFGVAVFRYGAPIYYANQSLFKKSLY
Sbjct  5632063  FVLGRTQQAQVLELGRAASREHYENVSSYHGLQTHFGVAVFRYGAPIYYANQSLFKKSLY  5632242

Query  566      KCANLKPVKEKAQHFKFEKNKNQEDTEEIPKGMSEDKQNKESEASSSVILMLDEKSSRNV  625
                KCANLKPVKEKAQHFKFEKNKNQEDTEEIPKGMSEDKQNKESEASSSVILMLDEKSSRNV
Sbjct  5632243  KCANLKPVKEKAQHFKFEKNKNQEDTEEIPKGMSEDKQNKESEASSSVILMLDEKSSRNV  5632422

Query  626      RSLVIDCSGILFLDTAGVNALKEVRKDYRELAVEVVLAQCNTSVLDSLERGGYFDSRKDG  685
                RSLVIDCSGILFLDTAGVNALKEVRKDYRELAVEVVLAQCNTSVLDSLERGGYFDSRKDG
Sbjct  5632423  RSLVIDCSGILFLDTAGVNALKEVRKDYRELAVEVVLAQCNTSVLDSLERGGYFDSRKDG  5632602

Query  686      EVGKNNMIFYTIADAVSYVQNLNVPNGDCDNKS  718
                EVGKNNMIFYTIADAVSYVQNLNVPNGDCDNKS
Sbjct  5632603  EVGKNNMIFYTIADAVSYVQNLNVPNGDCDNKS  5632701
```

Range 2: 5627754 to 5628350

Score:732 bits(1494), Expect:0.0,
Method:,
Identities:199/199(100%), Positives:199/199(100%), Gaps:0/199(0%)

```
Query  1        MLDGGDCCTAVEADPHQPLILERAEKEPEKKWHTVVSDRVKKHCSCSPEKAKSKILSFVP  60
                MLDGGDCCTAVEADPHQPLILERAEKEPEKKWHTVVSDRVKKHCSCSPEKAKSKILSFVP
Sbjct  5627754  MLDGGDCCTAVEADPHQPLILERAEKEPEKKWHTVVSDRVKKHCSCSPEKAKSKILSFVP  5627933

Query  61       ILQWLPHYKLKDWILGDIMSGLIVGILLVPQSIAYSLLASQDPIYGLYTSFFSSIIYAIL  120
                ILQWLPHYKLKDWILGDIMSGLIVGILLVPQSIAYSLLASQDPIYGLYTSFFSSIIYAIL
Sbjct  5627934  ILQWLPHYKLKDWILGDIMSGLIVGILLVPQSIAYSLLASQDPIYGLYTSFFSSIIYAIL  5628113

Query  121      GSSRHISVGIFGVLCLLVGQVVDRELALAGYLPESSFVSNNSDALLAGQGNSSNVKCDRS  180
                GSSRHISVGIFGVLCLLVGQVVDRELALAGYLPESSFVSNNSDALLAGQGNSSNVKCDRS
Sbjct  5628114  GSSRHISVGIFGVLCLLVGQVVDRELALAGYLPESSFVSNNSDALLAGQGNSSNVKCDRS  5628293

Query  181      CYAITVGATVTFTAGIYQV  199
                CYAITVGATVTFTAGIYQV
Sbjct  5628294  CYAITVGATVTFTAGIYQV  5628350
```

Range 3: 5630196 to 5630645

```
Score:551 bits(1124), Expect:7e-161,
Method:,
Identities:150/150(100%), Positives:150/150(100%), Gaps:0/150(0%)

Query  296      APIPFELFVVIIATLASHFGHFNSEYGSGVAGDIPTGFLPPQLPTWTLIPNVAVDAFSIA  355
                APIPFELFVVIIATLASHFGHFNSEYGSGVAGDIPTGFLPPQLPTWTLIPNVAVDAFSIA
Sbjct  5630196  APIPFELFVVIIATLASHFGHFNSEYGSGVAGDIPTGFLPPQLPTWTLIPNVAVDAFSIA  5630375

Query  356      IVGFAITVSLSEMFAKKHGYTVDANQEMYAIGFCNILPSFFRCFTTSAALTKTLVKESTG  415
                IVGFAITVSLSEMFAKKHGYTVDANQEMYAIGFCNILPSFFRCFTTSAALTKTLVKESTG
Sbjct  5630376  IVGFAITVSLSEMFAKKHGYTVDANQEMYAIGFCNILPSFFRCFTTSAALTKTLVKESTG  5630555

Query  416      CQTQMSGLVTAVILLLVLLVIAPLFYSLQK  445
                CQTQMSGLVTAVILLLVLLVIAPLFYSLQK
Sbjct  5630556  CQTQMSGLVTAVILLLVLLVIAPLFYSLQK  5630645



Range 4: 5628523 to 5628816

Score:359 bits(732), Expect:6e-103,
Method:,
Identities:98/98(100%), Positives:98/98(100%), Gaps:0/98(0%)

Query  198      QVLMGLLQVGFVSVYLSDSLLSGFATGASLTILTSQIKYLLGLKIPRPQGWFTLFKTWYS  257
                QVLMGLLQVGFVSVYLSDSLLSGFATGASLTILTSQIKYLLGLKIPRPQGWFTLFKTWYS
Sbjct  5628523  QVLMGLLQVGFVSVYLSDSLLSGFATGASLTILTSQIKYLLGLKIPRPQGWFTLFKTWYS  5628702

Query  258      LFTNIAKTNICDLITSLVCLAVLIPAKELNDRFKSKLK  295
                LFTNIAKTNICDLITSLVCLAVLIPAKELNDRFKSKLK
Sbjct  5628703  LFTNIAKTNICDLITSLVCLAVLIPAKELNDRFKSKLK  5628816
```

EK Northern snakehead *slc26a2*

```
Query: sulfate transporter [Channa argus] Query ID: XP_067375166.1 Length: 728


>Channa argus isolate OARG1902GOOAL chr9_contig_7, whole genome shotgun sequence
Sequence ID: SIWR01000094.1 Length: 3884873
Range 1: 735562 to 736416

Score:513 bits(1321), Expect:4e-158,
Method:Compositional matrix adjust.,
Identities:281/287(98%), Positives:283/287(98%), Gaps:2/287(0%)

Query  442     ISPLFYSLQKCVLAAIIVVNLRGALRKFTDIPRMWRVSRVDTSIWLITMASSAMVNTElg  501
               +SP F S  +CVLAAIIVVNLRGALRKFTDIPRMWRVSRVDTSIWLITMASSAMVNTELG
Sbjct  735562  LSPFFCS--RCVLAAIIVVNLRGALRKFTDIPRMWRVSRVDTSIWLITMASSAMVNTELG  735735

Query  502     llvgvlvsvFCILGRTQQVQVLELGRATTREHYEDLLSYHGLETHPGLAVFRYEAPIYYA  561
               LLVGVLVSVFCILGRTQQVQVLELGRATTREHYEDLLSYHGLETHPGLAVFRYEAPIYYA
Sbjct  735736  LLVGVLVSVFCILGRTQQVQVLELGRATTREHYEDLLSYHGLETHPGLAVFRYEAPIYYA  735915

Query  562     NQSLFKKSLYKCVGLDPVkeksmrikfkkkskqqkeVTGCSNMNSDKEDAHKEEEIEATA  621
               NQSLFKKSLYKCVGLDPVKEKSMRIKFKKKSKQQKEVTGCSNMNSDKEDAHKEEEIEATA
Sbjct  735916  NQSLFKKSLYKCVGLDPVKEKSMRIKFKKKSKQQKEVTGCSNMNSDKEDAHKEEEIEATA  736095

Query  622     NVTLMLNEKASRSLHSIVIDCSAVVFLDTAGVNALKEVRRDYEELGVRVVLAHCNISVLD  681
               NVTLMLNEKASRSLHSIVIDCSAVVFLDTAGVNALKEVRRDYEELGVRVVLAHCNISVLD
Sbjct  736096  NVTLMLNEKASRSLHSIVIDCSAVVFLDTAGVNALKEVRRDYEELGVRVVLAHCNISVLD  736275

Query  682     ILDRGGYYPDKKQGGTVDNKMIFFTIADAVHYTQSLSAPNGDCDTKQ  728
               ILDRGGYYPDKKQGGTVDNKMIFFTIADAVHYTQSLSAPNGDCDTKQ
Sbjct  736276  ILDRGGYYPDKKQGGTVDNKMIFFTIADAVHYTQSLSAPNGDCDTKQ  736416



Range 2: 732827 to 733441

Score:423 bits(1088), Expect:4e-127,
Method:Compositional matrix adjust.,
Identities:205/205(100%), Positives:205/205(100%), Gaps:0/205(0%)

Query  1       MNHGNDCYGATEEGAESGQQQPLILERVPKDAGKSWQTVASHRLKKHCLCTPAEAKSRIL  60
               MNHGNDCYGATEEGAESGQQQPLILERVPKDAGKSWQTVASHRLKKHCLCTPAEAKSRIL
Sbjct  732827  MNHGNDCYGATEEGAESGQQQPLILERVPKDAGKSWQTVASHRLKKHCLCTPAEAKSRIL  733006

Query  61      GFFPILKWLPHYRLRDWLLGDAMSGLIVGTLLVPQSIAYSLLASQDPIYGLYTSFFSSII  120
               GFFPILKWLPHYRLRDWLLGDAMSGLIVGTLLVPQSIAYSLLASQDPIYGLYTSFFSSII
Sbjct  733007  GFFPILKWLPHYRLRDWLLGDAMSGLIVGTLLVPQSIAYSLLASQDPIYGLYTSFFSSII  733186

Query  121     YALLGTSRHISVGIFGVLCLLVGQVVDRELALAGFLTESSSVGSDNSTVLFAGQGNGSLG  180
               YALLGTSRHISVGIFGVLCLLVGQVVDRELALAGFLTESSSVGSDNSTVLFAGQGNGSLG
Sbjct  733187  YALLGTSRHISVGIFGVLCLLVGQVVDRELALAGFLTESSSVGSDNSTVLFAGQGNGSLG  733366

Query  181     LECDRSCYAITVAATLTFTAGVYQV  205
               LECDRSCYAITVAATLTFTAGVYQV
Sbjct  733367  LECDRSCYAITVAATLTFTAGVYQV  733441
```

```
Range 3: 734161 to 734610

Score:278 bits(710), Expect:2e-77,
Method:Compositional matrix adjust.,
Identities:150/150(100%), Positives:150/150(100%), Gaps:0/150(0%)

Query  302     APIPFELFVVIIATLASHFGHFNTEYGSGVAGDIPTGFLPPRLPAWSLIPNVAVDAFSIA  361
               APIPFELFVVIIATLASHFGHFNTEYGSGVAGDIPTGFLPPRLPAWSLIPNVAVDAFSIA
Sbjct  734161  APIPFELFVVIIATLASHFGHFNTEYGSGVAGDIPTGFLPPRLPAWSLIPNVAVDAFSIA  734340

Query  362     IVGFAITVSLSELFAKKHGYTVDANQEMYAIGFCNILPSFFLCFSTSAALTKTLVKESTG  421
               IVGFAITVSLSELFAKKHGYTVDANQEMYAIGFCNILPSFFLCFSTSAALTKTLVKESTG
Sbjct  734341  IVGFAITVSLSELFAKKHGYTVDANQEMYAIGFCNILPSFFLCFSTSAALTKTLVKESTG  734520

Query  422     CHTQMSGlvsavvlllvllvISPLFYSLQK  451
               CHTQMSGLVSAVVLLLVLLVISPLFYSLQK
Sbjct  734521  CHTQMSGLVSAVVLLLVLLVISPLFYSLQK  734610
```

```
Range 4: 733586 to 733975

Score:200 bits(509), Expect:2e-51,
Method:Compositional matrix adjust.,
Identities:107/138(78%), Positives:111/138(80%), Gaps:8/138(5%)

Query  194     ATLTFTAGVYQVLMGLFQVGFISVYLSDSLLSGFATGASLTILTSQIKYLLGLKIPRPQG  253
               A L       QVLMGLFQVGFISVYLSDSLLSGFATGASLTILTSQIKYLLGLKIPRPQG
Sbjct  733586  ANLCLCISFSQVLMGLFQVGFISVYLSDSLLSGFATGASLTILTSQIKYLLGLKIPRPQG  733765

Query  254     WFTLFKTWSSLLINLADTNICDLVTSLVCLLILIPTKEFNERFKAKLKAPIPFELFVVII  313
               WFTLFKTWSSLLINLADTNICDLVTSLVCLLILIPTKEFNERFKAKLK          ++
Sbjct  733766  WFTLFKTWSSLLINLADTNICDLVTSLVCLLILIPTKEFNERFKAKLKVNNA-----KLV  733930

Query  314     ATLASHFGHFNTEYGSGV  331
                  + HF  +N   GSGV
Sbjct  733931  MERSQHFTKYN---GSGV  733975
```

EL Swamp eel *slc26a2*

```
Query: sulfate anion transporter 1 [Monopterus albus] Query ID: XP_020449704.1 Length: 726


>Monopterus albus unplaced genomic scaffold, M_albus_1.0 scaffold21.1, whole genome shotgun sequence
Sequence ID: NW_018127901.1 Length: 4120493
Range 1: 2843257 to 2844090

Score:1025 bits(2093), Expect:0.0,
Method:,
Identities:278/278(100%), Positives:278/278(100%), Gaps:0/278(0%)

Query  449      CVLAVIIVVNLRGALRKFTDIPHMWRVNRVDASIWLLTMATSAFVNTELGLLVGVLASAF  508
                CVLAVIIVVNLRGALRKFTDIPHMWRVNRVDASIWLLTMATSAFVNTELGLLVGVLASAF
Sbjct  2844090  CVLAVIIVVNLRGALRKFTDIPHMWRVNRVDASIWLLTMATSAFVNTELGLLVGVLASAF  2843911

Query  509      CVLGRTQQVHVLELGRTTTREHYEDLSSYHGLQTHPGVAVYRYDAPIYYANQSLFKKSLY  568
                CVLGRTQQVHVLELGRTTTREHYEDLSSYHGLQTHPGVAVYRYDAPIYYANQSLFKKSLY
Sbjct  2843910  CVLGRTQQVHVLELGRTTTREHYEDLSSYHGLQTHPGVAVYRYDAPIYYANQSLFKKSLY  2843731

Query  569      KCVGLDPVKEKTQRIKFNKKRRQQHGEDAEVPNVMSEDNVVHKEDDAESTTNVTLMLDGK  628
                KCVGLDPVKEKTQRIKFNKKRRQQHGEDAEVPNVMSEDNVVHKEDDAESTTNVTLMLDGK
Sbjct  2843730  KCVGLDPVKEKTQRIKFNKKRRQQHGEDAEVPNVMSEDNVVHKEDDAESTTNVTLMLDGK  2843551

Query  629      PSRSLHGIVIDCSSIVFLDTAGVNALKEVRKDYGELGVSVVLAQCNTSVLDTLERGGYHP  688
                PSRSLHGIVIDCSSIVFLDTAGVNALKEVRKDYGELGVSVVLAQCNTSVLDTLERGGYHP
Sbjct  2843550  PSRSLHGIVIDCSSIVFLDTAGVNALKEVRKDYGELGVSVVLAQCNTSVLDTLERGGYHP  2843371

Query  689      TKKESDEGESNSLFFTISDAVCYVQSFSAPSRDCDAKF  726
                TKKESDEGESNSLFFTISDAVCYVQSFSAPSRDCDAKF
Sbjct  2843370  TKKESDEGESNSLFFTISDAVCYVQSFSAPSRDCDAKF  2843257
```

```
Range 2: 2848941 to 2849549

Score:750 bits(1531), Expect:0.0,
Method:,
Identities:203/203(100%), Positives:203/203(100%), Gaps:0/203(0%)

Query  1        MATTAQQQKMRQRATSSSLSFWKESTKMWVGKNWRTTVLHQLKKHCLCTPKIAKSKILGF  60
                MATTAQQQKMRQRATSSSLSFWKESTKMWVGKNWRTTVLHQLKKHCLCTPKIAKSKILGF
Sbjct  2849549  MATTAQQQKMRQRATSSSLSFWKESTKMWVGKNWRTTVLHQLKKHCLCTPKIAKSKILGF  2849370

Query  61       FPILKWLPHYQLKDWLLGDIVSGLIVGILLVPQSIAYSLLASQDPIYGLYTSFFSSIIYA  120
                FPILKWLPHYQLKDWLLGDIVSGLIVGILLVPQSIAYSLLASQDPIYGLYTSFFSSIIYA
Sbjct  2849369  FPILKWLPHYQLKDWLLGDIVSGLIVGILLVPQSIAYSLLASQDPIYGLYTSFFSSIIYA  2849190
```

```
Query  121      LLGTSRHISVGIFGVLCLLVGQVVDRELALAGYLIEGSSIRHNDSTVPLAGPGNSSVGME  180
                LLGTSRHISVGIFGVLCLLVGQVVDRELALAGYLIEGSSIRHNDSTVPLAGPGNSSVGME
Sbjct  2849189  LLGTSRHISVGIFGVLCLLVGQVVDRELALAGYLIEGSSIRHNDSTVPLAGPGNSSVGME  2849010

Query  181      CDRGCYAITVGATLTFTAGVYQV  203
                CDRGCYAITVGATLTFTAGVYQV
Sbjct  2849009  CDRGCYAITVGATLTFTAGVYQV  2848941
```

Range 3: 2846641 to 2847087

Score:548 bits(1118), Expect:2e-159,
Method:,
Identities:149/149(100%), Positives:149/149(100%), Gaps:0/149(0%)

```
Query  300      APIPFELFVVIIATLASHFGHFNTEYGSSVAGDIPTGFLPPQLPNWSLIPNVAVDAFSIA  359
                APIPFELFVVIIATLASHFGHFNTEYGSSVAGDIPTGFLPPQLPNWSLIPNVAVDAFSIA
Sbjct  2847087  APIPFELFVVIIATLASHFGHFNTEYGSSVAGDIPTGFLPPQLPNWSLIPNVAVDAFSIA  2846908

Query  360      IVAFAITVSLSEMFAKKHGYTVDANQEMYAIGFCNILPSFFHCFTTSAALTKTLVKESTG  419
                IVAFAITVSLSEMFAKKHGYTVDANQEMYAIGFCNILPSFFHCFTTSAALTKTLVKESTG
Sbjct  2846907  IVAFAITVSLSEMFAKKHGYTVDANQEMYAIGFCNILPSFFHCFTTSAALTKTLVKESTG  2846728

Query  420      CQTQMSGLVSAFILLLVLLVIAPLPFRSK  448
                CQTQMSGLVSAFILLLVLLVIAPLPFRSK
Sbjct  2846727  CQTQMSGLVSAFILLLVLLVIAPLPFRSK  2846641
```

Range 4: 2848359 to 2848649

Score:357 bits(728), Expect:7e-102,
Method:,
Identities:97/97(100%), Positives:97/97(100%), Gaps:0/97(0%)

```
Query  203      VLMGLFHVGFVSIYLSDSLLSGFATGASLTILTSQFKFLLGLKIPRPQGWFTLFKTWYSL  262
                VLMGLFHVGFVSIYLSDSLLSGFATGASLTILTSQFKFLLGLKIPRPQGWFTLFKTWYSL
Sbjct  2848649  VLMGLFHVGFVSIYLSDSLLSGFATGASLTILTSQFKFLLGLKIPRPQGWFTLFKTWYSL  2848470

Query  263      FTNLGNANVCDLVTSLVCLLILIPTKELNDRFKAKLK  299
                FTNLGNANVCDLVTSLVCLLILIPTKELNDRFKAKLK
Sbjct  2848469  FTNLGNANVCDLVTSLVCLLILIPTKELNDRFKAKLK  2848359
```

EM Greater amberjack *slc26a2*

Query: sulfate anion transporter 1 [Seriola dumerili] Query ID: XP_022616056.1 Length: 728

>Seriola dumerili isolate Sdu_G_001 unplaced genomic scaffold, Sdu_1.0 BDQW01000331.1, whole genome shotgun sequence
Sequence ID: NW_019174586.1 Length: 13580752
Range 1: 9560977 to 9561816

Score:1016 bits(2075), Expect:0.0,
Method:,
Identities:278/280(99%), Positives:278/280(99%), Gaps:0/280(0%)

```
Query  449      LQKCVLAVIIVVNLRGALRKFTDIPRMWRANRGDTSIWLITMLTSSLVNTELGLLVGVLV  508
                L  CVLAVIIVVNLRGALRKFTDIPRMWRANRGDTSIWLITMLTSSLVNTELGLLVGVLV
Sbjct  9561816  LPRCVLAVIIVVNLRGALRKFTDIPRMWRANRGDTSIWLITMLTSSLVNTELGLLVGVLV  9561637

Query  509      SAFCVLGRTQQVQVLELGRAATREHYEDLSSYRGLQTHPGVAVFRYEAPIYYANQSLFKR  568
                SAFCVLGRTQQVQVLELGRAATREHYEDLSSYRGLQTHPGVAVFRYEAPIYYANQSLFKR
Sbjct  9561636  SAFCVLGRTQQVQVLELGRAATREHYEDLSSYRGLQTHPGVAVFRYEAPIYYANQSLFKR  9561457

Query  569      SLYKRVGLDPVREKTRHLKFKKKKSKQQKEVAKDPNMKSEGKNACKDEEAEATATVNLML  628
                SLYKRVGLDPVREKTRHLKFKKKKSKQQKEVAKDPNMKSEGKNACKDEEAEATATVNLML
Sbjct  9561456  SLYKRVGLDPVREKTRHLKFKKKKSKQQKEVAKDPNMKSEGKNACKDEEAEATATVNLML  9561277

Query  629      DEKSRSLHSIVIDCSAILFLDTAGVNALKEVRKDYGELGVKVVLAQCNTSLLDTLERGGY  688
                DEKSRSLHSIVIDCSAILFLDTAGVNALKEVRKDYGELGVKVVLAQCNTSLLDTLERGGY
Sbjct  9561276  DEKSRSLHSIVIDCSAILFLDTAGVNALKEVRKDYGELGVKVVLAQCNTSLLDTLERGGY  9561097

Query  689      YPDTKGGDGGENSIVFFTIADAVRYVQSLSAPNGDCDTKC  728
                YPDTKGGDGGENSIVFFTIADAVRYVQSLSAPNGDCDTKC
Sbjct  9561096  YPDTKGGDGGENSIVFFTIADAVRYVQSLSAPNGDCDTKC  9560977
```

Range 2: 9564914 to 9565528

Score:745 bits(1520), Expect:0.0,
Method:,
Identities:205/205(100%), Positives:205/205(100%), Gaps:0/205(0%)

```
Query  1        MTHGSDCCTAAEDGAESDQQQPLILERVEKEKGKSCRTAALRRLKKHCSCTPKKAKSKIL  60
                MTHGSDCCTAAEDGAESDQQQPLILERVEKEKGKSCRTAALRRLKKHCSCTPKKAKSKIL
Sbjct  9565528  MTHGSDCCTAAEDGAESDQQQPLILERVEKEKGKSCRTAALRRLKKHCSCTPKKAKSKIL  9565349
```

```
Query  61       GFFPILKWLPRYKLRDWILGDAMSGLIVGILLVPQSIAYSLLASQDPIYGLYTSFFSSII  120
                GFFPILKWLPRYKLRDWILGDAMSGLIVGILLVPQSIAYSLLASQDPIYGLYTSFFSSII
Sbjct  9565348  GFFPILKWLPRYKLRDWILGDAMSGLIVGILLVPQSIAYSLLASQDPIYGLYTSFFSSII  9565169

Query  121      YALLGTSRHISVGIFGVLCLLVGQVVDRELALAGYLTESSITSSNNSAVLLAGQGNGSVG  180
                YALLGTSRHISVGIFGVLCLLVGQVVDRELALAGYLTESSITSSNNSAVLLAGQGNGSVG
Sbjct  9565168  YALLGTSRHISVGIFGVLCLLVGQVVDRELALAGYLTESSITSSNNSAVLLAGQGNGSVG  9564989

Query  181      VECDRSCYAITVGATVTFTAGVYQV  205
                VECDRSCYAITVGATVTFTAGVYQV
Sbjct  9564988  VECDRSCYAITVGATVTFTAGVYQV  9564914
```

Range 3: 9563117 to 9563572

Score:552 bits(1125), Expect:5e-161,
Method:,
Identities:151/152(99%), Positives:151/152(99%), Gaps:0/152(0%)

```
Query  300      LKAPIPFELFVVIIATLASHFGHFNTEYGSSVAGDIPTGFLPPQLPLWSLIPNVAVDAFS  359
                L APIPFELFVVIIATLASHFGHFNTEYGSSVAGDIPTGFLPPQLPLWSLIPNVAVDAFS
Sbjct  9563572  LQAPIPFELFVVIIATLASHFGHFNTEYGSSVAGDIPTGFLPPQLPLWSLIPNVAVDAFS  9563393

Query  360      IAIVGFAITVSLSEMFAKKHGYTVDANQEMYAIGFCNILPSFFRCFSTSAALTKTLVKES  419
                IAIVGFAITVSLSEMFAKKHGYTVDANQEMYAIGFCNILPSFFRCFSTSAALTKTLVKES
Sbjct  9563392  IAIVGFAITVSLSEMFAKKHGYTVDANQEMYAIGFCNILPSFFRCFSTSAALTKTLVKES  9563213

Query  420      TGCQTQMSGLVSALVLLLVLLVIAPLFYSLQK  451
                TGCQTQMSGLVSALVLLLVLLVIAPLFYSLQK
Sbjct  9563212  TGCQTQMSGLVSALVLLLVLLVIAPLFYSLQK  9563117
```

Range 4: 9564317 to 9564610

Score:362 bits(737), Expect:1e-103,
Method:,
Identities:98/98(100%), Positives:98/98(100%), Gaps:0/98(0%)

```
Query  204      QVLMGIFQVGFVSVYLSDSLLSGFATGASLTILTSQFKYLLGLKIPRPQGWFTLFKTWYS  263
                QVLMGIFQVGFVSVYLSDSLLSGFATGASLTILTSQFKYLLGLKIPRPQGWFTLFKTWYS
Sbjct  9564610  QVLMGIFQVGFVSVYLSDSLLSGFATGASLTILTSQFKYLLGLKIPRPQGWFTLFKTWYS  9564431

Query  264      LFTNLGNTNICDLVTSLVCLLVLIPTKELNDRFKAKLK  301
                LFTNLGNTNICDLVTSLVCLLVLIPTKELNDRFKAKLK
Sbjct  9564430  LFTNLGNTNICDLVTSLVCLLVLIPTKELNDRFKAKLK  9564317
```

EN Japanese lates *slc26a2*

Query: sulfate transporter-like protein [Lates japonicus] Query ID: GLD70538.1 Length: 718

>Lates japonicus DNA, scaffold_372, whole genome shotgun sequence
Sequence ID: BRZM01000373.1 Length: 10773027
Range 1: 10180257 to 10181060

Score:980 bits(2001), Expect:0.0,
Method:,
Identities:268/268(100%), Positives:268/268(100%), Gaps:0/268(0%)

```
Query  451       CVLAVIIVVNLRGALRKFTDIPRMWRVNHVDTSIWLVTMLTSSLVNTELGLLVGVLVSAF  510
                 CVLAVIIVVNLRGALRKFTDIPRMWRVNHVDTSIWLVTMLTSSLVNTELGLLVGVLVSAF
Sbjct  10180257  CVLAVIIVVNLRGALRKFTDIPRMWRVNHVDTSIWLVTMLTSSLVNTELGLLVGVLVSAF  10180436

Query  511       CVLGRTQQVQVLELGRATTREHYEDLSSYRGLQTHPGVAVFRYEAPLYYANQSMFKKSLY  570
                 CVLGRTQQVQVLELGRATTREHYEDLSSYRGLQTHPGVAVFRYEAPLYYANQSMFKKSLY
Sbjct  10180437  CVLGRTQQVQVLELGRATTREHYEDLSSYRGLQTHPGVAVFRYEAPLYYANQSMFKKSLY  10180616

Query  571       KCVGLNPVKEKTRRLKFKKKSKQQEGVADATNIKSEKEDEATATVTLMPDEKSSLRSIVI  630
                 KCVGLNPVKEKTRRLKFKKKSKQQEGVADATNIKSEKEDEATATVTLMPDEKSSLRSIVI
Sbjct  10180617  KCVGLNPVKEKTRRLKFKKKSKQQEGVADATNIKSEKEDEATATVTLMPDEKSSLRSIVI  10180796

Query  631       DCSAVLFLDTAGVNALKDVRKDYGELGVKVVLAQCNTSVLDTLERGGYYPDKKGGDGES  690
                 DCSAVLFLDTAGVNALKDVRKDYGELGVKVVLAQCNTSVLDTLERGGYYPDKKGGDGES
Sbjct  10180797  DCSAVLFLDTAGVNALKDVRKDYGELGVKVVLAQCNTSVLDTLERGGYYPDKKGGDGES  10180976

Query  691       RIVFLTIADAVRYIQSLSAPNGDCDTKY  718
                 RIVFLTIADAVRYIQSLSAPNGDCDTKY
Sbjct  10180977  RIVFLTIADAVRYIQSLSAPNGDCDTKY  10181060
```

Range 2: 10176850 to 10177461

Score:745 bits(1521), Expect:0.0,
Method:,
Identities:204/204(100%), Positives:204/204(100%), Gaps:0/204(0%)

```
Query  1         MTHGDDCCTTAEDGADSDQQQPLILERVEKEAGKSCKTVVLQRLKKHCSCTPKKAKSKIL  60
                 MTHGDDCCTTAEDGADSDQQQPLILERVEKEAGKSCKTVVLQRLKKHCSCTPKKAKSKIL
Sbjct  10176850  MTHGDDCCTTAEDGADSDQQQPLILERVEKEAGKSCKTVVLQRLKKHCSCTPKKAKSKIL  10177029

Query  61        GFFPILKWLPRYQLRDWLLGDVMSGLIVGILLVPQSIAYSLLASQDPIYGLYTSFFSSII  120
                 GFFPILKWLPRYQLRDWLLGDVMSGLIVGILLVPQSIAYSLLASQDPIYGLYTSFFSSII
Sbjct  10177030  GFFPILKWLPRYQLRDWLLGDVMSGLIVGILLVPQSIAYSLLASQDPIYGLYTSFFSSII  10177209

Query  121       YTILGSSRHISVGIFGVLCLLVGQVVDRELALAGYLTESSISRNDSAVLLAGQGNGSIGV  180
                 YTILGSSRHISVGIFGVLCLLVGQVVDRELALAGYLTESSISRNDSAVLLAGQGNGSIGV
Sbjct  10177210  YTILGSSRHISVGIFGVLCLLVGQVVDRELALAGYLTESSISRNDSAVLLAGQGNGSIGV  10177389

Query  181       QCDRSCYAITVGATVTFTAGVYQV  204
                 QCDRSCYAITVGATVTFTAGVYQV
Sbjct  10177390  QCDRSCYAITVGATVTFTAGVYQV  10177461
```

Range 3: 10177550 to 10177843

Score:362 bits(737), Expect:0.0,
Method:,
Identities:98/98(100%), Positives:98/98(100%), Gaps:0/98(0%)

```
Query  203       QVLMGIFQVGFVSVYLSDSLLSGFATGASLTILTSQFKYLLGLKIPRPQGWFTLFKTWYS  262
                 QVLMGIFQVGFVSVYLSDSLLSGFATGASLTILTSQFKYLLGLKIPRPQGWFTLFKTWYS
Sbjct  10177550  QVLMGIFQVGFVSVYLSDSLLSGFATGASLTILTSQFKYLLGLKIPRPQGWFTLFKTWYS  10177729

Query  263       ILTNLGNTNICDLVTSLVCLLILIPTKELNDRFKAKLK  300
                 ILTNLGNTNICDLVTSLVCLLILIPTKELNDRFKAKLK
Sbjct  10177730  ILTNLGNTNICDLVTSLVCLLILIPTKELNDRFKAKLK  10177843
```

Range 4: 10178442 to 10178891

Score:550 bits(1121), Expect:1e-160,
Method:,
Identities:150/150(100%), Positives:150/150(100%), Gaps:0/150(0%)

```
Query  301       APIPFELFVVIIATLASHFGRFNTDYGSGVAGDIPTGFLPPQLPSWSLIPNVAIDAFSIA  360
                 APIPFELFVVIIATLASHFGRFNTDYGSGVAGDIPTGFLPPQLPSWSLIPNVAIDAFSIA
Sbjct  10178442  APIPFELFVVIIATLASHFGRFNTDYGSGVAGDIPTGFLPPQLPSWSLIPNVAIDAFSIA  10178621

Query  361       IVGFAITVSLSEMFAKKHGYTVDANQEMYAIGFCNILPSFFRCFTTSAALTKTLVKESTG  420
                 IVGFAITVSLSEMFAKKHGYTVDANQEMYAIGFCNILPSFFRCFTTSAALTKTLVKESTG
Sbjct  10178622  IVGFAITVSLSEMFAKKHGYTVDANQEMYAIGFCNILPSFFRCFTTSAALTKTLVKESTG  10178801

Query  421       CQTQMSGLVSALVLLLVLLVIAPLFYSLQK  450
                 CQTQMSGLVSALVLLLVLLVIAPLFYSLQK
Sbjct  10178802  CQTQMSGLVSALVLLLVLLVIAPLFYSLQK  10178891
```

EO Turbot *slc26a2*

Query: sulfate transporter [Scophthalmus maximus] Query ID: XP_035505604.2 Length: 728

>Scophthalmus maximus strain ysfricsl-2021 chromosome 9, ASM2237912v1, whole genome shotgun sequence
Sequence ID: NC_061523.1 Length: 25907553
Range 1: 25394617 to 25395426

Score:988 bits(2017), Expect:0.0,
Method:,
Identities:270/270(100%), Positives:270/270(100%), Gaps:0/270(0%)

```
Query  459       CVLAVIIVVNLRGALRKFADVPRMWRVNRVDASIWLVTMATSSLVNTELGLLVGVLASAF  518
                 CVLAVIIVVNLRGALRKFADVPRMWRVNRVDASIWLVTMATSSLVNTELGLLVGVLASAF
Sbjct  25395426  CVLAVIIVVNLRGALRKFADVPRMWRVNRVDASIWLVTMATSSLVNTELGLLVGVLASAF  25395247

Query  519       CVLGRTQRVRVCELGRAAAREHYEDLTSYRGLRTHPGVAVFRYEAPIYYANQSLFKKSLY  578
                 CVLGRTQRVRVCELGRAAAREHYEDLTSYRGLRTHPGVAVFRYEAPIYYANQSLFKKSLY
Sbjct  25395246  CVLGRTQRVRVCELGRAAAREHYEDLTSYRGLRTHPGVAVFRYEAPIYYANQSLFKKSLY  25395067

Query  579       DRVGLDPVKEKTRRLKFKKKTSEQEEEVADVTYRKPEKDEATAAATVTLDEKSRRSVRSV  638
                 DRVGLDPVKEKTRRLKFKKKTSEQEEEVADVTYRKPEKDEATAAATVTLDEKSRRSVRSV
Sbjct  25395066  DRVGLDPVKEKTRRLKFKKKTSEQEEEVADVTYRKPEKDEATAAATVTLDEKSRRSVRSV  25394887

Query  639       VIDCSAILFLDTAGVNALKEVRKDYEDLGVTVVLAQCHTSVREALERGGYCPDAGEDHGG  698
                 VIDCSAILFLDTAGVNALKEVRKDYEDLGVTVVLAQCHTSVREALERGGYCPDAGEDHGG
Sbjct  25394886  VIDCSAILFLDTAGVNALKEVRKDYEDLGVTVVLAQCHTSVREALERGGYCPDAGEDHGG  25394707

Query  699       ERRLVFFTIADAVRYVQNLSAANGDCDTKC  728
                 ERRLVFFTIADAVRYVQNLSAANGDCDTKC
Sbjct  25394706  ERRLVFFTIADAVRYVQNLSAANGDCDTKC  25394617
```

Range 2: 25398027 to 25398662

```
Score:774 bits(1579), Expect:0.0,
Method:,
Identities:212/212(100%), Positives:212/212(100%), Gaps:0/212(0%)

Query  1         MARDNAWCAPAEDDGAESDQQQQQQPLILERVEKEAGKDCRSAALHRLRKHCSCTSEKAK  60
                 MARDNAWCAPAEDDGAESDQQQQQQPLILERVEKEAGKDCRSAALHRLRKHCSCTSEKAK
Sbjct  25398662  MARDNAWCAPAEDDGAESDQQQQQQPLILERVEKEAGKDCRSAALHRLRKHCSCTSEKAK  25398483

Query  61        SKVLDFFPIVRWLPRYQLRDWILGDAMSGLIVGILLVPQSIAYSLLASQDPIYGLYTSFF  120
                 SKVLDFFPIVRWLPRYQLRDWILGDAMSGLIVGILLVPQSIAYSLLASQDPIYGLYTSFF
Sbjct  25398482  SKVLDFFPIVRWLPRYQLRDWILGDAMSGLIVGILLVPQSIAYSLLASQDPIYGLYTSFF  25398303

Query  121       SAIIYAVLGTSRHISVGIFGVLCLLVGQVVDRELALAGYLTESGIGSGISSNDSAVLLAG  180
                 SAIIYAVLGTSRHISVGIFGVLCLLVGQVVDRELALAGYLTESGIGSGISSNDSAVLLAG
Sbjct  25398302  SAIIYAVLGTSRHISVGIFGVLCLLVGQVVDRELALAGYLTESGIGSGISSNDSAVLLAG  25398123

Query  181       QGNGSVVLVCDRSCYAITVGATVTFTAGVYQV  212
                 QGNGSVVLVCDRSCYAITVGATVTFTAGVYQV
Sbjct  25398122  QGNGSVVLVCDRSCYAITVGATVTFTAGVYQV  25398027
```

```
Range 3: 25397645 to 25397938

Score:358 bits(729), Expect:0.0,
Method:,
Identities:98/98(100%), Positives:98/98(100%), Gaps:0/98(0%)

Query  211       QVLMGLFQVGFVSVYLSDSLLSGFATGASLTILTSQVKYLLGLKLPRPQGWFTLCKTWYS  270
                 QVLMGLFQVGFVSVYLSDSLLSGFATGASLTILTSQVKYLLGLKLPRPQGWFTLCKTWYS
Sbjct  25397938  QVLMGLFQVGFVSVYLSDSLLSGFATGASLTILTSQVKYLLGLKLPRPQGWFTLCKTWYS  25397759

Query  271       LLVNLGSTNVCDLVTSLVCLLVLIPTKELNDRFKAKLK  308
                 LLVNLGSTNVCDLVTSLVCLLVLIPTKELNDRFKAKLK
Sbjct  25397758  LLVNLGSTNVCDLVTSLVCLLVLIPTKELNDRFKAKLK  25397645
```

```
Range 4: 25396482 to 25396931

Score:545 bits(1112), Expect:3e-159,
Method:,
Identities:150/150(100%), Positives:150/150(100%), Gaps:0/150(0%)

Query  309       APIPFELFVVIIATLASHFGHFDTAYGSSVAGVIPTGFLAPQLPLWSLIPNVAVDAFSIA  368
                 APIPFELFVVIIATLASHFGHFDTAYGSSVAGVIPTGFLAPQLPLWSLIPNVAVDAFSIA
Sbjct  25396931  APIPFELFVVIIATLASHFGHFDTAYGSSVAGVIPTGFLAPQLPLWSLIPNVAVDAFSIA  25396752

Query  369       IVGFAITVSLSEMFAKKHGYAVDANQEMYAIGFCNILPSFFRCFTTSAALTKTLVKESTG  428
                 IVGFAITVSLSEMFAKKHGYAVDANQEMYAIGFCNILPSFFRCFTTSAALTKTLVKESTG
Sbjct  25396751  IVGFAITVSLSEMFAKKHGYAVDANQEMYAIGFCNILPSFFRCFTTSAALTKTLVKESTG  25396572

Query  429       CQTQVSGLVSALVLLLVLLVIAPLFYSLQK  458
                 CQTQVSGLVSALVLLLVLLVIAPLFYSLQK
Sbjct  25396571  CQTQVSGLVSALVLLLVLLVIAPLFYSLQK  25396482
```

EP Humphead wrasse *slc26a2*

```
Query: sulfate transporter [Cheilinus undulatus] Query ID: XP_041653610.1 Length: 723


>Cheilinus undulatus linkage group 10, ASM1832078v1, whole genome shotgun sequence
Sequence ID: NC_054874.1 Length: 51507803
Range 1: 45429169 to 45430002

Score:1007 bits(2056), Expect:0.0,
Method:,
Identities:276/278(99%), Positives:276/278(99%), Gaps:0/278(0%)

Query  446       LQKCVLAVIIVVNLRGALRKFTDVPRMWRVNRVDTAIWLITMATSALVNTELGLLVGVLV  505
                 L  CVLAVIIVVNLRGALRKFTDVPRMWRVNRVDTAIWLITMATSALVNTELGLLVGVLV
Sbjct  45429169  LLRCVLAVIIVVNLRGALRKFTDVPRMWRVNRVDTAIWLITMATSALVNTELGLLVGVLV  45429348

Query  506       SAFCILGRTQQAQALQLGRATTREHYEDMSSYRGLRTHPGVVVFRYNAPIYYANQNLFKK  565
                 SAFCILGRTQQAQALQLGRATTREHYEDMSSYRGLRTHPGVVVFRYNAPIYYANQNLFKK
Sbjct  45429349  SAFCILGRTQQAQALQLGRATTREHYEDMSSYRGLRTHPGVVVFRYNAPIYYANQNLFKK  45429528

Query  566       SLYRSVGLDPVKEKSRRIKFSKKNKKKEEAKGLLNLKSEKTAAGKEDKAEDGATVTLMLN  625
                 SLYRSVGLDPVKEKSRRIKFSKKNKKKEEAKGLLNLKSEKTAAGKEDKAEDGATVTLMLN
Sbjct  45429529  SLYRSVGLDPVKEKSRRIKFSKKNKKKEEAKGLLNLKSEKTAAGKEDKAEDGATVTLMLN  45429708

Query  626       SKSLSFHSLVMDCSTVLFLDTAGVNALKEVRKDYGELGIKVVLAQCNASVLDDLERGGYY  685
                 SKSLSFHSLVMDCSTVLFLDTAGVNALKEVRKDYGELGIKVVLAQCNASVLDDLERGGYY
Sbjct  45429709  SKSLSFHSLVMDCSTVLFLDTAGVNALKEVRKDYGELGIKVVLAQCNASVLDDLERGGYY  45429888

Query  686       DEKGPDGGENKRIFFTIADAVHHAQSLLAPNGDYDNKC  723
                 DEKGPDGGENKRIFFTIADAVHHAQSLLAPNGDYDNKC
Sbjct  45429889  DEKGPDGGENKRIFFTIADAVHHAQSLLAPNGDYDNKC  45430002
```

Range 2: 45423786 to 45424391

Score:743 bits(1517), Expect:0.0,
Method:,
Identities:202/202(100%), Positives:202/202(100%), Gaps:0/202(0%)

```
Query  1         MTLSADFCTTVEDEADSSPTEPLVLERVEKEKDKNCKAILSHQLKKCCSCTPKKVKSKIL  60
                 MTLSADFCTTVEDEADSSPTEPLVLERVEKEKDKNCKAILSHQLKKCCSCTPKKVKSKIL
Sbjct  45423786  MTLSADFCTTVEDEADSSPTEPLVLERVEKEKDKNCKAILSHQLKKCCSCTPKKVKSKIL  45423965

Query  61        GFVPILKWLPNYQLREWLLGDLMSGLIVGILLVPQSIAYSLLASQDPIYGLYTSFFASII  120
                 GFVPILKWLPNYQLREWLLGDLMSGLIVGILLVPQSIAYSLLASQDPIYGLYTSFFASII
Sbjct  45423966  GFVPILKWLPNYQLREWLLGDLMSGLIVGILLVPQSIAYSLLASQDPIYGLYTSFFASII  45424145

Query  121       YTLLGTSKHISVGIFGVLCLLVGQVVDRELALAGYLPKSSSSVRNNSEILPGNDNVVMEC  180
                 YTLLGTSKHISVGIFGVLCLLVGQVVDRELALAGYLPKSSSSVRNNSEILPGNDNVVMEC
Sbjct  45424146  YTLLGTSKHISVGIFGVLCLLVGQVVDRELALAGYLPKSSSSVRNNSEILPGNDNVVMEC  45424325

Query  181       DRSCYAITVGTTVTFTAGVYQV  202
                 DRSCYAITVGTTVTFTAGVYQV
Sbjct  45424326  DRSCYAITVGTTVTFTAGVYQV  45424391
```

Range 3: 45425670 to 45426119

Score:551 bits(1124), Expect:2e-160,
Method:,
Identities:150/150(100%), Positives:150/150(100%), Gaps:0/150(0%)

```
Query  299       APIPFELFVVIIATLASHFGHFNSEYLSDIAGDIPTGFLPPKAPMWSLIPNVAVDAFSIA  358
                 APIPFELFVVIIATLASHFGHFNSEYLSDIAGDIPTGFLPPKAPMWSLIPNVAVDAFSIA
Sbjct  45425670  APIPFELFVVIIATLASHFGHFNSEYLSDIAGDIPTGFLPPKAPMWSLIPNVAVDAFSIA  45425849

Query  359       IVGFAITVSLSEMFAKKHGYTVDANQEMYAIGFCNILPSFFRCFTTSAALTKTLVKESTG  418
                 IVGFAITVSLSEMFAKKHGYTVDANQEMYAIGFCNILPSFFRCFTTSAALTKTLVKESTG
Sbjct  45425850  IVGFAITVSLSEMFAKKHGYTVDANQEMYAIGFCNILPSFFRCFTTSAALTKTLVKESTG  45426029

Query  419       CQTQVSGLVTALVLLLVLLVIAPLFYSLQK  448
                 CQTQVSGLVTALVLLLVLLVIAPLFYSLQK
Sbjct  45426030  CQTQVSGLVTALVLLLVLLVIAPLFYSLQK  45426119
```

Range 4: 45425083 to 45425376

Score:362 bits(738), Expect:2e-103,
Method:,
Identities:98/98(100%), Positives:98/98(100%), Gaps:0/98(0%)

```
Query  201       QVLMGIFQVGFVSVYLSDSLLSGFATGASLTILTSQFKYLLGLEIPRPQGWFTLFKTWYS  260
                 QVLMGIFQVGFVSVYLSDSLLSGFATGASLTILTSQFKYLLGLEIPRPQGWFTLFKTWYS
Sbjct  45425083  QVLMGIFQVGFVSVYLSDSLLSGFATGASLTILTSQFKYLLGLEIPRPQGWFTLFKTWYS  45425262

Query  261       LFTHLGHTNICDLVTSLVCLLVLIPTKELNDRLKSKLK  298
                 LFTHLGHTNICDLVTSLVCLLVLIPTKELNDRLKSKLK
Sbjct  45425263  LFTHLGHTNICDLVTSLVCLLVLIPTKELNDRLKSKLK  45425376
```

EQ Chinese perch *slc26a2*

Query: sulfate transporter [Siniperca chuatsi] Query ID: XP_044061644.1 Length: 730

>Siniperca chuatsi isolate FFG_IHB_CAS linkage group LG8, ASM2008510v1, whole genome shotgun sequence
Sequence ID: NC_058049.1 Length: 31750086
Range 1: 3920631 to 3921467

Score:1018 bits(2079), Expect:0.0,
Method:,
Identities:277/279(99%), Positives:277/279(99%), Gaps:0/279(0%)

```
Query  452      LQKCVLAVIIVVNLRGALRKFTDIPRMWCVNRVDTSIWVITMATSALVNTELGLLVGVLV  511
                L  CVLAVIIVVNLRGALRKFTDIPRMWCVNRVDTSIWVITMATSALVNTELGLLVGVLV
Sbjct  3921467  LPRCVLAVIIVVNLRGALRKFTDIPRMWCVNRVDTSIWVITMATSALVNTELGLLVGVLV  3921288

Query  512      SAFCVLGRTQQAQVLELGRATSREHYEDLSSYRGLQTHSGVTVFRYEAPIYYANQSLFKK  571
                SAFCVLGRTQQAQVLELGRATSREHYEDLSSYRGLQTHSGVTVFRYEAPIYYANQSLFKK
Sbjct  3921287  SAFCVLGRTQQAQVLELGRATSREHYEDLSSYRGLQTHSGVTVFRYEAPIYYANQSLFKK  3921108

Query  572      SLYRCVGLDPVKEKTRRMKFKKKNKQEGEVAGVPNMKSEQEEAEGKEDEVEAGVTVTLML  631
                SLYRCVGLDPVKEKTRRMKFKKKNKQEGEVAGVPNMKSEQEEAEGKEDEVEAGVTVTLML
Sbjct  3921107  SLYRCVGLDPVKEKTRRMKFKKKNKQEGEVAGVPNMKSEQEEAEGKEDEVEAGVTVTLML  3920928

Query  632      NKKSSLRSLVIDCSAILFLDTAGVNALKEVRKDYGELGIKIVLAQCNASVLDDLERGGYY  691
                NKKSSLRSLVIDCSAILFLDTAGVNALKEVRKDYGELGIKIVLAQCNASVLDDLERGGYY
Sbjct  3920927  NKKSSLRSLVIDCSAILFLDTAGVNALKEVRKDYGELGIKIVLAQCNASVLDDLERGGYY  3920748

Query  692      PDKKGCDGGENKIMFFTIADAVRYVQSLSTPNGYYDNKS  730
```

```
               PDKKGCDGGENKIMFFTIADAVRYVQSLSTPNGYYDNKS
Sbjct  3920747  PDKKGCDGGENKIMFFTIADAVRYVQSLSTPNGYYDNKS  3920631
```

Range 2: 3925489 to 3926112

Score:761 bits(1554), Expect:0.0,
Method:,
Identities:208/208(100%), Positives:208/208(100%), Gaps:0/208(0%)

```
Query  1        MTHGDDCCPTAEDGAESDQQQQQQPLILERVEKETVKDWQSVVSHRLKKHCLCTPKKAKS  60
                MTHGDDCCPTAEDGAESDQQQQQQPLILERVEKETVKDWQSVVSHRLKKHCLCTPKKAKS
Sbjct  3926112  MTHGDDCCPTAEDGAESDQQQQQQPLILERVEKETVKDWQSVVSHRLKKHCLCTPKKAKS  3925933

Query  61       KILGFAPILKWLPRYQLGEWLLGDIMSGLIVGILLVPQSIAYSLLASQEPIYGLYTSFFS  120
                KILGFAPILKWLPRYQLGEWLLGDIMSGLIVGILLVPQSIAYSLLASQEPIYGLYTSFFS
Sbjct  3925932  KILGFAPILKWLPRYQLGEWLLGDIMSGLIVGILLVPQSIAYSLLASQEPIYGLYTSFFS  3925753

Query  121      SIIYAVLGTSKHISVGIFGVLCLLVGQVVDRELALAGYLTESSSISSNNSAILLAGQGND  180
                SIIYAVLGTSKHISVGIFGVLCLLVGQVVDRELALAGYLTESSSISSNNSAILLAGQGND
Sbjct  3925752  SIIYAVLGTSKHISVGIFGVLCLLVGQVVDRELALAGYLTESSSISSNNSAILLAGQGND  3925573

Query  181      SVGVECDRSCYAITVGATLTFTAGVYQV  208
                SVGVECDRSCYAITVGATLTFTAGVYQV
Sbjct  3925572  SVGVECDRSCYAITVGATLTFTAGVYQV  3925489
```

Range 3: 3923021 to 3923470

Score:553 bits(1128), Expect:2e-161,
Method:,
Identities:150/150(100%), Positives:150/150(100%), Gaps:0/150(0%)

```
Query  305      APIPFELFVVIIATLVSHFGHFNTDHGSGVVGDIPTGFLPPQLPMWTLIPNVAVDAFSIA  364
                APIPFELFVVIIATLVSHFGHFNTDHGSGVVGDIPTGFLPPQLPMWTLIPNVAVDAFSIA
Sbjct  3923470  APIPFELFVVIIATLVSHFGHFNTDHGSGVVGDIPTGFLPPQLPMWTLIPNVAVDAFSIA  3923291

Query  365      IVGFAITVSLSEMFAKKHGYTVDANQEMYAIGFCNILPSFFRCFTTSAALTKTLVKESTG  424
                IVGFAITVSLSEMFAKKHGYTVDANQEMYAIGFCNILPSFFRCFTTSAALTKTLVKESTG
Sbjct  3923290  IVGFAITVSLSEMFAKKHGYTVDANQEMYAIGFCNILPSFFRCFTTSAALTKTLVKESTG  3923111

Query  425      CQTQMSGLVSALVLLLVLLVIAPLFYSLQK  454
                CQTQMSGLVSALVLLLVLLVIAPLFYSLQK
Sbjct  3923110  CQTQMSGLVSALVLLLVLLVIAPLFYSLQK  3923021
```

Range 4: 3925050 to 3925343

Score:361 bits(736), Expect:2e-103,
Method:,
Identities:98/98(100%), Positives:98/98(100%), Gaps:0/98(0%)

```
Query  207      QVLMGIFQVGFVSVYLSDSLLSGFTTGASLTILTSQFKHLLGLKIPRPQGWFTLFKTWYS  266
                QVLMGIFQVGFVSVYLSDSLLSGFTTGASLTILTSQFKHLLGLKIPRPQGWFTLFKTWYS
Sbjct  3925343  QVLMGIFQVGFVSVYLSDSLLSGFTTGASLTILTSQFKHLLGLKIPRPQGWFTLFKTWYS  3925164

Query  267      LLVNLGNTNICDLVTSLVCLLILIPTKELNDRFKAKLK  304
                LLVNLGNTNICDLVTSLVCLLILIPTKELNDRFKAKLK
Sbjct  3925163  LLVNLGNTNICDLVTSLVCLLILIPTKELNDRFKAKLK  3925050
```

ER European seabass *slc26a2*

Query: sulfate transporter [Dicentrarchus labrax] Query ID: XP_051275855.1 Length: 725

>Dicentrarchus labrax unplaced genomic scaffold, dlabrax2021, whole genome shotgun sequence
Sequence ID: NW_026136713.1 Length: 32630001
Range 1: 27331528 to 27332361

Score:1013 bits(2068), Expect:0.0,
Method:,
Identities:276/278(99%), Positives:276/278(99%), Gaps:0/278(0%)

```
Query  448       LQKCVLAVIIVVNLRGALRKFMDIPRMWRVNHVDASIWVITMATSALVNTELGLLVGVLV  507
                 L  CVLAVIIVVNLRGALRKFMDIPRMWRVNHVDASIWVITMATSALVNTELGLLVGVLV
Sbjct  27332361  LSRCVLAVIIVVNLRGALRKFMDIPRMWRVNHVDASIWVITMATSALVNTELGLLVGVLV  27332182

Query  508       SALCVLGRTQKAQVLELGRATTREHYEDLLSYRGLGTHPGVAVFRYEAPIYYANQSLFKK  567
                 SALCVLGRTQKAQVLELGRATTREHYEDLLSYRGLGTHPGVAVFRYEAPIYYANQSLFKK
Sbjct  27332181  SALCVLGRTQKAQVLELGRATTREHYEDLLSYRGLGTHPGVAVFRYEAPIYYANQSLFKK  27332002

Query  568       SLYRCVGLDPVKEKTRIIKFKKKNKQQEEVTGGQNMKSEQKEAAGKEDVVEAGTTVTLMK  627
                 SLYRCVGLDPVKEKTRIIKFKKKNKQQEEVTGGQNMKSEQKEAAGKEDVVEAGTTVTLMK
Sbjct  27332001  SLYRCVGLDPVKEKTRIIKFKKKNKQQEEVTGGQNMKSEQKEAAGKEDVVEAGTTVTLMK  27331822

Query  628       SWRSLSSLVIDCSAILFLDTAGVNALKEVRKDYGELGIKVVLAQCNVSVLDDLERGGYYP  687
```

SWRSLSSLVIDCSAILFLDTAGVNALKEVRKDYGELGIKVVLAQCNVSVLDDLERGGYYP
Sbjct 27331821 SWRSLSSLVIDCSAILFLDTAGVNALKEVRKDYGELGIKVVLAQCNVSVLDDLERGGYYP 27331642

Query 688 EKKECDGGENKSVFFTIEDAVHYVQGLSTPNGDYDSKC 725
EKKECDGGENKSVFFTIEDAVHYVQGLSTPNGDYDSKC
Sbjct 27331641 EKKECDGGENKSVFFTIEDAVHYVQGLSTPNGDYDSKC 27331528

Range 2: 27335413 to 27336024

Score:746 bits(1522), Expect:0.0,
Method:,
Identities:204/204(100%), Positives:204/204(100%), Gaps:0/204(0%)

Query 1 MTHGDDCCATAEDGAESDQQRPLILERVEKDTAKNWKTLVSHRIKKHCLCTPKKAKSKIL 60
MTHGDDCCATAEDGAESDQQRPLILERVEKDTAKNWKTLVSHRIKKHCLCTPKKAKSKIL
Sbjct 27336024 MTHGDDCCATAEDGAESDQQRPLILERVEKDTAKNWKTLVSHRIKKHCLCTPKKAKSKIL 27335845

Query 61 GFVPILKWLPQYQLREWLLGDIMSGLIVGILLVPQSIAYSLLASQDPIYGLYTSFFSSII 120
GFVPILKWLPQYQLREWLLGDIMSGLIVGILLVPQSIAYSLLASQDPIYGLYTSFFSSII
Sbjct 27335844 GFVPILKWLPQYQLREWLLGDIMSGLIVGILLVPQSIAYSLLASQDPIYGLYTSFFSSII 27335665

Query 121 YALLGSSKHISVGIFGVLCLLVGQVVDRELALAGYLTESSISSNGSAILLAGQGNDSVGV 180
YALLGSSKHISVGIFGVLCLLVGQVVDRELALAGYLTESSISSNGSAILLAGQGNDSVGV
Sbjct 27335664 YALLGSSKHISVGIFGVLCLLVGQVVDRELALAGYLTESSISSNGSAILLAGQGNDSVGV 27335485

Query 181 ECDRSCYAITVGATVTFTAGVYQV 204
ECDRSCYAITVGATVTFTAGVYQV
Sbjct 27335484 ECDRSCYAITVGATVTFTAGVYQV 27335413

Range 3: 27333342 to 27333791

Score:551 bits(1123), Expect:9e-161,
Method:,
Identities:150/150(100%), Positives:150/150(100%), Gaps:0/150(0%)

Query 301 APIPFELFVVIIATLASHFGHFNTDYGSGVAGDIPTGFLPPQLPMWTLIPNVAVDAFSIA 360
APIPFELFVVIIATLASHFGHFNTDYGSGVAGDIPTGFLPPQLPMWTLIPNVAVDAFSIA
Sbjct 27333791 APIPFELFVVIIATLASHFGHFNTDYGSGVAGDIPTGFLPPQLPMWTLIPNVAVDAFSIA 27333612

Query 361 IVGFAITVSLSEMFAKKHGYTVDANQEMYAIGFCNILPSFFRCFTTSAALTKTLVKESTG 420
IVGFAITVSLSEMFAKKHGYTVDANQEMYAIGFCNILPSFFRCFTTSAALTKTLVKESTG
Sbjct 27333611 IVGFAITVSLSEMFAKKHGYTVDANQEMYAIGFCNILPSFFRCFTTSAALTKTLVKESTG 27333432

Query 421 CQTQVSGLVSALVLLLVLLVIAPLFYSLQK 450
CQTQVSGLVSALVLLLVLLVIAPLFYSLQK
Sbjct 27333431 CQTQVSGLVSALVLLLVLLVIAPLFYSLQK 27333342

Range 4: 27334995 to 27335288

Score:362 bits(737), Expect:1e-103,
Method:,
Identities:98/98(100%), Positives:98/98(100%), Gaps:0/98(0%)

Query 203 QVLMGIFQVGFVSVYLSDSLLSGFATGASLTILTSQFKYLLGLKIPRPQGWFTLFKTWYS 262
QVLMGIFQVGFVSVYLSDSLLSGFATGASLTILTSQFKYLLGLKIPRPQGWFTLFKTWYS
Sbjct 27335288 QVLMGIFQVGFVSVYLSDSLLSGFATGASLTILTSQFKYLLGLKIPRPQGWFTLFKTWYS 27335109

Query 263 LLINLGNTNICDLVTSLVCLLILIPTKELNDRFKAKLK 300
LLINLGNTNICDLVTSLVCLLILIPTKELNDRFKAKLK
Sbjct 27335108 LLINLGNTNICDLVTSLVCLLILIPTKELNDRFKAKLK 27334995

ES Large yellow croaker *slc26a2*

Query: sulfate transporter [Larimichthys crocea] Query ID: XP_019110735.2 Length: 718

>Larimichthys crocea isolate SSNF chromosome I, L_crocea_2.0, whole genome shotgun sequence
Sequence ID: NC_040011.1 Length: 43682218
Range 1: 13094086 to 13094895

Score:982 bits(2006), Expect:0.0,
Method:,
Identities:268/270(99%), Positives:268/270(99%), Gaps:0/270(0%)

Query 449 LQKCVLAVIIVVNLRGALRKFTEIPHMWRANRIDASIWLITMATSALVNTELGLLVGVLV 508
L CVLAVIIVVNLRGALRKFTEIPHMWRANRIDASIWLITMATSALVNTELGLLVGVLV
Sbjct 13094086 LPRCVLAVIIVVNLRGALRKFTEIPHMWRANRIDASIWLITMATSALVNTELGLLVGVLV 13094265

Query 509 SAFCVLGRTQQAQVLELGRATTREHYEDLSSYRGLGTHPGVAVFRYGAPIYYANQSLFKK 568
SAFCVLGRTQQAQVLELGRATTREHYEDLSSYRGLGTHPGVAVFRYGAPIYYANQSLFKK
Sbjct 13094266 SAFCVLGRTQQAQVLELGRATTREHYEDLSSYRGLGTHPGVAVFRYGAPIYYANQSLFKK 13094445

Query 569 SLYRCVGLDPVKEKAYRIKFNKKNKKQGKNTNMKSEEKEAAGKADEVEAGVTATFMNSSC 628

```
                 SLYRCVGLDPVKEKAYRIKFNKKNKKQGKNTNMKSEEKEAAGKADEVEAGVTATFMNSSC
Sbjct  13094446  SLYRCVGLDPVKEKAYRIKFNKKNKKQGKNTNMKSEEKEAAGKADEVEAGVTATFMNSSC  13094625

Query  629       SLRSLVIDCSTILFVDTAGVNALKEVRKDYGELGIKVVLAQCSASVLDDLERGGYYPGDE  688
                 SLRSLVIDCSTILFVDTAGVNALKEVRKDYGELGIKVVLAQCSASVLDDLERGGYYPGDE
Sbjct  13094626  SLRSLVIDCSTILFVDTAGVNALKEVRKDYGELGIKVVLAQCSASVLDDLERGGYYPGDE  13094805

Query  689       ENRVIFFTIADAVHYLQSLPTPNGDYDGKC  718
                 ENRVIFFTIADAVHYLQSLPTPNGDYDGKC
Sbjct  13094806  ENRVIFFTIADAVHYLQSLPTPNGDYDGKC  13094895
```

Range 2: 13090041 to 13090655

Score:751 bits(1533), Expect:0.0,
Method:,
Identities:205/205(100%), Positives:205/205(100%), Gaps:0/205(0%)

```
Query  1         MTHGDDCCTTAEDGVESDQQQQPLILERLEKQQDKNWKSVVSHQLKKRCSCTAERAKSKI  60
                 MTHGDDCCTTAEDGVESDQQQQPLILERLEKQQDKNWKSVVSHQLKKRCSCTAERAKSKI
Sbjct  13090041  MTHGDDCCTTAEDGVESDQQQQPLILERLEKQQDKNWKSVVSHQLKKRCSCTAERAKSKI  13090220

Query  61        LGFAPILRWLPRYQLREWLLGDAMSGLIVGILLVPQSIAYSLLASQDPIYGLYTSFFSSI  120
                 LGFAPILRWLPRYQLREWLLGDAMSGLIVGILLVPQSIAYSLLASQDPIYGLYTSFFSSI
Sbjct  13090221  LGFAPILRWLPRYQLREWLLGDAMSGLIVGILLVPQSIAYSLLASQDPIYGLYTSFFSSI  13090400

Query  121       IYAILGTSRHISVGIFGVLCLLVGQVVDRELALAGYLTESSVSSNSSGILLAGQRNDSVG  180
                 IYAILGTSRHISVGIFGVLCLLVGQVVDRELALAGYLTESSVSSNSSGILLAGQRNDSVG
Sbjct  13090401  IYAILGTSRHISVGIFGVLCLLVGQVVDRELALAGYLTESSVSSNSSGILLAGQRNDSVG  13090580

Query  181       VECDRSCYAITVGATVTFTAGVYQV  205
                 VECDRSCYAITVGATVTFTAGVYQV
Sbjct  13090581  VECDRSCYAITVGATVTFTAGVYQV  13090655
```

Range 3: 13090730 to 13091023

Score:360 bits(734), Expect:0.0,
Method:,
Identities:98/98(100%), Positives:98/98(100%), Gaps:0/98(0%)

```
Query  204       QVLMGIFQVGFVSVYLSNSLLSGFATGASLTILTSQFKYLLGLKIPRPQGWFTLFKTWYS  263
                 QVLMGIFQVGFVSVYLSNSLLSGFATGASLTILTSQFKYLLGLKIPRPQGWFTLFKTWYS
Sbjct  13090730  QVLMGIFQVGFVSVYLSNSLLSGFATGASLTILTSQFKYLLGLKIPRPQGWFTLFKTWYS  13090909

Query  264       LLSNLGNTNICDLVTSLVCLLVLIPTKELNDRFKGKLK  301
                 LLSNLGNTNICDLVTSLVCLLVLIPTKELNDRFKGKLK
Sbjct  13090910  LLSNLGNTNICDLVTSLVCLLVLIPTKELNDRFKGKLK  13091023
```

Range 4: 13091665 to 13092114

Score:553 bits(1127), Expect:2e-161,
Method:,
Identities:150/150(100%), Positives:150/150(100%), Gaps:0/150(0%)

```
Query  302       APIPFELFVVIIATLASHFGHFNTDYGSGVAGDIPTGFLPPQMPMWTLIPNVAVDAFSIA  361
                 APIPFELFVVIIATLASHFGHFNTDYGSGVAGDIPTGFLPPQMPMWTLIPNVAVDAFSIA
Sbjct  13091665  APIPFELFVVIIATLASHFGHFNTDYGSGVAGDIPTGFLPPQMPMWTLIPNVAVDAFSIA  13091844

Query  362       IVGFAITVSLSEMFAKKHGYTVDANQEMYAIGFCNILPSFFRCFTTSAALTKTLVKESTG  421
                 IVGFAITVSLSEMFAKKHGYTVDANQEMYAIGFCNILPSFFRCFTTSAALTKTLVKESTG
Sbjct  13091845  IVGFAITVSLSEMFAKKHGYTVDANQEMYAIGFCNILPSFFRCFTTSAALTKTLVKESTG  13092024

Query  422       CQTQVSGLVSALVLLLVLLVIAPLFYSLQK  451
                 CQTQVSGLVSALVLLLVLLVIAPLFYSLQK
Sbjct  13092025  CQTQVSGLVSALVLLLVLLVIAPLFYSLQK  13092114
```

ET Gilthead seabream *slc26a2*

Query: sulfate anion transporter 1 isoform X2 [Sparus aurata] Query ID: XP_030252838.1 Length: 728

>Sparus aurata chromosome 18, fSpaAur1.1, whole genome shotgun sequence
Sequence ID: NC_044204.1 Length: 37104330
Range 1: 5796185 to 5797021

Score:1017 bits(2076), Expect:0.0,
Method:,
Identities:277/279(99%), Positives:277/279(99%), Gaps:0/279(0%)

```
Query  450      LQKCVLAVIILVNLRGALKKFTDVPRMWRVNRVDASIWLITMATSALVNTELGLLVGVLV  509
                L  CVLAVIILVNLRGALKKFTDVPRMWRVNRVDASIWLITMATSALVNTELGLLVGVLV
Sbjct  5797021  LSRCVLAVIILVNLRGALKKFTDVPRMWRVNRVDASIWLITMATSALVNTELGLLVGVLV  5796842

Query  510      SAFCVLGRTQQAQVLELGRATNREHYEDLSSYRGLRTHPGVAVFRYDAPIYYANQSLFKK  569
```

```
                SAFCVLGRTQQAQVLELGRATNREHYEDLSSYRGLRTHPGVAVFRYDAPIYYANQSLFKK
Sbjct  5796841  SAFCVLGRTQQAQVLELGRATNREHYEDLSSYRGLRTHPGVAVFRYDAPIYYANQSLFKK  5796662

Query  570      SLYRCAGLDPVKEKTRRIKFQKKNKQQGETEEVQNMKSEEKEAAGKEEEVEAGATVTLMN  629
                SLYRCAGLDPVKEKTRRIKFQKKNKQQGETEEVQNMKSEEKEAAGKEEEVEAGATVTLMN
Sbjct  5796661  SLYRCAGLDPVKEKTRRIKFQKKNKQQGETEEVQNMKSEEKEAAGKEEEVEAGATVTLMN  5796482

Query  630      SSRSIRSLVIDCSSILFLDTAGVNALKEVRKDYGELGIKVVLAQCSASVLDDLERGGYYP  689
                SSRSIRSLVIDCSSILFLDTAGVNALKEVRKDYGELGIKVVLAQCSASVLDDLERGGYYP
Sbjct  5796481  SSRSIRSLVIDCSSILFLDTAGVNALKEVRKDYGELGIKVVLAQCSASVLDDLERGGYYP  5796302

Query  690      DQKGCDGGDDNKMIYFTIADAIYSIQSLPTPNGDYDKKC  728
                DQKGCDGGDDNKMIYFTIADAIYSIQSLPTPNGDYDKKC
Sbjct  5796301  DQKGCDGGDDNKMIYFTIADAIYSIQSLPTPNGDYDKKC  5796185
```

Range 2: 5800582 to 5801199

Score:752 bits(1535), Expect:0.0,
Method:,
Identities:206/206(100%), Positives:206/206(100%), Gaps:0/206(0%)

```
Query  1        MNHGDSHCATAEDDADGNQQRPLTLERVEKETKNFKSMVSHRLKKHCSCTPKKAKSKILG  60
                MNHGDSHCATAEDDADGNQQRPLTLERVEKETKNFKSMVSHRLKKHCSCTPKKAKSKILG
Sbjct  5801199  MNHGDSHCATAEDDADGNQQRPLTLERVEKETKNFKSMVSHRLKKHCSCTPKKAKSKILG  5801020

Query  61       FFPILRWLPRYQLRDWLLGDVMSGLIVGILLVPQSIAYSLLASQDPIYGLYTSFFSSIIY  120
                FFPILRWLPRYQLRDWLLGDVMSGLIVGILLVPQSIAYSLLASQDPIYGLYTSFFSSIIY
Sbjct  5801019  FFPILRWLPRYQLRDWLLGDVMSGLIVGILLVPQSIAYSLLASQDPIYGLYTSFFSSIIY  5800840

Query  121      ALLGTSRHISVGIFGVLCLLVGQVVDRELALAGYLTESSSSSISGNDSAILLAGQGNISV  180
                ALLGTSRHISVGIFGVLCLLVGQVVDRELALAGYLTESSSSSISGNDSAILLAGQGNISV
Sbjct  5800839  ALLGTSRHISVGIFGVLCLLVGQVVDRELALAGYLTESSSSSISGNDSAILLAGQGNISV  5800660

Query  181      AVGCDRSCYAITVGATVTFIAGVYQV  206
                AVGCDRSCYAITVGATVTFIAGVYQV
Sbjct  5800659  AVGCDRSCYAITVGATVTFIAGVYQV  5800582
```

Range 3: 5799283 to 5799732

Score:553 bits(1127), Expect:4e-161,
Method:,
Identities:150/150(100%), Positives:150/150(100%), Gaps:0/150(0%)

```
Query  303      APIPFELFVVIIATLASHFGHFNTDYGSGVAGDIPTGFLPPQLPMWSLIPNVAVDAFSIA  362
                APIPFELFVVIIATLASHFGHFNTDYGSGVAGDIPTGFLPPQLPMWSLIPNVAVDAFSIA
Sbjct  5799732  APIPFELFVVIIATLASHFGHFNTDYGSGVAGDIPTGFLPPQLPMWSLIPNVAVDAFSIA  5799553

Query  363      IVGFAITVSLSEMFAKKHGYTVDANQEMYAIGFCNILPSFFRCFTTSAALTKTLVKESTG  422
                IVGFAITVSLSEMFAKKHGYTVDANQEMYAIGFCNILPSFFRCFTTSAALTKTLVKESTG
Sbjct  5799552  IVGFAITVSLSEMFAKKHGYTVDANQEMYAIGFCNILPSFFRCFTTSAALTKTLVKESTG  5799373

Query  423      CQTQMSGLVTALVLLLVLLVIAPLFYSLQK  452
                CQTQMSGLVTALVLLLVLLVIAPLFYSLQK
Sbjct  5799372  CQTQMSGLVTALVLLLVLLVIAPLFYSLQK  5799283
```

Range 4: 5799895 to 5800191

Score:362 bits(738), Expect:1e-103,
Method:,
Identities:98/99(99%), Positives:99/99(100%), Gaps:0/99(0%)

```
Query  204      YQVLMGLFQVGFVSVYLSDSLLSGFATGASLTILTSQIKYLLGLKIPRPQGWFTLFKTWY  263
                +QVLMGLFQVGFVSVYLSDSLLSGFATGASLTILTSQIKYLLGLKIPRPQGWFTLFKTWY
Sbjct  5800191  FQVLMGLFQVGFVSVYLSDSLLSGFATGASLTILTSQIKYLLGLKIPRPQGWFTLFKTWY  5800012

Query  264      SLFANLGNTNICDLVTSLVCLLILIPTKEINDRFKAKLK  302
                SLFANLGNTNICDLVTSLVCLLILIPTKEINDRFKAKLK
Sbjct  5800011  SLFANLGNTNICDLVTSLVCLLILIPTKEINDRFKAKLK  5799895
```

EU Japanese pufferfish *slc26a2*

Query: sulfate transporter [Takifugu rubripes] Query ID: XP_003978276.2 Length: 717

>Takifugu rubripes chromosome 14, fTakRub1.2, whole genome shotgun sequence
Sequence ID: NC_042298.1 Length: 16036328
Range 1: 15889368 to 15890165

Score:964 bits(1969), Expect:0.0,
Method:,
Identities:266/266(100%), Positives:266/266(100%), Gaps:0/266(0%)

```
Query  452       CVLAVIIVVNLRGALQKFADIPRMWRVNRIDAAVWLVTMATSALVNTELGLLVGVMASAL  511
```

```
                CVLAVIIVVNLRGALQKFADIPRMWRVNRIDAAVWLVTMATSALVNTELGLLVGVMASAL
Sbjct  15890165  CVLAVIIVVNLRGALQKFADIPRMWRVNRIDAAVWLVTMATSALVNTELGLLVGVMASAL  15889986

Query  512       CVLGRTQRAQVLELGRTPSTEHYEALAAYRGLQTHPGVLVFRYAAPIYYANQSLFKRSLY  571
                 CVLGRTQRAQVLELGRTPSTEHYEALAAYRGLQTHPGVLVFRYAAPIYYANQSLFKRSLY
Sbjct  15889985  CVLGRTQRAQVLELGRTPSTEHYEALAAYRGLQTHPGVLVFRYAAPIYYANQSLFKRSLY  15889806

Query  572       RRAGLDPLQEKAQRLKFQKKKKAAADLAGAPNVSLKDDEGGAAVTLMTPPRSFHSLVIDC  631
                 RRAGLDPLQEKAQRLKFQKKKKAAADLAGAPNVSLKDDEGGAAVTLMTPPRSFHSLVIDC
Sbjct  15889805  RRAGLDPLQEKAQRLKFQKKKKAAADLAGAPNVSLKDDEGGAAVTLMTPPRSFHSLVIDC  15889626

Query  632       SAVLFVDTAGVTALKEVRKDYAAVGVKVVLAQCNPSVLDDLQRGGFFPDQSTHAAAAGTL  691
                 SAVLFVDTAGVTALKEVRKDYAAVGVKVVLAQCNPSVLDDLQRGGFFPDQSTHAAAAGTL
Sbjct  15889625  SAVLFVDTAGVTALKEVRKDYAAVGVKVVLAQCNPSVLDDLQRGGFFPDQSTHAAAAGTL  15889446

Query  692       VFFSIADAVHQVQRCSVGNGDYESKC  717
                 VFFSIADAVHQVQRCSVGNGDYESKC
Sbjct  15889445  VFFSIADAVHQVQRCSVGNGDYESKC  15889368
```

Range 2: 15891875 to 15892489

Score:744 bits(1519), Expect:0.0,
Method:,
Identities:205/205(100%), Positives:205/205(100%), Gaps:0/205(0%)

```
Query  1         MVLLGNDGGVAREGDEGEQQHALVLERVEKQPPASWRSLTSRRLKRCCSCSPQRVRSKVL  60
                 MVLLGNDGGVAREGDEGEQQHALVLERVEKQPPASWRSLTSRRLKRCCSCSPQRVRSKVL
Sbjct  15892489  MVLLGNDGGVAREGDEGEQQHALVLERVEKQPPASWRSLTSRRLKRCCSCSPQRVRSKVL  15892310

Query  61        GFLPILKWLPRYRLKEWLLGDVMSGLIVGILLVPQSIAYSLLASQDPIYGLYTSFFASII  120
                 GFLPILKWLPRYRLKEWLLGDVMSGLIVGILLVPQSIAYSLLASQDPIYGLYTSFFASII
Sbjct  15892309  GFLPILKWLPRYRLKEWLLGDVMSGLIVGILLVPQSIAYSLLASQDPIYGLYTSFFASII  15892130

Query  121       YALLGTSKHISVGIFGVLCLLVGQVVDRELALAGYLTERSGVGSNDSAALLAALGNNTSG  180
                 YALLGTSKHISVGIFGVLCLLVGQVVDRELALAGYLTERSGVGSNDSAALLAALGNNTSG
Sbjct  15892129  YALLGTSKHISVGIFGVLCLLVGQVVDRELALAGYLTERSGVGSNDSAALLAALGNNTSG  15891950

Query  181       VDCDRSCYAITVGATVTFTAGVYQV  205
                 VDCDRSCYAITVGATVTFTAGVYQV
Sbjct  15891949  VDCDRSCYAITVGATVTFTAGVYQV  15891875
```

Range 3: 15891506 to 15891799

Score:363 bits(739), Expect:0.0,
Method:,
Identities:98/98(100%), Positives:98/98(100%), Gaps:0/98(0%)

```
Query  204       QVLMGIFQVGFVSVYLSDSLLSGFATGASLTILTSQVKYILGLKFPRPQGWFTLFKTWYN  263
                 QVLMGIFQVGFVSVYLSDSLLSGFATGASLTILTSQVKYILGLKFPRPQGWFTLFKTWYN
Sbjct  15891799  QVLMGIFQVGFVSVYLSDSLLSGFATGASLTILTSQVKYILGLKFPRPQGWFTLFKTWYN  15891620

Query  264       LFANLGDTNVCDLVTSLVCLLILIPTKEINDRFKAKLK  301
                 LFANLGDTNVCDLVTSLVCLLILIPTKEINDRFKAKLK
Sbjct  15891619  LFANLGDTNVCDLVTSLVCLLILIPTKEINDRFKAKLK  15891506
```

Range 4: 15890907 to 15891356

Score:549 bits(1120), Expect:1e-160,
Method:,
Identities:150/150(100%), Positives:150/150(100%), Gaps:0/150(0%)

```
Query  302       APIPFELFVVIIATLASHFADFYNNYGSSVAGVIPTGFLPPRAPMWSLIPNVAVDAFSIA  361
                 APIPFELFVVIIATLASHFADFYNNYGSSVAGVIPTGFLPPRAPMWSLIPNVAVDAFSIA
Sbjct  15891356  APIPFELFVVIIATLASHFADFYNNYGSSVAGVIPTGFLPPRAPMWSLIPNVAVDAFSIA  15891177

Query  362       IVGFAITVSLSEMFAKKHGYSVDANQEMYAIGFCNILPSFFHCFSTSAALTKTLVKESTG  421
                 IVGFAITVSLSEMFAKKHGYSVDANQEMYAIGFCNILPSFFHCFSTSAALTKTLVKESTG
Sbjct  15891176  IVGFAITVSLSEMFAKKHGYSVDANQEMYAIGFCNILPSFFHCFSTSAALTKTLVKESTG  15890997

Query  422       CQSQVSGLVSGLVLLLVLLLIAPLFYSLQK  451
                 CQSQVSGLVSGLVLLLVLLLIAPLFYSLQK
Sbjct  15890996  CQSQVSGLVSGLVLLLVLLLIAPLFYSLQK  15890907
```

EV Three-spined stickleback *slc26a2*

Query: sulfate transporter [Gasterosteus aculeatus aculeatus] Query ID: XP_040031198.1 Length: 717

>Gasterosteus aculeatus aculeatus strain Lake Benthic chromosome 4, GAculeatus_UGA_version5, whole genome shotgun sequence
Sequence ID: NC_053215.1 Length: 34181212
Range 1: 4203055 to 4203879

Score:990 bits(2021), Expect:0.0,

Method:,
Identities:273/275(99%), Positives:273/275(99%), Gaps:0/275(0%)

```
Query  443      LQKCVLAVIILVNLRGALAKFLDVPAMWRVNRVDASIWLITMATSALVNTELGLLVGVLV  502
                L  CVLAVIILVNLRGALAKFLDVPAMWRVNRVDASIWLITMATSALVNTELGLLVGVLV
Sbjct  4203055  LPRCVLAVIILVNLRGALAKFLDVPAMWRVNRVDASIWLITMATSALVNTELGLLVGVLV  4203234

Query  503      SALCVLGRTQQARVLELGRAPTGEHYEDASSYRGLRTHPDVAVFRFEAPIYYANQSMFRK  562
                SALCVLGRTQQARVLELGRAPTGEHYEDASSYRGLRTHPDVAVFRFEAPIYYANQSMFRK
Sbjct  4203235  SALCVLGRTQQARVLELGRAPTGEHYEDASSYRGLRTHPDVAVFRFEAPIYYANQSMFRK  4203414

Query  563      SLYKRVGLDPVKEKTQLMKFKKKQQREEGGVPNGKSGETGGEGQKHDEVEAEGTVTLML  622
                SLYKRVGLDPVKEKTQLMKFKKKQQREEGGVPNGKSGETGGEGQKHDEVEAEGTVTLML
Sbjct  4203415  SLYKRVGLDPVKEKTQLMKFKKKQQREEGGVPNGKSGETGGEGQKHDEVEAEGTVTLML  4203594

Query  623      DHKPRLLRSLVIDCSAVLFLDTAGVNALKEVRKDYAELGVTVVLAQCSTSVLDSLQRGGY  682
                DHKPRLLRSLVIDCSAVLFLDTAGVNALKEVRKDYAELGVTVVLAQCSTSVLDSLQRGGY
Sbjct  4203595  DHKPRLLRSLVIDCSAVLFLDTAGVNALKEVRKDYAELGVTVVLAQCSTSVLDSLQRGGY  4203774

Query  683      CPVSGGENRIAFFSIADAVHHVQSLGAPNGGHGSE  717
                CPVSGGENRIAFFSIADAVHHVQSLGAPNGGHGSE
Sbjct  4203775  CPVSGGENRIAFFSIADAVHHVQSLGAPNGGHGSE  4203879
```

Range 2: 4199508 to 4200104

Score:720 bits(1469), Expect:0.0,
Method:,
Identities:199/199(100%), Positives:199/199(100%), Gaps:0/199(0%)

```
Query  1        MAPGSDDAAETEQRPLVLERVEKEPARGWRSAASTRLRKHCSCTSQKAKSQILGFVPILK  60
                MAPGSDDAAETEQRPLVLERVEKEPARGWRSAASTRLRKHCSCTSQKAKSQILGFVPILK
Sbjct  4199508  MAPGSDDAAETEQRPLVLERVEKEPARGWRSAASTRLRKHCSCTSQKAKSQILGFVPILK  4199687

Query  61       WLPRYQLREWLLGDAMSGLIVGILLVPQSIAYSLLASQDPIYGLYTSFFTSIIYAIFGTS  120
                WLPRYQLREWLLGDAMSGLIVGILLVPQSIAYSLLASQDPIYGLYTSFFTSIIYAIFGTS
Sbjct  4199688  WLPRYQLREWLLGDAMSGLIVGILLVPQSIAYSLLASQDPIYGLYTSFFTSIIYAIFGTS  4199867

Query  121      RHISAGVFGVLCLLVGQVVDRELALAGYITEGGDGIGGNDSALLLAGLGNGTAAAGCDKS  180
                RHISAGVFGVLCLLVGQVVDRELALAGYITEGGDGIGGNDSALLLAGLGNGTAAAGCDKS
Sbjct  4199868  RHISAGVFGVLCLLVGQVVDRELALAGYITEGGDGIGGNDSALLLAGLGNGTAAAGCDKS  4200047

Query  181      CYAITVGATVTFTAGVYQV  199
                CYAITVGATVTFTAGVYQV
Sbjct  4200048  CYAITVGATVTFTAGVYQV  4200104
```

Range 3: 4200587 to 4201036

Score:549 bits(1119), Expect:0.0,
Method:,
Identities:150/150(100%), Positives:150/150(100%), Gaps:0/150(0%)

```
Query  296      APIPFELFVVIIATLASHFGHFNTDFGSDVSGDIPTGFLPPQLPMWALIPNVAVDAFSIA  355
                APIPFELFVVIIATLASHFGHFNTDFGSDVSGDIPTGFLPPQLPMWALIPNVAVDAFSIA
Sbjct  4200587  APIPFELFVVIIATLASHFGHFNTDFGSDVSGDIPTGFLPPQLPMWALIPNVAVDAFSIA  4200766

Query  356      IVGFAITVSLSEMFAKKHGYAVDANQEMYAIGLCNILPSFFRCFTSSAALTKTLVKESTG  415
                IVGFAITVSLSEMFAKKHGYAVDANQEMYAIGLCNILPSFFRCFTSSAALTKTLVKESTG
Sbjct  4200767  IVGFAITVSLSEMFAKKHGYAVDANQEMYAIGLCNILPSFFRCFTSSAALTKTLVKESTG  4200946

Query  416      CQTQVSGLVSALVLLLVLLVIAPLFYSLQK  445
                CQTQVSGLVSALVLLLVLLVIAPLFYSLQK
Sbjct  4200947  CQTQVSGLVSALVLLLVLLVIAPLFYSLQK  4201036
```

Range 4: 4200173 to 4200466

Score:360 bits(733), Expect:0.0,
Method:,
Identities:98/98(100%), Positives:98/98(100%), Gaps:0/98(0%)

```
Query  198      QVLMGVFQVGFVSVYLSDSLLSGFATGASLTIFTSQFKYLLGLKIPRPQGWFVLFKTWRS  257
                QVLMGVFQVGFVSVYLSDSLLSGFATGASLTIFTSQFKYLLGLKIPRPQGWFVLFKTWRS
Sbjct  4200173  QVLMGVFQVGFVSVYLSDSLLSGFATGASLTIFTSQFKYLLGLKIPRPQGWFVLFKTWRS  4200352

Query  258      LLVNLGNTNVCDLVTSLVCLLVLIPVKELNNRFKAKLK  295
                LLVNLGNTNVCDLVTSLVCLLVLIPVKELNNRFKAKLK
Sbjct  4200353  LLVNLGNTNVCDLVTSLVCLLVLIPVKELNNRFKAKLK  4200466
```

EW Tristan klipfish *slc26a2*

Query: unnamed protein product Query ID: lcl|Query_328671 Length: 729

>Bovichtus diacanthus isolate 2004_03 flattened_line_8046, whole genome shotgun sequence
Sequence ID: QZNB01004029.1 Length: 35127

```
Range 1: 16560 to 17402

Score:1016 bits(2074), Expect:0.0,
Method:,
Identities:279/281(99%), Positives:279/281(99%), Gaps:0/281(0%)

Query  449    LQKCVLAVIILVNLRGALRKFTDIPSMWRVNRVDTSIWLITMATSALVNTELGLLVGVLV  508
              L  CVLAVIILVNLRGALRKFTDIPSMWRVNRVDTSIWLITMATSALVNTELGLLVGVLV
Sbjct  16560  LPRCVLAVIILVNLRGALRKFTDIPSMWRVNRVDTSIWLITMATSALVNTELGLLVGVLV  16739

Query  509    SALCVLGRTQQAQVLELGRATTREHYEELSSYRGLRTHPGVAVFRYDAPIYYANQSLFKK  568
              SALCVLGRTQQAQVLELGRATTREHYEELSSYRGLRTHPGVAVFRYDAPIYYANQSLFKK
Sbjct  16740  SALCVLGRTQQAQVLELGRATTREHYEELSSYRGLRTHPGVAVFRYDAPIYYANQSLFKK  16919

Query  569    SLYRCVGLDPVKEKTRIMKFKKKNKQQEEEAEVPNTKSAETEGAGKEDEVEAGATVTLVL  628
              SLYRCVGLDPVKEKTRIMKFKKKNKQQEEEAEVPNTKSAETEGAGKEDEVEAGATVTLVL
Sbjct  16920  SLYRCVGLDPVKEKTRIMKFKKKNKQQEEEAEVPNTKSAETEGAGKEDEVEAGATVTLVL  17099

Query  629    DNKSSRSLRSLVIDCSAILFLDTAGVNALKEVRKDYAELGIKVVLAQCNTSVLDALEKGG  688
              DNKSSRSLRSLVIDCSAILFLDTAGVNALKEVRKDYAELGIKVVLAQCNTSVLDALEKGG
Sbjct  17100  DNKSSRSLRSLVIDCSAILFLDTAGVNALKEVRKDYAELGIKVVLAQCNTSVLDALEKGG  17279

Query  689    YYPEKKGCDGGEDKIIFFTIADAVRYVQSLSAPNGDYDSKC  729
              YYPEKKGCDGGEDKIIFFTIADAVRYVQSLSAPNGDYDSKC
Sbjct  17280  YYPEKKGCDGGEDKIIFFTIADAVRYVQSLSAPNGDYDSKC  17402



Range 2: 13356 to 13970

Score:749 bits(1529), Expect:0.0,
Method:,
Identities:205/205(100%), Positives:205/205(100%), Gaps:0/205(0%)

Query  1      MTHGDECCTTAEDGSESNQQQPLILERVEKETPKSWQSVVTHRLKKHCSCTPKKAKSKIL  60
              MTHGDECCTTAEDGSESNQQQPLILERVEKETPKSWQSVVTHRLKKHCSCTPKKAKSKIL
Sbjct  13356  MTHGDECCTTAEDGSESNQQQPLILERVEKETPKSWQSVVTHRLKKHCSCTPKKAKSKIL  13535

Query  61     GFVPILKWLPRYQLREWLLGDVMSGLIVGILLVPQSIAYSLLASQDPIYGLYTSFFASII  120
              GFVPILKWLPRYQLREWLLGDVMSGLIVGILLVPQSIAYSLLASQDPIYGLYTSFFASII
Sbjct  13536  GFVPILKWLPRYQLREWLLGDVMSGLIVGILLVPQSIAYSLLASQDPIYGLYTSFFASII  13715

Query  121    YTLLGTSRHISVGIFGVLCLLVGQVVDRELALAGYLTESGSISSNDSAILLAGQGNGSVG  180
              YTLLGTSRHISVGIFGVLCLLVGQVVDRELALAGYLTESGSISSNDSAILLAGQGNGSVG
Sbjct  13716  YTLLGTSRHISVGIFGVLCLLVGQVVDRELALAGYLTESGSISSNDSAILLAGQGNGSVG  13895

Query  181    VVCDRSCYAITVGATVTFTAGVYQV  205
              VVCDRSCYAITVGATVTFTAGVYQV
Sbjct  13896  VVCDRSCYAITVGATVTFTAGVYQV  13970



Range 3: 14667 to 15116

Score:549 bits(1120), Expect:8e-155,
Method:,
Identities:150/150(100%), Positives:150/150(100%), Gaps:0/150(0%)

Query  302    APIPFELFVVVIATLASHFGHFNTDYGSGVAGNIPTGFLPPQLPLWTLIPNVAVDAFSIA  361
              APIPFELFVVVIATLASHFGHFNTDYGSGVAGNIPTGFLPPQLPLWTLIPNVAVDAFSIA
Sbjct  14667  APIPFELFVVVIATLASHFGHFNTDYGSGVAGNIPTGFLPPQLPLWTLIPNVAVDAFSIA  14846

Query  362    IVGFAITVSLSEMFAKKHGYTVDANQEMYAIGFCNILPSFFRCFTTSAALTKTLVKESTG  421
              IVGFAITVSLSEMFAKKHGYTVDANQEMYAIGFCNILPSFFRCFTTSAALTKTLVKESTG
Sbjct  14847  IVGFAITVSLSEMFAKKHGYTVDANQEMYAIGFCNILPSFFRCFTTSAALTKTLVKESTG  15026

Query  422    CQTQISGLISALVLLLVLLVIAPLFYSLQK  451
              CQTQISGLISALVLLLVLLVIAPLFYSLQK
Sbjct  15027  CQTQISGLISALVLLLVLLVIAPLFYSLQK  15116



Range 4: 14180 to 14473

Score:364 bits(741), Expect:6e-99,
Method:,
Identities:98/98(100%), Positives:98/98(100%), Gaps:0/98(0%)

Query  204    QVLMGIFQVGFVSVYLSDSLLSGFATGASLTILTSQFKYLLGLKIPRPQGWFTLFKTWYS  263
              QVLMGIFQVGFVSVYLSDSLLSGFATGASLTILTSQFKYLLGLKIPRPQGWFTLFKTWYS
Sbjct  14180  QVLMGIFQVGFVSVYLSDSLLSGFATGASLTILTSQFKYLLGLKIPRPQGWFTLFKTWYS  14359

Query  264    LFTNLKNTNMCDLVTSLVCLLILLPTKELNDRFKAKLK  301
              LFTNLKNTNMCDLVTSLVCLLILLPTKELNDRFKAKLK
Sbjct  14360  LFTNLKNTNMCDLVTSLVCLLILLPTKELNDRFKAKLK  14473
```

EX Patagonian blennie *slc26a2*

Query: unnamed protein product Query ID: lcl|Query_804249 Length: 708

```
>Eleginops maclovinus isolate 2004_01 flattened_line_19857, whole genome shotgun sequence
Sequence ID: QZNA01009953.1 Length: 13748
Range 1: 6326 to 7096

Score:935 bits(1910), Expect:0.0,
Method:,
Identities:257/257(100%), Positives:257/257(100%), Gaps:0/257(0%)

Query  452   CVLAVIILVNLRGALRKFLDIPSMWRVNRVDTSIWLVTMATSALVNTELGLLVGVLVSAL  511
             CVLAVIILVNLRGALRKFLDIPSMWRVNRVDTSIWLVTMATSALVNTELGLLVGVLVSAL
Sbjct  6326  CVLAVIILVNLRGALRKFLDIPSMWRVNRVDTSIWLVTMATSALVNTELGLLVGVLVSAL  6505

Query  512   CVIGRTQQAKVLELGRASTRGHYEDVSAYRGLRTHPGVAVFRYDAPIYYANQSLFKRSLY  571
             CVIGRTQQAKVLELGRASTRGHYEDVSAYRGLRTHPGVAVFRYDAPIYYANQSLFKRSLY
Sbjct  6506  CVIGRTQQAKVLELGRASTRGHYEDVSAYRGLRTHPGVAVFRYDAPIYYANQSLFKRSLY  6685

Query  572   KSAGLDPLKEKSRVMKFKKKSKVPDVNGTGKEEEAGVMLENKPLRSVVIDGSAISFLDTA  631
             KSAGLDPLKEKSRVMKFKKKSKVPDVNGTGKEEEAGVMLENKPLRSVVIDGSAISFLDTA
Sbjct  6686  KSAGLDPLKEKSRVMKFKKKSKVPDVNGTGKEEEAGVMLENKPLRSVVIDGSAISFLDTA  6865

Query  632   GVGALKEVRKDYAELGVQVVLAQCSTSVLDSLQRGGYYPDKKESDAGEETMIFYTIADAV  691
             GVGALKEVRKDYAELGVQVVLAQCSTSVLDSLQRGGYYPDKKESDAGEETMIFYTIADAV
Sbjct  6866  GVGALKEVRKDYAELGVQVVLAQCSTSVLDSLQRGGYYPDKKESDAGEETMIFYTIADAV  7045

Query  692   HHVQSLSAPNGDYDRKC  708
             HHVQSLSAPNGDYDRKC
Sbjct  7046  HHVQSLSAPNGDYDRKC  7096



Range 2: 1848 to 2462

Score:750 bits(1531), Expect:0.0,
Method:,
Identities:205/205(100%), Positives:205/205(100%), Gaps:0/205(0%)

Query  1     MTHADDCCTTAEDGVESDQQQPLILERVVEETKSWQSVVTHRLKKNCSCSPKKAKSKILG  60
             MTHADDCCTTAEDGVESDQQQPLILERVVEETKSWQSVVTHRLKKNCSCSPKKAKSKILG
Sbjct  1848  MTHADDCCTTAEDGVESDQQQPLILERVVEETKSWQSVVTHRLKKNCSCSPKKAKSKILG  2027

Query  61    FVPIVKWLPSYQLREWLLGDVMSGLIVGILLVPQSIAYSLLASQDPIYGLYTSFFASIIY  120
             FVPIVKWLPSYQLREWLLGDVMSGLIVGILLVPQSIAYSLLASQDPIYGLYTSFFASIIY
Sbjct  2028  FVPIVKWLPSYQLREWLLGDVMSGLIVGILLVPQSIAYSLLASQDPIYGLYTSFFASIIY  2207

Query  121   ALLGTSRHISVGIFGVLCLLVGQVVDRELALAGYLPESSSLLSSNDSALLLAGQGNGTFG  180
             ALLGTSRHISVGIFGVLCLLVGQVVDRELALAGYLPESSSLLSSNDSALLLAGQGNGTFG
Sbjct  2208  ALLGTSRHISVGIFGVLCLLVGQVVDRELALAGYLPESSSLLSSNDSALLLAGQGNGTFG  2387

Query  181   MDCDRSCYAITVGATVTFTAGVYQV  205
             MDCDRSCYAITVGATVTFTAGVYQV
Sbjct  2388  MDCDRSCYAITVGATVTFTAGVYQV  2462



Range 3: 4491 to 4940

Score:550 bits(1122), Expect:2e-155,
Method:,
Identities:150/150(100%), Positives:150/150(100%), Gaps:0/150(0%)

Query  302   APIPFELFVVVIATLASHFGNFNTDYGSGVAGTIPTGFLPPQMPLWSLIPNVAVDAFSIA  361
             APIPFELFVVVIATLASHFGNFNTDYGSGVAGTIPTGFLPPQMPLWSLIPNVAVDAFSIA
Sbjct  4491  APIPFELFVVVIATLASHFGNFNTDYGSGVAGTIPTGFLPPQMPLWSLIPNVAVDAFSIA  4670

Query  362   IVGFAITVSLSEMFAKKHGYTVDANQEMYAIGFCNILPSFFRCFTTSAALTKTLVKESTG  421
             IVGFAITVSLSEMFAKKHGYTVDANQEMYAIGFCNILPSFFRCFTTSAALTKTLVKESTG
Sbjct  4671  IVGFAITVSLSEMFAKKHGYTVDANQEMYAIGFCNILPSFFRCFTTSAALTKTLVKESTG  4850

Query  422   CQTQISGIISALVLLLVLLVIAPLFYSLQK  451
             CQTQISGIISALVLLLVLLVIAPLFYSLQK
Sbjct  4851  CQTQISGIISALVLLLVLLVIAPLFYSLQK  4940



Range 4: 3411 to 3704

Score:362 bits(737), Expect:2e-98,
Method:,
Identities:98/98(100%), Positives:98/98(100%), Gaps:0/98(0%)

Query  204   QVLMGIFQVGFVSVYLSDSLLSGFATGASLTILTSQFKYVLGLKIPRPQGWFTLFKTWYG  263
             QVLMGIFQVGFVSVYLSDSLLSGFATGASLTILTSQFKYVLGLKIPRPQGWFTLFKTWYG
Sbjct  3411  QVLMGIFQVGFVSVYLSDSLLSGFATGASLTILTSQFKYVLGLKIPRPQGWFTLFKTWYG  3590

Query  264   VLSNLQHTNICDLVTSLVCLLILLPTKELNDRFKSKLK  301
             VLSNLQHTNICDLVTSLVCLLILLPTKELNDRFKSKLK
Sbjct  3591  VLSNLQHTNICDLVTSLVCLLILLPTKELNDRFKSKLK  3704
```

EY Emerald rockcod *slc26a2*

Query: sulfate transporter [Trematomus bernacchii] Query ID: XP_033991302.1 Length: 707

>Trematomus bernacchii unplaced genomic scaffold, fTreBer1.1, whole genome shotgun sequence
Sequence ID: NW_022987660.1 Length: 1015485
Range 1: 321106 to 321708

Score:740 bits(1510), Expect:0.0,
Method:,
Identities:201/201(100%), Positives:201/201(100%), Gaps:0/201(0%)

```
Query  1       MTDADDFCPTAEDGAESDQQHPLILQRVQEEPKSWQTAVSNRLKKNCSCTPKKAKSKILG  60
               MTDADDFCPTAEDGAESDQQHPLILQRVQEEPKSWQTAVSNRLKKNCSCTPKKAKSKILG
Sbjct  321708  MTDADDFCPTAEDGAESDQQHPLILQRVQEEPKSWQTAVSNRLKKNCSCTPKKAKSKILG  321529

Query  61      FVPIVKWLPRYQLKEWILGDLMSGLIVGILLVPQSIAYSLLASQDPIYGLYTSFFASIIY  120
               FVPIVKWLPRYQLKEWILGDLMSGLIVGILLVPQSIAYSLLASQDPIYGLYTSFFASIIY
Sbjct  321528  FVPIVKWLPRYQLKEWILGDLMSGLIVGILLVPQSIAYSLLASQDPIYGLYTSFFASIIY  321349

Query  121     ALLGTSRHISVGIFGVLCLLVGQVVDRELALAGYLPENSILSSNDSSILLGNSSFGMDCD  180
               ALLGTSRHISVGIFGVLCLLVGQVVDRELALAGYLPENSILSSNDSSILLGNSSFGMDCD
Sbjct  321348  ALLGTSRHISVGIFGVLCLLVGQVVDRELALAGYLPENSILSSNDSSILLGNSSFGMDCD  321169

Query  181     RSCYAITVGATVTFTAGVYQV  201
               RSCYAITVGATVTFTAGVYQV
Sbjct  321168  RSCYAITVGATVTFTAGVYQV  321106
```

Range 2: 306674 to 307135

Score:567 bits(1157), Expect:4e-165,
Method:,
Identities:154/154(100%), Positives:154/154(100%), Gaps:0/154(0%)

```
Query  448     CVLAVIILVNLRGALRKFLDIPSMWRVNRVDTSIWLITMATSALVNTELGLLVGVLVSAL  507
               CVLAVIILVNLRGALRKFLDIPSMWRVNRVDTSIWLITMATSALVNTELGLLVGVLVSAL
Sbjct  307135  CVLAVIILVNLRGALRKFLDIPSMWRVNRVDTSIWLITMATSALVNTELGLLVGVLVSAL  306956

Query  508     CVLARTQQAKVLKLGRASNREYYEDVSSYRGLQTHPGVAVFRYDAPIYYANQSLFKKSLY  567
               CVLARTQQAKVLKLGRASNREYYEDVSSYRGLQTHPGVAVFRYDAPIYYANQSLFKKSLY
Sbjct  306955  CVLARTQQAKVLKLGRASNREYYEDVSSYRGLQTHPGVAVFRYDAPIYYANQSLFKKSLY  306776

Query  568     KSAGLDPLKEKSRVMKFKNKKEKQQDVPDVKPTH  601
               KSAGLDPLKEKSRVMKFKNKKEKQQDVPDVKPTH
Sbjct  306775  KSAGLDPLKEKSRVMKFKNKKEKQQDVPDVKPTH  306674
```

Range 3: 312850 to 313221

Score:453 bits(924), Expect:9e-131,
Method:,
Identities:124/124(100%), Positives:124/124(100%), Gaps:0/124(0%)

```
Query  324     GSGVAGTIPTGFLPPQMPLWSLIPNVAVDAFSIAIVGFAITVSLSEMFAKKHGYTVDANQ  383
               GSGVAGTIPTGFLPPQMPLWSLIPNVAVDAFSIAIVGFAITVSLSEMFAKKHGYTVDANQ
Sbjct  313221  GSGVAGTIPTGFLPPQMPLWSLIPNVAVDAFSIAIVGFAITVSLSEMFAKKHGYTVDANQ  313042

Query  384     EMYAIGFCNILPSFFRCFTTSAALTKTLVKESTGCQTQISGIISALVLLLVLLVIAPLFY  443
               EMYAIGFCNILPSFFRCFTTSAALTKTLVKESTGCQTQISGIISALVLLLVLLVIAPLFY
Sbjct  313041  EMYAIGFCNILPSFFRCFTTSAALTKTLVKESTGCQTQISGIISALVLLLVLLVIAPLFY  312862

Query  444     SLQK  447
               SLQK
Sbjct  312861  SLQK  312850
```

Range 4: 304267 to 304584

Score:384 bits(782), Expect:8e-110,
Method:,
Identities:106/106(100%), Positives:106/106(100%), Gaps:0/106(0%)

```
Query  602     EEEAGVTLMENKSLRSVVIDGSSISFLDTAGVNALKEVRKDYAELGVKVVLAQCNTSVLD  661
               EEEAGVTLMENKSLRSVVIDGSSISFLDTAGVNALKEVRKDYAELGVKVVLAQCNTSVLD
Sbjct  304584  EEEAGVTLMENKSLRSVVIDGSSISFLDTAGVNALKEVRKDYAELGVKVVLAQCNTSVLD  304405

Query  662     SLQRGGYYPKTESDGGEETVIFYTIEDAVHYVQNLSAANGDHDSKC  707
               SLQRGGYYPKTESDGGEETVIFYTIEDAVHYVQNLSAANGDHDSKC
Sbjct  304404  SLQRGGYYPKTESDGGEETVIFYTIEDAVHYVQNLSAANGDHDSKC  304267
```

Range 5: 318920 to 319213

Score:362 bits(738), Expect:2e-103,
Method:,
Identities:98/98(100%), Positives:98/98(100%), Gaps:0/98(0%)

```
Query  200     QVLMGIFQVGFVSVYLSDSLLSGFATGASLTILTSQFKYLLGLKIPRPQGWFTLFKTWYS  259
               QVLMGIFQVGFVSVYLSDSLLSGFATGASLTILTSQFKYLLGLKIPRPQGWFTLFKTWYS
Sbjct  319213  QVLMGIFQVGFVSVYLSDSLLSGFATGASLTILTSQFKYLLGLKIPRPQGWFTLFKTWYS  319034

Query  260     VLSNLQNTNACDLVTSLVCLMILLPTKELNDRFKAKLK  297
               VLSNLQNTNACDLVTSLVCLMILLPTKELNDRFKAKLK
Sbjct  319033  VLSNLQNTNACDLVTSLVCLMILLPTKELNDRFKAKLK  318920
```

Range 6: 315822 to 315902

Score:101 bits(204), Expect:1e-24,
Method:,
Identities:27/27(100%), Positives:27/27(100%), Gaps:0/27(0%)

```
Query  298     APIPFELFVVVIATLASHFGNFNTDYG  324
               APIPFELFVVVIATLASHFGNFNTDYG
Sbjct  315902  APIPFELFVVVIATLASHFGNFNTDYG  315822
```

EZ Marbled rockcod *slc26a2*

Query: unnamed protein product Query ID: lcl|Query_1843643 Length: 707

>Notothenia rossii genome assembly, contig: atg002755l_1, whole genome shotgun sequence
Sequence ID: CATIUU010002413.1 Length: 90101
Range 1: 38424 to 39032

Score:741 bits(1513), Expect:0.0,
Method:,
Identities:201/203(99%), Positives:203/203(100%), Gaps:0/203(0%)

```
Query  1      MTDADDFCPTAEDGAESDQQHPLILQRVQEEPKSWQTVVSNRLKKNCSCTPKKAKSKILG  60
              MTDADDFCPTAEDGAESDQQHPLILQRVQEEPKSWQTVVSNRLKKNCSCTPKKAKSKILG
Sbjct  38424  MTDADDFCPTAEDGAESDQQHPLILQRVQEEPKSWQTVVSNRLKKNCSCTPKKAKSKILG  38603

Query  61     FVPIVKWLPRYQLKEWILGDVMSGLIVGILLVPQSIAYSLLASQDPIYGLYTSFFASIIY  120
              FVPIVKWLPRYQLKEWILGDVMSGLIVGILLVPQSIAYSLLASQDPIYGLYTSFFASIIY
Sbjct  38604  FVPIVKWLPRYQLKEWILGDVMSGLIVGILLVPQSIAYSLLASQDPIYGLYTSFFASIIY  38783

Query  121    ALLGTSRHISVGIFGVLCLLVGQVVDRELALAGYLPENSILSSNDSSIVLGNSSFGMDCD  180
              ALLGTSRHISVGIFGVLCLLVGQVVDRELALAGYLPENSILSSNDSSIVLGNSSFGMDCD
Sbjct  38784  ALLGTSRHISVGIFGVLCLLVGQVVDRELALAGYLPENSILSSNDSSIVLGNSSFGMDCD  38963

Query  181    RSCYAITVGATVTFTAGVYQVLM  203
              RSCYAITVGATVTFTAGVYQV++
Sbjct  38964  RSCYAITVGATVTFTAGVYQVML  39032
```

Range 2: 52866 to 53327

Score:566 bits(1155), Expect:8e-160,
Method:,
Identities:154/154(100%), Positives:154/154(100%), Gaps:0/154(0%)

```
Query  448    CVLAVIILVNLRGALRKFLDIPSMWRVNRVDTSTWLITMATSALVNTELGLLVGVLVSAL  507
              CVLAVIILVNLRGALRKFLDIPSMWRVNRVDTSTWLITMATSALVNTELGLLVGVLVSAL
Sbjct  52866  CVLAVIILVNLRGALRKFLDIPSMWRVNRVDTSTWLITMATSALVNTELGLLVGVLVSAL  53045

Query  508    CVLARTQQAKVLKLGRASNREYYEDVSSYRGLQTHPGVAVFRYDAPIYYANQSLFKKSLY  567
              CVLARTQQAKVLKLGRASNREYYEDVSSYRGLQTHPGVAVFRYDAPIYYANQSLFKKSLY
Sbjct  53046  CVLARTQQAKVLKLGRASNREYYEDVSSYRGLQTHPGVAVFRYDAPIYYANQSLFKKSLY  53225

Query  568    KSAGLDPLKEKSRVMKFKKKKEKQQEVPDVKPTH  601
              KSAGLDPLKEKSRVMKFKKKKEKQQEVPDVKPTH
Sbjct  53226  KSAGLDPLKEKSRVMKFKKKKEKQQEVPDVKPTH  53327
```

Range 3: 46946 to 47317

Score:453 bits(924), Expect:1e-125,
Method:,
Identities:124/124(100%), Positives:124/124(100%), Gaps:0/124(0%)

```
Query  324    GSGVAGTIPTGFLPPQMPLWSLIPNVAVDAFSIAIVGFAITVSLSEMFAKKHGYTVDANQ  383
              GSGVAGTIPTGFLPPQMPLWSLIPNVAVDAFSIAIVGFAITVSLSEMFAKKHGYTVDANQ
Sbjct  46946  GSGVAGTIPTGFLPPQMPLWSLIPNVAVDAFSIAIVGFAITVSLSEMFAKKHGYTVDANQ  47125

Query  384    EMYAIGFCNILPSFFRCFTTSAALTKTLVKESTGCQTQISGIISALVLLLVLLVIAPLFY  443
              EMYAIGFCNILPSFFRCFTTSAALTKTLVKESTGCQTQISGIISALVLLLVLLVIAPLFY
Sbjct  47126  EMYAIGFCNILPSFFRCFTTSAALTKTLVKESTGCQTQISGIISALVLLLVLLVIAPLFY  47305

Query  444    SLQK  447
              SLQK
Sbjct  47306  SLQK  47317
```

Range 4: 59486 to 59803

Score:383 bits(780), Expect:2e-104,
Method:,
Identities:106/106(100%), Positives:106/106(100%), Gaps:0/106(0%)

```
Query  602    EDEAGVTLMENTSLRSVVIDGSSISFLDTAGVNALKEVRKDYAELGVKVVLAQCNTSVLD  661
              EDEAGVTLMENTSLRSVVIDGSSISFLDTAGVNALKEVRKDYAELGVKVVLAQCNTSVLD
Sbjct  59486  EDEAGVTLMENTSLRSVVIDGSSISFLDTAGVNALKEVRKDYAELGVKVVLAQCNTSVLD  59665

Query  662    SLQRGGYYPKTESDGGGEALIFYTIEDAVHYVQNLPAANGDHKSKC  707
              SLQRGGYYPKTESDGGGEALIFYTIEDAVHYVQNLPAANGDHKSKC
Sbjct  59666  SLQRGGYYPKTESDGGGEALIFYTIEDAVHYVQNLPAANGDHKSKC  59803
```

Range 5: 43012 to 43305

Score:363 bits(739), Expect:2e-98,
Method:,
Identities:98/98(100%), Positives:98/98(100%), Gaps:0/98(0%)

```
Query  200    QVLMGIFQVGFVSVYLSDSLLSGFATGASLTILTSQFKYLLGLKIPRPQGWFTLFKTWYS  259
              QVLMGIFQVGFVSVYLSDSLLSGFATGASLTILTSQFKYLLGLKIPRPQGWFTLFKTWYS
Sbjct  43012  QVLMGIFQVGFVSVYLSDSLLSGFATGASLTILTSQFKYLLGLKIPRPQGWFTLFKTWYS  43191

Query  260    VLSNLQNTNVCDLVTSLVCLMILLPTKELNDRFKAKLK  297
              VLSNLQNTNVCDLVTSLVCLMILLPTKELNDRFKAKLK
Sbjct  43192  VLSNLQNTNVCDLVTSLVCLMILLPTKELNDRFKAKLK  43305
```

Range 6: 45830 to 45910

Score:102 bits(205), Expect:9e-20,
Method:,
Identities:27/27(100%), Positives:27/27(100%), Gaps:0/27(0%)

```
Query  298    APIPFELFVVVIATLASHFGNFNIDYG  324
              APIPFELFVVVIATLASHFGNFNIDYG
Sbjct  45830  APIPFELFVVVIATLASHFGNFNIDYG  45910
```

FA Blackfin icefish *slc26a2*

Query: unnamed protein product Query ID: lcl|Query_1885865 Length: 705

>Chaenocephalus aceratus isolate KU_202001 CAv2_01299, whole genome shotgun sequence
Sequence ID: JAMFTG010001299.1 Length: 63882
Range 1: 33737 to 34237

Score:603 bits(1231), Expect:0.0,
Method:,
Identities:165/167(99%), Positives:167/167(100%), Gaps:0/167(0%)

```
Query  37     TVASNRLKKNCSCTPKKAKSKILGFVPIVKWLPRYQLKEWILGDVMSGLIVGILLVPQSI  96
              TVASNRLKKNCSCTPKKAKSKILGFVPIVKWLPRYQLKEWILGDVMSGLIVGILLVPQSI
Sbjct  33737  TVASNRLKKNCSCTPKKAKSKILGFVPIVKWLPRYQLKEWILGDVMSGLIVGILLVPQSI  33916

Query  97     AYSLLASQDPIYGLYTSFFASIIYAILGTSRHISVGIFGVLCLLVGQVVDRELALAGYLP  156
              AYSLLASQDPIYGLYTSFFASIIYAILGTSRHISVGIFGVLCLLVGQVVDRELALAGYLP
Sbjct  33917  AYSLLASQDPIYGLYTSFFASIIYAILGTSRHISVGIFGVLCLLVGQVVDRELALAGYLP  34096

Query  157    ENSILSSNDSSILLGNGSFGMDCDRSCYAITVGATVTFTAGVYQVLM  203
              ENSILSSNDSSILLGNGSFGMDCDRSCYAITVGATVTFTAGVYQV++
Sbjct  34097  ENSILSSNDSSILLGNGSFGMDCDRSCYAITVGATVTFTAGVYQVML  34237
```

Range 2: 33628 to 33735

Score:136 bits(275), Expect:0.0,
Method:,
Identities:35/36(97%), Positives:35/36(97%), Gaps:0/36(0%)

```
Query  1      MTDADDFCPTAEDGAESDQQHPLILQRVQEEPKSWQ  36
              MTD DDFCPTAEDGAESDQQHPLILQRVQEEPKSWQ
Sbjct  33628  MTDTDDFCPTAEDGAESDQQHPLILQRVQEEPKSWQ  33735
```

Range 3: 51611 to 52066

Score:557 bits(1137), Expect:3e-157,
Method:,

Identities:152/152(100%), Positives:152/152(100%), Gaps:0/152(0%)

```
Query  448    CVLAVIILVNLRGALRKFLDLPSMWRANRVDTAIWLVTMATSALVNTELGLLVGVLVSAL  507
              CVLAVIILVNLRGALRKFLDLPSMWRANRVDTAIWLVTMATSALVNTELGLLVGVLVSAL
Sbjct  51611  CVLAVIILVNLRGALRKFLDLPSMWRANRVDTAIWLVTMATSALVNTELGLLVGVLVSAL  51790

Query  508    CVLARTQQAKVLKLGRASNREYYEDVSSYRGLQTHPGVAVFRYDAPIYYANQSLFKKSLY  567
              CVLARTQQAKVLKLGRASNREYYEDVSSYRGLQTHPGVAVFRYDAPIYYANQSLFKKSLY
Sbjct  51791  CVLARTQQAKVLKLGRASNREYYEDVSSYRGLQTHPGVAVFRYDAPIYYANQSLFKKSLY  51970

Query  568    KSAGLDPLREKSRAMKFKRKKKQDVPEAKPTH  599
              KSAGLDPLREKSRAMKFKRKKKQDVPEAKPTH
Sbjct  51971  KSAGLDPLREKSRAMKFKRKKKQDVPEAKPTH  52066
```

Range 4: 43180 to 43551

Score:453 bits(923), Expect:1e-125,
Method:,
Identities:124/124(100%), Positives:124/124(100%), Gaps:0/124(0%)

```
Query  324    GSGVAGTIPTGFLPPQMPLWSLIPNVAVDAFSIAIVGFAITVSLSEMFAKKHGYTVDANQ  383
              GSGVAGTIPTGFLPPQMPLWSLIPNVAVDAFSIAIVGFAITVSLSEMFAKKHGYTVDANQ
Sbjct  43180  GSGVAGTIPTGFLPPQMPLWSLIPNVAVDAFSIAIVGFAITVSLSEMFAKKHGYTVDANQ  43359

Query  384    EMYAIGFCNILPSFFRCFTTSAALTKTLVKESTGCQTQISGIVSALLLLLVLLVIAPLFY  443
              EMYAIGFCNILPSFFRCFTTSAALTKTLVKESTGCQTQISGIVSALLLLLVLLVIAPLFY
Sbjct  43360  EMYAIGFCNILPSFFRCFTTSAALTKTLVKESTGCQTQISGIVSALLLLLVLLVIAPLFY  43539

Query  444    SLQK  447
              SLQK
Sbjct  43540  SLQK  43551
```

Range 5: 52854 to 53171

Score:380 bits(774), Expect:1e-103,
Method:,
Identities:106/106(100%), Positives:106/106(100%), Gaps:0/106(0%)

```
Query  600    EHEAGVTLMENKSLRSVVIDGSSISFLDTAGVNALKEVRKDYAELGVSVVLAQCSTSVLD  659
              EHEAGVTLMENKSLRSVVIDGSSISFLDTAGVNALKEVRKDYAELGVSVVLAQCSTSVLD
Sbjct  52854  EHEAGVTLMENKSLRSVVIDGSSISFLDTAGVNALKEVRKDYAELGVSVVLAQCSTSVLD  53033

Query  660    SLQRGGYYPKAEGDGGEEAVIFYTIEDAVRYVQSLSAANGDQDSKC  705
              SLQRGGYYPKAEGDGGEEAVIFYTIEDAVRYVQSLSAANGDQDSKC
Sbjct  53034  SLQRGGYYPKAEGDGGEEAVIFYTIEDAVRYVQSLSAANGDQDSKC  53171
```

Range 6: 37616 to 37909

Score:362 bits(737), Expect:3e-98,
Method:,
Identities:98/98(100%), Positives:98/98(100%), Gaps:0/98(0%)

```
Query  200    QVLMGIFQVGFVSVYLSDSLLSGFATGASLTILTSQFKYLLGLKIPRAQGWFTLFKTWYS  259
              QVLMGIFQVGFVSVYLSDSLLSGFATGASLTILTSQFKYLLGLKIPRAQGWFTLFKTWYS
Sbjct  37616  QVLMGIFQVGFVSVYLSDSLLSGFATGASLTILTSQFKYLLGLKIPRAQGWFTLFKTWYS  37795

Query  260    VLSNLQNTNVCDLVTSLVCLMILLPTKELNDRFKAKLK  297
              VLSNLQNTNVCDLVTSLVCLMILLPTKELNDRFKAKLK
Sbjct  37796  VLSNLQNTNVCDLVTSLVCLMILLPTKELNDRFKAKLK  37909
```

Range 7: 41348 to 41428

Score:104 bits(209), Expect:2e-20,
Method:,
Identities:27/27(100%), Positives:27/27(100%), Gaps:0/27(0%)

```
Query  298    APIPFELFMVVIATLASHFGNFNIDYG  324
              APIPFELFMVVIATLASHFGNFNIDYG
Sbjct  41348  APIPFELFMVVIATLASHFGNFNIDYG  41428
```

FB Antarctic spiny plunderfish *slc26a2*

Query: unnamed protein product Query ID: lcl|Query_1157913 Length: 707

>Harpagifer antarcticus genome assembly, contig: Scaff10x_910_arrow_ctg1, whole genome shotgun sequence
Sequence ID: CADEHL010001477.1 Length: 241852
Range 1: 11440 to 12048

Score:740 bits(1511), Expect:0.0,

```
Method:,
Identities:201/203(99%), Positives:203/203(100%), Gaps:0/203(0%)

Query  1      MTDADDFCPTAEDGAESDQQHPLVLQRVQEEPKSWQTVASNRLKKNCSCTPKKAKSKILG  60
              MTDADDFCPTAEDGAESDQQHPLVLQRVQEEPKSWQTVASNRLKKNCSCTPKKAKSKILG
Sbjct  11440  MTDADDFCPTAEDGAESDQQHPLVLQRVQEEPKSWQTVASNRLKKNCSCTPKKAKSKILG  11619

Query  61     FVPIVKWLPRYQLKEWILGDVMSGLIVGILLVPQSIAYSLLASQDPIYGLYTSFFASIIY  120
              FVPIVKWLPRYQLKEWILGDVMSGLIVGILLVPQSIAYSLLASQDPIYGLYTSFFASIIY
Sbjct  11620  FVPIVKWLPRYQLKEWILGDVMSGLIVGILLVPQSIAYSLLASQDPIYGLYTSFFASIIY  11799

Query  121    ALLGTSRHISVGIFGVLCLLVGQVVDRELALAGYLPENSILSSNDSSILLGNSSFGMDCD  180
              ALLGTSRHISVGIFGVLCLLVGQVVDRELALAGYLPENSILSSNDSSILLGNSSFGMDCD
Sbjct  11800  ALLGTSRHISVGIFGVLCLLVGQVVDRELALAGYLPENSILSSNDSSILLGNSSFGMDCD  11979

Query  181    RSCYAITVGATVTFTAGVYQVLM  203
              RSCYAITVGATVTFTAGVYQV++
Sbjct  11980  RSCYAITVGATVTFTAGVYQVML  12048
```

Range 2: 25908 to 26369

Score:565 bits(1153), Expect:1e-159,
Method:,
Identities:154/154(100%), Positives:154/154(100%), Gaps:0/154(0%)

```
Query  448    CVLAVIILVNLRGALRKFLDIPSMWRVNRVDTSIWLVTMATSALVNTELGLLVGVLVSAL  507
              CVLAVIILVNLRGALRKFLDIPSMWRVNRVDTSIWLVTMATSALVNTELGLLVGVLVSAL
Sbjct  25908  CVLAVIILVNLRGALRKFLDIPSMWRVNRVDTSIWLVTMATSALVNTELGLLVGVLVSAL  26087

Query  508    CVLARTQQAKVLKLGRASNREYYADVSSYRGLQTHPGVAVFRYDAPIYYANQSLFKKSLY  567
              CVLARTQQAKVLKLGRASNREYYADVSSYRGLQTHPGVAVFRYDAPIYYANQSLFKKSLY
Sbjct  26088  CVLARTQQAKVLKLGRASNREYYADVSSYRGLQTHPGVAVFRYDAPIYYANQSLFKKSLY  26267

Query  568    RSAGLDPLTEKSRVMKFKKKKEKQQEVPEAKPTH  601
              RSAGLDPLTEKSRVMKFKKKKEKQQEVPEAKPTH
Sbjct  26268  RSAGLDPLTEKSRVMKFKKKKEKQQEVPEAKPTH  26369
```

Range 3: 20699 to 21070

Score:453 bits(923), Expect:1e-125,
Method:,
Identities:124/124(100%), Positives:124/124(100%), Gaps:0/124(0%)

```
Query  324    GSGVAGTIPTGFLPPQMPLWSLIPNVAVDAFSIAIVGFAITVSLSEMFAKKHGYTVDANQ  383
              GSGVAGTIPTGFLPPQMPLWSLIPNVAVDAFSIAIVGFAITVSLSEMFAKKHGYTVDANQ
Sbjct  20699  GSGVAGTIPTGFLPPQMPLWSLIPNVAVDAFSIAIVGFAITVSLSEMFAKKHGYTVDANQ  20878

Query  384    EMYAIGFCNILPSFFRCFTTSAALTKTLVKESTGCQTQISGIVSALVLLLVLLVIAPLFY  443
              EMYAIGFCNILPSFFRCFTTSAALTKTLVKESTGCQTQISGIVSALVLLLVLLVIAPLFY
Sbjct  20879  EMYAIGFCNILPSFFRCFTTSAALTKTLVKESTGCQTQISGIVSALVLLLVLLVIAPLFY  21058

Query  444    SLQK  447
              SLQK
Sbjct  21059  SLQK  21070
```

Range 4: 29109 to 29426

Score:382 bits(779), Expect:2e-104,
Method:,
Identities:106/106(100%), Positives:106/106(100%), Gaps:0/106(0%)

```
Query  602    EDEAGVTLMENKSLRSVVIDGSSISFLDTAGVNALKEVRKDYAELGVKVVLAQCNTSVLD  661
              EDEAGVTLMENKSLRSVVIDGSSISFLDTAGVNALKEVRKDYAELGVKVVLAQCNTSVLD
Sbjct  29109  EDEAGVTLMENKSLRSVVIDGSSISFLDTAGVNALKEVRKDYAELGVKVVLAQCNTSVLD  29288

Query  662    SLQRGGYYPKTESDGGEETVIFYTIEDAVRYVQSLSAANGDHDSKC  707
              SLQRGGYYPKTESDGGEETVIFYTIEDAVRYVQSLSAANGDHDSKC
Sbjct  29289  SLQRGGYYPKTESDGGEETVIFYTIEDAVRYVQSLSAANGDHDSKC  29426
```

Range 5: 16727 to 17020

Score:364 bits(741), Expect:8e-99,
Method:,
Identities:98/98(100%), Positives:98/98(100%), Gaps:0/98(0%)

```
Query  200    QVLMGIFQVGFVSVYLSDSLLSGFATGASLTIFTSQFKYLLGLKIPRPQGWFTLFKTWYS  259
              QVLMGIFQVGFVSVYLSDSLLSGFATGASLTIFTSQFKYLLGLKIPRPQGWFTLFKTWYS
Sbjct  16727  QVLMGIFQVGFVSVYLSDSLLSGFATGASLTIFTSQFKYLLGLKIPRPQGWFTLFKTWYS  16906

Query  260    VLSNLQNTNVCDLVTSLVCLMILLPTKELNDRFKAKLK  297
              VLSNLQNTNVCDLVTSLVCLMILLPTKELNDRFKAKLK
Sbjct  16907  VLSNLQNTNVCDLVTSLVCLMILLPTKELNDRFKAKLK  17020
```

Range 6: 18616 to 18696

Score:102 bits(205), Expect:8e-20,
Method:,
Identities:27/27(100%), Positives:27/27(100%), Gaps:0/27(0%)

```
Query  298    APIPFELFVVVIATLASHFGNFNIDYG  324
              APIPFELFVVVIATLASHFGNFNIDYG
Sbjct  18616  APIPFELFVVVIATLASHFGNFNIDYG  18696
```

FC White-fin plunderfish *slc26a2*

Query: hypothetical protein JOQ06_026271 [Pogonophryne albipinna] Query ID: KAJ4921587.1 Length: 707

>Pogonophryne albipinna isolate SGF0006 Scaffold_90, whole genome shotgun sequence
Sequence ID: JAPTMU010000113.1 Length: 605536
Range 1: 35881 to 36489

Score:741 bits(1512), Expect:0.0,
Method:,
Identities:201/203(99%), Positives:203/203(100%), Gaps:0/203(0%)

```
Query  1      MTDADDFCPTAEDGAESDQQHPLILQRVQEEPKSWQTVVSNRLKKNCSCTPKKAKSKILG  60
              MTDADDFCPTAEDGAESDQQHPLILQRVQEEPKSWQTVVSNRLKKNCSCTPKKAKSKILG
Sbjct  35881  MTDADDFCPTAEDGAESDQQHPLILQRVQEEPKSWQTVVSNRLKKNCSCTPKKAKSKILG  36060

Query  61     FVPIVKWLPRYQLKKWILGDVMSGLIVGILLVPQSIAYSLLASQDPIYGLYTSFFASIIY  120
              FVPIVKWLPRYQLKKWILGDVMSGLIVGILLVPQSIAYSLLASQDPIYGLYTSFFASIIY
Sbjct  36061  FVPIVKWLPRYQLKKWILGDVMSGLIVGILLVPQSIAYSLLASQDPIYGLYTSFFASIIY  36240

Query  121    ALLGTSRHISVGIFGVLCLLVGQVVDRELALAGYLPENSILSSNDSSILLGNSSFGMDCD  180
              ALLGTSRHISVGIFGVLCLLVGQVVDRELALAGYLPENSILSSNDSSILLGNSSFGMDCD
Sbjct  36241  ALLGTSRHISVGIFGVLCLLVGQVVDRELALAGYLPENSILSSNDSSILLGNSSFGMDCD  36420

Query  181    RSCYAITVGATVTFTAGVYQVLM  203
              RSCYAITVGATVTFTAGVYQV++
Sbjct  36421  RSCYAITVGATVTFTAGVYQVML  36489
```

Range 2: 64729 to 65190

Score:565 bits(1153), Expect:3e-164,
Method:,
Identities:154/154(100%), Positives:154/154(100%), Gaps:0/154(0%)

```
Query  448    CVLAVIILVNLRGALRKFLDIPSMWRVNRVDTSIWLVTMATSALVNTELGLLVGVLVSAL  507
              CVLAVIILVNLRGALRKFLDIPSMWRVNRVDTSIWLVTMATSALVNTELGLLVGVLVSAL
Sbjct  64729  CVLAVIILVNLRGALRKFLDIPSMWRVNRVDTSIWLVTMATSALVNTELGLLVGVLVSAL  64908

Query  508    CVLARTQQAKVLKLGRASNREYYEDVSSYRGLQTLPGVAVFRYDAPIYYANQSLFKKSLY  567
              CVLARTQQAKVLKLGRASNREYYEDVSSYRGLQTLPGVAVFRYDAPIYYANQSLFKKSLY
Sbjct  64909  CVLARTQQAKVLKLGRASNREYYEDVSSYRGLQTLPGVAVFRYDAPIYYANQSLFKKSLY  65088

Query  568    KSAGLDPLREKSRVMKFKKKKEKQQEVPDAKPTH  601
              KSAGLDPLREKSRVMKFKKKKEKQQEVPDAKPTH
Sbjct  65089  KSAGLDPLREKSRVMKFKKKKEKQQEVPDAKPTH  65190
```

Range 3: 58247 to 58618

Score:454 bits(925), Expect:1e-130,
Method:,
Identities:124/124(100%), Positives:124/124(100%), Gaps:0/124(0%)

```
Query  324    GSGVAGTIPTGFLPPQMPLWSLIPNVAVDAFSIAIVGFAITVSLSEMFAKKHGYTVDANQ  383
              GSGVAGTIPTGFLPPQMPLWSLIPNVAVDAFSIAIVGFAITVSLSEMFAKKHGYTVDANQ
Sbjct  58247  GSGVAGTIPTGFLPPQMPLWSLIPNVAVDAFSIAIVGFAITVSLSEMFAKKHGYTVDANQ  58426

Query  384    EMYAIGFCNILPSFFRCFTTSAALTKTLVKESTGCQTQISGIITALVLLLVLLVIAPLFY  443
              EMYAIGFCNILPSFFRCFTTSAALTKTLVKESTGCQTQISGIITALVLLLVLLVIAPLFY
Sbjct  58427  EMYAIGFCNILPSFFRCFTTSAALTKTLVKESTGCQTQISGIITALVLLLVLLVIAPLFY  58606

Query  444    SLQK  447
              SLQK
Sbjct  58607  SLQK  58618
```

Range 4: 67136 to 67453

Score:383 bits(780), Expect:3e-109,
Method:,
Identities:106/106(100%), Positives:106/106(100%), Gaps:0/106(0%)

```
Query  602    EDEAGVTLMENKSLRSVVIDGSSISFLDTAGVNALKEVRKDYAELGVKVVLAQCNTSVLD  661
              EDEAGVTLMENKSLRSVVIDGSSISFLDTAGVNALKEVRKDYAELGVKVVLAQCNTSVLD
```

```
Sbjct  67136  EDEAGVTLMENKSLRSVVIDGSSISFLDTAGVNALKEVRKDYAELGVKVVLAQCNTSVLD  67315

Query  662    SLHRGGYYPKTESDGAEETVIFYTIEDAVRYVQSLSAANGDHDSKC  707
              SLHRGGYYPKTESDGAEETVIFYTIEDAVRYVQSLSAANGDHDSKC
Sbjct  67316  SLHRGGYYPKTESDGAEETVIFYTIEDAVRYVQSLSAANGDHDSKC  67453
```

Range 5: 50458 to 50751

Score:364 bits(741), Expect:1e-103,
Method:,
Identities:98/98(100%), Positives:98/98(100%), Gaps:0/98(0%)

```
Query  200    QVLMGIFQVGFVSIYLSDSLLSGFATGASLTILTSQFKYLLGLKIPRPQGWFTLFQTWYS  259
              QVLMGIFQVGFVSIYLSDSLLSGFATGASLTILTSQFKYLLGLKIPRPQGWFTLFQTWYS
Sbjct  50458  QVLMGIFQVGFVSIYLSDSLLSGFATGASLTILTSQFKYLLGLKIPRPQGWFTLFQTWYS  50637

Query  260    VLSNLQNTNVCDLVTSLVCLMILLPTKELNDRFKAKLK  297
              VLSNLQNTNVCDLVTSLVCLMILLPTKELNDRFKAKLK
Sbjct  50638  VLSNLQNTNVCDLVTSLVCLMILLPTKELNDRFKAKLK  50751
```

Range 6: 52545 to 52625

Score:102 bits(205), Expect:1e-24,
Method:,
Identities:27/27(100%), Positives:27/27(100%), Gaps:0/27(0%)

```
Query  298    APIPFELFVVVIATLASHFGNFNIDYG  324
              APIPFELFVVVIATLASHFGNFNIDYG
Sbjct  52545  APIPFELFVVVIATLASHFGNFNIDYG  52625
```

FD Inshore hagfish *slc26a2-like*

Query: unnamed protein product Query ID: lcl|Query_2457891 Length: 702

>Eptatretus burgeri genome assembly, contig: cluster12, whole genome shotgun sequence
Sequence ID: FYBX03000012.1 Length: 132019938
Range 1: 71106924 to 71107844

Score:1135 bits(2317), Expect:0.0,
Method:,
Identities:307/307(100%), Positives:307/307(100%), Gaps:0/307(0%)

```
Query  396       QEVIAIGCCNLIPAFFYSFTTSAALAKTLVKEATGCQTQVSGLITAMCLLLVLLVIAPLF  455
                 QEVIAIGCCNLIPAFFYSFTTSAALAKTLVKEATGCQTQVSGLITAMCLLLVLLVIAPLF
Sbjct  71107844  QEVIAIGCCNLIPAFFYSFTTSAALAKTLVKEATGCQTQVSGLITAMCLLLVLLVIAPLF  71107665

Query  456       YSLQKCVLAIVTVVNLRSAFRKFLDIPKMWQENCIDTIIWFITMLTSALISPELGLLIGI  515
                 YSLQKCVLAIVTVVNLRSAFRKFLDIPKMWQENCIDTIIWFITMLTSALISPELGLLIGI
Sbjct  71107664  YSLQKCVLAIVTVVNLRSAFRKFLDIPKMWQENCIDTIIWFITMLTSALISPELGLLIGI  71107485

Query  516       CISLLAFIIRTQWPRAVLLGRVEGTTDCYADLEAYTNVSEVKGVRIFSFSAPLYYANKDN  575
                 CISLLAFIIRTQWPRAVLLGRVEGTTDCYADLEAYTNVSEVKGVRIFSFSAPLYYANKDN
Sbjct  71107484  CISLLAFIIRTQWPRAVLLGRVEGTTDCYADLEAYTNVSEVKGVRIFSFSAPLYYANKDN  71107305

Query  576       FSTKLHSKTAVNPELVVAWRKNESKESKQKDEQSDTNGVIIVPDPFDFHTLVLDFSSVNF  635
                 FSTKLHSKTAVNPELVVAWRKNESKESKQKDEQSDTNGVIIVPDPFDFHTLVLDFSSVNF
Sbjct  71107304  FSTKLHSKTAVNPELVVAWRKNESKESKQKDEQSDTNGVIIVPDPFDFHTLVLDFSSVNF  71107125

Query  636       LDMVGTGVVKSTCSDYQTIGLRVLIAGCNPSVLDSLSRARFIDGNDEEVLFFSVHEAVEH  695
                 LDMVGTGVVKSTCSDYQTIGLRVLIAGCNPSVLDSLSRARFIDGNDEEVLFFSVHEAVEH
Sbjct  71107124  LDMVGTGVVKSTCSDYQTIGLRVLIAGCNPSVLDSLSRARFIDGNDEEVLFFSVHEAVEH  71106945

Query  696       ALARHSG  702
                 ALARHSG
Sbjct  71106944  ALARHSG  71106924
```

Range 2: 71108043 to 71108591

Score:685 bits(1397), Expect:0.0,
Method:,
Identities:183/183(100%), Positives:183/183(100%), Gaps:0/183(0%)

```
Query  214       ITMGIFQLGFIAIYLSEPMLSGFVTGASFVILTSQVKYMLGLHIPRTGGPGSLLLTWYYI  273
                 ITMGIFQLGFIAIYLSEPMLSGFVTGASFVILTSQVKYMLGLHIPRTGGPGSLLLTWYYI
Sbjct  71108591  ITMGIFQLGFIAIYLSEPMLSGFVTGASFVILTSQVKYMLGLHIPRTGGPGSLLLTWYYI  71108412

Query  274       LKNIGHTNICDLITSAICLFIMVPIKEVNERYKKKMKAPFPIDLIVIIFATLVSHFGKLE  333
                 LKNIGHTNICDLITSAICLFIMVPIKEVNERYKKKMKAPFPIDLIVIIFATLVSHFGKLE
Sbjct  71108411  LKNIGHTNICDLITSAICLFIMVPIKEVNERYKKKMKAPFPIDLIVIIFATLVSHFGKLE  71108232

Query  334       ENFDSSIAGEIPIGFKPPRAPSWTLVPSVLVDGIPIAIIGFAITVSLSEMFAKKHGYTIR  393
                 ENFDSSIAGEIPIGFKPPRAPSWTLVPSVLVDGIPIAIIGFAITVSLSEMFAKKHGYTIR
```

```
Sbjct  71108231  ENFDSSIAGEIPIGFKPPRAPSWTLVPSVLVDGIPIAIIGFAITVSLSEMFAKKHGYTIR  71108052

Query  394       PNQ  396
                 PNQ
Sbjct  71108051  PNQ  71108043
```

Range 3: 71120881 to 71121102

Score:263 bits(535), Expect:9e-73,
Method:,
Identities:73/74(99%), Positives:73/74(98%), Gaps:1/74(1%)

```
Query  82        YKIKEMLLGD-MSGLIVGVLLVPQSIAYSLLAGQDPIYGLYSSFFACIIYFILGSSRHIS  140
                 YKIKEMLLGD MSGLIVGVLLVPQSIAYSLLAGQDPIYGLYSSFFACIIYFILGSSRHIS
Sbjct  71121102  YKIKEMLLGDVMSGLIVGVLLVPQSIAYSLLAGQDPIYGLYSSFFACIIYFILGSSRHIS  71120923

Query  141       VSIFGVLCLMIGQV  154
                 VSIFGVLCLMIGQV
Sbjct  71120922  VSIFGVLCLMIGQV  71120881
```

Range 4: 71132862 to 71133047

Score:238 bits(483), Expect:4e-65,
Method:,
Identities:62/62(100%), Positives:62/62(100%), Gaps:0/62(0%)

```
Query  21        AELSDRNPRCLRHTLLHRRPDPPWNLQASLTKKLKNECVCTPRAAKNGLFNLFPVLQWLP  80
                 AELSDRNPRCLRHTLLHRRPDPPWNLQASLTKKLKNECVCTPRAAKNGLFNLFPVLQWLP
Sbjct  71133047  AELSDRNPRCLRHTLLHRRPDPPWNLQASLTKKLKNECVCTPRAAKNGLFNLFPVLQWLP  71132868

Query  81        KY  82
                 KY
Sbjct  71132867  KY  71132862
```

Range 5: 71109137 to 71109331

Score:228 bits(463), Expect:4e-62,
Method:,
Identities:63/65(97%), Positives:64/65(98%), Gaps:0/65(0%)

```
Query  153       QVVDRELIVAGFTSADDSQAHEKLNYSESHENVTFGGVVYTRSDFAILVGTTVTFVAGVY  212
                 QVVDRELIVAGFTSADDSQAHEKLNYSESHENVTFGGVVYTRSDFAILVGTTVTFVAGVY
Sbjct  71109331  QVVDRELIVAGFTSADDSQAHEKLNYSESHENVTFGGVVYTRSDFAILVGTTVTFVAGVY  71109152

Query  213       QITMG  217
                 Q+ MG
Sbjct  71109151  QVGMG  71109137
```

Range 6: 71191497 to 71191556

Score:77.2 bits(154), Expect:1e-16,
Method:,
Identities:20/20(100%), Positives:20/20(100%), Gaps:0/20(0%)

```
Query  1         MAEENGLISIPLLNRNIDRP  20
                 MAEENGLISIPLLNRNIDRP
Sbjct  71191556  MAEENGLISIPLLNRNIDRP  71191497
```

FE Sea lamprey *slc26a2-like*

Query: sulfate transporter-like isoform X1 [Petromyzon marinus] Query ID: XP_032806611.1 Length: 757

>Petromyzon marinus isolate kPetMar1 chromosome 9, kPetMar1.pri, whole genome shotgun sequence
Sequence ID: NC_046077.1 Length: 16569030
Range 1: 11062834 to 11063856

Score:1265 bits(2584), Expect:0.0,
Method:,
Identities:341/341(100%), Positives:341/341(100%), Gaps:0/341(0%)

```
Query  417       QEMIAIGFCNIVPAFFYSFTTSAALAKTLVKEATGCQTQMSAVVTAGVLLLVLLLIAPLF  476
                 QEMIAIGFCNIVPAFFYSFTTSAALAKTLVKEATGCQTQMSAVVTAGVLLLVLLLIAPLF
Sbjct  11062834  QEMIAIGFCNIVPAFFYSFTTSAALAKTLVKEATGCQTQMSAVVTAGVLLLVLLLIAPLF  11063013

Query  477       YSLQKCVLGVITIVNLRGAFRKFLDIPRMWQESRVDTSIWFVTMLASALISTEIGLLVGV  536
                 YSLQKCVLGVITIVNLRGAFRKFLDIPRMWQESRVDTSIWFVTMLASALISTEIGLLVGV
Sbjct  11063014  YSLQKCVLGVITIVNLRGAFRKFLDIPRMWQESRVDTSIWFVTMLASALISTEIGLLVGV  11063193

Query  537       CVSLLVFIVRTQRPRAALYGCVHDTNNFYADLEAYSNVAEVPGVRVFHFDAPLYYANKEY  596
```

```
                CVSLLVFIVRTQRPRAALYGCVHDTNNFYADLEAYSNVAEVPGVRVFHFDAPLYYANKEY
Sbjct  11063194  CVSLLVFIVRTQRPRAALYGCVHDTNNFYADLEAYSNVAEVPGVRVFHFDAPLYYANKEY  11063373

Query  597       FKSQLFLKTRVNPALEVAAQKKRQEKLQKRKKLSGSDNGEIVNKQAMLEFNKLLPTFDFH  656
                FKSQLFLKTRVNPALEVAAQKKRQEKLQKRKKLSGSDNGEIVNKQAMLEFNKLLPTFDFH
Sbjct  11063374  FKSQLFLKTRVNPALEVAAQKKRQEKLQKRKKLSGSDNGEIVNKQAMLEFNKLLPTFDFH  11063553

Query  657       TIVIDCSAMQFMDSVGVGAVKSIMKDYQSIGIRVILACCNPTVMDSLHRGKCISTIEDNL  716
                TIVIDCSAMQFMDSVGVGAVKSIMKDYQSIGIRVILACCNPTVMDSLHRGKCISTIEDNL
Sbjct  11063554  TIVIDCSAMQFMDSVGVGAVKSIMKDYQSIGIRVILACCNPTVMDSLHRGKCISTIEDNL  11063733

Query  717       TFCLIHDAVLYAEAEERKSHHDNNVAIDGLEGNREVGASEV  757
                TFCLIHDAVLYAEAEERKSHHDNNVAIDGLEGNREVGASEV
Sbjct  11063734  TFCLIHDAVLYAEAEERKSHHDNNVAIDGLEGNREVGASEV  11063856
```

Range 2: 11058276 to 11058707

Score:544 bits(1110), Expect:0.0,
Method:,
Identities:144/144(100%), Positives:144/144(100%), Gaps:0/144(0%)

```
Query  24        EMVAVGNGCTESPRGDGGTCVRIRLQVREQAPFNPKVALAKTVANNCCCSPHELKSFFLN  83
                EMVAVGNGCTESPRGDGGTCVRIRLQVREQAPFNPKVALAKTVANNCCCSPHELKSFFLN
Sbjct  11058276  EMVAVGNGCTESPRGDGGTCVRIRLQVREQAPFNPKVALAKTVANNCCCSPHELKSFFLN  11058455

Query  84        LFPVLRWLPKYKVREWILGDFMSGLIVGILLVPQCIAYSLLAGQDPIYGLYTSFFACIIY  143
                LFPVLRWLPKYKVREWILGDFMSGLIVGILLVPQCIAYSLLAGQDPIYGLYTSFFACIIY
Sbjct  11058456  LFPVLRWLPKYKVREWILGDFMSGLIVGILLVPQCIAYSLLAGQDPIYGLYTSFFACIIY  11058635

Query  144       FLMGTSKHISVGIFGVLCLMIGQV  167
                FLMGTSKHISVGIFGVLCLMIGQV
Sbjct  11058636  FLMGTSKHISVGIFGVLCLMIGQV  11058707
```

Range 3: 11061051 to 11061416

Score:449 bits(916), Expect:4e-145,
Method:,
Identities:122/122(100%), Positives:122/122(100%), Gaps:0/122(0%)

```
Query  234       QVVMGIFQMGFVSIYLSEPMLSGFVTGSSLTVLTSQMKYLLGIHIPRASGAGALLKTWYH  293
                QVVMGIFQMGFVSIYLSEPMLSGFVTGSSLTVLTSQMKYLLGIHIPRASGAGALLKTWYH
Sbjct  11061051  QVVMGIFQMGFVSIYLSEPMLSGFVTGSSLTVLTSQMKYLLGIHIPRASGAGALLKTWYH  11061230

Query  294       IFSQISNTNVCDVVTSIICILVLVPAKELNEHFKARLKAPFPIELVVVVAATLASHFGGF  353
                IFSQISNTNVCDVVTSIICILVLVPAKELNEHFKARLKAPFPIELVVVVAATLASHFGGF
Sbjct  11061231  IFSQISNTNVCDVVTSIICILVLVPAKELNEHFKARLKAPFPIELVVVVAATLASHFGGF  11061410

Query  354       KE  355
                KE
Sbjct  11061411  KE  11061416
```

Range 4: 11059947 to 11060156

Score:260 bits(529), Expect:6e-75,
Method:,
Identities:70/70(100%), Positives:70/70(100%), Gaps:0/70(0%)

```
Query  166       QVVDRELQVAGFAEEGIKGHDHVVNNSMNFSNSDEENGFTVMIGGLPCGRSCFAIAVGTT  225
                QVVDRELQVAGFAEEGIKGHDHVVNNSMNFSNSDEENGFTVMIGGLPCGRSCFAIAVGTT
Sbjct  11059947  QVVDRELQVAGFAEEGIKGHDHVVNNSMNFSNSDEENGFTVMIGGLPCGRSCFAIAVGTT  11060126

Query  226       VTFMAGVYQV  235
                VTFMAGVYQV
Sbjct  11060127  VTFMAGVYQV  11060156
```

Range 5: 11062139 to 11062321

Score:225 bits(457), Expect:4e-63,
Method:,
Identities:61/61(100%), Positives:61/61(100%), Gaps:0/61(0%)

```
Query  357       YSSSIAGHIPTGFLPPRAPEWTLIPRIAADAVPIAIIGFAITVSLSEMFAKKHGYTVRAN  416
                YSSSIAGHIPTGFLPPRAPEWTLIPRIAADAVPIAIIGFAITVSLSEMFAKKHGYTVRAN
Sbjct  11062139  YSSSIAGHIPTGFLPPRAPEWTLIPRIAADAVPIAIIGFAITVSLSEMFAKKHGYTVRAN  11062318

Query  417       Q  417
                Q
Sbjct  11062319  Q  11062321
```

Range 6: 11041128 to 11041196

Score:86.0 bits(172), Expect:6e-20,
Method:,

```
Identities:23/23(100%), Positives:23/23(100%), Gaps:0/23(0%)

Query  1         MGEQENEDSAVNKHLLGPTAEET  23
                 MGEQENEDSAVNKHLLGPTAEET
Sbjct  11041128  MGEQENEDSAVNKHLLGPTAEET  11041196
```

FF Inshore hagfish *slc26a12-like*

```
Query: unnamed protein product Query ID: lcl|Query_2100377 Length: 688


>Eptatretus burgeri genome assembly, contig: cluster12, whole genome shotgun sequence
Sequence ID: FYBX03000012.1 Length: 132019938
Range 1: 71373479 to 71374156

Score:852 bits(1739), Expect:0.0,
Method:,
Identities:226/226(100%), Positives:226/226(100%), Gaps:0/226(0%)

Query  1         MEENNEFSLKNSNKSSENTFSDSFDVEDGISHVCIQLEKQDQPQVTFLERVKMTAEKHLT  60
                 MEENNEFSLKNSNKSSENTFSDSFDVEDGISHVCIQLEKQDQPQVTFLERVKMTAEKHLT
Sbjct  71374156  MEENNEFSLKNSNKSSENTFSDSFDVEDGISHVCIQLEKQDQPQVTFLERVKMTAEKHLT  71373977

Query  61        CSPSCFKKVIFDIFPVLQWLPNYKFREYIIGDIMSGIVVGIISVPQSIAYSLLASVDPIY  120
                 CSPSCFKKVIFDIFPVLQWLPNYKFREYIIGDIMSGIVVGIISVPQSIAYSLLASVDPIY
Sbjct  71373976  CSPSCFKKVIFDIFPVLQWLPNYKFREYIIGDIMSGIVVGIISVPQSIAYSLLASVDPIY  71373797

Query  121       GLYASFFPCIIYFLFGTSRHTSIGIFAVLCLMIGQVVDQELQEAGYDLSVNTSNIHRYSN  180
                 GLYASFFPCIIYFLFGTSRHTSIGIFAVLCLMIGQVVDQELQEAGYDLSVNTSNIHRYSN
Sbjct  71373796  GLYASFFPCIIYFLFGTSRHTSIGIFAVLCLMIGQVVDQELQEAGYDLSVNTSNIHRYSN  71373617

Query  181       TEKLEYEMVSNLSTPFPTLVSNNCDRSCYAIRIAANITFLAGVYQV  226
                 TEKLEYEMVSNLSTPFPTLVSNNCDRSCYAIRIAANITFLAGVYQV
Sbjct  71373616  TEKLEYEMVSNLSTPFPTLVSNNCDRSCYAIRIAANITFLAGVYQV  71373479




Range 2: 71371135 to 71371764

Score:788 bits(1608), Expect:0.0,
Method:,
Identities:210/210(100%), Positives:210/210(100%), Gaps:0/210(0%)

Query  408       QEMLAIGCCNIIPSFFYSFATCATVTKTLLKDATGCMTQVSSLITAAVILLVLMVIAPLF  467
                 QEMLAIGCCNIIPSFFYSFATCATVTKTLLKDATGCMTQVSSLITAAVILLVLMVIAPLF
Sbjct  71371764  QEMLAIGCCNIIPSFFYSFATCATVTKTLLKDATGCMTQVSSLITAAVILLVLMVIAPLF  71371585

Query  468       HSLQNCVLATIIIVNLRGAFQKFFDTPRMWRVSHADALVWWVTMLASALLSTEVGLLVGI  527
                 HSLQNCVLATIIIVNLRGAFQKFFDTPRMWRVSHADALVWWVTMLASALLSTEVGLLVGI
Sbjct  71371584  HSLQNCVLATIIIVNLRGAFQKFFDTPRMWRVSHADALVWWVTMLASALLSTEVGLLVGI  71371405

Query  528       CFAAVCILVRMQRPSIIQLGRVPDTKQYADLNLYRGLLCVSDVVIFRFNAPLYYVNKNYF  587
                 CFAAVCILVRMQRPSIIQLGRVPDTKQYADLNLYRGLLCVSDVVIFRFNAPLYYVNKNYF
Sbjct  71371404  CFAAVCILVRMQRPSIIQLGRVPDTKQYADLNLYRGLLCVSDVVIFRFNAPLYYVNKNYF  71371225

Query  588       QAAIFCKSGLNPTLKVKKNAKDKPEETENE  617
                 QAAIFCKSGLNPTLKVKKNAKDKPEETENE
Sbjct  71371224  QAAIFCKSGLNPTLKVKKNAKDKPEETENE  71371135




Range 3: 71372412 to 71372963

Score:686 bits(1399), Expect:0.0,
Method:,
Identities:184/184(100%), Positives:184/184(100%), Gaps:0/184(0%)

Query  225       QVVMSILQLGFISVYLSEPLLSGFVTGSSITILTSQAKYLLGLPIPRYKGIGALVMNWVS  284
                 QVVMSILQLGFISVYLSEPLLSGFVTGSSITILTSQAKYLLGLPIPRYKGIGALVMNWVS
Sbjct  71372963  QVVMSILQLGFISVYLSEPLLSGFVTGSSITILTSQAKYLLGLPIPRYKGIGALVMNWVS  71372784

Query  285       IFKMIPNTNICDLVTSIICFLVMVPIKEMNIRYKHKMKTPFPIELLVVIVATLISHYVNL  344
                 IFKMIPNTNICDLVTSIICFLVMVPIKEMNIRYKHKMKTPFPIELLVVIVATLISHYVNL
Sbjct  71372783  IFKMIPNTNICDLVTSIICFLVMVPIKEMNIRYKHKMKTPFPIELLVVIVATLISHYVNL  71372604

Query  345       QERYGSNITGTISTGFQVPRLPEWNLVQSVAADTVSIAIVSFVFTISLSEIFAKKHGYVV  404
                 QERYGSNITGTISTGFQVPRLPEWNLVQSVAADTVSIAIVSFVFTISLSEIFAKKHGYVV
Sbjct  71372603  QERYGSNITGTISTGFQVPRLPEWNLVQSVAADTVSIAIVSFVFTISLSEIFAKKHGYVV  71372424

Query  405       RANQ  408
                 RANQ
Sbjct  71372423  RANQ  71372412




Range 4: 71362196 to 71362408

Score:265 bits(539), Expect:3e-74,
Method:,
```

```
Identities:71/71(100%), Positives:71/71(100%), Gaps:0/71(0%)

Query  618       DNGFSKCNTRKIGALVVDCSAMHFVDSPGADVLQELHQAYGRIGVLFLLANCSPTILETL  677
                 DNGFSKCNTRKIGALVVDCSAMHFVDSPGADVLQELHQAYGRIGVLFLLANCSPTILETL
Sbjct  71362408  DNGFSKCNTRKIGALVVDCSAMHFVDSPGADVLQELHQAYGRIGVLFLLANCSPTILETL  71362229

Query  678       QQSWILQAICK  688
                 QQSWILQAICK
Sbjct  71362228  QQSWILQAICK  71362196
```

FG Chicken *slc26a12*

```
Query: sulfate transporter-like isoform X1 [Gallus gallus] Query ID: XP_046756474.1 Length: 735


>Gallus gallus isolate bGalGal1 chromosome 13, bGalGal1.mat.broiler.GRCg7b, whole genome shotgun sequence
Sequence ID: NC_052544.1 Length: 17905061
Range 1: 7946134 to 7947729

Score:1960 bits(4005), Expect:0.0,
Method:,
Identities:531/532(99%), Positives:532/532(100%), Gaps:0/532(0%)

Query  204      YQILLGVLQLGFLSVYLSEPLLSGFVAGSSLTIITSQMKYLLGLNIPRHEGVGSFILTWV  263
                +QILLGVLQLGFLSVYLSEPLLSGFVAGSSLTIITSQMKYLLGLNIPRHEGVGSFILTWV
Sbjct  7947729  FQILLGVLQLGFLSVYLSEPLLSGFVAGSSLTIITSQMKYLLGLNIPRHEGVGSFILTWV  7947550

Query  264      DLFRYIPNTNICDLVTSLVALAIIIPVKMINDRYKDKMKAPVPIELLVVIIATVVSYYFK  323
                DLFRYIPNTNICDLVTSLVALAIIIPVKMINDRYKDKMKAPVPIELLVVIIATVVSYYFK
Sbjct  7947549  DLFRYIPNTNICDLVTSLVALAIIIPVKMINDRYKDKMKAPVPIELLVVIIATVVSYYFK  7947370

Query  324      FEERYKSSVCGAIPTGFRKPTLPDTKLFSSLAVDALPIAIIGFAMTVSLAEIFGKKHGYA  383
                FEERYKSSVCGAIPTGFRKPTLPDTKLFSSLAVDALPIAIIGFAMTVSLAEIFGKKHGYA
Sbjct  7947369  FEERYKSSVCGAIPTGFRKPTLPDTKLFSSLAVDALPIAIIGFAMTVSLAEIFGKKHGYA  7947190

Query  384      VRANQEMIAIGMCNLVPSFFYCFASSAALTKTLLKESTGTQTQLSSLVTSLVLLLVLLWI  443
                VRANQEMIAIGMCNLVPSFFYCFASSAALTKTLLKESTGTQTQLSSLVTSLVLLLVLLWI
Sbjct  7947189  VRANQEMIAIGMCNLVPSFFYCFASSAALTKTLLKESTGTQTQLSSLVTSLVLLLVLLWI  7947010

Query  444      APLFYSLQTAILGVVTIVNLRGGLRTFCETPRMWQLSKLDTAVWWTTMLASTLITTEIGL  503
                APLFYSLQTAILGVVTIVNLRGGLRTFCETPRMWQLSKLDTAVWWTTMLASTLITTEIGL
Sbjct  7947009  APLFYSLQTAILGVVTIVNLRGGLRTFCETPRMWQLSKLDTAVWWTTMLASTLITTEIGL  7946830

Query  504      LVGVCFALLCIIFRTQRPRAALLGKVSNTEIYEDQSAYKQLSSIANIKIFRFGSSLYYAN  563
                LVGVCFALLCIIFRTQRPRAALLGKVSNTEIYEDQSAYKQLSSIANIKIFRFGSSLYYAN
Sbjct  7946829  LVGVCFALLCIIFRTQRPRAALLGKVSNTEIYEDQSAYKQLSSIANIKIFRFGSSLYYAN  7946650

Query  564      KDYFKTALYQKTGVNPILLAARHQRAVTQAKADTGNRKSVFNSVFGCLKPSKKHMGKSPT  623
                KDYFKTALYQKTGVNPILLAARHQRAVTQAKADTGNRKSVFNSVFGCLKPSKKHMGKSPT
Sbjct  7946649  KDYFKTALYQKTGVNPILLAARHQRAVTQAKADTGNRKSVFNSVFGCLKPSKKHMGKSPT  7946470

Query  624      DVCLPSLDMHTLILDCGAMQFIDTTGLSVLKETHRDFQELGVQLLLANCNPLLRRRLRDG  683
                DVCLPSLDMHTLILDCGAMQFIDTTGLSVLKETHRDFQELGVQLLLANCNPLLRRRLRDG
Sbjct  7946469  DVCLPSLDMHTLILDCGAMQFIDTTGLSVLKETHRDFQELGVQLLLANCNPLLRRRLRDG  7946290

Query  684      GWAAGAHGGQLAFHSVHHAVQFAQQWHREQQESKERRDADPDPEDMSVQASL  735
                GWAAGAHGGQLAFHSVHHAVQFAQQWHREQQESKERRDADPDPEDMSVQASL
Sbjct  7946289  GWAAGAHGGQLAFHSVHHAVQFAQQWHREQQESKERRDADPDPEDMSVQASL  7946134



Range 2: 7948541 to 7949158

Score:762 bits(1556), Expect:0.0,
Method:,
Identities:205/206(99%), Positives:206/206(100%), Gaps:0/206(0%)

Query  1        MEDKSSQTSGLSKDVQSSLTPEEPPVDFMHIKLEENEPIDVRTKDFILKKAREVCKCNHQ  60
                MEDKSSQTSGLSKDVQSSLTPEEPPVDFMHIKLEENEPIDVRTKDFILKKAREVCKCNHQ
Sbjct  7949158  MEDKSSQTSGLSKDVQSSLTPEEPPVDFMHIKLEENEPIDVRTKDFILKKAREVCKCNHQ  7948979

Query  61       TIITFFCQLFPVLDWLPRYNIKTQLLGDIISGLLVGIVAIPQSISYSLLANQDPIYGIYT  120
                TIITFFCQLFPVLDWLPRYNIKTQLLGDIISGLLVGIVAIPQSISYSLLANQDPIYGIYT
Sbjct  7948978  TIITFFCQLFPVLDWLPRYNIKTQLLGDIISGLLVGIVAIPQSISYSLLANQDPIYGIYT  7948799

Query  121      NFFCSIIYVAMATSRHNSVGSFGVLCLMIGQSVNRHLQLAGYDDNTDSLLVGNATSSGNG  180
                NFFCSIIYVAMATSRHNSVGSFGVLCLMIGQSVNRHLQLAGYDDNTDSLLVGNATSSGNG
Sbjct  7948798  NFFCSIIYVAMATSRHNSVGSFGVLCLMIGQSVNRHLQLAGYDDNTDSLLVGNATSSGNG  7948619

Query  181      TGTCDRSCYAITVALSLSFLVGLYQI  206
                TGTCDRSCYAITVALSLSFLVGLYQ+
Sbjct  7948618  TGTCDRSCYAITVALSLSFLVGLYQV  7948541
```

FH American alligator *slc26a12*

```
Query: sulfate transporter [Alligator mississippiensis] Query ID: XP_059589402.1 Length: 738
```

>Alligator mississippiensis isolate rAllMis1 chromosome 9, rAllMis1, whole genome shotgun sequence
Sequence ID: NC_081832.1 Length: 79974352
Range 1: 60129598 to 60131199

Score:1959 bits(4002), Expect:0.0,
Method:,
Identities:533/534(99%), Positives:534/534(100%), Gaps:0/534(0%)

```
Query  205       YQILLGVFQLGFIAVYLSEPLLSGFVTGSSLTILTSQMKYLLGLKIPRHEGIGSLILTWV  264
                 +QILLGVFQLGFIAVYLSEPLLSGFVTGSSLTILTSQMKYLLGLKIPRHEGIGSLILTWV
Sbjct  60131199  FQILLGVFQLGFIAVYLSEPLLSGFVTGSSLTILTSQMKYLLGLKIPRHEGIGSLILTWV  60131020

Query  265       DIFRYIRITNICDLVTSLIALAVIVPIKEINARYKDKMKAPFPIELVVIVATLLSYYLN  324
                 DIFRYIRITNICDLVTSLIALAVIVPIKEINARYKDKMKAPFPIELVVIVATLLSYYLN
Sbjct  60131019  DIFRYIRITNICDLVTSLIALAVIVPIKEINARYKDKMKAPFPIELVVIVATLLSYYLN  60130840

Query  325       FEGQYNSAVCGTIPTGFRKPTVPDVHLFSSLAIDALPIAIIGFAMTVSLAEIFAKKHSYT  384
                 FEGQYNSAVCGTIPTGFRKPTVPDVHLFSSLAIDALPIAIIGFAMTVSLAEIFAKKHSYT
Sbjct  60130839  FEGQYNSAVCGTIPTGFRKPTVPDVHLFSSLAIDALPIAIIGFAMTVSLAEIFAKKHSYT  60130660

Query  385       VRANQEMIAIGMCNLVPSFFYSFASSAALAKTLLKESTGSHTQLSGLVTSAVLLLVLLWI  444
                 VRANQEMIAIGMCNLVPSFFYSFASSAALAKTLLKESTGSHTQLSGLVTSAVLLLVLLWI
Sbjct  60130659  VRANQEMIAIGMCNLVPSFFYSFASSAALAKTLLKESTGSHTQLSGLVTSAVLLLVLLWI  60130480

Query  445       APLFYSLQTCILGVVTIVNLRGGLLKFVDTHKMWQLSKVDSVVWWVTMLASSLLSTEIGL  504
                 APLFYSLQTCILGVVTIVNLRGGLLKFVDTHKMWQLSKVDSVVWWVTMLASSLLSTEIGL
Sbjct  60130479  APLFYSLQTCILGVVTIVNLRGGLLKFVDTHKMWQLSKVDSVVWWVTMLASSLLSTEIGL  60130300

Query  505       LVGVCFALLCIIFRTQRPRATLLGKVSNTEIYEDLFTYKKLSSIANIKIFRFESSLYYAN  564
                 LVGVCFALLCIIFRTQRPRATLLGKVSNTEIYEDLFTYKKLSSIANIKIFRFESSLYYAN
Sbjct  60130299  LVGVCFALLCIIFRTQRPRATLLGKVSNTEIYEDLFTYKKLSSIANIKIFRFESSLYYAN  60130120

Query  565       KDYFKSALYQKTGVNPAVVSAKQQKVEAKAKATMTNSNNCFSARFSCLKSAKKGVQKTEV  624
                 KDYFKSALYQKTGVNPAVVSAKQQKVEAKAKATMTNSNNCFSARFSCLKSAKKGVQKTEV
Sbjct  60130119  KDYFKSALYQKTGVNPAVVSAKQQKVEAKAKATMTNSNNCFSARFSCLKSAKKGVQKTEV  60129940

Query  625       PVPSIGMHTLIIDCGAMQFIDTVGLSALKETRQDYKEVGIQVLLANCNPSIRRLLQDGGW  684
                 PVPSIGMHTLIIDCGAMQFIDTVGLSALKETRQDYKEVGIQVLLANCNPSIRRLLQDGGW
Sbjct  60129939  PVPSIGMHTLIIDCGAMQFIDTVGLSALKETRQDYKEVGIQVLLANCNPSIRRLLQDGGW  60129760

Query  685       ASKTDDGEHLAFHSVHDAVQFAERHFEDSKAKEAAFLDSEVQDILEDLNFPADL  738
                 ASKTDDGEHLAFHSVHDAVQFAERHFEDSKAKEAAFLDSEVQDILEDLNFPADL
Sbjct  60129759  ASKTDDGEHLAFHSVHDAVQFAERHFEDSKAKEAAFLDSEVQDILEDLNFPADL  60129598
```

Range 2: 60132101 to 60132721

Score:770 bits(1571), Expect:0.0,
Method:,
Identities:206/207(99%), Positives:207/207(100%), Gaps:0/207(0%)

```
Query  1         METANTQKAEQNTEAQSSLELQNSSFHYVHVKLEEHEPTDLSTKELILKKAKEACKCNHQ  60
                 METANTQKAEQNTEAQSSLELQNSSFHYVHVKLEEHEPTDLSTKELILKKAKEACKCNHQ
Sbjct  60132721  METANTQKAEQNTEAQSSLELQNSSFHYVHVKLEEHEPTDLSTKELILKKAKEACKCNHQ  60132542

Query  61        SIITFFCKLFPVLEWLPRYQIKEQLLGDVISGFMVAIVAIPQSISYSLLASQDPIYGLYT  120
                 SIITFFCKLFPVLEWLPRYQIKEQLLGDVISGFMVAIVAIPQSISYSLLASQDPIYGLYT
Sbjct  60132541  SIITFFCKLFPVLEWLPRYQIKEQLLGDVISGFMVAIVAIPQSISYSLLASQDPIYGLYT  60132362

Query  121       NFFCCIIYFAMATSHHNSVGSFGVLCLMIGQSVNRQLRLAGYGEDDAGFALQMNSTFSSN  180
                 NFFCCIIYFAMATSHHNSVGSFGVLCLMIGQSVNRQLRLAGYGEDDAGFALQMNSTFSSN
Sbjct  60132361  NFFCCIIYFAMATSHHNSVGSFGVLCLMIGQSVNRQLRLAGYGEDDAGFALQMNSTFSSN  60132182

Query  181       GTATCEKSCYAIKVGISLSFLVGLYQI  207
                 GTATCEKSCYAIKVGISLSFLVGLYQ+
Sbjct  60132181  GTATCEKSCYAIKVGISLSFLVGLYQV  60132101
```

FI Green anole *slc26a12*

Query: PREDICTED: sulfate transporter-like [Anolis carolinensis] Query ID: XP_003217397.2 Length: 766

>Anolis carolinensis chromosome 2, AnoCar2.0, whole genome shotgun sequence
Sequence ID: NC_014777.1 Length: 199619895
Range 1: 122061812 to 122063434

Score:1991 bits(4068), Expect:0.0,
Method:,
Identities:540/541(99%), Positives:541/541(100%), Gaps:0/541(0%)

```
Query  226        IYQILLGVFQLGFISVYLSEPLLSGFVTGSSLTIITSQMNLVLGIKIPRHDGVGSLILTW  285
                  I+QILLGVFQLGFISVYLSEPLLSGFVTGSSLTIITSQMNLVLGIKIPRHDGVGSLILTW
Sbjct  122063434  IFQILLGVFQLGFISVYLSEPLLSGFVTGSSLTIITSQMNLVLGIKIPRHDGVGSLILTW  122063255

Query  286        IDIFRYIGKANICDLVTSLVSLAVIVPVKEVNRYYRDKMKAPFPIELLVVIAATLLSYFF  345
                  IDIFRYIGKANICDLVTSLVSLAVIVPVKEVNRYYRDKMKAPFPIELLVVIAATLLSYFF
Sbjct  122063254  IDIFRYIGKANICDLVTSLVSLAVIVPVKEVNRYYRDKMKAPFPIELLVVIAATLLSYFF  122063075

Query  346        DFNSKYKSKICGAIPTGFKQPAVPDLRLLSNLALDAIPIAIIGFAMTVSLAEIFGKKHGY  405
```

```
                 DFNSKYKSKICGAIPTGFKQPAVPDLRLLSNLALDAIPIAIIGFAMTVSLAEIFGKKHGY
Sbjct  122063074  DFNSKYKSKICGAIPTGFKQPAVPDLRLLSNLALDAIPIAIIGFAMTVSLAEIFGKKHGY  122062895

Query  406        PVRANQEMIAIGMGNLIPAFFSCFATSGALTKTLLKESTGCQTQISSLVSSVVMLLILLW  465
                 PVRANQEMIAIGMGNLIPAFFSCFATSGALTKTLLKESTGCQTQISSLVSSVVMLLILLW
Sbjct  122062894  PVRANQEMIAIGMGNLIPAFFSCFATSGALTKTLLKESTGCQTQISSLVSSVVMLLILLW  122062715

Query  466        IAPLFYSLQTCILGVVTIVNLRGGLRTFADIPKMWRISKIDTVVWWVTMLSSSLISTELG  525
                 IAPLFYSLQTCILGVVTIVNLRGGLRTFADIPKMWRISKIDTVVWWVTMLSSSLISTELG
Sbjct  122062714  IAPLFYSLQTCILGVVTIVNLRGGLRTFADIPKMWRISKIDTVVWWVTMLSSSLISTELG  122062535

Query  526        LLVGVCFALLCIIFRTQRPRATLLGRVNDSEIYEDQFTYKRISSIANIKIFRFDTSLYYA  585
                 LLVGVCFALLCIIFRTQRPRATLLGRVNDSEIYEDQFTYKRISSIANIKIFRFDTSLYYA
Sbjct  122062534  LLVGVCFALLCIIFRTQRPRATLLGRVNDSEIYEDQFTYKRISSIANIKIFRFDTSLYYA  122062355

Query  586        NKEYFKSSLFQKTGIQPSLVAAMQKKAKAKAKAIMDKNENCFSSKLNCLKLTKKSTTQVS  645
                 NKEYFKSSLFQKTGIQPSLVAAMQKKAKAKAKAIMDKNENCFSSKLNCLKLTKKSTTQVS
Sbjct  122062354  NKEYFKSSLFQKTGIQPSLVAAMQKKAKAKAKAIMDKNENCFSSKLNCLKLTKKSTTQVS  122062175

Query  646        EDVSAPVIDICTLIIDCGAMQFIDSVGLSVLKEIHQDYGKIGVQVLLANCNPATRHLLQA  705
                 EDVSAPVIDICTLIIDCGAMQFIDSVGLSVLKEIHQDYGKIGVQVLLANCNPATRHLLQA
Sbjct  122062174  EDVSAPVIDICTLIIDCGAMQFIDSVGLSVLKEIHQDYGKIGVQVLLANCNPATRHLLQA  122061995

Query  706        SGWLTGMEDSGLLFFHSIHAAVKFAEQQNQAQQKENEVVRETFLSQEPQNVLVDSSLERS  765
                 SGWLTGMEDSGLLFFHSIHAAVKFAEQQNQAQQKENEVVRETFLSQEPQNVLVDSSLERS
Sbjct  122061994  SGWLTGMEDSGLLFFHSIHAAVKFAEQQNQAQQKENEVVRETFLSQEPQNVLVDSSLERS  122061815

Query  766        L  766
                 L
Sbjct  122061814  L  122061812
```

Range 2: 122064864 to 122065550

Score:857 bits(1750), Expect:0.0,
Method:,
Identities:228/229(99%), Positives:229/229(100%), Gaps:0/229(0%)

```
Query  1          MPVKNPSVIYSTVCFNSCFHFSCLGVMENDTTTPKYEPNNDRPSSTLSQPSVPYVPIKLE  60
                 MPVKNPSVIYSTVCFNSCFHFSCLGVMENDTTTPKYEPNNDRPSSTLSQPSVPYVPIKLE
Sbjct  122065550  MPVKNPSVIYSTVCFNSCFHFSCLGVMENDTTTPKYEPNNDRPSSTLSQPSVPYVPIKLE  122065371

Query  61         EYETPGFSVKIFLKKVEKSCKCNQRSVLNFFLKLFPAIEWLSHYRIKEYLLGDIISGLLV  120
                 EYETPGFSVKIFLKKVEKSCKCNQRSVLNFFLKLFPAIEWLSHYRIKEYLLGDIISGLLV
Sbjct  122065370  EYETPGFSVKIFLKKVEKSCKCNQRSVLNFFLKLFPAIEWLSHYRIKEYLLGDIISGLLV  122065191

Query  121        GIVAIPQSISYALLASQDPIYGLYTNFFCPIIYFAMATSRHVCVGSFGVLCLMIGESVNR  180
                 GIVAIPQSISYALLASQDPIYGLYTNFFCPIIYFAMATSRHVCVGSFGVLCLMIGESVNR
Sbjct  122065190  GIVAIPQSISYALLASQDPIYGLYTNFFCPIIYFAMATSRHVCVGSFGVLCLMIGESVNR  122065011

Query  181        QLRLAGYESDGATVMMANATLNGTVFCDKSCYAISVATALAFLVGIYQI  229
                 QLRLAGYESDGATVMMANATLNGTVFCDKSCYAISVATALAFLVGIYQ+
Sbjct  122065010  QLRLAGYESDGATVMMANATLNGTVFCDKSCYAISVATALAFLVGIYQV  122064864
```

FJ Western clawed frog *slc26a12*

Query: sulfate transporter [Xenopus tropicalis] Query ID: XP_012815261.1 Length: 737

>Xenopus tropicalis strain Nigerian chromosome 3, UCB_Xtro_10.0, whole genome shotgun sequence
Sequence ID: NC_030679.2 Length: 153873357
Range 1: 44152654 to 44154255

Score:1970 bits(4026), Expect:0.0,
Method:,
Identities:534/534(100%), Positives:534/534(100%), Gaps:0/534(0%)

```
Query  204       QILLGVFQLGFISMYLSEPLLSGFVTGSSLTILTSQMKYLFGLKLTSRYGAGSLVLTWID  263
                QILLGVFQLGFISMYLSEPLLSGFVTGSSLTILTSQMKYLFGLKLTSRYGAGSLVLTWID
Sbjct  44152654  QILLGVFQLGFISMYLSEPLLSGFVTGSSLTILTSQMKYLFGLKLTSRYGAGSLVLTWID  44152833

Query  264       VFSNLKNTNICDLVTSIIAIAVIVPVKEINDRFKSKMKIPCPVELIVIIVATLVSHYFDF  323
                VFSNLKNTNICDLVTSIIAIAVIVPVKEINDRFKSKMKIPCPVELIVIIVATLVSHYFDF
Sbjct  44152834  VFSNLKNTNICDLVTSIIAIAVIVPVKEINDRFKSKMKIPCPVELIVIIVATLVSHYFDF  44153013

Query  324       HNNYKASICGTIPTGFKVPRAPNWGLIPSIAADAVPIAIIGFAMTISLAEIFAKKHGYTV  383
                HNNYKASICGTIPTGFKVPRAPNWGLIPSIAADAVPIAIIGFAMTISLAEIFAKKHGYTV
Sbjct  44153014  HNNYKASICGTIPTGFKVPRAPNWGLIPSIAADAVPIAIIGFAMTISLAEIFAKKHGYTV  44153193

Query  384       SSNQEMIAIGTCNFITSFFSGFVSCAALTKSLLRESTGANTQFNGIISSSVLLLVLLAIA  443
                SSNQEMIAIGTCNFITSFFSGFVSCAALTKSLLRESTGANTQFNGIISSSVLLLVLLAIA
Sbjct  44153194  SSNQEMIAIGTCNFITSFFSGFVSCAALTKSLLRESTGANTQFNGIISSSVLLLVLLAIA  44153373

Query  444       PLFYSLQNCVLGVITITSLRGALRKFADTPKMWRISKIDTVVWWVSMLASSLITTEIGLL  503
                PLFYSLQNCVLGVITITSLRGALRKFADTPKMWRISKIDTVVWWVSMLASSLITTEIGLL
Sbjct  44153374  PLFYSLQNCVLGVITITSLRGALRKFADTPKMWRISKIDTVVWWVSMLASSLITTEIGLL  44153553

Query  504       VAVCFSILCVIFRTQRPRATLLAKVTGTEIYEDQFTYKELSNIPNVKIYRFDASLYYANK  563
                VAVCFSILCVIFRTQRPRATLLAKVTGTEIYEDQFTYKELSNIPNVKIYRFDASLYYANK
Sbjct  44153554  VAVCFSILCVIFRTQRPRATLLAKVTGTEIYEDQFTYKELSNIPNVKIYRFDASLYYANK  44153733
```

```
Query  564       DYFKTTLYSKTDINPSLVSALQKKAKKEEEAAAIETKNRFIARFNIIKTSGKIRQSASVS  623
                 DYFKTTLYSKTDINPSLVSALQKKAKKEEEAAAIETKNRFIARFNIIKTSGKIRQSASVS
Sbjct  44153734  DYFKTTLYSKTDINPSLVSALQKKAKKEEEAAAIETKNRFIARFNIIKTSGKIRQSASVS  44153913

Query  624       VPQFDIHSLIIDCGAMQFIDTVGLGVLKEVKNDYEEIGIRVFLANCNPSVRSMLSNGGYM  683
                 VPQFDIHSLIIDCGAMQFIDTVGLGVLKEVKNDYEEIGIRVFLANCNPSVRSMLSNGGYM
Sbjct  44153914  VPQFDIHSLIIDCGAMQFIDTVGLGVLKEVKNDYEEIGIRVFLANCNPSVRSMLSNGGYM  44154093

Query  684       NNASSGIDLHIFHSVHDAVNFAERKYREKQRELQMNDAAFSFDPNQAISDDLKD  737
                 NNASSGIDLHIFHSVHDAVNFAERKYREKQRELQMNDAAFSFDPNQAISDDLKD
Sbjct  44154094  NNASSGIDLHIFHSVHDAVNFAERKYREKQRELQMNDAAFSFDPNQAISDDLKD  44154255
```

Range 2: 44149906 to 44150499

Score:737 bits(1504), Expect:0.0,
Method:,
Identities:197/198(99%), Positives:198/198(100%), Gaps:0/198(0%)

```
Query  8         QDSEDTSKNEQTEQSSSLTFNYIHVKLEKHAQPEQSTKDIIVEKTREYCTSGPKLLISIL  67
                 QDSEDTSKNEQTEQSSSLTFNYIHVKLEKHAQPEQSTKDIIVEKTREYCTSGPKLLISIL
Sbjct  44149906  QDSEDTSKNEQTEQSSSLTFNYIHVKLEKHAQPEQSTKDIIVEKTREYCTSGPKLLISIL  44150085

Query  68        FRIFPVLQWFPRYKIKKYLPGDITSGLIVGIVTIPQSIAYSVLANQDPIYGLYTNFFCCI  127
                 FRIFPVLQWFPRYKIKKYLPGDITSGLIVGIVTIPQSIAYSVLANQDPIYGLYTNFFCCI
Sbjct  44150086  FRIFPVLQWFPRYKIKKYLPGDITSGLIVGIVTIPQSIAYSVLANQDPIYGLYTNFFCCI  44150265

Query  128       IYFFMATSHHNCVGTFGVLCLMVGESVSKQLQAAGYNTDGTTTAAINSTLVGNMTCDKSC  187
                 IYFFMATSHHNCVGTFGVLCLMVGESVSKQLQAAGYNTDGTTTAAINSTLVGNMTCDKSC
Sbjct  44150266  IYFFMATSHHNCVGTFGVLCLMVGESVSKQLQAAGYNTDGTTTAAINSTLVGNMTCDKSC  44150445

Query  188       YAITVATSLTFIVGVYQI  205
                 YAITVATSLTFIVGVYQ+
Sbjct  44150446  YAITVATSLTFIVGVYQV  44150499
```

FK Two-lined caecilian *slc26a12*

Query: sulfate transporter-like isoform X1 [Rhinatrema bivittatum] Query ID: XP_029439129.1 Length: 750

>Rhinatrema bivittatum chromosome 18, aRhiBiv1.1, whole genome shotgun sequence
Sequence ID: NC_042632.1 Length: 61353747
Range 1: 56893095 to 56894699

Score:1982 bits(4050), Expect:0.0,
Method:,
Identities:534/535(99%), Positives:535/535(100%), Gaps:0/535(0%)

```
Query  216       YQILLGVFQLGFIAIYLSEPLLSGFVTGSSLTILTSQVKYLFGLSIPRHEGAGSLILTWI  275
                 +QILLGVFQLGFIAIYLSEPLLSGFVTGSSLTILTSQVKYLFGLSIPRHEGAGSLILTWI
Sbjct  56893095  FQILLGVFQLGFIAIYLSEPLLSGFVTGSSLTILTSQVKYLFGLSIPRHEGAGSLILTWI  56893274

Query  276       DIFRYIKETNICDLITSLVALAVIIPAKEINDKFKSKMKIPFPVELLVIIVATLVSHYFD  335
                 DIFRYIKETNICDLITSLVALAVIIPAKEINDKFKSKMKIPFPVELLVIIVATLVSHYFD
Sbjct  56893275  DIFRYIKETNICDLITSLVALAVIIPAKEINDKFKSKMKIPFPVELLVIIVATLVSHYFD  56893454

Query  336       FQQTYKSSICGTIPTGFQVPKAPDWSLFPRLASDALPIAIIGFAMTVSLAEIFAKKHGYS  395
                 FQQTYKSSICGTIPTGFQVPKAPDWSLFPRLASDALPIAIIGFAMTVSLAEIFAKKHGYS
Sbjct  56893455  FQQTYKSSICGTIPTGFQVPKAPDWSLFPRLASDALPIAIIGFAMTVSLAEIFAKKHGYS  56893634

Query  396       VRANQEMIAIGMCNLVPSFFYSFASCAALAKTLLRESTGAHTQLSSLVTSGVLLLVLLVI  455
                 VRANQEMIAIGMCNLVPSFFYSFASCAALAKTLLRESTGAHTQLSSLVTSGVLLLVLLVI
Sbjct  56893635  VRANQEMIAIGMCNLVPSFFYSFASCAALAKTLLRESTGAHTQLSSLVTSGVLLLVLLVI  56893814

Query  456       APLFYSLQNCILGVITITNLRGALRKFADTPNMWRISKVDTIVWWVSMLASSLITTEIGL  515
                 APLFYSLQNCILGVITITNLRGALRKFADTPNMWRISKVDTIVWWVSMLASSLITTEIGL
Sbjct  56893815  APLFYSLQNCILGVITITNLRGALRKFADTPNMWRISKVDTIVWWVSMLASSLITTEIGL  56893994

Query  516       LVAVCFSMLCVIFRTQMPRATLLAKVSDTEIYEDQFTYKELRSIPNVKIFRFDSSLYYAN  575
                 LVAVCFSMLCVIFRTQMPRATLLAKVSDTEIYEDQFTYKELRSIPNVKIFRFDSSLYYAN
Sbjct  56893995  LVAVCFSMLCVIFRTQMPRATLLAKVSDTEIYEDQFTYKELRSIPNVKIFRFDSSLYYAN  56894174

Query  576       KGYFKSMLYKKTEINPALVAAMQRKAKKLQEAAAAAPERRFFPRFNLIKTSSRPSNPLEF  635
                 KGYFKSMLYKKTEINPALVAAMQRKAKKLQEAAAAAPERRFFPRFNLIKTSSRPSNPLEF
Sbjct  56894175  KGYFKSMLYKKTEINPALVAAMQRKAKKLQEAAAAAPERRFFPRFNLIKTSSRPSNPLEF  56894354

Query  636       SIPQLDMHSLIIDCGAMQFIDTVGLDVLKETRRDYEDIGILVFLANCSPSVRHLLQEGGY  695
                 SIPQLDMHSLIIDCGAMQFIDTVGLDVLKETRRDYEDIGILVFLANCSPSVRHLLQEGGY
Sbjct  56894355  SIPQLDMHSLIIDCGAMQFIDTVGLDVLKETRRDYEDIGILVFLANCSPSVRHLLQEGGY  56894534

Query  696       FKNGHGDMEQLLFHSVHDAVQFAEGKYQQQQKEIDRINAAFSFNPNQAIADDLGI  750
                 FKNGHGDMEQLLFHSVHDAVQFAEGKYQQQQKEIDRINAAFSFNPNQAIADDLGI
Sbjct  56894535  FKNGHGDMEQLLFHSVHDAVQFAEGKYQQQQKEIDRINAAFSFNPNQAIADDLGI  56894699
```

Range 2: 56890890 to 56891525

Score:788 bits(1609), Expect:0.0,

```
Method:,
Identities:211/212(99%), Positives:212/212(100%), Gaps:0/212(0%)

Query  7         RMEPRTYSHAEQREEERTSCSSESAQRQACNYRHVRLEELVHPQRSPKELILQRSKELCK  66
                 RMEPRTYSHAEQREEERTSCSSESAQRQACNYRHVRLEELVHPQRSPKELILQRSKELCK
Sbjct  56890890  RMEPRTYSHAEQREEERTSCSSESAQRQACNYRHVRLEELVHPQRSPKELILQRSKELCK  56891069

Query  67        CTPSTLTNLLFKLLPVLKWLPRYQIKEQLPGDFVSGIIVGIVTIPQSIAYALLANQAPIY  126
                 CTPSTLTNLLFKLLPVLKWLPRYQIKEQLPGDFVSGIIVGIVTIPQSIAYALLANQAPIY
Sbjct  56891070  CTPSTLTNLLFKLLPVLKWLPRYQIKEQLPGDFVSGIIVGIVTIPQSIAYALLANQAPIY  56891249

Query  127       GIYTNFFSCIIYFFLATSHHNCVGSFGVLCLMIGEAVNKQLKIAGYIHDDGTTATSLVNA  186
                 GIYTNFFSCIIYFFLATSHHNCVGSFGVLCLMIGEAVNKQLKIAGYIHDDGTTATSLVNA
Sbjct  56891250  GIYTNFFSCIIYFFLATSHHNCVGSFGVLCLMIGEAVNKQLKIAGYIHDDGTTATSLVNA  56891429

Query  187       TMAANGSIICDKSCYAITVATSLTFLVGVYQI  218
                 TMAANGSIICDKSCYAITVATSLTFLVGVYQ+
Sbjct  56891430  TMAANGSIICDKSCYAITVATSLTFLVGVYQV  56891525
```

FL Coelacanth *slc26a12*

```
Query: PREDICTED: sulfate transporter-like [Latimeria chalumnae] Query ID: XP_006003851.1 Length: 778


>Latimeria chalumnae isolate SAIAB 97564 unplaced genomic scaffold, LatCha1 scaffold00970, whole genome shotgun sequence
Sequence ID: NW_005819980.1 Length: 831416
Range 1: 793105 to 794685

Score:1948 bits(3980), Expect:0.0,
Method:,
Identities:526/527(99%), Positives:527/527(100%), Gaps:0/527(0%)

Query  252     YQVAMGIFRLGFISVYLSEPLLSGFVTGASLTVLTSQVKYLFGLKIPRYTGAGSLALTWI  311
               +QVAMGIFRLGFISVYLSEPLLSGFVTGASLTVLTSQVKYLFGLKIPRYTGAGSLALTWI
Sbjct  793105  FQVAMGIFRLGFISVYLSEPLLSGFVTGASLTVLTSQVKYLFGLKIPRYTGAGSLALTWI  793284

Query  312     DIFTHIKETNICDLITSIVALAVSVPVKEINDRFKSKLKAPIPIELLVVIGATIISHYFD  371
               DIFTHIKETNICDLITSIVALAVSVPVKEINDRFKSKLKAPIPIELLVVIGATIISHYFD
Sbjct  793285  DIFTHIKETNICDLITSIVALAVSVPVKEINDRFKSKLKAPIPIELLVVIGATIISHYFD  793464

Query  372     FNGNYKSSISGSIPTGFLRPKSPDWSILPNVAADAVPIAIIGFATTISLAELFAKKHKYT  431
               FNGNYKSSISGSIPTGFLRPKSPDWSILPNVAADAVPIAIIGFATTISLAELFAKKHKYT
Sbjct  793465  FNGNYKSSISGSIPTGFLRPKSPDWSILPNVAADAVPIAIIGFATTISLAELFAKKHKYT  793644

Query  432     IDANQEMIALGMCNVVPAFFYSFASSAALAKTLLKESTGAHTQLNGLVSCGVLLLVLLLL  491
               IDANQEMIALGMCNVVPAFFYSFASSAALAKTLLKESTGAHTQLNGLVSCGVLLLVLLLL
Sbjct  793645  IDANQEMIALGMCNVVPAFFYSFASSAALAKTLLKESTGAHTQLNGLVSCGVLLLVLLLL  793824

Query  492     APLFYSLQKCILAIVTIINLKGALRKFYDVPNMWRINKIDTIIWWVTMLASSLITTELGL  551
               APLFYSLQKCILAIVTIINLKGALRKFYDVPNMWRINKIDTIIWWVTMLASSLITTELGL
Sbjct  793825  APLFYSLQKCILAIVTIINLKGALRKFYDVPNMWRINKIDTIIWWVTMLASSLITTELGL  794004

Query  552     LIGVCFSIFCVIFRTQRPRATLLAQVKNTEIYEDEFSYKKLNSIPNIKIFRFDTSLYYAN  611
               LIGVCFSIFCVIFRTQRPRATLLAQVKNTEIYEDEFSYKKLNSIPNIKIFRFDTSLYYAN
Sbjct  794005  LIGVCFSIFCVIFRTQRPRATLLAQVKNTEIYEDEFSYKKLNSIPNIKIFRFDTSLYYAN  794184

Query  612     KRYFKSMLYKKTGVNPTLLVAKQKKALKAQKNNENKSKFFSKHNISSNTTRSQKEVKEVD  671
               KRYFKSMLYKKTGVNPTLLVAKQKKALKAQKNNENKSKFFSKHNISSNTTRSQKEVKEVD
Sbjct  794185  KRYFKSMLYKKTGVNPTLLVAKQKKALKAQKNNENKSKFFSKHNISSNTTRSQKEVKEVD  794364

Query  672     APHVNMHFLIIDCGAMQFIDTVGLNVLKETLAEYREIHIDVLLANCNPSVRQLVQQSNYF  731
               APHVNMHFLIIDCGAMQFIDTVGLNVLKETLAEYREIHIDVLLANCNPSVRQLVQQSNYF
Sbjct  794365  APHVNMHFLIIDCGAMQFIDTVGLNVLKETLAEYREIHIDVLLANCNPSVRQLVQQSNYF  794544

Query  732     GNDTDCLLFHSVHDAVQFAEWEHLKKQKEIHALSAVVPEEVELQTKM  778
               GNDTDCLLFHSVHDAVQFAEWEHLKKQKEIHALSAVVPEEVELQTKM
Sbjct  794545  GNDTDCLLFHSVHDAVQFAEWEHLKKQKEIHALSAVVPEEVELQTKM  794685




Range 2: 790904 to 791530

Score:778 bits(1588), Expect:0.0,
Method:,
Identities:209/209(100%), Positives:209/209(100%), Gaps:0/209(0%)

Query  46      CGVRMDMDFSDQLKVRSSLGSTQDSPPLYMQIKLEEHEKEPVNIKELMLKKIKKVSTCTP  105
               CGVRMDMDFSDQLKVRSSLGSTQDSPPLYMQIKLEEHEKEPVNIKELMLKKIKKVSTCTP
Sbjct  790904  CGVRMDMDFSDQLKVRSSLGSTQDSPPLYMQIKLEEHEKEPVNIKELMLKKIKKVSTCTP  791083

Query  106     KRVVNLVVALFPVLKWLPQYKIKEYLLGDIMSGIILAIVVIPQSIAYALLANVDPIYGLY  165
               KRVVNLVVALFPVLKWLPQYKIKEYLLGDIMSGIILAIVVIPQSIAYALLANVDPIYGLY
Sbjct  791084  KRVVNLVVALFPVLKWLPQYKIKEYLLGDIMSGIILAIVVIPQSIAYALLANVDPIYGLY  791263

Query  166     TNFFSVLIYFFMSTSHHNCVGAFGVLCLMIGESVNKHVQNAGYDTVDDAAVSLLSNTTLP  225
               TNFFSVLIYFFMSTSHHNCVGAFGVLCLMIGESVNKHVQNAGYDTVDDAAVSLLSNTTLP
Sbjct  791264  TNFFSVLIYFFMSTSHHNCVGAFGVLCLMIGESVNKHVQNAGYDTVDDAAVSLLSNTTLP  791443

Query  226     SNGTLACDKSCYAIGVATALTFLVGIYQV  254
               SNGTLACDKSCYAIGVATALTFLVGIYQV
Sbjct  791444  SNGTLACDKSCYAIGVATALTFLVGIYQV  791530
```

```
Range 3: 788946 to 789077

Score:163 bits(330), Expect:2e-44,
Method:,
Identities:44/44(100%), Positives:44/44(100%), Gaps:0/44(0%)

Query  1       MGMTTNVSQTATCAKRLLFISVFANEEMNKLTKLGTELPSGLEI  44
               MGMTTNVSQTATCAKRLLFISVFANEEMNKLTKLGTELPSGLEI
Sbjct  788946  MGMTTNVSQTATCAKRLLFISVFANEEMNKLTKLGTELPSGLEI  789077
```

Supplementary Fig. S1. tBLASTn analyses of *slc26a1*, *slc26a2*, and *slc26a12* for vertebrate species listed in Table 1.

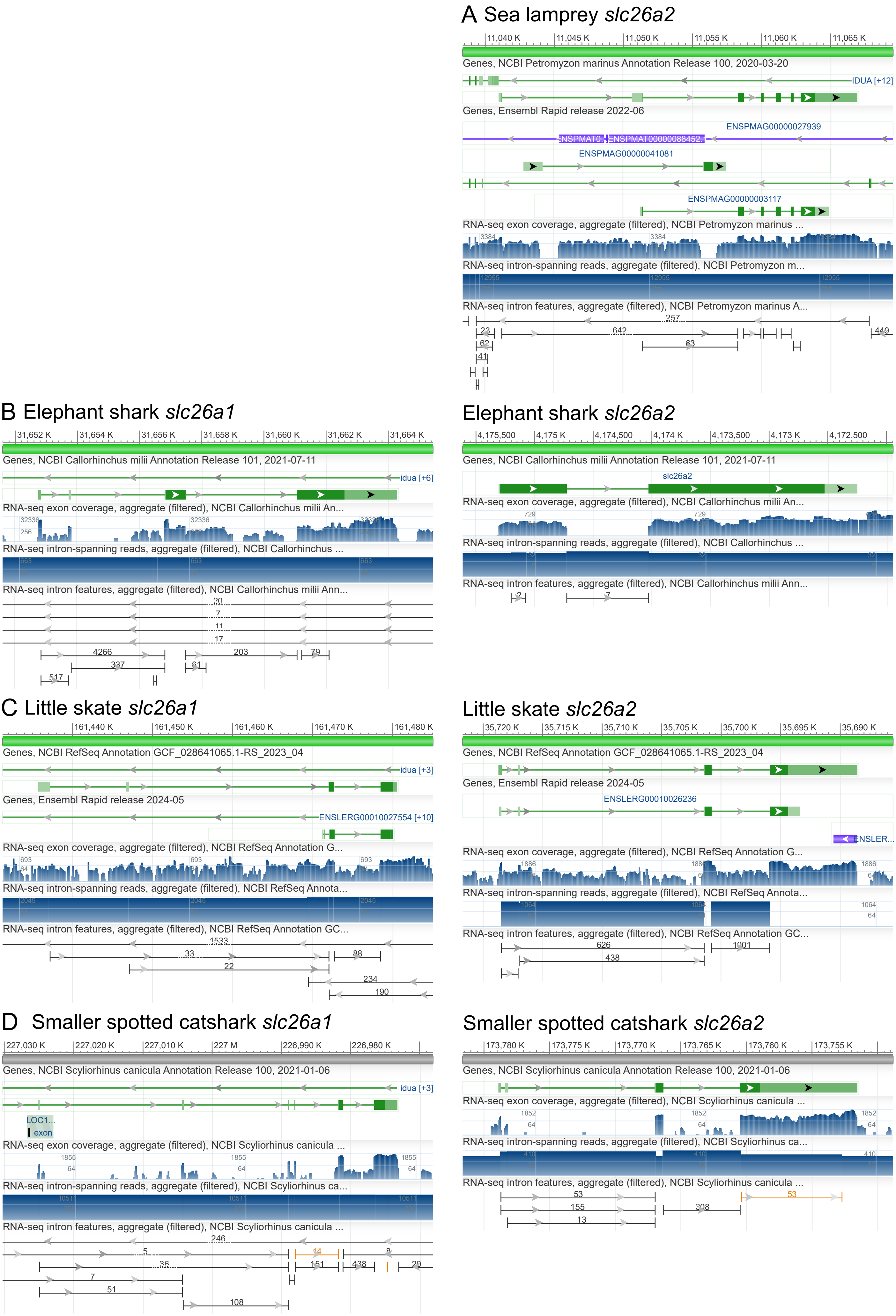
A Sea lamprey slc26a2
11,040 K
11,045 K
11,050 K
11,055 K
11,060 K
11,065 K
Genes, NCBI Petromyzon marinus Annotation Release 100, 2020-03-20
IDUA [+12]
Genes, Ensembl Rapid release 2022-06
ENSPMAG00000027939
ENSPMAG00000041081
ENSPMAG00000003117
RNA-seq exon coverage, aggregate (filtered), NCBI Petromyzon marinus ...
RNA-seq intron-spanning reads, aggregate (filtered), NCBI Petromyzon m...
RNA-seq intron features, aggregate (filtered), NCBI Petromyzon marinus A...
B Elephant shark slc26a1
31,652 K
31,654 K
31,656 K
31,658 K
31,660 K
31,662 K
31,664 K
Genes, NCBI Callorhinchus milii Annotation Release 101, 2021-07-11
idua [+6]
RNA-seq exon coverage, aggregate (filtered), NCBI Callorhinchus milii An...
RNA-seq intron-spanning reads, aggregate (filtered), NCBI Callorhinchus ...
RNA-seq intron features, aggregate (filtered), NCBI Callorhinchus milii Ann...
Elephant shark slc26a2
4,175,500
4,175 K
4,174,500
4,174 K
4,173,500
4,173 K
4,172,500
Genes, NCBI Callorhinchus milii Annotation Release 101, 2021-07-11
slc26a2
RNA-seq exon coverage, aggregate (filtered), NCBI Callorhinchus milii An...
RNA-seq intron-spanning reads, aggregate (filtered), NCBI Callorhinchus ...
RNA-seq intron features, aggregate (filtered), NCBI Callorhinchus milii Ann...
C Little skate slc26a1
161,440 K
161,450 K
161,460 K
161,470 K
161,480 K
Genes, NCBI RefSeq Annotation GCF_028641065.1-RS_2023_04
idua [+3]
Genes, Ensembl Rapid release 2024-05
ENSLERG00010027554 [+10]
RNA-seq exon coverage, aggregate (filtered), NCBI RefSeq Annotation G...
RNA-seq intron-spanning reads, aggregate (filtered), NCBI RefSeq Annota...
RNA-seq intron features, aggregate (filtered), NCBI RefSeq Annotation GC...
Little skate slc26a2
35,720 K
35,715 K
35,710 K
35,705 K
35,700 K
35,695 K
35,690 K
Genes, NCBI RefSeq Annotation GCF_028641065.1-RS_2023_04
Genes, Ensembl Rapid release 2024-05
ENSLERG00010026236
ENSLER...
RNA-seq exon coverage, aggregate (filtered), NCBI RefSeq Annotation G...
RNA-seq intron-spanning reads, aggregate (filtered), NCBI RefSeq Annota...
RNA-seq intron features, aggregate (filtered), NCBI RefSeq Annotation GC...
D Smaller spotted catshark slc26a1
227,030 K
227,020 K
227,010 K
227 M
226,990 K
226,980 K
Genes, NCBI Scyliorhinus canicula Annotation Release 100, 2021-01-06
idua [+3]
LOC1...
exon
RNA-seq exon coverage, aggregate (filtered), NCBI Scyliorhinus canicula ...
RNA-seq intron-spanning reads, aggregate (filtered), NCBI Scyliorhinus ca...
RNA-seq intron features, aggregate (filtered), NCBI Scyliorhinus canicula ...
Smaller spotted catshark slc26a2
173,780 K
173,775 K
173,770 K
173,765 K
173,760 K
173,755 K
Genes, NCBI Scyliorhinus canicula Annotation Release 100, 2021-01-06
RNA-seq exon coverage, aggregate (filtered), NCBI Scyliorhinus canicula ...
RNA-seq intron-spanning reads, aggregate (filtered), NCBI Scyliorhinus ca...
RNA-seq intron features, aggregate (filtered), NCBI Scyliorhinus canicula ...

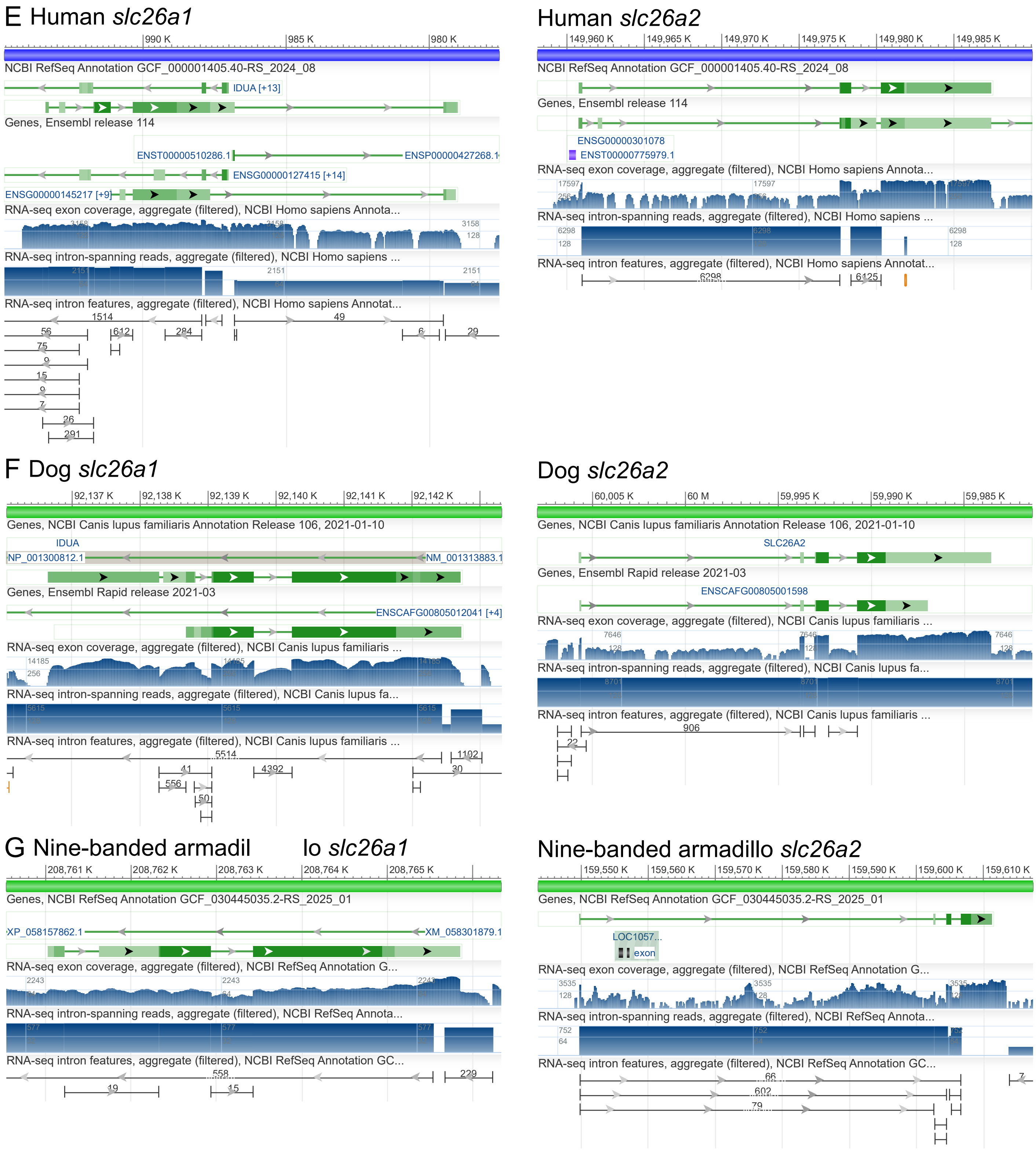
E Human slc26a1
Human slc26a2
F Dog slc26a1
Dog slc26a2
G Nine-banded armadillo slc26a1
Nine-banded armadillo slc26a2
NCBI RefSeq Annotation GCF_000001405.40-RS_2024_08
Genes, Ensembl release 114
IDUA [+13]
ENST00000510286.1
ENSP00000427268.1
ENSG00000127415 [+14]
ENSG00000145217 [+9]
ENSG00000301078
ENST00000775979.1
RNA-seq exon coverage, aggregate (filtered), NCBI Homo sapiens Annota...
RNA-seq intron-spanning reads, aggregate (filtered), NCBI Homo sapiens ...
RNA-seq intron features, aggregate (filtered), NCBI Homo sapiens Annotat...
Genes, NCBI Canis lupus familiaris Annotation Release 106, 2021-01-10
IDUA
NP_001300812.1
NM_001313883.1
SLC26A2
Genes, Ensembl Rapid release 2021-03
ENSCAFG00805012041 [+4]
ENSCAFG00805001598
RNA-seq exon coverage, aggregate (filtered), NCBI Canis lupus familiaris ...
RNA-seq intron-spanning reads, aggregate (filtered), NCBI Canis lupus fa...
RNA-seq intron features, aggregate (filtered), NCBI Canis lupus familiaris ...
Genes, NCBI RefSeq Annotation GCF_030445035.2-RS_2025_01
XP_058157862.1
XM_058301879.1
LOC1057...
exon
RNA-seq exon coverage, aggregate (filtered), NCBI RefSeq Annotation G...
RNA-seq intron-spanning reads, aggregate (filtered), NCBI RefSeq Annota...
RNA-seq intron features, aggregate (filtered), NCBI RefSeq Annotation GC...

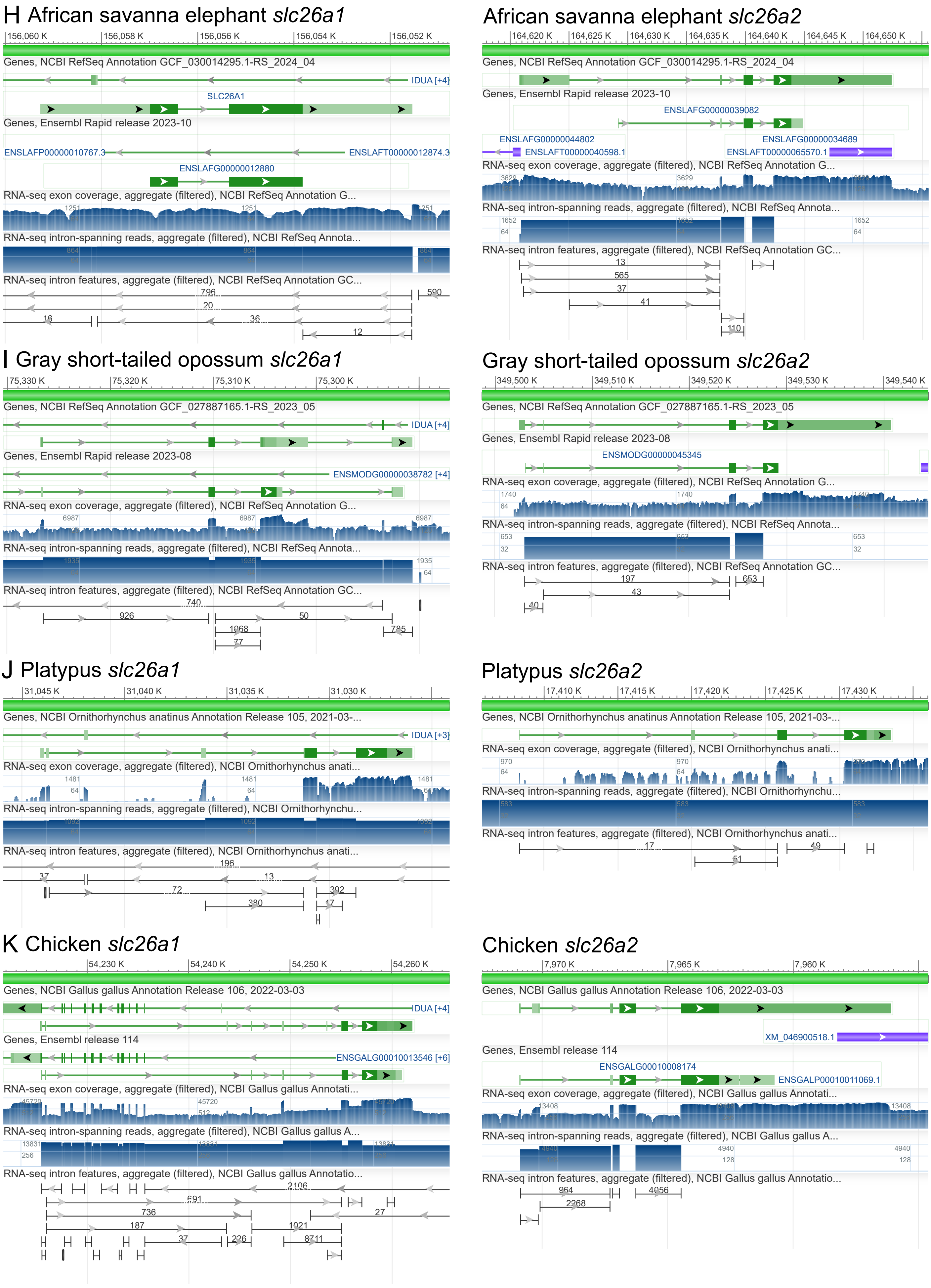

H African savanna elephant slc26a1
African savanna elephant slc26a2
Genes, NCBI RefSeq Annotation GCF_030014295.1-RS_2024_04
IDUA [+4]
SLC26A1
Genes, Ensembl Rapid release 2023-10
ENSLAFP00000010767.3
ENSLAFT00000012874.3
ENSLAFG00000012880
ENSLAFG00000039082
ENSLAFG00000044802
ENSLAFT00000040598.1
ENSLAFG00000034689
ENSLAFT00000065570.1
RNA-seq exon coverage, aggregate (filtered), NCBI RefSeq Annotation G...
RNA-seq intron-spanning reads, aggregate (filtered), NCBI RefSeq Annota...
RNA-seq intron features, aggregate (filtered), NCBI RefSeq Annotation GC...
I Gray short-tailed opossum slc26a1
Gray short-tailed opossum slc26a2
Genes, NCBI RefSeq Annotation GCF_027887165.1-RS_2023_05
Genes, Ensembl Rapid release 2023-08
ENSMODG00000038782 [+4]
ENSMODG00000045345
J Platypus slc26a1
Platypus slc26a2
Genes, NCBI Ornithorhynchus anatinus Annotation Release 105, 2021-03-...
IDUA [+3]
RNA-seq exon coverage, aggregate (filtered), NCBI Ornithorhynchus anati...
RNA-seq intron-spanning reads, aggregate (filtered), NCBI Ornithorhynchu...
RNA-seq intron features, aggregate (filtered), NCBI Ornithorhynchus anati...
K Chicken slc26a1
Chicken slc26a2
Genes, NCBI Gallus gallus Annotation Release 106, 2022-03-03
XM_046900518.1
Genes, Ensembl release 114
ENSGALG00010013546 [+6]
ENSGALG00010008174
ENSGALP00010011069.1
RNA-seq exon coverage, aggregate (filtered), NCBI Gallus gallus Annotati...
RNA-seq intron-spanning reads, aggregate (filtered), NCBI Gallus gallus A...
RNA-seq intron features, aggregate (filtered), NCBI Gallus gallus Annotatio...

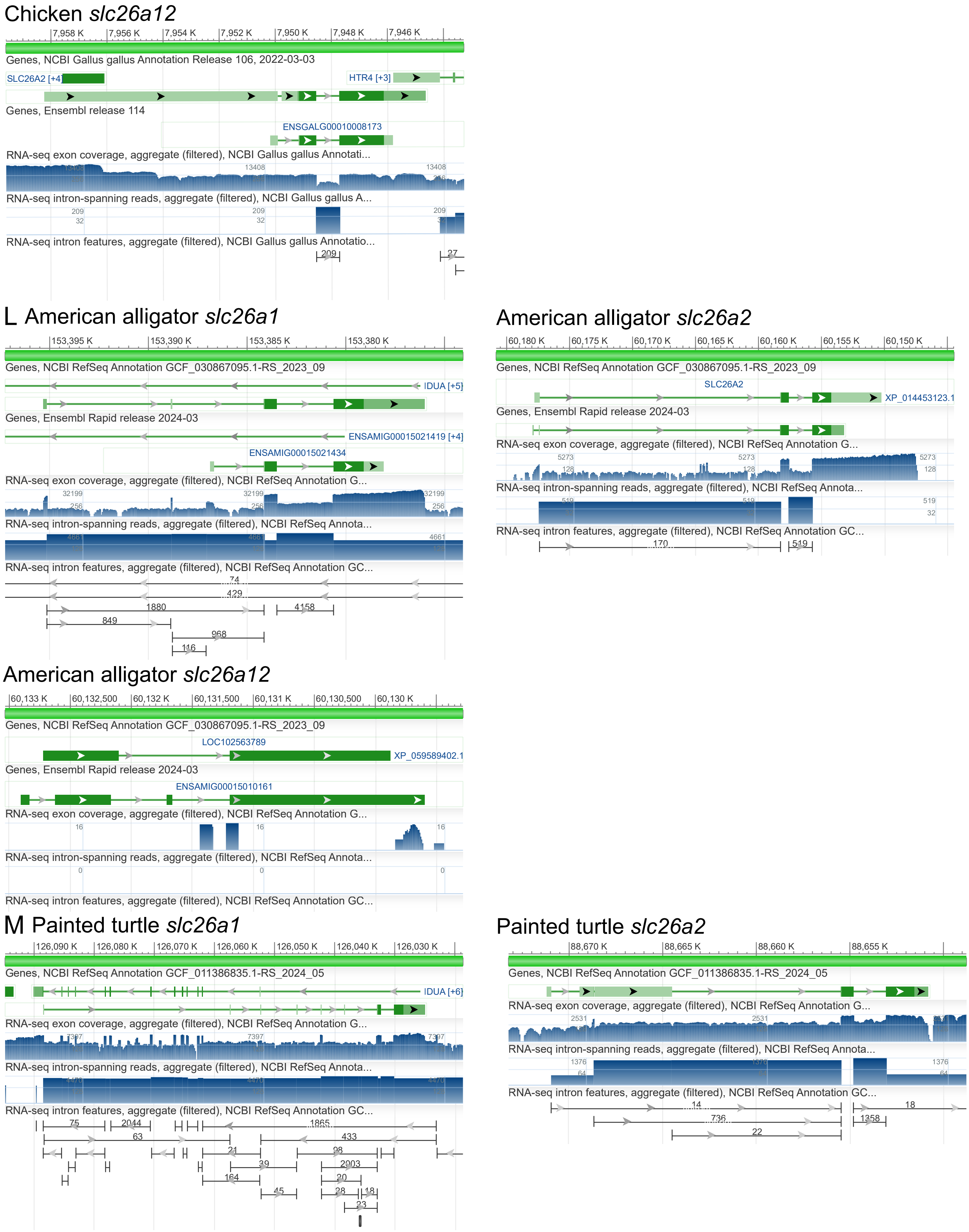

Chicken slc26a12
7,958 K 7,956 K 7,954 K 7,952 K 7,950 K 7,948 K 7,946 K
Genes, NCBI Gallus gallus Annotation Release 106, 2022-03-03
SLC26A2 [+4]
HTR4 [+3]
Genes, Ensembl release 114
ENSGALG00010008173
RNA-seq exon coverage, aggregate (filtered), NCBI Gallus gallus Annotati...
RNA-seq intron-spanning reads, aggregate (filtered), NCBI Gallus gallus A...
RNA-seq intron features, aggregate (filtered), NCBI Gallus gallus Annotatio...
L American alligator slc26a1
153,395 K 153,390 K 153,385 K 153,380 K
Genes, NCBI RefSeq Annotation GCF_030867095.1-RS_2023_09
IDUA [+5]
Genes, Ensembl Rapid release 2024-03
ENSAMIG00015021419 [+4]
ENSAMIG00015021434
RNA-seq exon coverage, aggregate (filtered), NCBI RefSeq Annotation G...
RNA-seq intron-spanning reads, aggregate (filtered), NCBI RefSeq Annota...
RNA-seq intron features, aggregate (filtered), NCBI RefSeq Annotation GC...
American alligator slc26a2
60,180 K 60,175 K 60,170 K 60,165 K 60,160 K 60,155 K 60,150 K
Genes, NCBI RefSeq Annotation GCF_030867095.1-RS_2023_09
SLC26A2
XP_014453123.1
Genes, Ensembl Rapid release 2024-03
RNA-seq exon coverage, aggregate (filtered), NCBI RefSeq Annotation G...
RNA-seq intron-spanning reads, aggregate (filtered), NCBI RefSeq Annota...
RNA-seq intron features, aggregate (filtered), NCBI RefSeq Annotation GC...
American alligator slc26a12
60,133 K 60,132,500 60,132 K 60,131,500 60,131 K 60,130,500 60,130 K
Genes, NCBI RefSeq Annotation GCF_030867095.1-RS_2023_09
LOC102563789
XP_059589402.1
Genes, Ensembl Rapid release 2024-03
ENSAMIG00015010161
RNA-seq exon coverage, aggregate (filtered), NCBI RefSeq Annotation G...
RNA-seq intron-spanning reads, aggregate (filtered), NCBI RefSeq Annota...
RNA-seq intron features, aggregate (filtered), NCBI RefSeq Annotation GC...
M Painted turtle slc26a1
126,090 K 126,080 K 126,070 K 126,060 K 126,050 K 126,040 K 126,030 K
Genes, NCBI RefSeq Annotation GCF_011386835.1-RS_2024_05
IDUA [+6]
RNA-seq exon coverage, aggregate (filtered), NCBI RefSeq Annotation G...
RNA-seq intron-spanning reads, aggregate (filtered), NCBI RefSeq Annota...
RNA-seq intron features, aggregate (filtered), NCBI RefSeq Annotation GC...
Painted turtle slc26a2
88,670 K 88,665 K 88,660 K 88,655 K
Genes, NCBI RefSeq Annotation GCF_011386835.1-RS_2024_05
RNA-seq exon coverage, aggregate (filtered), NCBI RefSeq Annotation G...
RNA-seq intron-spanning reads, aggregate (filtered), NCBI RefSeq Annota...
RNA-seq intron features, aggregate (filtered), NCBI RefSeq Annotation GC...

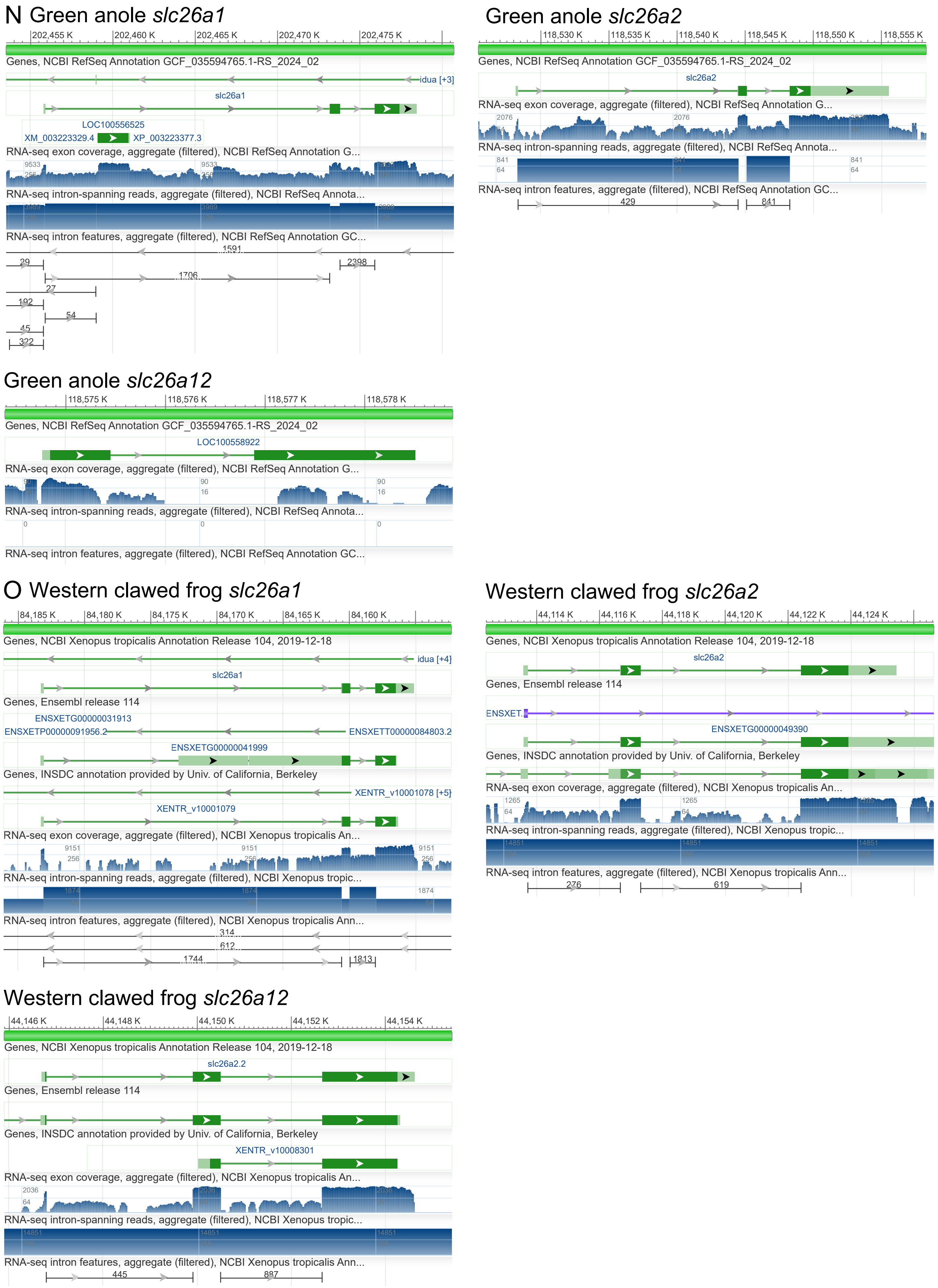

N Green anole slc26a1
Green anole slc26a2
Genes, NCBI RefSeq Annotation GCF_035594765.1-RS_2024_02
idua [+3]
slc26a1
LOC100556525
XM_003223329.4
XP_003223377.3
slc26a2
RNA-seq exon coverage, aggregate (filtered), NCBI RefSeq Annotation G...
RNA-seq intron-spanning reads, aggregate (filtered), NCBI RefSeq Annota...
RNA-seq intron features, aggregate (filtered), NCBI RefSeq Annotation GC...
Green anole slc26a12
LOC100558922
O Western clawed frog slc26a1
Western clawed frog slc26a2
Genes, NCBI Xenopus tropicalis Annotation Release 104, 2019-12-18
idua [+4]
slc26a1
Genes, Ensembl release 114
ENSXETG00000031913
ENSXETP00000091956.2
ENSXETT00000084803.2
ENSXETG00000041999
Genes, INSDC annotation provided by Univ. of California, Berkeley
XENTR_v10001078 [+5]
XENTR_v10001079
RNA-seq exon coverage, aggregate (filtered), NCBI Xenopus tropicalis An...
RNA-seq intron-spanning reads, aggregate (filtered), NCBI Xenopus tropic...
RNA-seq intron features, aggregate (filtered), NCBI Xenopus tropicalis Ann...
slc26a2
ENSXET...
ENSXETG00000049390
Western clawed frog slc26a12
slc26a2.2
XENTR_v10008301

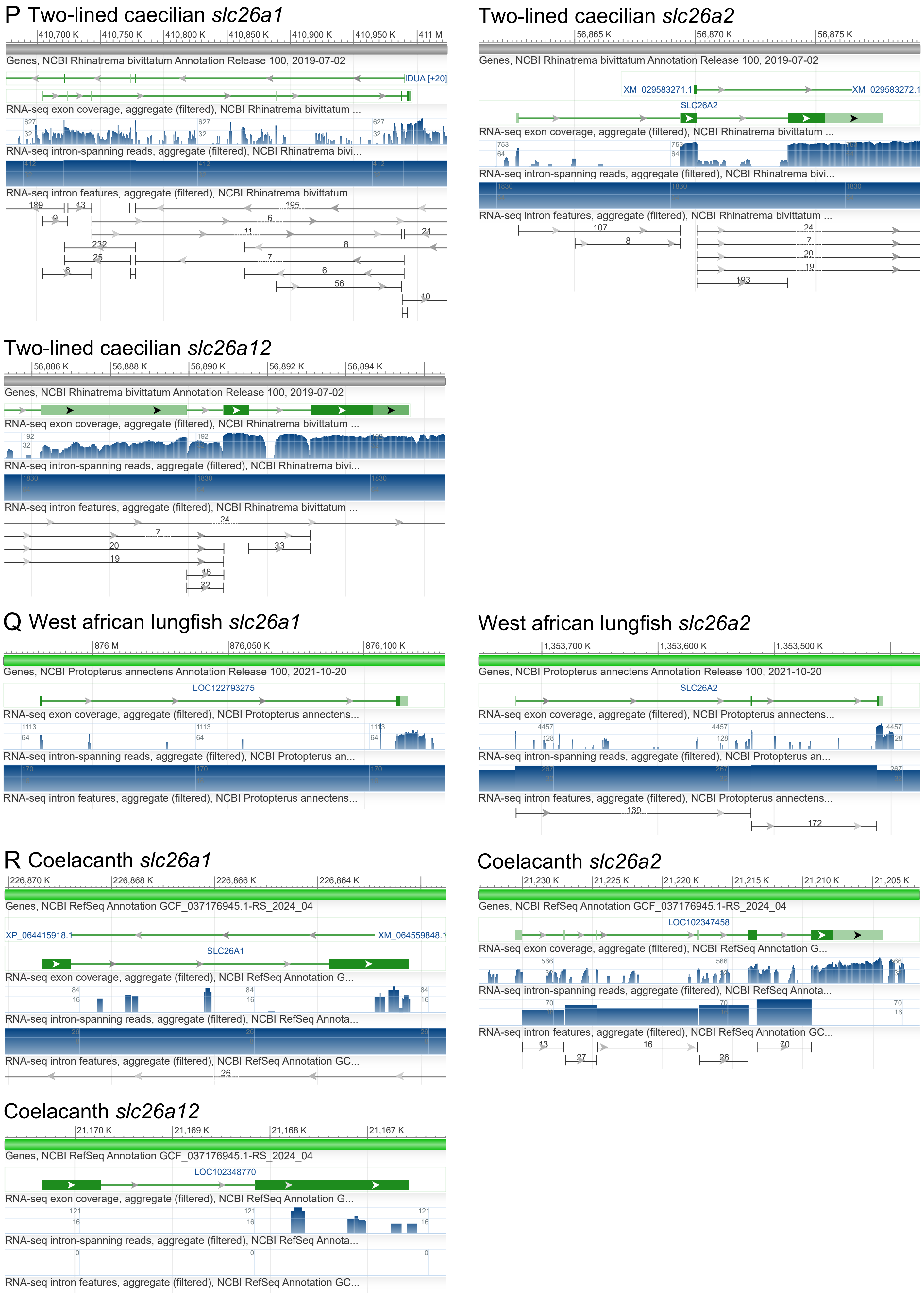
P Two-lined caecilian slc26a1
Genes, NCBI Rhinatrema bivittatum Annotation Release 100, 2019-07-02
IDUA [+20]
RNA-seq exon coverage, aggregate (filtered), NCBI Rhinatrema bivittatum ...
RNA-seq intron-spanning reads, aggregate (filtered), NCBI Rhinatrema bivi...
RNA-seq intron features, aggregate (filtered), NCBI Rhinatrema bivittatum ...
Two-lined caecilian slc26a2
Genes, NCBI Rhinatrema bivittatum Annotation Release 100, 2019-07-02
XM_029583271.1
XM_029583272.1
SLC26A2
RNA-seq exon coverage, aggregate (filtered), NCBI Rhinatrema bivittatum ...
RNA-seq intron-spanning reads, aggregate (filtered), NCBI Rhinatrema bivi...
RNA-seq intron features, aggregate (filtered), NCBI Rhinatrema bivittatum ...
Two-lined caecilian slc26a12
Genes, NCBI Rhinatrema bivittatum Annotation Release 100, 2019-07-02
RNA-seq exon coverage, aggregate (filtered), NCBI Rhinatrema bivittatum ...
RNA-seq intron-spanning reads, aggregate (filtered), NCBI Rhinatrema bivi...
RNA-seq intron features, aggregate (filtered), NCBI Rhinatrema bivittatum ...
Q West african lungfish slc26a1
Genes, NCBI Protopterus annectens Annotation Release 100, 2021-10-20
LOC122793275
RNA-seq exon coverage, aggregate (filtered), NCBI Protopterus annectens...
RNA-seq intron-spanning reads, aggregate (filtered), NCBI Protopterus an...
RNA-seq intron features, aggregate (filtered), NCBI Protopterus annectens...
West african lungfish slc26a2
Genes, NCBI Protopterus annectens Annotation Release 100, 2021-10-20
SLC26A2
RNA-seq exon coverage, aggregate (filtered), NCBI Protopterus annectens...
RNA-seq intron-spanning reads, aggregate (filtered), NCBI Protopterus an...
RNA-seq intron features, aggregate (filtered), NCBI Protopterus annectens...
R Coelacanth slc26a1
Genes, NCBI RefSeq Annotation GCF_037176945.1-RS_2024_04
XP_064415918.1
XM_064559848.1
SLC26A1
RNA-seq exon coverage, aggregate (filtered), NCBI RefSeq Annotation G...
RNA-seq intron-spanning reads, aggregate (filtered), NCBI RefSeq Annota...
RNA-seq intron features, aggregate (filtered), NCBI RefSeq Annotation GC...
Coelacanth slc26a2
Genes, NCBI RefSeq Annotation GCF_037176945.1-RS_2024_04
LOC102347458
RNA-seq exon coverage, aggregate (filtered), NCBI RefSeq Annotation G...
RNA-seq intron-spanning reads, aggregate (filtered), NCBI RefSeq Annota...
RNA-seq intron features, aggregate (filtered), NCBI RefSeq Annotation GC...
Coelacanth slc26a12
Genes, NCBI RefSeq Annotation GCF_037176945.1-RS_2024_04
LOC102348770
RNA-seq exon coverage, aggregate (filtered), NCBI RefSeq Annotation G...
RNA-seq intron-spanning reads, aggregate (filtered), NCBI RefSeq Annota...
RNA-seq intron features, aggregate (filtered), NCBI RefSeq Annotation GC...

S Reedfish *slc26a1*

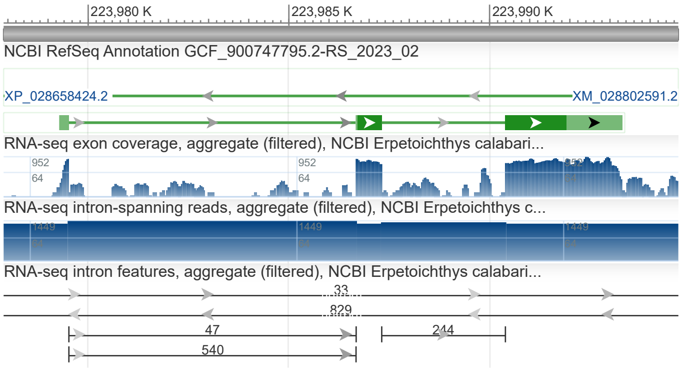

Reedfish *slc26a2*

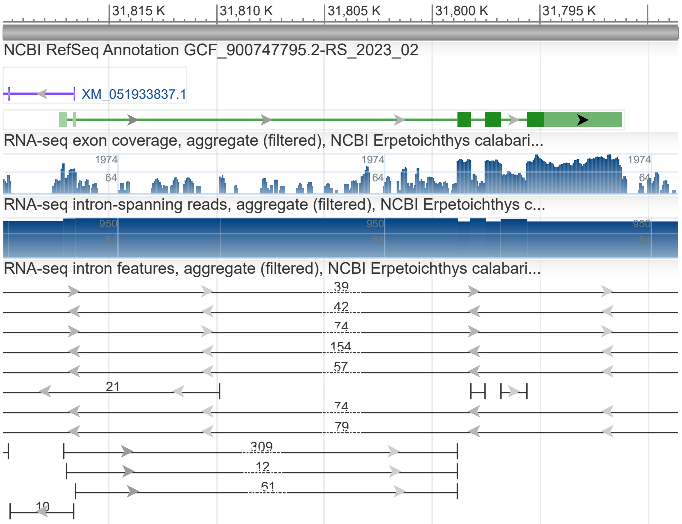

T Gray bichir *slc26a1*

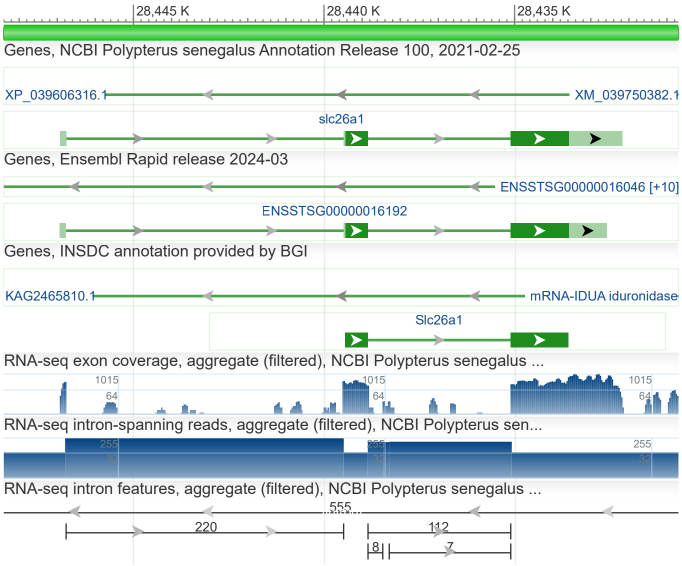

Gray bichir *slc26a2*

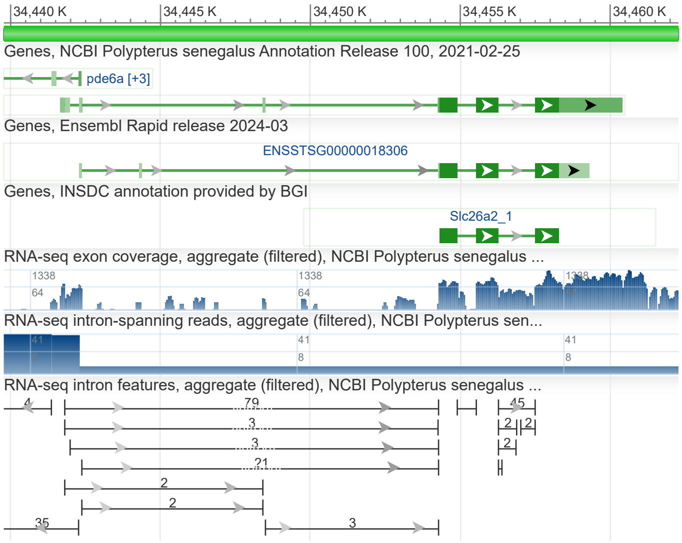

U Sterlet *slc26a1*

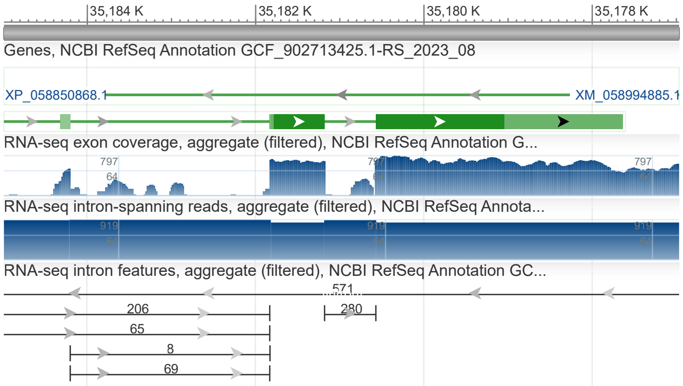

Sterlet *slc26a2*

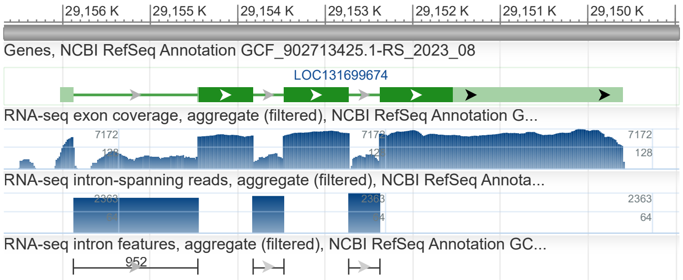

V Spotted gar *slc26a1*

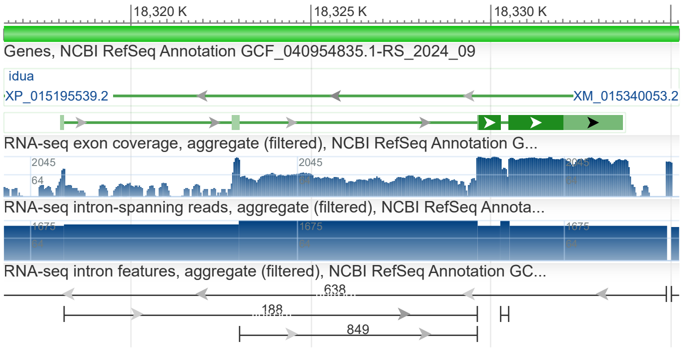

Spotted gar *slc26a2*

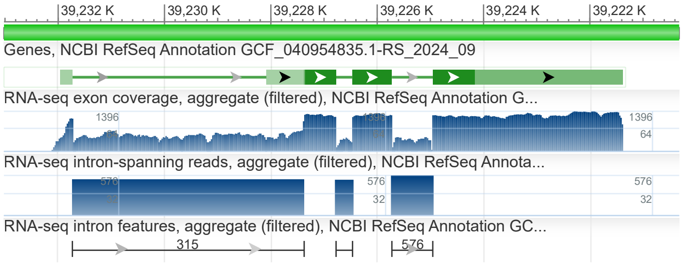

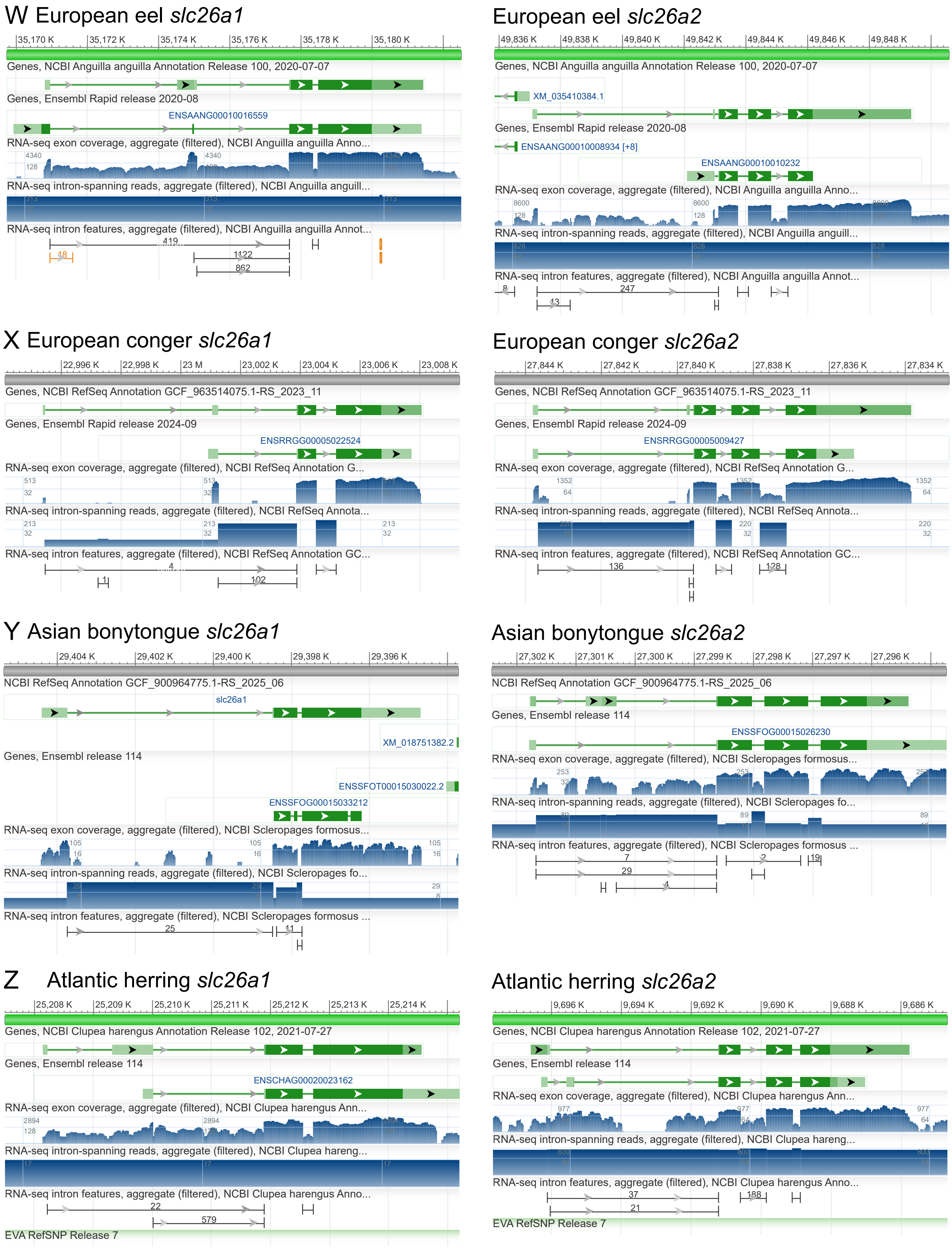
W European eel slc26a1
35,170 K 35,172 K 35,174 K 35,176 K 35,178 K 35,180 K
Genes, NCBI Anguilla anguilla Annotation Release 100, 2020-07-07
Genes, Ensembl Rapid release 2020-08
ENSAANG00010016559
RNA-seq exon coverage, aggregate (filtered), NCBI Anguilla anguilla Anno...
RNA-seq intron-spanning reads, aggregate (filtered), NCBI Anguilla anguill...
RNA-seq intron features, aggregate (filtered), NCBI Anguilla anguilla Annot...
419
18
1122
862
European eel slc26a2
49,836 K 49,838 K 49,840 K 49,842 K 49,844 K 49,846 K 49,848 K
Genes, NCBI Anguilla anguilla Annotation Release 100, 2020-07-07
XM_035410384.1
Genes, Ensembl Rapid release 2020-08
ENSAANG00010008934 [+8]
ENSAANG00010010232
RNA-seq exon coverage, aggregate (filtered), NCBI Anguilla anguilla Anno...
RNA-seq intron-spanning reads, aggregate (filtered), NCBI Anguilla anguill...
RNA-seq intron features, aggregate (filtered), NCBI Anguilla anguilla Annot...
8
247
13
X European conger slc26a1
22,996 K 22,998 K 23 M 23,002 K 23,004 K 23,006 K 23,008 K
Genes, NCBI RefSeq Annotation GCF_963514075.1-RS_2023_11
Genes, Ensembl Rapid release 2024-09
ENSRRGG00005022524
RNA-seq exon coverage, aggregate (filtered), NCBI RefSeq Annotation G...
RNA-seq intron-spanning reads, aggregate (filtered), NCBI RefSeq Annota...
RNA-seq intron features, aggregate (filtered), NCBI RefSeq Annotation GC...
4
1
102
European conger slc26a2
27,844 K 27,842 K 27,840 K 27,838 K 27,836 K 27,834 K
Genes, NCBI RefSeq Annotation GCF_963514075.1-RS_2023_11
Genes, Ensembl Rapid release 2024-09
ENSRRGG00005009427
RNA-seq exon coverage, aggregate (filtered), NCBI RefSeq Annotation G...
RNA-seq intron-spanning reads, aggregate (filtered), NCBI RefSeq Annota...
RNA-seq intron features, aggregate (filtered), NCBI RefSeq Annotation GC...
136
128
Y Asian bonytongue slc26a1
29,404 K 29,402 K 29,400 K 29,398 K 29,396 K
NCBI RefSeq Annotation GCF_900964775.1-RS_2025_06
slc26a1
XM_018751382.2
Genes, Ensembl release 114
ENSSFOT00015030022.2
ENSSFOG00015033212
RNA-seq exon coverage, aggregate (filtered), NCBI Scleropages formosus...
RNA-seq intron-spanning reads, aggregate (filtered), NCBI Scleropages fo...
RNA-seq intron features, aggregate (filtered), NCBI Scleropages formosus ...
25
11
Asian bonytongue slc26a2
27,302 K 27,301 K 27,300 K 27,299 K 27,298 K 27,297 K 27,296 K
NCBI RefSeq Annotation GCF_900964775.1-RS_2025_06
Genes, Ensembl release 114
ENSSFOG00015026230
RNA-seq exon coverage, aggregate (filtered), NCBI Scleropages formosus...
RNA-seq intron-spanning reads, aggregate (filtered), NCBI Scleropages fo...
RNA-seq intron features, aggregate (filtered), NCBI Scleropages formosus ...
7
2
19
29
4
Z Atlantic herring slc26a1
25,208 K 25,209 K 25,210 K 25,211 K 25,212 K 25,213 K 25,214 K
Genes, NCBI Clupea harengus Annotation Release 102, 2021-07-27
Genes, Ensembl release 114
ENSCHAG00020023162
RNA-seq exon coverage, aggregate (filtered), NCBI Clupea harengus Ann...
RNA-seq intron-spanning reads, aggregate (filtered), NCBI Clupea hareng...
RNA-seq intron features, aggregate (filtered), NCBI Clupea harengus Anno...
22
579
EVA RefSNP Release 7
Atlantic herring slc26a2
9,696 K 9,694 K 9,692 K 9,690 K 9,688 K 9,686 K
Genes, NCBI Clupea harengus Annotation Release 102, 2021-07-27
Genes, Ensembl release 114
RNA-seq exon coverage, aggregate (filtered), NCBI Clupea harengus Ann...
RNA-seq intron-spanning reads, aggregate (filtered), NCBI Clupea hareng...
RNA-seq intron features, aggregate (filtered), NCBI Clupea harengus Anno...
37
188
21
EVA RefSNP Release 7

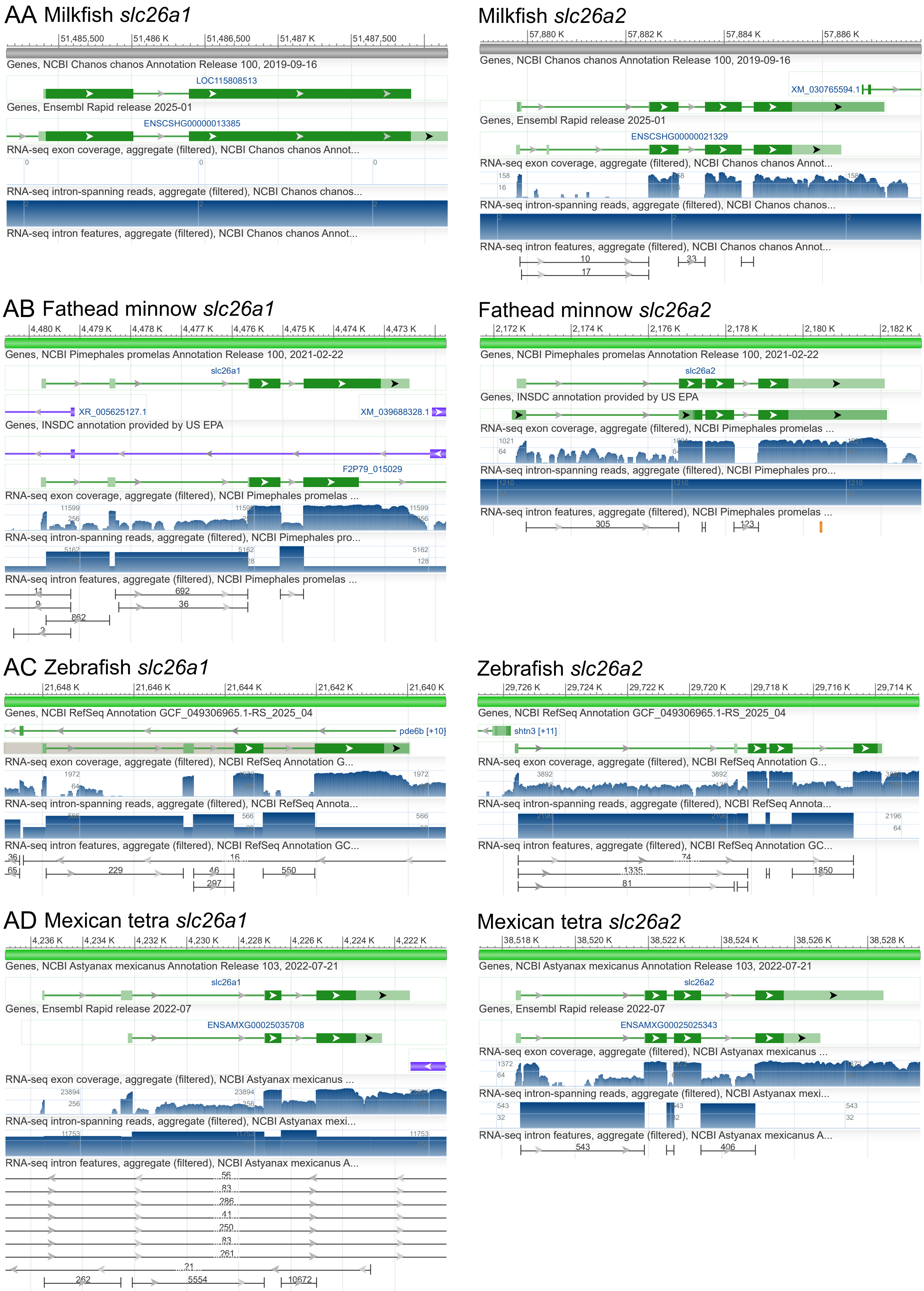

AA Milkfish slc26a1
Genes, NCBI Chanos chanos Annotation Release 100, 2019-09-16
LOC115808513
Genes, Ensembl Rapid release 2025-01
ENSCSHG00000013385
RNA-seq exon coverage, aggregate (filtered), NCBI Chanos chanos Annot...
RNA-seq intron-spanning reads, aggregate (filtered), NCBI Chanos chanos...
RNA-seq intron features, aggregate (filtered), NCBI Chanos chanos Annot...
Milkfish slc26a2
Genes, NCBI Chanos chanos Annotation Release 100, 2019-09-16
XM_030765594.1
Genes, Ensembl Rapid release 2025-01
ENSCSHG00000021329
RNA-seq exon coverage, aggregate (filtered), NCBI Chanos chanos Annot...
RNA-seq intron-spanning reads, aggregate (filtered), NCBI Chanos chanos...
RNA-seq intron features, aggregate (filtered), NCBI Chanos chanos Annot...
AB Fathead minnow slc26a1
Genes, NCBI Pimephales promelas Annotation Release 100, 2021-02-22
slc26a1
XR_005625127.1
XM_039688328.1
Genes, INSDC annotation provided by US EPA
F2P79_015029
RNA-seq exon coverage, aggregate (filtered), NCBI Pimephales promelas ...
RNA-seq intron-spanning reads, aggregate (filtered), NCBI Pimephales pro...
RNA-seq intron features, aggregate (filtered), NCBI Pimephales promelas ...
Fathead minnow slc26a2
Genes, NCBI Pimephales promelas Annotation Release 100, 2021-02-22
slc26a2
Genes, INSDC annotation provided by US EPA
RNA-seq exon coverage, aggregate (filtered), NCBI Pimephales promelas ...
RNA-seq intron-spanning reads, aggregate (filtered), NCBI Pimephales pro...
RNA-seq intron features, aggregate (filtered), NCBI Pimephales promelas ...
AC Zebrafish slc26a1
Genes, NCBI RefSeq Annotation GCF_049306965.1-RS_2025_04
pde6b [+10]
RNA-seq exon coverage, aggregate (filtered), NCBI RefSeq Annotation G...
RNA-seq intron-spanning reads, aggregate (filtered), NCBI RefSeq Annota...
RNA-seq intron features, aggregate (filtered), NCBI RefSeq Annotation GC...
Zebrafish slc26a2
Genes, NCBI RefSeq Annotation GCF_049306965.1-RS_2025_04
shtn3 [+11]
RNA-seq exon coverage, aggregate (filtered), NCBI RefSeq Annotation G...
RNA-seq intron-spanning reads, aggregate (filtered), NCBI RefSeq Annota...
RNA-seq intron features, aggregate (filtered), NCBI RefSeq Annotation GC...
AD Mexican tetra slc26a1
Genes, NCBI Astyanax mexicanus Annotation Release 103, 2022-07-21
slc26a1
Genes, Ensembl Rapid release 2022-07
ENSAMXG00025035708
RNA-seq exon coverage, aggregate (filtered), NCBI Astyanax mexicanus ...
RNA-seq intron-spanning reads, aggregate (filtered), NCBI Astyanax mexi...
RNA-seq intron features, aggregate (filtered), NCBI Astyanax mexicanus A...
Mexican tetra slc26a2
Genes, NCBI Astyanax mexicanus Annotation Release 103, 2022-07-21
slc26a2
Genes, Ensembl Rapid release 2022-07
ENSAMXG00025025343
RNA-seq exon coverage, aggregate (filtered), NCBI Astyanax mexicanus ...
RNA-seq intron-spanning reads, aggregate (filtered), NCBI Astyanax mexi...
RNA-seq intron features, aggregate (filtered), NCBI Astyanax mexicanus A...

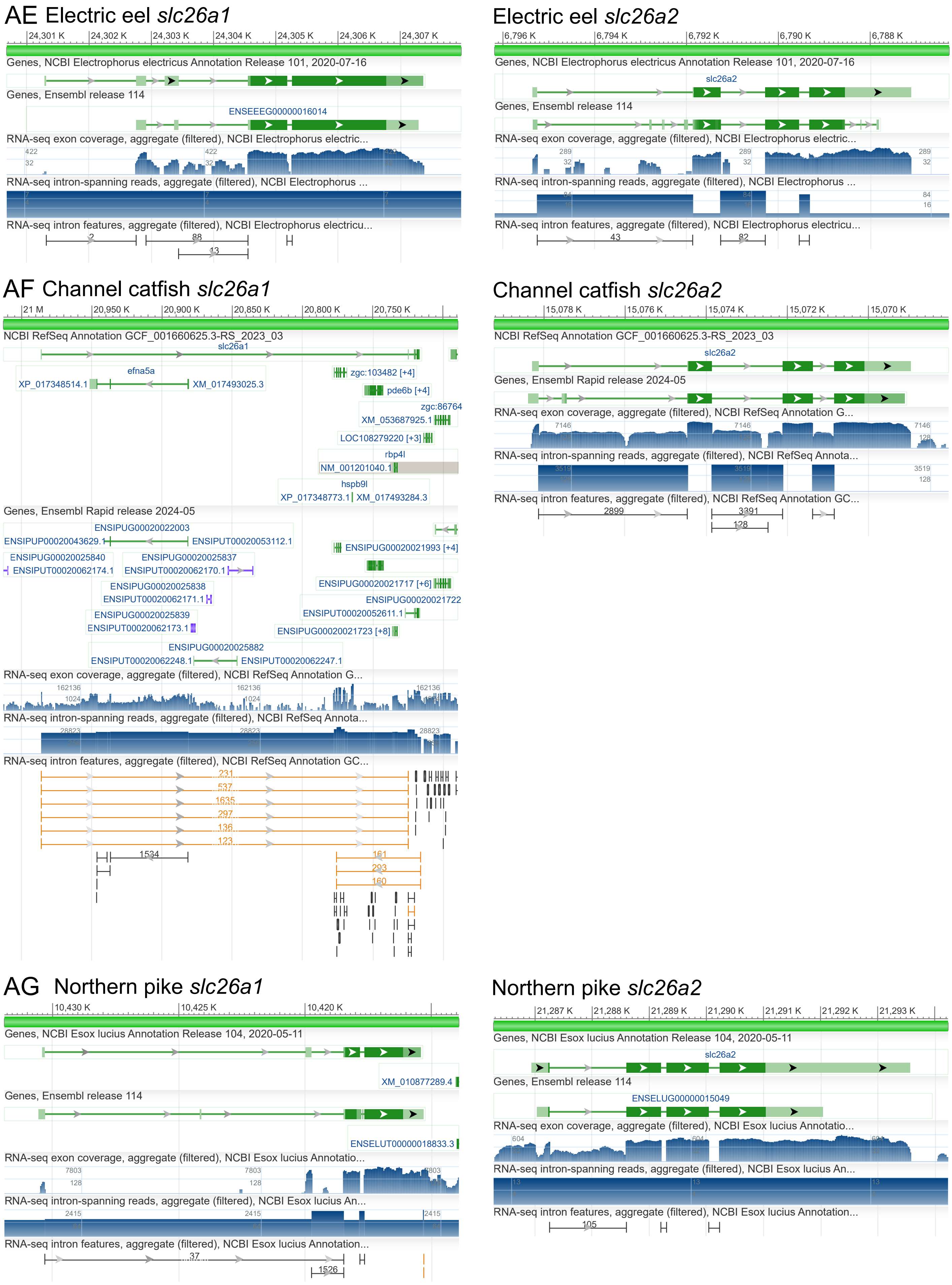

Supplementary Fig. S2. The exon-intron strucutre of *slc26a1*, *slc26a2*, and *slc26a12* in NCBI Genome Data Viewer for the species analyzed in Fig. 1. A-AG) Screen captures of a graphical comparison between predicted exon-intron structure and RNA-seq exon coverage, aggregate (filtered).

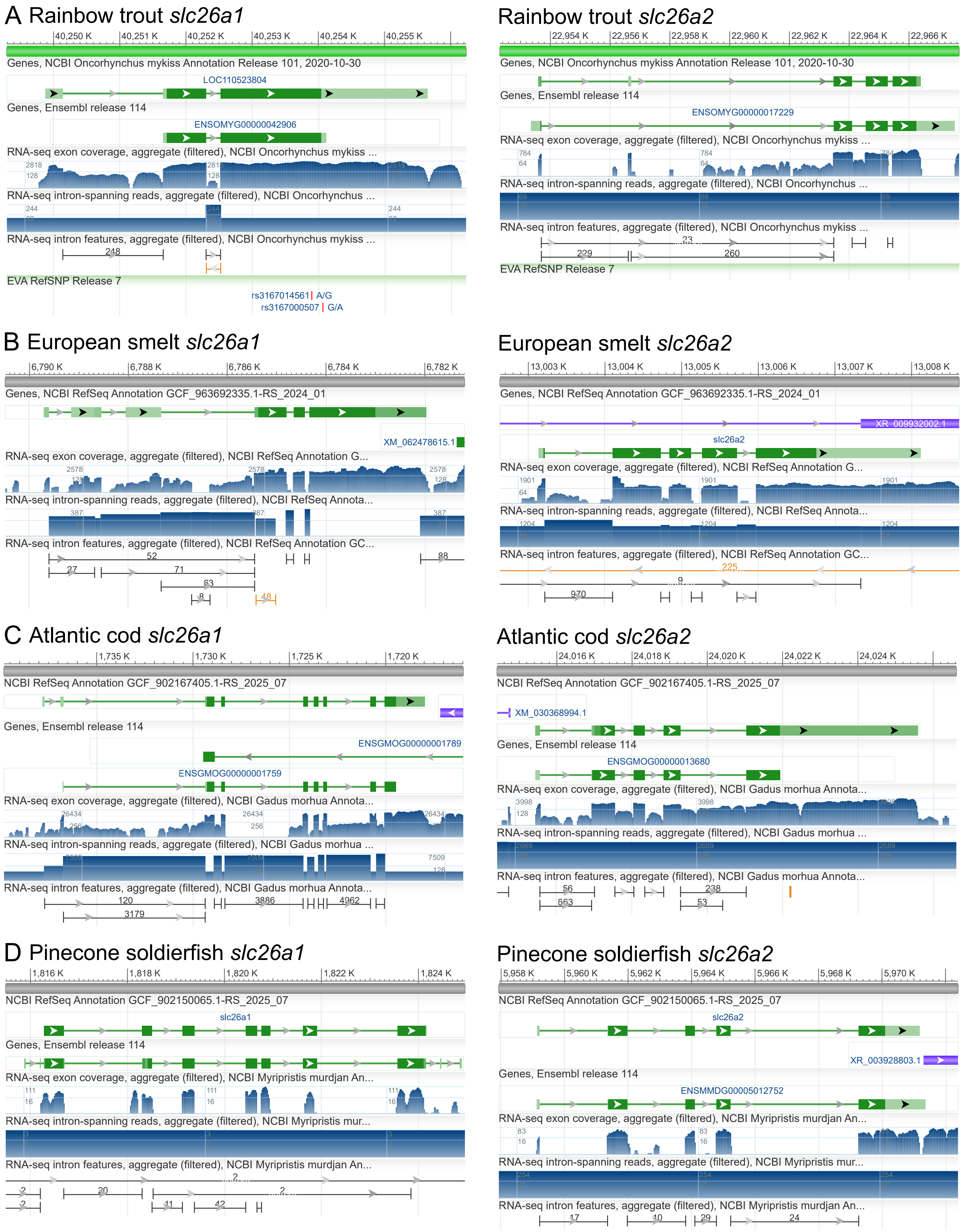

A Rainbow trout slc26a1
40,250 K
40,251 K
40,252 K
40,253 K
40,254 K
40,255 K
Genes, NCBI Oncorhynchus mykiss Annotation Release 101, 2020-10-30
LOC110523804
Genes, Ensembl release 114
ENSOMYG00000042906
RNA-seq exon coverage, aggregate (filtered), NCBI Oncorhynchus mykiss ...
RNA-seq intron-spanning reads, aggregate (filtered), NCBI Oncorhynchus ...
RNA-seq intron features, aggregate (filtered), NCBI Oncorhynchus mykiss ...
248
EVA RefSNP Release 7
rs3167014561 A/G
rs3167000507 G/A
Rainbow trout slc26a2
22,954 K
22,956 K
22,958 K
22,960 K
22,962 K
22,964 K
22,966 K
Genes, NCBI Oncorhynchus mykiss Annotation Release 101, 2020-10-30
Genes, Ensembl release 114
ENSOMYG00000017229
RNA-seq exon coverage, aggregate (filtered), NCBI Oncorhynchus mykiss ...
RNA-seq intron-spanning reads, aggregate (filtered), NCBI Oncorhynchus ...
RNA-seq intron features, aggregate (filtered), NCBI Oncorhynchus mykiss ...
23
229
260
EVA RefSNP Release 7
B European smelt slc26a1
6,790 K
6,788 K
6,786 K
6,784 K
6,782 K
Genes, NCBI RefSeq Annotation GCF_963692335.1-RS_2024_01
XM_062478615.1
RNA-seq exon coverage, aggregate (filtered), NCBI RefSeq Annotation G...
RNA-seq intron-spanning reads, aggregate (filtered), NCBI RefSeq Annota...
RNA-seq intron features, aggregate (filtered), NCBI RefSeq Annotation GC...
52
88
27
71
63
8
48
European smelt slc26a2
13,003 K
13,004 K
13,005 K
13,006 K
13,007 K
13,008 K
Genes, NCBI RefSeq Annotation GCF_963692335.1-RS_2024_01
XR_009932002.1
slc26a2
RNA-seq exon coverage, aggregate (filtered), NCBI RefSeq Annotation G...
RNA-seq intron-spanning reads, aggregate (filtered), NCBI RefSeq Annota...
RNA-seq intron features, aggregate (filtered), NCBI RefSeq Annotation GC...
225
9
970
C Atlantic cod slc26a1
1,735 K
1,730 K
1,725 K
1,720 K
NCBI RefSeq Annotation GCF_902167405.1-RS_2025_07
Genes, Ensembl release 114
ENSGMOG00000001789
ENSGMOG00000001759
RNA-seq exon coverage, aggregate (filtered), NCBI Gadus morhua Annota...
RNA-seq intron-spanning reads, aggregate (filtered), NCBI Gadus morhua ...
RNA-seq intron features, aggregate (filtered), NCBI Gadus morhua Annota...
120
3886
4962
3179
Atlantic cod slc26a2
24,016 K
24,018 K
24,020 K
24,022 K
24,024 K
NCBI RefSeq Annotation GCF_902167405.1-RS_2025_07
XM_030368994.1
Genes, Ensembl release 114
ENSGMOG00000013680
RNA-seq exon coverage, aggregate (filtered), NCBI Gadus morhua Annota...
RNA-seq intron-spanning reads, aggregate (filtered), NCBI Gadus morhua ...
RNA-seq intron features, aggregate (filtered), NCBI Gadus morhua Annota...
56
238
663
53
D Pinecone soldierfish slc26a1
1,816 K
1,818 K
1,820 K
1,822 K
1,824 K
NCBI RefSeq Annotation GCF_902150065.1-RS_2025_07
slc26a1
Genes, Ensembl release 114
RNA-seq exon coverage, aggregate (filtered), NCBI Myripristis murdjan An...
RNA-seq intron-spanning reads, aggregate (filtered), NCBI Myripristis mur...
RNA-seq intron features, aggregate (filtered), NCBI Myripristis murdjan An...
20
11
42
Pinecone soldierfish slc26a2
5,958 K
5,960 K
5,962 K
5,964 K
5,966 K
5,968 K
5,970 K
NCBI RefSeq Annotation GCF_902150065.1-RS_2025_07
slc26a2
XR_003928803.1
Genes, Ensembl release 114
ENSMMDG00005012752
RNA-seq exon coverage, aggregate (filtered), NCBI Myripristis murdjan An...
RNA-seq intron-spanning reads, aggregate (filtered), NCBI Myripristis mur...
RNA-seq intron features, aggregate (filtered), NCBI Myripristis murdjan An...
17
10
29
24

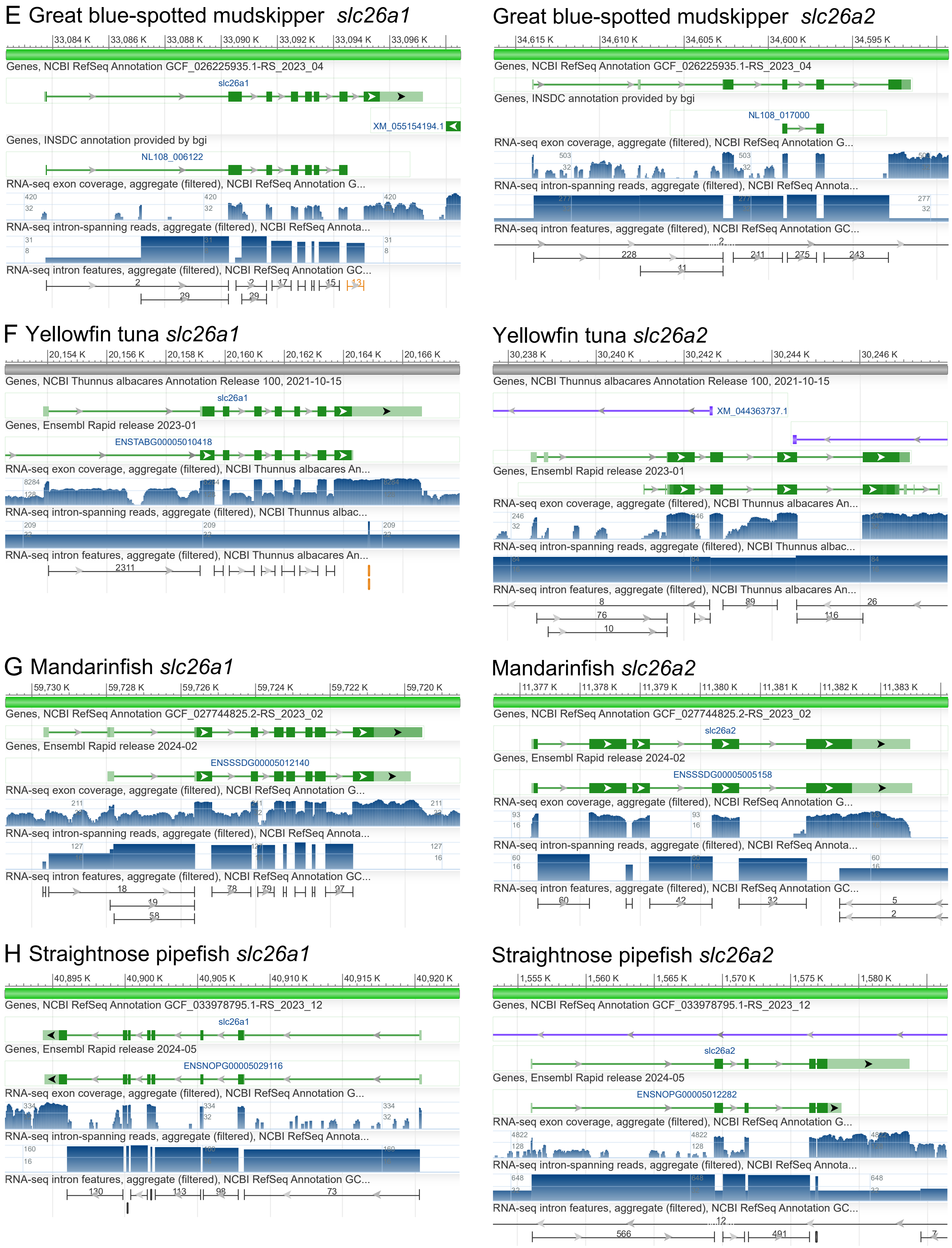
E Great blue-spotted mudskipper slc26a1
Genes, NCBI RefSeq Annotation GCF_026225935.1-RS_2023_04
slc26a1
XM_055154194.1
Genes, INSDC annotation provided by bgi
NL108_006122
RNA-seq exon coverage, aggregate (filtered), NCBI RefSeq Annotation G...
RNA-seq intron-spanning reads, aggregate (filtered), NCBI RefSeq Annota...
RNA-seq intron features, aggregate (filtered), NCBI RefSeq Annotation GC...
Great blue-spotted mudskipper slc26a2
Genes, NCBI RefSeq Annotation GCF_026225935.1-RS_2023_04
Genes, INSDC annotation provided by bgi
NL108_017000
RNA-seq exon coverage, aggregate (filtered), NCBI RefSeq Annotation G...
RNA-seq intron-spanning reads, aggregate (filtered), NCBI RefSeq Annota...
RNA-seq intron features, aggregate (filtered), NCBI RefSeq Annotation GC...
F Yellowfin tuna slc26a1
Genes, NCBI Thunnus albacares Annotation Release 100, 2021-10-15
slc26a1
Genes, Ensembl Rapid release 2023-01
ENSTABG00005010418
RNA-seq exon coverage, aggregate (filtered), NCBI Thunnus albacares An...
RNA-seq intron-spanning reads, aggregate (filtered), NCBI Thunnus albac...
RNA-seq intron features, aggregate (filtered), NCBI Thunnus albacares An...
Yellowfin tuna slc26a2
Genes, NCBI Thunnus albacares Annotation Release 100, 2021-10-15
XM_044363737.1
Genes, Ensembl Rapid release 2023-01
RNA-seq exon coverage, aggregate (filtered), NCBI Thunnus albacares An...
RNA-seq intron-spanning reads, aggregate (filtered), NCBI Thunnus albac...
RNA-seq intron features, aggregate (filtered), NCBI Thunnus albacares An...
G Mandarinfish slc26a1
Genes, NCBI RefSeq Annotation GCF_027744825.2-RS_2023_02
Genes, Ensembl Rapid release 2024-02
ENSSSDG00005012140
RNA-seq exon coverage, aggregate (filtered), NCBI RefSeq Annotation G...
RNA-seq intron-spanning reads, aggregate (filtered), NCBI RefSeq Annota...
RNA-seq intron features, aggregate (filtered), NCBI RefSeq Annotation GC...
Mandarinfish slc26a2
Genes, NCBI RefSeq Annotation GCF_027744825.2-RS_2023_02
slc26a2
Genes, Ensembl Rapid release 2024-02
ENSSSDG00005005158
RNA-seq exon coverage, aggregate (filtered), NCBI RefSeq Annotation G...
RNA-seq intron-spanning reads, aggregate (filtered), NCBI RefSeq Annota...
RNA-seq intron features, aggregate (filtered), NCBI RefSeq Annotation GC...
H Straightnose pipefish slc26a1
Genes, NCBI RefSeq Annotation GCF_033978795.1-RS_2023_12
slc26a1
Genes, Ensembl Rapid release 2024-05
ENSNOPG00005029116
RNA-seq exon coverage, aggregate (filtered), NCBI RefSeq Annotation G...
RNA-seq intron-spanning reads, aggregate (filtered), NCBI RefSeq Annota...
RNA-seq intron features, aggregate (filtered), NCBI RefSeq Annotation GC...
Straightnose pipefish slc26a2
Genes, NCBI RefSeq Annotation GCF_033978795.1-RS_2023_12
slc26a2
Genes, Ensembl Rapid release 2024-05
ENSNOPG00005012282
RNA-seq exon coverage, aggregate (filtered), NCBI RefSeq Annotation G...
RNA-seq intron-spanning reads, aggregate (filtered), NCBI RefSeq Annota...
RNA-seq intron features, aggregate (filtered), NCBI RefSeq Annotation GC...

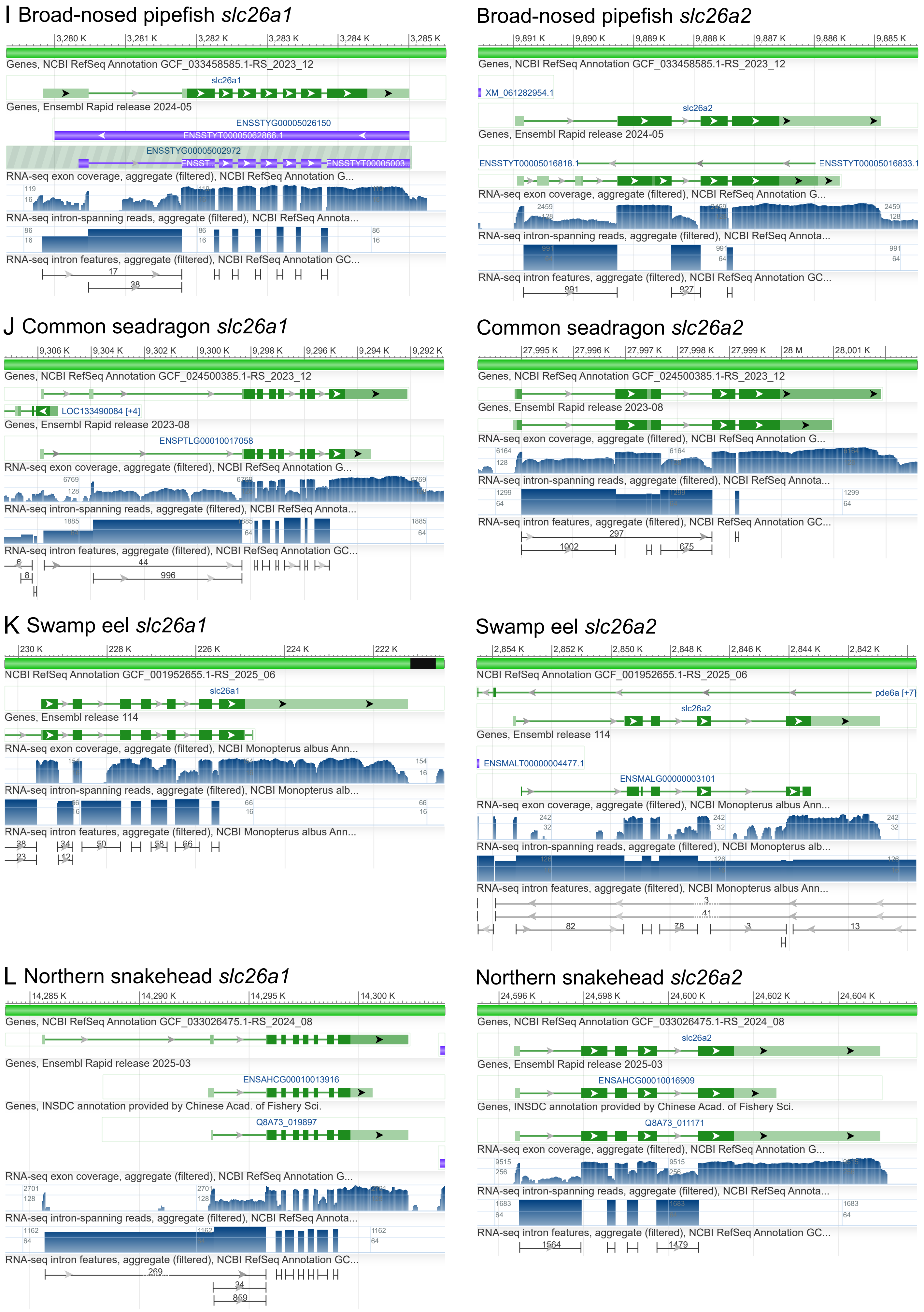

I Broad-nosed pipefish slc26a1
Genes, NCBI RefSeq Annotation GCF_033458585.1-RS_2023_12
slc26a1
Genes, Ensembl Rapid release 2024-05
ENSSTYG00005026150
ENSSTYT00005062866.1
ENSSTYG00005002972
RNA-seq exon coverage, aggregate (filtered), NCBI RefSeq Annotation G...
RNA-seq intron-spanning reads, aggregate (filtered), NCBI RefSeq Annota...
RNA-seq intron features, aggregate (filtered), NCBI RefSeq Annotation GC...
Broad-nosed pipefish slc26a2
Genes, NCBI RefSeq Annotation GCF_033458585.1-RS_2023_12
XM_061282954.1
slc26a2
Genes, Ensembl Rapid release 2024-05
ENSSTYT00005016818.1
ENSSTYT00005016833.1
RNA-seq exon coverage, aggregate (filtered), NCBI RefSeq Annotation G...
RNA-seq intron-spanning reads, aggregate (filtered), NCBI RefSeq Annota...
RNA-seq intron features, aggregate (filtered), NCBI RefSeq Annotation GC...
J Common seadragon slc26a1
Genes, NCBI RefSeq Annotation GCF_024500385.1-RS_2023_12
LOC133490084 [+4]
Genes, Ensembl Rapid release 2023-08
ENSPTLG00010017058
RNA-seq exon coverage, aggregate (filtered), NCBI RefSeq Annotation G...
RNA-seq intron-spanning reads, aggregate (filtered), NCBI RefSeq Annota...
RNA-seq intron features, aggregate (filtered), NCBI RefSeq Annotation GC...
Common seadragon slc26a2
Genes, NCBI RefSeq Annotation GCF_024500385.1-RS_2023_12
Genes, Ensembl Rapid release 2023-08
RNA-seq exon coverage, aggregate (filtered), NCBI RefSeq Annotation G...
RNA-seq intron-spanning reads, aggregate (filtered), NCBI RefSeq Annota...
RNA-seq intron features, aggregate (filtered), NCBI RefSeq Annotation GC...
K Swamp eel slc26a1
NCBI RefSeq Annotation GCF_001952655.1-RS_2025_06
slc26a1
Genes, Ensembl release 114
RNA-seq exon coverage, aggregate (filtered), NCBI Monopterus albus Ann...
RNA-seq intron-spanning reads, aggregate (filtered), NCBI Monopterus alb...
RNA-seq intron features, aggregate (filtered), NCBI Monopterus albus Ann...
Swamp eel slc26a2
NCBI RefSeq Annotation GCF_001952655.1-RS_2025_06
pde6a [+7]
slc26a2
Genes, Ensembl release 114
ENSMALT00000004477.1
ENSMALG00000003101
RNA-seq exon coverage, aggregate (filtered), NCBI Monopterus albus Ann...
RNA-seq intron-spanning reads, aggregate (filtered), NCBI Monopterus alb...
RNA-seq intron features, aggregate (filtered), NCBI Monopterus albus Ann...
L Northern snakehead slc26a1
Genes, NCBI RefSeq Annotation GCF_033026475.1-RS_2024_08
Genes, Ensembl Rapid release 2025-03
ENSAHCG00010013916
Genes, INSDC annotation provided by Chinese Acad. of Fishery Sci.
Q8A73_019897
RNA-seq exon coverage, aggregate (filtered), NCBI RefSeq Annotation G...
RNA-seq intron-spanning reads, aggregate (filtered), NCBI RefSeq Annota...
RNA-seq intron features, aggregate (filtered), NCBI RefSeq Annotation GC...
Northern snakehead slc26a2
Genes, NCBI RefSeq Annotation GCF_033026475.1-RS_2024_08
slc26a2
Genes, Ensembl Rapid release 2025-03
ENSAHCG00010016909
Genes, INSDC annotation provided by Chinese Acad. of Fishery Sci.
Q8A73_011171
RNA-seq exon coverage, aggregate (filtered), NCBI RefSeq Annotation G...
RNA-seq intron-spanning reads, aggregate (filtered), NCBI RefSeq Annota...
RNA-seq intron features, aggregate (filtered), NCBI RefSeq Annotation GC...

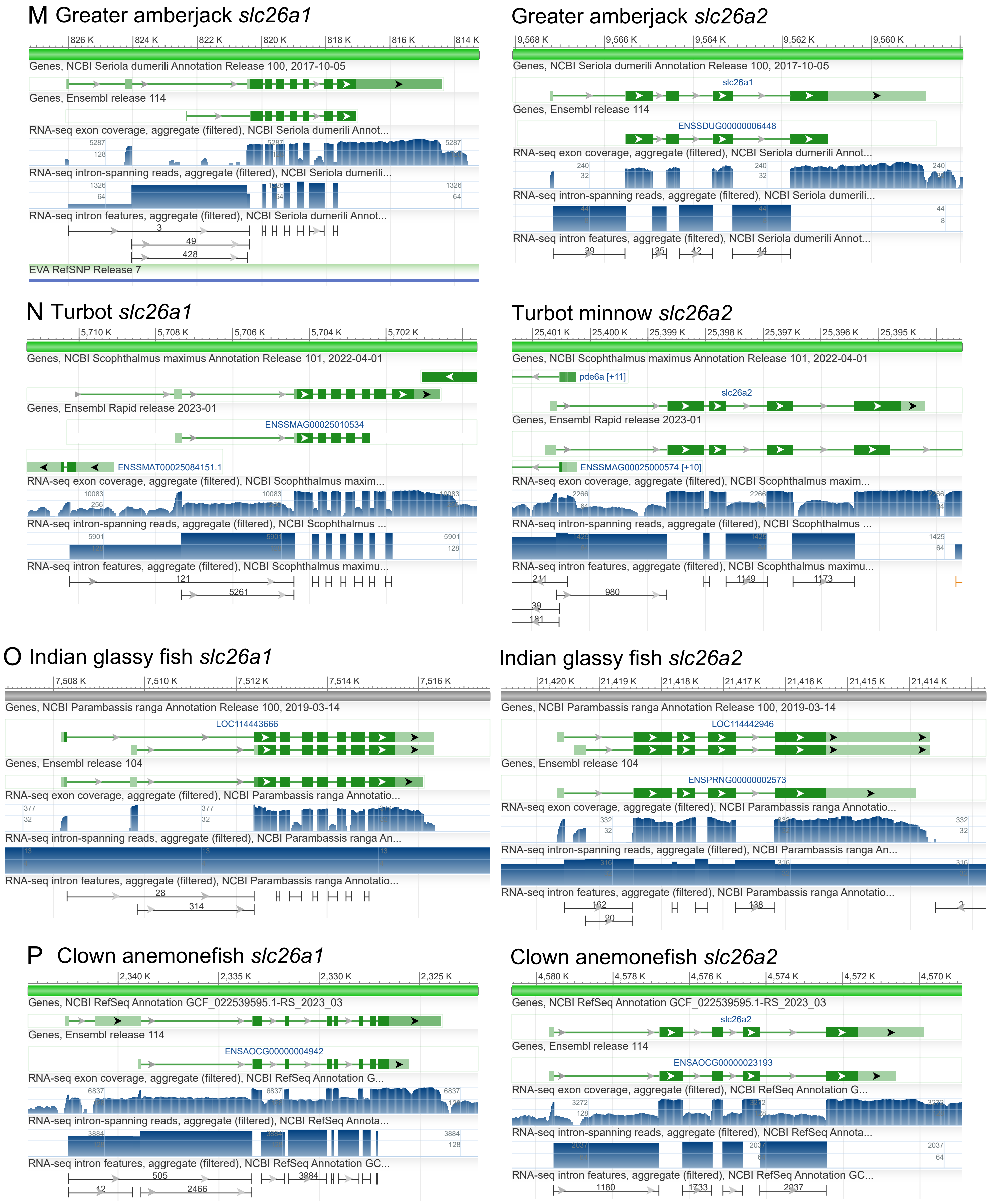

M Greater amberjack slc26a1
826 K 824 K 822 K 820 K 818 K 816 K 814 K
Genes, NCBI Seriola dumerili Annotation Release 100, 2017-10-05
Genes, Ensembl release 114
RNA-seq exon coverage, aggregate (filtered), NCBI Seriola dumerili Annot...
RNA-seq intron-spanning reads, aggregate (filtered), NCBI Seriola dumerili...
RNA-seq intron features, aggregate (filtered), NCBI Seriola dumerili Annot...
EVA RefSNP Release 7
Greater amberjack slc26a2
9,568 K 9,566 K 9,564 K 9,562 K 9,560 K
Genes, NCBI Seriola dumerili Annotation Release 100, 2017-10-05
slc26a1
Genes, Ensembl release 114
ENSSDUG00000006448
RNA-seq exon coverage, aggregate (filtered), NCBI Seriola dumerili Annot...
RNA-seq intron-spanning reads, aggregate (filtered), NCBI Seriola dumerili...
RNA-seq intron features, aggregate (filtered), NCBI Seriola dumerili Annot...
N Turbot slc26a1
5,710 K 5,708 K 5,706 K 5,704 K 5,702 K
Genes, NCBI Scophthalmus maximus Annotation Release 101, 2022-04-01
Genes, Ensembl Rapid release 2023-01
ENSSMAG00025010534
ENSSMAT00025084151.1
RNA-seq exon coverage, aggregate (filtered), NCBI Scophthalmus maxim...
RNA-seq intron-spanning reads, aggregate (filtered), NCBI Scophthalmus ...
RNA-seq intron features, aggregate (filtered), NCBI Scophthalmus maximu...
Turbot minnow slc26a2
25,401 K 25,400 K 25,399 K 25,398 K 25,397 K 25,396 K 25,395 K
Genes, NCBI Scophthalmus maximus Annotation Release 101, 2022-04-01
pde6a [+11]
slc26a2
Genes, Ensembl Rapid release 2023-01
ENSSMAG00025000574 [+10]
RNA-seq exon coverage, aggregate (filtered), NCBI Scophthalmus maxim...
RNA-seq intron-spanning reads, aggregate (filtered), NCBI Scophthalmus ...
RNA-seq intron features, aggregate (filtered), NCBI Scophthalmus maximu...
O Indian glassy fish slc26a1
7,508 K 7,510 K 7,512 K 7,514 K 7,516 K
Genes, NCBI Parambassis ranga Annotation Release 100, 2019-03-14
LOC114443666
Genes, Ensembl release 104
RNA-seq exon coverage, aggregate (filtered), NCBI Parambassis ranga Annotatio...
RNA-seq intron-spanning reads, aggregate (filtered), NCBI Parambassis ranga An...
RNA-seq intron features, aggregate (filtered), NCBI Parambassis ranga Annotatio...
Indian glassy fish slc26a2
21,420 K 21,419 K 21,418 K 21,417 K 21,416 K 21,415 K 21,414 K
Genes, NCBI Parambassis ranga Annotation Release 100, 2019-03-14
LOC114442946
Genes, Ensembl release 104
ENSPRNG00000002573
RNA-seq exon coverage, aggregate (filtered), NCBI Parambassis ranga Annotatio...
RNA-seq intron-spanning reads, aggregate (filtered), NCBI Parambassis ranga An...
RNA-seq intron features, aggregate (filtered), NCBI Parambassis ranga Annotatio...
P Clown anemonefish slc26a1
2,340 K 2,335 K 2,330 K 2,325 K
Genes, NCBI RefSeq Annotation GCF_022539595.1-RS_2023_03
Genes, Ensembl release 114
ENSAOCG00000004942
RNA-seq exon coverage, aggregate (filtered), NCBI RefSeq Annotation G...
RNA-seq intron-spanning reads, aggregate (filtered), NCBI RefSeq Annota...
RNA-seq intron features, aggregate (filtered), NCBI RefSeq Annotation GC...
Clown anemonefish slc26a2
4,580 K 4,578 K 4,576 K 4,574 K 4,572 K 4,570 K
Genes, NCBI RefSeq Annotation GCF_022539595.1-RS_2023_03
slc26a2
Genes, Ensembl release 114
ENSAOCG00000023193
RNA-seq exon coverage, aggregate (filtered), NCBI RefSeq Annotation G...
RNA-seq intron-spanning reads, aggregate (filtered), NCBI RefSeq Annota...
RNA-seq intron features, aggregate (filtered), NCBI RefSeq Annotation GC...

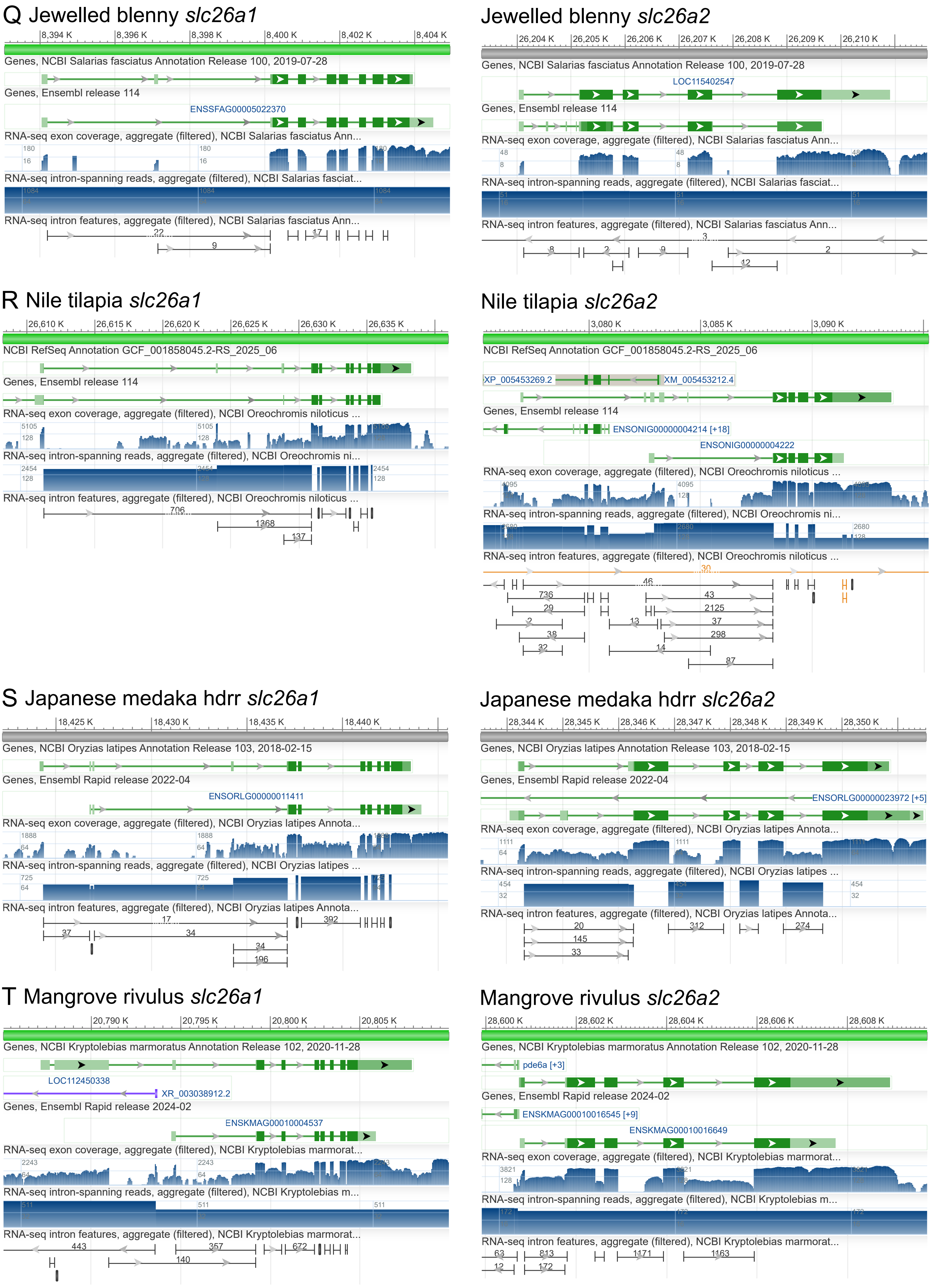

Q Jewelled blenny slc26a1
8,394 K
8,396 K
8,398 K
8,400 K
8,402 K
8,404 K
Genes, NCBI Salarias fasciatus Annotation Release 100, 2019-07-28
Genes, Ensembl release 114
ENSSFAG00005022370
RNA-seq exon coverage, aggregate (filtered), NCBI Salarias fasciatus Ann...
RNA-seq intron-spanning reads, aggregate (filtered), NCBI Salarias fasciat...
RNA-seq intron features, aggregate (filtered), NCBI Salarias fasciatus Ann...
Jewelled blenny slc26a2
26,204 K
26,205 K
26,206 K
26,207 K
26,208 K
26,209 K
26,210 K
Genes, NCBI Salarias fasciatus Annotation Release 100, 2019-07-28
LOC115402547
Genes, Ensembl release 114
RNA-seq exon coverage, aggregate (filtered), NCBI Salarias fasciatus Ann...
RNA-seq intron-spanning reads, aggregate (filtered), NCBI Salarias fasciat...
RNA-seq intron features, aggregate (filtered), NCBI Salarias fasciatus Ann...
R Nile tilapia slc26a1
26,610 K
26,615 K
26,620 K
26,625 K
26,630 K
26,635 K
NCBI RefSeq Annotation GCF_001858045.2-RS_2025_06
Genes, Ensembl release 114
RNA-seq exon coverage, aggregate (filtered), NCBI Oreochromis niloticus ...
RNA-seq intron-spanning reads, aggregate (filtered), NCBI Oreochromis ni...
RNA-seq intron features, aggregate (filtered), NCBI Oreochromis niloticus ...
Nile tilapia slc26a2
3,080 K
3,085 K
3,090 K
NCBI RefSeq Annotation GCF_001858045.2-RS_2025_06
XP_005453269.2
XM_005453212.4
Genes, Ensembl release 114
ENSONIG00000004214 [+18]
ENSONIG00000004222
RNA-seq exon coverage, aggregate (filtered), NCBI Oreochromis niloticus ...
RNA-seq intron-spanning reads, aggregate (filtered), NCBI Oreochromis ni...
RNA-seq intron features, aggregate (filtered), NCBI Oreochromis niloticus ...
S Japanese medaka hdrr slc26a1
18,425 K
18,430 K
18,435 K
18,440 K
Genes, NCBI Oryzias latipes Annotation Release 103, 2018-02-15
Genes, Ensembl Rapid release 2022-04
ENSORLG00000011411
RNA-seq exon coverage, aggregate (filtered), NCBI Oryzias latipes Annota...
RNA-seq intron-spanning reads, aggregate (filtered), NCBI Oryzias latipes ...
RNA-seq intron features, aggregate (filtered), NCBI Oryzias latipes Annota...
Japanese medaka hdrr slc26a2
28,344 K
28,345 K
28,346 K
28,347 K
28,348 K
28,349 K
28,350 K
Genes, NCBI Oryzias latipes Annotation Release 103, 2018-02-15
Genes, Ensembl Rapid release 2022-04
ENSORLG00000023972 [+5]
RNA-seq exon coverage, aggregate (filtered), NCBI Oryzias latipes Annota...
RNA-seq intron-spanning reads, aggregate (filtered), NCBI Oryzias latipes ...
RNA-seq intron features, aggregate (filtered), NCBI Oryzias latipes Annota...
T Mangrove rivulus slc26a1
20,790 K
20,795 K
20,800 K
20,805 K
Genes, NCBI Kryptolebias marmoratus Annotation Release 102, 2020-11-28
LOC112450338
XR_003038912.2
Genes, Ensembl Rapid release 2024-02
ENSKMAG00010004537
RNA-seq exon coverage, aggregate (filtered), NCBI Kryptolebias marmorat...
RNA-seq intron-spanning reads, aggregate (filtered), NCBI Kryptolebias m...
RNA-seq intron features, aggregate (filtered), NCBI Kryptolebias marmorat...
Mangrove rivulus slc26a2
28,600 K
28,602 K
28,604 K
28,606 K
28,608 K
Genes, NCBI Kryptolebias marmoratus Annotation Release 102, 2020-11-28
pde6a [+3]
Genes, Ensembl Rapid release 2024-02
ENSKMAG00010016545 [+9]
ENSKMAG00010016649
RNA-seq exon coverage, aggregate (filtered), NCBI Kryptolebias marmorat...
RNA-seq intron-spanning reads, aggregate (filtered), NCBI Kryptolebias m...
RNA-seq intron features, aggregate (filtered), NCBI Kryptolebias marmorat...

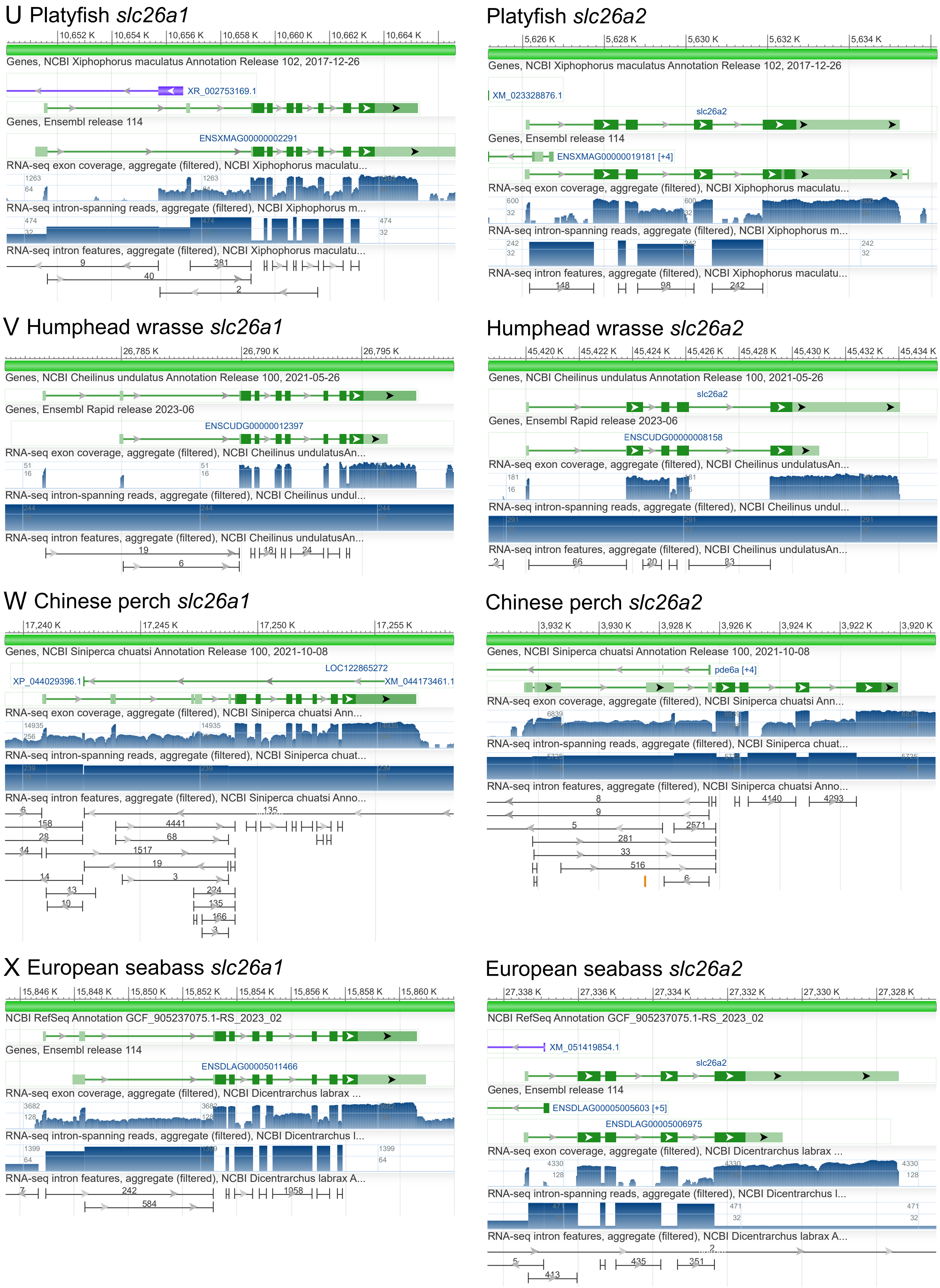
U Platyfish slc26a1
10,652 K
10,654 K
10,656 K
10,658 K
10,660 K
10,662 K
10,664 K
Genes, NCBI Xiphophorus maculatus Annotation Release 102, 2017-12-26
XR_002753169.1
Genes, Ensembl release 114
ENSXMAG00000002291
RNA-seq exon coverage, aggregate (filtered), NCBI Xiphophorus maculatu...
RNA-seq intron-spanning reads, aggregate (filtered), NCBI Xiphophorus m...
RNA-seq intron features, aggregate (filtered), NCBI Xiphophorus maculatu...
Platyfish slc26a2
5,626 K
5,628 K
5,630 K
5,632 K
5,634 K
Genes, NCBI Xiphophorus maculatus Annotation Release 102, 2017-12-26
XM_023328876.1
slc26a2
Genes, Ensembl release 114
ENSXMAG00000019181 [+4]
RNA-seq exon coverage, aggregate (filtered), NCBI Xiphophorus maculatu...
RNA-seq intron-spanning reads, aggregate (filtered), NCBI Xiphophorus m...
RNA-seq intron features, aggregate (filtered), NCBI Xiphophorus maculatu...
V Humphead wrasse slc26a1
26,785 K
26,790 K
26,795 K
Genes, NCBI Cheilinus undulatus Annotation Release 100, 2021-05-26
Genes, Ensembl Rapid release 2023-06
ENSCUDG00000012397
RNA-seq exon coverage, aggregate (filtered), NCBI Cheilinus undulatusAn...
RNA-seq intron-spanning reads, aggregate (filtered), NCBI Cheilinus undul...
RNA-seq intron features, aggregate (filtered), NCBI Cheilinus undulatusAn...
Humphead wrasse slc26a2
45,420 K
45,422 K
45,424 K
45,426 K
45,428 K
45,430 K
45,432 K
45,434 K
Genes, NCBI Cheilinus undulatus Annotation Release 100, 2021-05-26
slc26a2
Genes, Ensembl Rapid release 2023-06
ENSCUDG00000008158
RNA-seq exon coverage, aggregate (filtered), NCBI Cheilinus undulatusAn...
RNA-seq intron-spanning reads, aggregate (filtered), NCBI Cheilinus undul...
RNA-seq intron features, aggregate (filtered), NCBI Cheilinus undulatusAn...
W Chinese perch slc26a1
17,240 K
17,245 K
17,250 K
17,255 K
Genes, NCBI Siniperca chuatsi Annotation Release 100, 2021-10-08
LOC122865272
XP_044029396.1
XM_044173461.1
RNA-seq exon coverage, aggregate (filtered), NCBI Siniperca chuatsi Ann...
RNA-seq intron-spanning reads, aggregate (filtered), NCBI Siniperca chuat...
RNA-seq intron features, aggregate (filtered), NCBI Siniperca chuatsi Anno...
Chinese perch slc26a2
3,932 K
3,930 K
3,928 K
3,926 K
3,924 K
3,922 K
3,920 K
Genes, NCBI Siniperca chuatsi Annotation Release 100, 2021-10-08
pde6a [+4]
RNA-seq exon coverage, aggregate (filtered), NCBI Siniperca chuatsi Ann...
RNA-seq intron-spanning reads, aggregate (filtered), NCBI Siniperca chuat...
RNA-seq intron features, aggregate (filtered), NCBI Siniperca chuatsi Anno...
X European seabass slc26a1
15,846 K
15,848 K
15,850 K
15,852 K
15,854 K
15,856 K
15,858 K
15,860 K
NCBI RefSeq Annotation GCF_905237075.1-RS_2023_02
Genes, Ensembl release 114
ENSDLAG00000011466
RNA-seq exon coverage, aggregate (filtered), NCBI Dicentrarchus labrax ...
RNA-seq intron-spanning reads, aggregate (filtered), NCBI Dicentrarchus l...
RNA-seq intron features, aggregate (filtered), NCBI Dicentrarchus labrax A...
European seabass slc26a2
27,338 K
27,336 K
27,334 K
27,332 K
27,330 K
27,328 K
NCBI RefSeq Annotation GCF_905237075.1-RS_2023_02
XM_051419854.1
slc26a2
Genes, Ensembl release 114
ENSDLAG00005005603 [+5]
ENSDLAG00005006975
RNA-seq exon coverage, aggregate (filtered), NCBI Dicentrarchus labrax ...
RNA-seq intron-spanning reads, aggregate (filtered), NCBI Dicentrarchus l...
RNA-seq intron features, aggregate (filtered), NCBI Dicentrarchus labrax A...

Y Large yellow croaker *slc26a1*

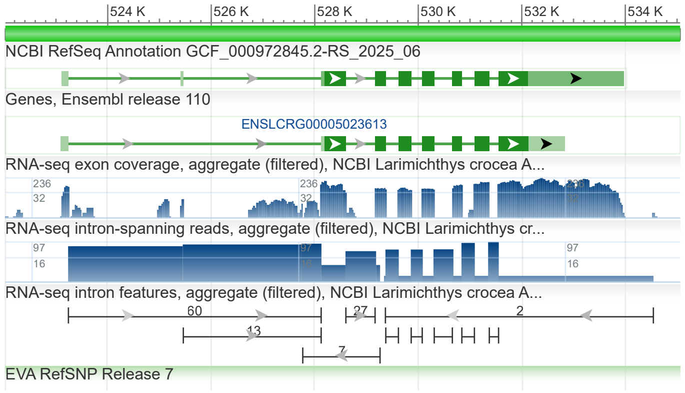

Large yellow croaker *slc26a2*

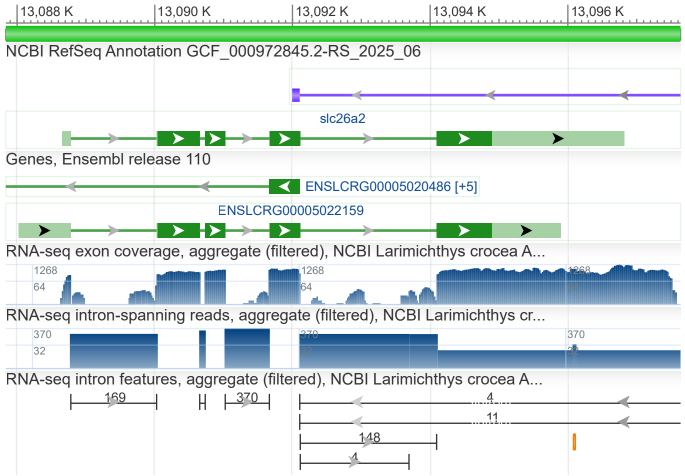

Z Gilthead seabream *slc26a1*

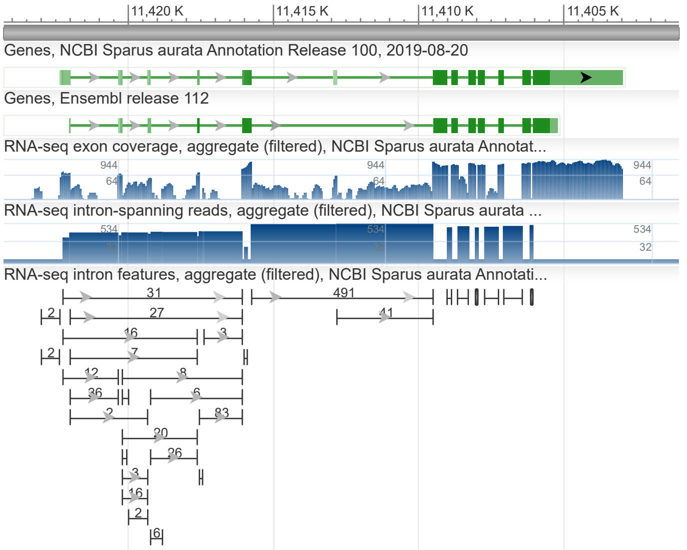

Gilthead seabream *slc26a2*

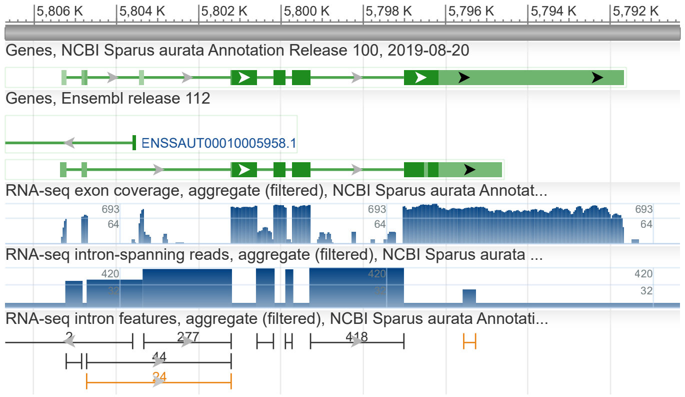

AA Japanese pufferfish *slc26a1*

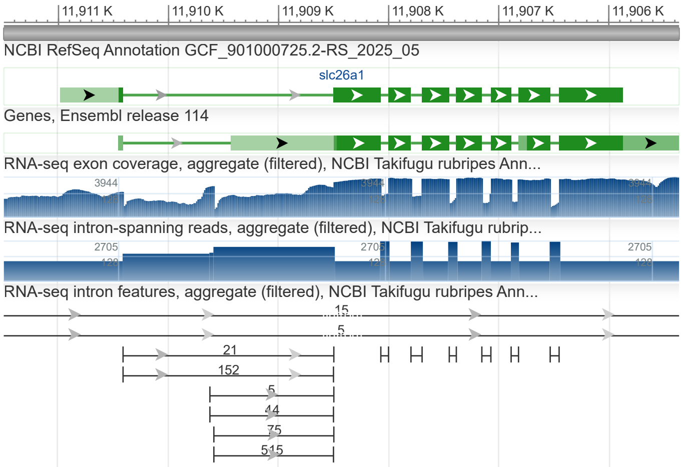

Japanese pufferfish *slc26a2*

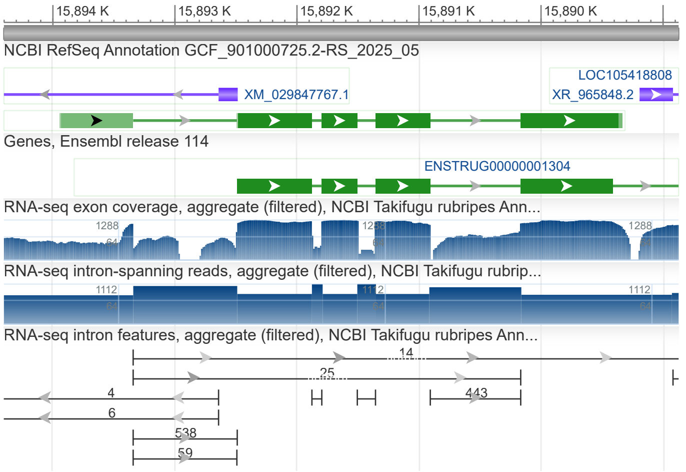

AB Three-spined stickleback *slc26a1*

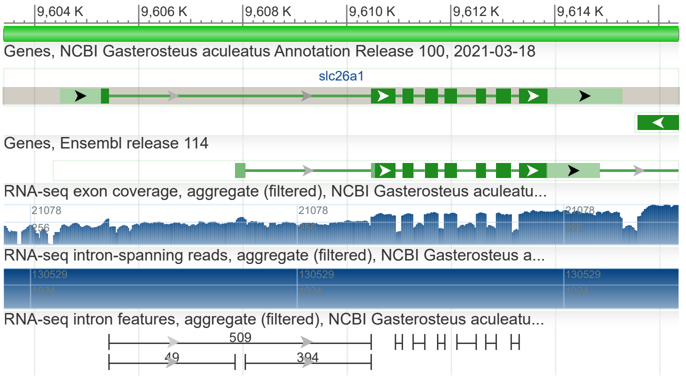

Three-spined stickleback *slc26a2*

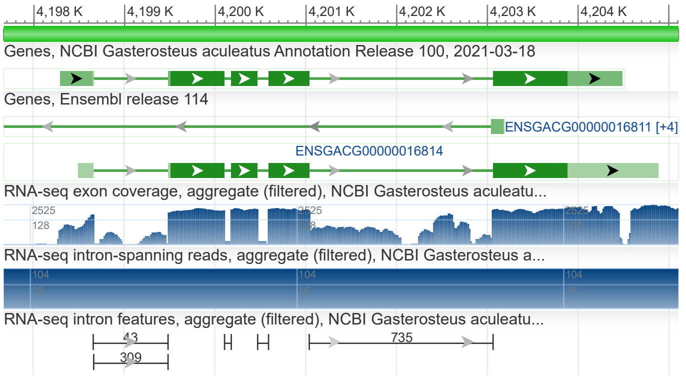

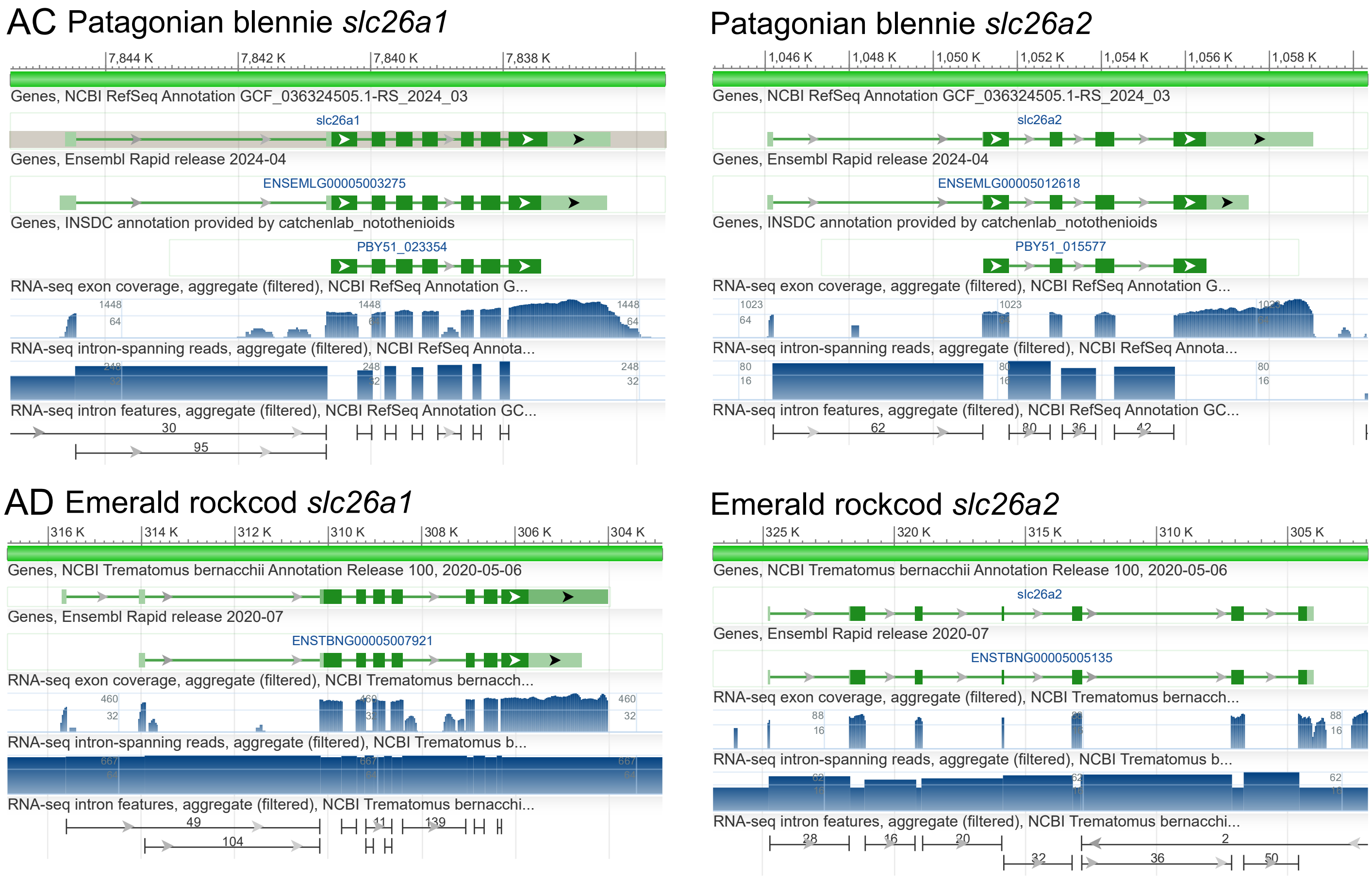

Supplementary Fig. S3 The exon-intron strucutre of *slc26a1* and *slc26a2* in NCBI Genome Data Viewer for the species analyzed in Fig. 4. A-AD) Screen captures of a graphical comparison between predicted exon-intron structure and RNA-seq exon coverage, aggregate (filtered).

Intron insertion sites: Red, phase 0; Green, phase 1; blue, phase 2

```
Peladilla Slc26a1              ---------------------------------------MENHDGVTEILPPPPLLERCARQRRPPLSVLRTRVKQGLTCSVPRVKATLTGFFPVVHWLPKYKLREYVWGDLMSGLIVGIILVPQAIAYCLLAGVDPIYGLYTSFFANIIYFFMGTSRHVSVGIFSLMSLMVGQVVDREV
Atlantic cod Slc26a1           -----------------------------------------MEPQDLPLTDPPQLLERRVRKAQPPLSALRSRVKEEVACSMPRARSTLLGFFPVVSWLPKYKLQEYVWGDLMSGVIVGIILVPQAIAYCLLAGVQPIYGLYTSFFANIIYFFMGTSRHVSVGIFSLMSLMVGQVVDREV
European smelt Slc26a1         ------------------------------------------MEAVMTPRSCVPSLERRARQRKPPLEVLKTKVKQGLACSVPRVRSTLTGFFPVVRWLPRYKLREYAWGDAMSGLIVGIILVPQAIAYCLLAGVDPIYGLYTSFFANIIYFFMGTSKHVSVGIFSLMSLMVGQVVDREV
Human Slc26a1                  ---------------------------------------MDESPEPLQQGRGPVPVRRQRPAPRGLREMLKARLWCSCSCSVLCVRALVQDLLPATRWLRQYRPREYLAGDVMSGLVIGIILVPQAIAYSLLAGLQPIYSLYTSFFANLIYFLMGTSRHVSVGIFSLLCLMVGQVVDREL
European smelt Slc26a2         -------------------MNVNSEYMMATLRPAVDENDSYSVAKDSDLQLAIVLERREKTEEPWNTVFARRLQKHCFCSSAQIAKASVLRFIPILQWLPRYHLKEWLLGDIMSGLIVGILLVPQSIAYSLLAGQEPIYGLYTSFFSSIIYSFLGTSRHVSVGIFGVLCLLVGQVVDREV
Zebrafish Slc26a2              -------------------------------MPTEGSCNNLSAEEDAKREPNLPFRLEECVKEKVSWKEALRKKVKKCSCSTARVKSQIMKSFPIVKWLPRYRFKDWIIGDAMSGLIVGILLVPQSIAYSLLAGQDPIYGLYTSFFANIIYALLGTSRHISVGMFGVLCLLVGQVVDREL
Big-belly seahorse Slc26a2     -----------------------------MMPHEDACCTASGDRTKNEHYEPLILERVPEEAGQRCQVVLSRYFREHCSCSPQKIKSKILDFVPILRWLPHYQLRDWLLGDMMSGLIVGILLVPQSIAYSLLAGQDPIYGLYTSFFSSIIYALLGTSKHISVGIFGVLCLLVGQVVDREL
Emerald rockcod Slc26a2        ------------------------------MTDADDFCPTAEDGAESDQQHPLILQRVQEEP-KSWQTAVSNRLKKNCSCTPKKAKSKILGFVPIVKWLPRYQLKEWILGDLMSGLIVGILLVPQSIAYSLLASQDPIYGLYTSFFASIIYALLGTSRHISVGIFGVLCLLVGQVVDREL
Atlantic cod Slc26a2          ------------------------------------MTLDEEPSDTEDPHHPLVLHRIKKDEEVSWRTSASRQIRGHCSCTKDKARAKLLGFFPILKWLPRYQLRDWILGDVMSGVIVGILLVPQSIAYSLLAGQEPIYGLYTSFFSAIIYAFLGTSRHISVGIFGVLCLLVGQVVEREL
Human Slc26a2                  MSSESKEQHNVSPRDSAEGNDSYPSGIHLELQRESSTDFKQFETNDQCRPYHRILIERQEKSDTNFKEFVIKKLQKNCQCSPAKAKNMILGFLPVLQWLPKYDLKKNILGDVMSGLIVGILLVPQSIAYSLLAGQEPVYGLYTSFFASIIYFLLGTSRHISVGIFGVLCLMIGETVDREL
Inshore hagfish Slc26a2-like   -----------MAEENGLISIPLLNRNIDRPAELSDRN---------PRCLRHTLLHRRPDPPWNLQASLTKKLKNECVCTPRAAKNGLFNLFPVLQWLPKYKIKEMLLGDMSG-LIVGVLLVPQSIAYSLLAGQDPIYGLYSSFFACIIYFILGSSRHISVSIFGVLCLMIGQVVDREL
Sea lamprey Slc26a2-like       --------MGEQENEDSAVNKHLLGPTAEETEMVAVGNGCTESPRGDGGTCVRIRLQVREQAPFNPKVALAKTVANNCCCSPHELKSFFLNLFPVLRWLPKYKVREWILGDFMSGLIVGILLVPQCIAYSLLAGQDPIYGLYTSFFACIIYFLMGTSKHISVGIFGVLCLMIGQVVDREL
Inshore hagfish Slc26a12-like  -------------------MEENNEFSLKNSNKSSENTFSDSFDVEDGISHVCIQLEKQDQPQVTFLERVKMTAEKHLTCSPSCFKKVIFDIFPVLQWLPNYKFREYIIGDIMSGIVVGIISVPQSIAYSLLASVDPIYGLYASFFPCIIYFLFGTSRHTSIGIFAVLCLMIGQVVDQEL
Chicken Slc26a12               -----------------------MEDKSSQTSGLSKDVQSSLTPEEPPVDFMHIKLEENEPIDVRTKDFILKKAREVCKCNHQTIITFFCQLFPVLDWLPRYNIKTQLLGDIISGLLVGIVAIPQSISYSLLANQDPIYGIYTNFFCSIIYVAMATSRHNSVGSFGVLCLMIGQSVNRHL
Human Slc26a3                  -----------------------------------MIEPFGNQYIVARPVYSTNAFEENHKKTGRHHKTFLDHLKVCCSCSPQKAKRIVLSLFPIASWLPAYRLKEWLLSDIVSGISTGIVAVLQGLAFALLVDIPPVYGLYASFFPAIIYLFFGTSRHISVGPFPILSMMVGLAVSGAV
                                                                                                   ..        .    .*    **   *   :     .*  .  :  *:: : *  :::.**..   *:*.:*:.**   :**   :.:*:* *:.  *  ::.:::*  *.   :

Peladilla Slc26a1              YLAGFDMSDGSTVTMAPGMW-NDTGPGDTNLTAAGVEVMGMECGKECYAISVAAATTFLAGIYQVLMAVFRLGFVSVYLSAPMLDGFATGASFTILTVQAKYLLGLKIARHQGYGTVVVTWVRILSNIHHTNLCDLVTSAICIFVLVAGKELQERFKDRLKIPIPTELVVVLGATLASHF
Atlantic cod Slc26a1           FQAGFDLSDTD-----PGSF-NGTG-VASNLSAATVEVMGVVYSKECYAIAIAAALTLLAGIYQVLMAVFRLGFVSVYLSAPMLDGFATGASLTILTVQAKYLLGLKIPRHQGYGTVVVTWINIFANIQNTNLCDLVTSAICIFVLVAGKEIQDRYKSRLKIPLPTELVVVAGATLASHF
European smelt Slc26a1         FLAGYDLNEDG------------ASAVLNDSLLVNGTAGGLKGEKEYYAISIAAAVTFLAGIYQVLMAVFRLGFVSVFLSAPMLDGFATGASFTILTVQAKYLLGLKIPRYQGYGTVVVTWVNIFSNIHHTNLCDLITSAICIFVLVAGKELQERYKDRLKIPLPTELLVVAGATLTSHF
Human Slc26a1                  QLAGFDPSQDG-------------LQPGANSSTLNGSAAMLDCGRDCYAIRVATALTLMTGLYQVLMGVLRLGFVSAYLSQPLLDGFAMGASVTILTSQLKHLLGVRIPRHQGPGMVVLTWLSLLRGAGQANVCDVVTSTVCLAVLLAAKELSDRYRHRLRVPLPTELLVIVVATLVSHF
European smelt Slc26a2         AAAGYITEGYSNKTTSF-----ILGRLGN-----DTAGAGLVCDKSCYAIMVGATVTFTAGVYQVMMGLLQVGFVSVYLSDSLLSGFATGASLTILTSQLKYLLGLKLPRAQGWFTLIKTWFSLLQNMADTNLCDLFTSLLCLLVLVPTKELNERFKTRLKAPIPWELFVVIAATVASHF
Zebrafish Slc26a2              TLAGYSSDIN-------------QTTLGN-----VDNSTGPICDRSCYAIMVGATLTFTAGIYQVLMGLLQIGFVSVFLSDSLLSGFATGASLTILTSQIKYFLGLHLPRVQGWGSLIKTWISLFKNLGHTNLCDLITSLICLLVLVPAKELNDRFKAKLKAPIPFELFVVIAATLASHF
Big-belly seahorse Slc26a2     AVAGYLVDGGVGGNG--------SANLRG-----SNSSMVEACDRSCYAITVGATVTFIAGVYQVLMAIFQVGFVSVYLSNSLLSGFATGASLTILTSQVKYMLGLKITRPQGWFTFFKTWFSVLANLGNSNLCDLITSLVCLLLLLPTKELNDRYKSKLKAPIPFELFVVIIATLASHF
Emerald rockcod Slc26a2        ALAGYLPENSILSSN--------DSSILL-----GNSSFGMDCDRSCYAITVGATVTFTAGVYQVLMGIFQVGFVSVYLSDSLLSGFATGASLTILTSQFKYLLGLKIPRPQGWFTLFKTWYSVLSNLQNTNACDLVTSLVCLMILLPTKELNDRFKAKLKAPIPFELFVVVIATLASHF
Atlantic cod Slc26a2           AAAGYPSDTVLVAGGGYNT--SAALLVAQ-----GNSSLGRACDKSCYAIVVGATVTFTAGVYQLLMGLLQVGFVSVYLSDSLLSGFATGASLTILTSQLKYLLGLRIPRPQGWFTLIKTWYALLTNLANTNVCDLVTSLVCLLVLVPSKELNDRFKAKLKAPIPFELFVVIIATLASHV
Human Slc26a2                  QKAGYDNAHSAPSLG------MVSNGSTL-----LNHTSDRICDKSCYAIMVGSTVTFIAGVYQVAMGFFQVGFVSVYLSDALLSGFVTGASFTILTSQAKYLLGLNLPRTNGVGSLITTWIHVFRNIHKTNLCDLITSLLCLLVLLPTKELNEHFKSKLKAPIPIELVVVVAATLASHF
Inshore hagfish Slc26a2-like   IVAGFTSADDSQAHEKLNY--SESHEN--------VTFGGVVYTRSDFAILVGTTVTFVAGVYQITMGIFQLGFIAIYLSEPMLSGFVTGASFVILTSQVKYMLGLHIPRTGGPGSLLLTWYYILKNIGHTNICDLITSAICLFIMVPIKEVNERYKKKMKAPFPIDLIVIIFATLVSHF
Sea lamprey Slc26a2-like       QVAGFAEEGIKGHDHVVNN--SMNFSNSDEENGFTVMIGGLPCGRSCFAIAVGTTVTFMAGVYQVVMGIFQMGFVSIYLSEPMLSGFVTGSSLTVLTSQMKYLLGIHIPRASGAGALLKTWYHIFSQISNTNVCDVVTSIICILVLVPAKELNEHFKARLKAPFPIELVVVVAATLASHF
Inshore hagfish Slc26a12-like  QEAGYDLSVNTSNIHRYSNTEKLEYEMVSNLSTPFPTLVSNNCDRSCYAIRIAANITFLAGVYQVVMSILQLGFISVYLSEPLLSGFVTGSSITILTSQAKYLLGLPIPRYKGIGALVMNWVSIFKMIPNTNICDLVTSIICFLVMVPIKEMNIRYKHKMKTPFPIELLVVIVATLISHY
Chicken Slc26a12               QLAGYDDNTDSLLVG----------------NATSSGNGTGTCDRSCYAITVALSLSFLVGLYQILLGVLQLGFLSVYLSEPLLSGFVAGSSLTIITSQMKYLLGLNIPRHEGVGSFILTWVDLFRYIPNTNICDLVTSLVALAIIIPVKMINDRYKDKMKAPVPIELLVVIIATVVSYY
Human Slc26a3                  SKAVPDRNATTLGLP-------------------NNSNNSSLLDDERVRVAAAASVTVLSGIIQLAFGILRIGFVVIYLSESLISGFTTAAAVHVLVSQLKFIFQLTVPSHTDPVSIFKVLYSVFSQIEKTNIADLVTALIVLLVVSIVKEINQRFKDKLPVPIPIEFIMTVIAAGVSYG
                                 *                                           .    :    .    :.   *: *: :..:::**:   :** .::.**.  .::.  ::.  * *.:: : :.    .     ..       ::     .:* .*:.*: : : ::    * :.  ::: ::   *.* ::.:    *:  *:

Peladilla Slc26a1              GDLHGLYGSSVSGHIPTGFISPTVPSFGLMPRVALDAVPLAVISFAFTVSLSEMFAKKNGYTVRPNQEMLAIGCCNIIPSFFHCFTTSAALAKTMVKDSTGCQTQVSSLVSAVVVLLVLLFFAPFFYALQKCVLACIIIVSLRGALRKILDVPSKWRASRADAVVWLVAMGATALISVEL
Atlantic cod Slc26a1           GNLNALYGSSVSGHIPTGFIPPTVPALDLMHRVALDAVPMAVISFAFTVSLSEMFAKKNGYTVRPNQEMLAIGCCNIIPSFFHCFTTSAALAKTMVKDSTGCQTQVSSLISALVVLLVLLFFAPYFYSLQKCVLACIIIVSLRGALRKFRDVPAKWRVSRADAVVWLVAMAATVLISVEI
European smelt Slc26a1         GDLHGRYDSSVSGHIPTGFIPPTVPNFGLMPRVALDAIPLAVISFAFTVSLSEMFAKKNGYTVRPNQEMLAIGCCNIIPSFFHCFTTSAALAKTMVKDSTGCQTQVSSLVSALVVLLVLLFFAPYFHALQKCVLACIIIVSLRGALRKFRDVPSKWRASKTDAVVWLVTVAASALITVEL
Human Slc26a1                  GQLHKRFGSSVAGDIPTGFMPPQVPEPRLMQRVALDAVALALVAAAFSISLAEMFARSHGYSVRANQELLAVGCCNVLPAFLHCFATSAALAKSLVKTATGCRTQLSSVVSATVVLLVLLALAPLFHDLQRSVLACVIVVSLRGALRKVWDLPRLWRMSPADALVWAGTAATCMLVSTEA
European smelt Slc26a2         GCFQEVYGSDVAGTIPTGFLPPQMPSWSLIPAVAVDAFSIAVVGFAITVSLSEMFAKKHGYQVDPNQEMYAIGFCNILPSFFRCFTTSAALTKTLVKESTGCKTQLSGLVTALVLLLVLLLIAPLFYSLQKCVLAVIIIVNLSGALRKFTEIPQMWHVNRVDASIWLITAATSALINTEL
Zebrafish Slc26a2              GHFKDTYGSDVAGTIPTGFMAPQSPNLSLVPNIAIDALSIAIVGFAITVSLSEMFAKKHGYIVNANQELNAIGNCNIISSYFHCFTVSAALTKTLVKESTGCHTQISGLVTALVLLLVMLVIAPAFYSLQKCVLAVIIVVNLRGALRKFGDIPQMWRVNRVDTVIWLLTMATSALVNTEL
Big-belly seahorse Slc26a2     GRFNGDYGSDVAGEIPTGFLPPQLPAWSLIPNVAMDAFSIALVGFAITVSLSEMFAKKHGYRVDANQEMYAIGFCNILPSFFRCFTTSAALTKTLVKESTGCQTQVSGLVSAVVLLLVLLVIAPLFYSLQKCVLAVIIVVNLRGALRKFADVPSMWHTNRIDAAIWLITMATSALINTEL
Emerald rockcod Slc26a2        GNFNTDYGSGVAGTIPTGFLPPQMPLWSLIPNVAVDAFSIAIVGFAITVSLSEMFAKKHGYTVDANQEMYAIGFCNILPSFFRCFTTSAALTKTLVKESTGCQTQISGIISALVLLLVLLVIAPLFYSLQKCVLAVIILVNLRGALRKFLDIPSMWRVNRVDTSIWLITMATSALVNTEL
Atlantic cod Slc26a2           GRFNAEYGSGVAGAIPTGFLPPQLPDWSLIPSVAVDAFSIAVVGFAITVSLSEMFAKKHGYAVDANQEMYAIGFCNVLPSFFRCFTTSAALTKTLVKESTGCQSQVSGLVTALVLLLVLLFIAPFFYSLQKCVLAVIIVVNLRGALRKFADVPRMWLANRIDACVWLITMATSALVNTEL
Human Slc26a2                  GKLHENYNSSIAGHIPTGFMPPKVPEWNLIPSVAVDAIAISIIGFAITVSLSEMFAKKHGYTVKANQEMYAIGFCNIIPSFFHCFTTSAALAKTLVKESTGCHTQLSGVVTALVLLLVLLVIAPLFYSLQKSVLGVITIVNLRGALRKFRDLPKMWSISRMDTVIWFVTMLSSALLSTEI
Inshore hagfish Slc26a2-like   GKLEENFDSSIAGEIPIGFKPPRAPSWTLVPSVLVDGIPIAIIGFAITVSLSEMFAKKHGYTIRPNQEVIAIGCCNLIPAFFYSFTTSAALAKTLVKEATGCQTQVSGLITAMCLLLVLLVIAPLFYSLQKCVLAIVTVVNLRSAFRKFLDIPKMWQENCIDTIIWFITMLTSALISPEL
Sea lamprey Slc26a2-like       GGFKENYSSSIAGHIPTGFLPPRAPEWTLIPRIAADAVPIAIIGFAITVSLSEMFAKKHGYTVRANQEMIAIGFCNIVPAFFYSFTTSAALAKTLVKEATGCQTQMSAVVTAGVLLLVLLLIAPLFYSLQKCVLGVITIVNLRGAFRKFLDIPRMWQESRVDTSIWFVTMLASALISTEI
Inshore hagfish Slc26a12-like  VNLQERYGSNITGTISTGFQVPRLPEWNLVQSVAADTVSIAIVSFVFTISLSEIFAKKHGYVVRANQEMLAIGCCNIIPSFFYSFATCATVTKTLLKDA TGCMTQVSSLITAAVILLVLMVIAPLFHSLQNCVLATIIIVNLRGAFQKFFDTPRMWRVSHADALVWWVTMLASALLSTEV
Chicken Slc26a12               FKFEERYKSSVCGAIPTGFRKPTLPDTKLFSSLAVDALPIAIIGFAMTVSLAEIFGKKHGYAVRANQEMIAIGMCNLVPSFFYCFASSAALTKTLLKESTGTQTQLSSLVTSLVLLLVLLWIAPLFYSLQTAILGVVTIVNLRGGLRTFCETPRMWQLSKLDTAVWWTTMLASTLITTEI
Human Slc26a3                  CDFKNRFKVAVVGDMNPGFQPPITPDVETFQNTVGDCFGIAMVAFAVAFSVASVYSLKYDYPLDGNQELIALGLGNIVCGVFRGFAGSTALSRSAVQESTGGKTQIAGLIGAIIVLIVVLAIGFLLAPLQKSVLAALALGNLKGMLMQFAEIGRLWRKDKYDCLIWIMTFIFTIVLGLGL
                                 :.   :    : * :  **  *  *    .      * .  ::::.  ..:.*::.::.  .  .* :   ***: *:*  *:: .  :   *: .:::::: :: :**  :*::.:: :  :*:*:: :.   :   ** .:*.  :  : .*  .  :  .  :     *  .   *  :*  :     ::
```

```
Peladilla Slc26a1             GLVVGVVFSMLCVIVQTQRPKVALLGRVAD-TDFYEDIKEYQNLTAPEG-VQIFRFQAPLYYANKDSFLKALYKAVGLEPFLESTRRRKAEKKARELASKLPKSAGENNNSNGDVSTSLVVGEN------------------------------------KFHTIILDCSAMPFMDSTGM
Atlantic cod Slc26a1          GLVVGVVFSMVCVIFVTQNPKVSLLGRSED-SELYEDLDEYENLAAPAG-VRVFRFQAPLYYANKDSFLRTLYETVGVDPFLEMTSRRKRDKKATKRLAAKQAGEDSENRKNGKLCVGLVRREL------------------------------------EFHTIVLDCSAIPFIDSAGM
European smelt Slc26a1        GLLVGVVFSMICVIFQTQNPKVSLLGQAGT-SELYEDIAEYKDLSAPSG-VRIFRFQAPLYYANKETFLKSLYKAVGLEPFLELTRRRKAQKKS--LLAAKQAEASRGDKTNGDAIVGLVNADLL------------------------------------FHTIVLDCSSVPFIDSAGM
Human Slc26a1                 GLLAGVILSLLSLAGRTQRPRTALLARIGD-TAFYEDATEFEGLVPEPG-VRVFRFGGPLYYANKDFFLQSLYSLTGLDAGCMAARRKEGGSETGVGEGGPAQGEDLGPVSTR---AALVPAAA------------------------------------GFHTVVIDCAPLLFLDAAGV
European smelt Slc26a2        GLLVGVLVSAFCVLVRTQRAQGLELGRVGT-SELYEDLAGYHGIQSQPG-IAIFRYESPIYYANQSLFKKCLYHRLGLDPVKEKVHRKKLEKNRKREGQMRAGENRTKGQEAEMTTNVFLPVKS------------------------------------SFHSVVIDCSPVLFLDTAGV
Zebrafish Slc26a2             GLLVGVVVSAFCVLGRTQFAQALQLGQAGD-RELFEDIASYNGLQTQPD-VAVFRYEAPIYYANQSLFKKSLYRNVGLDPLKEKAKQRKLEKQRKQ-----KHSREDQKQEMDASTNVNLLQHS------------------------------------SFHTLVIDCSSVLFLDTAGV
Big-belly seahorse Slc26a2    GLLVGILVSAFCVLGRTQRAQAQELGRTSD-RDHYEVLSAYGGLHAHPG-VAVFRYPAPIYYANQSLFKKSLYQSAGLDPVKEKSRRIKFEKKFKRQNTEPELPCKEDGEGTAGITLSLD--------------------------------------QEPLRSLVLDCSAVFFLDSAGV
Emerald rockcod Slc26a2       GLLVGVLVSALCVLARTQQAKVLKLGRASN-REYYEDVSSYRGLQTHPG-VAVFRYDAPIYYANQSLFKKSLYKSAGLDPLKEKSRVMKFKNKKEKQQDVPDVKPTHEEEAGVTLME-----------------------------------------NKSLRSVVIDGSSISFLDTAGV
Atlantic cod Slc26a2          GLVVGVLVSAFCVLGRTQRAQAVALGRSSSGRELYEDLQAYEGLRAAPGGVAVFRYQAPVYYANQSLFKSSLFRAAGLDPVKEKARRRKLEKKKKDRSDPGKEEEAAATTADVAALGRSDDAENGAVGKQGAGESDAGVTKVLMPAGKRKEET---APGGGVHSVVLDCSAVLFLDTAGV
Human Slc26a2                 GLLVGVCFSIFCVILRTQKPKSSLLGLVEE-SEVFESVSAYKNLQIKPG-IKIFRFVAPLYYINKECFKSALYKQT-VNPILIKVAWKKAAKRKIKEKVVTLGGIQDEMSVQLSHDP------------------------------------------LELHTIVIDCSAIQFLDTAGI
Inshore hagfish Slc26a2-like  GLLIGICISLLAFIIRTQWPRAVLLGRVEGTTDCYADLEAYTNVSEVKG-VRIFSFSAPLYYANKDNFSTKLHSKTAVNPELVVAWRKNESKESKQKDEQSDTNG----------VIIVPDP------------------------------------FDFHTLVLDFSSVNFLDMVGT
Sea lamprey Slc26a2-like      GLLVGVCVSLLVFIVRTQRPRAALYGCVHDTNNFYADLEAYSNVAEVPG-VRVFHFDAPLYYANKEYFKSQLFLKTRVNPALEVAAQKKRQEKLQKRKKLSGSDNGEIVNKQAMLEFNKLLPT------------------------------------FDFHTIVIDCSAMQFMDSVGV
Inshore hagfish Slc26a12-like GLLVGICFAAVCILVRMQRPSIIQLGRVPD-TKQYADLNLYRGLLCVSD-VVIFRFNAPLYYVNKNYFQAAIFCKSGLNPTLKVKKNAKDKPEETENEDNGFSKCN----------------------------------------------------TRKIGALVVDCSAMHFVDSPGA
Chicken Slc26a12              GLLVGVCFALLCIIFRTQRPRAALLGKVSN-TEIYEDQSAYKQLSSIAN-IKIFRFGSSLYYANKDYFKTALYQKTGVNPILLAARHQRAVTQAKADTGNRKSVFNSVFGCLKPSKKHMGKSPTDVC-----------------------------LPSLDMHTLILDCGAMQFIDTTGL
Human Slc26a3                 GLAASVAFQLLTIVFRTQFPKCSTLANIGR-TNIYKNKKDYYDMYEPEG-VKIFRCPSPIYFANIGFFRRKLIDAVGFSPLRILRKRNKALRKIRKLQKQGLLQVTPKGFICTVDTIKDSDEELDNNQIEVLDQPINTTDLPFHIDWNDDLPLNIEVPKISLHSLILDFSAVSFLDVSSV
                              **  .:.  . .     *.     .         :      :  :     . : :.    ..:*: *    *    :       ...          .    .                                                   .  ::::* ..: *:*  .

Peladilla Slc26a1             GTFKGLVKDYSEVGVNLLLASCNTNVIDTLRKGLFFGKG--DKDMDSMLFYTIHAAVLYANSQVAPPALPADSAV-----------------------------
Atlantic cod Slc26a1          GAFAGLVKDYGELGVAVLLAGCNTPVIDALRRGAFFGEK--DRDMSLRLFYTVHAAVLSARSRAAAEGHTAGDSQV----------------------------
European smelt Slc26a1        GTLKAVLKEYQEVDVSVLLAVCNTNVIDTLRRGSYFGKN--DKDIDSLLFHTVHSAVLYASEVANTADKAAGDTWPGETAEDSVV-------------------
Human Slc26a1                 STLQDLRRDYGALGISLLLACCSPPVRDILSRGGFLGEGPGDTAEEEQLFLSVHDAVQTARARHRELEATDAHL------------------------------
European smelt Slc26a2        NSLKEVYEDYKELGVKVLLAQCNFSVRASLQRGGYYPDNDG---EREHVFFTISDAVHYAQCLSGENGACDPHC------------------------------
Zebrafish Slc26a2             NALKEVFKDYKELGVNVLLAQCNTSVIDSLRRGDYYDPNKG---TKDIQFHTIGEAILYGQSLKSQNGNCDTSV------------------------------
Big-belly seahorse Slc26a2    NALKEVHSDYGELGVAVVLAQCNTAVLDALRRGGYFSEKK--GEDDRLTFFTIADAVRHLQRAFVPNGTC----------------------------------
Emerald rockcod Slc26a2       NALKEVRKDYAELGVKVLLAQCNTSVLDSLQRGGYYPKTESDGGEETVIFYTIEDAVHYVQNLSAANGDHDSKC------------------------------
Atlantic cod Slc26a2          NALKEVRKDYQDVGVRLLLARCSASVLDTLERGGYSPLNAD---REEPVFFSIGDAVRYAQNVSNGGEDKCDTYC-----------------------------
Human Slc26a2                 HTLKEVRRDYEAIGIQVLLAQCNPTVRDSLTNGEYCKKEEE-----NLLFYSVYEAMAFAEVSKNQKGVCVPNGLSLSSD------------------------
Inshore hagfish Slc26a2-like  GVVKSTCSDYQTIGLRVLIAGCNPSVLDSLSRARFIDGNDE-----EVLFFSVHEAVEHALARHSG--------------------------------------
Sea lamprey Slc26a2-like      GAVKSIMKDYQSIGIRVILACCNPTVMDSLHRGKCISTIED-----NLTFCLIHDAVLYAEAELIQLFVANMVI------------------------------
Inshore hagfish Slc26a12-like DVLQELHQAYGRIGVLFLLANCSPTILETLQQSWILQAICK---------------------------------------------------------------
Chicken Slc26a12              SVLKETHRDFQELGVQLLLANCNPLLRRRLRDGGWAAGAHGGQLAFHSVHHAVQFAQQWHREQQESKERRDADPDPEDMSVQASL-------------------
Human Slc26a3                 RGLKSILQEFIRIKVDVYIVGTDDDFIEKLNRYEFFDGEVKS----SIFFLTIHDAVLHILMKKDYSTSKFNPSQEKDGKIDFTINTNGGLRNRVYEVPVETKF
                                .     :  : : . :.  .  .    *
```

Supplementary Fig. S4. Multiple alignment and comparison of intron positions

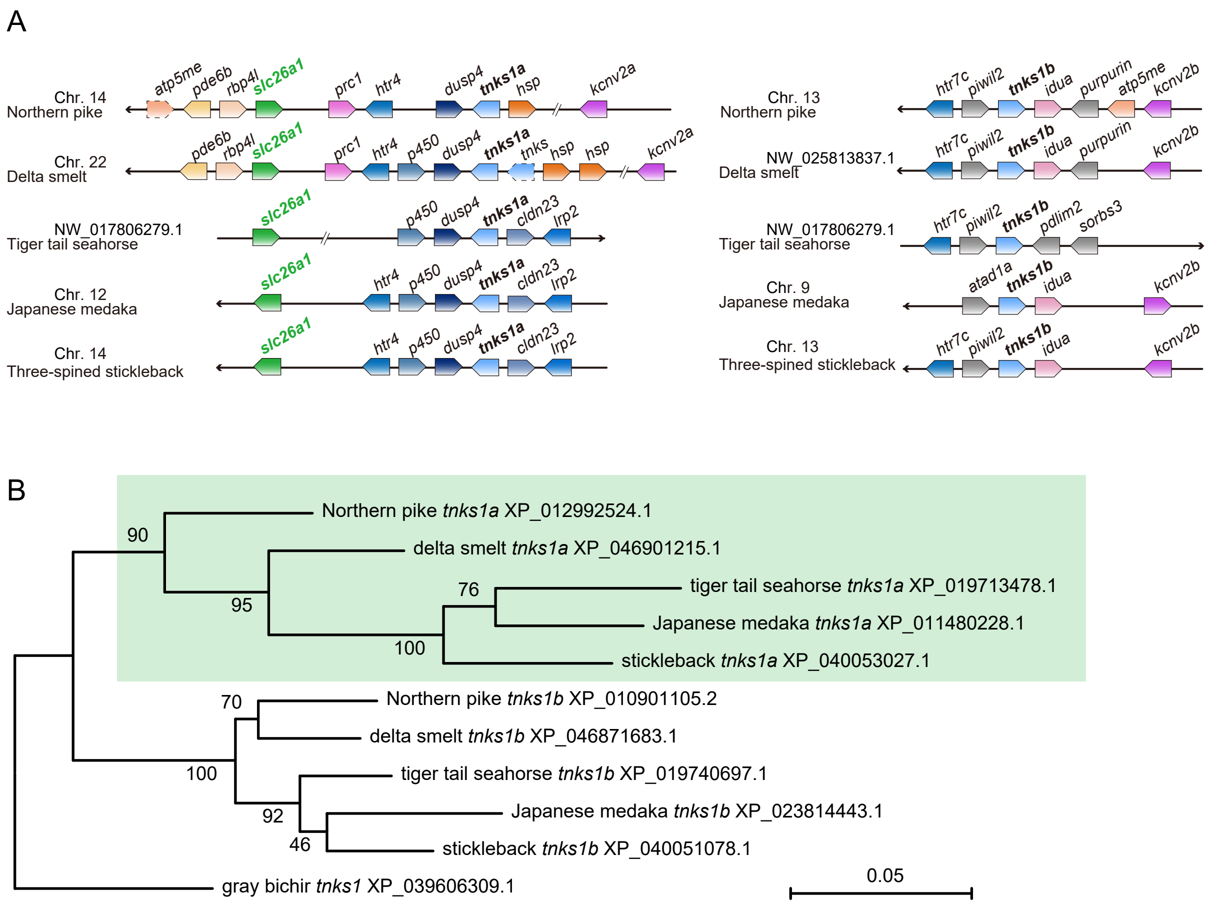

Supplementary Fig. S5. Conserved synteny of *slc26a1* in the teleost specific whole-genome duplication. (A) synteny and (B) phylogenetic analyses of *tnks1* ohnologs, *tnks1a* and *tnks1b*. In teleost, *slc26a1* is always present in the locus near *tnks1a* but not *tnks1b*.
